# Palladium-Catalyzed Decarboxylative Asymmetric Allylic Alkylation of Thietane 1,1-Dioxides

**DOI:** 10.1021/acs.orglett.1c04075

**Published:** 2021-12-16

**Authors:** Gillian Laidlaw, Vilius Franckevičius

**Affiliations:** Department of Chemistry, Lancaster University, Bailrigg, Lancaster LA1 4YB, U.K.

## Abstract

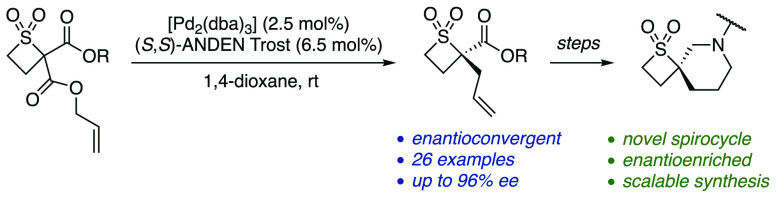

A palladium-catalyzed decarboxylative asymmetric allylic alkylation of thietane 1,1-dioxides via linear enolate intermediates from racemic starting materials has been developed. This process installs an α-sulfonyl tetrasubstituted stereogenic center with high enantioselectivity. The potential to transform the alkylated products to novel types of enantioenriched spirocycles for medicinal chemistry applications has also been demonstrated.

Four-membered-ring-containing spirocycles have become particularly attractive building blocks in drug discovery,^1^ with much attention placed on the development of synthetic routes to achiral (**1**, Scheme 1A),^2^ as well as chiral but racemic (**2**) spirocycles.^3^ In contrast, chiral, enantiopure analogues **3** are much less common.^4^ In particular, there are no examples of enantiopure thietane 1,1-dioxide containing spirocycles **4**.

**Scheme 1:**
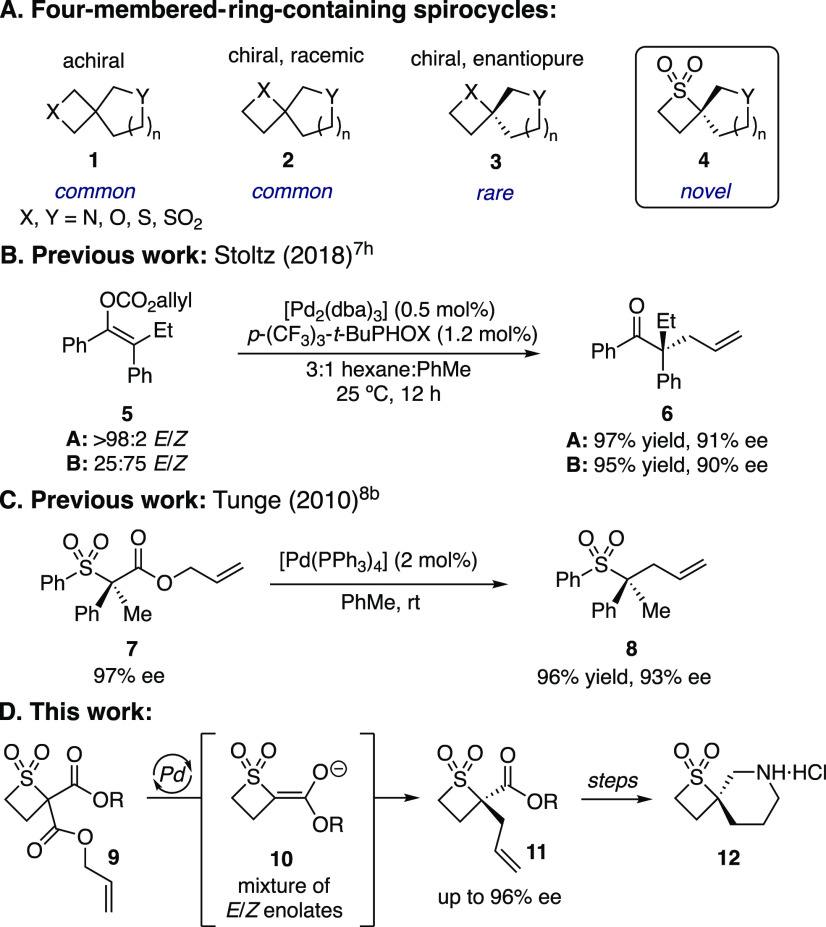
Introduction

To access tetrasubstituted carbon centers in enantioenriched form, we sought to utilize the palladium-catalyzed decarboxylative asymmetric allylic alkylation (Pd-DAAA) reaction,^5^ most frequently employed in the asymmetric alkylation of cyclic enolates.^6^ However, the DAAA reaction of linear enolates has been less developed due to the need for stereoselective enolization of the carbonyl substrates in order to achieve high levels of asymmetric induction in the alkylation step.^7^ The Stoltz group discovered that the Pd-DAAA reaction of linear enol carbonate **5** gives **6** with high enantioselectivity irrespective of the ratio of *E*/*Z* enol carbonates **5** due to a palladium-mediated interconversion of the intermediate enolates prior to alkylation (Scheme 1B).^7h^

In contrast to enolates, the asymmetric allylic alkylation of α-anions of sulfones is more challenging.^8^ Tunge and co-workers developed an enantiospecific, stereoretentive decarboxylative allylic alkylation of linear sulfones **7** to **8** (Scheme 1C).^8b^ Their study revealed that allylic alkylation occurred faster than racemization of the α-sulfonyl anion, retaining the stereochemical information in the process. The enantioselective allylic alkylation of *racemic* α-sulfonyl nucleophiles remains elusive.^9^

To enable an enantioconvergent alkylation of racemic sulfones, we incorporated a carbonyl group in thietane 1,1-dioxide **9** as a means of simultaneously stabilizing the α-sulfonyl anion and ensuring complete stereoablation via planar enolate **10** (Scheme 1D). However, as decarboxylation would likely lead to a mixture of *E*/*Z* enolates **10**, a palladium-mediated interconversion of the enolates would be required in order to obtain **11** with high ee. Herein we report the first palladium-catalyzed asymmetric allylic alkylation of thietane 1,1-dioxides to generate α-sulfonyl stereogenic tetrasubstituted carbon centers in **11** from racemic starting materials without the need for geometrically pure, preformed enol carbonate precursors. We illustrate the utility of these products in the synthesis of the novel, enantioenriched thietane 1,1-dioxide containing spirocycle **12**.

Our studies began with a three-step synthesis of precursors **16** and **17** (Scheme 2). The oxidation of commercially available thietane (**13**) to thietane 1,1-dioxide (**14**) and allyl ester installation gave **15**, which was derivatized in a divergent manner to substrates **16** and **17**, bearing ketone and ester substituents, respectively.

**Scheme 2:**
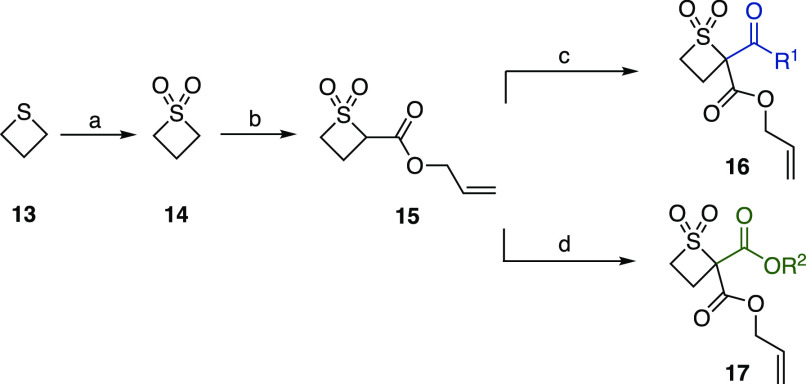
Three-Step Synthesis of Ketone and Ester Precursors Reagents and conditions: (a) KMnO_4_ (2 equiv), CH_2_Cl_2_/H_2_O, rt, 94%; (b) LiHMDS (2.1 equiv), allyl chloroformate, THF, – 78 °C, 68%; (c) NaHMDS (1.1 equiv), R^1^COCl, THF, 0 °C; (d) KHMDS (1.1 equiv), R^2^OCOCl, THF, 0 °C. Abbreviations: THF, tetrahydrofuran; HMDS, bis(trimethylsilyl)amide.

The development of the Pd-DAAA process was undertaken using ketone substrate **16a** (Table 1; see the Supporting Information for the full optimization study). Initial experiments to identify a suitable ligand for the enantioselective conversion of **16a** to **18a** revealed that the use of PHOX **L1**, as well as DACH phenyl and naphthyl Trost ligands **L2** and **L3**, resulted in poor ee in 1,4-dioxane as the solvent (8%, 34% and 24% ee, respectively, entries 1–3). In contrast, (*S*,*S*)-ANDEN Trost ligand **L4** gave a high ee of 83% of **18a** (entry 4), despite the acyclic nature of the enolate intermediate. It was found that a reaction of lower concentration (0.04 M, entry 4) yielded **18a** with higher ee in comparison to those with greater concentrations (0.1 and 0.2 M, entries 5 and 6, respectively). Reactions in acetonitrile and toluene were poorly selective (entries 7 and 8). 1,4-Dioxane was also superior to other ethereal solvents (entries 9 and 10), presumably due to its ability to better stabilize caged ion pairs.^10^ Despite obtaining an excellent yield (98%) of **18a** in CH_2_Cl_2_ (entry 11), the enantioselectivity was also lower in comparison to 1,4-dioxane. Attempts to lower the temperature to 0 °C using a 3/1 1,4-dioxane/CH_2_Cl_2_ mixture resulted in no improvement in ee (entry 12), with the reaction in 1,4-dioxane at room temperature being optimal (entry 4).

**Table 1:**
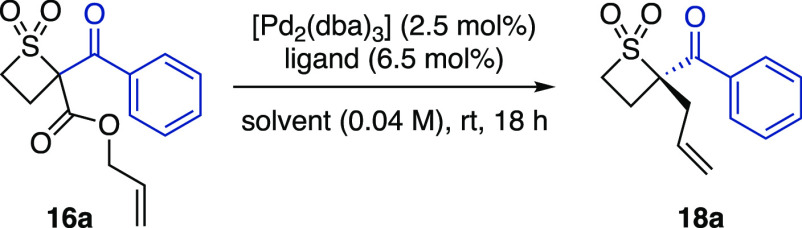
Reaction Optimization Studies^a^

entry	ligand	solvent	yield (%)^b^	ee (%)^c^
1	**L1**	1,4-dioxane	38	8
2	**L2**	1,4-dioxane	80	34
3	**L3**	1,4-dioxane	80	24
4	**L4**	1,4-dioxane	78	83
5	**L4**	1,4-dioxane (0.1 M)	84	79
6	**L4**	1,4-dioxane (0.2 M)	80	75
7	**L4**	MeCN	71	1
8	**L4**	PhMe	78	22
9	**L4**	Et_2_O	71	38
10	**L4**	THF	70	41
11	**L4**	CH_2_Cl_2_	98	65
12	**L4**	1,4-dioxane/CH_2_Cl_2_ (3/1)^d^	82	81

a Conditions: **16a** (0.17 mmol), [Pd_2_(dba)_3_] (2.5 mol %), (**L1**–**4**) (6.5 mol %). Abbreviation: dba, dibenzylideneacetone.

b Isolated yield.

c Enantiomeric excess determined by chiral HPLC;

d Performed at 0 °C.
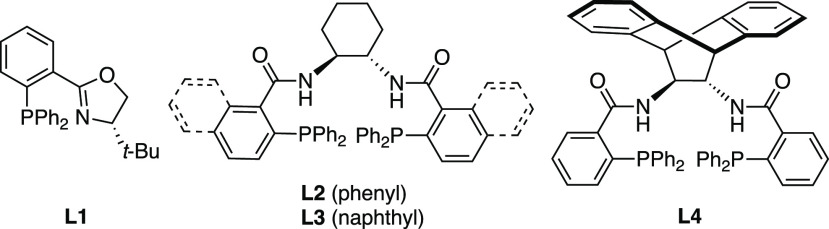

With the optimal conditions identified, the substrate scope was investigated by subjecting racemic precursors **16** and **17** to the catalytic reaction (Scheme 3). We discovered that electronics had little effect on the enantioselectivity of the reaction, with all substituted phenyl aromatic ketones giving ee values of products **18a**–**f** of 83–86%. In spite of the increased size of the *o*-toluoyl ketone substituent in **18b**, the yield and ee remained high. *p*-Fluoro- and bromo-substituted phenyl substituents were tolerated, with no oxidative addition occurring at the C–Br bond. A slightly lower ee of 79% was obtained for methyl ester containing product **18g**. Aromatic heterocycles, such as furyl- and pyridyl-containing **18h**,**i**, were obtained with 81% and 72% ee, respectively. Substitution on the allyl group at the internal or terminal position (**18j**–**m**) necessitated a higher catalyst loading due to the lower reactivity of these substrates, and the ee also dropped significantly to 52–55%. Prenylated substrate **18n** did not undergo the reaction, presumably due to large steric hindrance at the allyl functionality for the initial oxidative addition step. Alkyl-substituted ketones were isolated with consistently high ee values of >90% in the case of large substituents, such as adamantyl (**18o**), *tert*-butyl (**18p**), cyclohexyl (**18q**), and isopropyl (**18r**). However, as the steric bulk of the substituent decreased, the enantioselectivity of the reaction fell: the ee values of **18s**,**t** were 81% and 69%, respectively, whereas product **18u** bearing a methyl ketone substituent was isolated with a much lower 39% ee. Using the same reaction conditions, ester-substituted products were all obtained with high enantioselectivity, including phenyl aromatic esters **19a**–**c**, *tert*-butyl ester **19d**, and methyl ester **19e**. X-ray crystal structures of **19a**,**b** confirmed the absolute stereochemical configuration of the major enantiomer of product using (*S*,*S*)-ANDEN phenyl Trost ligand **L4**.^11^ Substrates with an amide as the stabilizing group did not undergo decarboxylation under the optimized conditions.

**Scheme 3:**
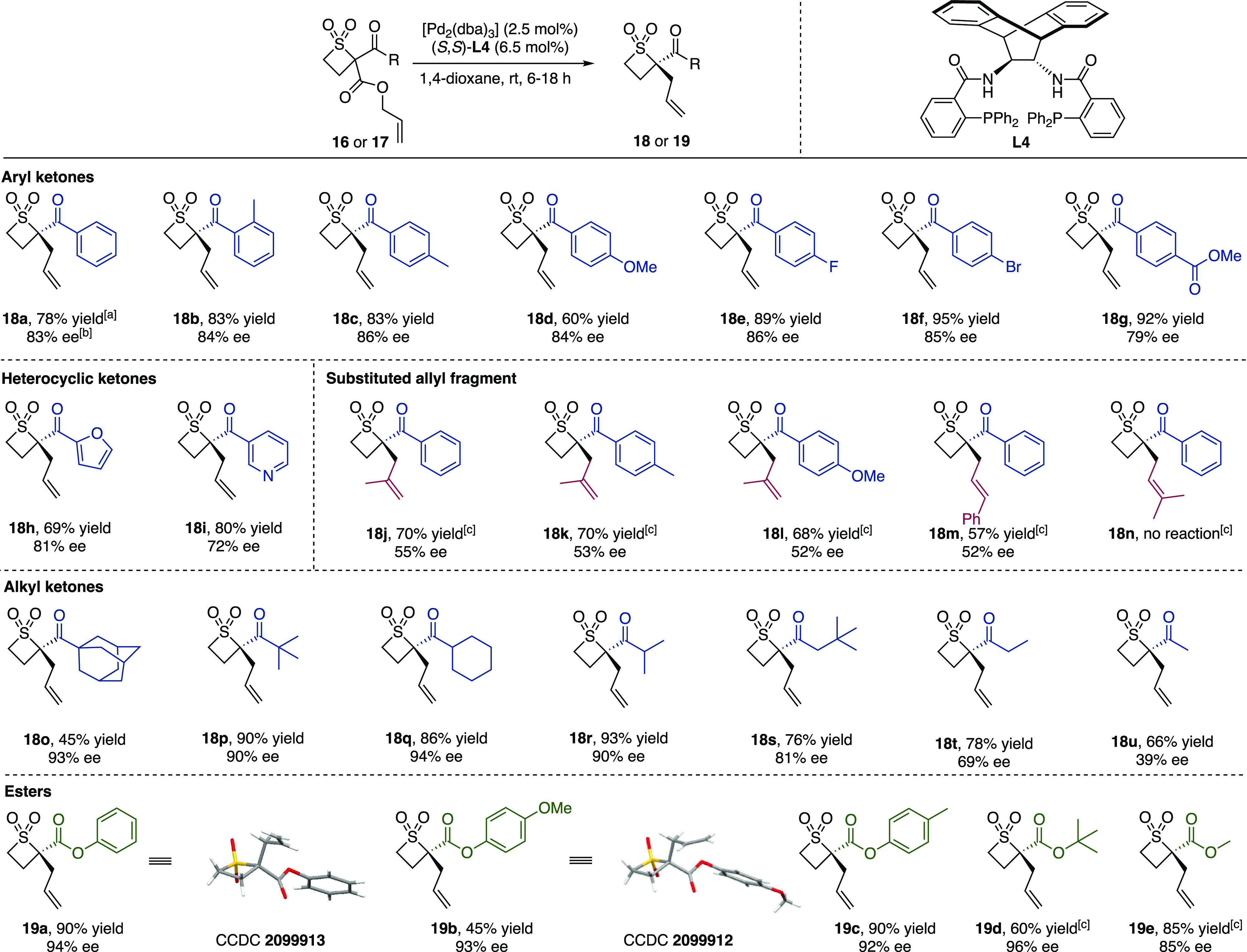
Pd-DAAA of Thietane 1,1-Dioxides Isolated yields are given. Enantiomeric excess determined by chiral HPLC. Conditions: [Pd_2_(dba)_3_] (5 mol %), (*S*,*S*)-**L4** (13 mol %).

Given that the alkene geometry of acyclic enolates can affect the stereochemical outcome of the allylic alkylation reaction,^7d,7h−7j^ we sought to explore the importance of the enolate geometry on both the sense and magnitude of enantioinduction. In this context, the *E*- and *Z*-enol carbonates **20** were prepared and reacted under the standard conditions (Scheme 4). Both isomers of **20** afforded (*S*)-**18p** as the major enantiomer in 76% ee from (*E*)-**20** and 88% ee from (*Z*)-**20** with (*S*,*S*)-**L4** as the chiral ligand. In the case of (*E*)-**20**, the level of enantioselectivity was slightly lower than that when allyl ester **16p** was used as the substrate (90% ee of **18p**). We therefore postulate that a palladium-mediated interconversion of *E*- and *Z*-enolates occurs and that alkylation of the *Z*-enolate results in the formation of **18p**. (For a rationale of the origins of stereocontrol using the Trost “wall-and-flap” model, see the Supporting Information.)

**Scheme 4:**
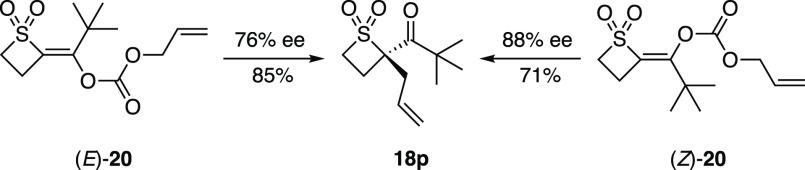
Effect of Enolate Geometry Conditions: [Pd_2_(dba)_3_] (2.5 mol %), (*S*,*S*)-**L4** (6.5 mol %), 1,4-dioxane (0.04 M), rt, 18 h.

To gain further insight into the mechanism of this reaction, an enolate crossover experiment of **16a** and deuterium-labeled [D]-**16c** was performed (Scheme 5A). The isolated product mixture comprised all four crossover compounds **18a**, [D]-**18a**, **18c**, and [D]-**18c**, as confirmed by high-resolution mass spectrometry. The complete scrambling of enolates suggests that the ion pairs generated in this reaction undergo fast ion exchange.^7d^ To test whether an enolate as part of an ion pair is a long-lived intermediate in the reaction, a water additive was used (Scheme 5B). We expected water to quench a free enolate to at least some extent if such a species was present in the reaction and significantly affect the yield, and potentially ee, of product **18a**. However, neither the yield nor the enantioselectivity of the reaction was affected even when up to 20 equiv of water was added (see the Supporting Information for further details), indicating that a free enolate is an unlikely species in the reaction.

**Scheme 5:**
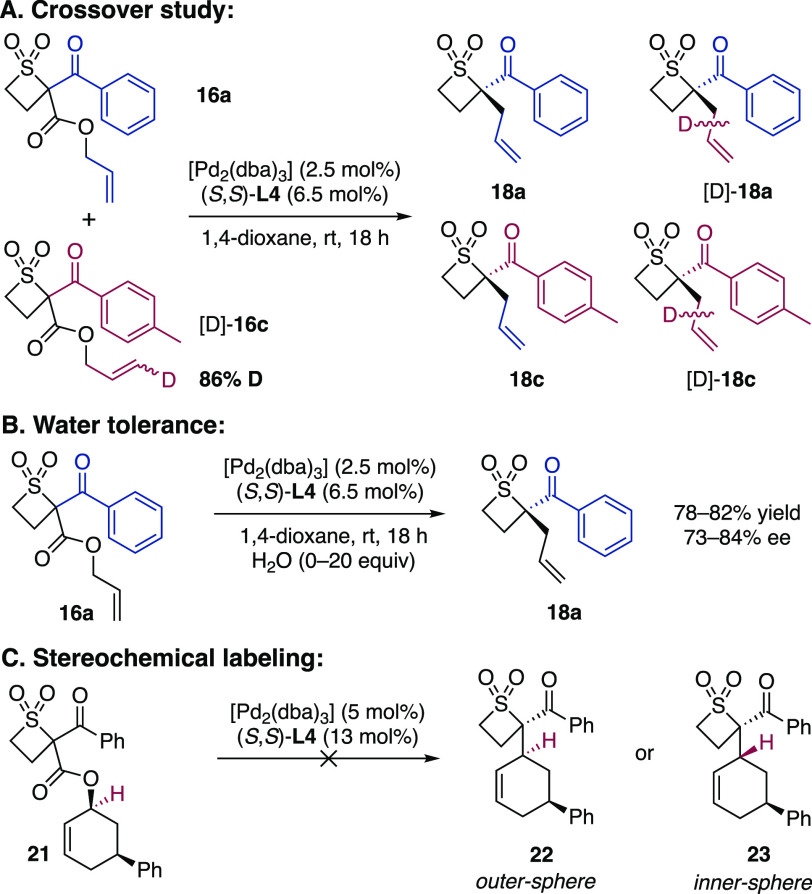
Mechanistic Study

Finally, to elucidate whether an inner- or outer-sphere enolate alkylation operates, substituted allylic electrophile *cis*-**21** was prepared (Scheme 5C). In the case of an outer-sphere alkylation, net retention of the allylic center would be expected in **22**. Alternatively, the inner-sphere mechanism would result in net stereochemical inversion in **23**. Unfortunately, **21** failed to undergo the desired alkylation due to the sterically encumbered nature of the allylic electrophile (see the Supporting Information for further details).

Using this information, a catalytic cycle for the Pd-DAAA reaction of thietane 1,1-dioxide is proposed (Scheme 6). Following ionization of **16**, palladium-carboxylate ion pair **24** is formed. We believe it is at this stage that ion crossover occurs. Given that the reaction is unaffected by water, it is likely that a free enolate is not formed due to the slow decarboxylation of **24**.^12^ This decarboxylation step is assisted by palladium (**25**),^13^ which facilitates the requisite *E*/*Z* enolate equilibrium between **26a** and **26b** via a carbon-bound palladium enolate to afford enantioenriched **18** and release the palladium(0) catalyst.

**Scheme 6:**
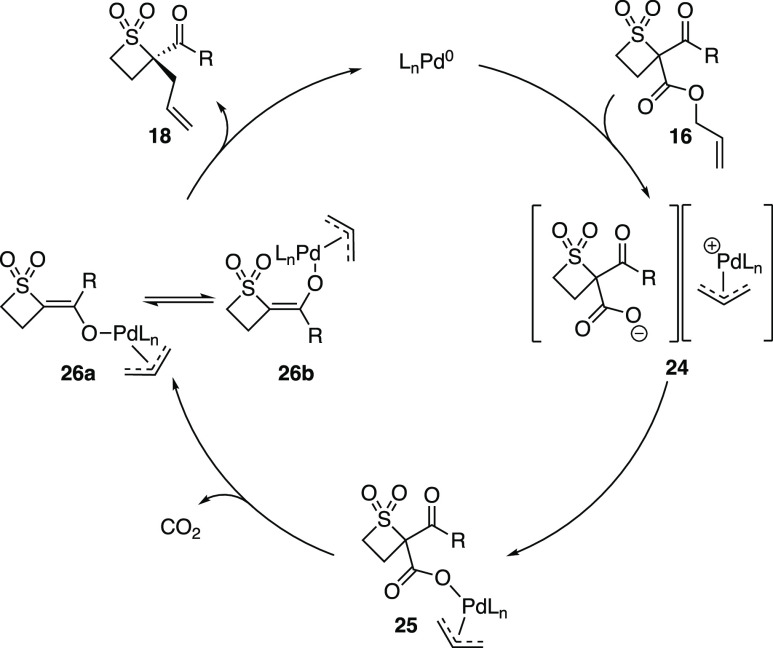
Reaction Mechanism

The enantioenriched tetrasubstituted thietane 1,1-dioxides obtained by the Pd-DAAA methodology are excellent building blocks for further derivatization into novel spirocycles (Scheme 7). In this context, the key allylic alkylation process of **17d** was scaled up, furnishing **19d** in 86% yield and 96% ee on a 5 g scale. The alkene in **19d** was subjected to a hydroboration, oxidation, and reductive amination sequence, and removal of the *tert*-butyl group afforded amino acid **27**. Subsequent lactamization, amide reduction, and deprotection furnished spirocycle **12**. Finally, to exemplify the utility of spirocycle **12** in the generation of compound libraries, the amine in **12** successfully underwent reductive amination, amide bond formation, and Buchwald–Hartwig processes, efficiently furnishing products **28a**–**c**.

**Scheme 7:**
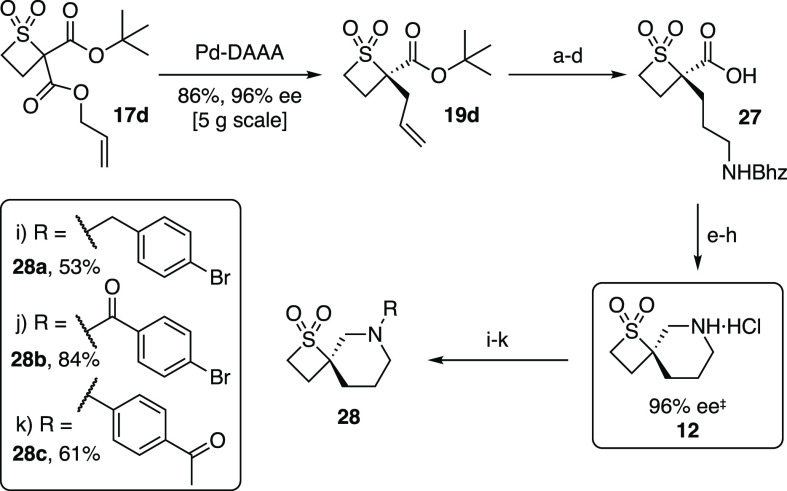
Synthesis and Functionalization of Spirocycle **12** Conditions: (a) 9-BBN, NaOH, H_2_O_2_, THF, 62%; (b) Dess–Martin periodinane, CH_2_Cl_2_, 78%; (c) NH_2_Bhz, AcOH, NaBH(OAc)_3_, DCE, 57%; (d) TFA, CH_2_Cl_2_, quantitative; (e) EDCI·HCl, pyridine, DMAP, CH_2_Cl_2_, 89%; (f) BH_3_·THF, THF, 96%; (g) Pd(OH)_2_/C, H_2_, EtOH, TFA, 81%; (h) 4 N HCl, 1,4-dioxane, 95%. Functionalizations: (i) **12** (0.15 mmol), 4-bromobenzylaldehyde, AcOH, NaBH(OAc)_3_, DCE, 53%; (j) **12** (0.07 mmol), 4-bromobenzoyl chloride, Et_3_N, CH_2_Cl_2_, 84%; (k) **12** (0.15 mmol), 4-bromoacetophenone, Pd(OAc)_2_, *rac*-BINAP, Cs_2_CO_3_, toluene, 61%. Abbreviations: 9-BBN, 9-borabicyclo[3.3.1]nonane; Bhz, benzhydryl; DCE, 1,2-dichloroethane; TFA, trifluoroacetic acid; EDCI, *N*-(3-(dimethylamino)propyl)-*N′*-ethylcarbodiimide; DMAP, 4-(dimethylamino)pyridine; BINAP, 2,2′-bis(diphenylphosphino)-1,1′-binaphthalene. ^‡^For the purposes of ee determination by chiral HPLC analysis, **12** was first Boc-protected (see the Supporting Information, **28d**).

In conclusion, we have developed the first enantioconvergent palladium-catalyzed DAAA reaction of thietane 1,1-dioxides, in which a carbonyl substituent enables the key stereoablation required to produce enantioenriched alkylated products from racemic precursors. In spite of the likely formation of both *E-* and *Z-*enolates in the reaction, a palladium-mediated enolate interconversion is thought to occur, resulting in high levels of enantioselectivity. The synthetic utility of these products has been demonstrated by their expedient derivatization into a novel, enantioenriched thietane 1,1-dioxide containing spirocycle as a high-value sp^3^-rich building block for use in medicinal chemistry applications.

## Supporting Information Available

Details on experimental procedures, characterization data and NMR spectra for novel compounds, and data for the determination of enantiomeric excess (PDF)

## Accession Codes

CCDC 2099912–2099913 contain the supplementary crystallographic data for this paper. These data can be obtained free of charge via www.ccdc.cam.ac.uk/data_request/cif, or by emailing data_request@ccdc.cam.ac.uk, or by contacting The Cambridge Crystallographic Data Centre, 12 Union Road, Cambridge CB2 1EZ, UK; fax: +44 1223 336033.
