# Supplementary material for: Palladium-Catalyzed Decarboxylative Asymmetric Allylic Alkylation of Thietane 1,1-Dioxides
Source: Org Lett. 2021 Dec 16;24(1):400–5. doi: 10.1021/acs.orglett.1c04075 (PMC8762707; doi:10.1021/acs.orglett.1c04075)
Supplement: Supplementary file 1 — ol1c04075_si_001.pdf [file ol1c04075_si_001.pdf]

Supporting Information

Palladium-Catalyzed Decarboxylative Asymmetric Allylic Alkylation of Thietane 1,1-Dioxides

Gillian Laidlaw and Vilius Franckevičius*

Lancaster University, Department of Chemistry, Bailrigg, Lancaster, LA1 4YB, UK

*e-mail: v.franckevicius@lancaster.ac.uk

Contents

1. General Experimental Section S-2

2. Experimental Procedures and Characterisation Data S-4

    2.1 Synthesis of Allyl Ester Starting Material **15** S-4

    2.2 Synthesis of Substituted Allyl Ester Starting Material **31** S-5

    2.3 Synthesis of β-keto Ester Precursors S-9

    2.4 Palladium-Catalysed Decarboxylative Asymmetric Allylic Alkylation S-31

    2.5 Synthesis of Spirocycle **12** S-54

    2.6 Functionalisations of Spirocycle **12** S-61

3. Mechanistic Study S-64

    3.1 Synthesis of *E*- and *Z*- Enol Carbonates **20** S-64

    3.2 Crossover Study S-70

    3.3 Water Tolerance Study S-78

    3.4 Stereochemical Labelling S-79

4. Rationale for Origins of Stereocontrol S-83

5. X-ray Crystallography Data S-85

6. ^{1}H and ^{13}C NMR Spectra S-89

7. HPLC Data S-171

8. References S-198

1. General Experimental Section

1.1. Solvents, Reagents and Starting Materials

Oven-dried glassware was used for all reactions under an argon atmosphere. Dry solvents were obtained from commercial sources or obtained from an Innovative Technologies PureSolv solvent drying system. All reagents and solvents were used as supplied. All reactions conducted above room temperature were heated using a heating block on a stirrer hotplate. Petrol refers to the fraction of petroleum that boils between 40 ºC and 60 ºC. Aqueous solutions were saturated unless otherwise stated. Removal of solvents under vacuum refers to the use of a rotary evaporator at 40 °C, with further drying on a high vacuum line. VWR Chemicals silica gel (40–63 µm particle size) was used for flash column chromatography. Thin layer chromatography (TLC) was carried out using Merck KgaA silica gel 60 F254 aluminium-backed plates. Ultraviolet irradiation (254 nm) and staining with potassium permanganate solution or acidic ammonium molybdate as appropriate were used to visualise TLC plates. X-ray crystal structures **19a** and **19b** were obtained by vapor diffusion using hexane/EtOAc.

1.2. Instrumentation

^{1}H NMR spectra were obtained using either a Bruker AVANCE III 400 spectrometer or a Bruker FOURIER 300 spectrometer. ^{13}C NMR spectra were recorded on the same spectrometers at 100 MHz or 75 MHz, respectively. For ^{1}H NMR spectra recorded in $CDCl_3$, the residual protic solvent $CHCl_3$ (δ_H = 7.26 ppm) was used as the internal reference. For ^{13}C NMR spectra, the central resonance of $CDCl_3$ (δ_C = 77.2 ppm) was used as the internal reference. For ^{1}H NMR spectra recorded in DMSO-d_6, the residual protic solvent (δ_H = 2.50 ppm) was used as the internal reference. For ^{13}C NMR spectra, the central resonance of DMSO-d_6 (δ_C = 39.5 ppm) was used as the internal reference. For ^{1}H NMR spectra recorded in benzene-d_6, the residual protic solvent (δ_H = 7.16 ppm) was used as the internal reference. For ^{13}C NMR spectra, the central resonance of benzene-d_6 (δ_C = 128.1 ppm) was used as the internal reference. NMR data are reported as follows: chemical shift, δ_H (in parts per million, ppm), (number of protons, multiplicity, coupling constant, *J* in Hertz). Couplings are expressed as one, or a combination of: s, singlet; br s, broad singlet; d, doublet; t, triplet; q, quartet; quint, quintet; sextet; septet and m, multiplet. When coincidental couplings constants were observed

in the NMR spectra, the apparent multiplicity of the proton resonance in these cases was reported.

High resolution mass spectra (HRMS) were recorded using a Shimadzu LCMS-IT-TOF instrument using ESI or APCI conditions. Infra-red spectra were recorded on an Agilent Technologies Cary 630 FTIR spectrometer. Melting points were measured on a Sanyo Gallenkamp capillary melting point apparatus. Enantiomeric excesses were determined by chiral HPLC on a Shimadzu NEXERA X2 UHPLC instrument equipped with a UV detector, using either a CHIRALCEL OD-H or CHIRALPAK AD-H column. Optical rotations were measured in using an AA-65 Automatic Polarimeter. Where X-ray crystal structures were obtained, single crystals were selected and mounted, on a Mitegen loop using Paratone-N oil, on a SuperNova, Dual, Cu at zero, AtlasS2 diffractometer. The crystals were kept at 100 K during data collection, and data reduction was performed using CrysAlisPro1.171.38.44a (Rigaku Oxford Diffraction, 2015).

2. Experimental Procedures and Characterisation Data

2.1 Synthesis of Allyl Ester Starting Material 15

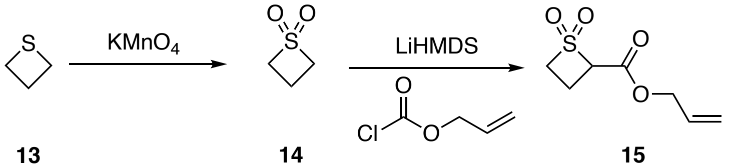

Trimethylene sulfide **13** was purchased from Sigma Aldrich and used without further purification.

Thietane 1,1-dioxide (14)

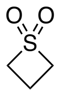

14

Thietane **13** (6.12 mL, 85 mmol) and potassium permanganate (26.9 g, 170 mmol) were added to a 1:1 mixture of CH_2Cl_2:H_2O (600 mL). The reaction mixture was stirred vigorously at room temperature overnight. The mixture was filtered under reduced pressure and the aqueous layer was extracted with CH_2Cl_2 (200 mL). The organic phase was separated, and the aqueous phase was extracted further with CH_2Cl_2 (3 x 100 mL). The combined organic phase was washed with aq. $Na_2S_2O_3$ (10%, 100 mL), dried ($MgSO_4$) and concentrated under reduced pressure to afford thietane 1,1-dioxide **14** (6.99 g, 78%) as a colourless solid.

^{1}H NMR: (400 MHz, $CDCl_3$) δ 4.16 – 4.12 (4H, m), 2.21 – 2.12 (2H, m).

^{13}C NMR: (100 MHz, $CDCl_3$) δ 65.7, 6.0.

IR: ν_{max} (neat/cm^{-1}): 2968, 1302, 1127.

All characterisation data are consistent with those reported in the literature.[1]

Allyl 1,1-dioxothietane-2-carboxylate (15)

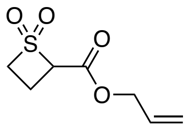

15

A solution of thietane 1,1-dioxide **14** (1.50 g, 14.1 mmol) in THF (15 mL) was added to a solution of LiHMDS (1 M in THF, 29.6 mL, 29.6 mmol) in THF (60 mL) at –78 °C. The reaction mixture was stirred at –78 ºC for 1 h. Allyl chloroformate (1.65 mL, 15.5 mmol) was added dropwise and the mixture was stirred at –78 ºC for 2 h. The reaction was quenched at –78 °C with aq. HCl (1 N, 10 mL), allowed to warm to room temperature and diluted with water (10 mL). The mixture was extracted with EtOAc (3 x 100 mL), washed with brine (30 mL), dried ($MgSO_4$) and concentrated under reduced pressure. Purification by flash column chromatography [SiO_2 deactivated with 2% NEt_3; 12:1 hexane:EtOAc] afforded allylic ester **15** (1.81 g, 68%) as a colourless solid. R_f = 0.22 [3:1 petrol:EtOAc]. **mp**: 40 – 42 ºC.

^{1}H NMR: (400 MHz, $CDCl_3$) δ 5.98 – 5.87 (1H, m), 5.39 (1H, dq, *J* = 17.2, 1.4 Hz), 5.29 (1H, dq, *J* = 10.4, 1.2 Hz), 5.11 – 5.06 (1H, m), 4.76 – 4.71 (2H, m), 4.25 – 4.14 (2H, m), 2.65 – 2.53 (1H, m), 2.37 – 2.26 (1H, m).

^{13}C NMR: (100 MHz, $CDCl_3$) δ 164.0, 131.1, 119.6, 79.8, 67.3, 65.8, 9.7.

HRMS: (ESI-TOF) m/z: $[M–H]^-$ calcd for $C_7H_9O_2S$ 189.0227; found 189.0232.

IR: ν_{max} (neat/cm^{-1}): 3035, 2959, 1742, 1312, 1125.

2.2 Synthesis of Substituted Allyl Ester Starting Material 31

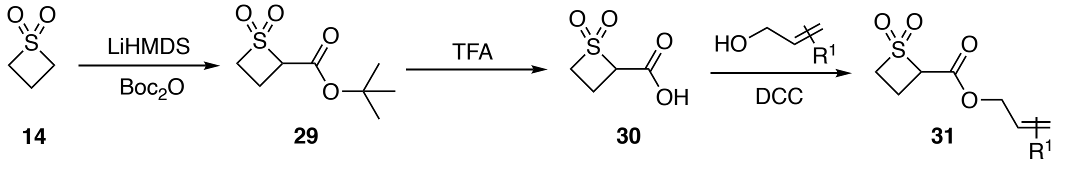

tert-Butyl 1,1-dioxothietane-2-carboxylate (29)

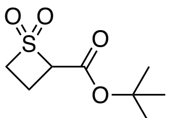

29

A solution of thietane 1,1-dioxide **14** (5.30 g, 50 mmol) in THF (100 mL) was added dropwise to a solution of LiHMDS (1 M in THF, 105 mL, 105 mmol) at –78 ºC, and the reaction was stirred at –78 ºC for 1 h. Di-*tert*-butyl dicarbonate (12.6 mL, 55 mmol) was added dropwise and the mixture was stirred at –78 ºC for 3 h. The reaction was quenched at –78 ºC with aq. HCl (1 N, 50 mL) and was allowed to warm to room temperature. The mixture was diluted

with water (100 mL) and extracted with EtOAc (3 x 100 mL). The combined organic layers were washed with brine (100 mL), dried ($MgSO_4$) and concentrated under reduced pressure. Purification by flash column chromatography [SiO_2, 9:1 hexane:EtOAc] afforded **29** (8.62 g, 84%) as a colourless solid. $\boldsymbol{R}_\mathbf{f}$ = 0.41 [2:1 petrol:EtOAc]. **mp**: 56 – 57 ºC.

^{1}H NMR: (400 MHz, $CDCl_3$) δ 5.00 – 4.95 (1H, m), 4.20 – 4.11 (2H, m), 2.59 – 2.49 (1H, m), 2.29 – 2.19 (1H, m), 1.52 (9H, s).

^{13}C NMR: (100 MHz, $CDCl_3$) δ 163.2, 84.2, 80.8, 65.1, 28.1, 9.6.

HRMS: (APCI-TOF) m/z: $[M–H]^–$ calcd for $C_8H_{13}O_4S$ 205.0540; found 205.0546.

IR: ν_{max} (neat/cm^{-1}): 2987, 2942, 1725, 1313, 1129.

1,1-Dioxothietane-2-carboxylic acid (30)

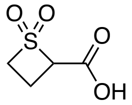

30

A solution of **29** (250 mg, 1.22 mmol) in CH_2Cl_2 (10 mL) was cooled to 0 ºC. Trifluoroacetic acid (0.94 mL, 12.2 mmol) was added dropwise and the reaction was stirred at 0 ºC for 6 h. The mixture was allowed to warm to room temperature, then concentrated under reduced pressure to afford **30** (175 mg, 98%) as a brown solid which was used in the next step without further purification. **mp**: 118 – 119 ºC.

^{1}H NMR: (400 MHz, DMSO-d_6) δ 13.5 (1H, br s), 5.40 – 5.33 (1H, m), 4.27 – 4.18 (1H, m), 4.14 – 4.06 (1H, m), 2.27 – 2.18 (2H, m).

^{13}C NMR: (100 MHz, DMSO-d_6) δ 165.2, 79.4, 64.9, 9.5.

HRMS: (ESI-TOF) m/z: $[M–H]^–$ calcd for $C_4H_5O_4S$ 148.9914; found 148.9921.

IR: ν_{max} (neat/cm^{-1}): 3239, 2991, 1304, 1172.

2-Methylallyl 1,1-dioxothietane-2-carboxylate (31a)

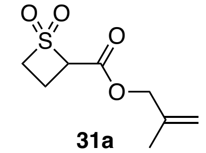

31a

To a suspension of **30** (150 mg, 1.0 mmol) in CH_2Cl_2 (2 mL) was added a solution of 2-methyl-2-propen-1-ol (162 μL, 2.0 mmol) and a few crystals of 4-dimethylaminopyridine in CH_2Cl_2 (3 mL). The mixture was cooled to 0 ºC. A solution of *N*,*N*'-dicyclohexylcarbodiimide (227 mg, 1.1 mmol) in CH_2Cl_2 (2 mL) was added. The mixture was allowed to warm to room temperature and stirred at ambient temperature for 18 h. The reaction mixture was filtered through Celite twice, then concentrated under reduced pressure. Purification using flash column chromatography [SiO_2; 7:1 hexane:EtOAc] afforded **31a** (88 mg, 43%) as a yellow oil. $\boldsymbol{R_f}$ = 0.26 [3:1 petrol:EtOAc].

^{1}H NMR: (400 MHz, $CDCl_3$) δ 5.13 – 5.07 (1H, m), 5.06 – 5.04 (1H, m), 5.00 – 4.97 (1H, m), 4.71 (1H, d, *J* = 12.7 Hz), 4.62 (1H, d, *J* = 12.8 Hz), 4.23 – 4.15 (2H, m), 2.65 – 2.55 (1H, m), 2.37 – 2.27 (1H, m), 1.79 (3H, d, *J* = 0.4 Hz).

^{13}C NMR: (100 MHz, $CDCl_3$) δ 164.0, 139.0, 114.6, 79.8, 70.1, 65.3, 19.5, 9.7.

HRMS: (APCI-TOF) m/z: $[M+H]^+$ calcd for $C_8H_{12}O_4S$ 205.0529; found 205.0521.

IR: ν_{max} (neat/cm^{-1}): 2976, 1735, 1317, 1131.

(*E*)-Cinnamyl 1,1-dioxothietane-2-carboxylate (31b)

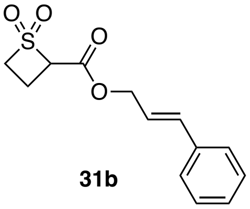

31b

To a suspension of **30** (150 mg, 1.0 mmol) in CH_2Cl_2 (2 mL) was added a solution of 3-phenyl-2-propen-1-ol (260 μL, 2.0 mmol) and a few crystals of 4-dimethylaminopyridine in CH_2Cl_2 (3 mL). The mixture was cooled to 0 ºC. A solution of *N*,*N*'-dicyclohexylcarbodiimide (227 mg, 1.1 mmol) in CH_2Cl_2 (2 mL). The mixture was allowed to warm to room temperature and stirred at ambient temperature for 18 h. The reaction mixture was filtered through Celite twice,

then concentrated under reduced pressure. Purification by flash column chromatography [SiO_2; 10:1 hexane:EtOAc] afforded **31b** (89 mg, 34%) as a colourless solid. ***R*f** = 0.29 [3:1 petrol:EtOAc]. **mp**: 128 – 130 ºC.

^{1}H NMR: (400 MHz, $CDCl_3$) δ 7.42 – 7.38 (2H, m), 7.35 – 7.31 (2H, m), 7.29 – 7.27 (1H, m), 6.72 (1H, d, *J* = 15.9 Hz), 6.29 (1H, dt, *J* = 15.9, 6.5 Hz), 5.11 (1H, ddt, *J* = 9.9, 6.8, 0.9 Hz), 4.91 (2H, dt, *J* = 6.6, 1.6 Hz), 4.26 – 4.15 (2H, m), 2.67 – 2.57 (1H, m), 2.38 – 2.29 (1H, m).

^{13}C NMR: (100 MHz, $CDCl_3$) δ 164.1, 135.9, 135.5, 128.8, 128.5, 126.9, 122.0, 79.8, 67.4, 65.3, 9.8.

HRMS: molecular ion not detected for $C_{13}H_{14}O_4S$.

IR: ν_{max} (neat/cm^{-1}): 3049, 2976, 2929, 1735, 1312, 1127.

3-Methylbut-2-enyl 1,1-dioxothietane-2-carboxylate (31c)

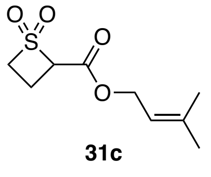

31c

To a suspension of **30** (100 mg, 0.67 mmol) in CH_2Cl_2 (2 mL) was added a solution of 3-methyl-2-buten-1-ol (271 μL, 2.67 mmol) and a few crystals of 4-dimethylaminopyridine in CH_2Cl_2 (3 mL). The mixture was cooled to 0 ºC. A solution of *N*,*N*'-dicyclohexylcarbodiimide (153 mg, 0.74 mmol) in CH_2Cl_2 (2 mL) was added. The mixture was allowed to warm to room temperature and stirred at ambient temperature for 18 h. The reaction mixture was filtered through Celite twice, then concentrated under reduced pressure. Purification by flash column chromatography [SiO_2; 9:1 hexane:EtOAc] afforded **31c** (82 mg, 56%) as a colourless solid. ***R*f** = 0.68 [3:2 petrol:EtOAc]. **mp**: 44 – 46 ºC.

^{1}H NMR: (400 MHz, $CDCl_3$) δ 5.40 – 5.34 (1H, m), 5.04 (1H, ddt, *J* = 9.8, 6.8, 1.1 Hz), 4.77 (1H, dd, *J* = 12.2, 7.4 Hz), 4.71 (1H, dd, *J* = 12.1, 7.4 Hz), 4.22 – 4.13 (2H, m), 2.64 – 2.54 (1H, m), 2.29 (1H, ddd, *J* = 12.4, 9.8, 6.7 Hz), 1.77 (3H, s), 1.73 (3H, s).

^{13}C NMR: (100 MHz, $CDCl_3$) δ 164.3, 140.8, 117.7, 79.9, 65.3, 63.7, 25.9, 18.2, 9.8.

HRMS: molecular ion not detected for $C_9H_{14}O_4S$.

IR: ν_{max} (neat/cm^{-1}): 3043, 2980, 2929, 1729, 1315, 1127.

2.3 Synthesis of β-keto Ester Precursors

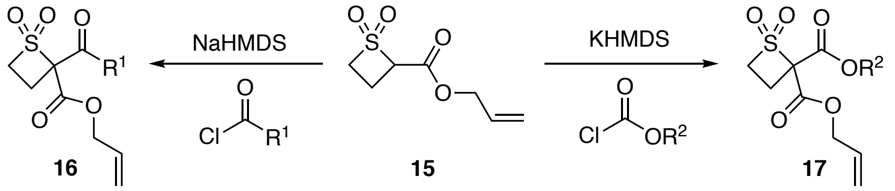

Acid chlorides and chloroformates were purchased from Sigma Aldrich and used without further purification.

Allyl 2-benzoyl-1,1-dioxo-thietane-2-carboxylate (16a)

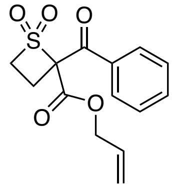

16a

A solution of NaHMDS (1 M in THF, 1.2 mL, 1.2 mmol) in THF (10 mL) was cooled to 0 ºC. A solution of allyl ester **15** (200 mg, 1.05 mmol) in THF (5 mL) was added dropwise and the reaction mixture was stirred at 0 ºC for 30 minutes. Benzoyl chloride (160 μL, 1.15 mmol) was added dropwise and the mixture stirred at 0 ºC for 4 h. The reaction was quenched with aq. HCl (1 N, 1 mL), allowed to warm to room temperature and diluted with water (10 mL). The mixture was extracted with EtOAc (3 x 20 mL), washed with brine (10 mL), dried ($MgSO_4$) and concentrated under reduced pressure. Purification by flash column chromatography [SiO_2; 10:1 hexane:EtOAc] afforded **16a** (220 mg, 70%) as a colourless oil. $\boldsymbol{R}_f$ = 0.40 [3:1 petrol:EtOAc].

^{1}H NMR: (400 MHz, $CDCl_3$) δ 8.01 – 7.96 (2H, m), 7.62 (1H, tt, J = 7.4, 1.2 Hz), 7.53 – 7.47 (2H, m), 5.77 (1H, ddt, J = 17.4, 10.2, 5.8 Hz), 5.22 (1H, dq, J = 5.8, 1.2 Hz), 5.20 – 5.18 (1H, m), 4.73 (1H, ddt, J = 13.1, 5.8, 1.4 Hz), 4.67 (1H, ddt, J = 13.1, 5.8, 1.4 Hz), 4.47 (1H, dt, J = 12.4, 10.2 Hz), 4.08 (1H, ddd, J = 12.5, 10.2, 3.4 Hz), 2.95 (1H, dt, J = 12.4, 10.1 Hz), 2.77 (1H, ddd, J = 12.5, 10.4, 3.4 Hz).

^{13}C NMR: (100 MHz, $CDCl_3$) δ 187.6, 164.5, 134.8, 134.5, 130.3, 129.7, 128.8, 120.0, 95.7, 67.9, 63.6, 17.5.

HRMS: (APCI-TOF) m/z: $[M+H]^+$ calcd for $C_{14}H_{15}O_5S$ 295.0635; found 295.0621.

IR: ν_{max} (neat/cm^{-1}): 3032, 2970, 1733, 1682, 1332, 1138.

Allyl 2-(2-methylbenzoyl)-1,1-dioxo-thietane-2-carboxylate (16b)

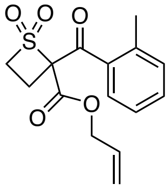

16b

A solution of NaHMDS (1 M in THF, 1.2 mL, 1.2 mmol) in THF (10 mL) was cooled to 0 ºC. A solution of allyl ester **15** (200 mg, 1.05 mmol) in THF (5 mL) was added dropwise and the reaction mixture was stirred at 0 ºC for 30 minutes. *o*-Tolyl chloride (151 μL, 1.15 mmol) was added dropwise and the mixture stirred at 0 ºC for 4 h. The reaction was quenched with aq. HCl (1 N, 1 mL), allowed to warm to room temperature and diluted with water (10 mL). The mixture was extracted with EtOAc (3 x 20 mL), washed with brine (10 mL), dried ($MgSO_4$) and concentrated under reduced pressure. Purification by flash column chromatography [SiO_2; 12:1 hexane:EtOAc] afforded **16b** (201 mg, 62%) as a colourless solid. $\boldsymbol{R}_f$ = 0.70 [3:2 petrol:EtOAc]. **mp**: 46 – 48 ºC.

^{1}H NMR: (400 MHz, $CDCl_3$) δ 7.59 (1H, dd, *J* = 8.4, 1.4 Hz), 7.43 (1H, td, *J* = 7.5, 1.2 Hz), 7.32 – 7.27 (2H, m), 5.72 (1H, ddt, *J* = 17.2, 10.4, 5.8 Hz), 5.27 – 5.18 (2H, m), 4.70 (1H, ddt, *J* = 13.0, 5.8, 1.3 Hz), 4.62 (1H, ddt, *J* = 13.0, 5.8, 1.3 Hz), 4.36 (1H, ddd, *J* = 12.5, 10.2, 8.7 Hz), 4.12 (1H, ddd, *J* = 12.5, 9.9, 4.9 Hz), 2.87 – 2.72 (2H, m), 2.51 (3H, s).

^{13}C NMR: (100 MHz, $CDCl_3$) δ 189.9, 164.6, 140.2, 134.5, 132.7, 132.4, 130.4, 129.0, 125.9, 120.0, 97.5, 67.9, 63.6, 21.5, 18.1.

HRMS: (APCI-TOF) m/z: $[M+H]^+$ calcd for $C_{15}H_{17}O_5S$ 309.0791; found 309.0779.

IR: ν_{max} (neat/cm^{-1}): 3039, 2970, 1727, 1679, 1332, 1136.

Allyl 2-(4-methylbenzoyl)-1,1-dioxo-thietane-2-carboxylate (16c)

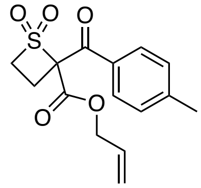

16c

A solution of NaHMDS (1 M in THF, 1.2 mL, 1.2 mmol) in THF (10 mL) was cooled to 0 ºC. A solution of allyl ester **15** (200 mg, 1.05 mmol) in THF (5 mL) was added dropwise and the reaction mixture was stirred at 0 ºC for 30 minutes. *p*-Tolyl chloride (151 μL, 1.15 mmol) was added dropwise and the mixture stirred at 0 ºC for 4 h. The reaction was quenched with aq. HCl (1 N, 1 mL), allowed to warm to room temperature and diluted with water (10 mL). The mixture was extracted with EtOAc (3 x 20 mL), washed with brine (10 mL), dried ($MgSO_4$) and concentrated under reduced pressure. Purification by flash column chromatography [SiO_2; 12:1 hexane:EtOAc] afforded **16c** (214 mg, 66%) as a colourless solid. $\boldsymbol{R}_{\mathbf{f}}$ = 0.70 [3:2 petrol:EtOAc]. **mp**: 59 – 61 ºC.

^{1}H NMR: (400 MHz, $CDCl_3$) δ 7.89 (2H, d, *J* = 8.3 Hz), 7.30 (2H, d, *J* = 8.6 Hz), 5.79 (1H, ddt, *J* = 17.1, 10.4, 5.8 Hz), 5.23 (1H, dq, *J* = 8.8, 1.3 Hz), 5.21 – 5.19 (1H, m), 4.74 (1H, ddt, *J* = 13.0, 5.7, 1.4 Hz), 4.68 (1H, ddt, *J* = 13.0, 5.7, 1.4 Hz), 4.45 (1H, dt, *J* = 12.4, 10.2 Hz), 4.07 (1H, ddd, *J* = 13.6, 10.2, 3.4 Hz), 2.95 (1H, dt, *J* = 12.5, 10.1 Hz), 2.77 (1H, ddd, *J* = 13.8, 10.4, 3.4 Hz), 2.42 (3H, s).

^{13}C NMR: (100 MHz, $CDCl_3$) δ 187.1, 164.6, 145.8, 132.3, 130.4, 129.9, 129.6, 120.0, 95.8, 67.9, 63.5, 22.0, 17.6.

HRMS: (APCI-TOF) m/z: $[M+H]^+$ calcd for $C_{15}H_{17}O_5S$ 309.0791; found 309.0780.

IR: ν_{max} (neat/cm^{-1}): 3035, 2980, 1735, 1685, 1336, 1138.

Allyl 2-(4-methoxybenzoyl)-1,1-dioxo-thietane-2-carboxylate (16d)

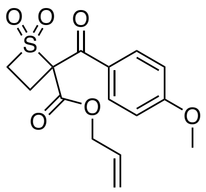

16d

A solution of NaHMDS (1 M in THF, 1.2 mL, 1.2 mmol) in THF (10 mL) was cooled to 0 ºC. A solution of allyl ester **15** (200 mg, 1.05 mmol) in THF (5 mL) was added dropwise and the reaction mixture was stirred at 0 ºC for 30 minutes. *p*-Anisoyl chloride (156 μL, 1.15 mmol) was added dropwise and the mixture stirred at 0 ºC for 4 h. The reaction was quenched with aq. HCl (1 N, 1 mL), allowed to warm to room temperature and diluted with water (10 mL). The mixture was extracted with EtOAc (3 x 20 mL), washed with brine (10 mL), dried ($MgSO_4$) and concentrated under reduced pressure. Purification by flash column chromatography [SiO_2; 16:1 hexane:EtOAc] afforded **16d** (140 mg, 41%) as a colourless oil. $\boldsymbol{R}_f$ = 0.18 [3:1 petrol:EtOAc].

^{1}H NMR: (400 MHz, $CDCl_3$) δ 8.02 (2H, d, *J* = 9.0 Hz), 6.99 (2H, d, *J* = 9.1 Hz), 5.82 (1H, ddt, *J* = 17.2, 10.5, 5.8 Hz), 5.27 (1H, dq, *J* = 9.4, 1.2 Hz), 5.24 – 5.22 (1H, m), 4.79 – 4.67 (2H, m), 4.46 (1H, dt, *J* = 12.4, 10.1 Hz), 4.08 (1H, ddd, *J* = 12.5, 10.2, 3.4 Hz), 3.90 (3H, s), 2.98 (1H, dt, *J* = 12.4, 10.1 Hz), 2.79 (1H, ddd, *J* = 12.5, 10.4, 3.4 Hz).

^{13}C NMR: (100 MHz, $CDCl_3$) δ 185.7, 164.6, 164.5, 132.2, 130.3, 127.7, 119.8, 114.0, 95.7, 67.8, 63.3, 55.6, 17.4.

HRMS: (APCI-TOF) m/z: $[M+H]^+$ calcd for $C_{15}H_{17}O_6S$ 325.0740; found 325.0725.

IR: ν_{max} (neat/cm^{-1}): 3035, 2950, 1736, 1662, 1319, 1138.

Allyl 2-(4-fluorobenzoyl)-1,1-dioxo-thietane-2-carboxylate (16e)

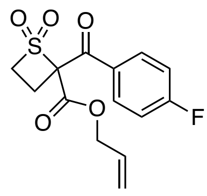

16e

A solution of NaHMDS (1 M in THF, 0.6 mL, 0.6 mmol) in THF (5 mL) was cooled to 0 ºC. A solution of allyl ester **15** (100 mg, 0.53 mmol) in THF (10 mL) was added dropwise and the reaction mixture was stirred at 0 ºC for 30 minutes. 4-Fluorobenzoyl chloride (71 μL, 0.6 mmol) was added dropwise and the mixture stirred at 0 ºC for 3 h. The reaction was quenched with aq. HCl (1 N, 1 mL), allowed to warm to room temperature and diluted with water (10 mL). The mixture was extracted with EtOAc (3 x 20 mL), washed with brine (10 mL), dried ($MgSO_4$) and concentrated under reduced pressure. Purification by flash column

chromatography [SiO_2; 10:1 hexane:EtOAc] afforded **16e** (41 mg, 25%) as a colourless oil. ***R*f** = 0.31 [2:1 petrol:EtOAc].

^{1}H NMR: (400 MHz, $CDCl_3$) δ 8.07 – 8.02 (2H, m), 7.21 – 7.15 (2H, m), 5.79 (1H, ddt, *J* = 17.6, 10.0, 5.6 Hz), 5.27 – 5.21 (2H, m), 4.75 (1H, ddt, *J* = 12.8, 6.0, 1.2 Hz), 4.68 (1H, ddt, *J* = 12.8, 6.0, 1.2 Hz), 4.48 (1H, dt, *J* = 12.8, 10.0 Hz), 4.10 (1H, ddd, *J* = 12.4, 10.0, 3.2 Hz), 2.96 (1H, dt, *J* = 12.8, 10.0 Hz), 2.78 (1H, ddd, *J* = 12.4, 10.4, 3.6 Hz).

^{13}C NMR: (100 MHz, $CDCl_3$) δ 186.2, 166.6 (d, *J* = 255.2 Hz), 164.4, 132.7 (d, *J* = 9.5 Hz), 131.3, 130.2, 120.3, 116.1 (d, *J* = 22.0 Hz), 95.6, 68.1, 63.6, 17.4.

^{19}F{^{1}H} NMR: (376 MHz, $CDCl_3$) δ –102.3.

HRMS: (APCI-TOF) m/z: $[M+H]^+$ calcd for $C_{14}H_{14}O_5FS$ 313.0540; found 313.0531.

IR: ν_{max} (neat/cm^{-1}): 3035, 1733, 1597, 1334, 1138.

Allyl 2-(4-bromobenzoyl)-1,1-dioxo-thietane-2-carboxylate (16f)

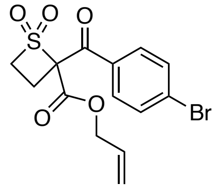

16f

A solution of NaHMDS (1 M in THF, 1.2 mL, 1.2 mmol) in THF (10 mL) was cooled to 0 ºC. A solution of allyl ester **15** (200 mg, 1.05 mmol) in THF (5 mL) was added dropwise and the reaction mixture was stirred at 0 ºC for 30 minutes. 4-Bromobenzoyl chloride (250 mg, 1.15 mmol) was added dropwise and the mixture stirred at 0 ºC for 4 h. The reaction was quenched with aq. HCl (1 N, 1 mL), allowed to warm to room temperature and diluted with water (10 mL). The mixture was extracted with EtOAc (3 x 20 mL), washed with brine (10 mL), dried ($MgSO_4$) and concentrated under reduced pressure. Purification by flash column chromatography [SiO_2; 7:1 hexane:EtOAc] afforded **16f** (291 mg, 75%) as a colourless oil. ***R*f** = 0.55 [2:1 petrol:EtOAc].

^{1}H NMR: (400 MHz, $CDCl_3$) δ 7.87 (2H, d, *J* = 8.8 Hz), 7.65 (2H, d, *J* = 8.8 Hz), 5.79 (1H, ddt, *J* = 17.4, 10.2, 5.9 Hz), 5.27 – 5.21 (2H, m), 4.74 (1H, ddt, *J* = 13.0, 5.8, 1.3 Hz), 4.68

(1H, ddt, J = 12.9, 6.0, 1.3 Hz), 4.48 (1H, dt, J = 12.5, 10.2 Hz), 4.09 (1H, ddd, J = 13.6, 10.3, 3.4 Hz), 2.94 (1H, dt, J = 12.5, 10.1 Hz), 2.77 (1H, ddd, J = 13.8, 10.4, 3.4 Hz).

^{13}C NMR: (100 MHz, $CDCl_3$) δ 186.9, 164.3, 133.6, 132.2, 131.2, 130.2, 130.1, 120.4, 95.6, 68.1, 63.7, 17.3.

HRMS: (APCI-TOF) m/z: $[M+H]^+$ calcd for $C_{14}H_{14}O_5S^{79}Br$ 372.9731; found 372.9740.

IR: ν_{max} (neat/cm^{-1}): 3084, 2963, 1735, 1681, 1323, 1138.

Allyl 2-(4-(methoxycarbonyl)benzoyl)-1,1-dioxo-thietane-2-carboxylate (16g)

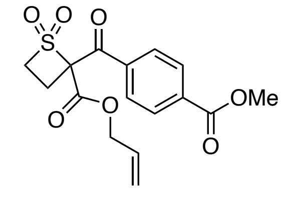

16g

A solution of NaHMDS (1 M in THF, 0.6 mL, 0.6 mmol) in THF (10 mL) was cooled to 0 ºC. A solution of allyl ester **15** (100 mg, 0.53 mmol) in THF (5 mL) was added dropwise and the reaction mixture was stirred at 0 ºC for 30 minutes. Methyl 4-(chlorocarbonyl)benzoate (120 mg, 0.6 mmol) in THF (5 mL) was added dropwise and the mixture stirred at 0 ºC for 3 h. The reaction was quenched with aq. HCl (1 N, 1 mL), allowed to warm to room temperature and diluted with water (10 mL). The mixture was extracted with EtOAc (3 x 20 mL), washed with brine (10 mL), dried ($MgSO_4$) and concentrated under reduced pressure. Purification by flash column chromatography [SiO_2; 10:1 hexane:EtOAc] afforded **16g** (46 mg, 25%) as a colourless solid. $\boldsymbol{R_f}$ = 0.41 [2:1 petrol:EtOAc]. **mp**: 120 – 122 ºC.

^{1}H NMR: (400 MHz, $CDCl_3$) δ 8.19 – 8.16 (2H, m), 8.07 – 8.04 (2H, m), 5.77 (1H, ddt, J = 17.4, 10.3, 5.9 Hz), 5.26 – 5.23 (1H, m), 5.22 – 5.19 (1H, m), 4.74 (1H, ddt, J = 12.8, 5.9, 1.2 Hz), 4.67 (1H, ddt, J = 12.9, 6.0, 1.3 Hz), 4.50 (1H, dt, J = 12.4, 10.3 Hz), 4.12 (1H, ddd, J = 13.7, 10.2, 3.4 Hz), 3.95 (3H, s), 2.96 (1H, dt, J = 12.5, 10.1 Hz), 2.78 (1H, ddd, J = 12.5, 10.4, 3.4 Hz).

^{13}C NMR: (100 MHz, $CDCl_3$) δ 187.5, 166.1, 164.3, 138.0, 135.0, 131.1, 129.9, 129.6, 120.4, 95.7, 68.1, 63.9, 52.7, 17.4.

HRMS: (APCI-TOF) m/z: $[M+H]^+$ calcd for $C_{16}H_{17}O_7S$ 353.0690; found 353.0677.

IR: ν_{max} (neat/cm^{-1}): 2978, 1731, 1686, 1334, 1138.

Allyl 2-(furan-2-carbonyl)-1,1-dioxo-thietane-2-carboxylate (16h)

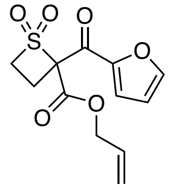

16h

A solution of NaHMDS (1 M in THF, 1.2 mL, 1.2 mmol) in THF (10 mL) was cooled to 0 ºC. A solution of allyl ester **15** (200 mg, 1.05 mmol) in THF (5 mL) was added dropwise and the reaction mixture was stirred at 0 ºC for 30 minutes. 2-Furoyl chloride (115 μL, 1.15 mmol) was added dropwise and the mixture stirred at 0 ºC for 4 h. The reaction was quenched with aq. HCl (1 N, 1 mL), allowed to warm to room temperature and diluted with water (10 mL). The mixture was extracted with EtOAc (3 x 20 mL), washed with brine (10 mL), dried ($MgSO_4$) and concentrated under reduced pressure. Purification by flash column chromatography [SiO_2; 9:1 – 5:1 hexane:EtOAc] afforded **16h** (155 mg, 52%) as a colourless oil. $\boldsymbol{R}_\mathbf{f}$ = 0.23 [3:1 petrol:EtOAc].

^{1}H NMR: (400 MHz, $CDCl_3$) δ 7.68 (1H, dd, *J* = 1.7, 0.8 Hz), 7.44 (1H, dd, *J* = 3.7, 0.8 Hz), 6.62 (1H, dd, *J* = 3.7, 1.7 Hz), 5.85 – 5.75 (1H, m), 5.25 (1H, dq, *J* = 6.6, 1.2 Hz), 5.21 (1H, t, *J* = 1.3 Hz), 4.76 (1H, ddt, *J* = 13.2, 5.6, 1.4 Hz), 4.67 (1H, ddt, *J* = 13.2, 5.8, 1.4 Hz), 4.52 – 4.43 (1H, m), 4.10 (1H, ddd, *J* = 12.6, 10.3, 3.4 Hz), 2.93 (1H, dt, *J* = 12.4, 10.0 Hz), 2.68 (1H, ddd, *J* = 12.6, 10.4, 3.4 Hz).

^{13}C NMR: (100 MHz, $CDCl_3$) δ 175.8, 164.1, 151.1, 148.0, 130.4, 120.5, 119.7, 113.2, 95.4, 67.8, 64.4, 16.2.

HRMS: (APCI-TOF) m/z: [M+H]$^+$ calcd for $C_{12}H_{13}O_6S$ 285.0427; found 285.0436.

IR: ν_{max} (neat/cm^{-1}): 3035, 2953, 1740, 1668, 1330, 1138.

Allyl 1,1-dioxo-2-(pyridine-3-carbonyl)thietane-2-carboxylate (16i)

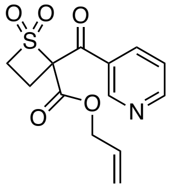

16i

According to a literature procedure,[2] to a suspension of nicotinic acid (175 mg, 1.42 mmol) in oxalyl chloride (1.75 mL, 20.6 mmol) was added DMF (1 drop) and the mixture was stirred at room temperature for 30 minutes. Oxalyl chloride was removed under reduced pressure and an aliquot of product was quenched with MeOH. TLC analysis confirmed methyl ester formation. Crude acid chloride was used in the next step without further analysis or purification.

A solution of NaHMDS (1 M in THF, 1.2 mL, 1.2 mmol) in THF (10 mL) was cooled to 0 ºC. A solution of allyl ester **15** (200 mg, 1.05 mmol) in THF (5 mL) was added dropwise and the reaction mixture was stirred at 0 ºC for 45 minutes. A solution of freshly prepared nicotinoyl chloride (1.42 mmol) in THF (10 mL) was added dropwise and the mixture stirred at 0 ºC for 4 h. The reaction was quenched with aq. HCl (1 N, 1 mL), allowed to warm to room temperature and diluted with water (10 mL). The mixture was extracted with EtOAc (3 x 20 mL), washed with brine (10 mL), dried ($MgSO_4$) and concentrated under reduced pressure. Purification by flash column chromatography [SiO_2; 9:1 hexane:EtOAc] afforded **16i** (55 mg, 17%) as a sticky colourless oil. $\boldsymbol{R}_\mathbf{f}$ = 0.41 [3:2 petrol:EtOAc].

^{1}H NMR: (400 MHz, $CDCl_3$) δ 9.19 (1H, s), 8.80 (1H, d, J = 3.8 Hz), 8.29 – 8.25 (1H, m), 7.45 (1H, dd, J = 7.8, 4.7 Hz), 5.77 (1H, ddt, J = 16.4, 9.8, 6.4 Hz), 5.27 – 5.23 (1H, m), 5.22 – 5.20 (1H, m), 4.76 – 4.65 (2H, m), 4.48 (1H, dt, J = 12.4, 10.2 Hz), 4.12 (1H, ddd, J = 13.8, 10.3, 3.5 Hz), 2.96 (1H, dt, J = 12.5, 9.9 Hz), 2.79 (1H, ddd, J = 14.0, 10.5, 3.6 Hz).

^{13}C NMR: (100 MHz, $CDCl_3$) δ 187.1, 163.8, 154.2, 150.8, 150.1, 137.2, 130.1, 123.5, 120.5, 95.6, 68.2, 64.0, 16.7.

HRMS: (APCI-TOF) m/z: $[M+H]^+$ calcd for $C_{13}H_{14}NO_5S$ 296.0587; found 296.0575.

IR: ν_{max} (neat/cm^{-1}): 3039, 2976, 1735, 1321, 1183.

2-Methylallyl 2-benzoyl-1,1-dioxo-thietane-2-carboxylate (16j)

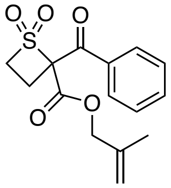

16j

A solution of NaHMDS (1 M in THF, 0.33 mL, 0.33 mmol) in THF (10 mL) was cooled to 0 ºC. A solution of allyl ester **31a** (50 mg, 0.25 mmol) in THF (5 mL) was added and the reaction mixture was stirred at 0 ºC for 15 minutes. Benzoyl chloride (40 µL, 0.33 mmol) was added dropwise and the mixture stirred at 0 ºC for 3 h. The reaction was quenched with aq. HCl (1 N, 1 mL), allowed to warm to room temperature and diluted with water (10 mL). The mixture was extracted with EtOAc (3 x 20 mL), washed with brine (10 mL), dried ($MgSO_4$) and concentrated under reduced pressure. Purification by flash column chromatography [SiO_2; 9:1 hexane:EtOAc] afforded **16j** (43 mg, 56%) as a colourless oil. $\boldsymbol{R}_f$ = 0.48 [2:1 petrol:EtOAc].

^{1}H NMR: (400 MHz, $CDCl_3$) δ 8.02 – 7.98 (2H, m), 7.63 (1H, tt, *J* = 7.4, 1.2 Hz), 7.53 – 7.48 (2H, m), 4.89 – 4.84 (2H, m), 4.65 (1H, d, *J* = 12.7 Hz), 4.59 (1H, d, *J* = 12.7 Hz), 4.48 (1H, dt, *J* = 12.4, 10.2 Hz), 4.10 (1H, ddd, *J* = 12.6, 10.2, 3.4 Hz), 2.97 (1H, dt, *J* = 12.5, 10.1 Hz), 2.78 (1H, ddd, *J* = 12.5, 10.4, 3.4 Hz), 1.56 (3H, s).

^{13}C NMR: (100 MHz, $CDCl_3$) δ 187.6, 164.6, 138.3, 134.8, 134.5, 129.7, 128.9, 115.1, 95.8, 70.9, 63.6, 19.3, 17.6.

HRMS: (APCI-TOF) m/z: $[M+H]^+$ calcd for $C_{15}H_{17}O_5S$ 309.0791; found 309.0779.

IR: ν_{max} (neat/cm^{-1}): 3035, 2976, 1731, 1682, 1332, 1138.

2-Methylallyl 2-(4-methylbenzoyl)-1,1-dioxo-thietane-2-carboxylate (16k)

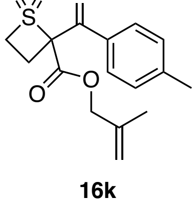

16k

A solution of NaHMDS (1 M in THF, 0.80 mL, 0.80 mmol) in THF (10 mL) was cooled to 0 ºC. A solution of allyl ester **31a** (125 mg, 0.60 mmol) in THF (5 mL) was added and the reaction mixture was stirred at 0 ºC for 30 minutes. *p*-Tolyl chloride (105 μL, 0.80 mmol) was added dropwise and the mixture stirred at 0 ºC for 3 h. The reaction was quenched with aq. HCl (1 N, 1 mL), allowed to warm to room temperature and diluted with water (10 mL). The mixture was extracted with EtOAc (3 x 20 mL), washed with brine (10 mL), dried ($MgSO_4$) and concentrated under reduced pressure. Purification by flash column chromatography [SiO_2; 9:1 hexane:EtOAc] afforded **16k** (114 mg, 59%) as a colourless oil. $\boldsymbol{R_f}$ = 0.66 [2:1 petrol:EtOAc].

^{1}H NMR: (400 MHz, $CDCl_3$) δ 7.90 (2H, d, *J* = 8.4 Hz), 7.29 (2H, d, *J* = 8.0 Hz), 4.89 – 4.86 (2H, m), 4.65 (1H, d, *J* = 12.8 Hz), 4.59 (1H, d, *J* = 12.8 Hz), 4.45 (1H, dt, *J* = 12.5, 10.2 Hz), 4.08 (1H, ddd, *J* = 12.5, 10.1, 3.4 Hz), 2.96 (1H, dt, *J* = 12.5, 10.1 Hz), 2.76 (1H, ddd, *J* = 12.5, 10.4, 3.4 Hz), 2.42 (3H, s), 1.59 (3H, s).

^{13}C NMR: (100 MHz, $CDCl_3$) δ 187.1, 164.7, 145.8, 138.3, 132.3, 129.9, 129.6, 114.9, 95.8, 70.8, 63.5, 22.0, 19.4, 17.7.

HRMS: (APCI-TOF) m/z: $[M+H]^+$ calcd for $C_{16}H_{19}O_5S$ 323.0948; found 323.0936.

IR: ν_{max} (neat/cm^{-1}): 3035, 2974, 1731, 1677, 1332, 1138.

2-Methylallyl 2-(4-methoxybenzoyl)-1,1-dioxo-thietane-2-carboxylate (16l)

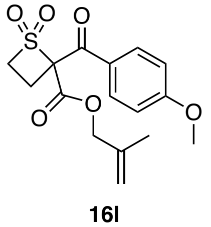

16l

A solution of NaHMDS (1 M in THF, 0.40 mL, 0.40 mmol) in THF (10 mL) was cooled to 0 ºC. A solution of allyl ester **31a** (75 mg, 0.36 mmol) in THF (5 mL) was added and the reaction mixture was stirred at 0 ºC for 15 minutes. 4-Methoxybenzoyl chloride (60 μL, 0.40 mmol) was added dropwise and the mixture stirred at 0 ºC for 3 h. The reaction was quenched with aq. HCl (1 N, 1 mL), allowed to warm to room temperature and diluted with water (10 mL). The mixture was extracted with EtOAc (3 x 20 mL), washed with brine (10 mL), dried ($MgSO_4$) and concentrated under reduced pressure. Purification by flash column

chromatography [SiO_2; 10:1 hexane:EtOAc] afforded **16l** (66 mg, 55%) as a colourless oil. ***R*f** = 0.43 [2:1 petrol:EtOAc].

^{1}H NMR: (400 MHz, $CDCl_3$) δ 7.99 (2H, d, *J* = 9.1 Hz), 6.97 (2H, d, *J* = 9.0 Hz), 4.91 – 4.85 (2H, m), 4.65 (1H, d, *J* = 17.0 Hz), 4.60 (1H, d, *J* = 17.0 Hz), 4.44 (1H, dt, *J* = 12.5, 10.3 Hz), 4.06 (1H, ddd, *J* = 12.6, 10.2, 3.4 Hz), 3.87 (3H, s), 2.96 (1H, dt, *J* = 12.5, 10.1 Hz), 2.77 (1H, ddd, *J* = 12.5, 10.4, 3.4 Hz), 1.60 (3H, s).

^{13}C NMR: (100 MHz, $CDCl_3$) δ 185.9, 164.7, 164.7, 138.3, 132.3, 127.8, 114.4, 114.9, 95.8, 70.8, 63.3, 55.7, 19.4, 17.6.

HRMS: (APCI-TOF) m/z: $[M+H]^+$ calcd for $C_{16}H_{19}O_6S$ 339.0897; found 339.0883.

IR: ν_{max} (neat/cm^{-1}): 2972, 2939, 1731, 1332, 1136.

(*E*)-Cinnamyl 2-benzoyl-1,1-dioxo-thietane-2-carboxylate (16m)

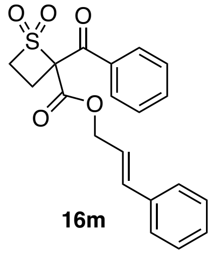

16m

A solution of NaHMDS (1 M in THF, 0.30 mL, 0.30 mmol) in THF (5 mL) was cooled to 0 ºC. A solution of allyl ester **31b** (60 mg, 0.23 mmol) in THF (5 mL) was added and the reaction mixture was stirred at 0 ºC for 15 minutes. Benzoyl chloride (38 µL, 0.30 mmol) was added dropwise and the reaction mixture was stirred at 0 ºC for 4 h. The reaction was quenched with aq. HCl (1 N, 1 mL), allowed to warm to room temperature and diluted with water (10 mL). The mixture was extracted with EtOAc (3 x 20 mL), washed with brine (10 mL), dried ($MgSO_4$) and concentrated under reduced pressure. Purification by flash column chromatography [SiO_2; 10:1 – 6:1 hexane:EtOAc] afforded **16m** (51 mg, 60%) as a colourless oil. ***R*f** = 0.37 [2:1 petrol:EtOAc].

^{1}H NMR: (400 MHz, $CDCl_3$) δ 8.02 – 7.98 (2H, m), 7.60 (1H, tt, *J* = 7.4, 1.2 Hz), 7.51 – 7.45 (2H, m), 7.34 – 7.26 (5H, m), 6.53 (1H, d, *J* = 15.9 Hz), 6.10 (1H, dt, *J* = 15.9, 6.5 Hz), 4.90 (1H, ddd, *J* = 12.7, 6.4, 1.3 Hz), 4.83 (1H, ddd, *J* = 12.6, 6.5, 1.3 Hz), 4.48 (1H, dt, *J* = 12.2,

10.3 Hz), 4.10 (1H, ddd, J = 12.6, 10.2, 3.6 Hz), 2.99 (1H, dt, J = 12.4, 10.1 Hz), 2.78 (1H, ddd, J = 12.5, 10.4, 3.5 Hz).

^{13}C NMR: (100 MHz, $CDCl_3$) δ 187.6, 164.7, 135.9, 135.8, 134.5, 129.7, 128.9, 128.8, 128.6, 126.9, 121.0, 95.8, 67.9, 63.7, 17.5.

HRMS: (APCI-TOF) m/z: $[M+Na]^+$ calcd for $C_{20}H_{18}O_5SNa$ 393.0767; found 393.0773.

IR: ν_{max} (neat/cm^{-1}): 3058, 2965, 1735, 1675, 1338, 1138.

3-Methylbut-2-enyl 2-benzoyl-1,1-dioxo-thietane-2-carboxylate (16n)

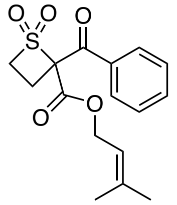

16n

A solution of NaHMDS (1 M in THF, 0.25 mL, 0.25 mmol) in THF (5 mL) was cooled to 0 ºC. A solution of allyl ester **31c** (40 mg, 0.18 mmol) in THF (5 mL) was added and the reaction mixture was stirred at 0 ºC for 15 minutes. Benzoyl chloride (30 µL, 0.25 mmol) was added dropwise and the reaction mixture was stirred at 0 ºC for 4 h. The reaction was quenched with aq. HCl (1 N, 1 mL), allowed to warm to room temperature and diluted with water (10 mL). The mixture was extracted with EtOAc (3 x 20 mL), washed with brine (10 mL), dried ($MgSO_4$) and concentrated under reduced pressure. Purification by flash column chromatography [SiO_2; 10:1 hexane:EtOAc] afforded **16n** (37 mg, 64%) as a yellow oil. R_f = 0.69 [3:2 petrol:EtOAc].

^{1}H NMR: (400 MHz, $CDCl_3$) δ 7.77 – 7.72 (2H, m), 7.39 (1H, tt, J = 7.4, 1.2 Hz), 7.29 – 7.24 (2H, m), 4.99 – 4.93 (1H, m), 4.50 (1H, dd, J = 12.0, 7.3 Hz), 4.42 (1H, dd, J = 12.0, 7.4 Hz), 4.27 – 4.18 (1H, m), 3.81 (1H, ddd, J = 12.5, 10.2, 3.5 Hz), 2.72 (1H, dt, J = 12.4, 10.1 Hz), 2.51 (1H, ddd, J = 12.4, 10.4, 3.5 Hz), 1.45 (3H, s), 1.35 (3H, s).

^{13}C NMR: (100 MHz, $CDCl_3$) δ 187.7, 164.8, 141.7, 134.9, 134.4, 129.7, 128.7, 116.8, 95.8, 64.3, 63.6, 25.8, 18.1, 17.5.

HRMS: (ESI-TOF) m/z: $[M+Na]^+$ calcd for $C_{16}H_{18}O_5SNa$ 345.0767; found 345.0758.

IR: ν_{max} (neat/cm^{-1}): 3032, 2974, 2935, 1727, 1684, 1334, 1138.

Allyl 2-(adamantane-1-carbonyl)-1,1-dioxo-thietane-2-carboxylate (16o)

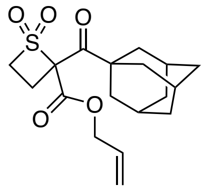

16o

A solution of NaHMDS (1 M in THF, 1.2 mL, 1.2 mmol) in THF (10 mL) was cooled to 0 ºC. A solution of allyl ester **15** (200 mg, 1.05 mmol) in THF (5 mL) was added dropwise and the reaction mixture was stirred at 0 ºC for 30 minutes. 1-Adamantanecarbonyl chloride (230 mg, 1.15 mmol) was added dropwise and the mixture stirred at 0 ºC for 4 h. The reaction was quenched with aq. HCl (1 N, 1 mL), allowed to warm to room temperature and diluted with water (10 mL). The mixture was extracted with EtOAc (3 x 20 mL), washed with brine (10 mL), dried ($MgSO_4$) and concentrated under reduced pressure. Purification by flash column chromatography [SiO_2; 10:1 hexane:EtOAc] afforded **16o** (145 mg, 40%) as a colourless oil. $\boldsymbol{R_f}$ = 0.53 [2:1 petrol:EtOAc].

^{1}H NMR: (400 MHz, $CDCl_3$) δ 6.00 – 5.89 (1H, m), 5.42 (1H, dq, *J* = 17.2, 1.3 Hz), 5.34 (1H, dq, *J* = 10.4, 1.0 Hz), 4.77 (2H, dq, *J* = 6.2, 1.1 Hz), 4.32 (1H, dt, *J* = 12.4, 10.0 Hz), 3.95 (1H, ddd, *J* = 13.2, 10.1, 3.1 Hz), 2.85 (1H, dt, *J* = 12.4, 10.0 Hz), 2.47 (1H, ddd, *J* = 12.4, 10.2, 3.1 Hz), 2.06 – 2.00 (3H, m), 1.99 – 1.96 (6H, m), 1.73 – 1.68 (6H, m).

^{13}C NMR: (100 MHz, $CDCl_3$) δ 203.1, 165.0, 130.4, 121.1, 97.0, 67.9, 62.8, 47.4, 38.3, 36.5, 28.1, 18.3.

HRMS: (APCI-TOF) m/z: $[M+H]^+$ calcd for $C_{18}H_{25}O_5S$ 353.1417; found 353.1403.

IR: ν_{max} (neat/cm^{-1}): 2905, 2851, 1733, 1697, 1332, 1136.

Allyl 2-(2,2-dimethylpropanoyl)-1,1-dioxo-thietane-2-carboxylate (16p)

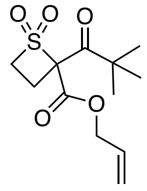

16p

A solution of NaHMDS (1 M in THF, 1.2 mL, 1.2 mmol) in THF (10 mL) was cooled to 0 ºC. A solution of allyl ester **15** (200 mg, 1.05 mmol) in THF (5 mL) was added dropwise and the reaction mixture was stirred at 0 ºC for 30 minutes. Trimethylacetyl chloride (141 μL, 1.15 mmol) was added dropwise and the mixture stirred at 0 ºC for 4 h. The reaction was quenched with aq. HCl (1 N, 1 mL), allowed to warm to room temperature and diluted with water (10 mL). The mixture was extracted with EtOAc (3 x 20 mL), washed with brine (10 mL), dried ($MgSO_4$) and concentrated under reduced pressure. Purification by flash column chromatography [SiO_2; 10:1 hexane:EtOAc] afforded **16p** (179 mg, 62%) as a colourless solid. $\boldsymbol{R}_f$ = 0.39 [3:1 petrol:EtOAc]. **mp**: 74 – 76 ºC.

^{1}H NMR: (400 MHz, $CDCl_3$) δ 5.99 – 5.88 (1H, m), 5.42 (1H, dq, J = 17.2, 1.3 Hz), 5.34 (1H, dq, J = 10.4, 1.1 Hz), 4.76 (2H, dq, J = 6.1, 1.2 Hz), 4.34 (1H, dt, J = 12.4, 10.0 Hz), 4.00 (1H, ddd, J = 13.3, 10.1, 3.2 Hz), 2.84 (1H, dt, J = 12.4, 10.0 Hz), 2.51 (1H, ddd, J = 13.5, 10.3, 3.2 Hz), 1.27 (9H, s).

^{13}C NMR: (100 MHz, $CDCl_3$) δ 204.1, 164.9, 130.3, 121.0, 97.2, 67.9, 62.9, 44.9, 27.5, 18.4.

HRMS: (APCI-TOF) m/z: $[M+H]^+$ calcd for $C_{12}H_{19}O_5S$ 275.0948; found 275.0949.

IR: ν_{max} (neat/cm^{-1}): 3041, 2976, 2875, 1733, 1701, 1332, 1136.

Allyl 2-(cyclohexanecarbonyl)-1,1-dioxo-thietane-2-carboxylate (16q)

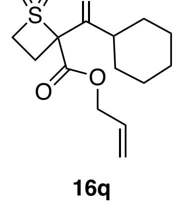

16q

A solution of NaHMDS (1 M in THF, 1.2 mL, 1.2 mmol) in THF (10 mL) was cooled to 0 ºC. A solution of allyl ester **15** (200 mg, 1.05 mmol) in THF (5 mL) was added dropwise and the reaction mixture was stirred at 0 ºC for 30 minutes. Cyclohexane carbonyl chloride (154 μL, 1.15 mmol) was added dropwise and the mixture stirred at 0 ºC for 4 h. The reaction was quenched with aq. HCl (1 N, 1 mL), allowed to warm to room temperature and diluted with water (10 mL). The mixture was extracted with EtOAc (3 x 20 mL), washed with brine (10 mL), dried ($MgSO_4$) and concentrated under reduced pressure. Purification by flash column chromatography [SiO_2; 9:1 hexane:EtOAc] afforded **16q** (227 mg, 72%) as a colourless oil. R_f = 0.39 [3:1 petrol:EtOAc].

^{1}H NMR: (400 MHz, $CDCl_3$) δ 6.01 – 5.91 (1H, m), 5.44 (1H, dq, *J* = 17.2, 1.3 Hz), 5.36 (1H, dq, *J* = 10.4, 1.1 Hz), 4.79 (2H, dq, *J* = 6.2, 1.2 Hz), 4.13 – 3.99 (2H, m), 3.07 – 2.98 (1H, m), 2.86 (1H, ddd, *J* = 16.2, 9.9, 6.2 Hz), 2.56 (1H, ddd, *J* = 18.1, 10.1, 7.8 Hz), 2.05 – 1.16 (10H, m).

^{13}C NMR: (100 MHz, $CDCl_3$) δ 199.3, 162.9, 130.5, 120.8, 98.7, 68.1, 63.6, 49.7, 30.4, 28.5, 25.7, 25.7, 25.4, 14.8.

HRMS: (APCI-TOF) m/z: $[M+H]^+$ calcd for $C_{14}H_{21}O_5S$ 301.1104; found 301.1101.

IR: ν_{max} (neat/cm^{-1}): 2929, 2853, 1735, 1699, 1328, 1132.

Allyl 2-(2-methylpropanoyl)-1,1-dioxo-thietane-2-carboxylate (16r)

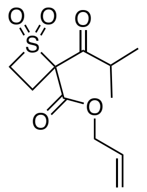

16r

A solution of NaHMDS (1 M in THF, 1.2 mL, 1.2 mmol) in THF (10 mL) was cooled to 0 ºC. A solution of allyl ester **15** (200 mg, 1.05 mmol) in THF (5 mL) was added dropwise and the reaction mixture was stirred at 0 ºC for 30 minutes. Isobutyryl chloride (119 μL, 1.15 mmol) was added dropwise and the mixture stirred at 0 ºC for 4 h. The reaction was quenched with aq. HCl (1 N, 1 mL), allowed to warm to room temperature and diluted with water (10 mL). The mixture was extracted with EtOAc (3 x 20 mL), washed with brine (10 mL), dried ($MgSO_4$) and concentrated under reduced pressure. Purification by flash column

chromatography [SiO_2; 9:1 hexane:EtOAc] afforded **16r** (198 mg, 73%) as a colourless oil. ***R*f** = 0.56 [3:1 petrol:EtOAc].

^{1}H NMR: (400 MHz, $CDCl_3$) δ 5.99 – 5.88 (1H, m), 5.41 (1H, dq, *J* = 17.2, 1.3 Hz), 5.32 (1H, dq, *J* = 10.4, 1.2 Hz), 4.83 – 4.71 (2H, m), 4.14 – 3.99 (2H, m), 3.26 (1H, sept, *J* = 6.7 Hz), 2.83 (1H, ddd, *J* = 12.4, 10.1, 6.3 Hz), 2.56 (1H, ddd, *J* = 12.3, 10.3, 7.6 Hz), 1.18 (3H, d, *J* = 6.8 Hz), 1.07 (3H, d, *J* = 6.8 Hz).

^{13}C NMR: (100 MHz, $CDCl_3$) δ 200.8, 162.9, 130.5, 120.6, 98.5, 68.0, 63.7, 39.6, 20.0, 18.8, 15.0.

HRMS: (APCI-TOF) m/z: $[M+H]^+$ calcd for $C_{11}H_{17}O_5S$ 261.0791; found 261.0782.

IR: ν_{max} (neat/cm^{-1}): 3032, 2980, 1736, 1710, 1332, 1138.

Allyl 2-(3,3-dimethylbutanoyl)-1,1-dioxo-thietane-2-carboxylate (16s)

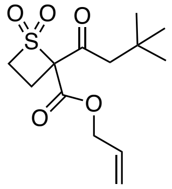

16s

A solution of NaHMDS (1 M in THF, 1.2 mL, 1.2 mmol) in THF (10 mL) was cooled to 0 ºC. A solution of allyl ester **15** (200 mg, 1.05 mmol) in THF (5 mL) was added dropwise and the reaction mixture was stirred at 0 ºC for 30 minutes. *tert*-Butyl acetyl chloride (154 µL, 1.15 mmol) was added dropwise and the mixture stirred at 0 ºC for 4 h. The reaction was quenched with aq. HCl (1 N, 1 mL), allowed to warm to room temperature and diluted with water (10 mL). The mixture was extracted with EtOAc (3 x 20 mL), washed with brine (10 mL), dried ($MgSO_4$) and concentrated under reduced pressure. Purification by flash column chromatography [SiO_2; 10:1 hexane:EtOAc] afforded **16s** (184 mg, 57%) as a colourless oil. ***R*f** = 0.55 [3:1 petrol:EtOAc].

^{1}H NMR: (400 MHz, $CDCl_3$) δ 5.95 (1H, ddt, *J* = 17.2, 10.4, 6.0 Hz), 5.42 (1H, dq, *J* = 17.2, 1.3 Hz), 5.34 (1H, dq, *J* = 10.4, 1.1 Hz), 4.78 (2H, dt, *J* = 6.0, 1.2 Hz), 4.12 (1H, ddd, *J* = 12.6, 10.4, 6.6 Hz), 4.02 (1H, ddd, *J* = 12.6, 10.4, 6.8 Hz), 2.92 – 2.77 (3H, m), 2.57 (1H, ddd, *J* = 12.4, 10.4, 6.8 Hz), 1.04 (9H, s).

^{13}C NMR: (100 MHz, $CDCl_3$) δ 194.8, 162.9, 130.6, 120.6, 99.0, 68.0, 63.5, 53.7, 31.1, 29.5, 14.9.

HRMS: (APCI-TOF) m/z: $[M+H]^+$ calcd for $C_{13}H_{21}O_5S$ 289.1104, found 289.1094.

IR: ν_{max} (neat/cm^{-1}): 2955, 2871, 1736, 1718, 1334, 1135.

Allyl 1,1-dioxo-2-propanoyl-thietane-2-carboxylate (16t)

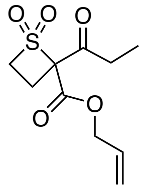

16t

A solution of NaHMDS (1 M in THF, 0.6 mL, 0.6 mmol) in THF was cooled to 0 °C. A solution of allyl ester **15** (100 mg, 0.53 mmol) in THF (5 mL) was added dropwise and the reaction mixture was stirred at 0 °C for 30 minutes. Propionyl chloride (53 μL, 0.6 mmol) was added dropwise and the mixture stirred at 0 °C for 3 h. The reaction was quenched with aq. HCl (1 N, 1 mL), allowed to warm to room temperature and diluted with water (10 mL). The mixture was extracted with EtOAc (3 x 20 mL), washed with brine (20 mL), dried ($MgSO_4$) and concentrated under reduced pressure. Purification by flash column chromatography [SiO_2; 10:1 hexane:EtOAc] afforded **16t** (64 mg, 50%) as a colourless oil. R_f = 0.49 [2:1 petrol:EtOAc].

^{1}H NMR: (400 MHz, $CDCl_3$) δ 5.95 (1H, ddt, J = 17.2, 10.4, 5.9 Hz), 5.41 (1H, dq, J = 17.2, 10.3 Hz), 5.34 (1H, dq, J = 10.4, 1.1 Hz), 4.79 (2H, dq, J = 6.0, 1.6 Hz), 4.16 (1H, ddd, J = 12.7, 10.4, 6.7 Hz), 4.06 (1H, ddd, J = 12.7, 10.3, 6.9 Hz), 3.03 – 2.79 (3H, m), 2.62 (1H, ddd, J = 12.4, 10.4, 6.9 Hz), 1.15 (3H, t, J = 7.1 Hz).

^{13}C NMR: (100 MHz, $CDCl_3$) δ 197.2, 162.9, 130.5, 120.5, 98.4, 68.1, 63.9, 35.8, 14.8, 8.0.

HRMS: (APCI-TOF) m/z: $[M+H]^+$ calcd for $C_{10}H_{15}O_5S$ 247.0635; found 247.0638.

IR: ν_{max} (neat/cm^{-1}): 3030, 2983, 2944, 1738, 1712, 1332, 1138.

Allyl 2-acetyl-1,1-dioxo-thietane-2-carboxylate (16u)

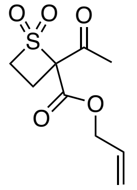

16u

A solution of NaHMDS (1 M in THF, 1.2 mL, 1.2 mmol) in THF (10 mL) was cooled to 0 ºC. A solution of allyl ester **15** (200 mg, 1.05 mmol) in THF (5 mL) was added dropwise and the reaction mixture was stirred at 0 ºC for 30 minutes. Acetyl chloride (82 µL, 1.15 mmol) was added dropwise and the mixture stirred at 0 ºC for 4 h. The reaction was quenched with aq. HCl (1 N, 0.5 mL), allowed to warm to room temperature and diluted with water (10 mL). The mixture was extracted with EtOAc (3 x 15 mL), washed with brine (10 mL), dried ($MgSO_4$) and concentrated under reduced pressure. Purification by flash column chromatography [SiO_2; 10:1 hexane:EtOAc] afforded **16u** (100 mg, 41%) as a colourless oil. $\boldsymbol{R}_f$ = 0.42 [3:1 petrol:EtOAc].

^{1}H NMR: (400 MHz, $CDCl_3$) δ 6.00 – 5.89 (1H, m), 5.41 (1H, dq, *J* = 17.2, 1.4 Hz), 5.34 (1H, dq, *J* = 10.4, 1.1 Hz), 4.81 – 4.78 (2H, m), 4.17 (1H, ddd, *J* = 12.7, 10.5, 6.7 Hz), 4.06 (1H, ddd, *J* = 12.8, 10.5, 6.7 Hz), 2.83 (1H, ddd, *J* = 12.4, 10.5, 6.8 Hz), 2.63 (1H, ddd, *J* = 12.5, 10.6, 6.7 Hz), 2.56 (3H, s).

^{13}C NMR: (100 MHz, $CDCl_3$) δ 194.0, 162.6, 130.5, 120.5, 98.6, 68.1, 64.0, 29.8, 14.5.

HRMS: (APCI-TOF) m/z: $[M+H]^+$ calcd for $C_9H_{13}O_5S$ 233.0478; found 233.0473.

IR: ν_{max} (neat/cm^{-1}): 3030, 2980, 1736, 1710, 1332, 1136.

2-Allyl 2'-phenyl 1,1-dioxothietane-2,2-dicarboxylate (17a)

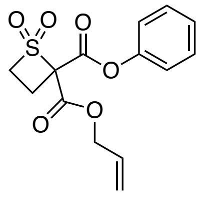

17a

A solution of KHMDS (0.5 M in toluene, 2.4 mL, 1.2 mmol) in THF (10 mL) was cooled to 0 ºC. A solution of allyl ester **15** (200 mg, 1.05 mmol) in THF (5 mL) was added dropwise and the reaction mixture was stirred at 0 ºC for 30 minutes. Phenyl chloroformate (170 μL, 1.15 mmol) was added dropwise and the mixture was stirred at 0 ºC for 4 h. The reaction was quenched with aq. HCl (1 N, 1 mL), allowed to warm to room temperature and diluted with water (10 mL). The mixture was extracted with EtOAc (3 x 20 mL), washed with brine (10 mL), dried ($MgSO_4$) and concentrated under reduced pressure. Purification by flash column chromatography [SiO_2; 6:1 hexane:EtOAc] afforded **17a** (268 mg, 77%) as a colourless solid. $\boldsymbol{R}_f$ = 0.48 [2:1 petrol:EtOAc]. **mp**: 58 – 60 ºC.

^{1}H NMR: (400 MHz, $CDCl_3$) δ 7.44 – 7.38 (2H, m), 7.29 (1H, tt, J = 7.6, 1.2 Hz), 7.19 – 7.16 (2H, m), 5.98 (1H, ddt, J = 17.2, 10.4, 5.6 Hz), 5.46 (1H, dq, J = 17.2, 1.2 Hz), 5.34 (1H, dq, J = 10.4, 1.2 Hz), 4.87 (2H, dt, J = 5.6, 1.6 Hz), 4.43 – 4.26 (2H, m), 2.83 (2H, ddd, J = 13.6, 8.0, 6.8 Hz).

^{13}C NMR: (100 MHz, $CDCl_3$) δ 162.8, 162.1, 150.4, 130.6, 129.8, 126.9, 121.3, 120.0, 92.6, 68.2, 65.2, 17.2.

HRMS: (APCI-TOF) m/z: $[M+H]^+$ calcd for $C_{14}H_{15}O_6S$ 311.0584; found 311.0588.

IR: ν_{max} (neat/cm^{-1}): 3030, 2939, 2972, 1742, 1332, 1177.

2-Allyl 2'-(4-methoxyphenyl) 1,1-dioxothietane-2,2-dicarboxylate (17b)

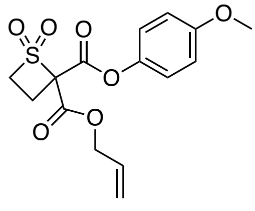

17b

A solution of KHMDS (0.5 M in toluene, 2.4 mL, 1.2 mmol) in THF (10 mL) was cooled to 0 ºC. A solution of allyl ester **15** (200 mg, 1.05 mmol) in THF (5 mL) was added dropwise and the reaction mixture was stirred at 0 ºC for 30 minutes. 4-Methoxyphenyl chloroformate (160 μL, 1.15 mmol) was added dropwise and the mixture was stirred at 0 ºC for 4 h. The reaction was quenched with aq. HCl (1 N, 1 mL), allowed to warm to room temperature and diluted with water (10 mL). The mixture was extracted with EtOAc (3 x 20 mL), washed with

brine (10 mL), dried ($MgSO_4$) and concentrated under reduced pressure. Purification by flash column chromatography [SiO_2; 20:1 – 10:1 hexane:EtOAc] afforded **17b** (271 mg, 76%) as a colourless oil. ***R*f** = 0.20 [3:2 petrol:EtOAc].

^{1}H NMR: (400 MHz, $CDCl_3$) δ 7.11 (2H, dt, *J* = 9.2, 3.0 Hz), 6.92 (2H, dt, *J* = 9.2, 3.0 Hz), 5.99 (1H, ddt, *J* = 17.2, 10.4, 5.8 Hz), 5.47 (1H, dq, *J* = 17.2, 1.4 Hz), 5.35 (1H, dq, *J* = 10.5, 1.2 Hz), 4.87 (2H, dt, *J* = 5.7, 1.4 Hz), 4.42 – 4.28 (2H, m), 3.83 (3H, s), 2.90 – 2.77 (2H, m).

^{13}C NMR: (100 MHz, $CDCl_3$) δ 162.9, 162.4, 158.1, 143.9, 130.6, 122.1, 120.0, 114.7, 92.6, 68.1, 65.2, 55.8, 17.2.

HRMS: (APCI-TOF) m/z: $[M+H]^+$ calcd for $C_{15}H_{17}O_7S$ 341.0690; found 341.0687.

IR: ν_{max} (neat/cm^{-1}): 3002, 2955, 1736, 1340, 1176.

2-Allyl 2'-(*p*-tolyl) 1,1-dioxothietane-2,2-dicarboxylate (17c)

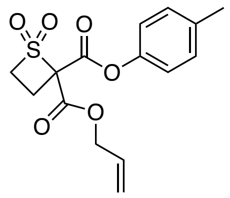

17c

A solution of KHMDS (0.5 M in toluene, 2.4 mL, 1.2 mmol) in THF (10 mL) was cooled to 0 ºC. A solution of allyl ester **15** (200 mg, 1.05 mmol) in THF (5 mL) was added dropwise and the reaction mixture was stirred at 0 ºC for 30 minutes. *p*-Tolyl chloroformate (165 μL, 1.15 mmol) was added dropwise and the mixture was stirred at 0 ºC for 4 h. The reaction was quenched with aq. HCl (1 N, 1 mL), allowed to warm to room temperature and diluted with water (10 mL). The mixture was extracted with EtOAc (3 x 20 mL), washed with brine (10 mL), dried ($MgSO_4$) and concentrated under reduced pressure. Purification by flash column chromatography [SiO_2; 10:1 hexane:EtOAc] afforded **17c** (250 mg, 73%) as a colourless solid. ***R*f** = 0.57 [2:1 petrol:EtOAc]. **mp**: 58 – 59 ºC.

^{1}H NMR: (400 MHz, $CDCl_3$) δ 7.22 – 7.17 (2H, m), 7.05 (2H, d, *J* = 8.4 Hz), 5.96 (1H, ddt, *J* = 17.2, 10.5, 5.7 Hz), 5.45 (1H, dq, *J* = 17.2, 1.5 Hz), 5.33 (1H, dq, *J* = 10.4, 1.2 Hz), 4.86 (2H, dt, *J* = 5.7, 1.3 Hz), 4.40 – 4.26 (2H, m), 2.88 – 2.76 (2H, m), 2.34 (3H, s).

^{13}C NMR: (100 MHz, $CDCl_3$) δ 162.9, 162.2, 148.2, 136.7, 130.6, 130.3, 120.9, 120.0, 92.6, 68.1, 65.2, 21.1, 17.2.

HRMS: (APCI-TOF) m/z: $[M+H]^+$ calcd for $C_{15}H_{17}O_6S$ 325.0740; found 325.0749.

IR: ν_{max} (neat/cm^{-1}): 3047, 2994, 2950, 1735, 1334, 1190.

2-Allyl 2'-*tert*-butyl 1,1-dioxothietane-2,2-dicarboxylate (17d)

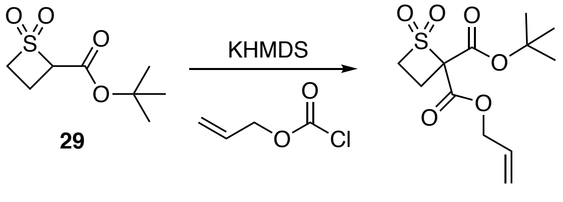

A solution of KHMDS (0.5 M in toluene, 64.1 mL, 32.0 mmol) in THF (180 mL) was cooled to 0 ºC. A solution of *tert*-butyl ester **29** (6.0 g, 29.1 mmol) in THF (20 mL) was added dropwise and the reaction mixture was stirred at 0 ºC for 15 minutes. Allyl chloroformate (3.41 mL, 32.0 mmol) was added dropwise and the reaction mixture was stirred at 0 ºC for 3 h. The reaction was quenched with aq. HCl (1 N, 30 mL), allowed to warm to room temperature and diluted with water (100 mL). The mixture was extracted with EtOAc (3 x 100 mL), washed with brine (100 mL), dried ($MgSO_4$) and concentrated under reduced pressure. Purification by flash column chromatography [SiO_2; 19:1 – 9:1 hexane:EtOAc] afforded **17d** (5.50 g, 60%) as a colourless oil. R_f = 0.70 [2:1 petrol:EtOAc].

^{1}H NMR: (400 MHz, $CDCl_3$) δ 5.98 – 5.88 (1H, m), 5.41 (1H, dq, *J* = 17.2, 1.4 Hz), 5.30 (1H, dd, *J* = 10.4, 1.2 Hz), 4.77 (2H, dt, *J* = 5.8, 1.3 Hz), 4.23 – 4.15 (2H, m), 2.73 – 2.57 (2H, m), 1.52 (9H, s).

^{13}C NMR: (100 MHz, $CDCl_3$) δ 163.4, 161.8, 130.9, 119.6, 93.3, 85.5, 67.6, 64.5, 27.9, 17.2.

HRMS: (APCI-TOF) m/z: $[M+Na]^+$ calcd for $C_{12}H_{18}O_6SNa$ 313.0716; found 313.0719.

IR: ν_{max} (neat/cm^{-1}): 2981, 2937, 1735, 1340, 1138.

2'-Allyl 2-methyl 1,1-dioxothietane-2,2-dicarboxylate (17e)

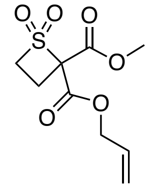

17e

A solution of KHMDS (0.5 M in toluene, 1.2 mL, 0.60 mmol) in THF (10 mL) was cooled to 0 ºC. A solution of allyl ester **15** (100 mg, 0.53 mmol) in THF (5 mL) was added dropwise and the reaction mixture was stirred at 0 ºC for 30 minutes. Methyl chloroformate (48 μL, 0.60 mmol) was added dropwise and the mixture was stirred at 0 ºC for 3 h. The reaction was quenched with aq. HCl (1 N, 1 mL), allowed to warm to room temperature and diluted with water (10 mL). The mixture was extracted with EtOAc (3 x 20 mL), washed with brine (10 mL), dried ($MgSO_4$) and concentrated under reduced pressure. Purification by flash column chromatography [SiO_2; 5:1 hexane:EtOAc] afforded **17e** (90 mg, 69%) as a colourless oil. $\boldsymbol{R}_f$ = 0.31 [3:1 petrol:EtOAc].

^{1}H NMR: (400 MHz, $CDCl_3$) δ 5.92 (1H, ddt, J = 17.2, 10.5, 5.7 Hz), 5.40 (1H, dq, J = 17.2, 1.4 Hz), 5.30 (1H, dq, J = 10.5, 1.2 Hz), 4.78 (2H, dt, J = 5.7, 1.4 Hz), 4.27 – 4.21 (2H, m), 3.90 (3H, s), 2.75 – 2.68 (2H, m).

^{13}C NMR: (100 MHz, $CDCl_3$) δ 163.6, 162.8, 130.7, 119.6, 92.5, 67.9, 64.8, 54.2, 17.1.

HRMS: (APCI-TOF) m/z: $[M+H]^+$ calcd for $C_9H_{13}O_6S$ 249.0427; found 249.0428.

IR: ν_{max} (neat/cm^{-1}): 3035, 2957, 1735, 1340, 1142.

2.4 Palladium-Catalysed Decarboxylative Allylic Alkylation

Table 1. Development of Palladium-Catalysed Decarboxylative Asymmetric Allylic Alkylation

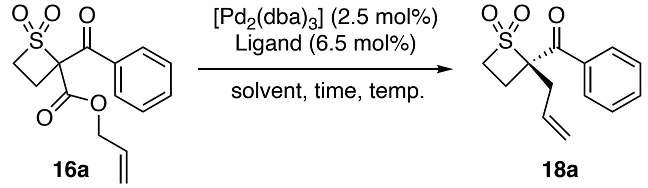

16a **18a**

Entry[a]	Ligand	Solvent	Temp (°C)	Conc (M)	Time (h)	Yield (%)[b]	ee (%)[c]
1	**L1**	1,4-dioxane	rt	0.04	6	38	8
2	**L2**	1,4-dioxane	rt	0.04	6	80	34
3	**L3**	1,4-dioxane	rt	0.04	6	80	24
**4**	**L4**	**1,4-dioxane**	**rt**	**0.04**	**6**	**74**	**83**
5	**L4**	MeCN	rt	0.04	6	71	1
6	**L4**	MTBE	rt	0.04	6	82	20
7	**L4**	PhMe	rt	0.04	6	78	22
8	**L4**	Et_2O	rt	0.04	6	71	38
9	**L4**	DME	rt	0.04	6	71	42
10	**L4**	THF	rt	0.04	6	70	41
11	**L4**	CH_2Cl_2	rt	0.04	6	98	65
12	**L4**	$CHCl_3$	rt	0.04	6	82	74
13	**L4**	2:1 1,4-dioxane:THF	0	0.04	8	80	74
14	**L4**	3:1 1,4-dioxane:THF	0	0.04	8	75	78
15	**L4**	3:1 1,4-dioxane:CH_2Cl_2	0	0.04	8	82	81
16	**L4**	1,4-dioxane	rt	0.1	6	84	79
17	**L4**	1,4-dioxane	rt	0.2	6	80	75

Conditions: Sulfone **16a** (0.17 mmol), $[Pd_2(dba)_3]$ (2.5 mol%), ligand (**L1** –**L4**) (6.5 mol%); [b] isolated yield; [c] enantiomeric excess determined by chiral HPLC; [d] performed at 0 °C. dba = dibenzylideneacetone.

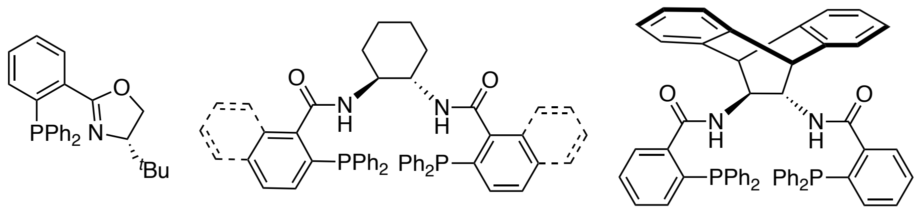

L1 **L2** (phenyl) **L3** (naphthyl) **L4**

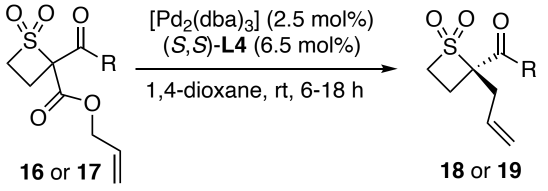

For the purposes of chiral HPLC analysis, all racemic products were prepared using 10 mol% $Pd(PPh_3)_4$ in 1,4-dioxane at rt for 18 h.

(2*S*)-[2-Allyl-1,1-dioxo-thietan-2-yl]-phenyl-methanone (18a)

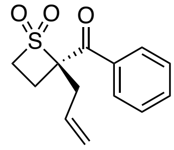

18a

A vial was charged with substrate **16a** (50 mg, 0.17 mmol), $Pd_2(dba)_3$ (3.5 mg, 0.004 mmol), (*S*,*S*)-ANDEN Trost ligand **L4** (8.9 mg, 0.011 mmol) and 1,4-dioxane (4 mL). The reaction mixture was stirred at room temperature for 18 h. The mixture was concentrated under reduced pressure. Purification by flash chromatography [SiO_2; 6:1 hexane:EtOAc] afforded **18a** (33 mg, 78%) as a colourless oil. $\boldsymbol{R}_f$ = 0.59 [2:1 petrol: EtOAc].

^{1}H NMR: (400 MHz, C_6H_6) δ 8.14 – 8.09 (2H, m), 7.02 – 6.95 (3H, m), 5.19 (1H, ddt, *J* = 17.2, 9.6, 7.2 Hz), 4.76 – 4.72 (1H, m), 4.61 (1H, dq, *J* = 16.8, 1.6 Hz), 3.13 – 3.03 (2H, m), 3.02 – 2.96 (1H, m), 2.75 – 2.67 (2H, m), 1.30 (1H, ddd, *J* = 12.4, 10.0, 5.2 Hz)

^{13}C NMR: (100 MHz, C_6H_6) δ 192.4, 134.7, 133.6, 129.7, 129.6, 128.9, 120.3, 92.5, 59.7, 39.7, 15.1.

HRMS: (APCI-TOF) m/z: $[M+H]^+$ calcd for $C_{13}H_{15}O_3S$ 251.0742; found 251.0736.

IR: ν_{max} (neat/cm^{-1}): 3065, 2974, 1675, 1313, 1127.

Chiral HPLC: (OD-H, hexane/*i*-PrOH = 95/5, flow rate = 1.0 mL/min, l = 224 nm) t_R = 11.6 min (minor), 14.1 min (major). 83% ee.

$[\alpha]_D^{20}$ = +27.5 (*c* = 0.20, $CHCl_3$).

(2*S*)-[2-Allyl-1,1-dioxo-thietan-2-yl]-(*o*-tolyl)methanone (18b)

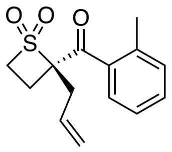

18b

A vial was charged with substrate **16b** (50 mg, 0.16 mmol), $Pd_2(dba)_3$ (3.6 mg, 0.004 mmol), (*S*,*S*)-ANDEN Trost ligand **L4** (8.1 mg, 0.010 mmol) and 1,4-dioxane (4 mL). The reaction mixture was stirred at room temperature for 18 h. The mixture was concentrated under reduced pressure. Purification by flash chromatography [SiO_2; 6:1 hexane:EtOAc] afforded **18b** (36 mg, 83%) as a colourless solid. $\boldsymbol{R_f}$ = 0.70 [3:2 petrol:EtOAc]. **mp**: 52 – 53 ºC.

^{1}H NMR: (400 MHz, $CDCl_3$) δ 7.67 (1H, dd, *J* = 7.8, 1.2 Hz), 7.42 (1H, td, *J* = 7.5, 1.4 Hz), 7.36 – 7.27 (2H, m), 5.51 – 5.41 (1H, m), 5.11 (1H, dq, *J* = 10.1, 1.3 Hz), 4.95 (1H, dq, *J* = 16.9, 1.4 Hz), 4.12 – 4.00 (2H, m), 3.12 – 3.01 (2H, m), 2.94 (1H, ddt, *J* = 14.5, 7.2, 1.2 Hz), 2.44 (3H, s), 2.03 (1H, ddd, *J* = 15.8, 10.3, 5.6 Hz).

^{13}C NMR: (100 MHz, $CDCl_3$) δ 194.6, 140.0, 134.0, 132.5, 132.1, 129.3, 128.5, 125.9, 121.0, 93.8, 60.8, 39.2, 21.4, 15.4.

HRMS: (APCI-TOF) m/z: $[M+H]^+$ calcd for $C_{14}H_{17}O_3S$ 265.0893; found 265.0888.

IR: ν_{max} (neat/cm^{-1}): 3039, 2972, 1679, 1332, 1138.

Chiral HPLC: (OD-H, hexane/*i*-PrOH = 95/5, flow rate = 1.0 mL/min, l = 224 nm) t_R = 12.6 min (minor), 16.9 min (major). 84% ee.

$[\alpha]_D^{20}$ = +182.4 (*c* = 0.26, $CHCl_3$).

(2*S*)-[2-Allyl-1,1-dioxo-thietan-2-yl]-(*p*-tolyl)methanone (18c)

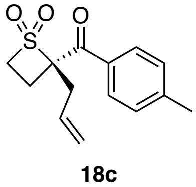

18c

A vial was charged with substrate **16c** (50 mg, 0.16 mmol), $Pd_2(dba)_3$ (3.6 mg, 0.004 mmol), (*S*,*S*)-ANDEN Trost ligand **L4** (8.1 mg, 0.010 mmol) and 1,4-dioxane (4 mL). The reaction mixture was stirred at room temperature for 18 h. The mixture was concentrated under reduced pressure. Purification by flash chromatography [SiO_2; 6:1 hexane:EtOAc] afforded **18c** (35 mg, 83%) as a colourless oil. $\boldsymbol{R_f}$ = 0.70 [3:2 petrol:EtOAc].

^{1}H NMR: (400 MHz, $CDCl_3$) δ 7.92 – 7.88 (2H, m), 7.31 (2H, dd, *J* = 8.6, 0.5 Hz), 5.53 – 5.42 (1H, m), 5.12 (1H, dq, *J* = 10.1, 1.4 Hz), 5.05 (1H, dq, *J* = 16.9, 1.4 Hz), 4.07 (1H, ddd, *J* = 12.9, 10.5, 8.9 Hz), 3.97 (1H, ddd, *J* = 12.9, 10.7, 4.6 Hz), 3.17 (1H, ddq, *J* = 14.4, 6.6, 1.2 Hz), 3.06 – 2.97 (2H, m), 2.43 (3H, s), 2.07 (1H, ddd, *J* = 12.5, 10.6, 4.6 Hz).

^{13}C NMR: (100 MHz, $CDCl_3$) δ 191.5, 132.8, 132.4, 130.8, 129.5, 128.9, 121.4, 92.7, 60.1, 39.9, 21.9, 15.4.

HRMS: (APCI-TOF) m/z: $[M+H]^+$ calcd for $C_{14}H_{17}O_3S$ 265.0893; found 265.0892.

IR: ν_{max} (neat/cm^{-1}): 3030, 2976, 1671, 1313, 1123.

Chiral HPLC: (OD-H, hexane/*i*-PrOH = 95/5, flow rate = 1.0 mL/min, l = 224 nm) t_R = 16.8 min (minor), 22.2 min (major). 86% ee.

$[\alpha]_D^{20}$ = +52.3 (*c* = 0.22, $CHCl_3$).

(2*S*)-[2-Allyl-1,1-dioxo-thietan-2-yl]-(4-methoxyphenyl)methanone (18d)

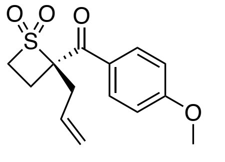

18d

A vial was charged with substrate **16d** (40 mg, 0.12 mmol), $Pd_2(dba)_3$ (3.0 mg, 0.003 mmol), (*S*,*S*)-ANDEN Trost ligand **L4** (6.4 mg, 0.0078 mmol) and 1,4-dioxane (3 mL). The reaction mixture was stirred at room temperature for 18 h. The mixture was concentrated under reduced pressure. Purification by flash chromatography [SiO_2; 10:1 hexane:EtOAc] afforded **18d** (20 mg, 60%) as a colourless oil. $\boldsymbol{R_f}$ = 0.41 [2:1 petrol:EtOAc].

^{1}H NMR: (400 MHz, $CDCl_3$) δ 7.97 (2H, d, *J* = 8.8 Hz), 6.99 (2H, d, *J* = 9.2 Hz), 5.55 – 5.43 (1H, m), 5.13 (1H, dq, *J* = 10.1, 0.9 Hz), 5.07 (1H, dq, *J* = 16.9, 1.4 Hz), 4.06 (1H, ddd, *J* = 12.9, 10.5, 9.1 Hz), 3.94 (1H, ddd, *J* = 12.9, 10.7, 4.4 Hz), 3.89 (3H, s), 3.16 (1H, ddq, *J* = 14.4, 6.6, 1.2 Hz), 3.06 – 2.96 (2H, m), 2.07 (1H, ddd, *J* = 12.5, 10.5, 4.4 Hz).

^{13}C NMR: (100 MHz, $CDCl_3$) δ 190.5, 164.4, 131.9, 129.4, 126.9, 121.1, 114.3, 92.6, 60.0, 55.7, 40.1, 15.5.

HRMS: (APCI-TOF) m/z: $[M+H]^+$ calcd for $C_{14}H_{17}O_4S$ 281.0842; found 281.0828.

IR: ν_{max} (neat/cm^{-1}): 3032, 2965, 1680, 1318, 1130.

Chiral HPLC: (OD-H, hexane/*i*-PrOH = 95/5, flow rate = 1.0 mL/min, l = 224 nm) t_R = 29.0 min (minor), 37.2 min (major). 84% ee.

$[\alpha]_D^{20}$ = +69.4 (*c* = 0.12, $CHCl_3$).

(2*S*)-[2-Allyl-1,1-dioxo-thietan-2-yl]-(4-fluorophenyl)methanone (18e)

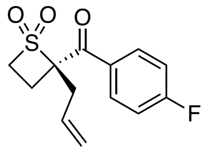

18e

A vial was charged with substrate **16e** (25 mg, 0.08 mmol), $Pd_2(dba)_3$ (1.8 mg, 0.002 mmol), (*S*,*S*)-ANDEN Trost ligand **L4** (4.2 mg, 0.005 mmol) and 1,4-dioxane (2 mL). The reaction mixture was stirred at room temperature for 6 h. The mixture was concentrated under reduced pressure. Purification by flash chromatography [SiO_2; 9:1 hexane:EtOAc] afforded **18e** (19 mg, 89%) as a colourless oil. R_f = 0.44 [2:1 petrol:EtOAc].

^{1}H NMR: (400 MHz, $CDCl_3$) δ 8.04 – 7.98 (2H, m), 7.22 – 7.16 (2H, m), 5.52 – 5.41 (1H, m), 5.13 (1H, m), 5.04 (1H, dq, *J* = 16.8, 1.6 Hz), 4.09 (1H, ddd, *J* = 13.2, 10.8, 8.8 Hz), 3.98 (1H, ddd, *J* = 12.8, 10.4, 4.4 Hz), 3.16 – 3.08 (1H, m), 3.07 – 2.97 (2H, m), 2.09 (1H, ddd, *J* = 12.4, 10.4, 4.4 Hz).

^{13}C NMR: (100 MHz, $CDCl_3$) δ 190.5, 166.1 (d, *J* = 255.6 Hz), 132.1 (d, *J* = 9.5 Hz), 130.4, 128.9, 121.3, 116.2 (d, *J* = 21.9 Hz), 92.4, 60.1, 39.6, 15.3.

^{19}**F{^{1}H} NMR**: (376 MHz, $CDCl_3$) δ –103.0.

HRMS: (APCI-TOF) m/z: $[M+H]^+$ calcd for $C_{13}H_{14}O_3FS$ 269.0642; found 269.0630.

IR: ν_{max} (neat/cm^{-1}): 3030, 2976, 1677, 1315, 1159.

Chiral HPLC: (AD-H, hexane/*i*-PrOH = 90/10, flow rate = 1.0 mL/min, l = 254 nm) t_R = 20.6 min (minor), 21.3 min (major). 86% ee.

$[\alpha]_D^{22}$ = +62.5 (*c* = 0.12, $CHCl_3$).

(2*S*)-[2-Allyl-1,1-dioxo-thietan-2-yl]-(4-bromophenyl)methanone (18f)

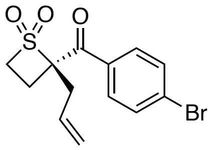

18f

A vial was charged with substrate **16f** (50 mg, 0.13 mmol), $Pd_2(dba)_3$ (2.8 mg, 0.003 mmol), (*S*,*S*)-ANDEN Trost ligand **L4** (7.3 mg, 0.009 mmol) and 1,4-dioxane (4 mL). The reaction mixture was stirred at room temperature for 18 h. The mixture was concentrated under reduced pressure. Purification by flash chromatography [SiO_2; 6:1 hexane:EtOAc] afforded **18f** (42 mg, 95%) as a colourless solid. $\boldsymbol{R_f}$ = 0.35 [3:1 petrol:EtOAc]. **mp**: 71 – 73 ºC.

^{1}**H NMR**: (400 MHz, $CDCl_3$) δ 7.83 (2H, d, *J* = 8.8 Hz), 7.66 (2H, d, *J* = 8.4 Hz), 5.53 – 5.40 (1H, m), 5.13 (1H, dq, *J* = 10.1, 1.3 Hz), 5.04 (1H, dq, *J* = 16.9, 1.4 Hz), 4.09 (1H, ddd, *J* = 13.0, 10.6, 8.8 Hz), 3.98 (1H, ddd, *J* = 13.0, 10.7, 4.7 Hz), 3.11 (1H, ddq, *J* = 14.5, 6.8, 1.1 Hz), 3.06 – 2.97 (2H, m), 2.08 (1H, ddd, *J* = 15.2, 10.6, 4.7 Hz).

^{13}**C NMR**: (100 MHz, $CDCl_3$) δ 191.5, 132.8, 132.4, 130.8, 129.5, 128.9, 121.4, 92.6, 60.3, 39.6, 15.3.

HRMS: (APCI-TOF) m/z: $[M+H]^+$ calcd for $C_{13}H_{14}O_3S^{79}Br$ 328.9842; found 328.9850.

IR: ν_{max} (neat/cm^{-1}): 3032, 2965, 1675, 1313, 1127.

Chiral HPLC: (OD-H, hexane/*i*-PrOH = 95/5, flow rate = 1.0 mL/min, l = 224 nm) t_R = 16.6 min (minor), 18.1 (major). 85% ee.

$[\alpha]_D^{20}$ = +31.8 (c = 0.29, $CHCl_3$).

(2*S*)-[2-Allyl-1,1-dioxo-thietan-2-yl]-(4-methylbenzoate)methanone (18g)

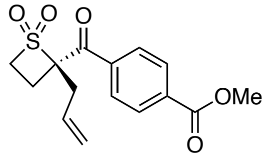

18g

A vial was charged with substrate **16g** (15 mg, 0.04 mmol), $Pd_2(dba)_3$ (1.0 mg, 0.001 mmol), (*S*,*S*)-ANDEN Trost ligand **L4** (3.0 mg, 0.003 mmol) and 1,4-dioxane (1 mL). The reaction mixture was stirred at room temperature for 18 h. The mixture was concentrated under reduced pressure. Purification by flash chromatography [SiO_2; 5:1 – 2:1 heptane:EtOAc] afforded **18g** (12 mg, 92%) as a yellow solid. R_f = 0.23 [2:1 petrol:EtOAc]. **mp**: 78 – 80 ºC.

^{1}H NMR: (400 MHz, $CDCl_3$) δ 8.17 (2H, dt, *J* = 8.7, 2.0 Hz), 8.00 (2H, dt, *J* = 8.8, 2.0 Hz), 5.45 (1H, ddt, *J* = 17.0, 9.8, 7.3 Hz), 5.11 (1H, dd, *J* = 10.1, 1.2 Hz), 4.99 (1H, dq, *J* = 16.9, 1.4 Hz), 4.11 (1H, ddd, *J* = 13.0, 10.5, 8.6 Hz), 4.01 (1H, ddd, *J* = 13.0, 10.7, 4.9 Hz), 3.95 (3H, s) 3.16 – 3.08 (1H, m), 3.07 – 2.97 (2H, m), 2.09 (1H, ddd, *J* = 12.4, 10.5, 4.8 Hz).

^{13}C NMR: (100 MHz, $CDCl_3$) δ 192.2, 166.1, 137.5, 134.6, 130.1, 129.3, 128.8, 121.4, 92.7, 60.5, 52.7, 39.4, 15.3.

HRMS: (APCI-TOF) m/z: $[M+H]^+$ calcd for $C_{15}H_{17}O_5S$ 309.0791; found 309.0786.

IR: ν_{max} (neat/cm^{-1}): 2953, 1720, 1681, 1317, 1108.

Chiral HPLC: (OD-H, hexane/*i*-PrOH = 95/5, flow rate = 1.0 mL/min, l = 254 nm) t_R = 25.7 min (minor), 33.9 min (major). 79% ee.

$[\alpha]_D^{23}$ = +33.3 (c = 0.06, $CHCl_3$).

(2*S*)-[2-Allyl-1,1-dioxo-thietan-2-yl]-(2-furyl)methanone (18h)

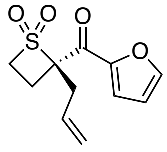

18h

A vial was charged with substrate **16h** (25 mg, 0.09 mmol), $Pd_2(dba)_3$ (2.0 mg, 0.002 mmol), (*S*,*S*)-ANDEN Trost ligand **L4** (4.6 mg, 0.006 mmol) and 1,4-dioxane (2 mL). The reaction mixture was stirred at room temperature for 6 h. The mixture was concentrated under reduced pressure. Purification by flash chromatography [SiO_2; 6:1 hexane:EtOAc] afforded **18h** (15 mg, 69%) as a colourless oil. R_f = 0.59 [3:2 petrol:EtOAc].

^{1}H NMR: (400 MHz, $CDCl_3$) δ 7.70 (1H, dd, *J* = 1.7, 0.7 Hz), 7.40 (1H, dd, *J* = 3.6, 0.7 Hz), 6.61 (1H, dd, *J* = 3.7, 1.7 Hz), 5.61 – 5.50 (1H, m), 5.18 – 5.09 (2H, m), 4.05 (1H, ddd, *J* = 13.0, 10.6, 8.5 Hz), 3.95 (1H, ddd, *J* = 13.0, 10.8, 4.9 Hz), 3.33 (1H, ddq, *J* = 14.5, 6.8, 0.9 Hz), 3.03 (1H, ddt, *J* = 14.5, 7.6, 1.0 Hz), 2.99 – 2.90 (1H, m), 2.05 (1H, ddd, *J* = 12.5, 10.6, 4.9 Hz).

^{13}C NMR: (100 MHz, $CDCl_3$) δ 180.7, 151.0, 147.7, 129.5, 121.1, 120.0, 113.1, 92.7, 61.1, 38.7, 14.9.

HRMS: (APCI-TOF) m/z: $[M+H]^+$ calcd for $C_{11}H_{13}O_4S$ 241.0529; found 241.0518.

IR: ν_{max} (neat/cm^{-1}): 3134, 2968, 1662, 1313, 1127.

Chiral HPLC: (AD-H, hexane/*i*-PrOH = 90/10, flow rate = 1.0 mL/min, l = 224 nm) t_R = 12.5 min (minor), 13.1 min (major). 81% ee.

$[\alpha]_D^{20}$ = –105.7 (*c* = 0.03, $CHCl_3$).

(2-Allyl-1,1-dioxo-thietan-2-yl)-(3-pyridyl)methanone (18i)

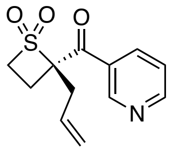

18i

A vial was charged with substrate **16i** (30 mg, 0.10 mmol), $Pd_2(dba)_3$ (2.3 mg, 0.0025 mmol), (*S*,*S*)-ANDEN Trost ligand **L4** (5.3 mg, 0.0065 mmol) and 1,4-dioxane (3 mL). The reaction mixture was stirred at room temperature for 18 h. The mixture was concentrated under reduced pressure. Purification by flash chromatography [SiO_2; 10:1 hexane:EtOAc] afforded **18i** (20 mg, 80%) as a yellow oil. R_f = 0.13 [3:1 petrol:EtOAc].

^{1}H NMR: (400 MHz, $CDCl_3$) δ 9.17 (1H, s), 8.84 (1H, s), 8.24 (1H, dd, *J* = 8.0, 1.9 Hz), 7.48 (1H, dd, *J* = 7.9, 4.9 Hz), 5.52 – 5.41 (1H, m), 5.14 (1H, dd, *J* = 10.1, 1.0 Hz), 5.02 (1H, dd, *J* = 16.9, 1.3 Hz), 4.12 (1H, ddd, *J* = 13.2, 10.4, 8.4 Hz), 4.03 (1H, ddd, *J* = 12.8, 10.8, 5.2 Hz), 3.13 – 3.02 (3H, m), 2.10 (1H, ddd, *J* = 15.7, 10.6, 5.2 Hz).

^{13}C NMR: (100 MHz, $CDCl_3$) δ 191.7, 154.0, 150.6, 136.6, 128.6, 123.8, 121.6, 92.7, 60.7, 39.3, 15.1.

HRMS: (APCI-TOF) m/z: $[M+H]^+$ calcd for $C_{12}H_{14}NO_3S$ 252.0689; found 252.0680.

IR: ν_{max} (neat/cm^{-1}): 3034, 2970, 1736, 1319, 1131.

Chiral HPLC: (OD-H, hexane/*i*-PrOH = 95/5, flow rate = 1.0 mL/min, l = 214 nm) t_R = 40.5 min (minor), 49.8 min (major). 72% ee.

$[\alpha]_D^{21}$ = +36.7 (*c* = 0.15, $CHCl_3$).

(2*S*)-[2-(2-Methylallyl)-1,1-dioxo-thietan-2-yl]-phenyl-methanone (18j)

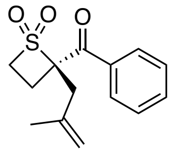

18j

A vial was charged with substrate **16j** (20 mg, 0.07 mmol), $Pd_2(dba)_3$ (2.9 mg, 0.003 mmol), (*S*,*S*)-ANDEN Trost ligand **L4** (6.5 mg, 0.008 mmol) and 1,4-dioxane (2 mL). The reaction mixture was stirred at room temperature for 4 h. The mixture was concentrated under reduced pressure. Purification by flash chromatography [SiO_2; 5:1 hexane:EtOAc] afforded **18j** (12 mg, 70%) as a colourless oil. R_f = 0.56 [2:1 petrol:EtOAc].

^{1}H NMR: (400 MHz, $CDCl_3$) δ 7.94 – 7.90 (2H, m), 7.60 (1H, tt, *J* = 7.4, 1.3 Hz), 7.53 – 7.48 (2H, m), 4.79 (1H, t, *J* = 1.4 Hz), 4.57 (1H, t, *J* = 1.0 Hz), 4.12 (1H, ddd, *J* = 13.0, 10.5, 8.0 Hz), 4.05 (1H, ddd, *J* = 13.0, 10.7, 5.7 Hz), 3.25 (1H, d, *J* = 15.2 Hz), 3.21 (1H, m), 3.09 (1H, d, *J* = 15.2 Hz), 2.17 (1H, ddd, *J* = 12.3, 10.5, 5.6 Hz), 1.49 (3H, s).

^{13}C NMR: (100 MHz, $CDCl_3$) δ 193.1, 138.4, 134.5, 133.6, 129.3, 128.8, 116.2, 92.9, 60.8, 42.5, 23.1, 15.1.

HRMS: (APCI-TOF) m/z: $[M+H]^+$ calcd for $C_{14}H_{17}O_3S$ 265.0893; found 265.0885.

IR: ν_{max} (neat/cm^{-1}): 3026, 2972, 2922, 1675, 1317, 1131.

Chiral HPLC: (OD-H, hexane/*i*-PrOH = 95/5, flow rate = 1.0 mL/min, l = 224 nm) t_R = 13.8 min (minor), 16.9 min (major). 55% ee.

$[\alpha]_D^{20}$ = +81.3 (*c* = 0.08, $CHCl_3$).

(2*S*)-[2-(2-Methylallyl)-1,1-dioxo-thietan-2-yl]-(*p*-tolyl)-methanone (18k)

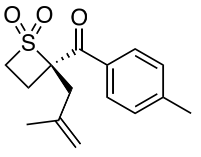

18k

A vial was charged with substrate **16k** (10 mg, 0.03 mmol), $Pd_2(dba)_3$ (1.4 mg, 0.002 mmol), (*S*,*S*)-ANDEN Trost ligand **L4** (3.3 mg, 0.004 mmol) and 1,4-dioxane (1 mL). The reaction mixture was stirred at room temperature for 4 h. The mixture was concentrated under reduced pressure. Purification by flash chromatography [SiO_2; 9:1 hexane:EtOAc] afforded **18k** (6 mg, 70%) as a yellow solid. ***R*f** = 0.59 [2:1 petrol:EtOAc]. **mp**: 67 – 68 ºC.

^{1}H NMR: (400 MHz, $CDCl_3$) δ 7.84 (2H, d, *J* = 8.3 Hz), 7.30 (2H, d, *J* = 8.0 Hz), 4.80 (1H, t, *J* = 1.4 Hz), 4.59 (1H, br s), 4.10 (1H, ddd, *J* = 13.0, 10.4, 8.1 Hz), 4.02 (1H, ddd, *J* = 12.9, 10.5, 5.6 Hz), 3.26 (1H, d, *J* = 15.2 Hz), 3.20 (1H, m), 3.08 (1H, d, *J* = 15.2 Hz), 2.42 (3H, s), 2.16 (1H, ddd, *J* = 12.4, 10.4, 5.6 Hz), 1.49 (3H, s).

^{13}C NMR: (100 MHz, $CDCl_3$) δ 192.5, 144.7, 138.5, 131.9, 129.5, 129.5, 116.2, 92.9, 60.7, 43.3, 23.1, 21.7, 15.1.

HRMS: (APCI-TOF) m/z: $[M+H]^+$ calcd for $C_{15}H_{19}O_3S$ 279.1049; found 279.1043.

IR: ν_{max} (neat/cm^{-1}): 3032, 2970, 2924, 1671, 1315, 1131.

Chiral HPLC: (OD-H, hexane/*i*-PrOH = 97/3, flow rate = 1.0 mL/min, l = 224 nm) t_R = 17.7 min (minor), 24.0 min (major). 53% ee.

$[\alpha]_D^{22}$ = +75.0 (*c* = 0.04, $CHCl_3$).

(2*S*)-[2-(2-Methylallyl)-1,1-dioxo-thietan-2-yl]-(4-methoxyphenyl)-methanone (18l)

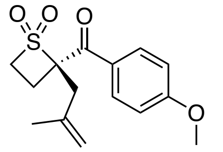

18l

A vial was charged with substrate **16l** (10 mg, 0.03 mmol), $Pd_2(dba)_3$ (1.4 mg, 0.002 mmol), (*S*,*S*)-ANDEN Trost ligand **L4** (3.2 mg, 0.004 mmol) and 1,4-dioxane (1 mL). The reaction mixture was stirred at room temperature for 4 h. The mixture was concentrated under reduced pressure. Purification by flash chromatography [SiO_2; 9:1 hexane:EtOAc] afforded **18l** (6 mg, 68%) as a yellow oil. R_f = 0.50 [2:1 petrol:EtOAc].

^{1}H NMR: (400 MHz, $CDCl_3$) δ 7.93 (2H, d, *J* = 9.0 Hz), 6.98 (2H, d, *J* = 9.0 Hz), 4.81 (1H, t, *J* = 1.4 Hz), 4.61 (1H, br s), 4.09 (1H, ddd, *J* = 13.0, 10.4, 8.3 Hz), 3.99 (1H, ddd, *J* = 12.9, 10.6, 5.4 Hz), 3.88 (3H, s), 3.25 (1H, d, *J* = 12.8 Hz), 3.19 – 3.12 (1H, m), 3.09 (1H, d, *J* = 14.8 Hz), 2.17 (1H, ddd, *J* = 12.4, 10.5, 5.4 Hz), 1.50 (3H, s).

^{13}C NMR: (100 MHz, $CDCl_3$) δ 191.3, 164.1, 138.7, 131.9, 127.4, 116.5, 114.2, 92.9, 60.7, 55.7, 43.1, 23.2, 15.3.

HRMS: (APCI-TOF) m/z: $[M+H]^+$ calcd for $C_{15}H_{19}O_4S$ 295.0999; found 295.0998.

IR: ν_{max} (neat/cm^{-1}): 3076, 2970, 2842, 1310, 1129.

Chiral HPLC: (OD-H, hexane/*i*-PrOH = 97/3, flow rate = 1.0 mL/min, l = 224 nm) t_R = 32.7 min (minor), 43.5 min (major). 52% ee.

$[\alpha]_D^{22}$ = –50.0 (*c* = 0.01, $CHCl_3$).

(2*S*)-[2-[(*E*)-Cinnamyl]-1,1-dioxo-thietan-2-yl]-phenyl-methanone (18m)

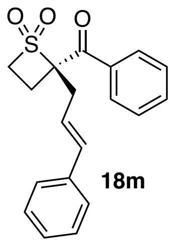

18m

A vial was charged with substrate **16m** (20 mg, 0.05 mmol), $Pd_2(dba)_3$ (2.5 mg, 0.003 mmol), (*S*,*S*)-ANDEN Trost ligand **L4** (5.7 mg, 0.007 mmol) and 1,4-dioxane (1 mL). The reaction mixture was stirred at room temperature for 4 h. The mixture was concentrated under reduced pressure. Purification by flash chromatography [SiO_2; 5:1 hexane:EtOAc] afforded **18m** (10 mg, 57%) as a colourless oil. R_f = 0.50 [2:1 petrol:EtOAc].

^{1}H NMR: (400 MHz, $CDCl_3$) δ 8.02 – 7.98 (2H, m), 7.64 (1H, tt, *J* = 7.3, 1.3 Hz), 7.57 – 7.52 (2H, m), 7.30 – 7.20 (5H, m), 6.33 (1H, d, *J* = 15.8 Hz), 5.86 – 5.78 (1H, m), 4.15 – 4.07 (1H, m), 4.00 (1H, ddd, *J* = 12.9, 10.7, 4.6 Hz), 3.31 (1H, dd, *J* = 14.6, 7.1 Hz), 3.12 (1H, dd, *J* = 14.4, 1.2 Hz), 3.10 – 3.02 (1H, m), 2.15 (1H, ddd, *J* = 12.5, 10.4, 4.6 Hz).

^{13}C NMR: (100 MHz, $CDCl_3$) δ 192.4, 136.2, 136.0, 134.1, 134.0, 129.5, 129.1, 128.7, 128.2, 126.5, 120.1, 93.0, 60.3, 39.0, 15.4.

HRMS: (APCI-TOF) m/z: $[M+H]^+$ calcd for $C_{19}H_{19}O_3S$ 327.1049; found 327.1059.

IR: ν_{max} (neat/cm^{-1}): 3058, 3028, 2965, 1675, 1315, 1131.

Chiral HPLC: (OD-H, hexane/*i*-PrOH = 95/5, flow rate = 1.0 mL/min, l = 250 nm) t_R = 45.8 min (minor), 57.3 min (major). 52% ee.

$[\alpha]_D^{20}$ = –66.7 (*c* = 0.09, $CHCl_3$).

[2-(3-Methylbut-2-enyl)-1,1-dioxo-thietan-2-yl]-phenyl-methanone (18n) and **(1,1-Dioxothietan-2-yl)-phenyl-methanone (32)**

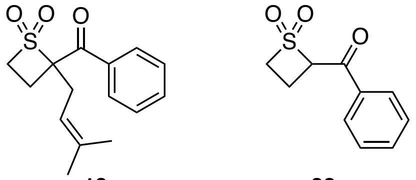

18n **32**

A vial was charged with substrate **16n** (40 mg, 0.12 mmol), $Pd_2(dba)_3$ (5.6 mg, 0.006 mmol) and (*S*,*S*)–ANDEN Trost ligand **L4** (5.7 mg, 0.016 mmol) and 1,4-dioxane (3 mL). The reaction mixture was stirred at room temperature for 4 days. The mixture was concentrated under reduced pressure. Purification by column chromatography [SiO_2; 5:1 hexane:EtOAc] afforded a mixture of starting material **16n** and non-alkylated product **32** in a 1:1.7 ratio, corresponding to starting material **16n** (15 mg, 39%) and non-alkylated product **32** (15 mg, 60%) as a colourless solid.

Instead, **18n** was prepared in racemic form. A vial was charged with substrate **16n** (10 mg, 0.03 mmol), $Pd(PPh_3)_4$ (3.5 mg, 0.003 mmol) and 1,4-dioxane (2 mL). The reaction mixture was stirred at room temperature for 4 h. The mixture was concentrated under reduced pressure and purification by flash column chromatography [SiO_2; 5:1 hexane:EtOAc] afforded **18n** (7 mg, 84%) as a colourless oil. $\boldsymbol{R_f}$ = 0.59 [2:1 petrol:EtOAc].

^{1}H NMR: (400 MHz, $CDCl_3$) δ 7.97 – 7.95 (2H, m), 7.60 (1H, tt, *J* = 7.4, 1.3 Hz), 7.53 – 7.48 (2H, m), 4.82 – 4.76 (1H, m), 4.08 (1H, ddd, *J* = 12.9, 10.5, 8.9 Hz), 3.97 (1H, ddd, *J* = 12.9, 10.7, 4.5 Hz), 3.13 (1H, dd, *J* = 14.8, 7.5 Hz), 3.07 – 2.96 (2H, m), 2.02 (1H, ddd, *J* = 12.4, 10.6, 4.5 Hz), 1.61 (3H, d, *J* = 1.0 Hz), 1.35 (3H, s).

^{13}C NMR: (100 MHz, $CDCl_3$) δ 192.7, 138.2, 134.2, 133.9, 129.3, 128.9, 114.5, 93.4, 60.1, 34.2, 26.0, 17.9, 15.6.

HRMS: (APCI-TOF) m/z: $[M+H]^+$ calcd for $C_{15}H_{19}O_3S$ 279.1049; found 279.1040.

IR: ν_{max} (neat/cm^{-1}): 3026, 2968, 2916, 1675, 1312, 1129.

(32) $\boldsymbol{R_f}$ = 0.28 [2:1 petrol:EtOAc]. **mp**: 70 – 72 ºC.

^{1}H NMR: (400 MHz, $CDCl_3$) δ 8.09 – 8.04 (2H, m), 7.68 (1H, tt, *J* = 7.4, 1.9 Hz), 7.59 – 7.52 (2H, m), 5.99 – 5.92 (1H, m), 4.33 – 4.12 (2H, m), 3.07 – 2.93 (1H, m), 2.38 – 2.24 (1H, m).

^{13}C NMR: (100 MHz, $CDCl_3$) δ 187.1, 135.4, 134.7, 129.2, 128.7, 82.3, 64.2, 7.7.

HRMS: (APCI-TOF) m/z: $[M+H]^+$ calcd for $C_{10}H_{11}O_3S$ 211.0423; found 211.0419.

IR: ν_{max} (neat/cm^{-1}): 3065, 3047, 2968, 1677.

(2*S*)-1-Adamantyl-[2-Allyl-1,1-dioxo-thietan-2-yl]methanone (18o)

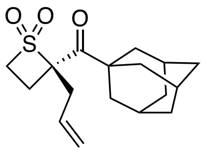

18o

A vial was charged with substrate **16o** (30 mg, 0.08 mmol), $Pd_2(dba)_3$ (2.3 mg, 0.004 mmol), (*S*,*S*)-ANDEN Trost ligand **L4** (4.9 mg, 0.010 mmol) and 1,4-dioxane (2 mL). The reaction mixture was stirred at room temperature for 18 h. The mixture was concentrated under reduced pressure. Purification by flash chromatography [SiO_2; 6:1 hexane:EtOAc] afforded **18o** (12 mg, 45%) as a colourless solid. $\boldsymbol{R_f}$ = 0.67 [2:1 petrol:EtOAc]. **mp**: 136 – 137 ºC.

^{1}H NMR: (400 MHz, $CDCl_3$) δ 5.59 – 5.48 (1H, m), 5.25 (1H, br s), 5.22 (1H, dq, *J* = 6.3, 1.2 Hz), 3.89 (1H, dt, *J* = 12.8, 10.3 Hz), 3.76 (1H, ddd, *J* = 12.9, 10.5, 3.1 Hz), 3.17 (1H, ddq, *J* = 15.0, 6.1, 1.3 Hz), 2.90 (1H, ddt, *J* = 15.0, 7.8, 1.0 Hz), 2.77 – 2.68 (1H, m), 2.09 – 1.97 (10H, m), 1.74 (6H, br s).

^{13}C NMR: (100 MHz, $CDCl_3$) δ 207.9, 130.2, 121.3, 94.9, 58.9, 47.0, 38.4, 37.9, 36.5, 28.1, 16.5.

HRMS: (APCI-TOF) m/z: $[M+H]^+$ calcd for $C_{17}H_{25}O_3S$ 309.1519; found 309.1510.

IR: ν_{max} (neat/cm^{-1}): 3013, 2905, 1677, 1315, 1131.

Chiral HPLC: (OD-H, hexane/*i*-PrOH = 95/5, flow rate = 1.0 mL/min, l = 214 nm) t_R = 8.9 min (minor), 10.0 min (major). 93% ee.

$[\alpha]_D^{20}$ = –41.7 (*c* = 0.06, $CHCl_3$).

(2*S*)-1-[2-Allyl-1,1-dioxo-thietan-2-yl]-2,2-dimethyl-propan-1-one (18p)

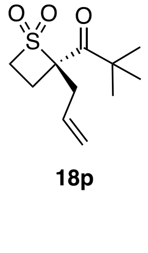

18p

A vial was charged with substrate **16p** (50 mg, 0.18 mmol), $Pd_2(dba)_3$ (3.6 mg, 0.004 mmol), (*S*,*S*)-ANDEN Trost ligand **L4** (10.0 mg, 0.012 mmol) and 1,4-dioxane (4 mL). The reaction mixture was stirred at room temperature for 18 h. The mixture was concentrated under reduced pressure. Purification by flash chromatography [SiO_2; 6:1 hexane:EtOAc] afforded **18p** (37 mg, 90%) as a colourless oil. R_f = 0.34 [2:1 petrol: EtOAc].

^{1}H NMR: (400 MHz, $CDCl_3$) δ 5.60 – 5.49 (1H, m), 5.26 – 5.24 (1H, m), 5.22 (1H, dq, *J* = 7.2, 1.4 Hz), 3.95 – 3.86 (1H, m), 3.79 (1H, ddd, *J* = 14.0, 10.6, 3.4 Hz), 3.12 (1H, ddq, *J* = 15.0, 6.3, 1.3 Hz), 2.91 (1H, ddt, *J* = 15.0, 7.6, 1.2 Hz), 2.80 – 2.70 (1H, m), 2.00 (1H, ddd, *J* = 13.8, 10.4, 3.4 Hz), 1.31 (9H, s).

^{13}C NMR: (100 MHz, $CDCl_3$) δ 208.6, 130.0, 121.3, 95.0, 59.2, 44.3, 37.7, 27.7, 16.5.

HRMS: (APCI-TOF) m/z: $[M+H]^+$ calcd for $C_{11}H_{19}O_3S$ 231.1049; found 231.1044.

IR: ν_{max} (neat/cm^{-1}): 3084, 2974, 1725, 1317, 1127.

Chiral HPLC: (OD-H, hexane/*i*-PrOH = 95/5, flow rate = 1.0 mL/min, l = 220 nm) t_R = 9.7 min (minor), 10.3 min (major). 90% ee.

$[\alpha]_D^{20}$ = –83.3 (*c* = 0.21, $CHCl_3$).

(2*S*)-[2-Allyl-1,1-dioxo-thietan-2-yl]-cyclohexyl-methanone (18q)

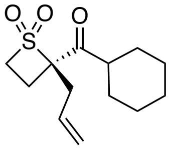

18q

A vial was charged with substrate **16q** (50 mg, 0.17 mmol), $Pd_2(dba)_3$ (3.7 mg, 0.004 mmol), (*S*,*S*)-ANDEN Trost ligand **L4** (9.0 mg, 0.011 mmol) and 1,4-dioxane (4 mL). The reaction mixture was stirred at room temperature for 18 h. The mixture was concentrated under reduced pressure. Purification by flash chromatography [SiO_2; 6:1 hexane:EtOAc] afforded **18q** (37 mg, 86%) as a colourless oil. R_f = 0.63 [3:1 petrol:EtOAc].

^{1}H NMR: (400 MHz, $CDCl_3$) δ 5.58 – 5.47 (1H, m), 5.21 (1H, dq, *J* = 5.6, 1.2 Hz), 5.18 (1H, dq, *J* = 12.1, 1.2 Hz), 3.97 – 3.85 (2H, m), 3.10 – 3.03 (1H, m), 2.96 (1H, ddt, *J* = 15.2, 7.7,

1.1 Hz), 2.82 – 2.74 (1H, m), 2.68 (1H, tt, *J* = 11.3, 3.0 Hz), 1.87 (1H, ddd, *J* = 16.6, 10.0, 6.6 Hz), 2.05 – 1.97 (1H, m), 1.82 – 1.74 (3H, m), 1.72 – 1.64 (1H, m), 1.43 – 1.19 (5H, m).

^{13}C NMR: (100 MHz, $CDCl_3$) δ 205.5, 129.8, 120.9, 95.2, 61.5, 48.9, 36.6, 30.1, 28.4, 25.7, 14.6.

HRMS: (APCI-TOF) m/z: $[M+H]^+$ calcd for $C_{13}H_{21}O_3S$ 257.1206; found 257.1206.

IR: ν_{max} (neat/cm^{-1}): 2929, 2853, 1677, 1306, 1127.

Chiral HPLC: (OD-H, hexane/*i*-PrOH = 95/5, flow rate = 1.0 mL/min, l = 214 nm) t_R = 7.8 min (minor), 8.9 min (major). 94% ee.

$[\alpha]_D^{20}$ = –88.5 (*c* = 0.21, $CHCl_3$).

(2*S*)-1-[2-Allyl-1,1-dioxo-thietan-2-yl]-2-methyl-propan-1-one (18r)

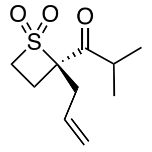

18r

A vial was charged with substrate **16r** (50 mg, 0.19 mmol), $Pd_2(dba)_3$ (4.4 mg, 0.0048 mmol), (*S*,*S*)-ANDEN Trost ligand **L4** (10.0 mg, 0.012 mmol) and 1,4-dioxane (4 mL). The reaction mixture was stirred at room temperature for 18 h. The mixture was concentrated under reduced pressure. Purification by flash chromatography [SiO_2; 6:1 hexane:EtOAc] afforded **18r** (38 mg, 93%) as a colourless oil. R_f = 0.28 [3:1 petrol:EtOAc].

^{1}H NMR: (400 MHz, $CDCl_3$) δ 5.60 – 5.49 (1H, m), 5.24 – 5.21 (1H, m), 5.19 (1H, dq, *J* = 11.1, 1.2 Hz), 3.99 – 3.87 (2H, m), 3.12 – 3.05 (1H, m), 3.02 – 2.92 (2H, m), 2.85 – 2.77 (1H, m), 1.90 (1H, ddd, *J* = 12.3, 10.1, 6.5 Hz), 1.21 (3H, d, *J* = 6.7 Hz), 1.15 (3H, d, *J* = 6.7 Hz).

^{13}C NMR: (100 MHz, $CDCl_3$) δ 206.8, 129.7, 121.0, 95.2, 61.5, 38.7, 36.6, 20.0, 19.0, 14.8.

HRMS: (APCI-TOF) m/z: $[M+H]^+$ calcd for $C_{10}H_{17}O_3S$ 217.0893; found 217.0885.

IR: ν_{max} (neat/cm^{-1}): 3097, 2980, 1716, 1302, 1123.

Chiral HPLC: (OD-H, hexane/*i*-PrOH = 95/5, flow rate = 1.0 mL/min, l = 214 nm) t_R = 9.9 min (minor), 10.8 min (major). 90% ee.

$[\alpha]_D^{20}$ = –96.9 (*c* = 0.19, $CHCl_3$).

(2*S*)-1-[2-Allyl-1,1-dioxo-thietan-2-yl]-3,3-dimethyl-butan-1-one (18s)

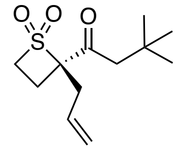

18s

A vial was charged with substrate **16s** (50 mg, 0.17 mmol), $Pd_2(dba)_3$ (3.6 mg, 0.004 mmol), (*S*,*S*)-ANDEN Trost ligand **L4** (9.0 mg, 0.011 mmol) and 1,4-dioxane (4 mL). The reaction mixture was stirred at room temperature for 18 h. The mixture was concentrated under reduced pressure. Purification by flash chromatography [SiO_2; 6:1 hexane:EtOAc] afforded **18s** (31 mg, 76%) as a colourless oil. R_f = 0.67 [2:1 petrol: EtOAc].

^{1}H NMR: (400 MHz, $CDCl_3$) δ 5.60 – 5.49 (1H, m), 5.22 – 5.20 (1H, m), 5.18 (1H, dq, *J* = 7.4. 1.2 Hz), 3.97 – 3.84 (2H, m), 3.05 – 2.98 (1H, m), 2.93 (1H, ddt, *J* = 15.1, 7.0, 1.3 Hz), 2.89 – 2.80 (1H, m), 2.62 (1H, d, *J* = 18.6 Hz), 2.51 (1H, d, *J* = 18.6 Hz), 1.85 (1H, ddd, *J* = 17.0, 9.7, 7.3 Hz), 1.06 (9H, s).

^{13}C NMR: (100 MHz, $CDCl_3$) δ 200.2, 129.4, 120.8, 94.6, 61.6, 52.6, 37.4, 30.7, 29.6, 15.1.

HRMS: (APCI-TOF) m/z: $[M+H]^+$ calcd for $C_{12}H_{21}O_3S$ 245.1206; found 245.1200.

IR: ν_{max} (neat/cm^{-1}): 3084, 2953, 2871, 1714, 1319, 1131.

Chiral HPLC: (OD-H, hexane/*i*-PrOH = 95/5, flow rate = 1.0 mL/min, l = 214 nm) t_R = 6.5 min (minor), 7.7 min (major). 81% ee.

$[\alpha]_D^{20}$= –16.6 (*c* = 0.08, $CHCl_3$).

(2*S*)-1-[2-Allyl-1,1-dioxo-thietan-2-yl]propan-1-one (18t)

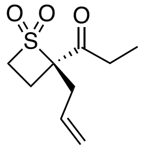

18t

A vial was charged with substrate **16t** (40 mg, 0.16 mmol), $Pd_2(dba)_3$ (3.7 mg, 0.004 mmol), (*S*,*S*)-ANDEN Trost ligand **L4** (8.1 mg, 0.010 mmol) and 1,4-dioxane (4 mL). The reaction mixture was stirred at room temperature for 6 h. The mixture was concentrated under reduced pressure. Purification by flash chromatography [SiO_2; 9:1 hexane:EtOAc] afforded **18t** (25 mg, 78%) as a colourless oil. R_f = 0.50 [2:1 petrol:EtOAc].

^{1}H NMR: (400 MHz, $CDCl_3$) δ 5.58 – 5.48 (1H, m), 5.21 – 5.19 (1H, m), 5.16 (1H, dq, *J* = 5.3, 1.3 Hz), 3.95 (2H, t, *J* = 8.3 Hz), 3.10 – 3.02 (1H, m), 2.94 (1H, dt, *J* = 6.9, 1.3 Hz), 2.93 – 2.85 (1H, m), 2.72 (1H, dq, *J* = 18.3, 7.1 Hz), 2.59 (1H, dq, *J* = 18.3, 7.1 Hz), 1.92 – 1.83 (1H, m), 1.14 (3H, t, *J* = 7.1 Hz).

^{13}C NMR: (100 MHz, $CDCl_3$) δ 202.1, 129.4, 120.7, 94.5, 61.9, 37.1, 34.0, 15.3, 7.7.

HRMS: (APCI-TOF) m/z: $[M+H]^+$ calcd for $C_9H_{15}O_3S$ 203.0736; found 203.0744.

IR: ν_{max} (neat/cm^{-1}): 3082, 2981, 2942, 1712, 1317, 1131.

Chiral HPLC: (OD-H, hexane/*i*-PrOH = 97/3, flow rate = 1.0 mL/min, l = 214 nm) t_R = 13.8 min (minor), 14.7 min (major). 69% ee.

$[\alpha]_D^{22}$ = –25.0 (*c* = 0.14, $CHCl_3$).

(2*S*)-1-[2-Allyl-1,1-dioxo-thietan-2-yl]ethanone (18u)

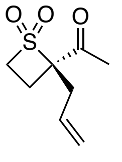

18u

A vial was charged with substrate **16u** (50 mg, 0.21 mmol), $Pd_2(dba)_3$ (5.0 mg, 0.0055 mmol), (*S*,*S*)-ANDEN Trost ligand **L4** (11.6 mg, 0.0143 mmol) and 1,4-dioxane (5 mL). The reaction

mixture was stirred at room temperature for 18 h. The mixture was concentrated under reduced pressure. Purification by flash chromatography [SiO_2; 6:1 hexane:EtOAc] afforded **18u** (26 mg, 66%) as a colourless oil. $\boldsymbol{R}_\mathbf{f}$ = 0.65 [3:2 petrol:EtOAc].

^{1}H NMR: (400 MHz, $CDCl_3$) δ 5.62 – 5.51 (1H, m), 5.23 – 5.18 (2H, m), 3.98 (1H, t, *J* = 8.9 Hz), 3.97 (1H, t, *J* = 13.0 Hz), 3.09 – 3.03 (1H, m), 2.97 (1H, ddt, *J* = 15.0, 6.7, 1.2 Hz), 2.92 – 2.84 (1H, m), 2.35 (3H, s), 1.94 – 1.86 (1H, m).

^{13}C NMR: (100 MHz, $CDCl_3$) δ 199.2, 129.2, 120.8, 94.6, 61.9, 37.1, 28.1, 15.1.

HRMS: (APCI-TOF) m/z: $[M+H]^+$ calcd for $C_8H_{13}O_3S$ 189.0580; found 189.0576.

IR: ν_{max} (neat/cm^{-1}): 3084, 2970, 1710, 1312, 1120.

Chiral HPLC: (OD-H, hexane/*i*-PrOH = 95/5, flow rate = 1.0 mL/min, l = 210 nm) t_R = 13.9 min (minor), 14.6 min (major). 39% ee.

$[\alpha]_D^{20}$ = +21.4 (*c* = 0.07, $CHCl_3$).

(2*S*)-Phenyl 2-allyl-1,1-dioxo-thietane-2-carboxylate (19a)

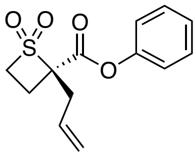

19a

A vial was charged with substrate **17a** (50 mg, 0.16 mmol), $Pd_2(dba)_3$ (3.7 mg, 0.004 mmol), (*S*,*S*)-ANDEN Trost ligand **L4** (9.0 mg, 0.010 mmol) and 1,4-dioxane (4 mL). The reaction mixture was stirred at room temperature for 6 h. The mixture was concentrated under reduced pressure. Purification by flash chromatography [SiO_2; 5:1 hexane:EtOAc] afforded **19a** (38 mg, 90%) as a colourless solid. $\boldsymbol{R}_\mathbf{f}$ = 0.51 [2:1 petrol:EtOAc]. **mp**: 58 – 60 ºC.

^{1}H NMR: (400 MHz, $CDCl_3$) δ 7.43 – 7.37 (2H, m), 7.28 (1H, tt, *J* = 7.4, 1.1 Hz), 7.16 – 7.12 (2H, m), 5.77 (1H, ddt, *J* = 17.1, 10.1, 7.0 Hz), 5.36 – 5.27 (2H, m), 4.22 (1H, ddd, *J* = 13.1, 10.7, 7.1 Hz), 4.12 (1H, ddd, *J* = 13.0, 10.5, 6.2 Hz), 3.31 (1H, dd, *J* = 14.4, 6.8 Hz), 2.95 (1H, ddt, *J* = 14.4, 7.2, 1.1 Hz), 2.92 – 2.84 (1H, m), 2.13 (1H, ddd, *J* = 17.6, 10.5, 7.2 Hz).

^{13}C NMR: (100 MHz, $CDCl_3$) δ 165.8, 150.6, 129.8, 129.6, 126.7, 121.5, 121.0, 89.6, 63.2, 37.2, 16.8.

HRMS: (APCI-TOF) m/z: $[M+H]^+$ calcd for $C_{13}H_{15}O_4S$ 267.0686; found 267.0686.

IR: ν_{max} (neat/cm^{-1}): 3054, 2970, 1751, 1315, 1138.

Chiral HPLC: (OD-H, hexane/*i*-PrOH = 95/5, flow rate = 1.0 mL/min, l = 214 nm) t_R = 18.0 min (major), 21.0 min (minor). 94% ee.

$[\alpha]_D^{20}$ = +25.8 (*c* = 0.31, $CHCl_3$).

(2*S*)-(4-Methoxyphenyl) 2-allyl-1,1-dioxo-thietane-2-carboxylate (19b)

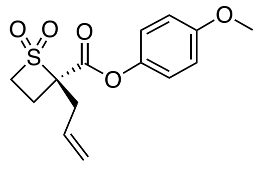

19b

A vial was charged with substrate **17b** (50 mg, 0.15 mmol), $Pd_2(dba)_3$ (3.4 mg, 0.004 mmol), (*S*,*S*)-ANDEN Trost ligand **L4** (7.9 mg, 0.010 mmol) and 1,4-dioxane (4 mL). The reaction mixture was stirred at room temperature for 6 h. The mixture was concentrated under reduced pressure. Purification by flash chromatography [SiO_2; 12:1 hexane:EtOAc] afforded **19b** (20 mg, 45%) as a colourless solid. R_f = 0.49 [3:2 petrol:EtOAc]. **mp**: 50 – 51 °C.

^{1}H NMR: (400 MHz, $CDCl_3$) δ 7.05 (2H, d, *J* = 9.1 Hz), 6.89 (2H, d, *J* = 9.2 Hz), 5.75 (1H, ddt, *J* = 17.0, 10.1, 7.0 Hz), 5.35 – 5.25 (2H, m), 4.21 (1H, ddd, *J* = 13.3, 10.8, 7.2 Hz), 4.10 (1H, ddd, *J* = 13.1, 10.5, 6.2 Hz), 3.80 (3H, s), 3.29 (1H, dd, *J* = 14.4, 6.9 Hz), 2.93 (1H, ddt, *J* = 14.3, 7.2, 1.1 Hz), 2.87 (1H, ddd, *J* = 16.9, 10.8, 6.1 Hz), 2.12 (1H, ddd, *J* = 17.6, 10.4, 7.1 Hz).

^{13}C NMR: (100 MHz, $CDCl_3$) δ 166.1, 157.9, 144.1, 129.7, 122.3, 120.9, 114.7, 89.6, 63.2, 55.8, 37.2, 16.8.

HRMS: (APCI-TOF) m/z: $[M+H]^+$ calcd for $C_{14}H_{17}O_5S$ 297.0791; found 297.0803.

IR: ν_{max} (neat/cm^{-1}): 3006, 2957, 1750, 1325, 1190.

Chiral HPLC: (OD-H, hexane/*i*-PrOH = 90/10, flow rate = 1.0 mL/min, l = 230 nm) t_R = 18.5 min (major), 25.0 min (minor). 93% ee.

$[\alpha]_D^{20}$ = –12.7 (*c* = 0.06, $CHCl_3$).

(2*S*)-*p*-Tolyl 2-allyl-1,1-dioxo-thietane-2-carboxylate (19c)

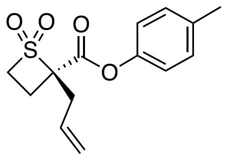

19c

A vial was charged with substrate **17c** (50 mg, 0.15 mmol), $Pd_2(dba)_3$ (3.5 mg, 0.004 mmol), (*S*,*S*)-ANDEN Trost ligand **L4** (7.9 mg, 0.010 mmol) and 1,4-dioxane (4 mL). The reaction mixture was stirred at room temperature for 6 h. The mixture was concentrated under reduced pressure. Purification by flash chromatography [SiO_2; 5:1 hexane:EtOAc] afforded **19c** (38 mg, 90%) as a colourless solid. $\boldsymbol{R_f}$ = 0.54 [3:2 petrol:EtOAc]. **mp**: 83 – 85 ºC.

^{1}H NMR: (400 MHz, $CDCl_3$) δ 7.21 – 7.17 (2H, m), 7.01 (2H, d, *J* = 8.5 Hz), 5.76 (1H, ddt, *J* = 17.1, 10.0, 7.0 Hz), 5.35 – 5.26 (2H, m), 4.21 (1H, ddd, *J* = 13.0, 10.6, 7.1 Hz), 4.10 (1H, ddd, *J* = 13.2, 10.8, 6.4 Hz), 3.30 (1H, dd, *J* = 14.3, 6.8 Hz), 2.94 (1H, ddt, *J* = 14.4, 7.2, 1.0 Hz), 2.87 (1H, ddd, *J* = 17.0, 10.9, 6.2 Hz), 2.35 (3H, s), 2.12 (1H, ddd, *J* = 17.5, 10.5, 7.2 Hz).

^{13}C NMR: (100 MHz, $CDCl_3$) δ 166.0, 148.4, 136.4, 130.2, 129.7, 121.1, 120.9, 89.6, 63.2, 37.2, 21.1, 16.8.

HRMS: (APCI-TOF) m/z: $[M+H]^+$ calcd for $C_{14}H_{17}O_4S$ 281.0842; found 281.0849.

IR: ν_{max} (neat/cm^{-1}): 3034, 2965, 2927, 1746, 1321, 1190.

Chiral HPLC: (OD-H, hexane/*i*-PrOH = 95/5, flow rate = 1.0 mL/min, l = 234 nm) t_R = 16.1 min (major), 24.2 min (minor). 92% ee.

$[\alpha]_D^{20}$ = +13.7 (*c* = 0.31, $CHCl_3$).

(2*S*)-*tert*-Butyl 2-allyl-1,1-dioxo-thietane-2-carboxylate (19d)

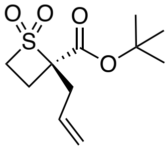

19d

A vial was charged with substrate **17d** (50 mg, 0.17 mmol), $Pd_2(dba)_3$ (7.4 mg, 0.009 mmol), (*S*,*S*)-ANDEN Trost ligand **L4** (17.8 mg, 0.020 mmol) and 1,4-dioxane (4 mL). The reaction mixture was stirred at room temperature for 18 h. The mixture was concentrated under reduced pressure. Purification by flash chromatography [SiO_2; 9:1 hexane:EtOAc] afforded **19d** (25 mg, 60%) as a colourless solid.

Scale Up Procedure for ***19d****.* To $Pd_2(dba)_3$ (798 mg, 0.871 mmol) and (*S*,*S*)-ANDEN Trost ligand **L4** (1.84 g, 2.26 mmol) was added 1,4-dioxane (435 mL) and the mixture was stirred for 30 min. A solution of substrate **17d** (5.05 g, 17.41 mmol) in 1,4-dioxane (10 mL) was added and the mixture was stirred at room temperature overnight. The mixture was concentrated under reduced pressure and purification by flash chromatography [SiO_2; 49:1 – 19:1 – 9:1 hexane:EtOAc] afforded **19d** (3.70 g, 86%) as a colourless solid. $\boldsymbol{R}_f$ = 0.62 [2:1 petrol:EtOAc]. **mp**: 56 – 57 ºC.

^{1}H NMR: (400 MHz, $CDCl_3$) δ 5.68 – 5.57 (1H, m), 5.24 – 5.16 (2H, m), 4.07 (1H, ddd, *J* = 13.0, 10.7, 7.0 Hz), 3.99 (1H, ddd, *J* = 13.0, 10.4, 6.4 Hz), 3.06 (1H, dd, *J* = 14.2, 6.6 Hz), 2.80 – 2.67 (2H, m), 1.95 (1H, ddd, *J* = 17.4, 10.4, 7.0 Hz), 1.52 (9H, s).

^{13}C NMR: (100 MHz, $CDCl_3$) δ 165.4, 130.0, 120.3, 90.1, 84.1, 62.6, 37.4, 28.0, 16.5.

HRMS: (APCI-TOF) m/z: $[M+Na]^+$ calcd for $C_{11}H_{18}O_4SNa$ 269.0818; found 269.0818.

IR: ν_{max} (neat/cm^{-1}): 2980, 2935, 1727, 1321, 1148.

Chiral HPLC: (OD-H, hexane/*i*-PrOH = 95/5, flow rate = 1.0 mL/min, l = 220 nm) t_R = 6.6 min (minor), 7.5 min (major). 96% ee.

$[\alpha]_D^{22}$ = +77.8 (*c* = 0.09, $CHCl_3$).

(2*S*)-Methyl 2-allyl-1,1-dioxo-thietane-2-carboxylate (19e)

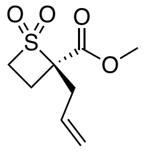

19e

A vial was charged with substrate **17e** (20 mg, 0.08 mmol), $Pd_2(dba)_3$ (3.7 mg, 0.004 mmol), (*S*,*S*)-ANDEN Trost ligand **L4** (8.1 mg, 0.011 mmol) and 1,4-dioxane (2 mL). The reaction mixture was stirred at room temperature for 3 h. The mixture was concentrated under reduced pressure. Purification by flash chromatography [SiO_2; 10:1 hexane:EtOAc] afforded **19e** (14 mg, 85%) as a colourless oil. R_f = 0.45 [2:1 petrol:EtOAc].

^{1}H NMR: (400 MHz, $CDCl_3$) δ 5.68 – 5.56 (1H, m), 5.24 – 5.17 (2H, m), 4.12 (1H, ddd, *J* = 13.0, 10.8, 7.2 Hz), 4.04 (1H, ddd, *J* = 13.0, 10.4, 6.4 Hz), 3.86 (3H, s), 3.10 (1H, dd, *J* = 14.3, 6.5 Hz), 2.84 – 2.72 (2H, m), 2.03 (1H, ddd, *J* = 12.3, 10.3, 7.1 Hz).

^{13}C NMR: (100 MHz, $CDCl_3$) δ 167.1, 129.8, 120.7, 89.6, 62.8, 53.7, 37.1, 16.6.

HRMS: (APCI-TOF) m/z: $[M+H]^+$ calcd for $C_8H_{13}O_4S$ 205.0529; found 205.0520.

IR: ν_{max} (neat/cm^{-1}): 3082, 2970, 1735, 1319, 1127.

Chiral HPLC: (OD-H, hexane/*i*-PrOH = 95/5, flow rate = 1.0 mL/min, l = 214 nm) t_R = 13.3 min (minor), 13.9 min (major). 85% ee.

$[\alpha]_D^{22}$ = +88.9 (*c* = 0.09, $CHCl_3$).

2.5 Synthesis of Spirocycle 12

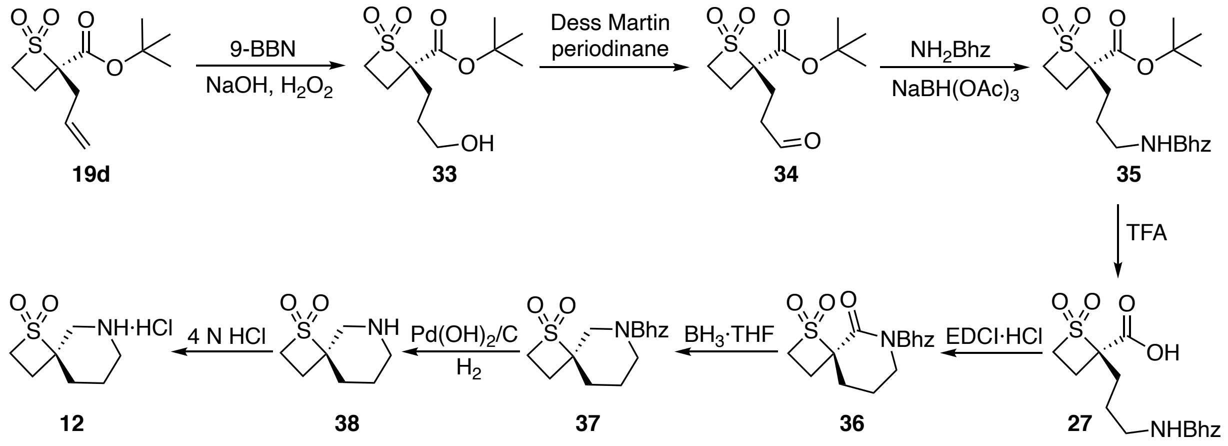

For the purposes of chiral HPLC analysis, **19d** was made in racemic form from **17d** using 10 mol% $Pd(PPh_3)_4$ in 1,4-dioxane and used in the synthesis of *rac*-**12**.

tert-Butyl (2*S*)-2-(3-hydroxypropyl)-1,1-dioxo-thietane-2-carboxylate (33)

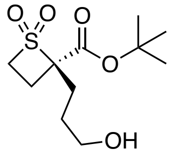

33

9-BBN monomer (0.5 M in THF, 150 mL, 75.0 mmol) was cooled to 0 ºC. A solution of **19d** (3.69 g, 15.0 mmol) in THF (35 mL) was added dropwise and the mixture was stirred at 0 ºC for 30 min, the mixture was allowed to warm to room temperature and stirred at ambient temperature overnight. The mixture was cooled to 0 ºC and a solution of aq. NaOH (3 N, 35 mL) was added dropwise. The mixture was stirred at 0 ºC for 30 min, then H_2O_2 (30% w/w in H_2O, 35 mL) was added dropwise. The mixture was allowed to warm to room temperature and stirred at ambient temperature for 5 h. The reaction was quenched slowly with aq. $Na_2S_2O_3$ (20 mL), diluted with water (500 mL), and extracted with EtOAc (3 x 200 mL). The organic layer was washed with brine (100 mL), dried ($MgSO_4$) and concentrated under reduced pressure. Purification by flash column chromatography [SiO_2; 4:1 – 2:1 hexane:EtOAc] afforded **33** (2.45 g, 62%) as an opaque oil. $\boldsymbol{R}_f$ = 0.51 [EtOAc].

^{1}H NMR: (400 MHz, $CDCl_3$) δ 4.08 (1H, ddd, *J* = 13.0, 10.6, 7.0 Hz), 4.00 (1H, ddd, *J* = 13.0, 10.4, 6.2 Hz), 3.76 – 3.60 (2H, m), 2.75 (1H, ddd, *J* = 11.8, 10.7, 6.2 Hz), 2.39 (1H, ddd,

J = 13.6, 11.2, 5.4 Hz), 2.14 (1H, ddd, J = 13.6, 11.2, 4.9 Hz), 1.95 (1H, ddd, J = 12.1, 10.4, 7.1 Hz), 1.70 – 1.61 (1H, m), 1.59 – 1.45 (1H, m), 1.52 (9H, s).

^{13}C NMR: (100 MHz, $CDCl_3$) δ 165.8, 91.2, 84.2, 62.6, 62.1, 29.4, 28.0, 27.4, 17.3.

HRMS: (APCI-TOF) m/z: $[M+Na]^+$ calcd for $C_{11}H_{20}O_5SNa$ 287.0924; found 287.0916.

IR: ν_{max} (neat/cm^{-1}): 3548, 2935, 2873, 1723, 1319, 1151.

$[\alpha]_D^{22}$ = +60.0 (c = 0.05, $CHCl_3$).

***tert*-Butyl (2*S*)-1,1-dioxo-2-(3-oxopropyl)thietane-2-carboxylate (34)**

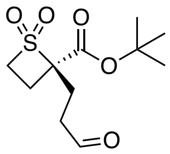

34

To a solution of **33** (2.45 g, 9.28 mmol) in CH_2Cl_2 (180 mL) was added $NaHCO_3$ (7.80 g, 92.8 mmol), followed by Dess-Martin periodinane (7.14 g, 16.8 mmol) portionwise. The reaction mixture was stirred at room temperature for 3 h. The reaction was quenched with aq. $Na_2S_2O_3$ (10%, 100 mL) and stirred for 30 min. The organic layer was separated, and the aqueous layer was extracted with CH_2Cl_2 (3 x 100 mL). The combined organic layers were washed with aq. $NaHCO_3$ (400 mL), brine (500 mL), dried ($MgSO_4$) and concentrated under reduced pressure. Purification by flash column chromatography [SiO_2; 3:1 – 2:1 hexane:EtOAc] afforded **34** (1.90 g, 78%) as a colourless solid. $\boldsymbol{R_f}$ = 0.20 [2:1 petrol:EtOAc]. **mp**: 56 – 57 ºC.

^{1}H NMR: (400 MHz, $CDCl_3$) δ 9.78 (1H, t, J = 0.9 Hz), 4.10 (1H, ddd, J = 13.0, 10.6, 7.4 Hz), 4.03 (1H, ddd, J = 12.9, 10.3, 6.0 Hz), 2.73 (1H, ddd, J = 12.1, 10.6, 6.0 Hz), 2.61 – 2.54 (3H, m), 2.48 – 2.41 (1H, m), 1.92 (1H, ddd, J = 12.1, 10.3, 7.3 Hz), 1.53 (9H, s).

^{13}C NMR: (100 MHz, $CDCl_3$) δ 199.5, 165.3, 90.3, 84.7, 62.8, 39.0, 28.0, 25.5, 17.8.

HRMS: (APCI-TOF) m/z: $[M–H]^-$ calcd for $C_{11}H_{17}O_5S$ 261.0802; found 261.0808.

IR: ν_{max} (neat/cm^{-1}): 2980, 2939, 1720, 1317, 1131.

$[\alpha]_D^{22}$ = +66.7 (c = 0.45, $CHCl_3$).

***tert*-Butyl (2*S*)-2-[3-(benzhydrylamino)propyl]-1,1-dioxo-thietane-2-carboxylate (35)**

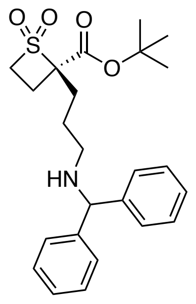

35

A solution of **34** (1.85 g, 7.06 mmol), benzhydrylamine (1.34 mL, 7.77 mmol) and acetic acid (0.81 mL, 14.12 mmol) in DCE (100 mL) was stirred at room temperature for 6 h. Sodium triacetoxyborohydride (2.25 g, 10.4 mmol) was added and the reaction was stirred at room temperature overnight. The reaction was quenched with aq. $NaHCO_3$ (100 mL). The organic layer was separated and the aqueous layer was extracted with CH_2Cl_2 (5 x 100 mL). The combined organic layer was washed with brine (500 mL), dried ($MgSO_4$) and concentrated under reduced pressure. The oily residue was triturated with MeOH (25 mL). The resulting solid was isolated by suction filtration, washed with cold MeOH (10 mL) and dried *in vacuo* to afford **35** (1.19 g). The filtrate was concentrated and purified by flash column chromatography [SiO_2, 4:1 hexane:EtOAc + 1% Et_3N] to afford an extra 0.54 g of **35**. Product from both purifications were combined to give **35** (1.73 g, 57%) as a colourless solid. $\boldsymbol{R_f}$ = 0.54 [1:1 petrol:EtOAc]. **mp**: 131 – 133 ºC.

^{1}H NMR: (400 MHz, $CDCl_3$) δ 7.40 (4H, d, *J* = 8.0 Hz), 7.32 (4H, t, *J* = 7.8 Hz), 7.25 – 7.21 (2H, m), 4.80 (1H, s), 4.08 (1H, ddd, *J* = 13.0, 10.6, 7.0 Hz), 3.99 (1H, ddd, *J* = 12.9, 10.4, 6.3 Hz), 2.78 – 2.70 (1H, m), 2.64 (2H, t, *J* = 6.8 Hz), 2.39 (1H, ddd, *J* = 13.6, 11.6, 5.2 Hz), 2.11 (1H, ddd, *J* = 13.5, 11.7, 4.8 Hz), 1.90 (1H, ddd, *J* = 12.0, 10.4, 7.0 Hz), 1.56 – 1.53 (1H, m), 1.51 (9H, s), 1.47 – 1.40 (1H, m).

^{13}C NMR: (100 MHz, $CDCl_3$) δ 165.8, 144.1, 128.7, 127.4, 127.2, 91.5, 84.0, 67.6, 62.5, 47.7, 30.8, 28.0, 25.1, 17.2.

HRMS: (ESI-TOF) m/z: $[M+H]^+$ calcd for $C_{24}H_{32}NO_4S$ 430.2047; found 430.2050.

IR: ν_{max} (neat/cm^{-1}): 3322, 2935, 2806, 1716, 1313, 1123.

$[\alpha]_D^{22}$ = +55.0 (*c* = 0.1, $CHCl_3$).

(2*S*)-2-[3-(Benzhydrylamino)propyl]-1,1-dioxo-thietane-2-carboxylic acid (27)

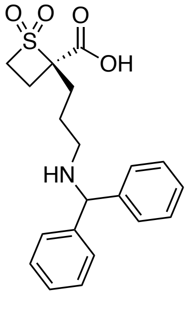

27

To a solution of **35** (1.73 g, 4.03 mmol) in CH_2Cl_2 (40 mL) was added TFA (10 mL) and the mixture was stirred at room temperature for 18 h. The reaction mixture was concentrated under reduced pressure to afford **27** (1.51 g, quant.) as a colourless solid which was used without further purification.

^{1}H NMR: (400 MHz, DMSO-d_6) δ 9.58 (1H, s, OH), 7.57 (4H, d, J = 7.4 Hz), 7.47 (4H, t, J = 7.6 Hz), 7.43 – 7.37 (2H, m), 5.60 (1H, s), 4.19 (1H, ddd, J = 13.2, 10.4, 7.6 Hz), 4.10 (1H, ddd, J = 13.2, 10.5, 5.6 Hz), 2.90 (2H, br s), 2.54 – 2.45 (1H, m), 2.14 (1H, td, J = 12.8, 4.8 Hz), 2.08 – 1.96 (2H, m), 1.81 – 1.66 (1H, m), 1.65 – 1.51 (1H, m).

^{13}C NMR: (100 MHz, DMSO-d_6) δ 167.7, 136.4, 129.1, 128.8, 127.6, 89.1, 64.5, 62.1, 43.8, 29.2, 20.7, 16.2.

HRMS: (ESI-TOF) m/z: $[M+H]^+$ calcd for $C_{20}H_{24}NO_4S$ 374.1421; found 374.1439.

IR: ν_{max} (neat/cm^{-1}): 3076, 2803, 2849, 1306, 1140.

(4*S*)-8-Benzhydryl-1,1-dioxo-1-thia-8-azaspiro[3.5]nonan-9-one (36)

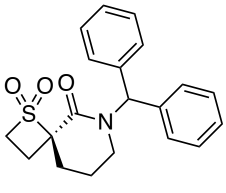

36

A suspension of crude **27** (1.51 g, 4.03 mmol) in CH_2Cl_2 (100 mL) was cooled to 0 ºC. *N*-Ethyl-*N'*-(3-dimethylaminopropyl)carbodiimide hydrochloride (1.54 g, 8.06 mmol), pyridine (1.63 mL, 20.2 mmol) and DMAP (54 mg, 0.04 mmol) were added sequentially. The reaction

mixture was stirred at 0 ºC for 30 min, then allowed to warm to room temperature and stirred at ambient temperature for 24 h. The reaction was quenched with aq. HCl (1 N, 100 mL), extracted with CH_2Cl_2 (3 x 100 mL), washed with brine (300 mL), dried ($MgSO_4$) and concentrated under reduced pressure. Purification by flash column chromatography [SiO_2; 3:1 – 2:1 hexane:EtOAc] afforded **36** (1.27 g, 89%) as a colourless solid. $\boldsymbol{R}_f$ = 0.19 [2:1 petrol:EtOAc]. **mp**: 144 – 145 ºC.

^{1}H NMR: (400 MHz, $CDCl_3$) δ 7.40 – 7.32 (5H, m), 7.32 – 7.27 (3H, m), 7.26 – 7.22 (2H, m), 7.20 (1H, s), 4.43 (1H, ddd, J = 12.4, 10.6, 9.8 Hz), 3.96 (1H, ddd, J = 12.8, 10.0, 3.0 Hz), 3.29 – 3.22 (1H, m), 3.05 – 2.96 (1H, m), 2.91 (1H, td, J = 11.3, 2.9 Hz), 2.87 – 2.79 (1H, m), 1.98 – 1.88 (2H, m), 1.85 – 1.76 (2H, m).

^{13}C NMR: (100 MHz, $CDCl_3$) δ 165.2, 138.4, 137.7, 129.8, 128.8, 128.7, 128.1, 128.1, 127.5, 88.7, 64.3, 61.4, 44.0, 28.7, 21.3, 19.2.

HRMS: (ESI-TOF) m/z: $[M+H]^+$ calcd for $C_{20}H_{22}NO_3S$ 356.1315; found 356.1315.

IR: ν_{max} (neat/cm^{-1}): 3030, 2935, 2870, 1627, 1310, 1123.

$[\alpha]_D^{22}$ = +151.6 (c = 0.32, $CHCl_3$).

(4*S*)-8-Benzhydryl-1-thia-8-azaspiro[3.5]nonane 1,1-dioxide (37)

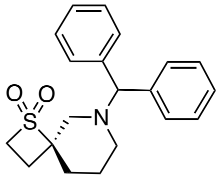

37

To a solution of **36** (1.23 g, 3.46 mmol) in THF (25 mL) was added BH_3·THF (1 M in THF, 10.4 mL, 10.4 mmol) and the mixture was heated at reflux for 3 h. A fresh portion of BH_3·THF (1 M in THF, 10.4 mL, 10.4 mmol) was added and the mixture was stirred at reflux for a further 2 h. The mixture was allowed to cool to room temperature, quenched dropwise with water (15 mL), and basified with aq. NaOH (10%, 100 mL). The mixture was extracted with EtOAc (3 x 100 mL), washed with brine (300 mL), dried ($MgSO_4$) and concentrated under reduced pressure. Purification by flash column chromatography [SiO_2; 4:1 hexane:EtOAc] afforded **37** (1.13 g, 96%) as a colourless solid. $\boldsymbol{R}_f$ = 0.30 [2:1 petrol:EtOAc]. **mp**: 163 ºC (decomposition).

^{1}**H NMR**: (400 MHz, $CDCl_3$) δ 7.48 – 7.38 (4H, m), 7.31 – 7.27 (4H, m), 7.22 – 7.16 (2H, m), 4.35 (1H, s), 3.89 (1H, ddd, J = 13.1, 10.4, 7.3 Hz), 3.77 (1H, ddd, J = 13.1, 10.5, 5.7 Hz), 3.10 (1H, d, J = 11.5 Hz), 2.56 – 2.45 (2H, m), 2.32 – 2.25 (1H, m), 2.24 – 2.14 (1H, m), 2.01 (1H, td, J = 11.6, 7.4 Hz), 1.90 – 1.71 (3H, m), 1.68 – 1.57 (1H, m).

^{13}**C NMR**: (100 MHz, $CDCl_3$) δ 142.2, 141.8, 128.7, 128.6, 128.4, 128.0, 127.3, 127.3, 84.0, 76.1, 59.6, 56.1, 51.3, 31.5, 23.2, 20.7.

HRMS: (ESI-TOF) m/z: $[M+H]^+$ calcd for $C_{20}H_{24}NO_2S$ 342.1522; found 342.1509.

IR: ν_{max} (neat/cm^{-1}): 3058, 2940, 2785, 1304, 1120.

$[\alpha]_D^{22}$ = +39.1 (c = 0.32, $CHCl_3$).

(4*S*)-1-Thia-8-azaspiro[3.5]nonane 1,1-dioxide (38)

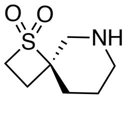

38

To a suspension of **37** (1.09 g, 3.20 mmol) in EtOH (50 mL) was added TFA (5 mL) and $Pd(OH)_2$ (220 mg, 20 wt% on carbon). The reaction was stirred under a hydrogen atmosphere for 2.5 h. The reaction mixture was filtered through a plug of Celite and concentrated. The residue was suspended between EtOAc (75 mL) and aq. HCl (1 N, 25 mL). The aqueous layer was basified with 3 N NaOH (250 mL) to pH >10 and extracted with CH_2Cl_2 (5 x 100 mL). The combined organic phase was washed with brine (500 mL), dried ($MgSO_4$) and concentrated under reduced pressure to give **38** (451 mg, 81%) as a yellow oil. R_f = baseline [EtOAc].

^{1}**H NMR**: (400 MHz, DMSO-d_6) δ 4.06 (2H, t, J = 8.7 Hz), 3.44 (1H, d, J = 13.2 Hz), 3.32 (1H, br s), 3.23 (1H, d, J = 13.2 Hz), 2.91 – 2.80 (2H, m), 2.23 – 2.15 (1H, m), 2.09 – 1.99 (1H, m), 1.94 – 1.87 (2H, m), 1.68 (2H, quint., J = 5.8 Hz).

^{13}**C NMR**: (100 MHz, DMSO-d_6) δ 79.8, 60.0, 46.6, 43.2, 28.4, 20.9, 19.2.

HRMS: (APCI-TOF) m/z: $[M+H]^+$ calcd for $C_7H_{14}NO_2S$ 176.0740; found 176.0744.

IR: ν_{max} (neat/cm^{-1}): 3330, 2942, 2855, 1297, 1121.

$[\alpha]_D^{22}$ = +30.0 (c = 0.05, $CHCl_3$).

(4*S*)-1-Thia-8-azaspiro[3.5]nonane 1,1-dioxide hydrochloride (12)

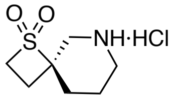

12

38 (449 mg, 2.56 mmol) was dissolved in a mixture of Et_2O (150 mL) and EtOAc (30 mL). HCl in 1,4-dioxane (4 N, 1.28 mL, 5.12 mmol) was added dropwise. The reaction mixture was stirred at room temperature for 2 h. The precipitate was isolated by centrifuge to provide salt **12** (512 mg, 95%) as a colourless solid.

^{1}H NMR: (400 MHz, DMSO-d_6) δ 9.92 (1H, br s), 9.41 (1H, br s), 4.23 – 4.13 (2H, m), 3.52 – 3.45 (1H, m), 3.43 – 3.35 (1H, m), 3.06 – 2.96 (1H, m), 2.94 – 2.84 (1H, m), 2.38 – 2.28 (1H, m), 2.14 – 2.06 (1H, m), 2.03 – 1.92 (2H, m), 1.82 – 1.71 (2H, m).

^{13}C NMR: (100 MHz, DMSO-d_6) δ 78.5, 60.5, 44.5, 42.0, 27.2, 19.5, 18.9.

HRMS: (APCI-TOF) m/z: $[M+H]^+$ calcd for $C_7H_{14}NO_2S$ 176.0740; found 176.0731.

IR: ν_{max} (neat/cm^{-1}): 2924, 2786, 2719, 2611, 1295, 1123.

$[\alpha]_D^{21}$ = +5.6 (c = 0.27, MeOH).

*To determine the ee of **12**, amine **12** was converted to carbamate **28d**:*

***tert*-Butyl (4*S*)-1,1-dioxo-1-thia-8-azaspiro[3.5]nonane-8-carboxylate (28d)**

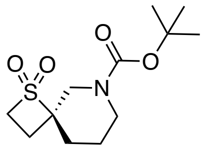

28d

A solution of **12** (14 mg, 0.07 mmol), triethylamine (40 μL, 0.28 mmol), di-*tert*-butyl dicarbonate (60 μL, 0.13 mmol) and a few crystals of DMAP in CH_2Cl_2 (5 mL) was stirred at room temperature for 48 h. The reaction was quenched with HCl (1 N, 5 mL). The aqueous

phase was extracted with CH_2Cl_2 (3 x 15 mL). The combined organic extracts were washed with brine (20 mL), dried ($MgSO_4$) and concentrated under reduced pressure. Purification by flash column chromatography [SiO_2; 1:1 – 0:1 hexane:EtOAc] afforded **28d** (19 mg, 98%) as a colourless oil. $\boldsymbol{R}_f$ = 0.72 [EtOAc].

^{1}H NMR: (400 MHz, DMSO-d_6, VT, 75 ºC) δ 4.06 – 3.93 (3H, m), 3.51 (1H, d, J = 13.6 Hz), 3.47 – 3.39 (1H, m), 3.31 – 3.15 (1H, m), 2.26 – 2.18 (1H, m), 1.94 – 1.84 (3H, m), 1.62 – 1.53 (2H, m), 1.42 (9H, s).

^{13}C NMR: (100 MHz, DMSO-d_6, VT, 75 ºC) δ 153.3, 81.2, 78.8, 59.3, 46.5, 42.1, 29.5, 27.7, 21.7, 18.6.

HRMS: (ESI-TOF) m/z: $[M+Na]^+$ calcd for $C_{12}H_{21}NO_4SNa$ 298.1084, found 298.1061.

IR: ν_{max} (neat/cm^{-1}): 2974, 2862, 1688, 1310, 1125.

Chiral HPLC: (AD-H, hexane/*i*-PrOH = 95/5, flow rate = 1.0 mL/min, l = 212 nm) t_R = 9.4 min (minor), 24.1 min (major). 96% ee.

$[\alpha]_D^{22}$ = –12.5 (c = 0.12, $CHCl_3$).

For the purposes of HPLC analysis, *rac*-**12** was Boc-protected to *rac*-**28d** using the same procedure.

2.6. Functionalisations of Spirocycle 12

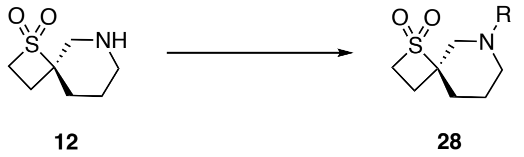

(4*S*)-8-[(4-Bromophenyl)methyl]-1-thia-8-azaspiro[3.5]nonane 1,1-dioxide (28a)

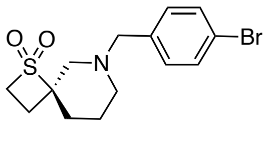

28a

A solution of **12** (28 mg, 0.15 mmol), acetic acid (17 mL, 0.30 mmol), and 4-bromobenzaldehyde (31 mg, 0.17 mmol) in DCE (4 mL) was stirred at room temperature for 15 minutes. Sodium triacetoxyborohydride (48 mg, 0.23 mmol) was added and the reaction was stirred at room temperature overnight. The reaction was quenched with aq. $NaHCO_3$ (10 mL), the aqueous phase was extracted with CH_2Cl_2 (3 x 15 mL). The organic extracts were combined, washed with brine (20 mL), dried ($MgSO_4$) and concentrated under reduced pressure. Purification by flash column chromatography [SiO_2; 2:1 pentane:EtOAc] afforded **28a** (27 mg, 53%) as a colourless oil. $\boldsymbol{R}_f$ = 0.71 [EtOAc].

^{1}H NMR: (400 MHz, $CDCl_3$) δ 7.43 (2H, d, *J* = 8.4 Hz), 7.22 (2H, d, *J* = 8.5 Hz), 3.91 (1H, ddd, *J* = 13.2, 10.4, 7.4 Hz), 3.84 (1H, ddd, *J* = 13.2, 10.5, 6.0 Hz), 3.51 (2H, s), 3.01 (1H, d, *J* = 11.6 Hz), 2.53 (1H, d, *J* = 11.8 Hz), 2.51 – 2.42 (1H, m), 2.34 – 2.16 (2H, m), 2.00 – 1.92 (1H, m), 1.90 – 1.75 (3H, m), 1.68 – 1.61 (1H, m).

^{13}C NMR: (100 MHz, $CDCl_3$) δ 136.9, 131.4, 130.5, 121.0, 83.3, 62.0, 59.6, 57.2, 52.6, 30.9, 22.8, 20.4.

HRMS: (ESI-TOF) m/z: $[M+H]^+$ calcd for $C_{14}H_{19}NO_2S^{79}Br$ 344.0314; found 344.0302.

IR: ν_{max} (neat/cm^{-1}): 2942, 2789, 1302, 1120.

$[\alpha]_D^{22}$ = –3.8 (*c* = 0.26, $CHCl_3$).

(4*S*)-(4-Bromophenyl)-[1,1-dioxo-1-thia-8-azaspiro[3.5]nonan-8-yl]methanone (28b)

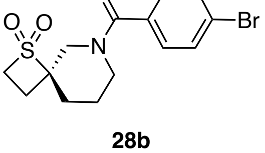

28b

A solution of **38** (14 mg, 0.07 mmol) and triethylamine (20 mL, 0.14 mmol) in CH_2Cl_2 (3 mL) was cooled to 0 ºC. A solution of 4-bromobenzoyl chloride (22 mg, 0.10 mmol) in CH_2Cl_2 (1 mL) was added. The reaction was stirred at 0 ºC for 10 minutes, then allowed to warm to room temperature and stirred at ambient temperature overnight. The reaction was quenched with aq. $NaHCO_3$ (10 mL). The aqueous phase was extracted with CH_2Cl_2 (3 x 15 mL). The combined organic extracts were washed with brine (20 mL), dried ($MgSO_4$) and concentrated under

reduced pressure. Purification by flash column chromatography [SiO_2; 1:1 – 0:1 hexane:EtOAc] afforded **28b** (21 mg, 84%) as a colourless sticky oil. ***R*f** = 0.34 [EtOAc].

^{1}H NMR: (400 MHz, DMSO-d_6, VT, 130 ºC) δ 7.63 (2H, d, *J* = 8.4 Hz), 7.33 (2H, d, *J* = 8.4 Hz), 4.14 (1H, d, *J* = 14.0 Hz), 4.05 – 3.93 (2H, m), 3.68 (1H, d, *J* = 14.0 Hz), 3.60 (1H, br s), 3.22 – 3.13 (1H, m), 2.38 – 2.29 (1H, m), 2.00 – 1.85 (3H, m), 1.69 – 1.59 (2H, m).

^{13}C NMR: (100 MHz, DMSO-d_6, VT, 130 ºC) δ 168.1, 134.7, 130.6, 128.3, 122.2, 81.3, 59.3, 46.9, 43.6, 29.4, 21.5, 18.6.

HRMS: (ESI-TOF) m/z: $[M+H]^+$ calcd for $C_{14}H_{17}NO_3S^{79}Br$ 358.0107; found 358.0111.

IR: ν_{max} (neat/cm^{-1}): 2950, 2862, 1634, 1306, 1123.

$[\alpha]_D^{22}$ = –21.9 (*c* = 0.16, $CHCl_3$).

(4*S*)-1-[4-[1,1-Dioxo-1-thia-8-azaspiro[3.5]nonan-8-yl]phenyl]ethanone (28c)

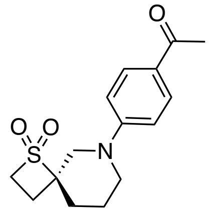

28c

A solution of **12** (28 mg, 0.15 mmol) and 4-bromoacetophenone (90 mg, 0.45 mmol) in toluene (2 mL) was added to a flask containing $Pd(OAc)_2$ (3.5 mg, 0.02 mmol), BINAP (19 mg, 0.03 mmol) and Cs_2CO_3 (75 mg, 0.23 mmol). The mixture was stirred at room temperature for 30 minutes, then heated to 100 ºC overnight. The mixture was allowed to cool to room temperature, filtered through a plug of celite and concentrated under reduced pressure. Purification by flash column chromatography [SiO_2; 3:2 pentane:EtOAc] afforded **28c** (27 mg, 61%) as a colourless oil. ***R*f** = 0.56 [EtOAc].

^{1}H NMR: (400 MHz, $CDCl_3$) δ 7.89 (2H, d, *J* = 8.8 Hz), 7.00 (2H, d, *J* = 8.8 Hz), 4.05 (1H, d, *J* = 13.2 Hz), 4.03 – 3.96 (2H, m), 3.46 – 3.41 (1H, m), 3.38 (1H, d, *J* = 13.2 Hz), 3.15 – 3.08 (1H, m), 2.52 (3H, s), 2.50 – 2.43 (1H, m), 2.07 – 1.90 (4H, m), 1.87 – 1.79 (1H, m).

^{13}C NMR: (100 MHz, $CDCl_3$) δ 196.8, 154.4, 130.4, 129.0, 115.4, 82.7, 60.0, 53.6, 47.6, 31.0, 26.4, 22.9, 20.3.

HRMS: (APCI-TOF) m/z: $[M+H]^+$ calcd for $C_{15}H_{20}NO_3S$ 294.1158; found 294.1144.

IR: ν_{max} (neat/cm^{-1}): 2946, 2823, 1664, 1597, 1304, 1118.

$[\alpha]_D^{22} = -137.5$ (c = 0.04, $CHCl_3$).

3. Mechanistic Study

3.1 Synthesis of *E*- and *Z*- Enol Carbonates 20

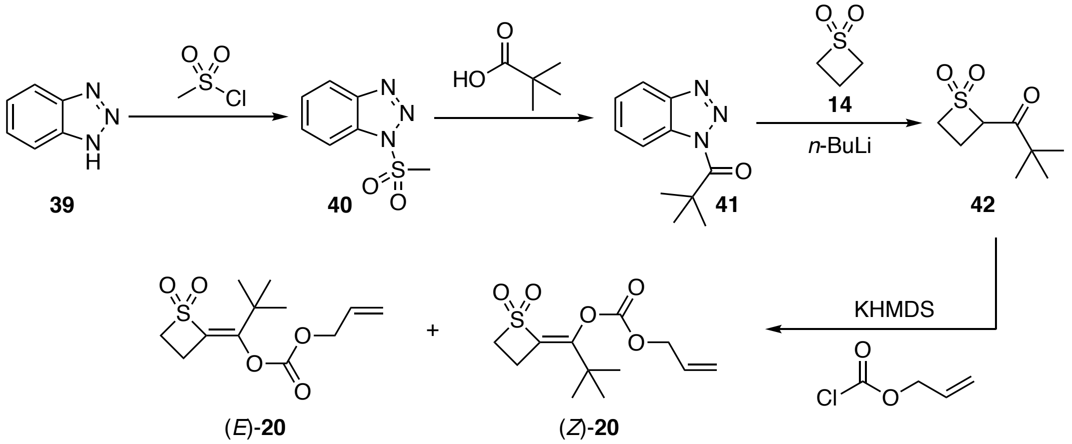

1-Methylsulfonylbenzotriazole (40)

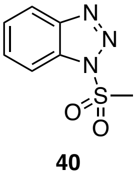

40

40 was prepared using a literature procedure.[3]

1-(Benzotriazol-1-yl)-2,2-dimethyl-propan-1-one (41)

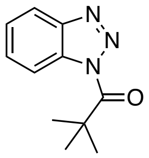

41

A solution of **40** (750 mg, 3.78 mmol), pivalic acid (435 μL, 3.78 mmol) and triethylamine (0.72 mL, 5.10 mmol) in THF (20 mL) was heated to 80 ºC for 18 h. The mixture was cooled to room temperature, then concentrated under reduced pressure. The residue was dissolved in $CHCl_3$ (30 mL), washed with water (10 mL), dried ($MgSO_4$) and concentrated to afford **41** (755 mg, 98%) as a colourless solid. $\boldsymbol{R_f}$ = 0.74 [2:1 petrol:EtOAc]. **mp**: 58 – 60 ºC.

^{1}H NMR: (400 MHz, $CDCl_3$) δ 8.30 (1H, dt, *J* = 8.3, 0.9 Hz), 8.11 (1H, dt, *J* = 8.2, 0.9 Hz), 7.66 – 7.61 (1H, m), 7.52 – 7.45 (1H, m), 1.64 (9H, s).

^{13}C NMR: (100 MHz, $CDCl_3$) δ 177.6, 145.1, 132.4, 130.4, 126.1, 120.1, 115.2, 42.7, 27.9.

HRMS: (APCI-TOF) m/z: $[M+H]^+$ calcd for $C_{11}H_{14}N_3O$ 204.1131; found 204.1122.

IR: ν_{max} (neat/cm^{-1}): 3026, 2976, 2935, 1720.

1-(1,1-Dioxothietan-2-yl)-2,2-dimethyl-propan-1-one (42)

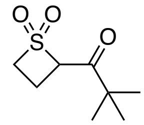

42

A solution of **14** (365 mg, 3.45 mmol) in THF (20 mL) was cooled to 0 ºC. *n*-BuLi (1.51 M, 4.8 mL, 7.24 mmol) was added dropwise and the mixture was stirred at 0 ºC for 1 h. The mixture was cooled to –78 ºC. A solution of **41** (700 mg, 3.45 mmol) in THF (6 mL) was added dropwise and the mixture was stirred at –78 ºC for 3 h. The reaction was quenched at –78 ºC with aq. NH_4Cl (3 mL) and allowed to warm to room temperature. The mixture was diluted with water (10 mL), extracted with EtOAc (3 x 20 mL), washed with brine (10 mL), dried ($MgSO_4$) and concentrated under reduced pressure. Purification by flash column

chromatography [SiO_2; 7:1 – 4:1 hexane:EtOAc] afforded **42** (113 mg, 17%) as a colourless oil. ***R*f** = 0.29 [2:1 petrol:EtOAc].

^{1}H NMR: (400 MHz, $CDCl_3$) δ 5.56 – 5.51 (1H, m), 4.19 – 4.08 (2H, m), 2.73 – 2.61 (1H, m), 2.15 – 2.06 (1H, m), 1.22 (9H, s).

^{13}C NMR: (100 MHz, $CDCl_3$) δ 203.1, 80.7, 64.5, 43.9, 25.7, 8.2.

HRMS: (APCI-TOF) m/z: $[M+H]^+$ calcd for $C_8H_{15}O_3S$ 191.0736; found 191.0733.

IR: ν_{max} (neat/cm^{-1}): 2972, 2875, 1709, 1325, 1138.

Allyl [(1*Z*)-1-(1,1-dioxothietan-2-ylidene)-2,2-dimethyl-propyl] carbonate (*Z*-20) and **Allyl [(1*E*)-1-(1,1-dioxothietan-2-ylidene)-2,2-dimethyl-propyl] carbonate (*E*-20)**

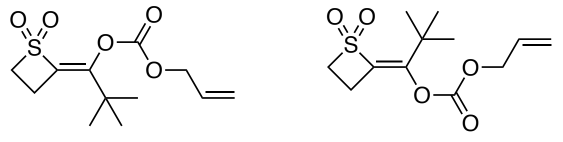

(*Z*)-**20** (*E*)-**20**

A solution of **42** (25 mg, 0.13 mmol) in THF (2 mL) was added to a solution of KHMDS (0.5 M in toluene, 0.36 mL, 0.18 mmol) in THF (1 mL), and the mixture was stirred at room temperature for 1 h. Allyl chloroformate (20 µL, 0.18 mmol) was added dropwise and the reaction mixture was stirred at room temperature for 3 h. The reaction was quenched with aq. HCl (1N, 3 mL) and diluted with water (3 mL). The mixture was extracted with EtOAc (3 x 10 mL), washed with brine (5 mL), dried ($MgSO_4$) and concentrated under reduced pressure. Purification by flash column chromatography [SiO_2; 9:1 hexane:EtOAc] afforded **(*Z*)-20** (12 mg, 34%) as a colourless solid, and **(*E*)-20** (6 mg, 17%) as a colourless oil.

(*Z*)-20:

***R*f** = 0.29 [2:1 petrol:EtOAc]. **mp**: 61 – 63 ºC.

^{1}H NMR: (400 MHz, $CDCl_3$) δ 5.97 (1H, ddt, *J* = 17.2, 10.4, 5.8 Hz), 5.40 (1H, dq, *J* = 17.2, 1.4 Hz), 5.31 (1H, dq, *J* = 10.4, 1.2 Hz), 4.72 (2H, dt, *J* = 5.8, 1.3 Hz), 4.02 – 3.96 (2H, m), 3.04 – 2.99 (2H, m), 1.22 (9H, s).

^{13}C NMR: (100 MHz, $CDCl_3$) δ 156.6, 152.3, 142.9, 131.1, 119.7, 70.0, 62.4, 37.4, 27.6, 16.7.

IR: ν_{max} (neat/cm^{-1}): 2978 (C–H), 1768 (C=O), 1313 (S=O), 1142 (S=O).

HRMS: (APCI-TOF) m/z: $[M+H]^+$ calcd for $C_{12}H_{19}O_5S$ 275.0948; found 275.0954.

(*E*)-20:

***R*f** = 0.62 [2:1 petrol:EtOAc].

^{1}H NMR: (400 MHz, $CDCl_3$) δ 5.95 (1H, ddt, *J* = 17.2, 10.4, 5.9 Hz), 5.41 (1H, dq, *J* = 17.2, 1.4 Hz), 5.34 (1H, dq, *J* = 10.4, 1.2 Hz), 4.70 (2H, dt, *J* = 5.8, 1.3 Hz), 3.95 – 3.86 (2H, m), 2.68 – 2.59 (2H, m), 1.28 (9H, s).

^{13}C NMR: (100 MHz, $CDCl_3$) δ 158.7, 150.8, 143.3, 130.9, 120.1, 69.7, 59.3, 37.6, 27.0, 14.3.

IR: ν_{max} (neat/cm^{-1}): 3019, 2970, 1763, 1317, 1127.

Selected 1D nOe was observed between H1 and H2 in (*Z*)-**20** and not in (*E*)-**20**.

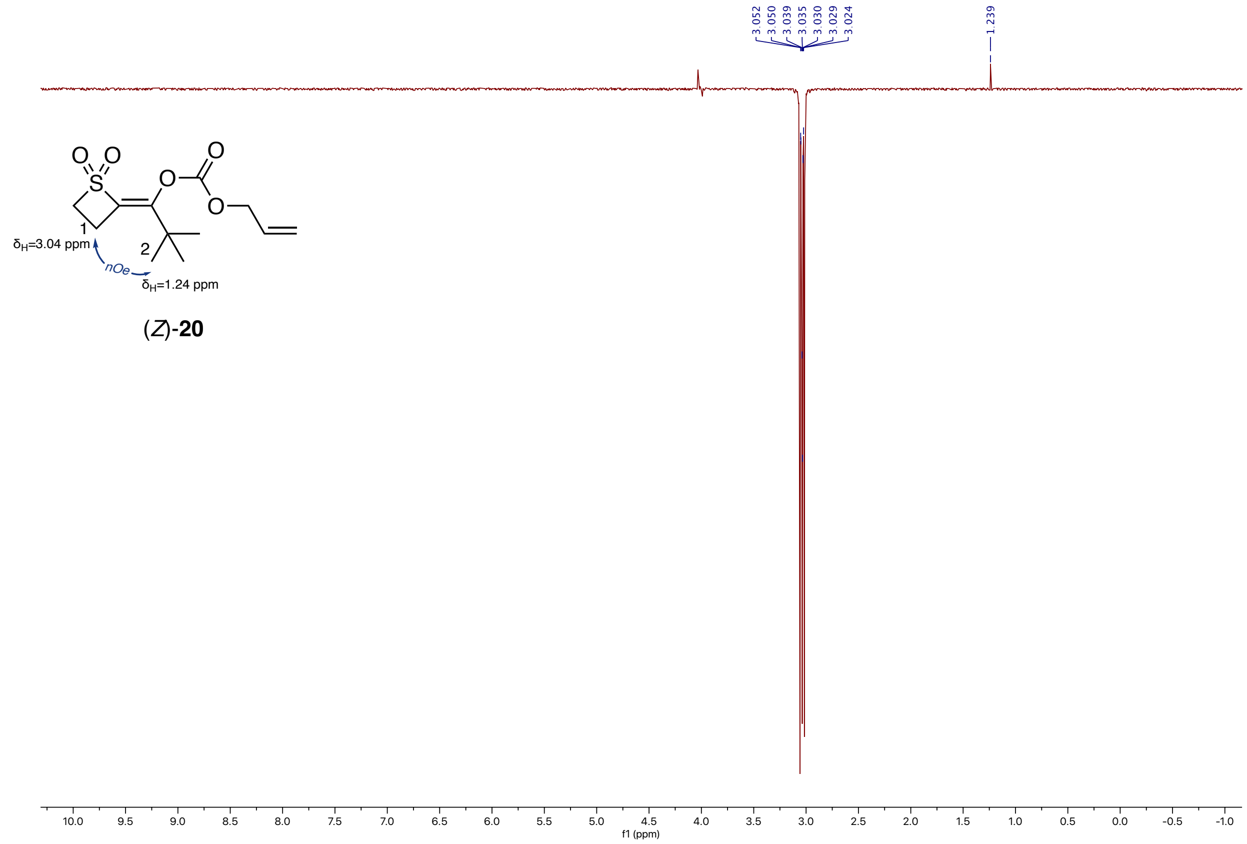
3.052
3.050
3.039
3.035
3.030
3.029
3.024
1.239
δH=3.04 ppm
nOe
δH=1.24 ppm
1
2
f1 (ppm)

(*Z*)-**20**

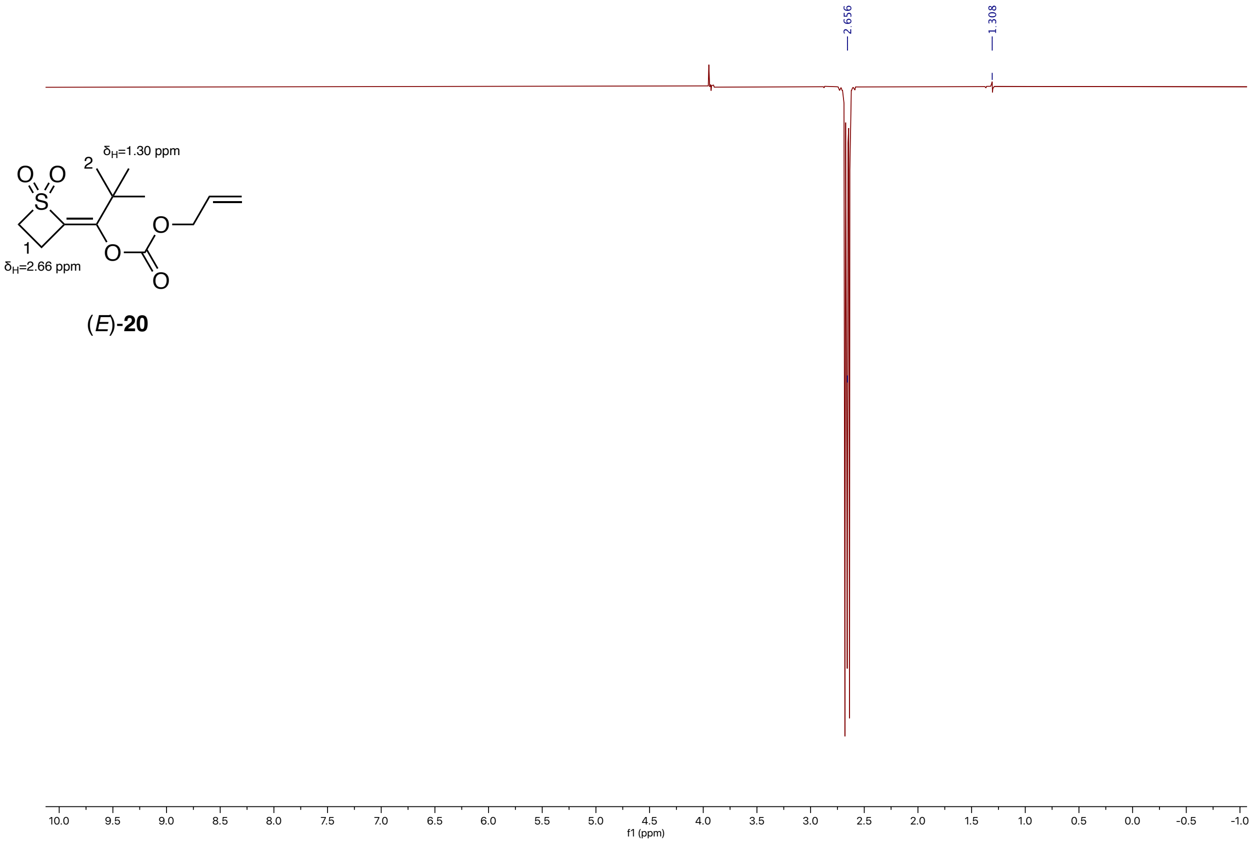
2.656
1.308
δH=1.30 ppm
δH=2.66 ppm
1
2
f1 (ppm)

(*E*)-**20**

(2*S*)-1-[2-Allyl-1,1-dioxo-thietan-2-yl]-2,2-dimethyl-propan-1-one (18p)

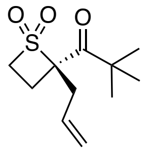

18p

A vial was charged with (*Z*)-**20** (20 mg, 0.073 mmol), $Pd_2(dba)_3$ (1.7 mg, 0.0018 mmol), (*S*,*S*)-ANDEN Trost ligand **L4** (3.8 mg, 0.047 mmol) and 1,4-dioxane (1.8 mL). The reaction mixture was stirred at room temperature for 18 h. The mixture was concentrated under reduced pressure. Purification by flash chromatography [SiO_2; 5:1 hexane:EtOAc] afforded **18p** (12.0 mg, 71%) as a colourless oil. R_f = 0.34 [2:1 petrol: EtOAc].

Chiral HPLC: (OD-H, hexane/*i*-PrOH = 95/5, flow rate = 1.0 mL/min, l = 220 nm) t_R = 9.7 min (minor), 10.3 min (major). 88% ee.

A vial was charged with (*E*)-**20** (20 mg, 0.073 mmol), $Pd_2(dba)_3$ (1.7 mg, 0.0018 mmol), (*S*,*S*)-ANDEN Trost ligand **L4** (3.8 mg, 0.047 mmol) and 1,4-dioxane (1.8 mL). The reaction mixture was stirred at room temperature for 18 h. The mixture was concentrated under reduced pressure. Purification by flash chromatography [SiO_2; 5:1 hexane:EtOAc] afforded **18p** (14.2 mg, 85%) as a colourless oil. R_f = 0.34 [2:1 petrol: EtOAc].

Chiral HPLC: (OD-H, hexane/*i*-PrOH = 95/5, flow rate = 1.0 mL/min, l = 220 nm) t_R = 9.7 min (minor), 10.3 min (major). 76% ee.

3.2 Crossover Study

3-Trimethylsilylprop-2-ynyl 1,1-dioxothietane-2-carboxylate (43)

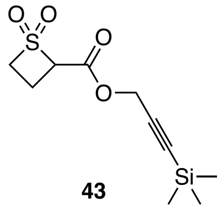

43

A suspension of **30** (1.0 g, 6.66 mmol), 3-(Trimethylsilyl)propargyl alcohol (1.97 mL, 13.3 mmol) and a few crystals of 4-dimethylaminopyridine in CH_2Cl_2 (15 mL) was cooled to 0 ºC. A solution of *N*,*N'*-dicyclohexylcarbodiimide (1.51 g, 7.32 mmol) in CH_2Cl_2 (10 mL) was added. The mixture was allowed to warm to room temperature and stirred at ambient temperature for 18 h. The reaction mixture was filtered through Celite twice, then concentrated under reduced pressure. Purification using flash column chromatography [SiO_2; 10:1 – 4:1 hexane:EtOAc] afforded **43** (1.13 g, 65%) as a colourless solid. R_f = 0.18 [4:1 petrol:EtOAc]. **mp**: 86 – 87 ºC.

^{1}H NMR: (400 MHz, $CDCl_3$) δ 5.14 –5.08 (1H, m), 4.93 (1H, d, *J* = 15.6 Hz), 4.74 (1H, d, *J* = 15.6 Hz), 4.27 – 4.15 (2H, m) 2.65 – 2.55 (1H, m), 2.38 – 2.28 (1H, m), 0.19 (9H, s).

^{13}C NMR: (100 MHz, $CDCl_3$) δ 163.7, 97.7, 93.7, 79.6, 65.5, 54.8, 9.7, –0.3.

HRMS: (APCI-TOF) m/z: $[M–H]^-$ calcd for $C_{10}H_{15}O_4SiS$ 259.0466; found 259.0466.

IR: ν_{max} (neat/cm^{-1}): 2972, 1735, 1317, 1127.

[D]-Prop-2-ynyl 1,1-dioxothietane-2-carboxylate (44)

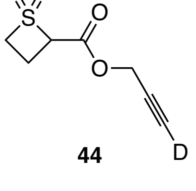

44

43 (400 mg, 1.54 mmol) was dissolved in THF (6 mL). Deuterium oxide (8 mL) was added, followed by tetrabutylammonium fluoride (1M in THF, 1.70 mL, 1.70 mmol), and the reaction mixture was stirred at room temperature for 2 h. The mixture was diluted with water, extracted with EtOAc (3 x 15 mL), washed brine (15 mL), dried ($MgSO_4$), and concentrated under

reduced pressure. Purification by flash column chromatography [SiO_2; 5:1 hexane:EtOAc] afforded **44** (109 mg, 38%, 88% D) as a colourless oil. $\boldsymbol{R_f}$ = 0.22 [3:1 petrol:EtOAc].

^{1}H NMR: (400 MHz, $CDCl_3$) δ 5.13 – 5.08 (1H, m), 4.91 (1H, d, J = 15.6 Hz), 4.78 (1H, d, J = 15.6 Hz), 4.27 – 4.16 (2H, m), 2.66 – 2.57 (1H, m), 2.54 (0.12H, t, J = 2.4 Hz), 2.39 – 2.29 (1H, m).

^{13}C NMR: (100 MHz, $CDCl_3$) δ 163.7, 79.5, 65.5, 54.0, 9.7.

HRMS: (APCI-TOF) m/z: $[M–H]^–$ calcd for $C_7H_6DO_4S$ 188.0133; found 188.0149.

IR: $ν_{max}$ (neat/cm^{-1}): 2970, 2584, 1740, 1317, 1131.

[D]-Allyl 1,1-dioxothietane-2-carboxylate ([D]-15)

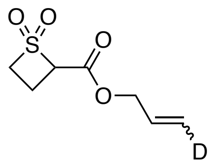

[D]-**15**

A suspension of **44** (100 mg, 0.53 mmol), Pd/$CaCO_3$ (10mg) and quinoline (125 μL, 1.06 mmol) in EtOAc (7 mL) was degassed with argon. The mixture was cooled to 0 ºC, and then stirred under a hydrogen atmosphere for 30 minutes. The suspension was filtered through a pad of celite, washed with aq. HCl (1 N, 5 mL), brine (5 mL), dried ($MgSO_4$), and concentrated under reduced pressure. Purification by flash column chromatography [SiO_2; 4:1 hexane:EtOAc] afforded [D]-**15** (65 mg, 64%, 86% D) as an orange oil. $\boldsymbol{R_f}$ = 0.26 [3:1 petrol:EtOAc].

^{1}H NMR: (400 MHz, $CDCl_3$) δ 5.99 – 5.88 (1H, m), 5.42 – 5.35 (0.30H, m), 5.32 – 5.27 (0.84H, m), 5.12 – 5.06 (1H, m), 4.80 – 4.69 (2H, m), 4.25 – 4.14 (2H, m), 2.66 – 2.54 (1H, m), 2.37 – 2.27 (1H, m).

^{13}C NMR: (100 MHz, $CDCl_3$) δ 164.0, 131.0, 119.4, 79.8, 67.3, 65.3, 9.7.

[D]-Allyl 2-(4-methylbenzoyl)-1,1-dioxo-thietane-2-carboxylate ([D]-16c)

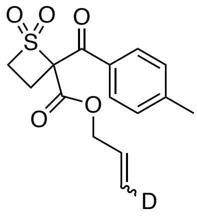

[D]-**16c**

A solution of NaHMDS (1 M in THF, 0.35 mL, 0.35 mmol) in THF (5 mL) was cooled to 0 ºC. A solution of allyl ester [D]-**15** (60 mg, 0.31 mmol) in THF (5 mL) was added dropwise and the reaction mixture was stirred at 0 ºC for 20 minutes. *p*-Tolyl chloride (47 μL, 0.35 mmol) was added dropwise and the mixture stirred at 0 ºC for 3 h. The reaction was quenched with aq. HCl (1 N, 1 mL), allowed to warm to room temperature and diluted with water (10 mL). The mixture was extracted with EtOAc (3 x 15 mL), washed with brine (10 mL), dried ($MgSO_4$) and concentrated under reduced pressure. Purification by flash column chromatography [SiO_2; 4:1 hexane:EtOAc] afforded [D]-**16c** (61 mg, 63%, 86% D) as a colourless oil. $\boldsymbol{R_f}$ = 0.58 [3:1 petrol:EtOAc].

^{1}H NMR: (400 MHz, $CDCl_3$) δ 7.89 (2H, dt, *J* = 8.4, 2.0 Hz), 7.32 – 7.28 (2H, m), 5.84 – 5.74 (1H, m), 5.26 – 5.22 (0.23H, m), 5.22 – 5.18 (0.91H, m), 4.71 (2H, dddd, *J* = 18.8, 13.2, 5.6, 1.2 Hz), 4.45 (1H, dt, *J* = 12.4, 10.4 Hz), 4.07 (1H, ddd, *J* = 12.4, 10.0, 3.2 Hz), 2.95 (1H, dt, *J* = 12.4, 10.4 Hz), 2.77 (1H, ddd, *J* = 12.4, 10.4, 3.2 Hz), 2.42 (3H, s).

^{13}C NMR: (100 MHz, $CDCl_3$) δ 187.1, 164.6, 145.8, 132.3, 130.3, 129.9, 129.6, 119.7, 95.8, 67.9, 63.5, 22.0, 17.6.

HRMS: (APCI-TOF) m/z: $[M+H]^+$ calcd for $C_{15}H_{16}DO_5S$ 310.0854; found 310.0853.

IR: ν_{max} (neat/cm^{-1}): 3034, 2959, 1731, 1677, 1332, 1140.

A vial was charged with **16a** (25 mg, 0.085 mmol), [D]-**16c** (26 mg, 0.084 mmol), $Pd_2(dba)_3$ (3.5 mg, 0.004 mmol), (*S*,*S*)-ANDEN Trost Ligand (**L4**) (8.9 mg, 0.011 mmol) and 1,4-dioxane (4 mL). The reaction mixture was stirred at room temperature for 18 h. The mixture was concentrated under reduced pressure. Purification by flash chromatography [SiO_2; 7:1 hexane:EtOAc] afforded [D]/**18a** and [D]/**18c** (29 mg) in a 1:1.2 ratio, corresponding to 12.8 mg, 51% yield of [D]/**18a** and 16.2 mg, 63% yield of [D]/**18c**.

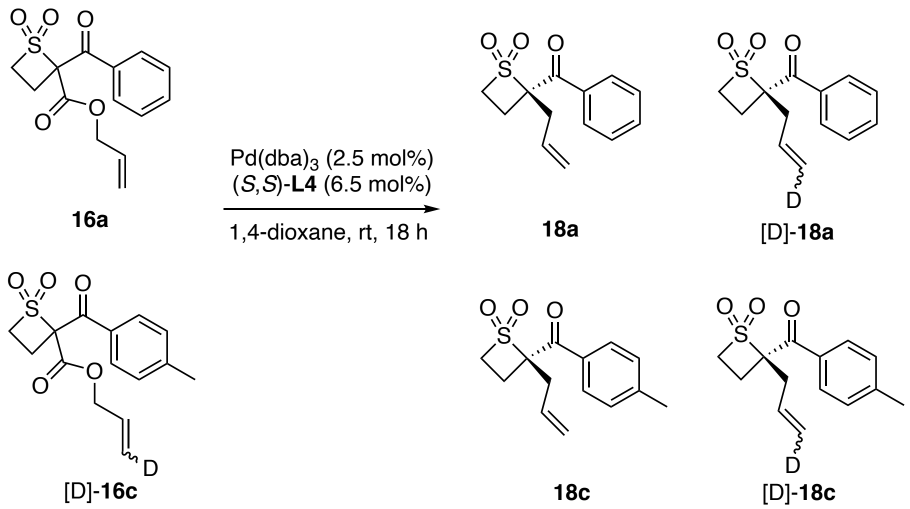

The presence of **18a**, [D]-**18a**, **18c** and [D]-**18c** was confirmed by high resolution mass spectrometry.

Enolate Crossover High Resolution Mass Spectrometry Data

(2*S*)-[2-Allyl-1,1-dioxo-thietan-2-yl]-phenyl-methanone (18a)

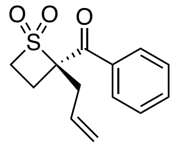

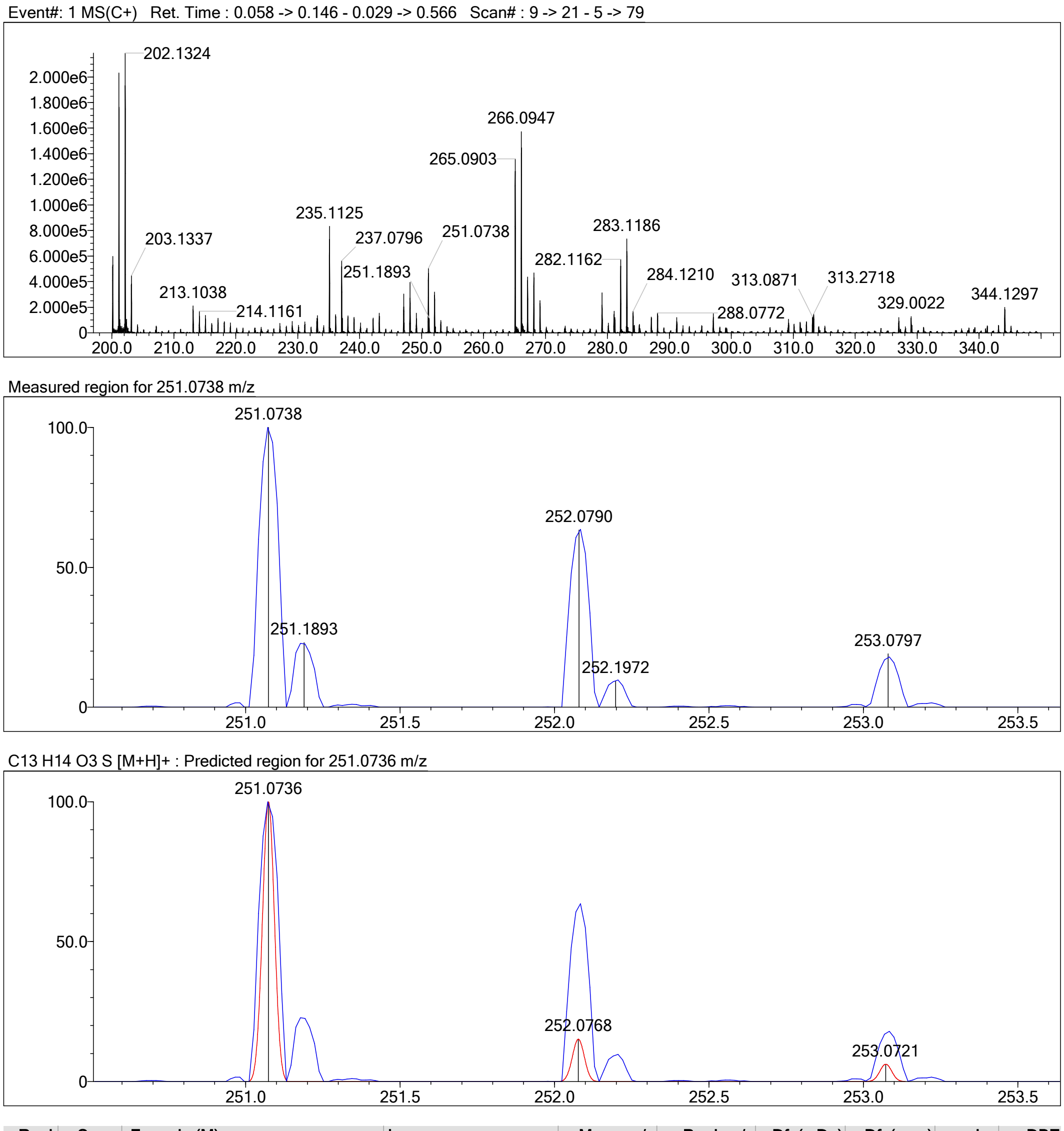

Rank	Score	Formula (M)	Ion	Meas. m/z	Pred. m/z	Df. (mDa)	Df. (ppm)	Iso	DBE
1	41.87	C13 H14 O3 S	[M+H]+	251.0738	251.0736	0.2	0.80	41.87	7.0

(2*S*)-[2-(3-[D]-Allyl)-1,1-dioxo-thietan-2-yl]-phenyl-methanone ([D]-18a)

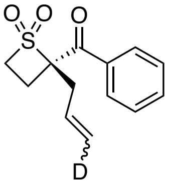

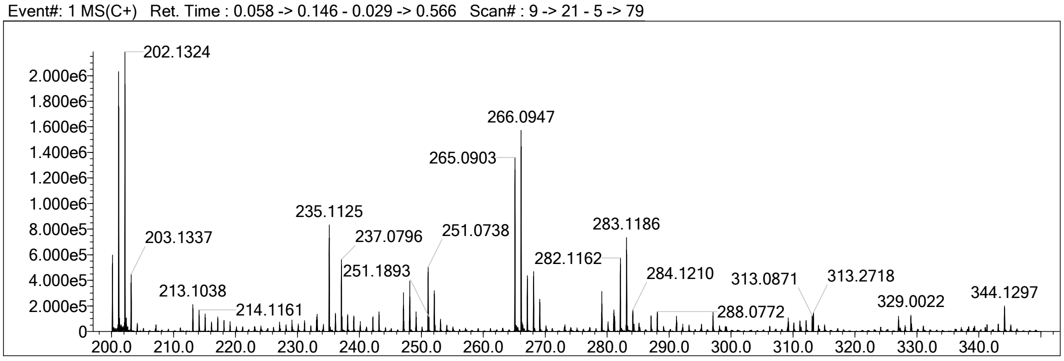

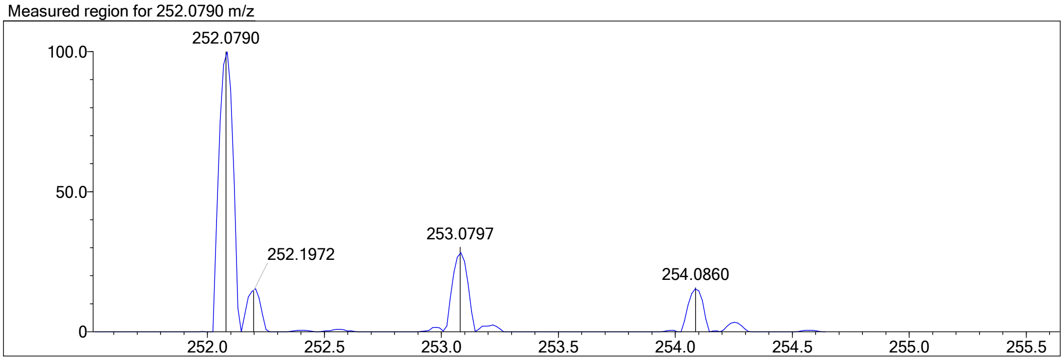

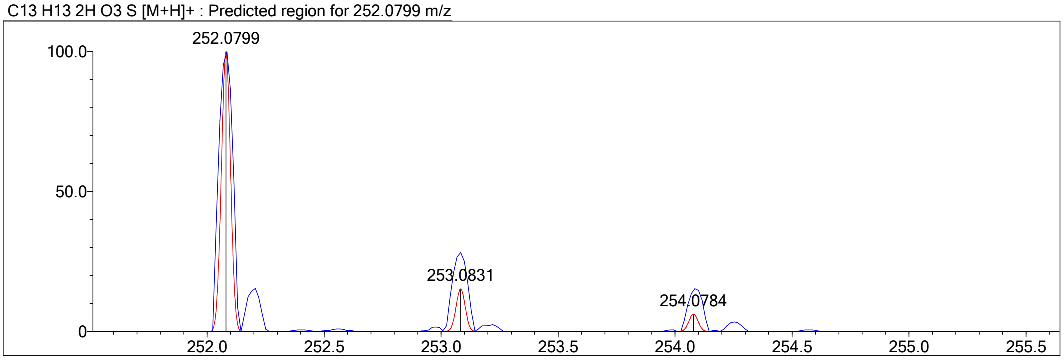

Rank	Score	Formula (M)	Ion	Meas. m/z	Pred. m/z	Df. (mDa)	Df. (ppm)	Iso	DBE
1	49.83	C13 H13 2H O3 S	[M+H]+	252.0790	252.0799	-0.9	-3.57	53.25	7.0

(2*S*)-[2-Allyl-1,1-dioxo-thietan-2-yl]-(*p*-tolyl)methanone (18c)

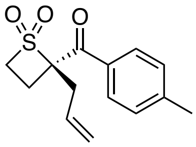

Event#: 1 MS(C+) Ret. Time : 0.058 -> 0.146 - 0.029 -> 0.566 Scan# : 9 -> 21 - 5 -> 79

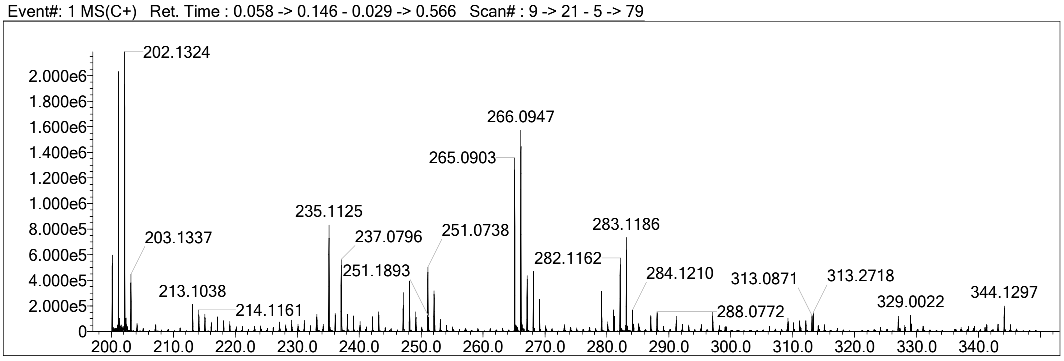

Measured region for 265.0903 m/z

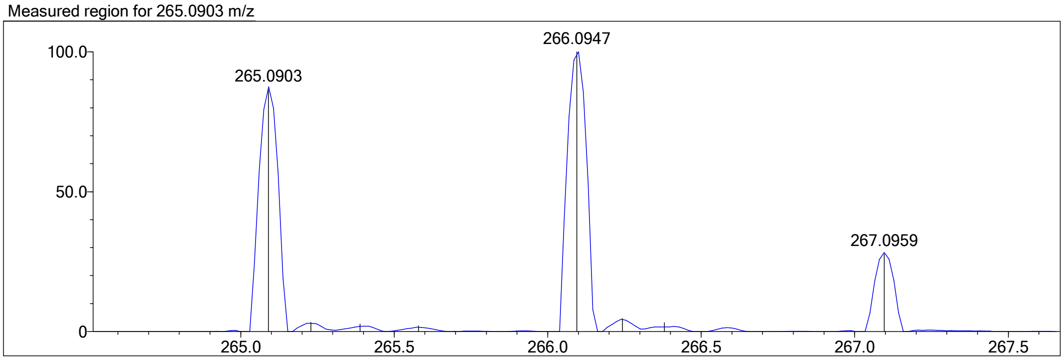

C14 H16 O3 S [M+H]+ : Predicted region for 265.0893 m/z

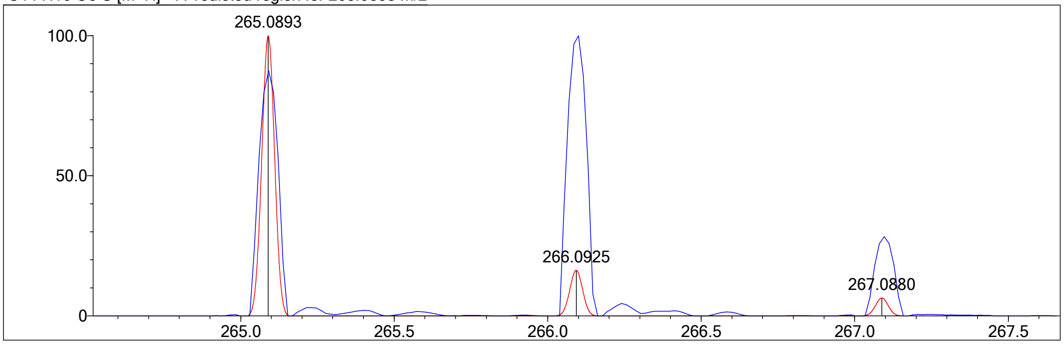

Rank	Score	Formula (M)	Ion	Meas. m/z	Pred. m/z	Df. (mDa)	Df. (ppm)	Iso	DBE
1	38.40	C14 H16 O3 S	[M+H]+	265.0903	265.0893	1.0	3.77	41.26	7.0

(2*S*)-[2-(3-[D]-Allyl)-1,1-dioxo-thietan-2-yl]-(*p*-tolyl)methanone ([D]-18c)

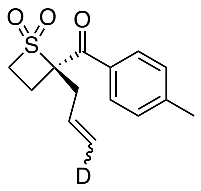

Event#: 1 MS(C+) Ret. Time : 0.058 -> 0.146 - 0.029 -> 0.566 Scan# : 9 -> 21 - 5 -> 79

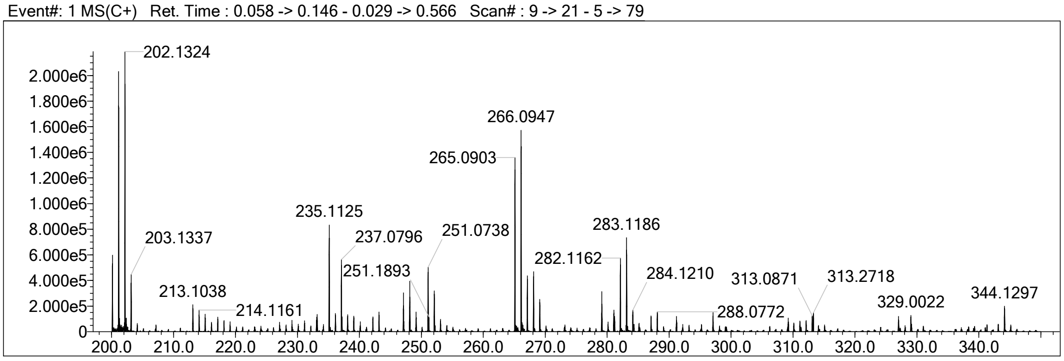

Measured region for 266.0947 m/z

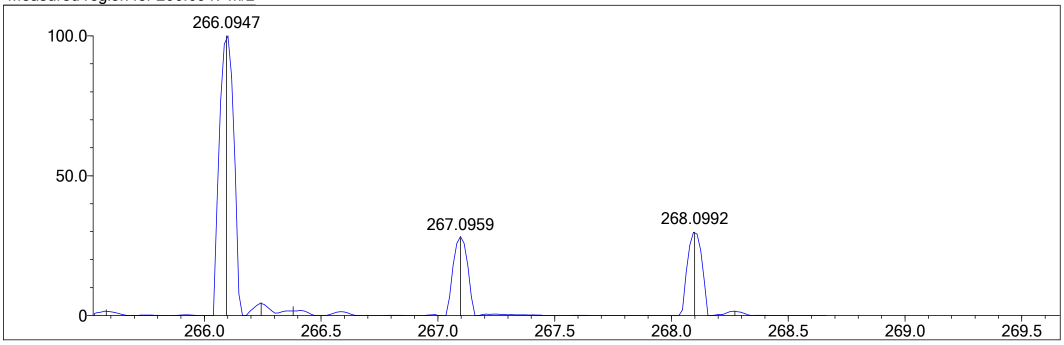

C14 H15 2H O3 S [M+H]+ : Predicted region for 266.0956 m/z

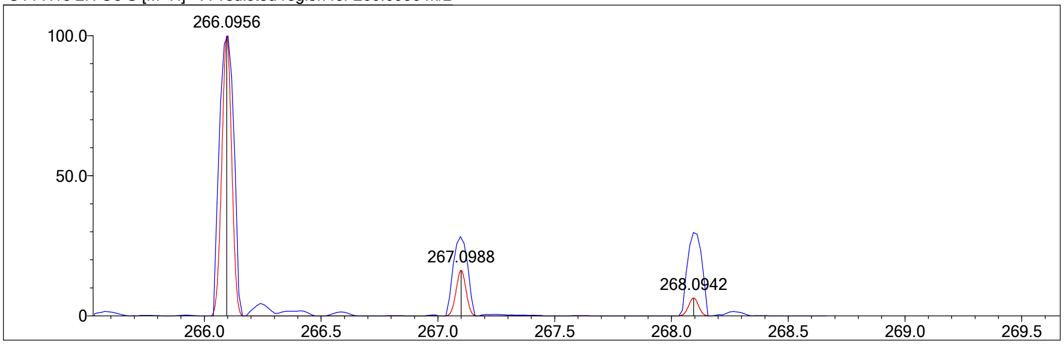

Rank	Score	Formula (M)	Ion	Meas. m/z	Pred. m/z	Df. (mDa)	Df. (ppm)	Iso	DBE
2	45.06	C14 H15 2H O3 S	[M+H]+	266.0947	266.0956	-0.9	-3.38	47.91	7.0

3.3 Water Tolerance Study

A flask containing $Pd_2(dba)_3$ (2 mg, 0.003 mmol), (*S*,*S*)-ANDEN Trost Ligand (**L4**) (6 mg, 0.007 mmol) and 1,4-dioxane (1.5 mL) was stirred at room temperature for 5 minutes. H_2O (X equiv) was added, followed by a solution of **16a** (30 mg, 0.10 mmol) in 1,4-dioxane (2.5 mL). The reaction was stirred at room temperature for 18 h. The mixture was concentrated under reduced pressure. Purification by flash chromatography [SiO_2; 5:1 hexane:EtOAc] afforded **18a**.

Table 2. Water Tolerance Study

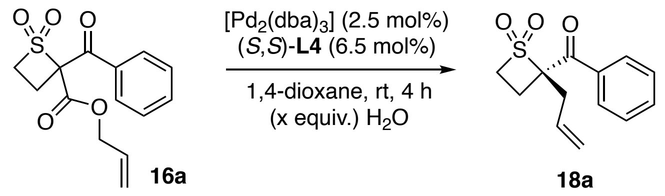

Entry	H_2O (equiv.)	Yield (%)[a]	ee (%)[b]
1	0	78	84
2	0.5	78	84
3	1	82	81
4	5	82	80
5	10	78	76
6	20	78	73

[a] Isolated yield; [b] Determined by chiral HPLC

3.4 Stereochemical Labelling

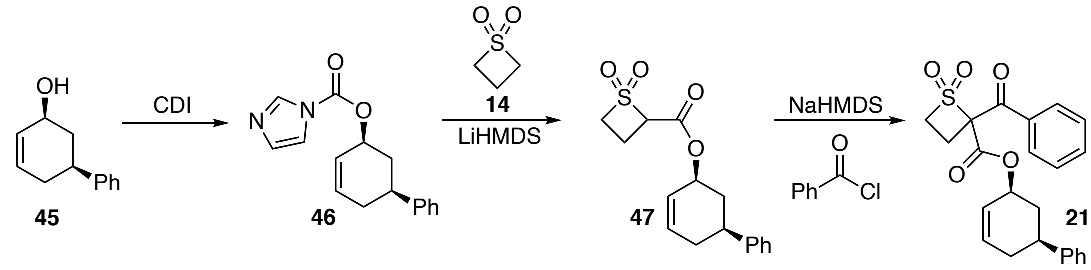

45 was prepared by a literature procedure.[4]

(*cis*-5-Phenylcyclohex-2-en-1-yl) imidazole-1-carboxylate (46)

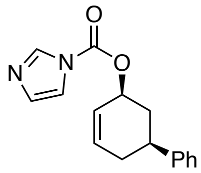

46

45 (400 mg, 2.30 mmol) was dissolved in CH_2Cl_2 (20 mL). 1,1'-Carbonyldiimidazole (932 mg, 5.75 mmol) was added in portions. The suspension was stirred at room temperature for 2 h. The reaction mixture was concentrated under reduced pressure. Purification by flash column chromatography [3:1 Petrol:EtOAc] gave **46** (536 mg, 87%) as a colourless solid. $\boldsymbol{R}_\mathbf{f}$ = 0.24 [3:1 petrol:EtOAc]. **mp**: 104– 106 ºC.

^{1}H NMR: (400 MHz, $CDCl_3$) δ 8.10 (1H, br s), 7.40 (1H, t, *J* = 1.2 Hz), 7.34 – 7.30 (2H, m), 7.26 – 7.22 (3H, m), 7.07 (1H, dd, *J* = 2, 0.8 Hz), 6.11 – 6.05 (1H, m), 5.84 – 5.79 (1H, m), 5.78 – 5.72 (1H, m), 3.05 (1H, dddd, *J* = 12.9, 10.4, 5.1, 2.6 Hz), 2.52 – 2.36 (2H, m), 2.35 – 2.23 (1H, m), 2.05 (1H, td, *J* = 12.6, 9.8 Hz).

^{13}C NMR: (100 MHz, $CDCl_3$) δ 148.4, 144.5, 137.2, 132.4, 130.2, 128.8, 126.9, 126.9, 125.3, 117.1, 75.5, 38.7, 35.0, 33.2.

HRMS: Molecular ion not observed for $C_{16}H_{16}N_2O_2$.

IR: ν_{max} (neat/cm^{-1}): 3157, 2950, 2931, 1741.

(*cis*-5-Phenylcyclohex-2-en-1-yl) 1,1-dioxothietane-2-carboxylate (47)

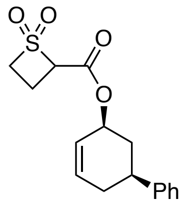

47

A solution of LiHMDS (1 M in THF, 2.94 mL, 2.94 mmol.) in THF (10 mL) was cooled to –78 ºC. A solution of **14** (148 mg, 1.40 mmol) in THF (3 mL) was added and the reaction mixture was stirred at –78 ºC for 30 minutes. A solution of **46** (400 mg, 1.50 mmol) in THF (3 mL) was added dropwise. The mixture was stirred at –78 ºC for 3 h, then allowed to warm to room temperature and stirred at ambient temperature for a further 1 h. The reaction was quenched with aq. HCl (1 N, 0.5 mL) and diluted with water (5 mL). The mixture was extracted with EtOAc (3 x 15 mL), washed with brine (10 mL), dried ($MgSO_4$) and concentrated under reduced pressure. Purification by flash column chromatography [SiO_2; 10:1 hexane:EtOAc] afforded **47** (193 mg, 45%) as a colourless oil. $\boldsymbol{R}_f$ = 0.37 [2:1 petrol:EtOAc].

^{1}H NMR: (400 MHz, $CDCl_3$) δ 7.34 – 7.28 (2H, m), 7.24 – 7.19 (3H, m), 6.02 – 5.94 (1H, m), 5.78 – 5.67 (2H, m), 5.10 – 5.01 (1H, m), 4.27 – 4.12 (2H, m), 3.04 – 2.92 (1H, m), 2.64 – 2.53 (1H, m), 2.45 – 2.14 (4H, m), 1.99 – 1.88 (1H, m).

^{13}C NMR: (100 MHz, $CDCl_3$) δ 163.9, 144.8, 131.6, 131.2, 128.5, 126.7, 126.2, 80.0, 74.1, 65.4, 39.1, 35.0, 33.5, 9.7.

HRMS: (APCI-TOF) m/z: $[M+Na]^+$ calcd for $C_{16}H_{18}O_4SNa$ 329.0818; found 329.0826.

IR: ν_{max} (neat/cm^{-1}): 3030, 2963, 2927, 1731, 1326, 1190.

(*cis*-5-Phenylcyclohex-2-en-1-yl) 2-benzoyl-1,1-dioxo-thietane-2-carboxylate (21)

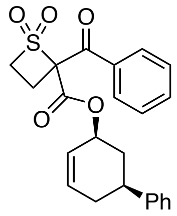

21

A solution of NaHMDS (1 M in THF, 0.4 mL, 0.40 mmol) in THF (10 mL) was cooled to 0 ºC. A solution of **47** (100 mg, 0.33 mmol) in THF (2 mL) was added and the reaction mixture was stirred at 0 ºC for 15 minutes. Benzoyl chloride (56 µL, 0.40 mmol) was added dropwise and the mixture stirred at 0 °C for 3 h. The reaction was quenched with aq. HCl (1 N, 1 mL), allowed to warm to room temperature and diluted with water (10 mL). The mixture was extracted with EtOAc (3 x 20 mL), washed with brine (10 mL), dried ($MgSO_4$) and concentrated under reduced pressure. Purification by flash column chromatography [SiO_2; 7:1 hexane:EtOAc] afforded **21** (82 mg, 60%) as a colourless oil. $\boldsymbol{R}_{\mathbf{f}}$ = 0.58 [2:1 petrol:EtOAc].

^{1}H NMR: (400 MHz, $CDCl_3$, mixture of diastereoisomers) δ 7.71 – 7.42 (5H, m), 7.36 – 7.13 (5H, m), 5.95 – 5.87 (1H, m), 5.71 – 5.63 (1H, m), 5.60 – 5.52 (1H, m), 4.42 (1H, ddt, *J* = 12.4, 10.4, 4.0 Hz), 4.07 (1H, ddd, *J* = 12.4, 10.4, 3.6 Hz), 2.97 – 2.87 (2H, m), 2.75 (1H, ddd, *J* = 12.5, 10.4, 3.5 Hz), 2.35 – 2.09 (3H, m), 1.79 – 1.68 (1H, m).

^{13}C NMR: (100 MHz, $CDCl_3$, mixture of diastereoisomers) δ 187.9, 187.8, 164.5, 164.4, 144.6, 144.6, 134.9, 134.9, 134.4, 134.4, 134.2, 134.2, 129.7, 129.7, 129.1, 128.8, 128.8, 126.9, 126.8, 126.8, 126.7, 125.4, 125.3, 124.0, 97.4, 96.0, 74.9, 74.9, 63.7, 63.6, 62.0, 41.5, 39.7, 38.8, 38.8, 34.6, 34.6, 33.6, 33.2, 33.2, 31.6, 17.5, 17.4, 12.3.

HRMS: (APCI-TOF) m/z: $[M+Na]^+$ calcd for $C_{23}H_{22}O_5SNa$ 433.1080; found 433.1078.

IR: ν_{max} (neat/cm^{-1}): 3054, 3028, 2953, 1727, 1682, 1338, 1138.

A vial was charged with substrate **21** (42 mg, 0.1 mmol), $Pd_2(dba)_3$ (4.7 mg, 0.005 mmol), (*S*,*S*)-ANDEN Trost ligand **L4** (10.8 mg, 0.01 mmol) in 1,4-dioxane (3 mL). The reaction mixture was stirred at the stated temperature for the stated duration (entry 1–3). The mixture was concentrated under reduced pressure and ^{1}H NMR analysis indicated that either no reaction had taken place, or a mixture of starting material **21** and non-alkylated product **32** had formed.

Table 3. Stereochemical labelling

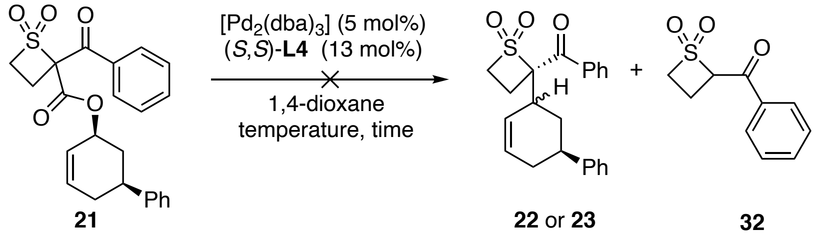

Entry	Conditions	Outcome[a]
1	RT, 24 h	**21** only
2	40 °C, 5 days	1:1.6 (**21**:**32**)
3	80 °C, 48 h	1:10.7 (**21**:**32**)

[a]Determined by ^{1}H NMR analysis of the crude product mixture

4. Rationale for Origins of Stereocontrol using (*S*,*S*)-ANDEN Phenyl Trost L4.

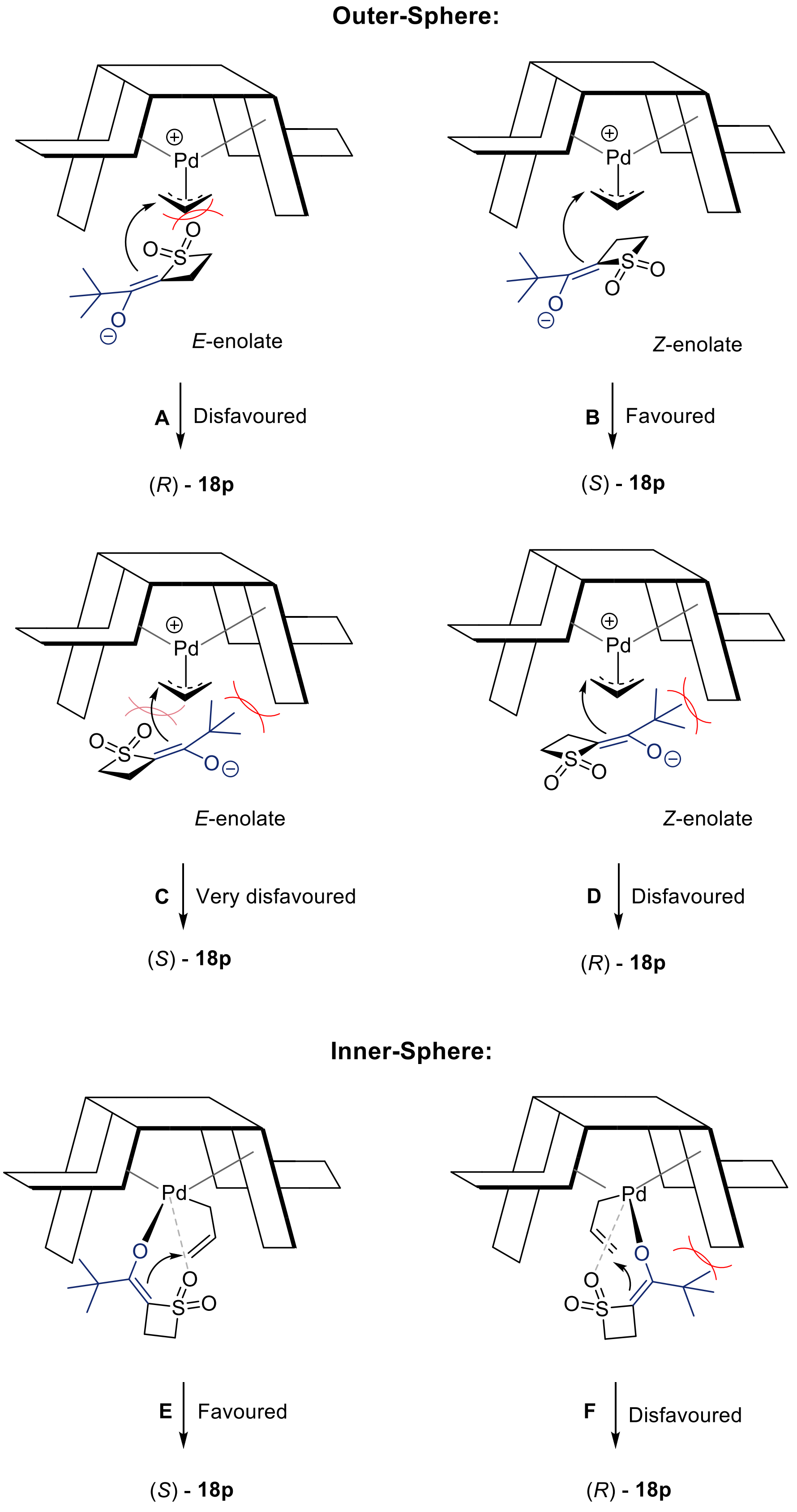

A rationale for the *E*/*Z* enolate equilibrium using the Trost model is shown above. In an outer-sphere alkylation mechanism, the enolate would prefer to approach the π-allyl system with the large *tert*-butyl substituent residing under the open 'flap'. As this leads to a steric clash between the ligand backbone and the sulfone when in *E*-geometry (pathway A), the *E*-enolate equilibrates to the *Z*-geometry, resulting in reduced steric interactions in the transition state of alkylation (pathway B), affording the major enantiomer (*S*)-**18p**. The approach from the opposite face of the enolate is disfavoured due to steric interactions of the *tert*-butyl group with the 'wall' of the ligand for both the *E*- and *Z*-enolates of **16p** (pathways C and D). As the enolate exclusively alkylates *via* pathway B, the (*S*)-enantiomer is formed with high enantioselectivity.

In principle, an inner-sphere alkylation mechanism could also be invoked when the enolate geometry is *Z* due to the stabilising interaction between the oxygen atom of the sulfone and the palladium centre.[5] Pathway E would be favoured over pathway F by minimising the steric clash between the *tert*-butyl substituent and the 'wall' of the ligand in the transition state, also leading to (*S*)-**18p** as the major enantiomer.

5. X-ray Crystallography Data

X-Ray Crystal Structure of 19a: CCDC 2099913

Table 4. Crystal data and structure refinement for **19a**

Empirical formula	$C_{13}H_{14}O_4S$
Formula weight	266.30
Temperature/K	100.01(10)
Crystal system	trigonal
Space group	$P3_1$
a/Å	13.07541(6)
b/Å	13.07541(6)
c/Å	6.55984(4)
α/°	90
β/°	90
γ/°	120
Volume/Å^3	971.259(11)
Z	3
ρ_{calc}g/cm^3	1.366
μ/mm^{-1}	2.275
F(000)	420.0
Crystal size/mm^3	0.406 × 0.176 × 0.093
Radiation	Cu Kα (λ = 1.54184)
2Θ range for data collection/°	7.808 to 152.984
Index ranges	$-16 \le h \le 16, -16 \le k \le 16, -8 \le l \le 7$
Reflections collected	52146
Independent reflections	2663 [R_{int} = 0.0371, R_{sigma} = 0.0110]
Data/restraints/parameters	2663/1/163
Goodness-of-fit on F^2	1.101
Final R indexes [I>=2σ (I)]	R_1 = 0.0244, wR_2 = 0.0673
Final R indexes [all data]	R_1 = 0.0244, wR_2 = 0.0673
Largest diff. peak/hole / e Å^{-3}	0.22/-0.21
Flack parameter	0.005(11)

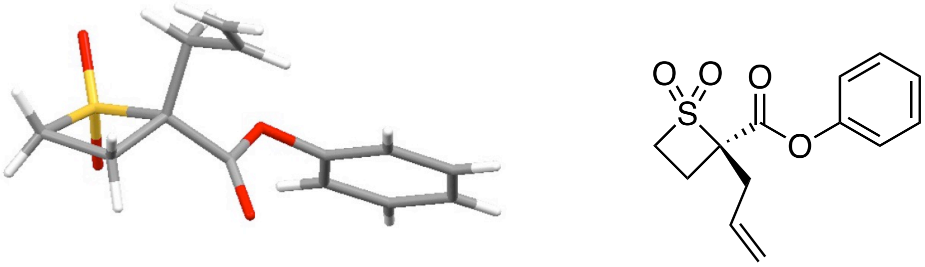

Figure 1. Crystal Structure of **19a**

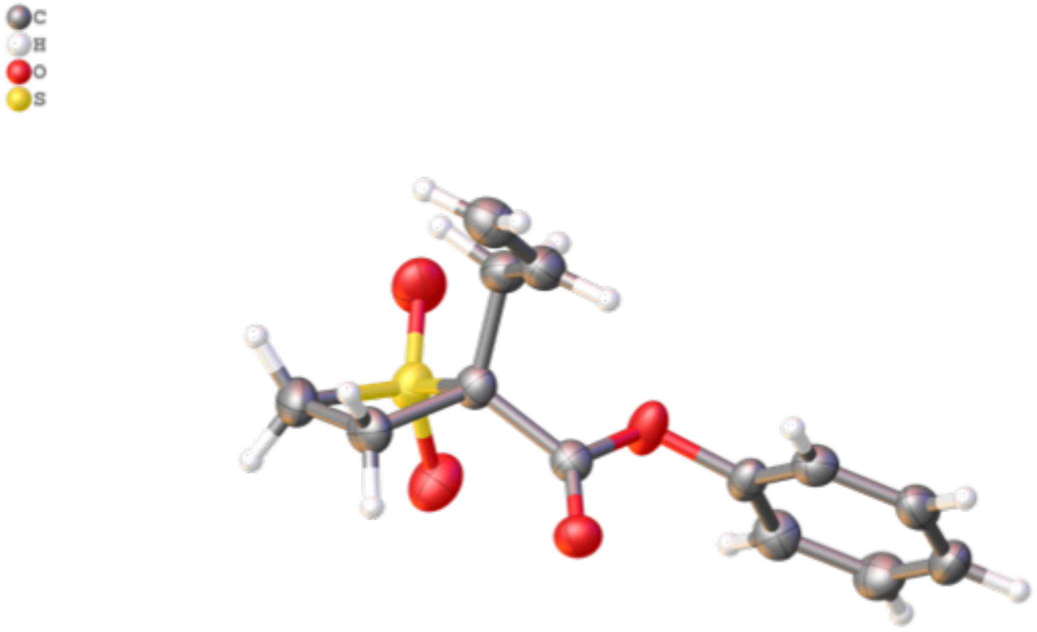

Figure 2: Structure **19a** with 70% probability thermal ellipsoids.

X-Ray Crystal Structure of 19b: CCDC **2099912**

Table 5. Crystal data and structure refinement for **19b**

Empirical formula	$C_{14}H_{16}O_5S$
Formula weight	296.33
Temperature/K	99.9(3)
Crystal system	monoclinic
Space group	$P2_1$
a/Å	6.29014(5)
b/Å	11.77074(11)
c/Å	9.67164(8)
α/°	90
β/°	102.3332(7)
γ/°	90
Volume/Å^3	699.558(10)
Z	2
ρ_{calc}g/cm^3	1.407
μ/mm^{-1}	2.218
F(000)	312.0
Crystal size/mm^3	0.1 × 0.1 × 0.08
Radiation	CuKα (λ = 1.54184)
2Θ range for data collection/°	9.36 to 152.9
Index ranges	$-7 \leq h \leq 7, -13 \leq k \leq 14, -12 \leq l \leq 12$
Reflections collected	29945
Independent reflections	2769 [R_{int} = 0.0327, R_{sigma} = 0.0108]
Data/restraints/parameters	2769/1/182
Goodness-of-fit on F^2	1.061
Final R indexes [I>=2σ (I)]	R_1 = 0.0249, wR_2 = 0.0682
Final R indexes [all data]	R_1 = 0.0249, wR_2 = 0.0682
Largest diff. peak/hole / e Å^{-3}	0.22/-0.28
Flack parameter	-0.004(8)

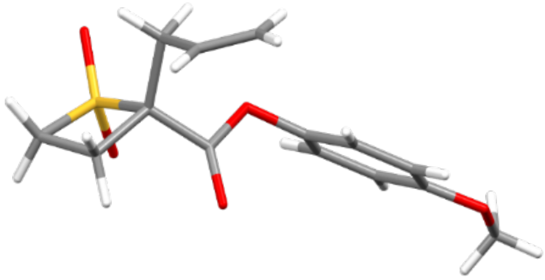

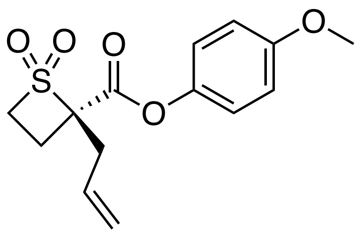

Figure 3. Crystal Structure of **19b**

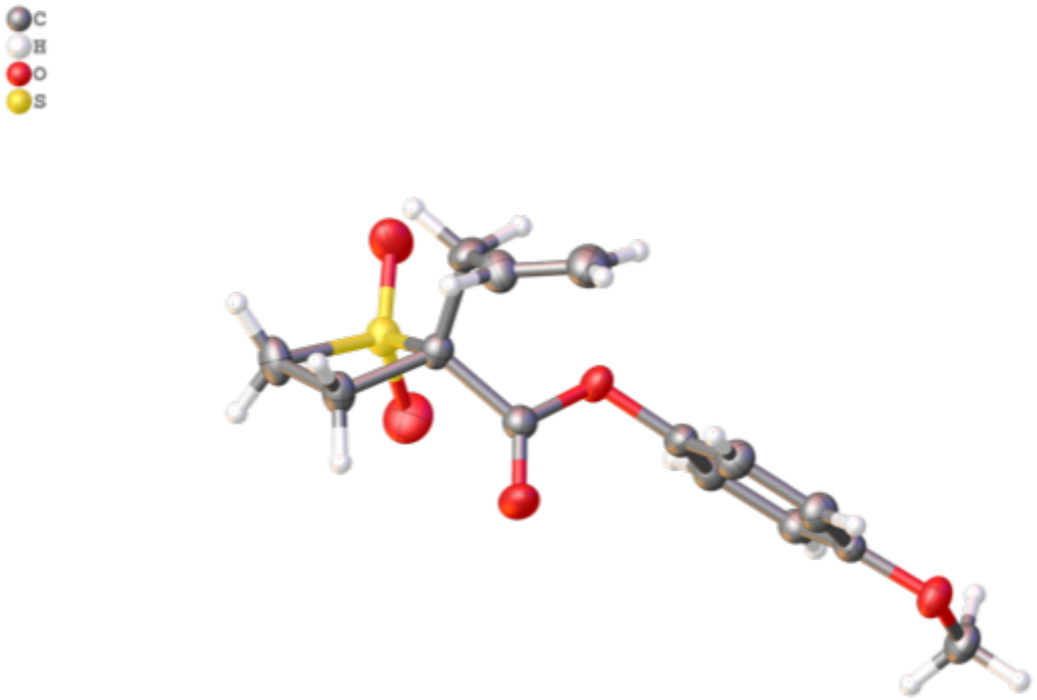

Figure 4: Structure **19b** with 70% probability thermal ellipsoids.

6. ^{1}H and ^{13}C NMR Spectra

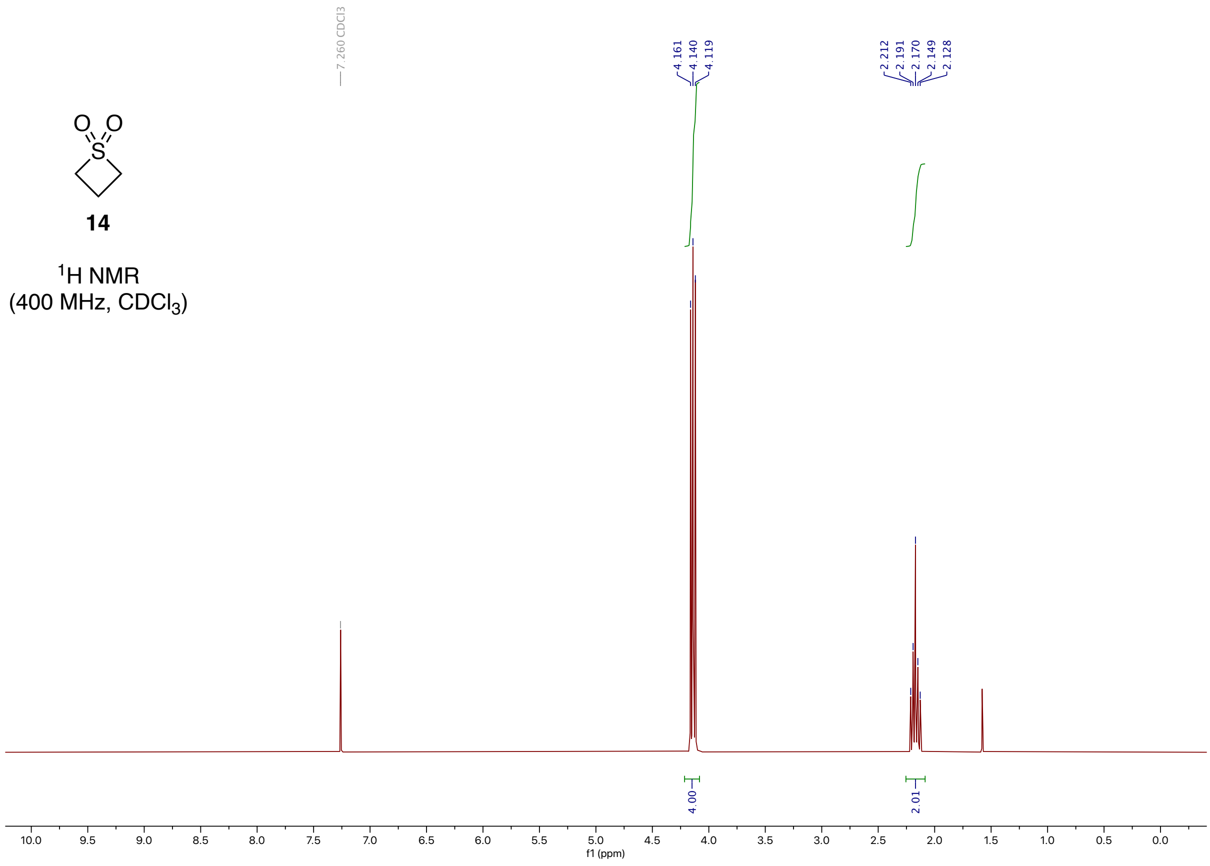

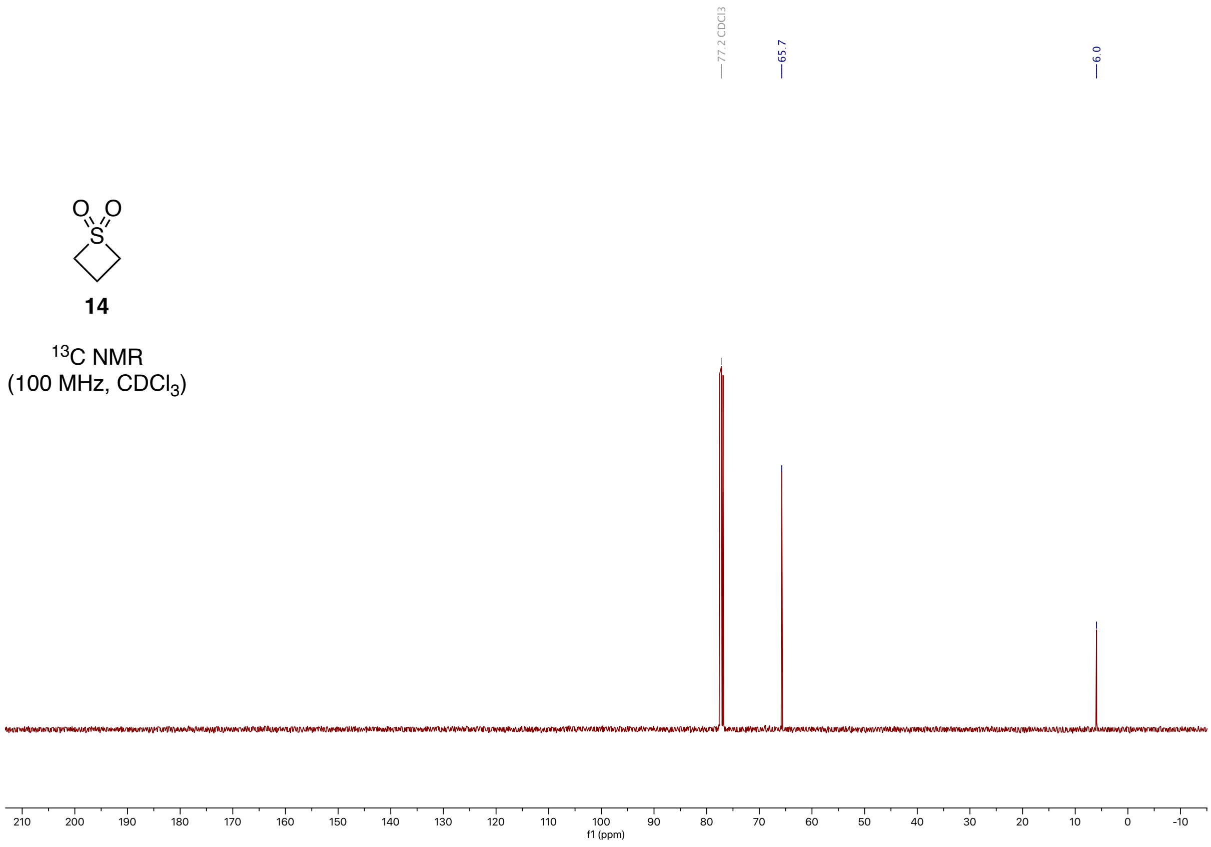

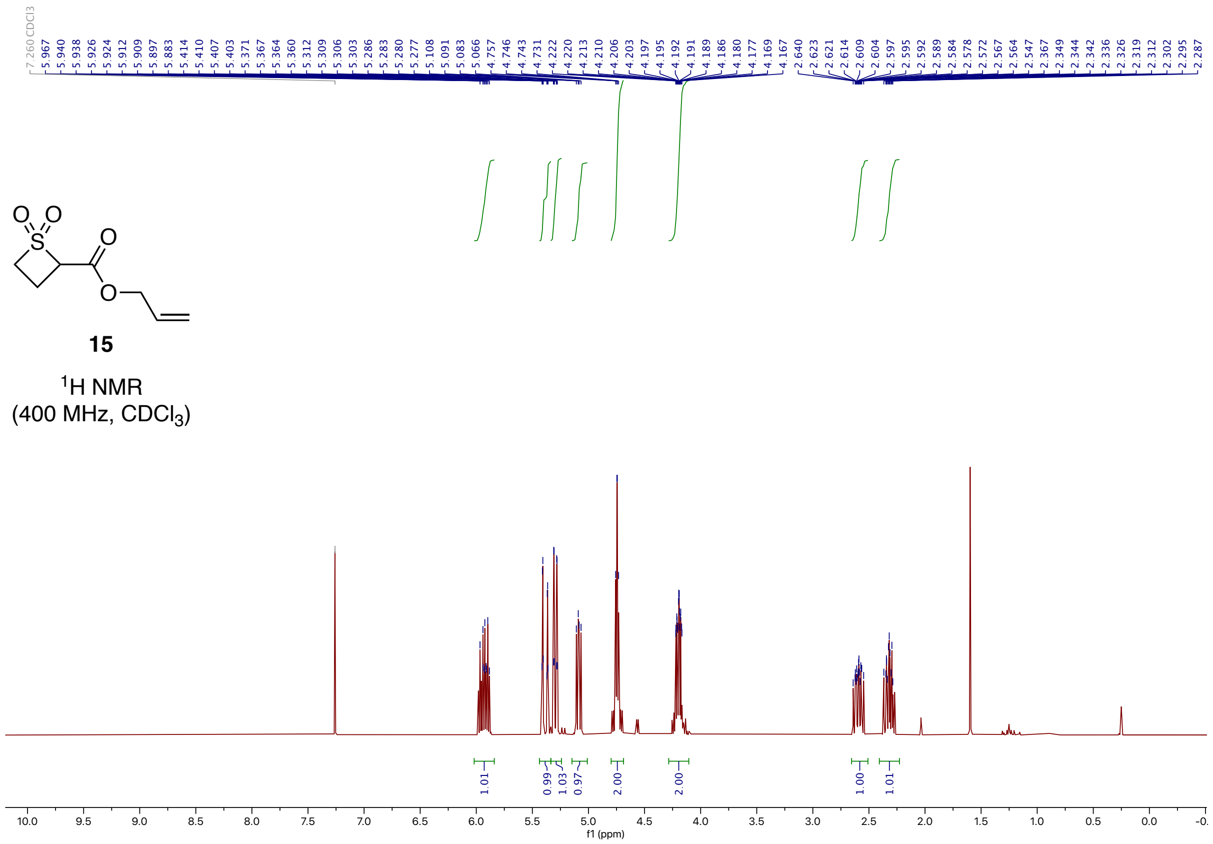

15

^{1}H NMR
(400 MHz, $CDCl_3$)

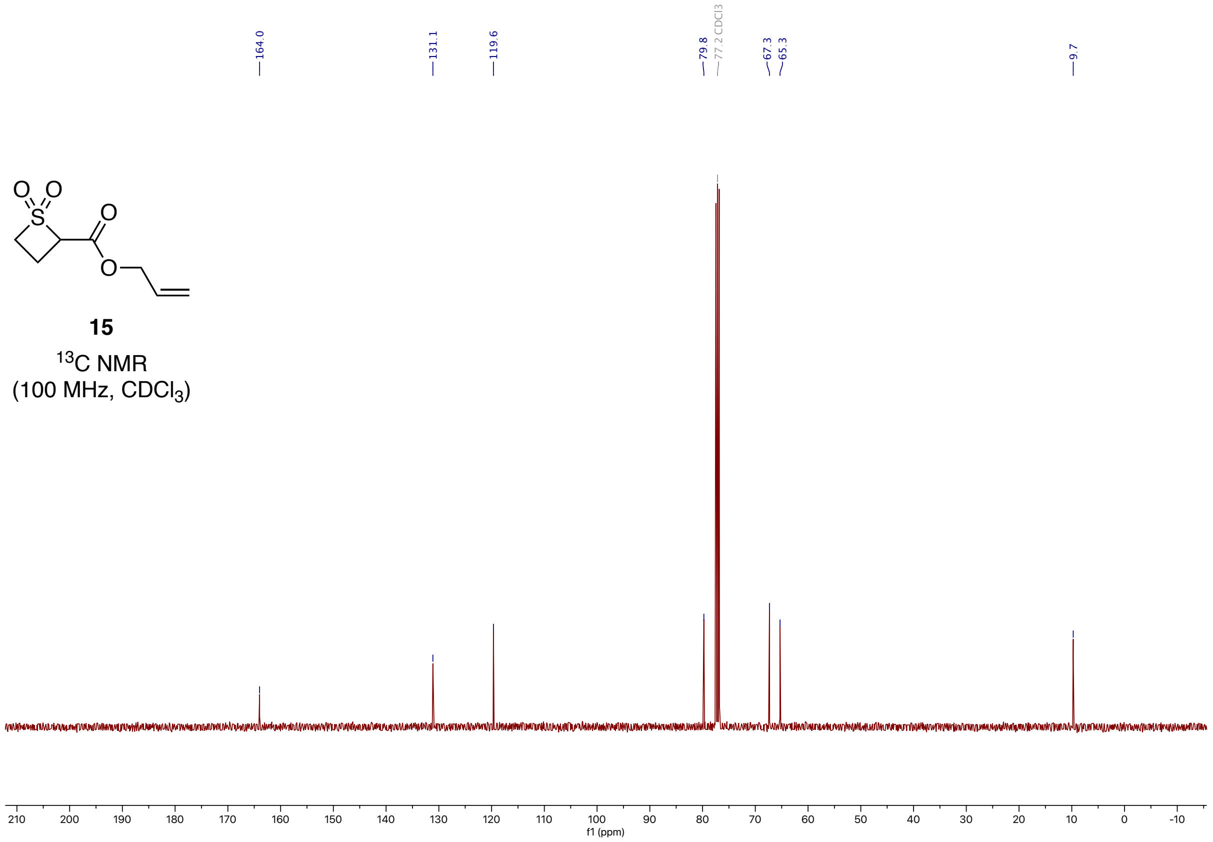

15

^{13}C NMR
(100 MHz, $CDCl_3$)

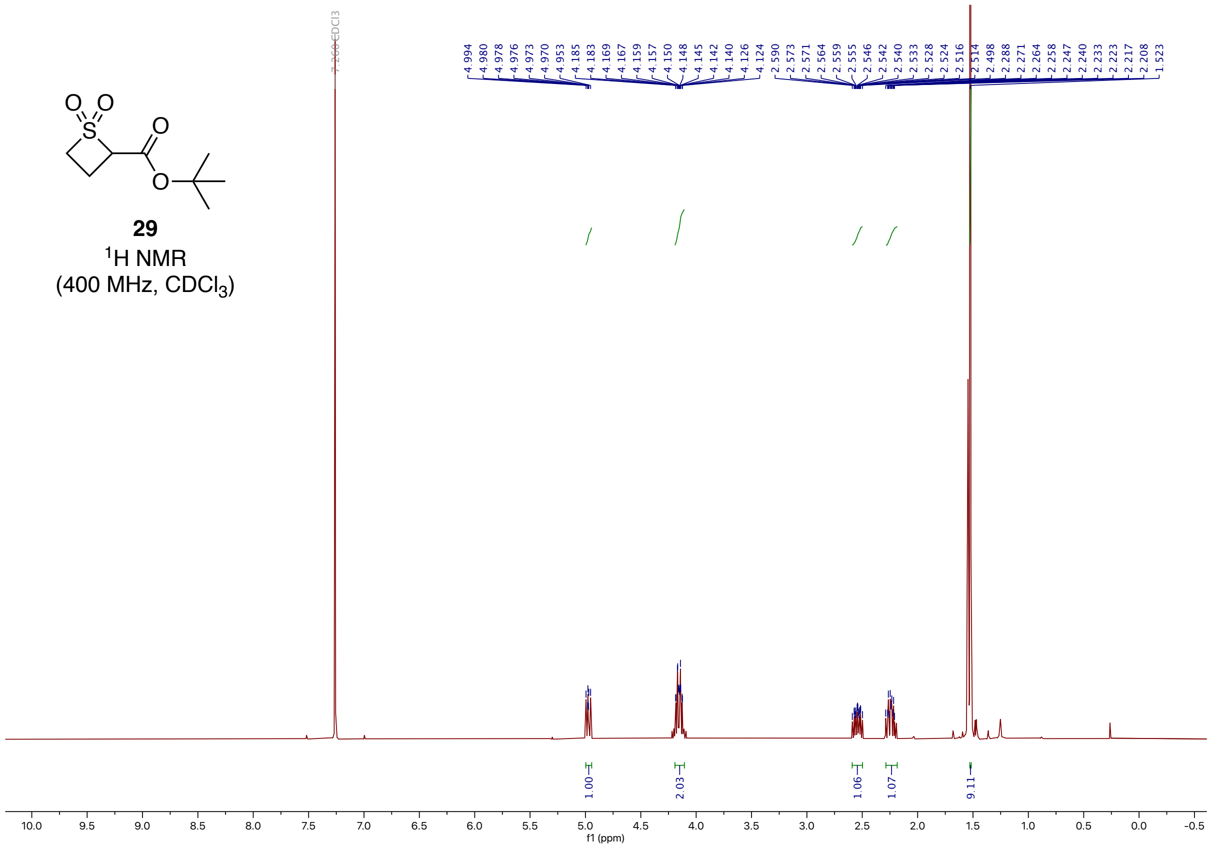
29
1H NMR
(400 MHz, CDCl3)
7.260 CDCl3
4.994
4.980
4.978
4.976
4.973
4.970
4.953
4.185
4.183
4.169
4.167
4.159
4.157
4.150
4.148
4.145
4.142
4.140
4.126
4.124
2.590
2.573
2.571
2.564
2.559
2.555
2.546
2.542
2.540
2.533
2.528
2.524
2.516
2.514
2.498
2.288
2.271
2.264
2.258
2.247
2.240
2.233
2.223
2.217
2.208
1.523
1.00
2.03
1.06
1.07
9.11
f1 (ppm)

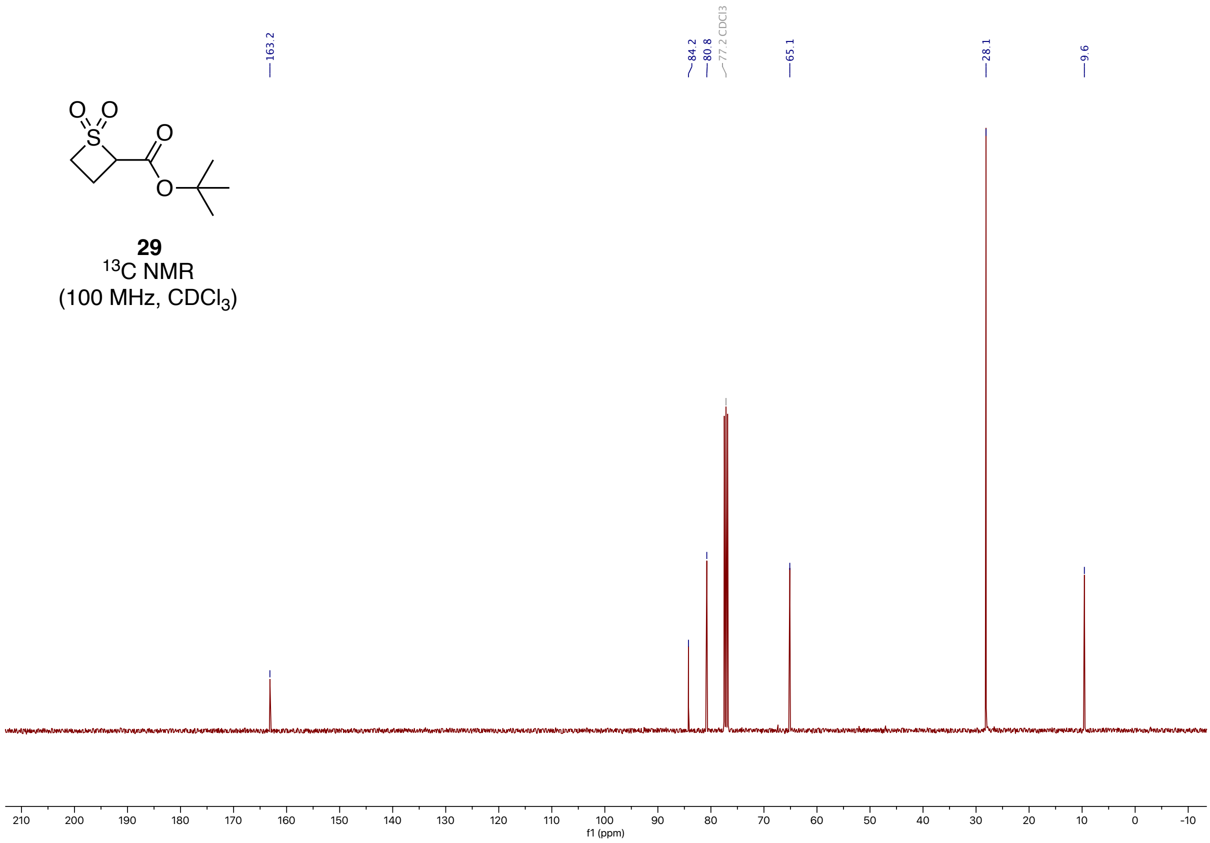
29
13C NMR
(100 MHz, CDCl3)
163.2
84.2
80.8
77.2 CDCl3
65.1
28.1
9.6
f1 (ppm)

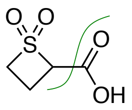

30
^{1}H NMR
(400 MHz, DMSO-d_6)

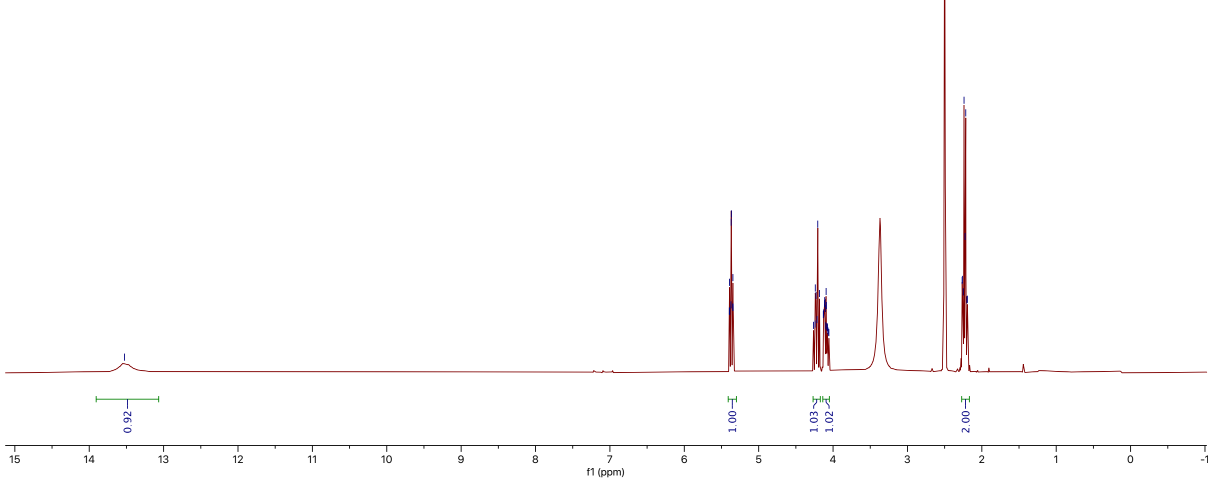

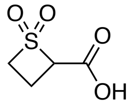

30
^{13}C NMR
(100 MHz, DMSO-d_6)

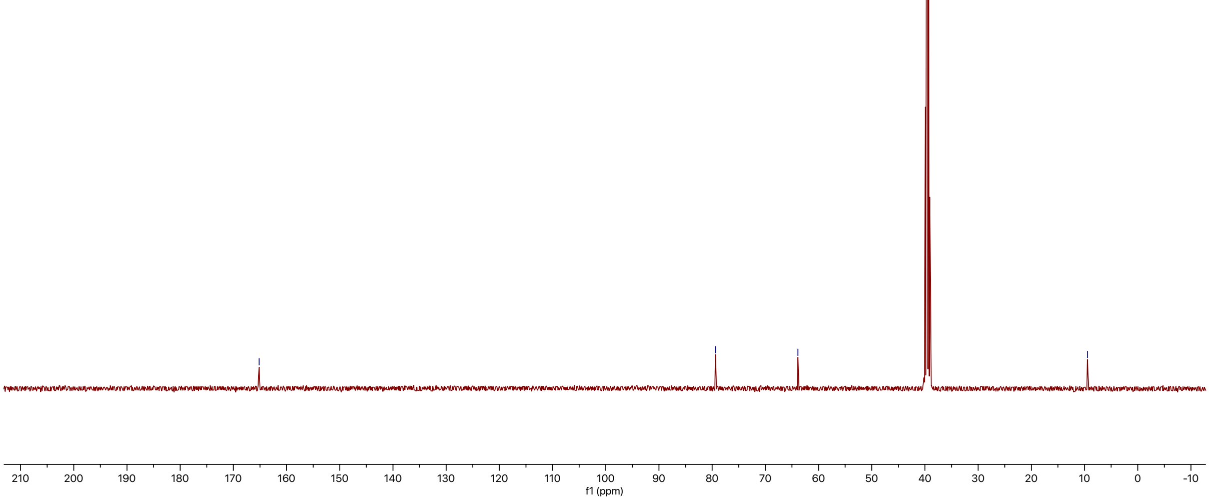

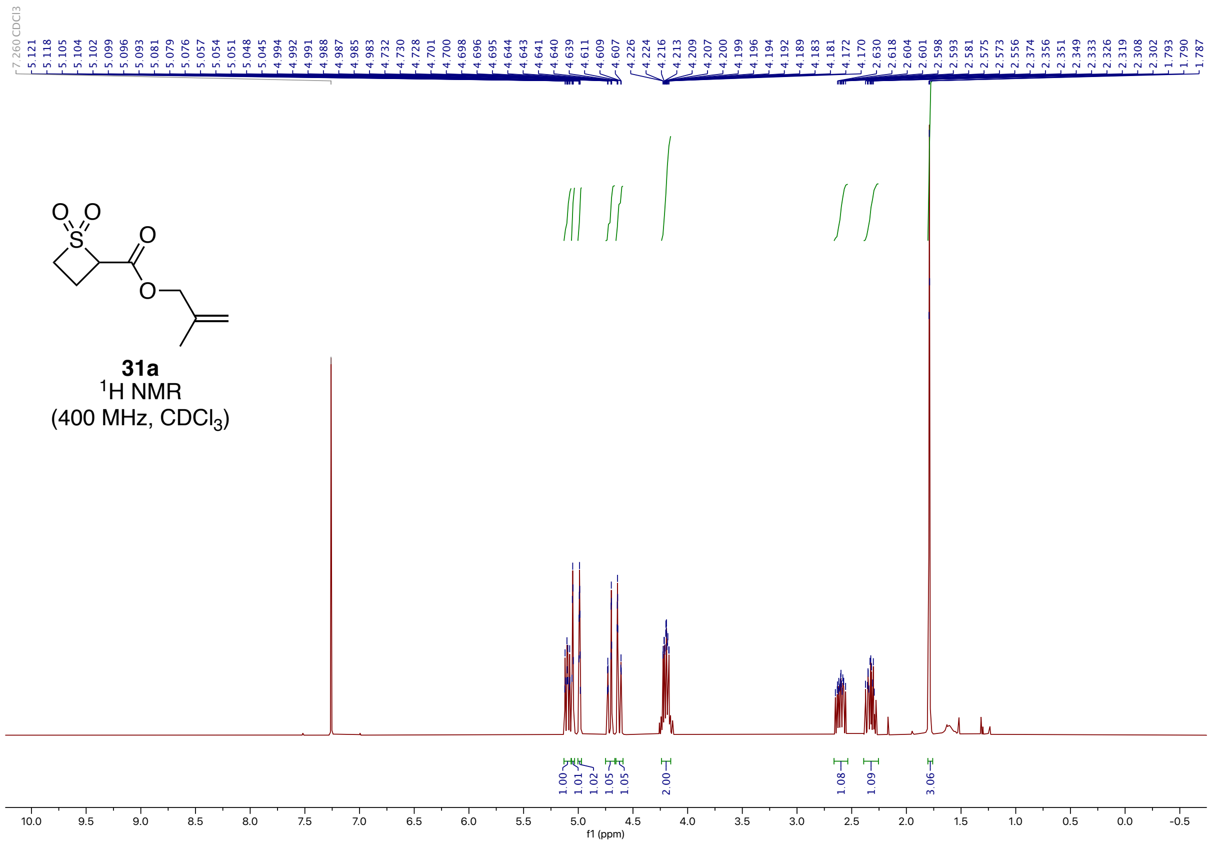
31a
1H NMR
(400 MHz, CDCl3)
f1 (ppm)

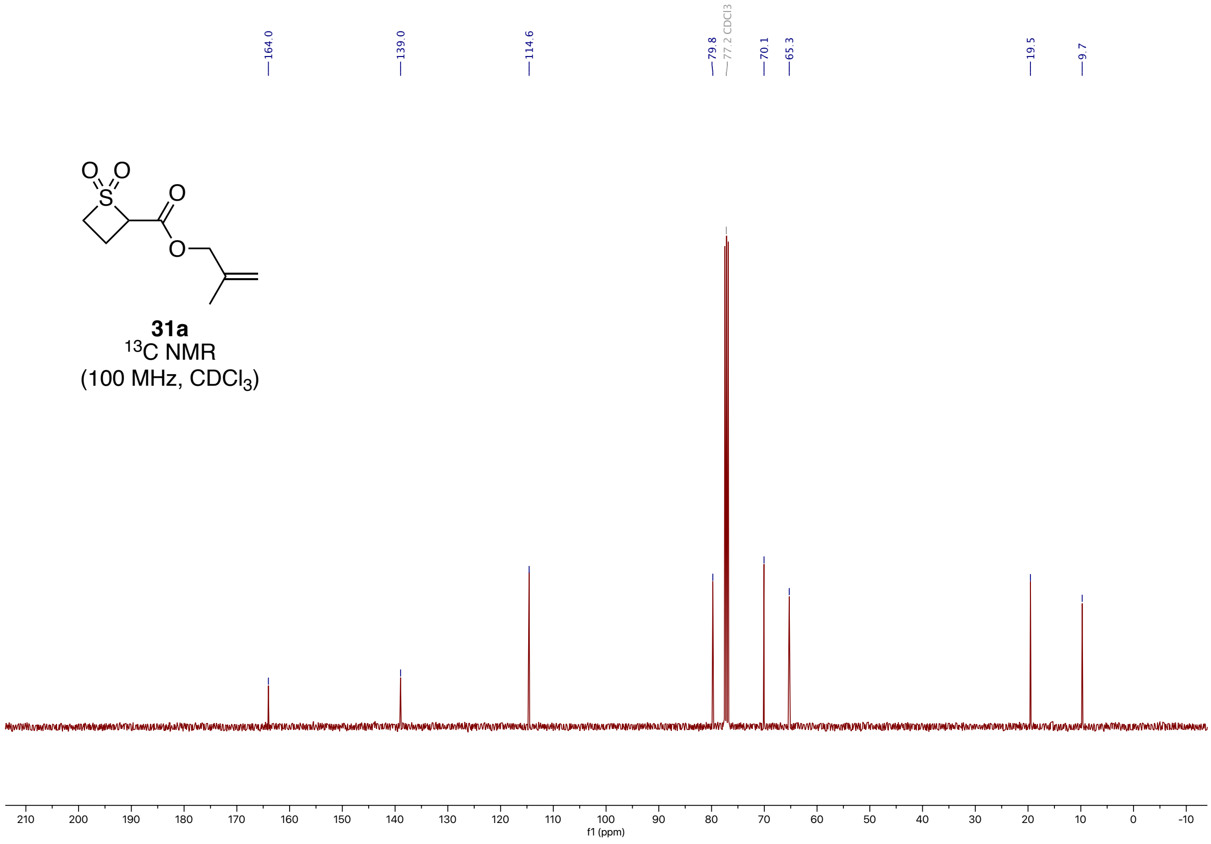
164.0
139.0
114.6
79.8
77.2 CDCl3
70.1
65.3
19.5
9.7
31a
13C NMR
(100 MHz, CDCl3)
f1 (ppm)

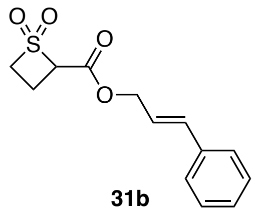

31b

^{1}H NMR

(400 MHz, $CDCl_3$)

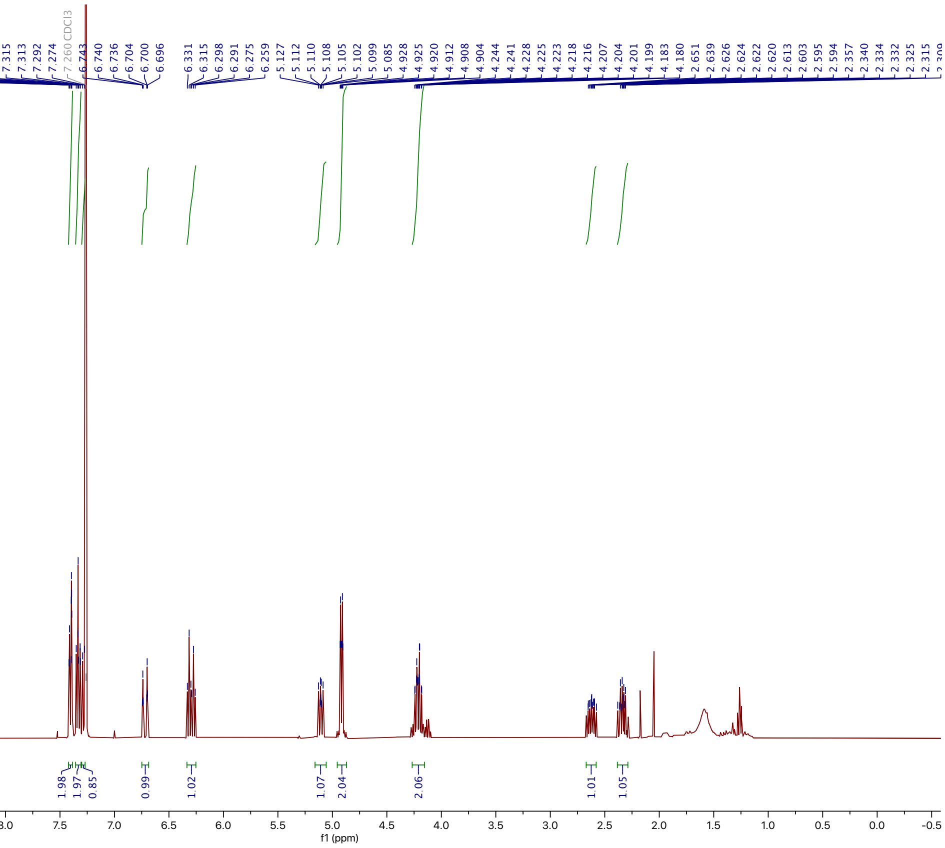

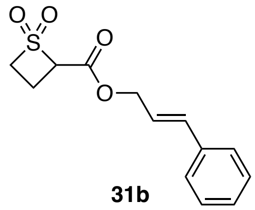

31b

^{13}C NMR

(100 MHz, $CDCl_3$)

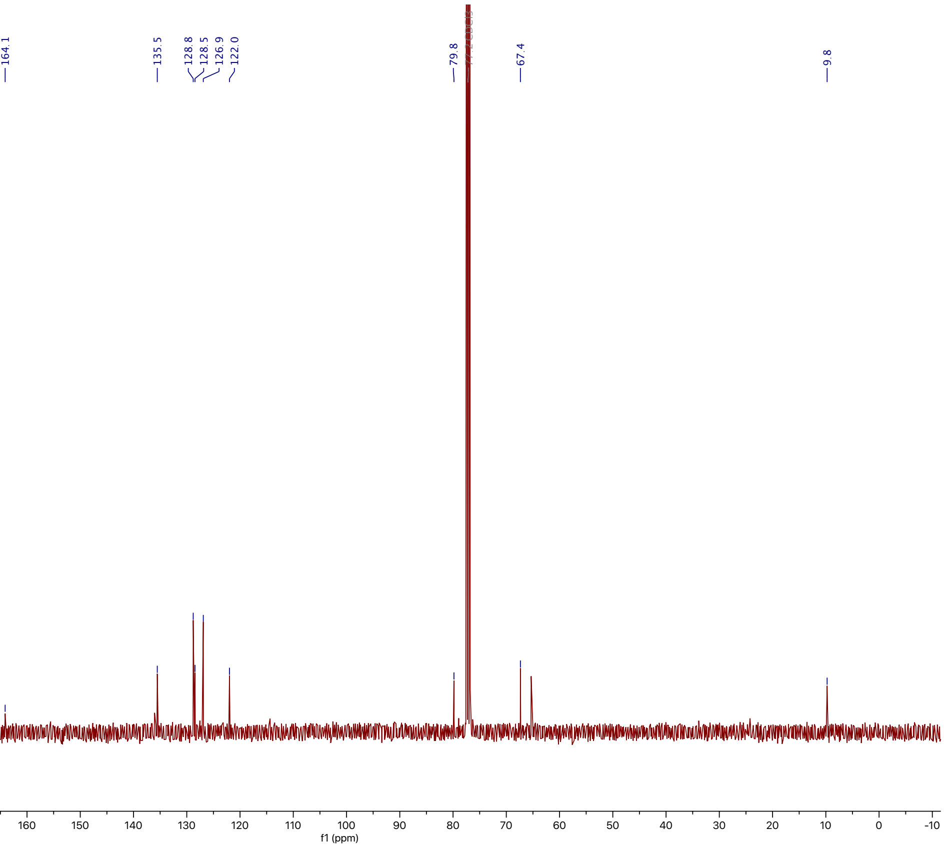

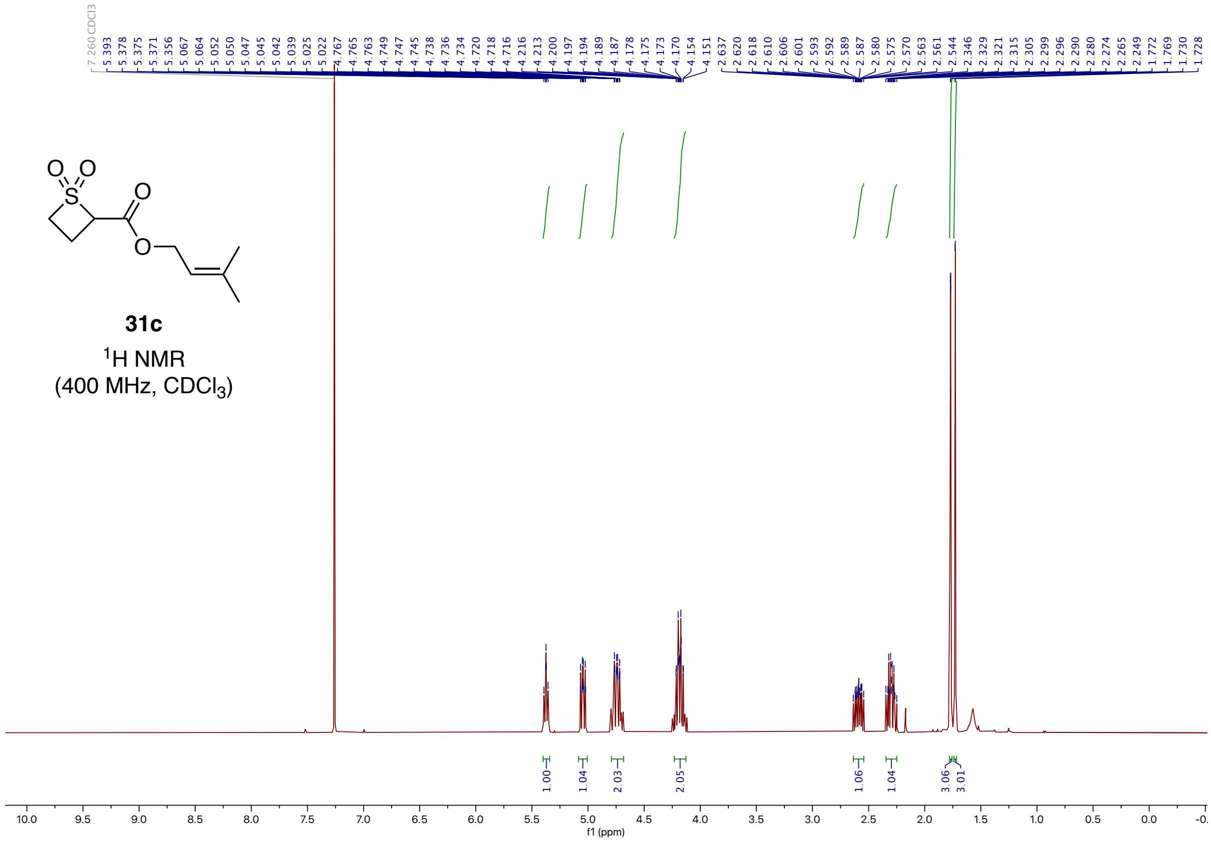

31c

^{1}H NMR

(400 MHz, $CDCl_3$)

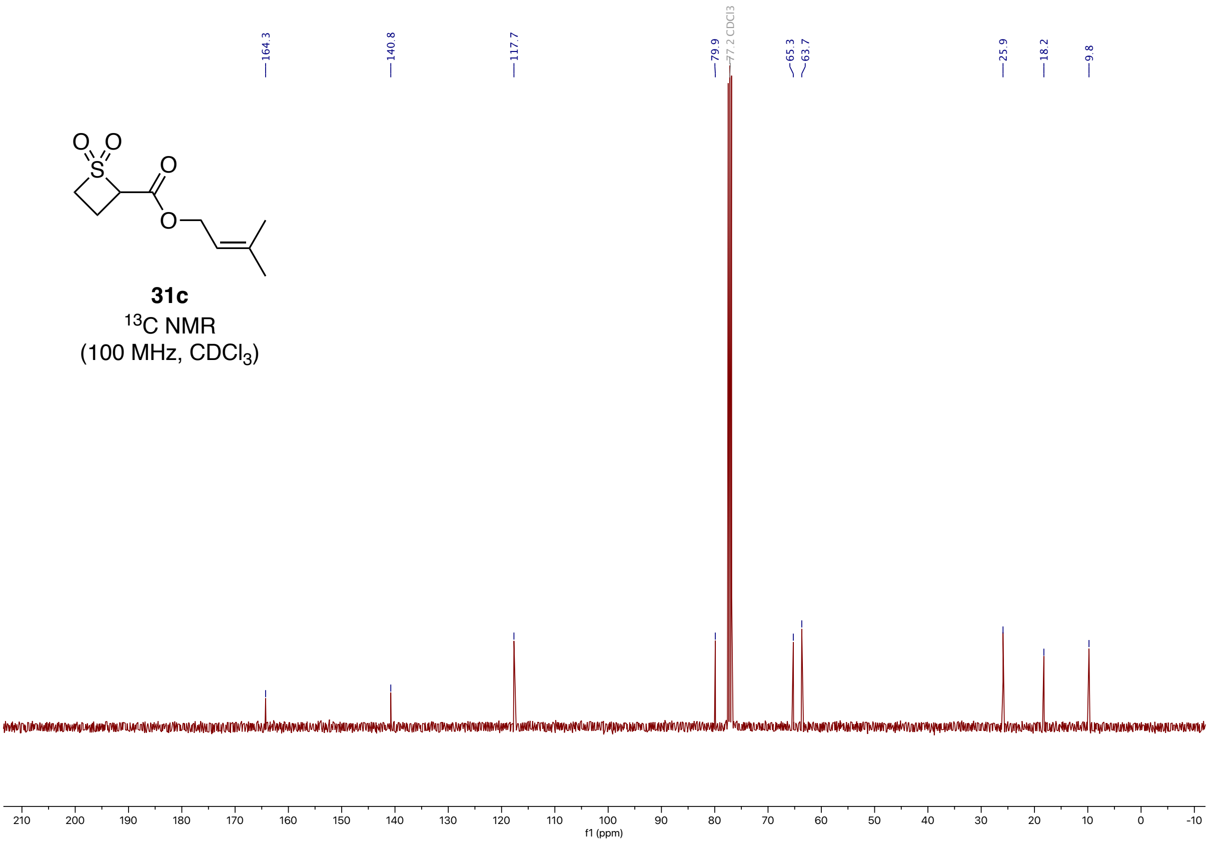

31c

^{13}C NMR

(100 MHz, $CDCl_3$)

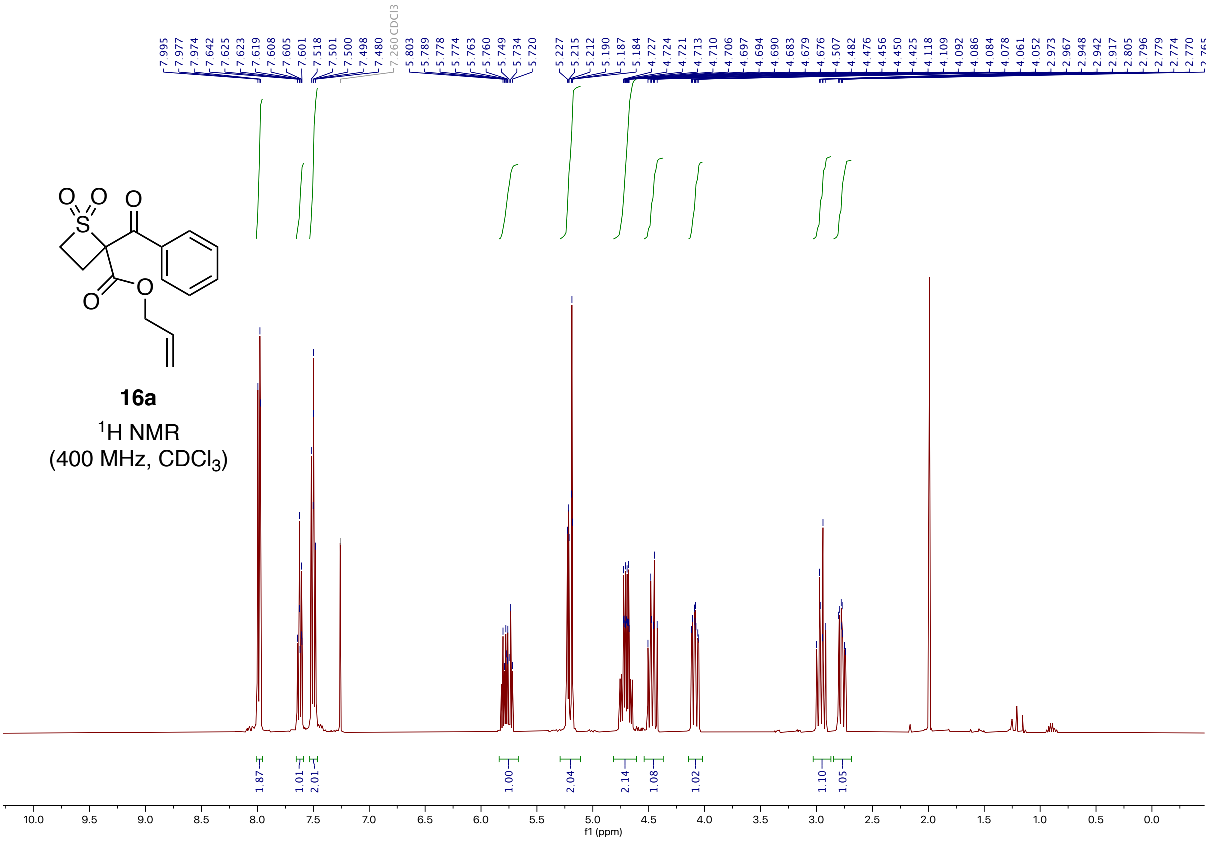
7.995
7.977
7.974
7.642
7.625
7.623
7.619
7.608
7.605
7.601
7.518
7.501
7.500
7.498
7.480
7.260 CDCl3
5.803
5.789
5.778
5.774
5.763
5.760
5.749
5.734
5.720
5.227
5.215
5.212
5.190
5.187
5.184
4.727
4.724
4.721
4.713
4.710
4.706
4.697
4.694
4.690
4.683
4.679
4.676
4.507
4.482
4.476
4.456
4.450
4.425
4.118
4.109
4.092
4.086
4.084
4.078
4.061
4.052
2.973
2.967
2.948
2.942
2.917
2.805
2.796
2.779
2.774
2.770
16a
^{1}H NMR
(400 MHz, $CDCl_3$)
1.87
1.01
2.01
1.00
2.04
2.14
1.08
1.02
1.10
1.05
f1 (ppm)

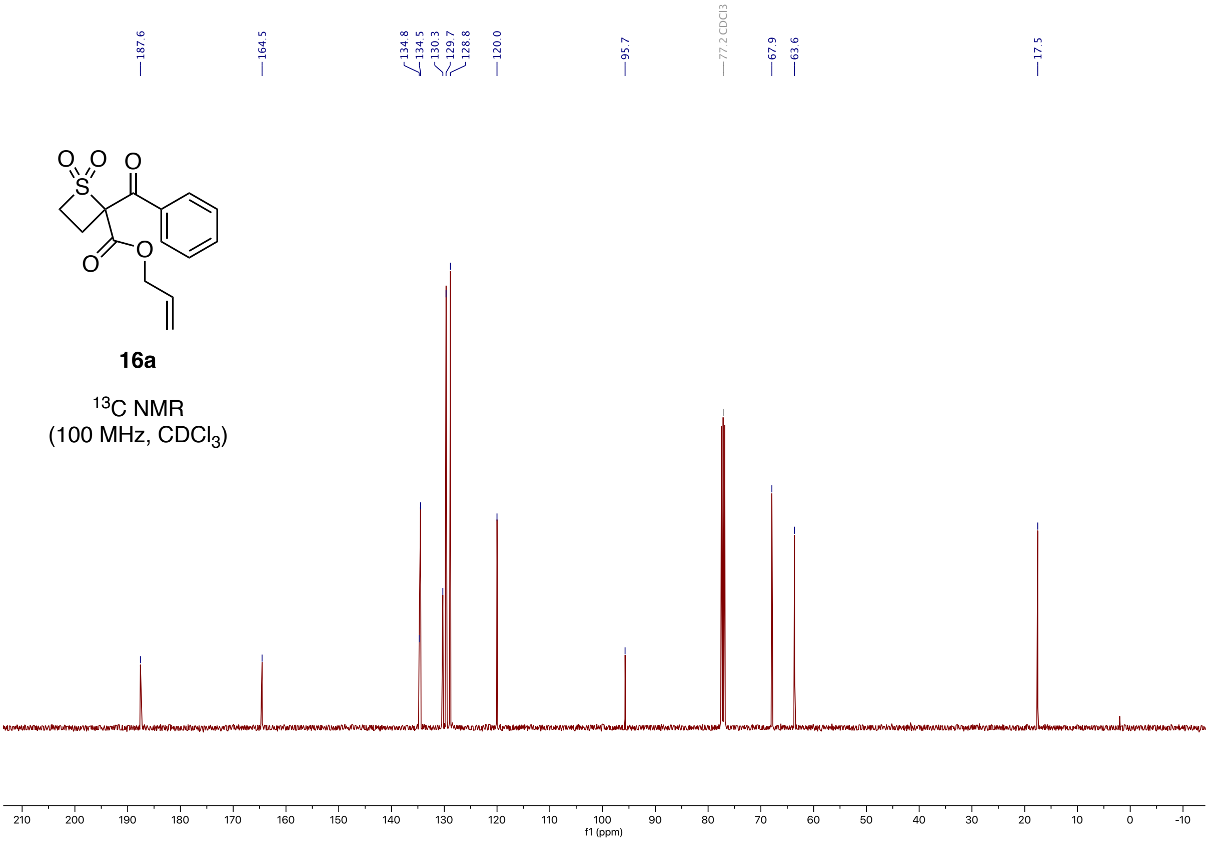
187.6
164.5
134.8
134.5
130.3
129.7
128.8
120.0
95.7
77.2 CDCl3
67.9
63.6
17.5
16a
^{13}C NMR
(100 MHz, $CDCl_3$)
f1 (ppm)

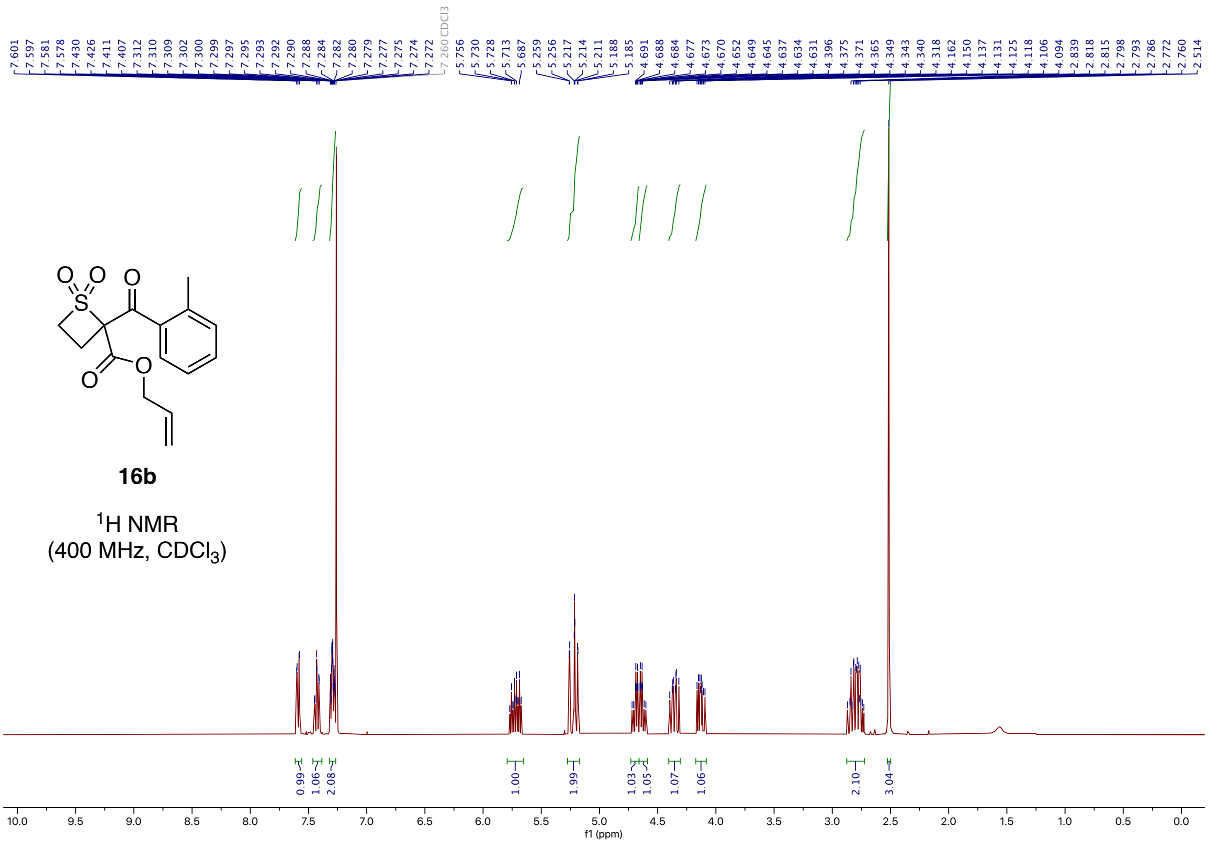
16b
¹H NMR
(400 MHz, CDCl3)
f1 (ppm)

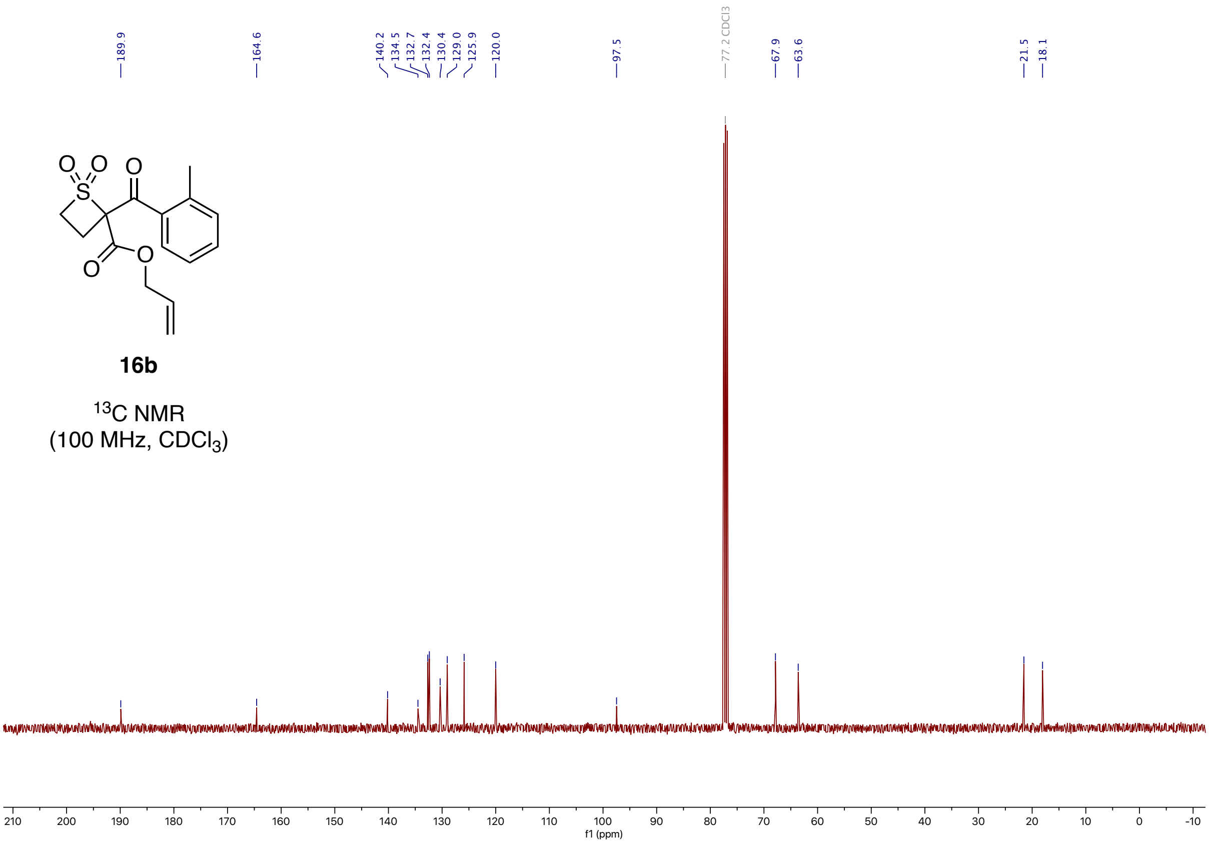
16b
¹³C NMR
(100 MHz, CDCl3)
f1 (ppm)

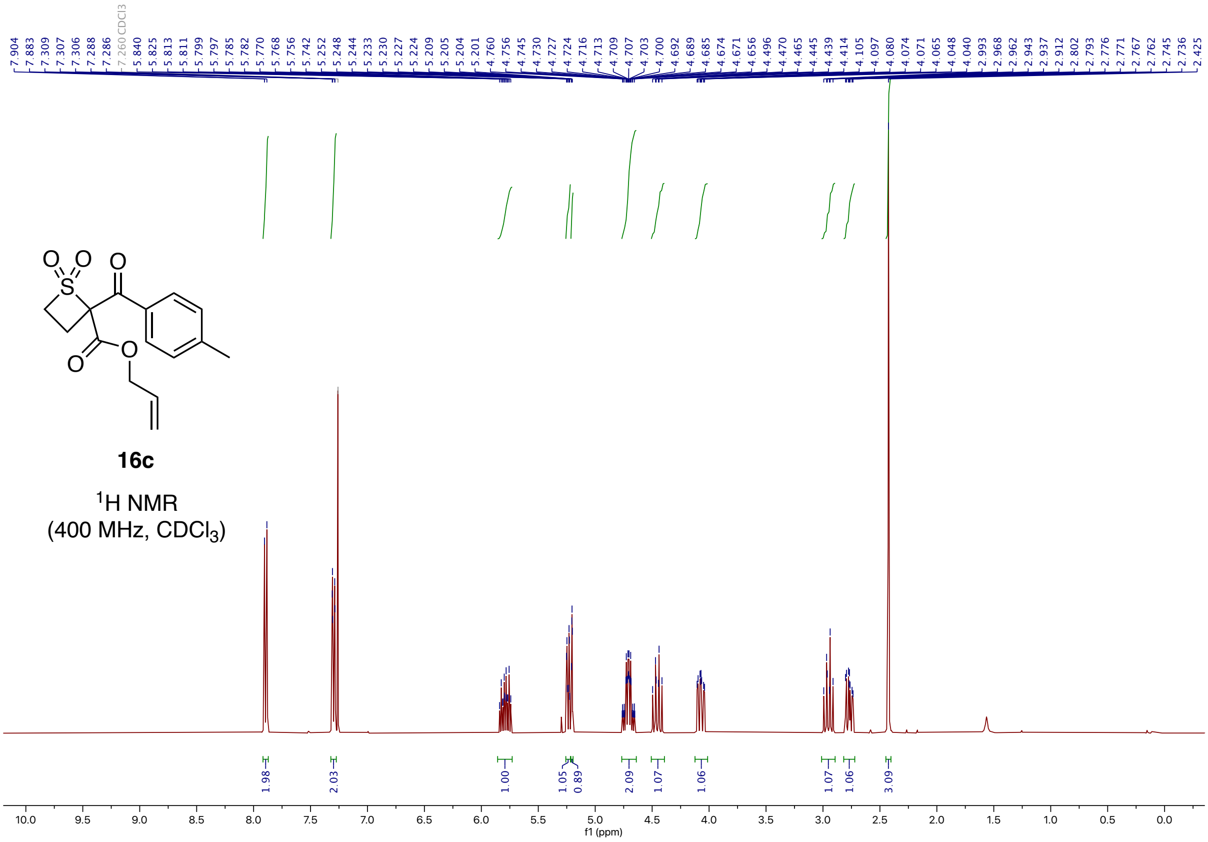
7.904 7.883 7.309 7.307 7.306 7.288 7.286 7.260 CDCl3 5.840 5.825 5.813 5.811 5.799 5.797 5.785 5.782 5.770 5.768 5.756 5.742 5.252 5.248 5.244 5.233 5.230 5.227 5.224 5.209 5.205 5.204 5.201 4.760 4.756 4.745 4.730 4.727 4.724 4.716 4.713 4.709 4.707 4.703 4.700 4.692 4.689 4.685 4.674 4.671 4.656 4.496 4.470 4.465 4.445 4.439 4.414 4.105 4.097 4.080 4.074 4.071 4.065 4.048 4.040 2.993 2.968 2.962 2.943 2.937 2.912 2.802 2.793 2.776 2.771 2.767 2.762 2.745 2.736 2.425
16c
^{1}H NMR
(400 MHz, $CDCl_3$)
1.98 2.03 1.00 1.05 0.89 2.09 1.07 1.06 1.07 1.06 3.09
f1 (ppm)

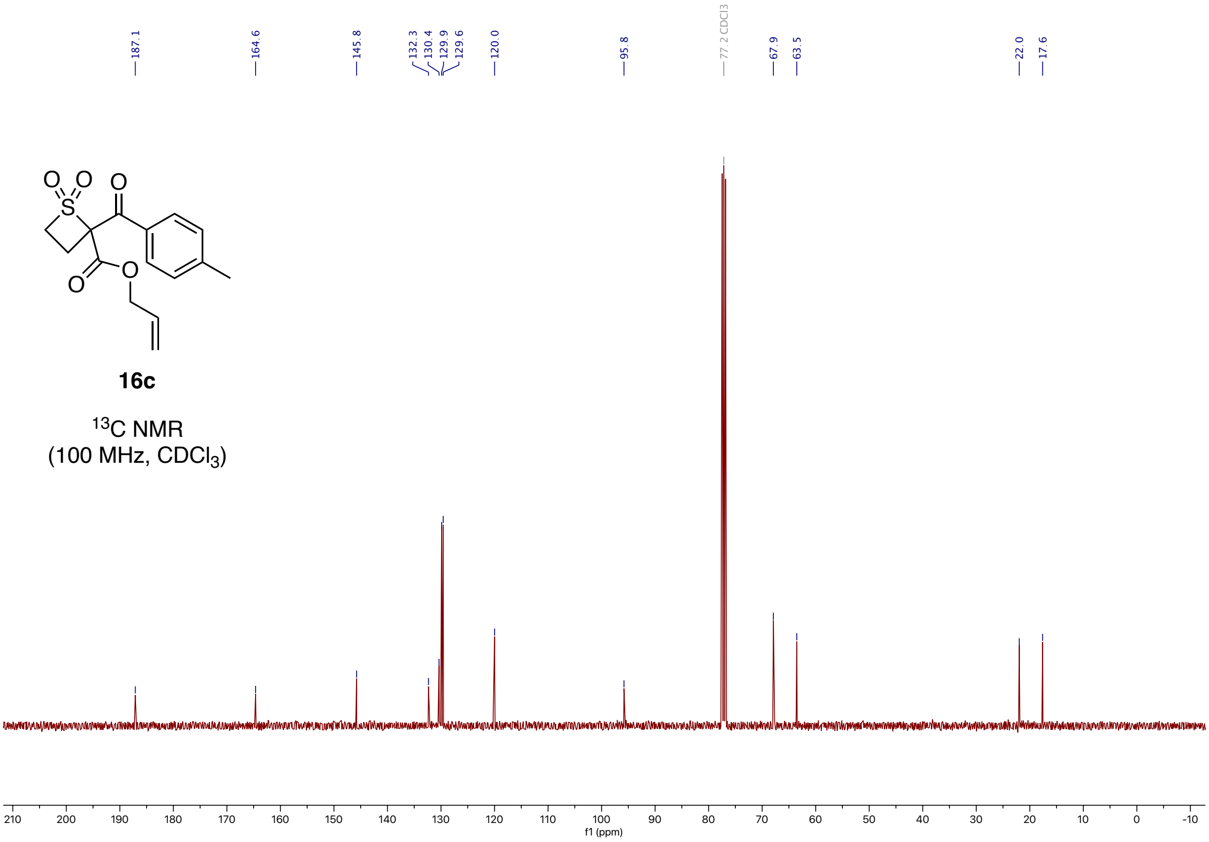
187.1 164.6 145.8 132.3 130.4 129.9 129.6 120.0 95.8 77.2 CDCl3 67.9 63.5 22.0 17.6
16c
^{13}C NMR
(100 MHz, $CDCl_3$)
f1 (ppm)

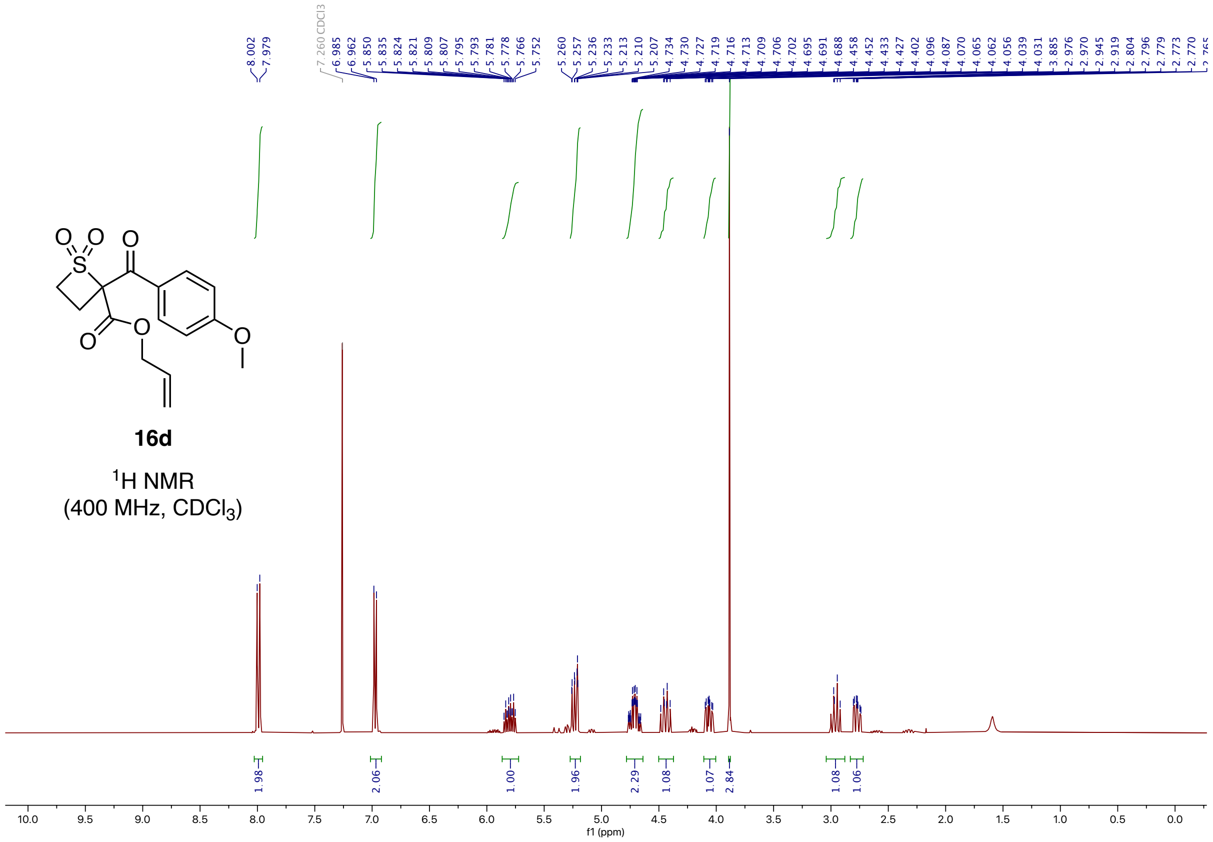
8.002
7.979
7.260 CDCl3
6.985
6.962
5.850
5.835
5.824
5.821
5.809
5.807
5.795
5.793
5.781
5.778
5.766
5.752
5.260
5.257
5.236
5.233
5.213
5.210
5.207
4.734
4.730
4.727
4.719
4.716
4.713
4.709
4.706
4.702
4.695
4.691
4.688
4.458
4.452
4.433
4.427
4.402
4.096
4.087
4.070
4.065
4.062
4.056
4.039
4.031
3.885
2.976
2.970
2.945
2.919
2.804
2.796
2.779
2.773
2.770
[illegible]
16d
1H NMR
(400 MHz, CDCl3)
1.98
2.06
1.00
1.96
2.29
1.08
1.07
2.84
1.08
1.06
f1 (ppm)

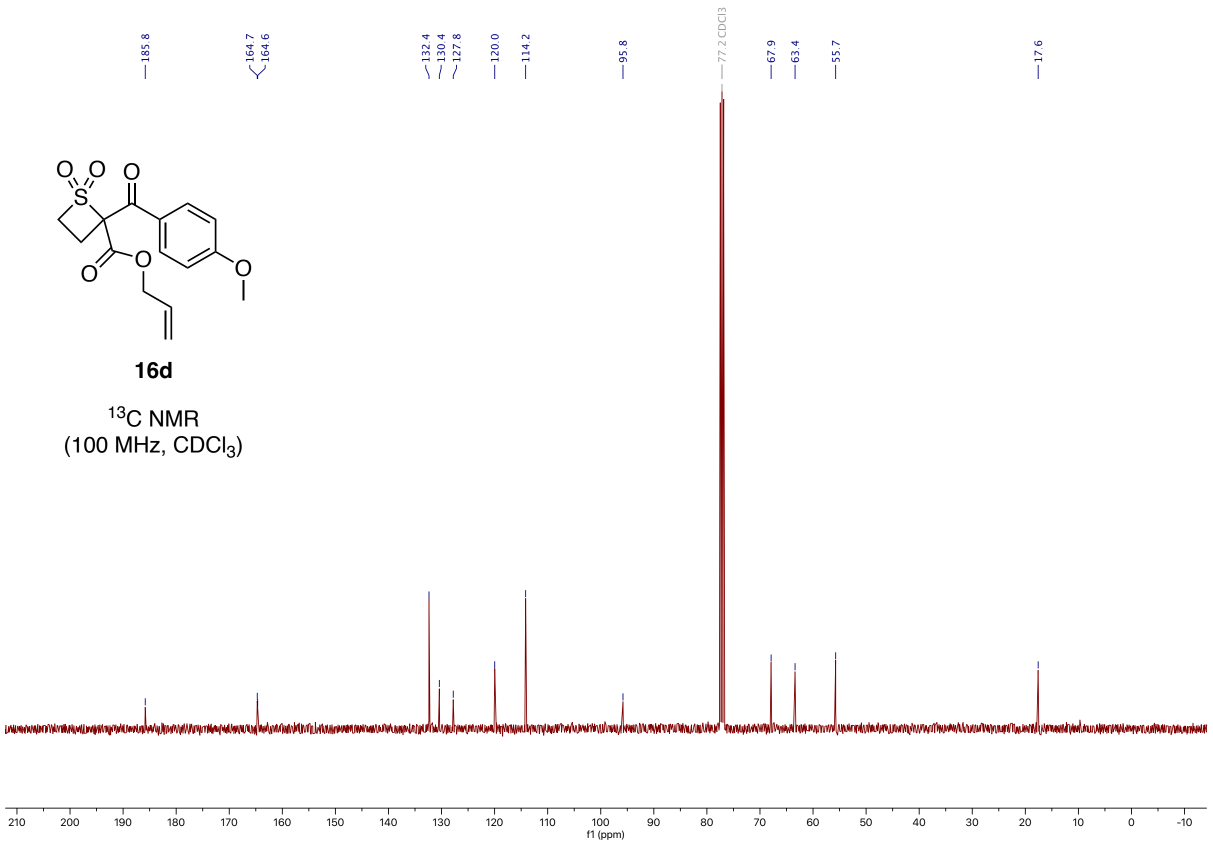
185.8
164.7
164.6
132.4
130.4
127.8
120.0
114.2
95.8
77.2 CDCl3
67.9
63.4
55.7
17.6
16d
13C NMR
(100 MHz, CDCl3)
f1 (ppm)

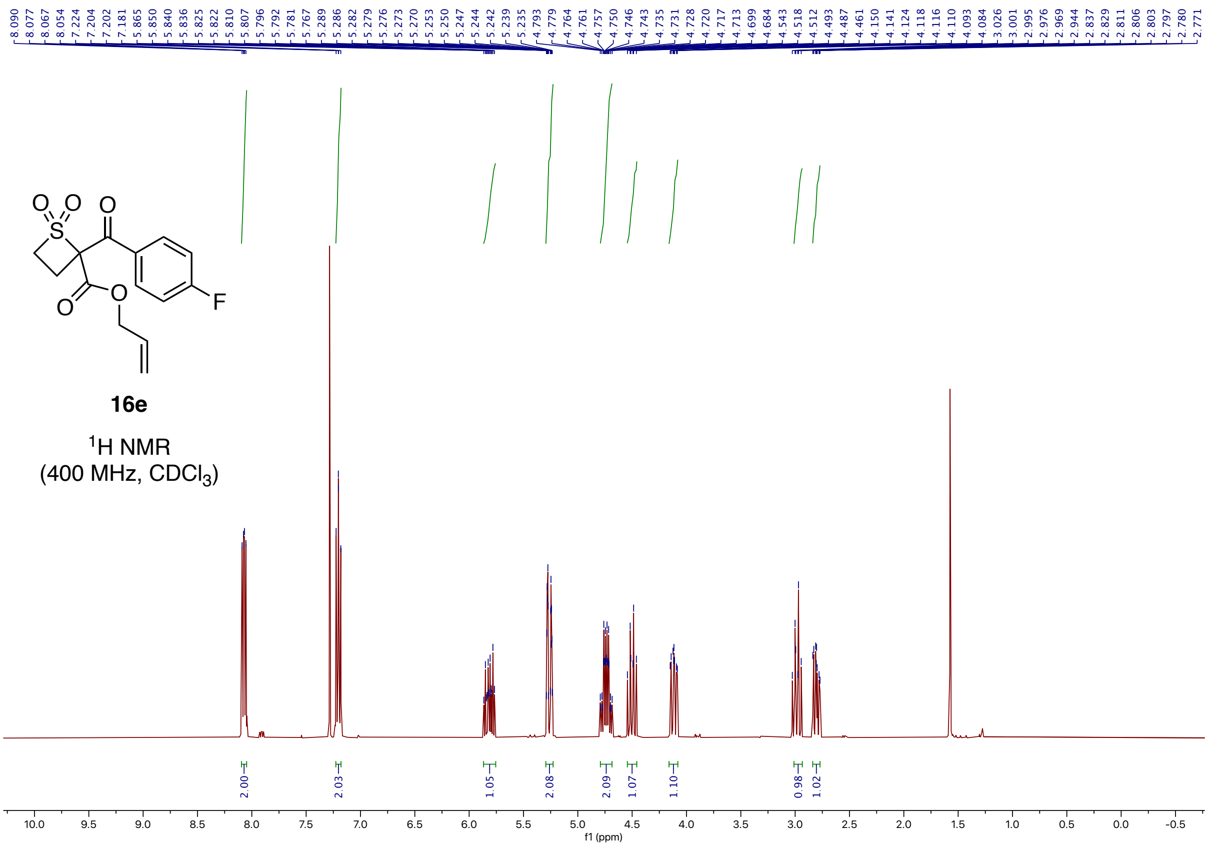
8.090 8.077 8.067 8.054 7.224 7.204 7.202 7.181 5.865 5.850 5.840 5.836 5.825 5.822 5.810 5.807 5.796 5.792 5.781 5.767 5.289 5.286 5.282 5.279 5.276 5.273 5.270 5.253 5.250 5.247 5.244 5.242 5.239 5.235 4.793 4.779 4.764 4.761 4.757 4.750 4.746 4.743 4.735 4.731 4.728 4.720 4.717 4.713 4.699 4.684 4.543 4.518 4.512 4.493 4.487 4.461 4.150 4.141 4.124 4.118 4.116 4.110 4.093 4.084 3.026 3.001 2.995 2.976 2.969 2.944 2.837 2.829 2.811 2.806 2.803 2.797 2.780 2.771
F
16e
1H NMR
(400 MHz, CDCl3)
2.00 2.03 1.05 2.08 2.09 1.07 1.10 0.98 1.02
f1 (ppm)

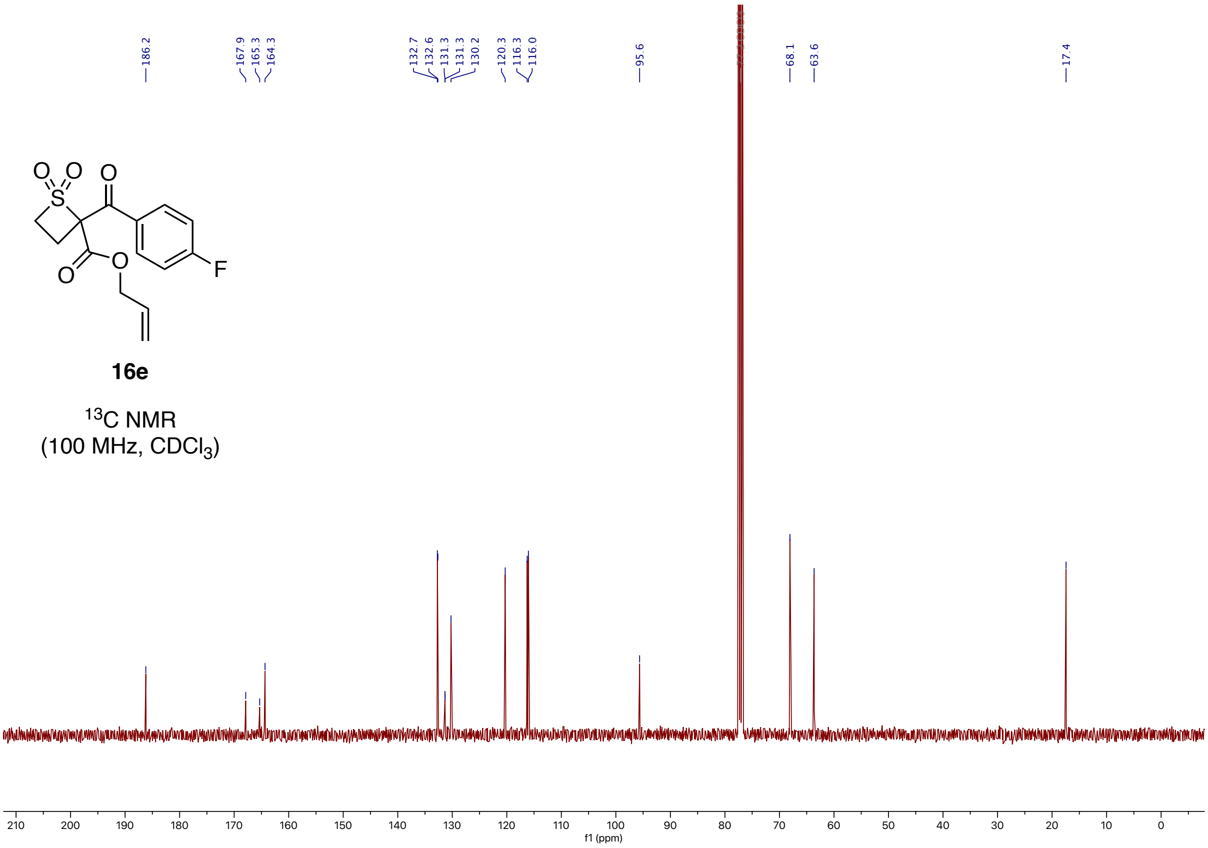
186.2 167.9 165.3 164.3 132.7 132.6 131.3 131.3 130.2 120.3 116.3 116.0 95.6 68.1 63.6 17.4
F
16e
13C NMR
(100 MHz, CDCl3)
f1 (ppm)

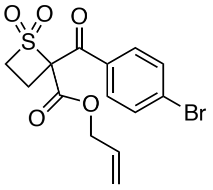

16f

^{1}H NMR
(400 MHz, $CDCl_3$)

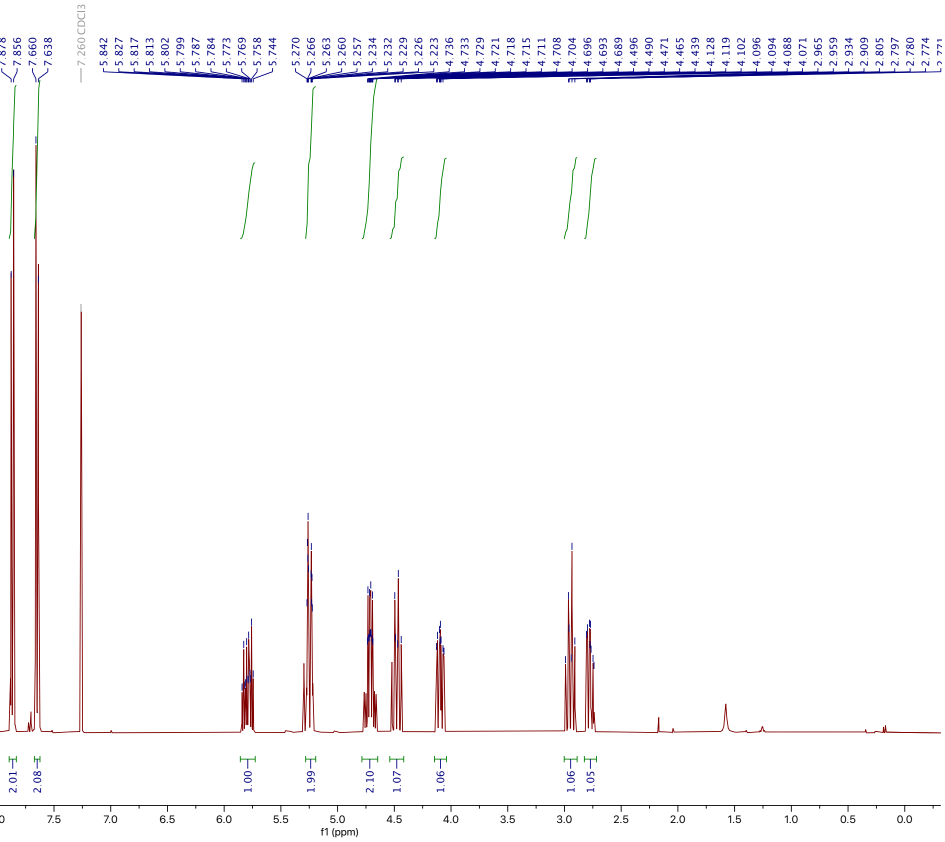

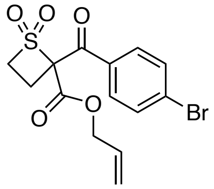

16f

^{13}C NMR
(100 MHz, $CDCl_3$)

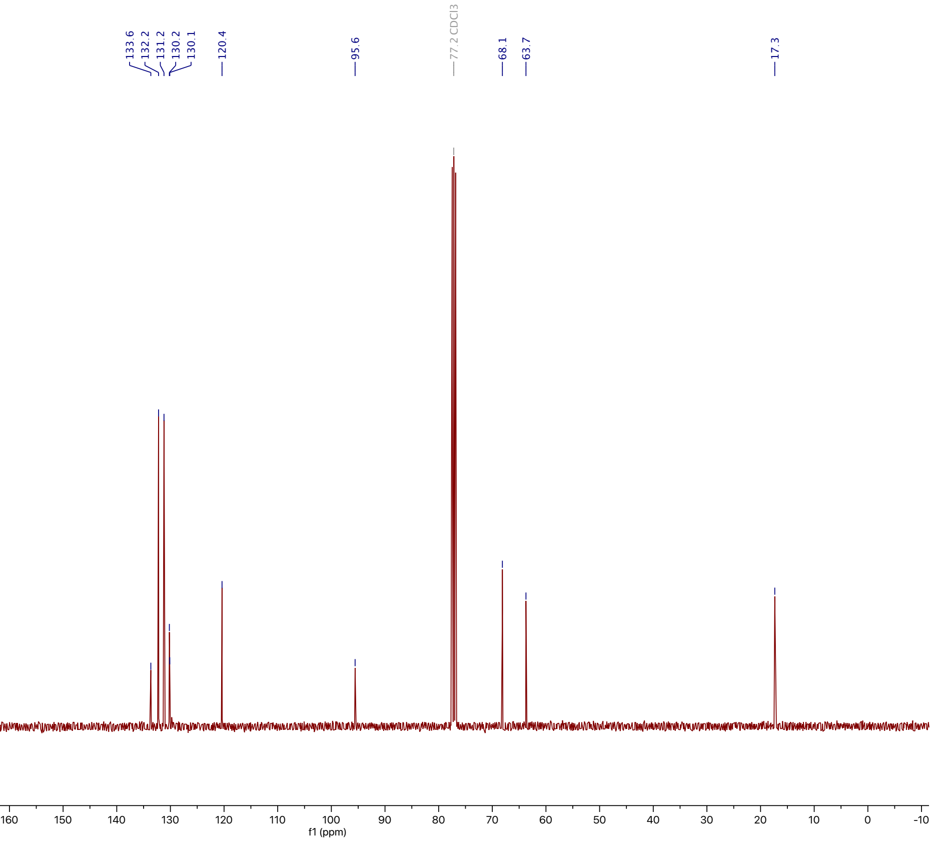

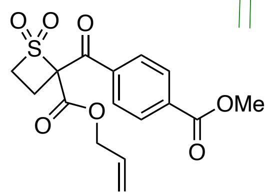

16g

^1H NMR
(400 MHz, $CDCl_3$)

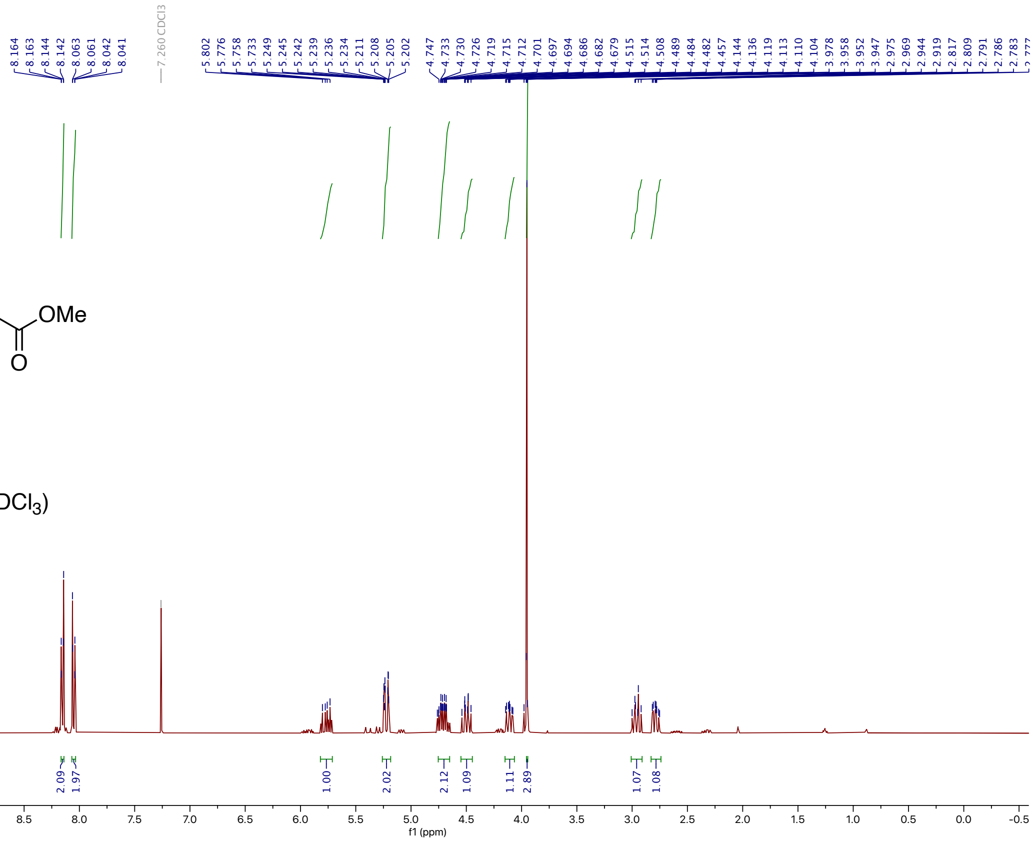

16g

^{13}C NMR
(100 MHz, $CDCl_3$)

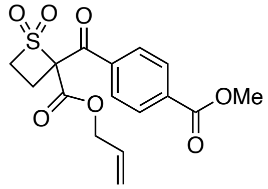

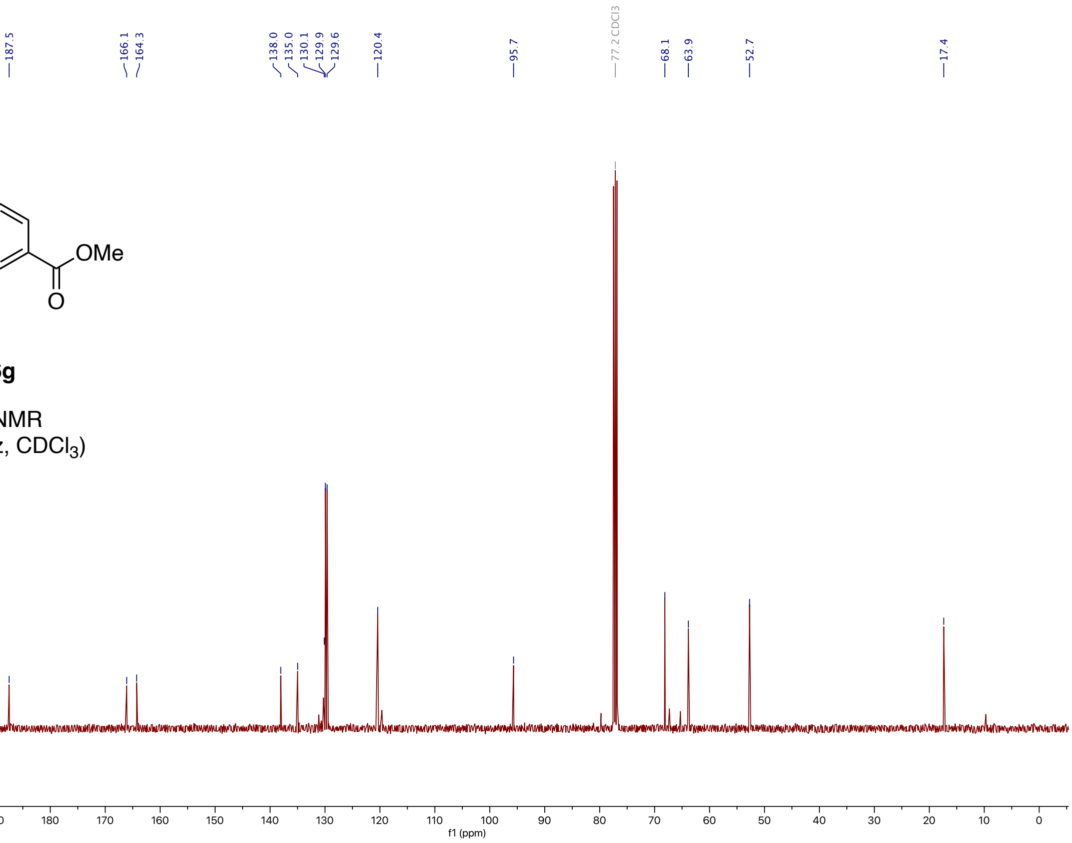

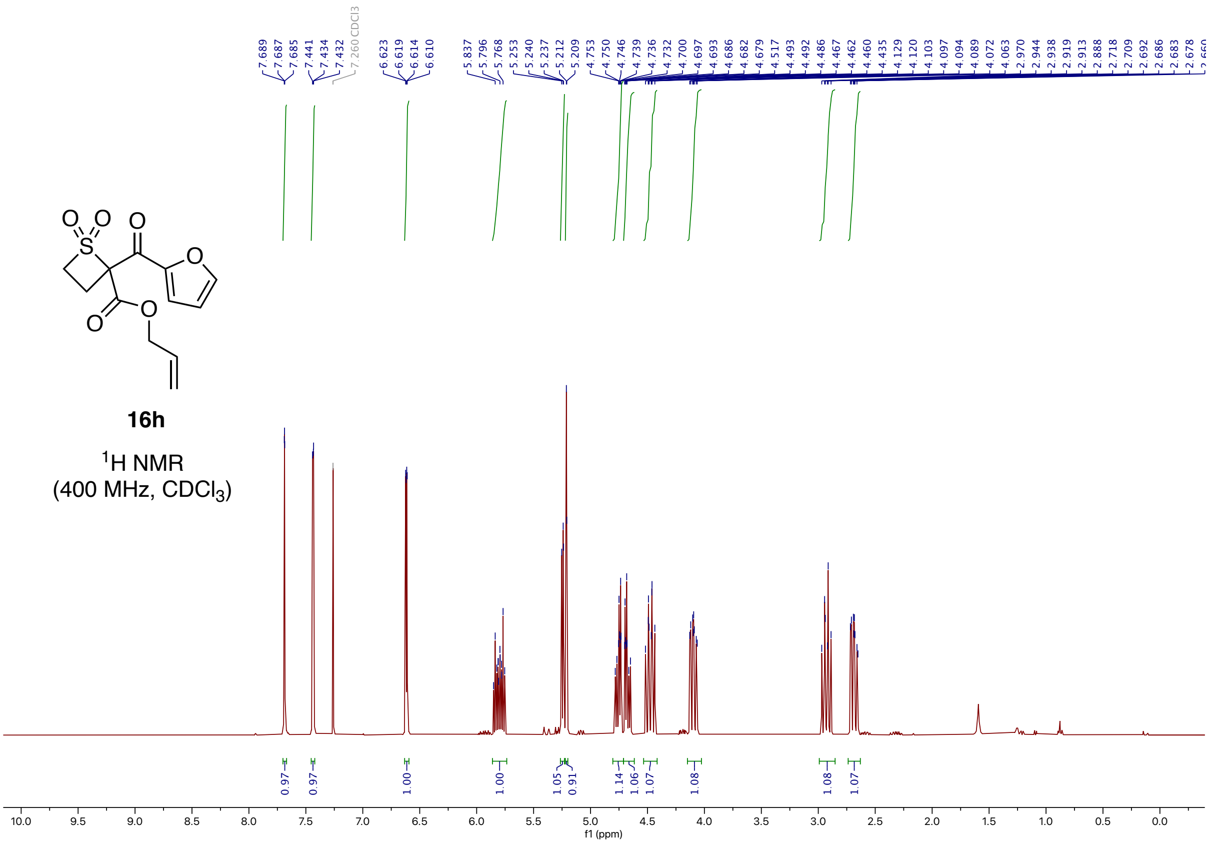
7.689
7.687
7.685
7.441
7.434
7.432
7.260 CDCl3
6.623
6.619
6.614
6.610
5.837
5.796
5.768
5.253
5.240
5.237
5.212
5.209
4.753
4.750
4.746
4.739
4.736
4.732
4.700
4.697
4.693
4.686
4.682
4.679
4.517
4.493
4.492
4.486
4.467
4.462
4.460
4.435
4.129
4.120
4.103
4.097
4.094
4.089
4.072
4.063
2.970
2.944
2.938
2.919
2.913
2.888
2.718
2.709
2.692
2.686
2.683
2.678
16h
1H NMR
(400 MHz, CDCl3)
0.97
0.97
1.00
1.00
1.05
0.91
1.14
1.06
1.07
1.08
1.08
1.07
f1 (ppm)

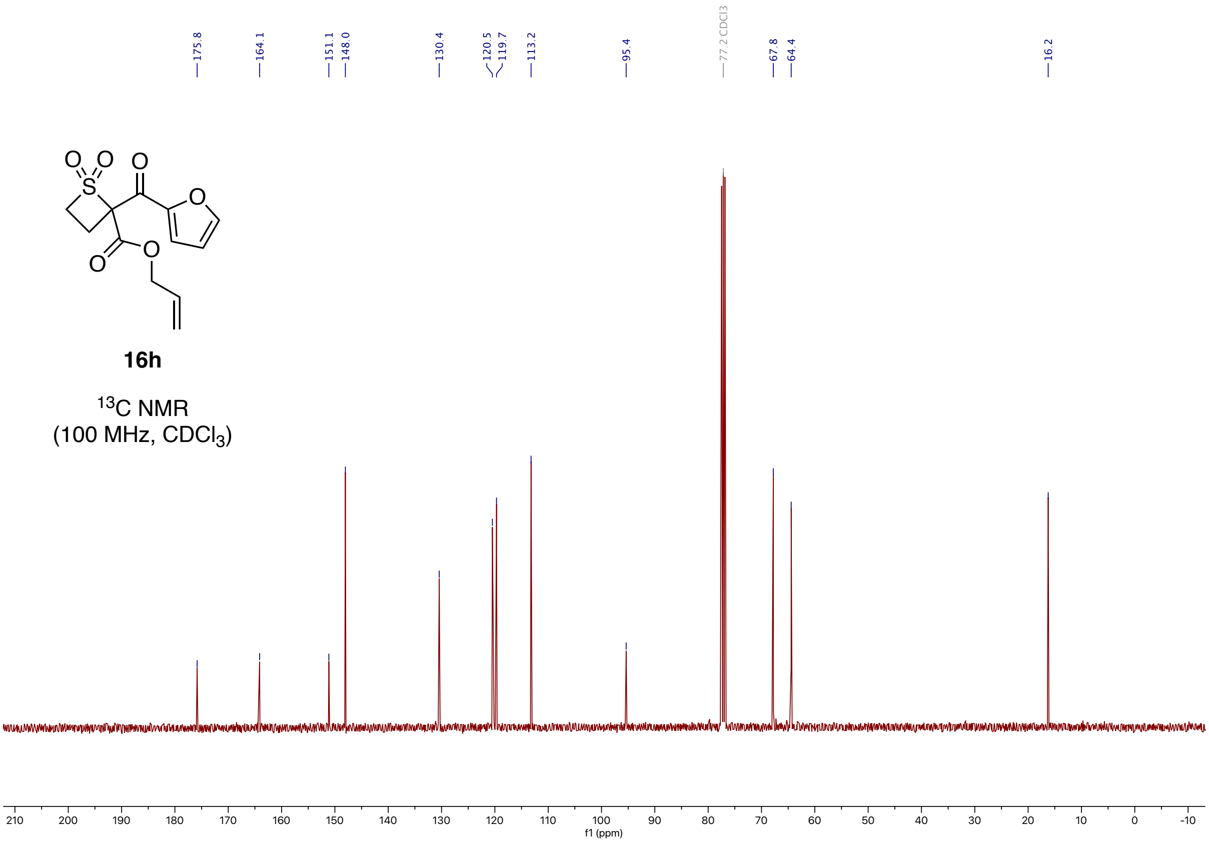
175.8
164.1
151.1
148.0
130.4
120.5
119.7
113.2
95.4
77.2 CDCl3
67.8
64.4
16.2
16h
13C NMR
(100 MHz, CDCl3)
f1 (ppm)

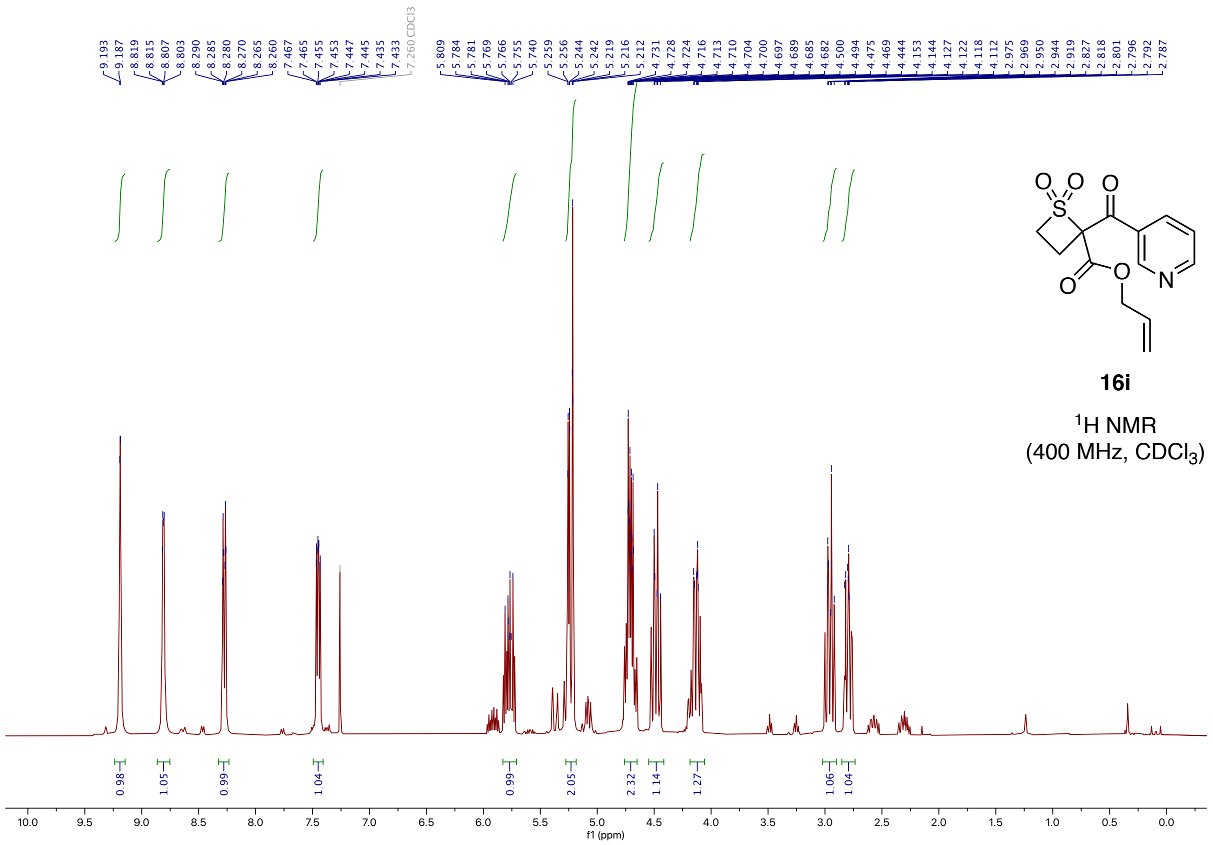
16i
^{1}H NMR
(400 MHz, CDCl$_3$)
f1 (ppm)

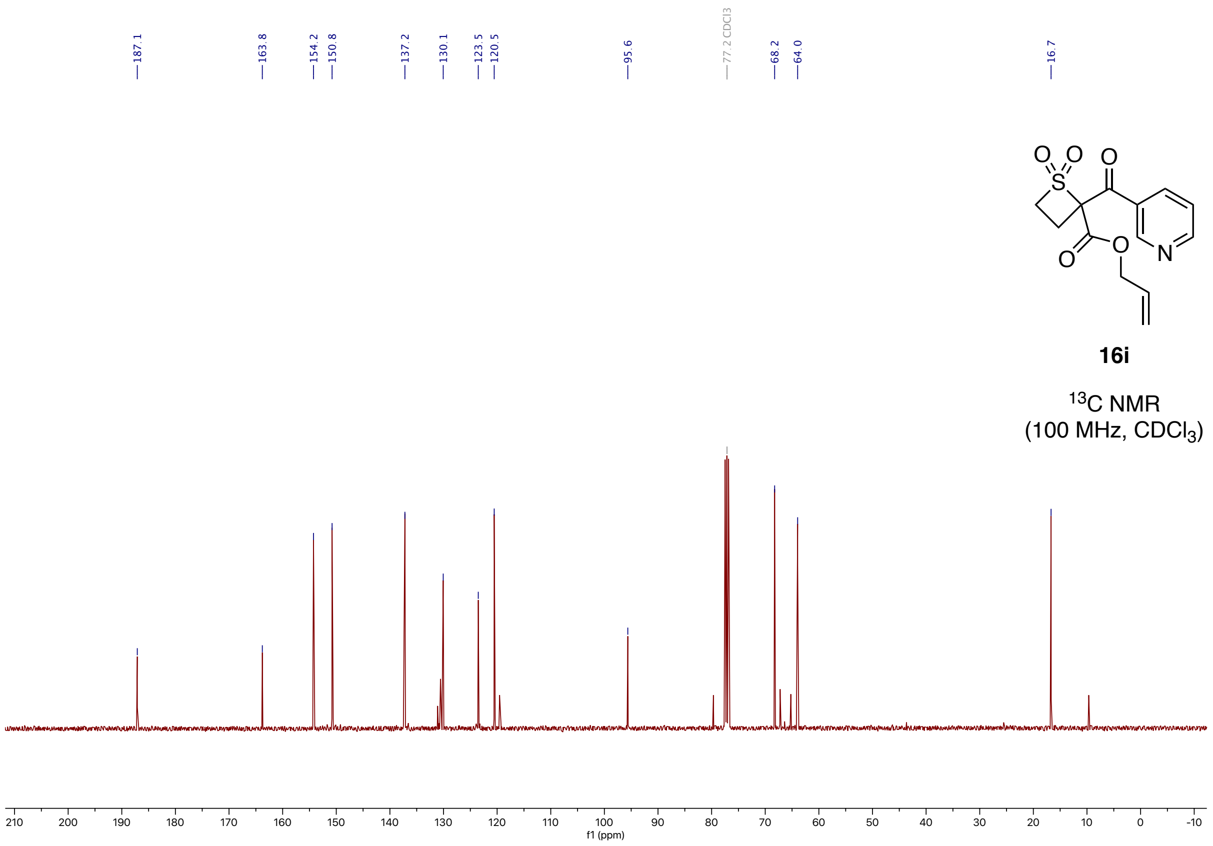
16i
^{13}C NMR
(100 MHz, CDCl$_3$)
187.1
163.8
154.2
150.8
137.2
130.1
123.5
120.5
95.6
77.2 CDCl3
68.2
64.0
16.7
f1 (ppm)

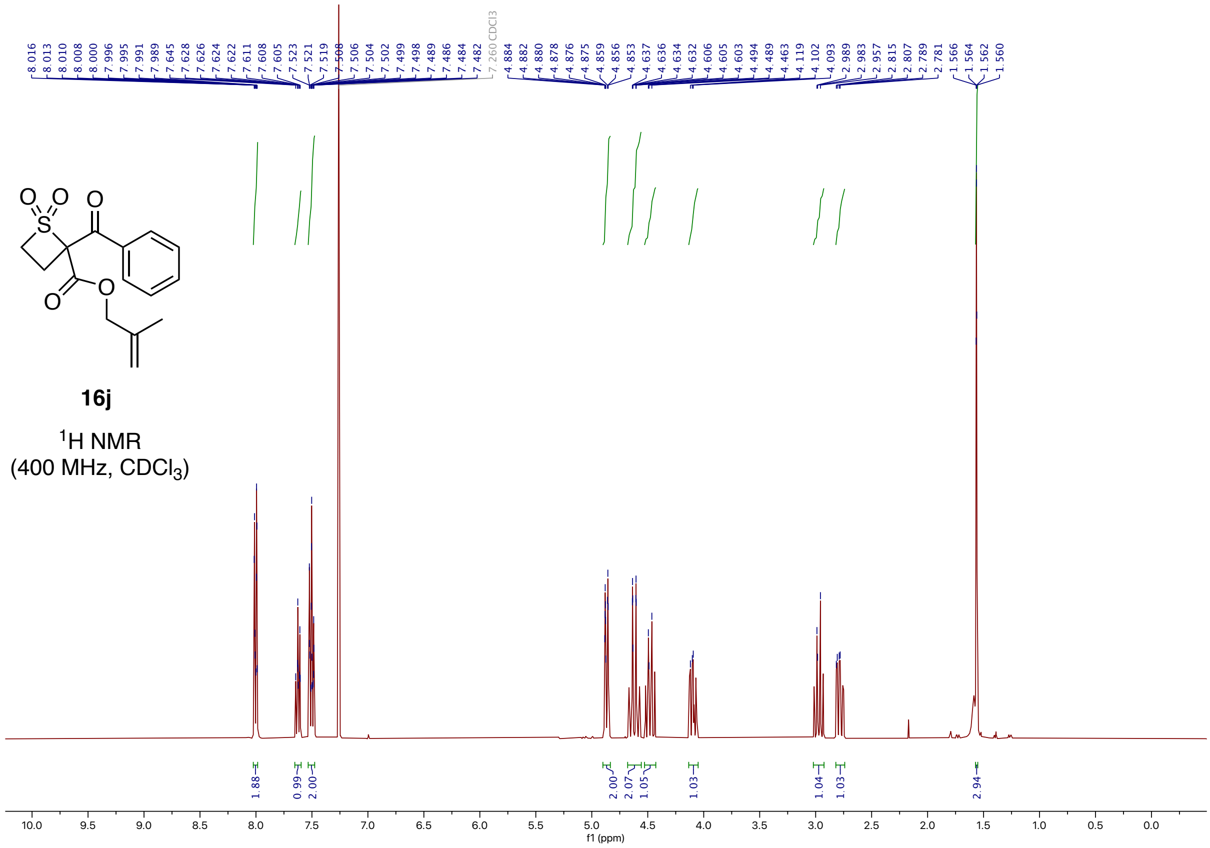

16j

^{1}H NMR
(400 MHz, $CDCl_3$)

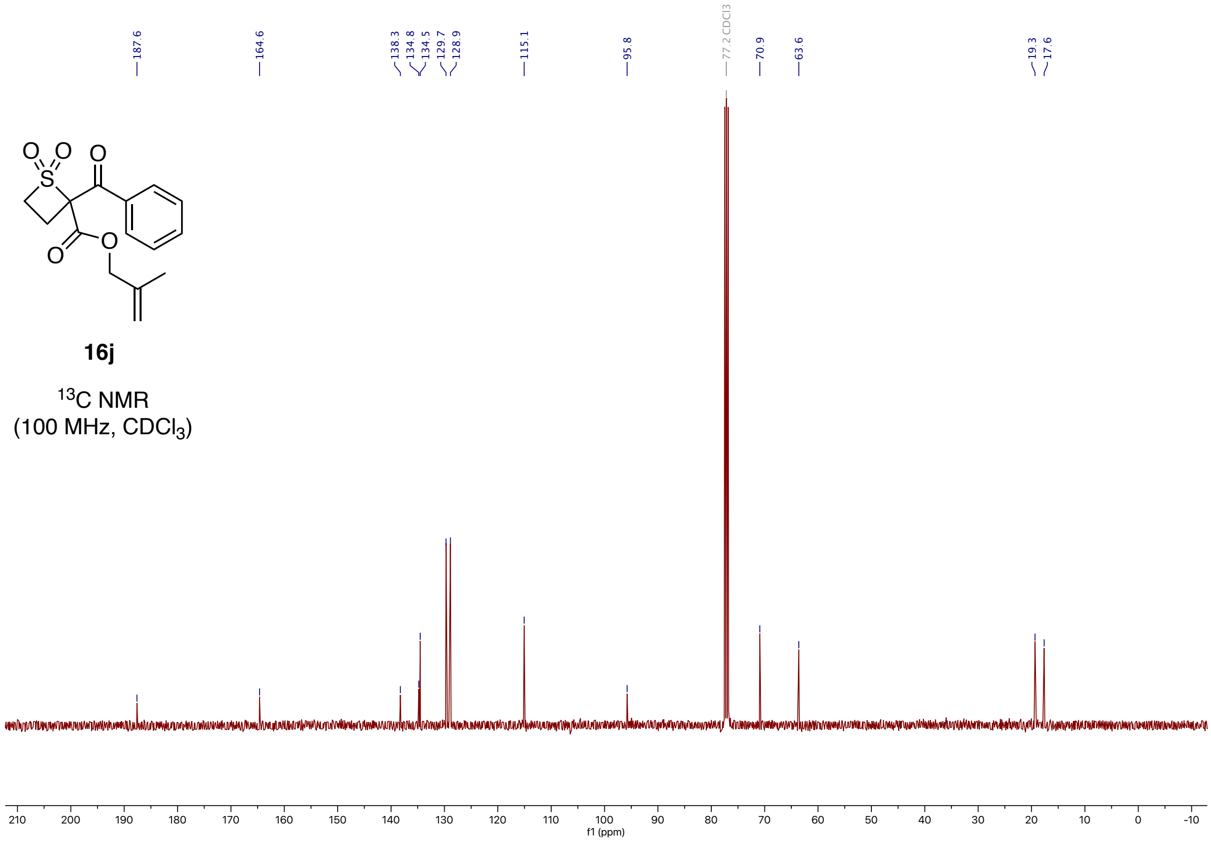

16j

^{13}C NMR
(100 MHz, $CDCl_3$)

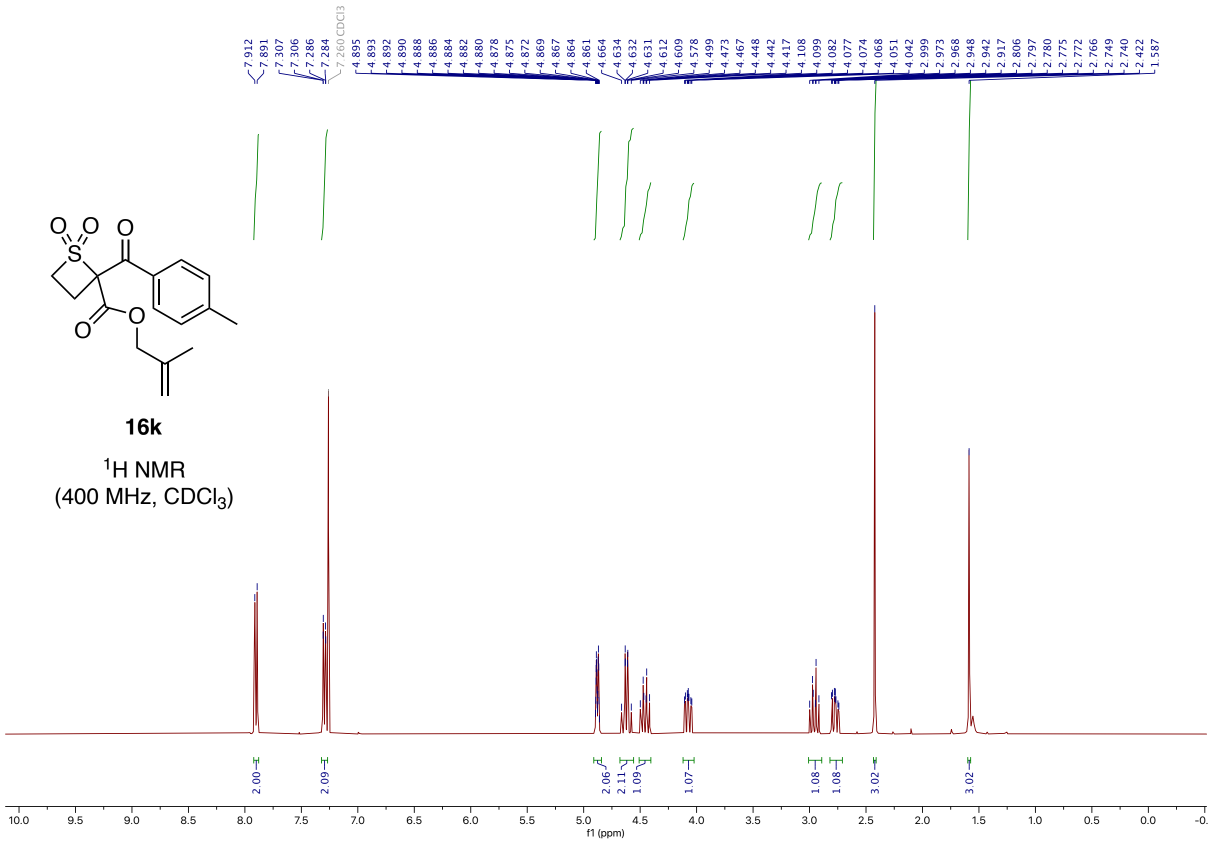
7.912
7.891
7.307
7.306
7.286
7.284
7.260 CDCl3
4.895
4.893
4.892
4.890
4.888
4.886
4.884
4.882
4.880
4.878
4.875
4.872
4.869
4.867
4.864
4.861
4.664
4.634
4.632
4.631
4.612
4.609
4.578
4.499
4.473
4.467
4.448
4.442
4.417
4.108
4.099
4.082
4.077
4.074
4.068
4.051
4.042
2.999
2.973
2.968
2.948
2.942
2.917
2.806
2.797
2.780
2.775
2.772
2.766
2.749
2.740
2.422
1.587
16k
1H NMR
(400 MHz, CDCl3)
2.00
2.09
2.06
2.11
1.09
1.07
1.08
1.08
3.02
3.02
10.0
9.5
9.0
8.5
8.0
7.5
7.0
6.5
6.0
5.5
5.0
4.5
4.0
3.5
3.0
2.5
2.0
1.5
1.0
0.5
0.0
f1 (ppm)

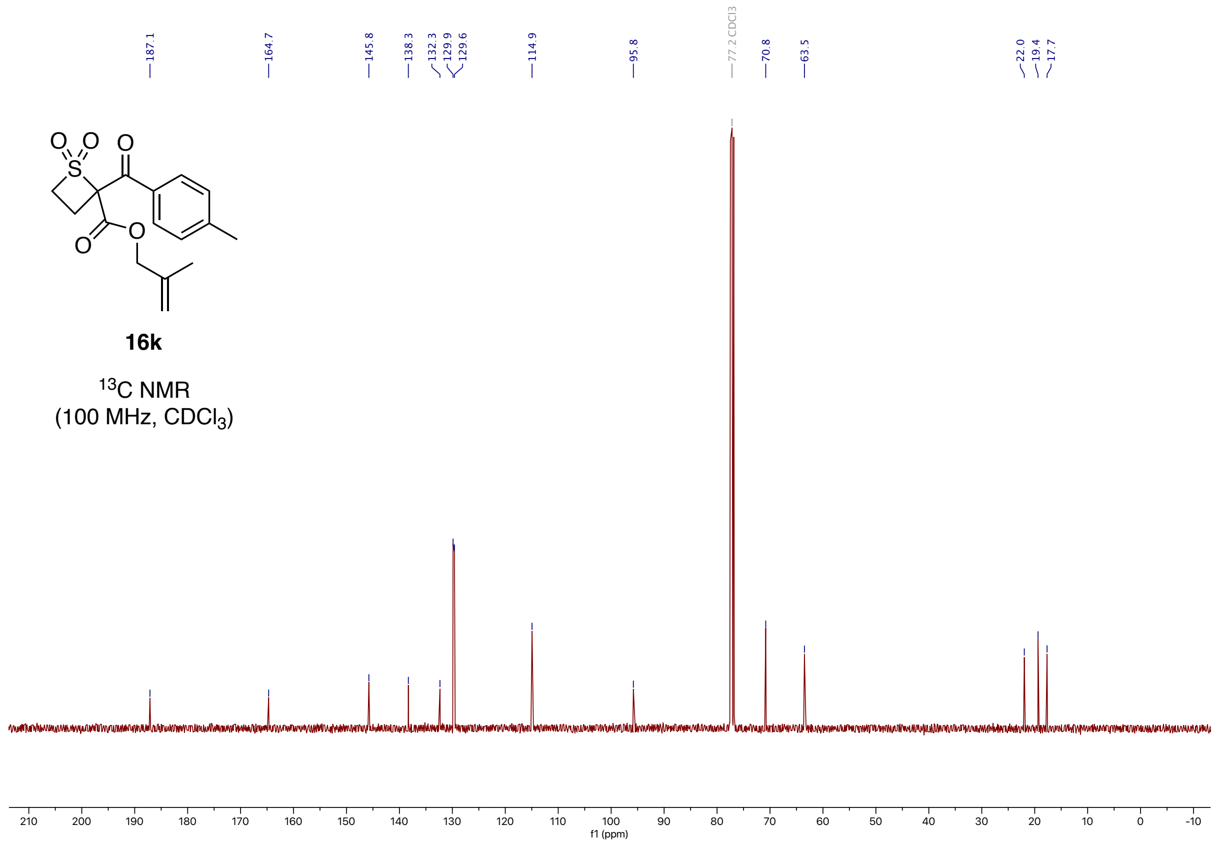
187.1
164.7
145.8
138.3
132.3
129.9
129.6
114.9
95.8
77.2 CDCl3
70.8
63.5
22.0
19.4
17.7
16k
13C NMR
(100 MHz, CDCl3)
210
200
190
180
170
160
150
140
130
120
110
100
90
80
70
60
50
40
30
20
10
0
-10
f1 (ppm)

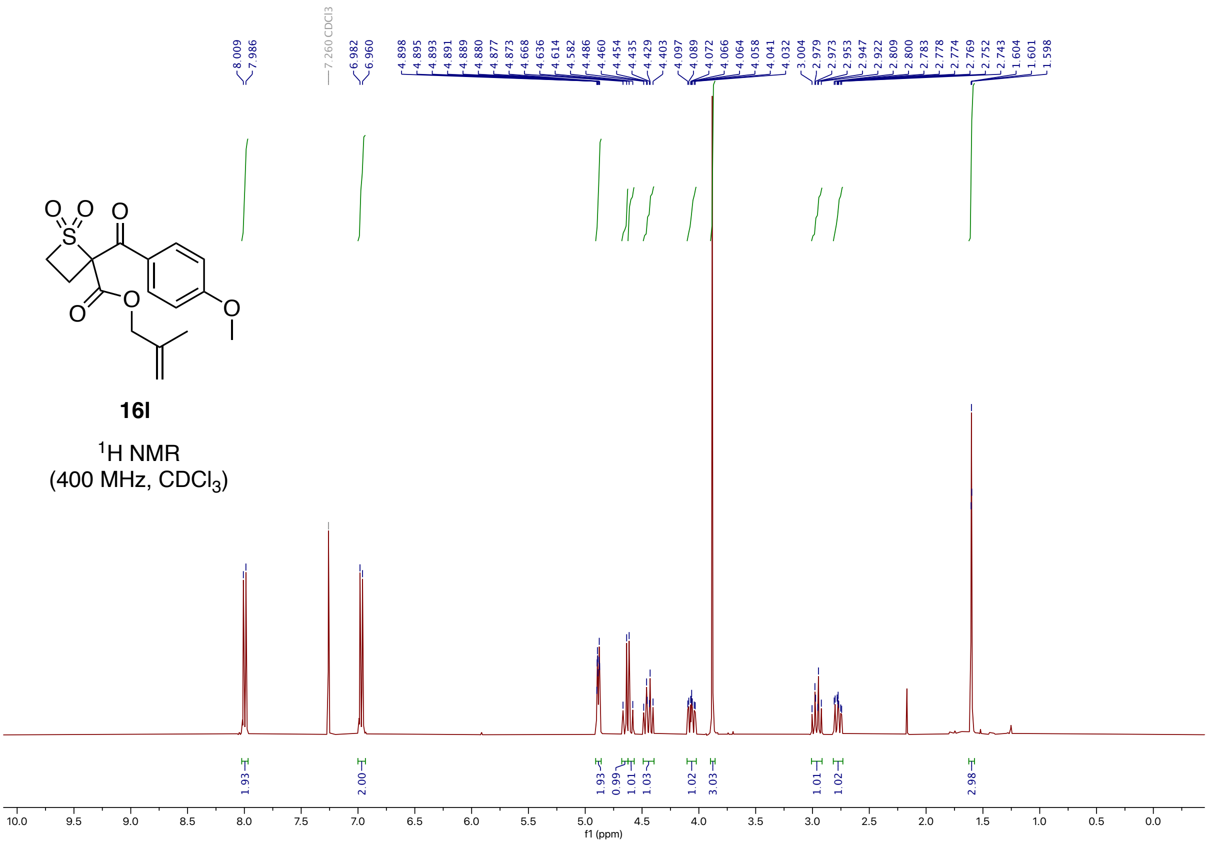

16l

^{1}H NMR
(400 MHz, $CDCl_3$)

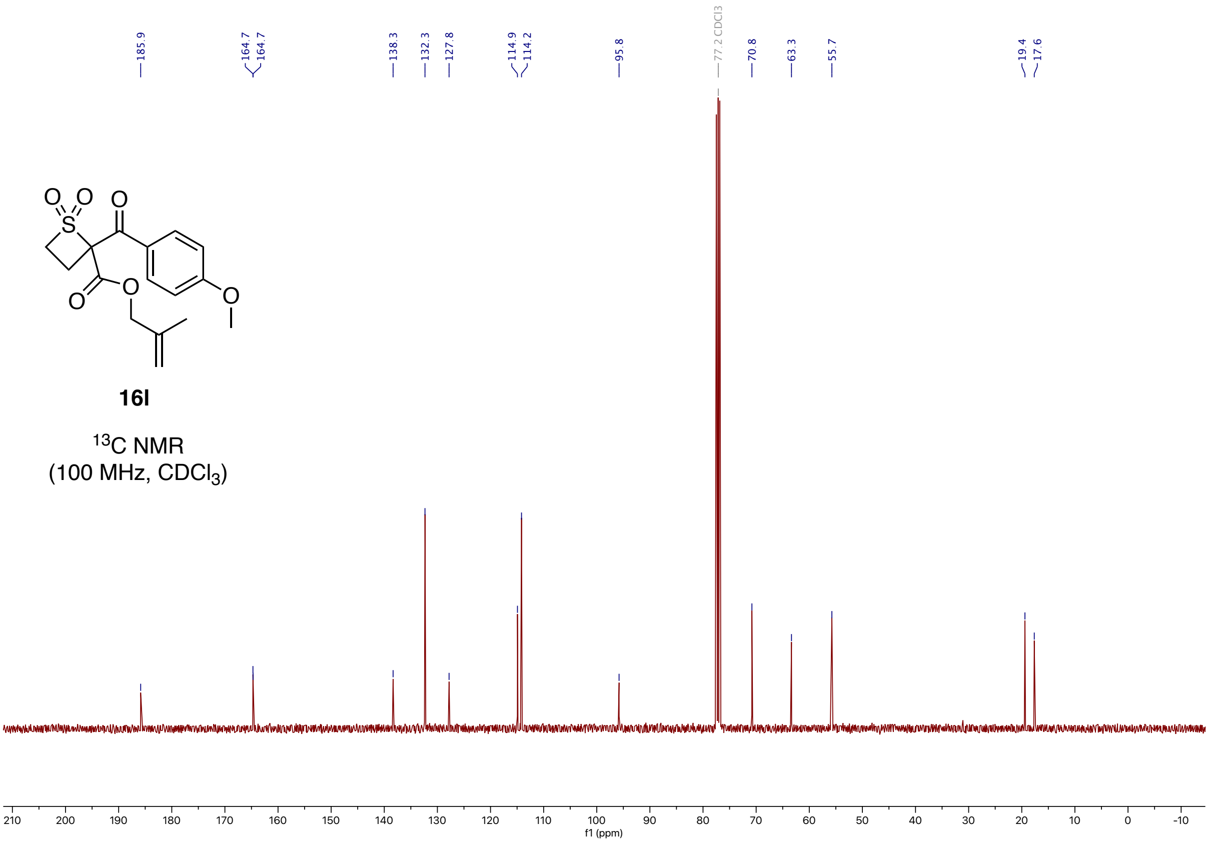

16l

^{13}C NMR
(100 MHz, $CDCl_3$)

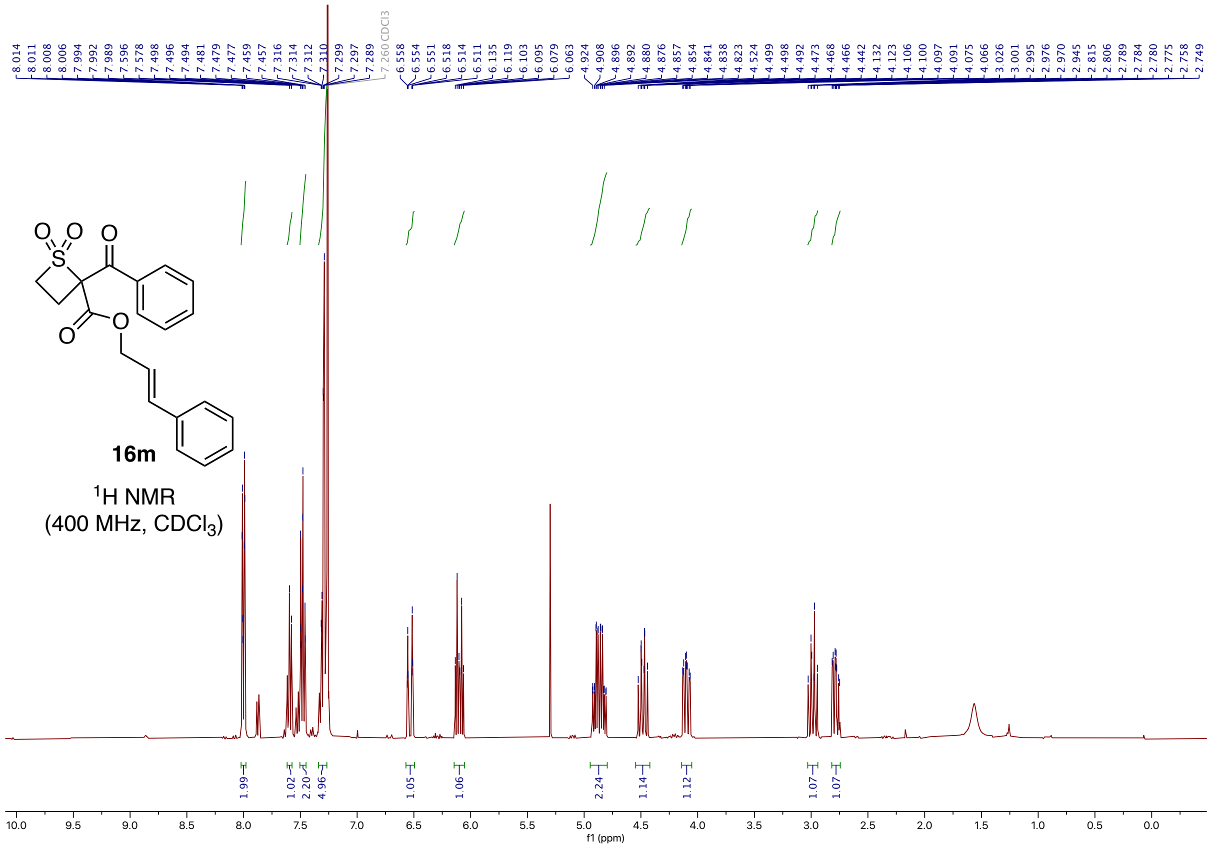
8.014 8.011 8.008 8.006 7.994 7.992 7.989 7.596 7.578 7.498 7.496 7.494 7.481 7.479 7.477 7.459 7.457 7.316 7.314 7.312 7.310 7.299 7.297 7.289
7.260 CDCl3
6.558 6.554 6.551 6.518 6.514 6.511 6.135 6.119 6.103 6.095 6.079 6.063
4.924 4.908 4.896 4.892 4.880 4.876 4.857 4.854 4.841 4.838 4.823 4.524 4.499 4.498 4.492 4.473 4.468 4.466 4.442 4.132 4.123 4.106 4.100 4.097 4.091 4.075 4.066
3.026 3.001 2.995 2.976 2.970 2.945 2.815 2.806 2.789 2.784 2.780 2.775 2.758 2.749
16m
^{1}H NMR
(400 MHz, $CDCl_3$)
1.99 1.02 2.20 4.96 1.05 1.06 2.24 1.14 1.12 1.07 1.07
f1 (ppm)

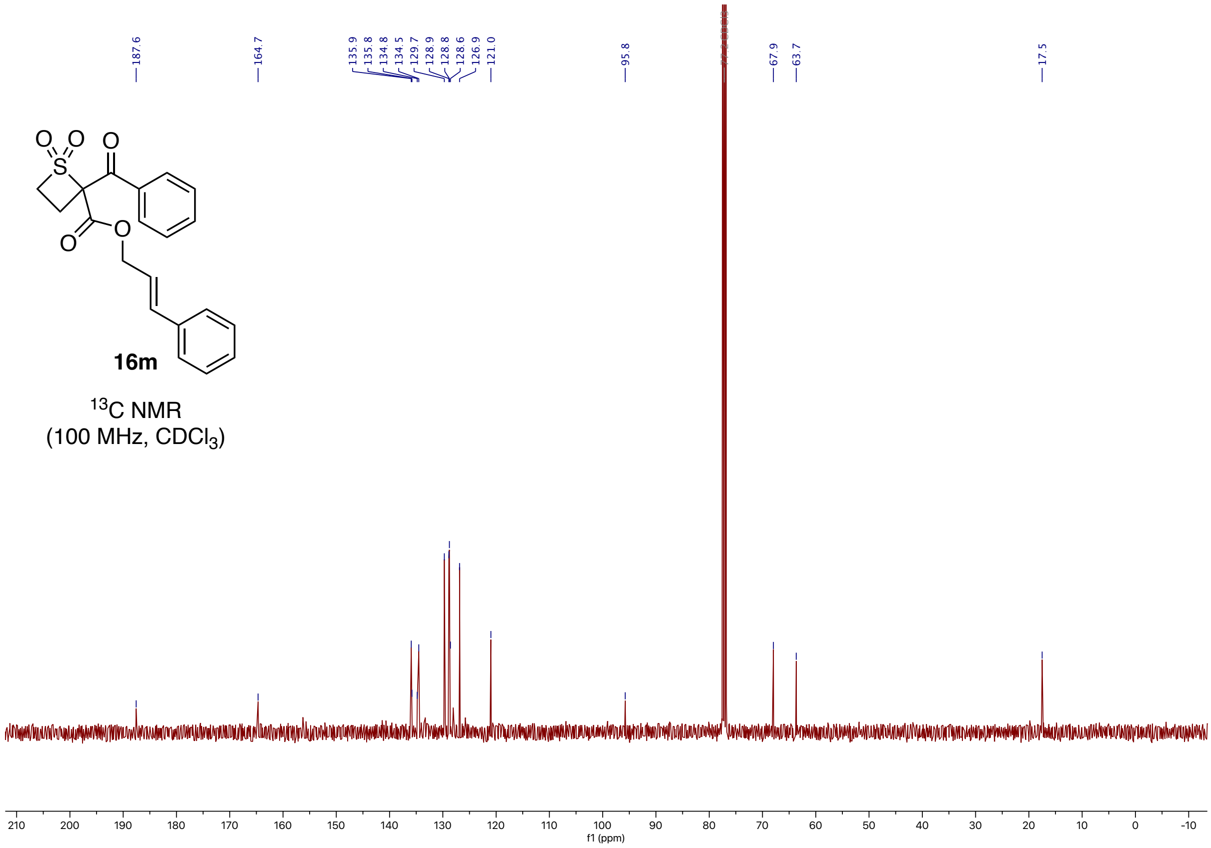
187.6
164.7
135.9 135.8 134.8 134.5 129.7 128.9 128.8 128.6 126.9 121.0
95.8
67.9
63.7
17.5
16m
^{13}C NMR
(100 MHz, $CDCl_3$)
f1 (ppm)

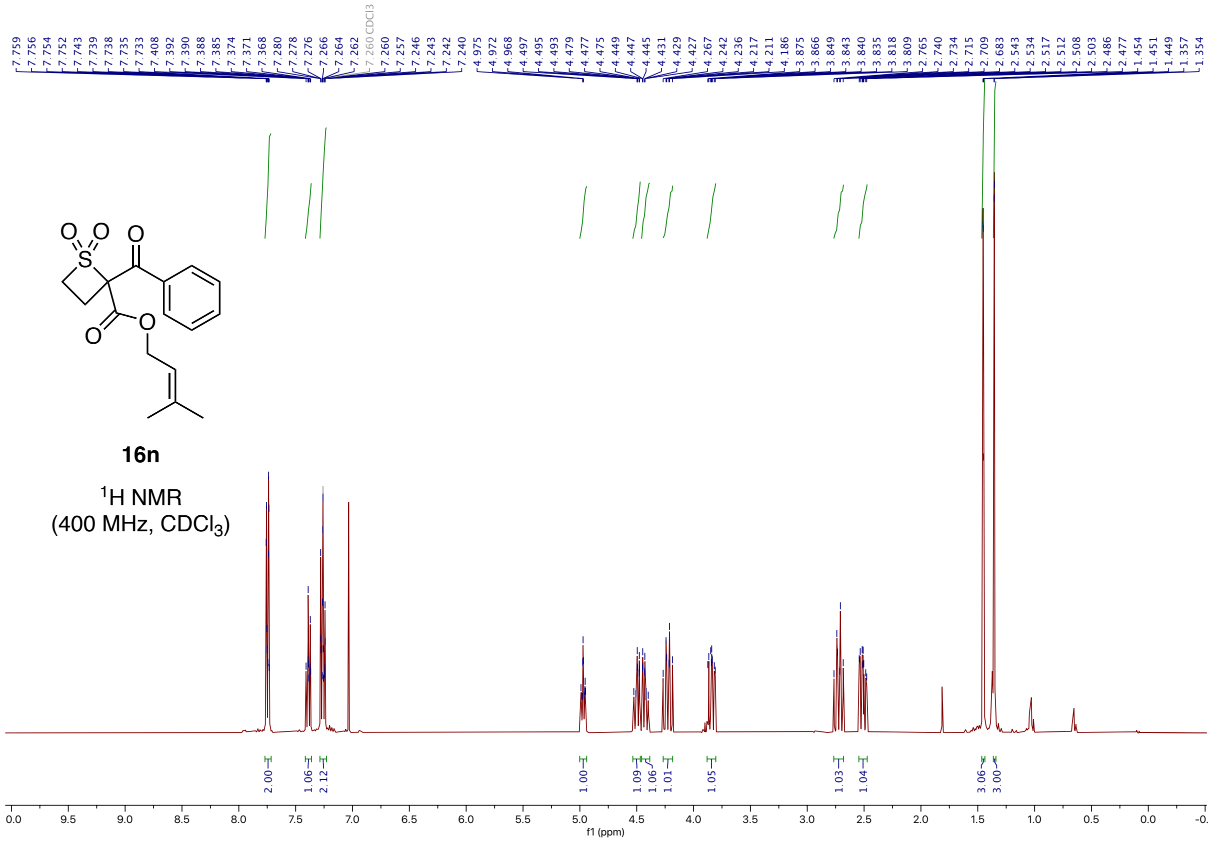
16n
^{1}H NMR
(400 MHz, CDCl$_3$)
f1 (ppm)

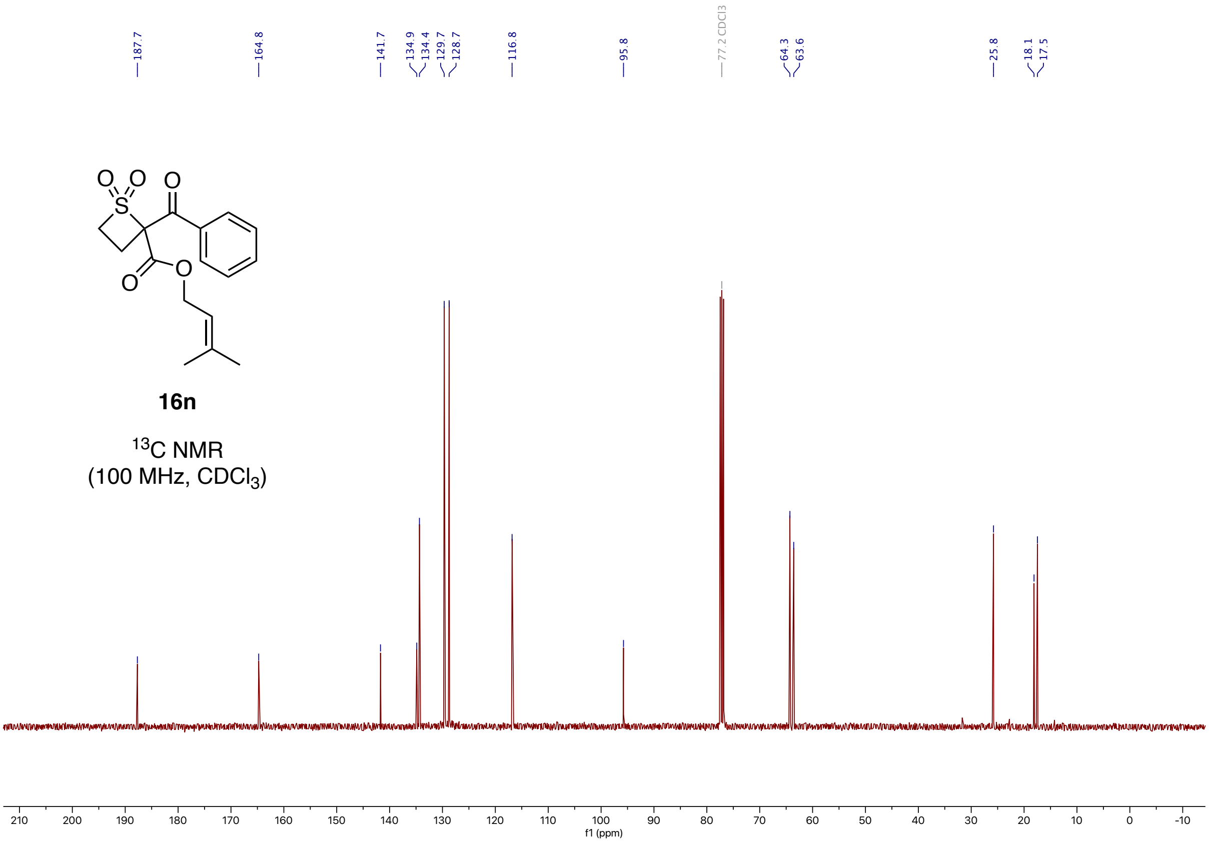
16n
^{13}C NMR
(100 MHz, CDCl$_3$)
187.7
164.8
141.7
134.9
134.4
129.7
128.7
116.8
95.8
77.2 CDCl3
64.3
63.6
25.8
18.1
17.5
f1 (ppm)

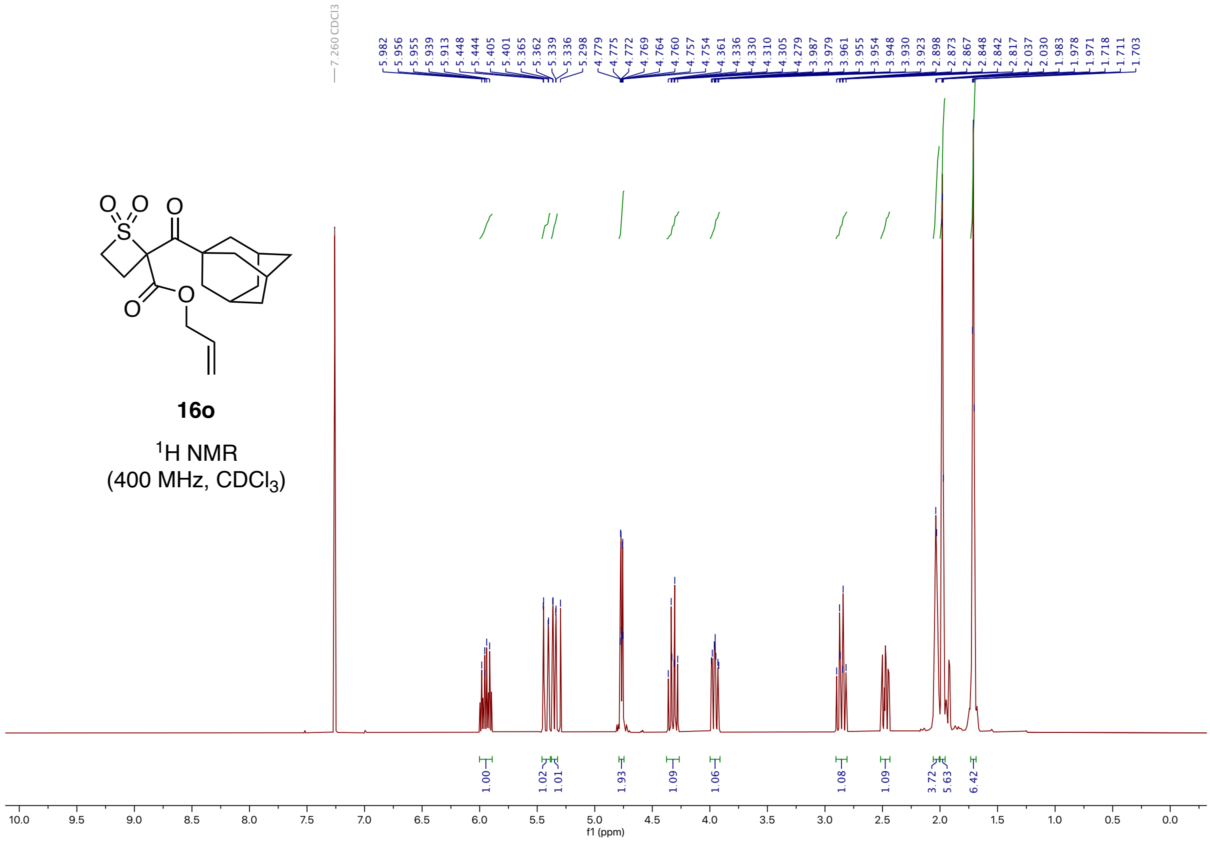

16o
^{1}H NMR
(400 MHz, CDCl$_3$)

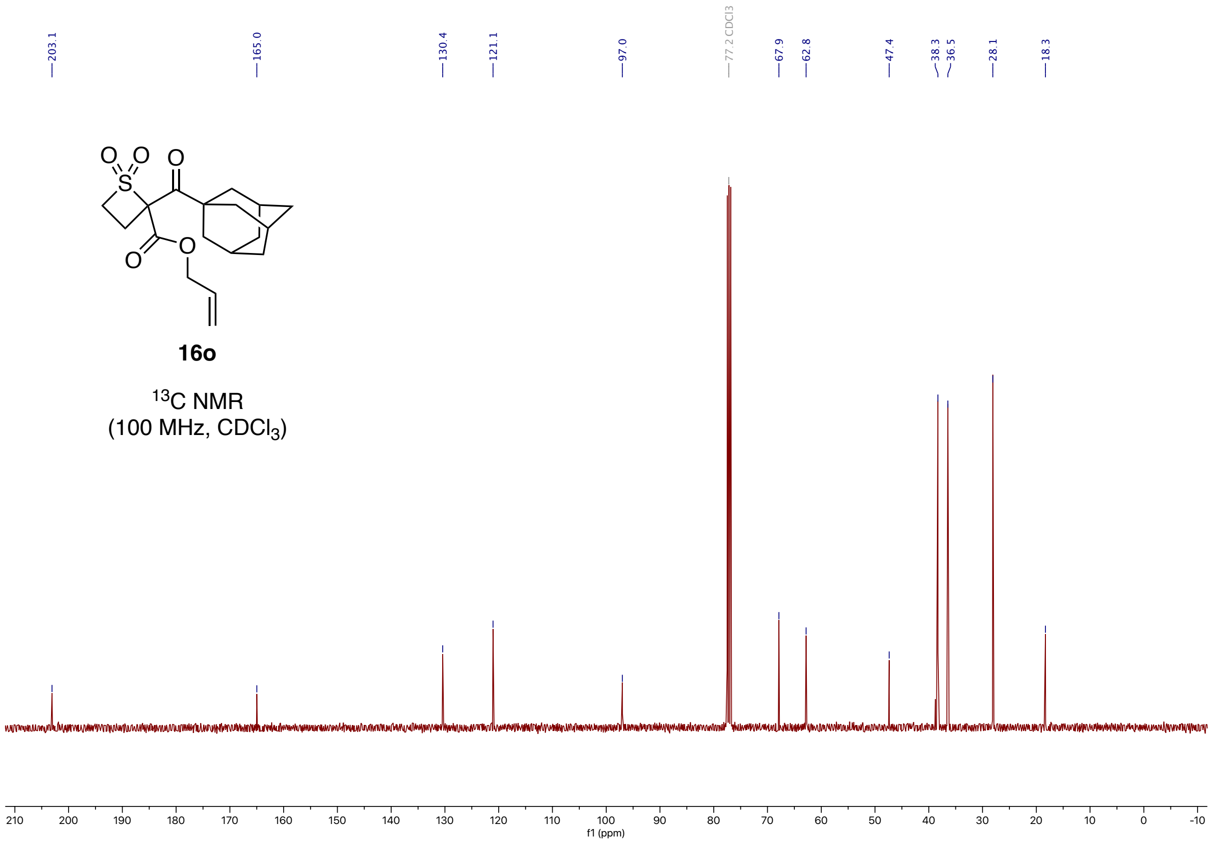

16o
^{13}C NMR
(100 MHz, CDCl$_3$)

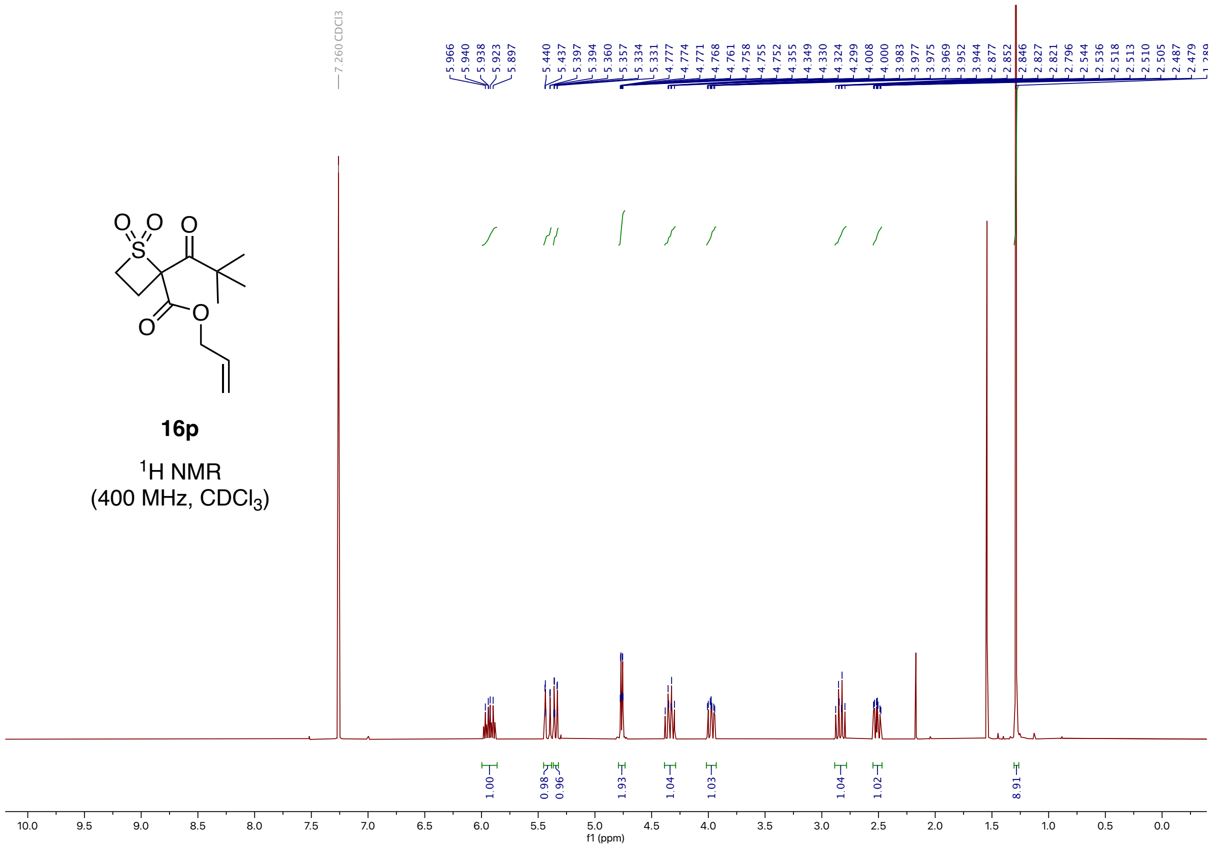
7.260 CDCl3
5.966
5.940
5.938
5.923
5.897
5.440
5.437
5.397
5.394
5.360
5.357
5.334
5.331
4.777
4.774
4.771
4.768
4.761
4.758
4.755
4.752
4.355
4.349
4.330
4.324
4.299
4.008
4.000
3.983
3.977
3.975
3.969
3.952
3.944
2.877
2.852
2.846
2.827
2.821
2.796
2.544
2.536
2.518
2.513
2.510
2.505
2.487
2.479
16p
1H NMR
(400 MHz, CDCl3)
1.00
0.98
0.96
1.93
1.04
1.03
1.04
1.02
8.91
f1 (ppm)

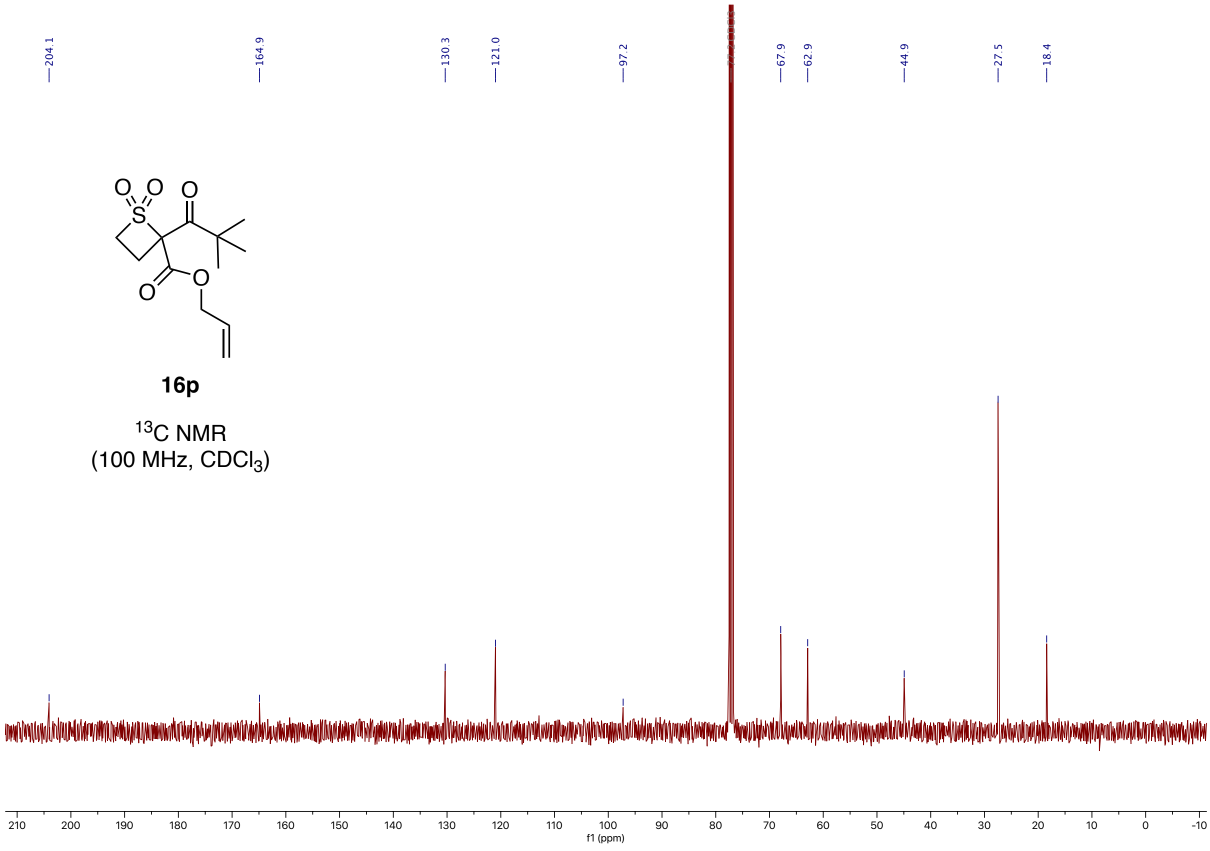
204.1
164.9
130.3
121.0
97.2
67.9
62.9
44.9
27.5
18.4
16p
13C NMR
(100 MHz, CDCl3)
f1 (ppm)

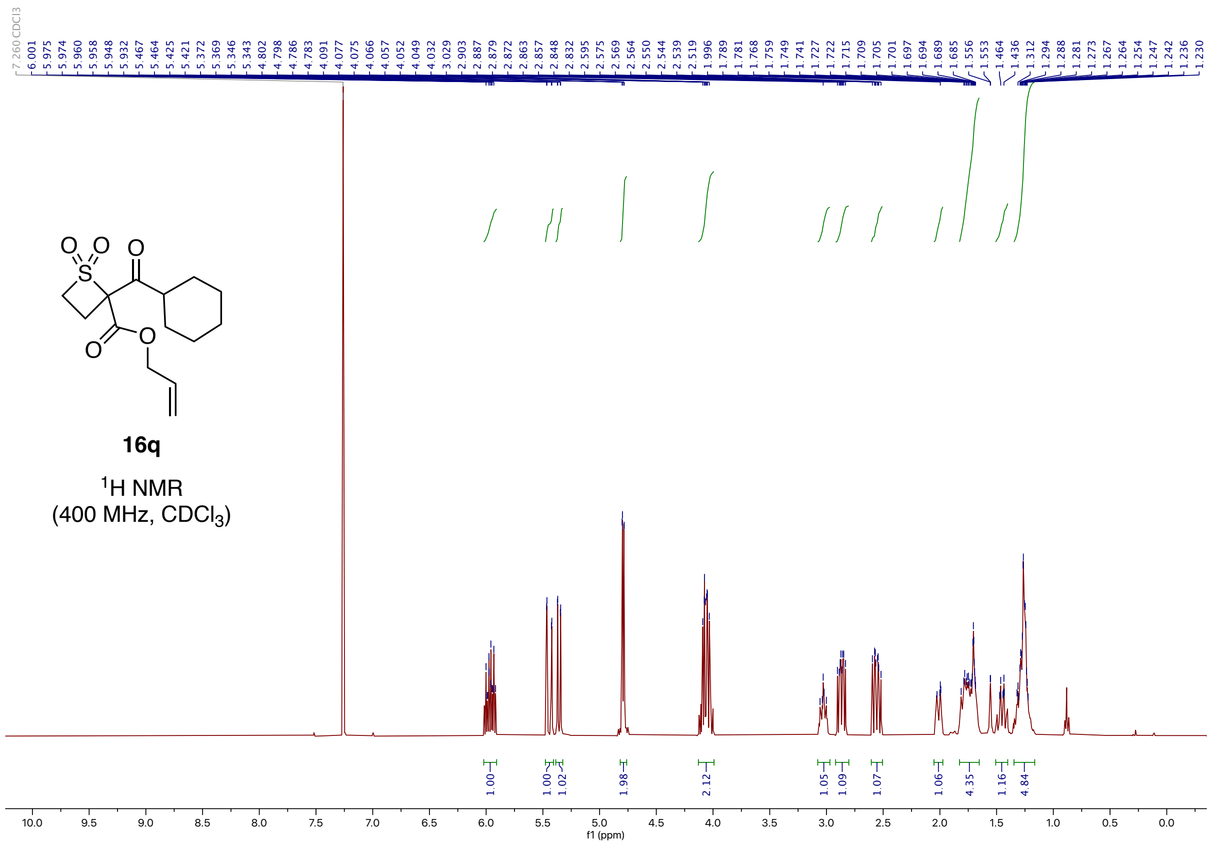
16q
1H NMR
(400 MHz, CDCl3)
f1 (ppm)

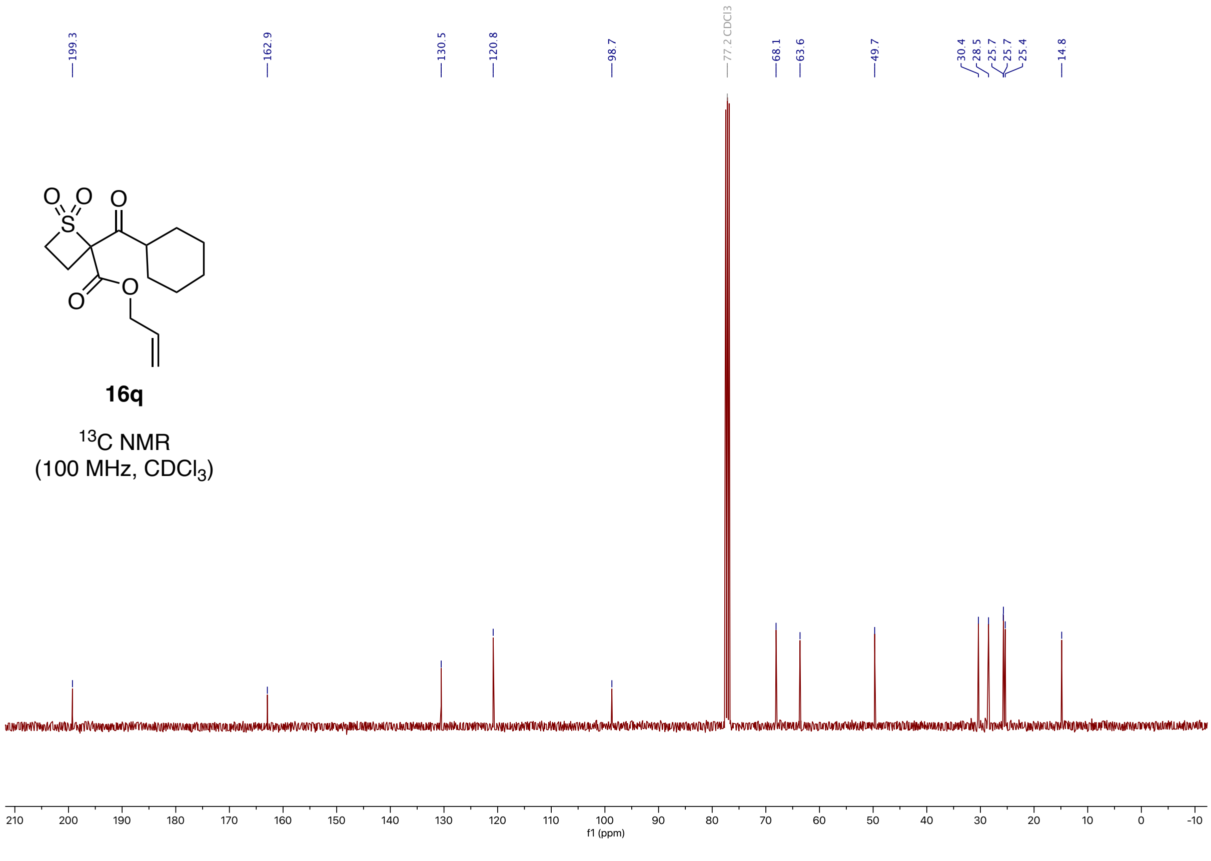
16q
13C NMR
(100 MHz, CDCl3)
f1 (ppm)

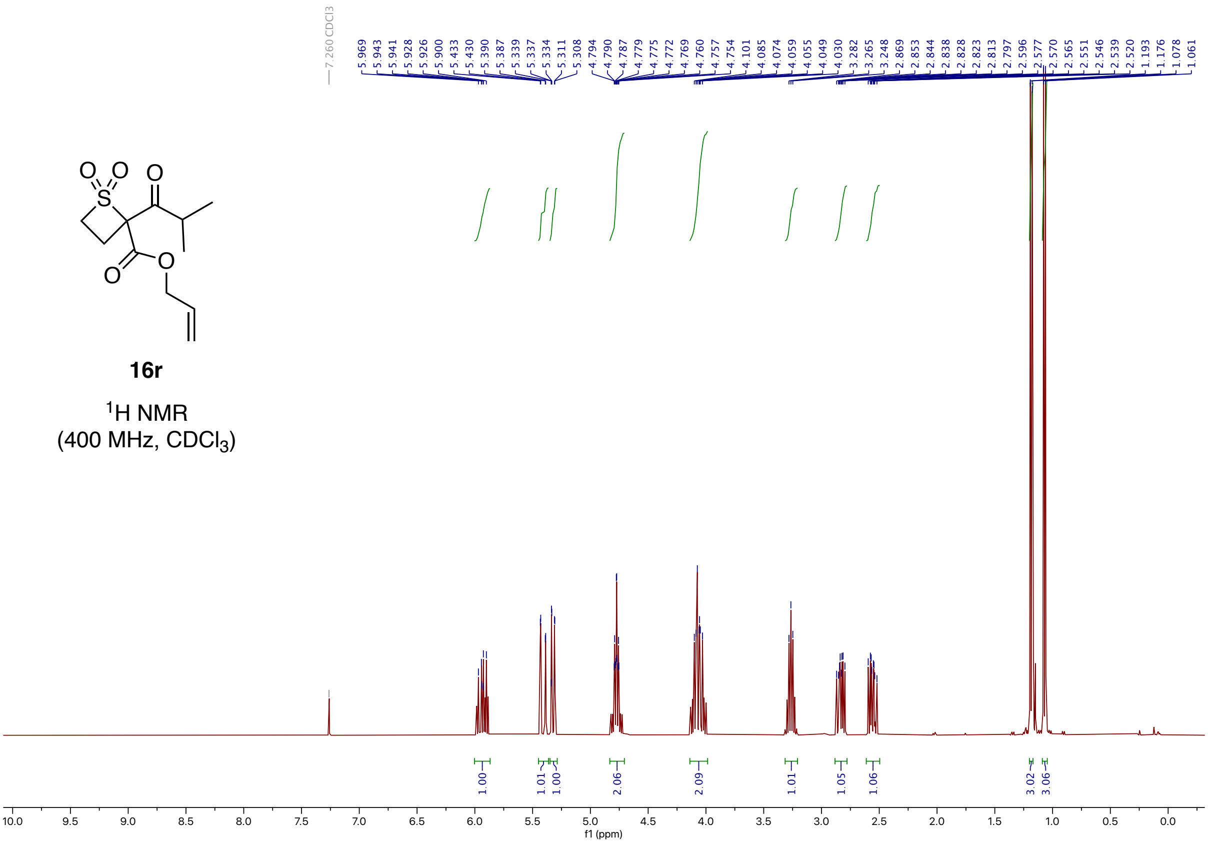

16r
^{1}H NMR
(400 MHz, $CDCl_3$)
7.260 CDCl3
5.969 5.943 5.941 5.928 5.926 5.900 5.433 5.430 5.390 5.387 5.339 5.337 5.334 5.311 5.308 4.794 4.790 4.787 4.779 4.775 4.772 4.769 4.760 4.757 4.754 4.101 4.085 4.074 4.059 4.055 4.049 4.030 3.282 3.265 3.248 2.869 2.853 2.844 2.838 2.828 2.823 2.813 2.797 2.596 2.577 2.570 2.565 2.551 2.546 2.539 2.520 1.193 1.176 1.078 1.061
1.00 1.01 1.00 2.06 2.09 1.01 1.05 1.06 3.02 3.06
f1 (ppm)

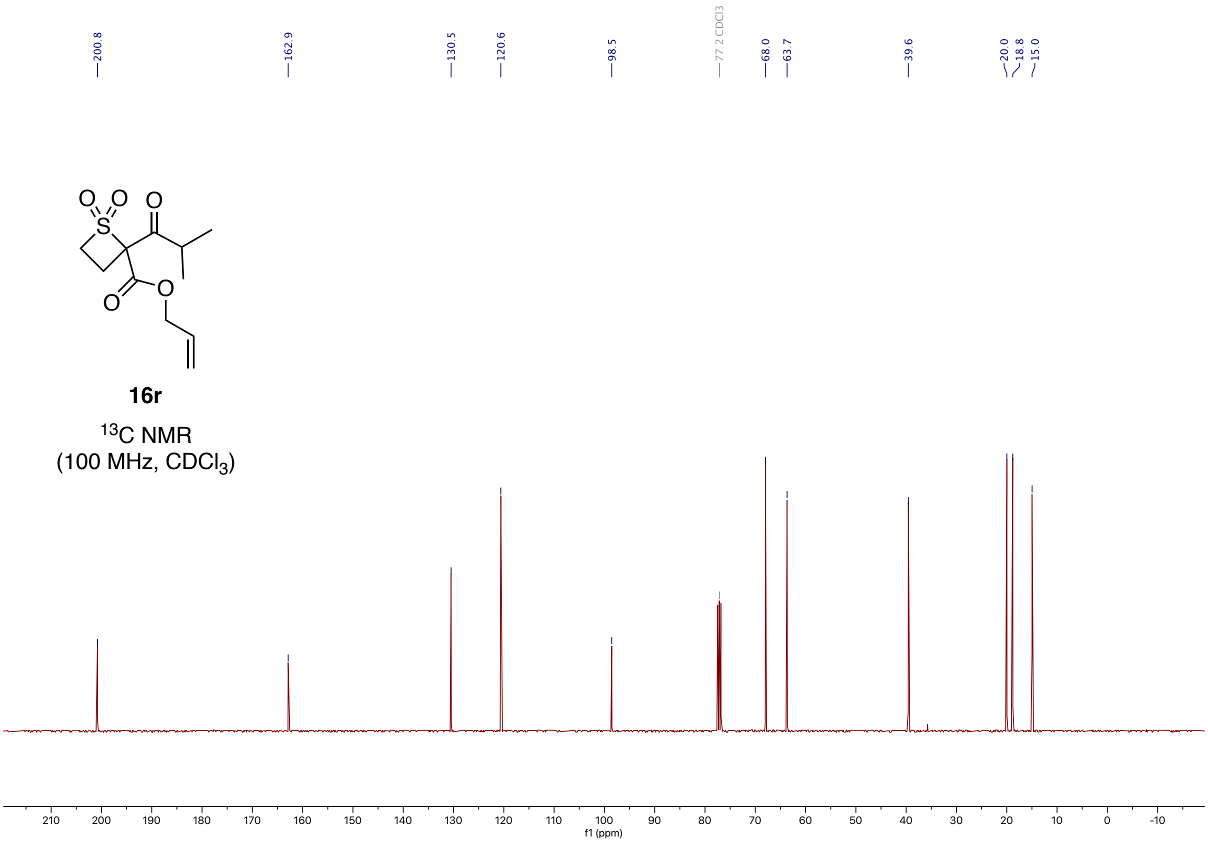

16r
^{13}C NMR
(100 MHz, $CDCl_3$)
200.8 162.9 130.5 120.6 98.5 77.2 CDCl3 68.0 63.7 39.6 20.0 18.8 15.0
f1 (ppm)

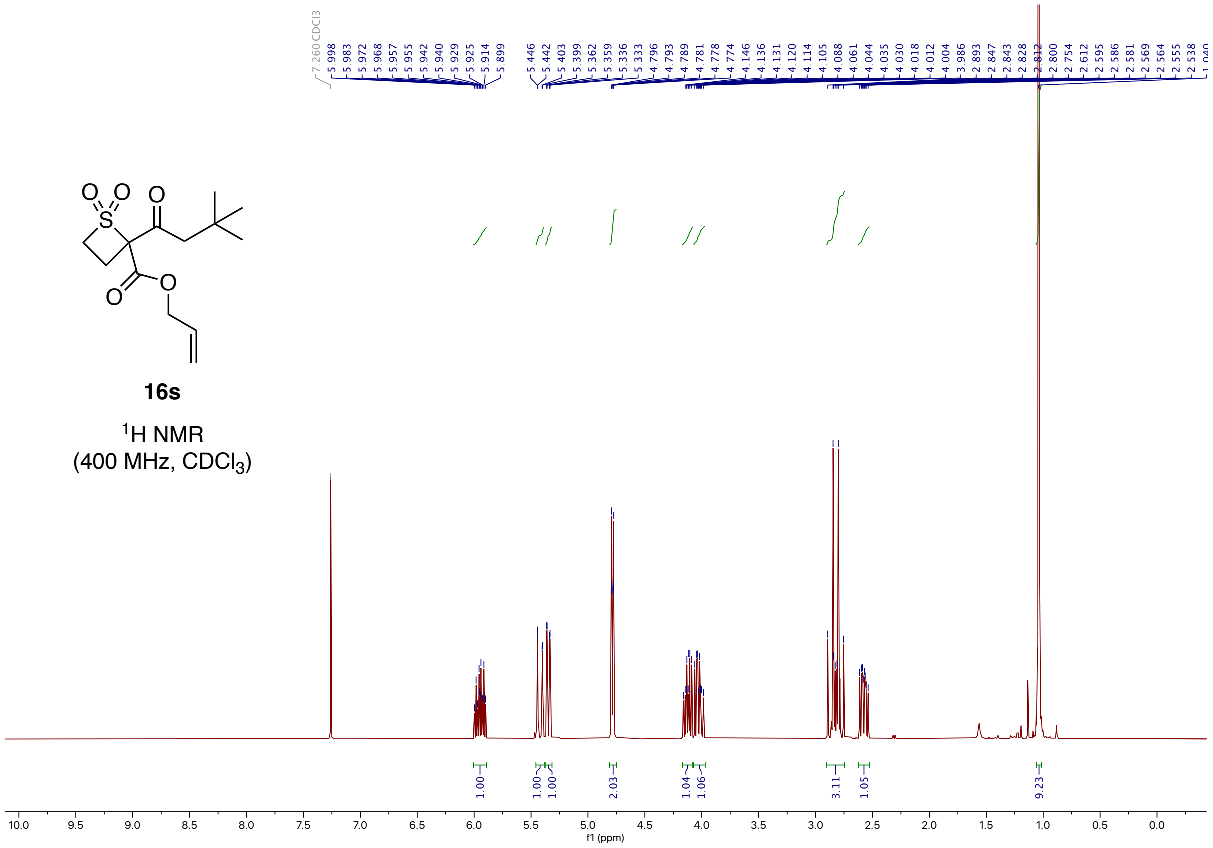

16s

^{1}H NMR
(400 MHz, $CDCl_3$)

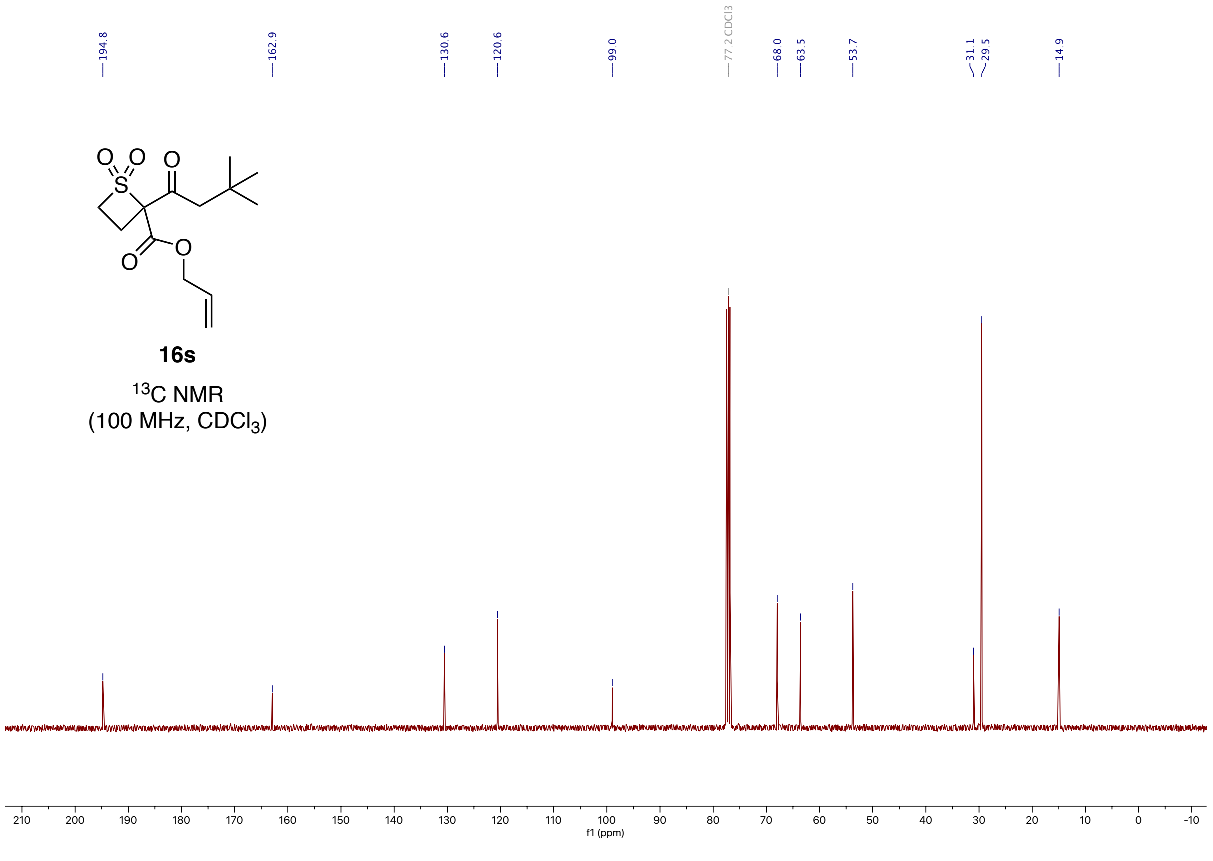

16s

^{13}C NMR
(100 MHz, $CDCl_3$)

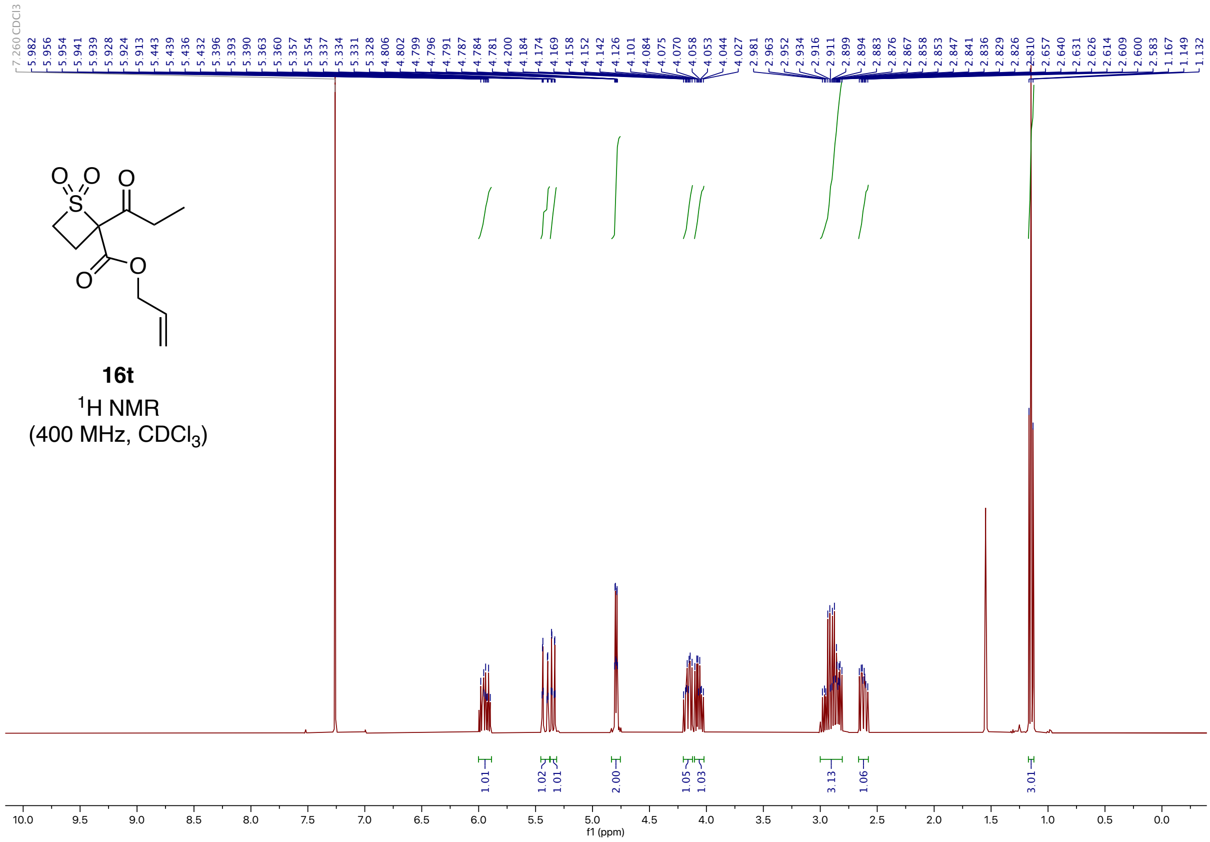
7.260 CDCl3
5.982
5.956
5.954
5.941
5.939
5.928
5.924
5.913
5.443
5.439
5.436
5.432
5.396
5.393
5.390
5.363
5.360
5.357
5.354
5.337
5.334
5.331
5.328
4.806
4.802
4.799
4.796
4.791
4.787
4.784
4.781
4.200
4.184
4.174
4.169
4.158
4.152
4.142
4.126
4.101
4.084
4.075
4.070
4.058
4.053
4.044
4.027
2.981
2.963
2.952
2.934
2.916
2.911
2.899
2.894
2.883
2.876
2.867
2.858
2.853
2.847
2.841
2.836
2.829
2.826
2.810
2.657
2.640
2.631
2.626
2.614
2.609
2.600
2.583
1.167
1.149
1.132
16t
^{1}H NMR
(400 MHz, CDCl$_3$)
1.01
1.02
1.01
2.00
1.05
1.03
3.13
1.06
3.01
f1 (ppm)

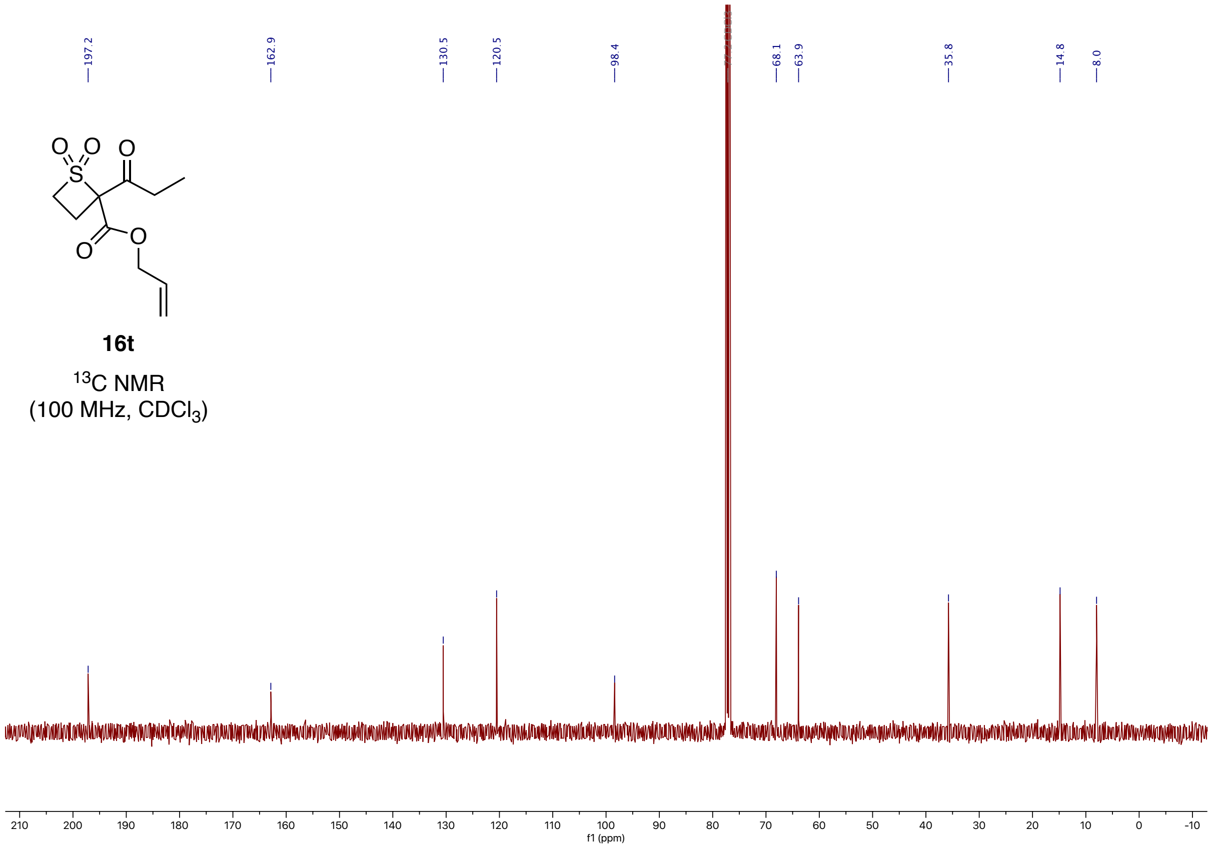
197.2
162.9
130.5
120.5
98.4
68.1
63.9
35.8
14.8
8.0
16t
^{13}C NMR
(100 MHz, CDCl$_3$)
f1 (ppm)

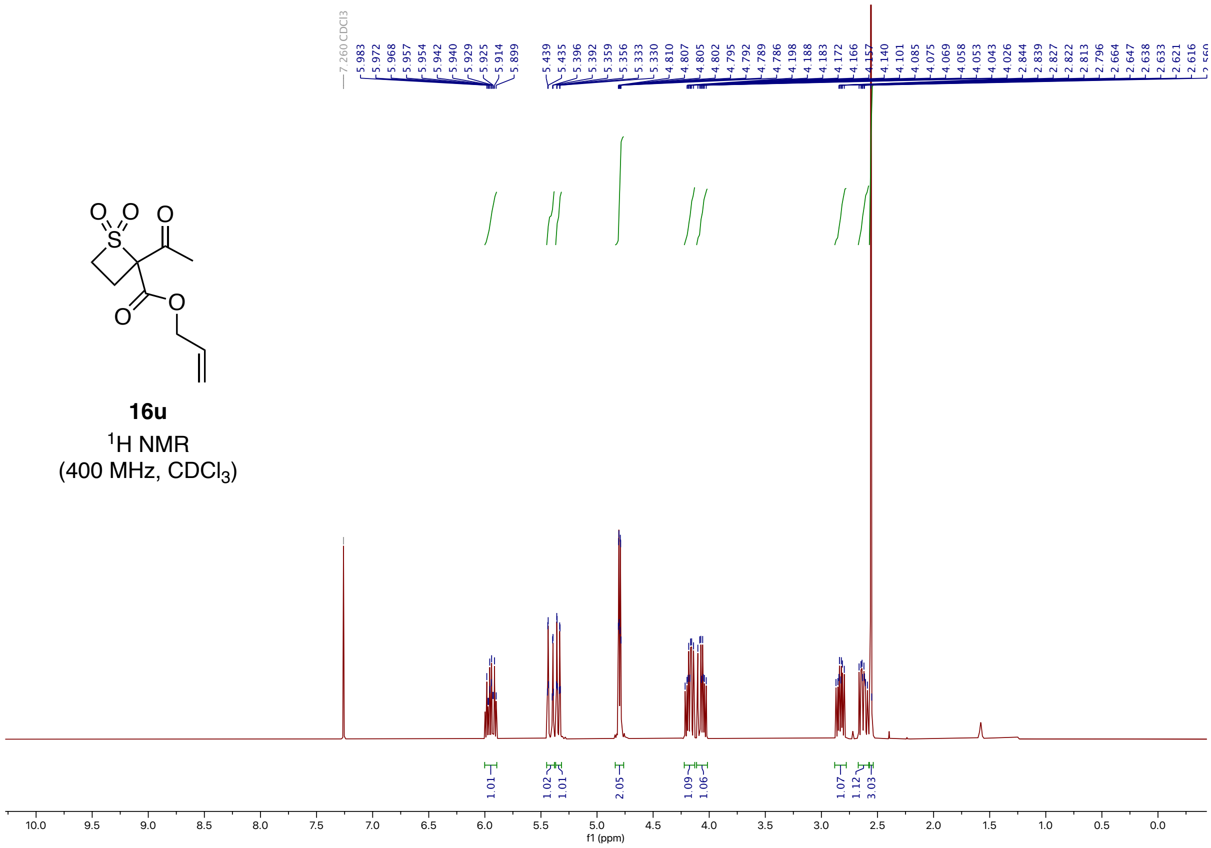

16u

^{1}H NMR
(400 MHz, $CDCl_3$)

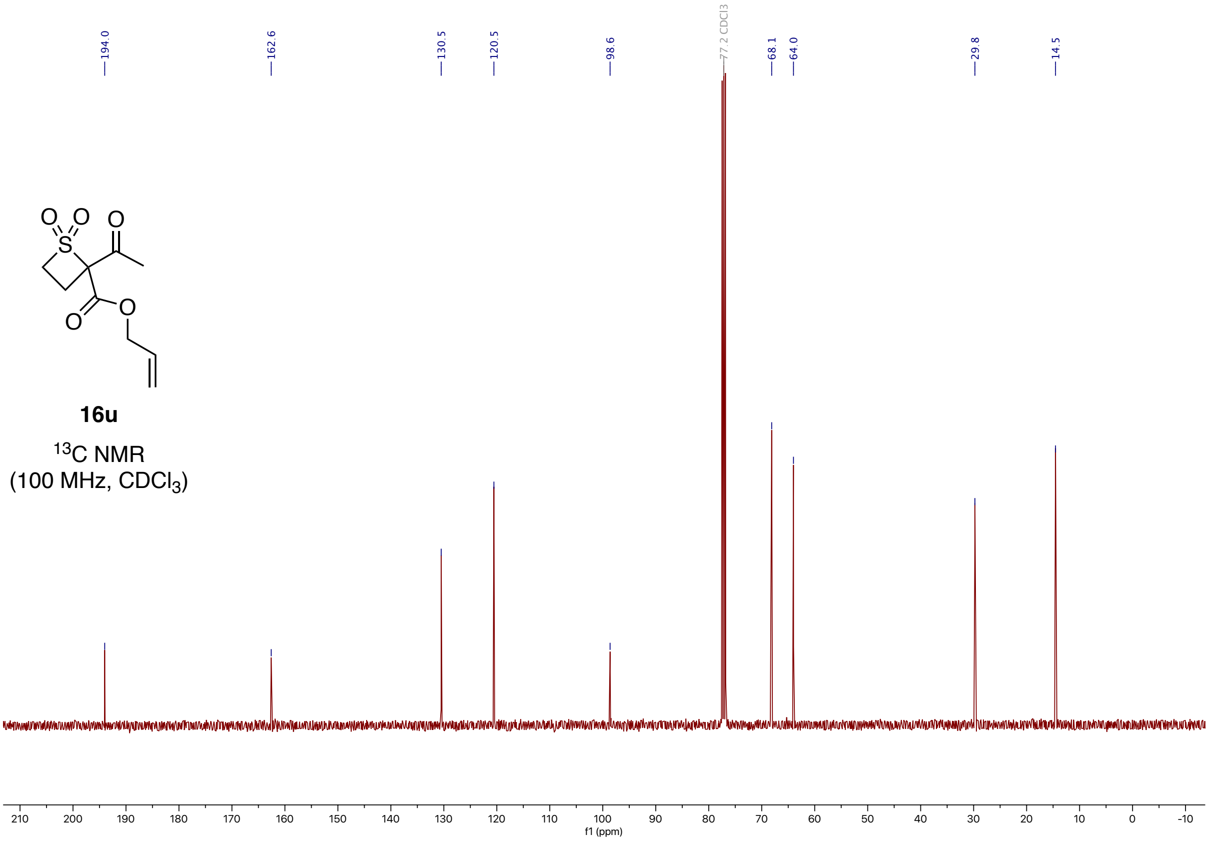

16u

^{13}C NMR
(100 MHz, $CDCl_3$)

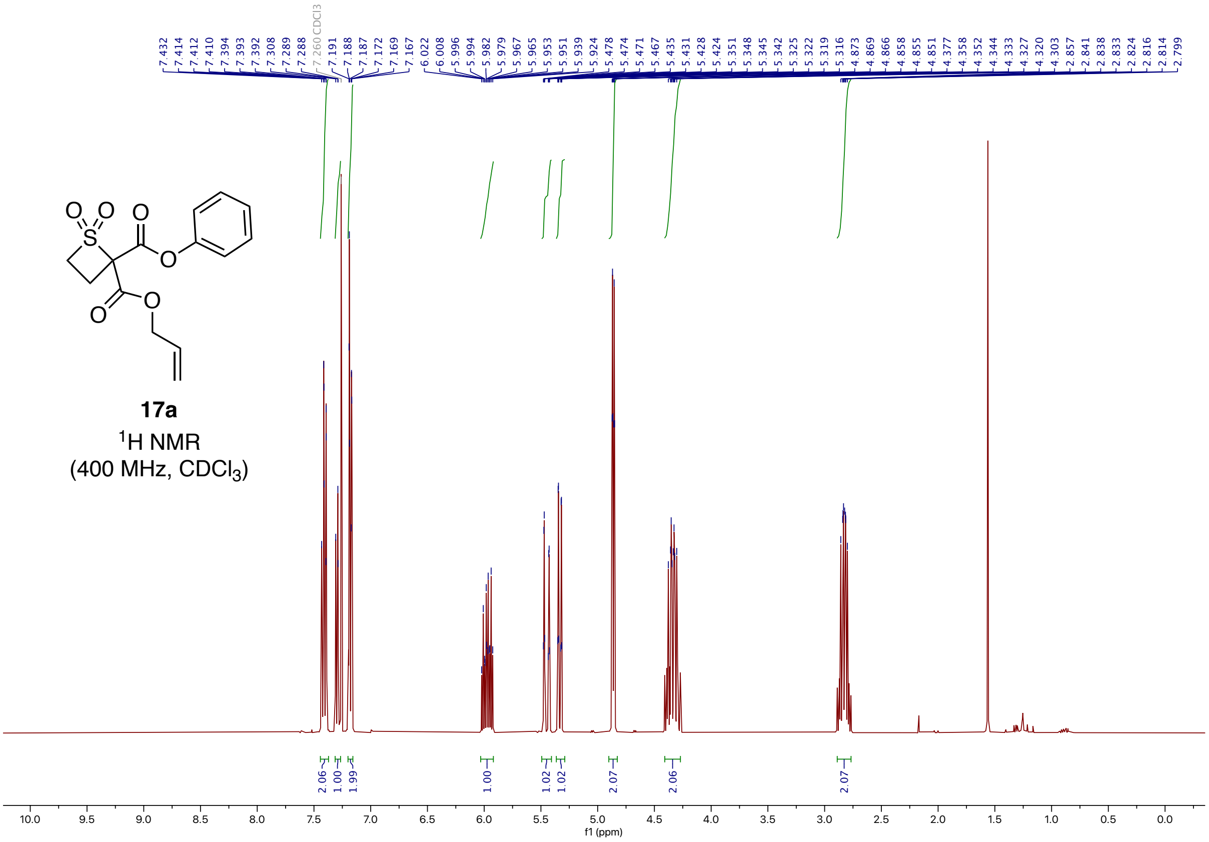
7.432
7.414
7.412
7.410
7.394
7.393
7.392
7.308
7.289
7.288
7.260 CDCl3
7.191
7.188
7.187
7.172
7.169
7.167
6.022
6.008
5.996
5.994
5.982
5.979
5.967
5.965
5.953
5.951
5.939
5.924
5.478
5.474
5.471
5.467
5.435
5.431
5.428
5.424
5.351
5.348
5.345
5.342
5.325
5.322
5.319
5.316
4.873
4.869
4.866
4.858
4.855
4.851
4.377
4.358
4.352
4.344
4.333
4.327
4.320
4.303
2.857
2.841
2.838
2.833
2.824
2.816
2.814
2.799
17a
1H NMR
(400 MHz, CDCl3)
2.06
1.00
1.99
1.00
1.02
1.02
2.07
2.06
2.07
f1 (ppm)

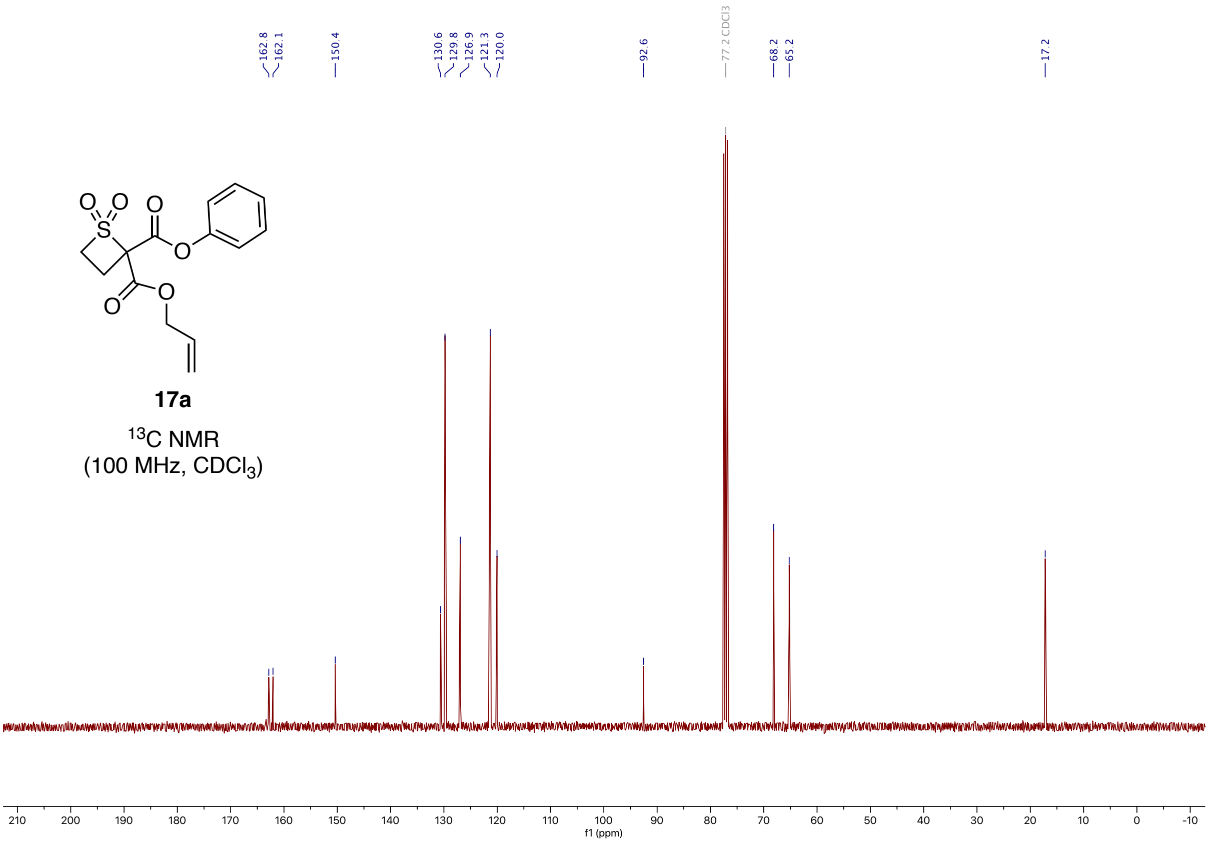
162.8
162.1
150.4
130.6
129.8
126.9
121.3
120.0
92.6
77.2 CDCl3
68.2
65.2
17.2
17a
13C NMR
(100 MHz, CDCl3)
f1 (ppm)

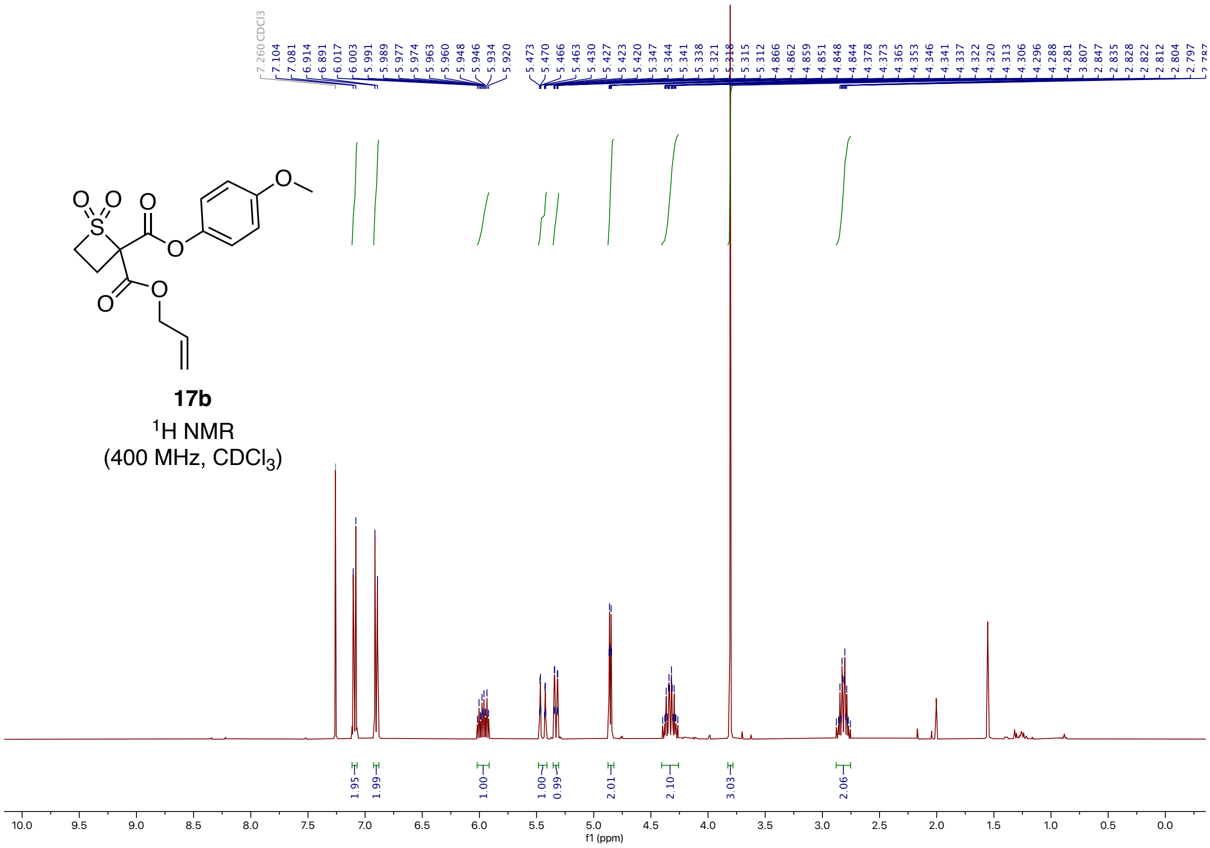
7.260 CDCl3
7.104
7.081
6.914
6.891
6.017
6.003
5.991
5.989
5.977
5.974
5.963
5.960
5.948
5.946
5.934
5.920
5.473
5.470
5.466
5.463
5.430
5.427
5.423
5.420
5.347
5.344
5.341
5.338
5.321
5.318
5.315
5.312
4.866
4.862
4.859
4.851
4.848
4.844
4.378
4.373
4.365
4.353
4.346
4.341
4.337
4.322
4.320
4.313
4.306
4.296
4.288
4.281
3.807
2.847
2.835
2.828
2.822
2.812
2.804
2.797
17b
1H NMR
(400 MHz, CDCl3)
1.95
1.99
1.00
1.00
0.99
2.01
2.10
3.03
2.06
f1 (ppm)

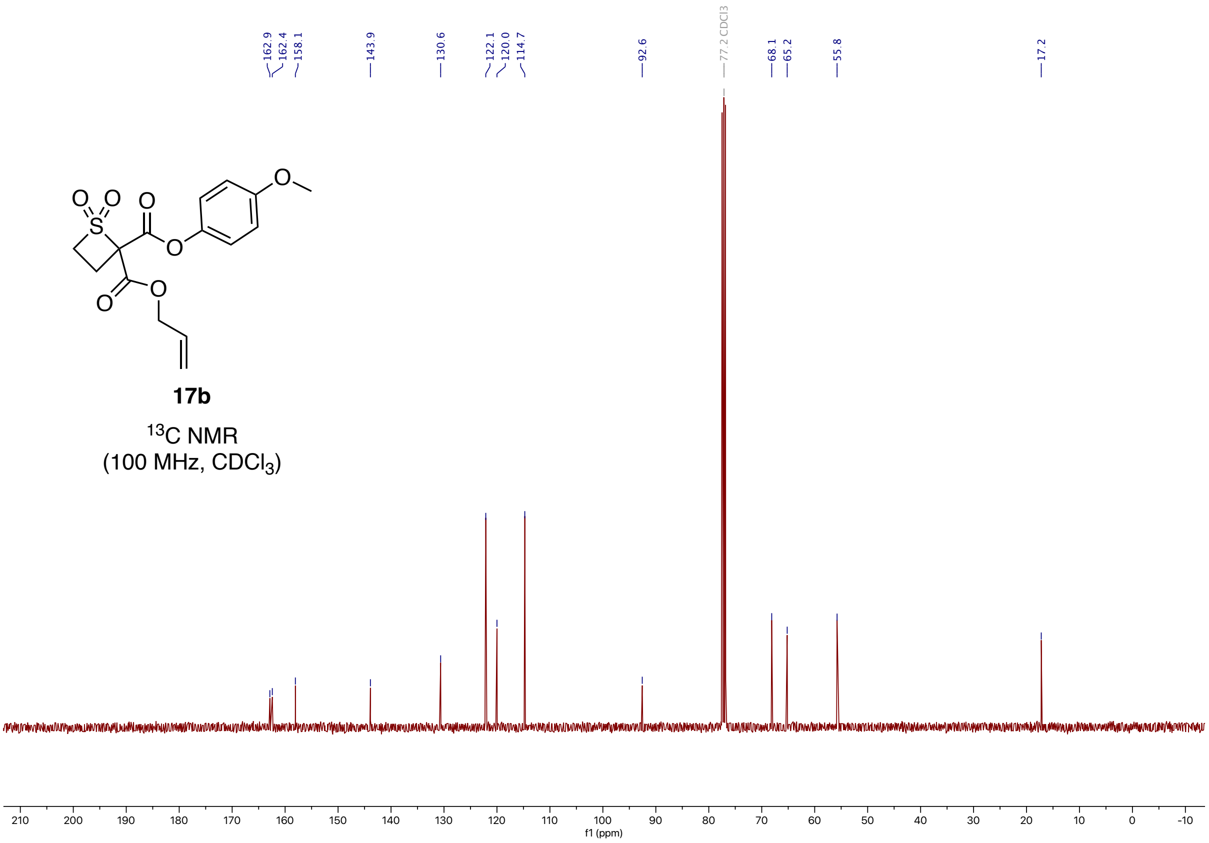
162.9
162.4
158.1
143.9
130.6
122.1
120.0
114.7
92.6
77.2 CDCl3
68.1
65.2
55.8
17.2
17b
13C NMR
(100 MHz, CDCl3)
f1 (ppm)

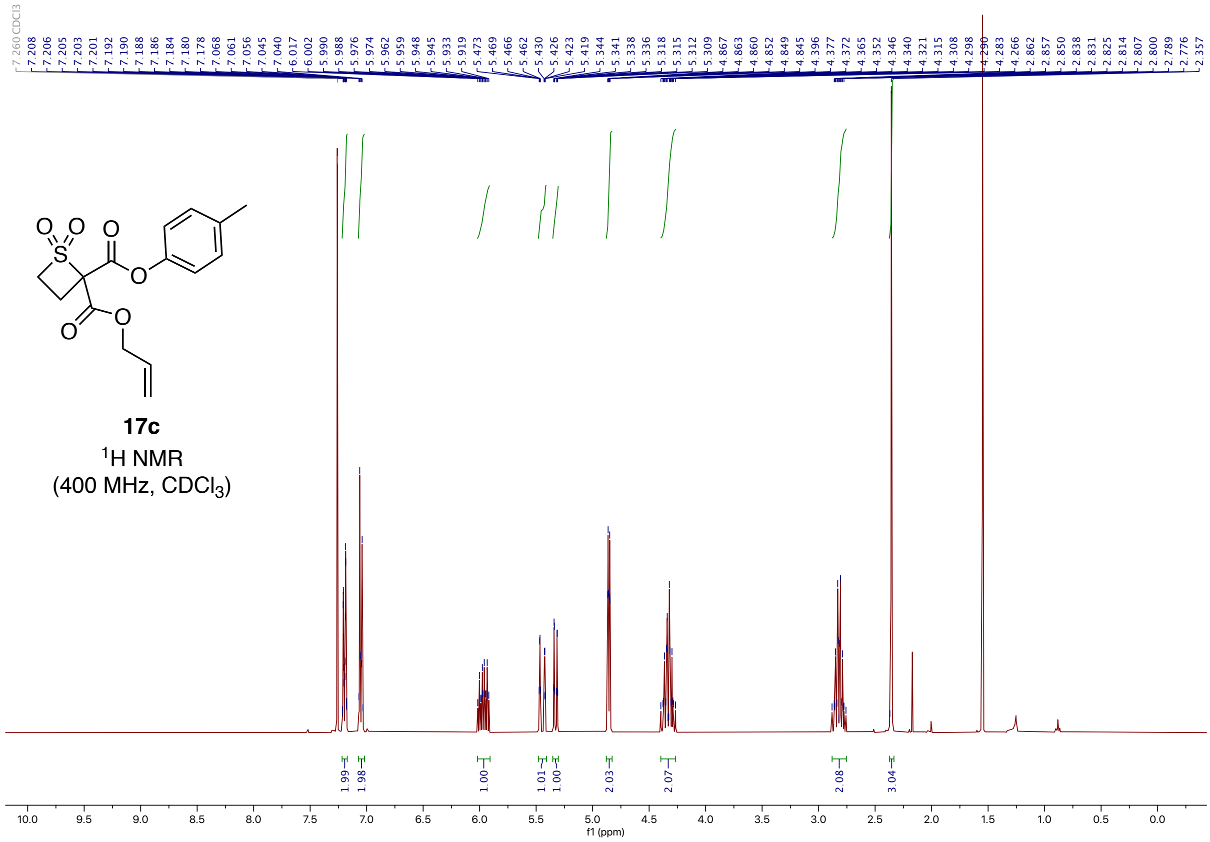
17c
1H NMR
(400 MHz, CDCl3)
f1 (ppm)

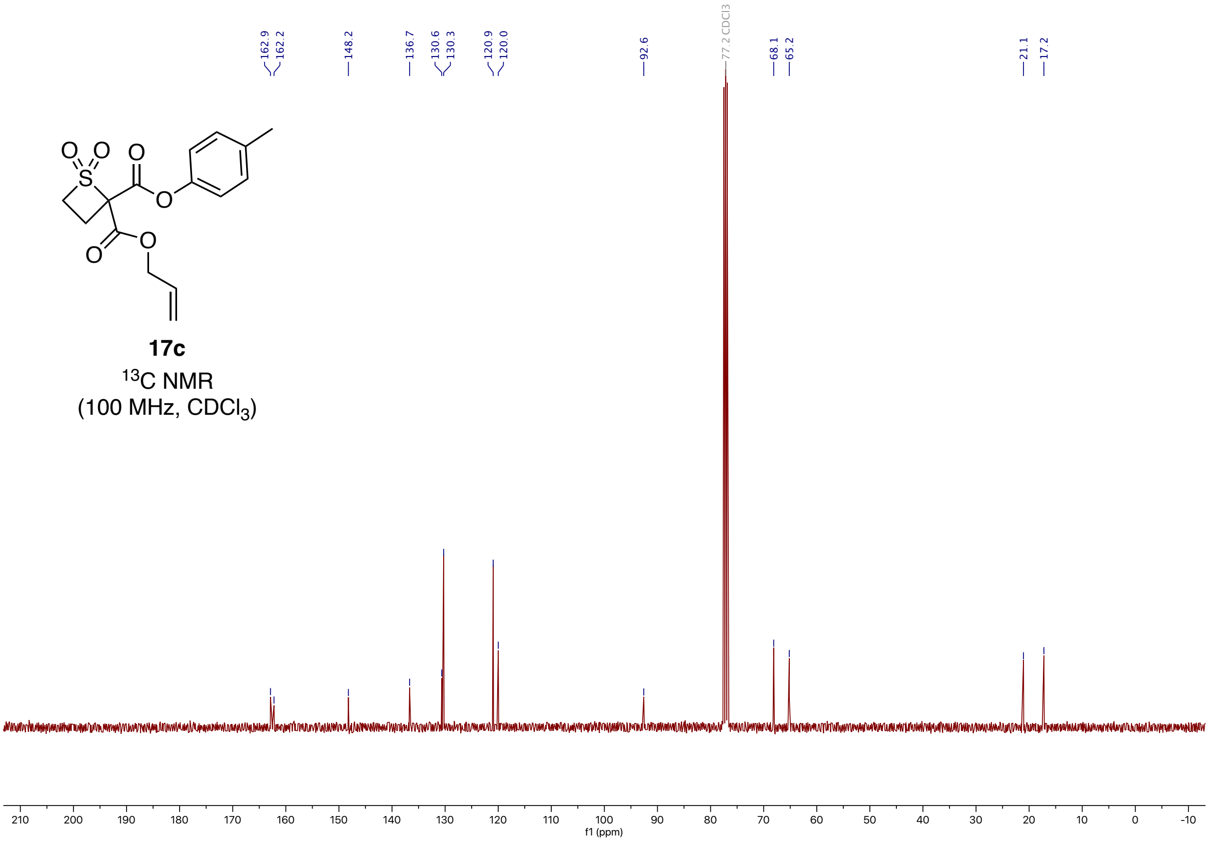
17c
13C NMR
(100 MHz, CDCl3)
162.9
162.2
148.2
136.7
130.6
130.3
120.9
120.0
92.6
77.2 CDCl3
68.1
65.2
21.1
17.2
f1 (ppm)

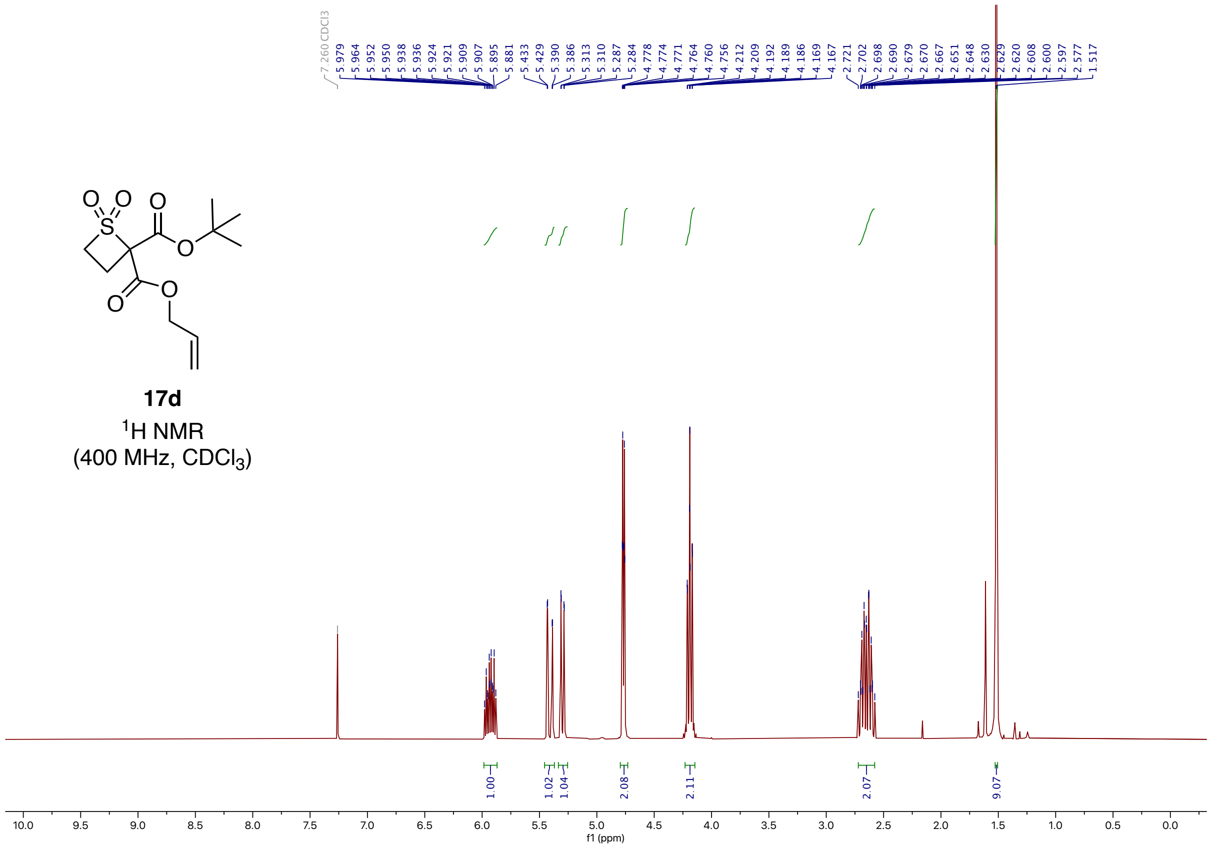
7.260 CDCl3
5.979
5.964
5.952
5.950
5.938
5.936
5.924
5.921
5.909
5.907
5.895
5.881
5.433
5.429
5.390
5.386
5.313
5.310
5.287
5.284
4.778
4.774
4.771
4.764
4.760
4.756
4.212
4.209
4.192
4.189
4.186
4.169
4.167
2.721
2.702
2.698
2.690
2.679
2.670
2.667
2.651
2.648
2.630
2.629
2.620
2.608
2.600
2.597
2.577
1.517
17d
1H NMR
(400 MHz, CDCl3)
1.00
1.02
1.04
2.08
2.11
2.07
9.07
f1 (ppm)

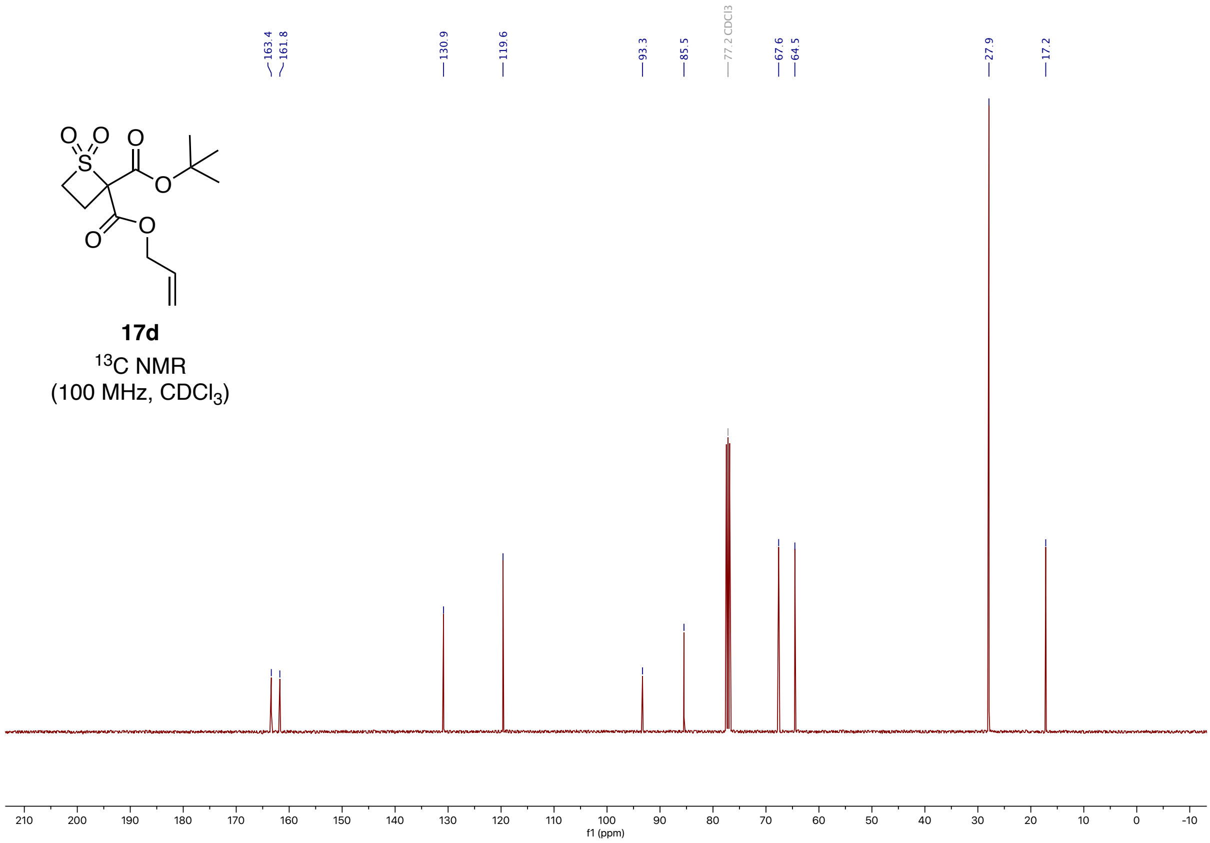
163.4
161.8
130.9
119.6
93.3
85.5
77.2 CDCl3
67.6
64.5
27.9
17.2
17d
13C NMR
(100 MHz, CDCl3)
f1 (ppm)

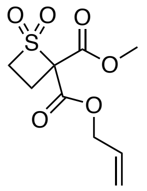

17e
^{1}H NMR
(400 MHz, $CDCl_3$)

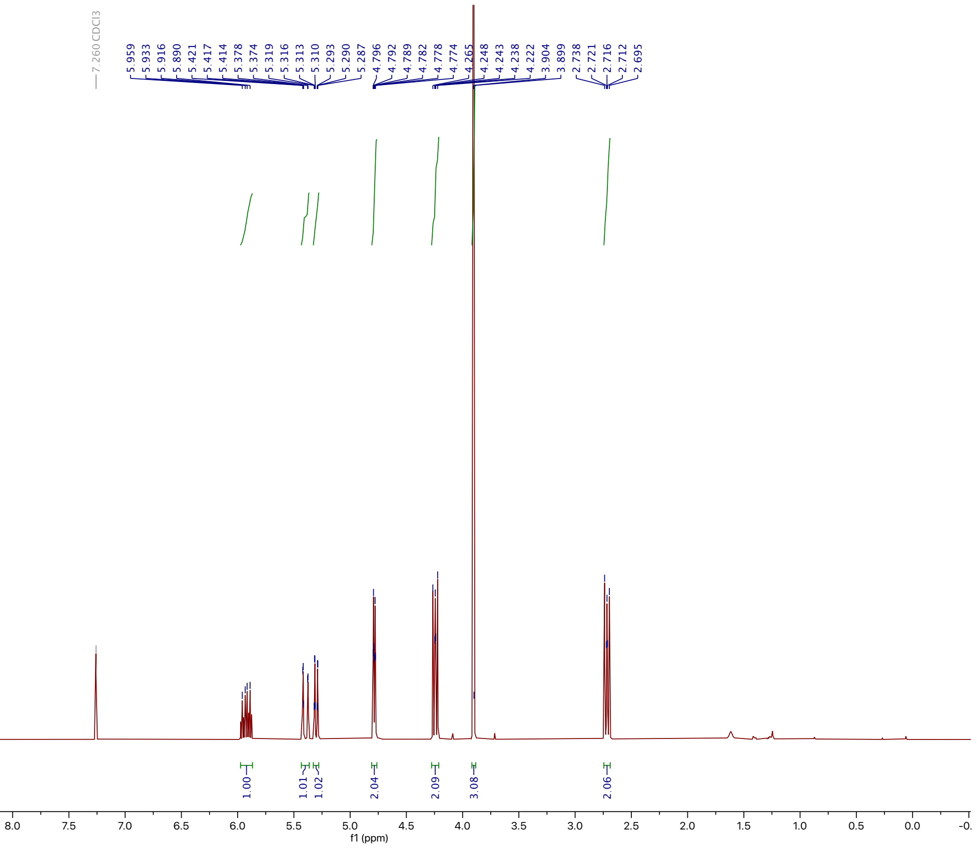

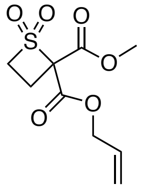

17e
^{13}C NMR
(100 MHz, $CDCl_3$)

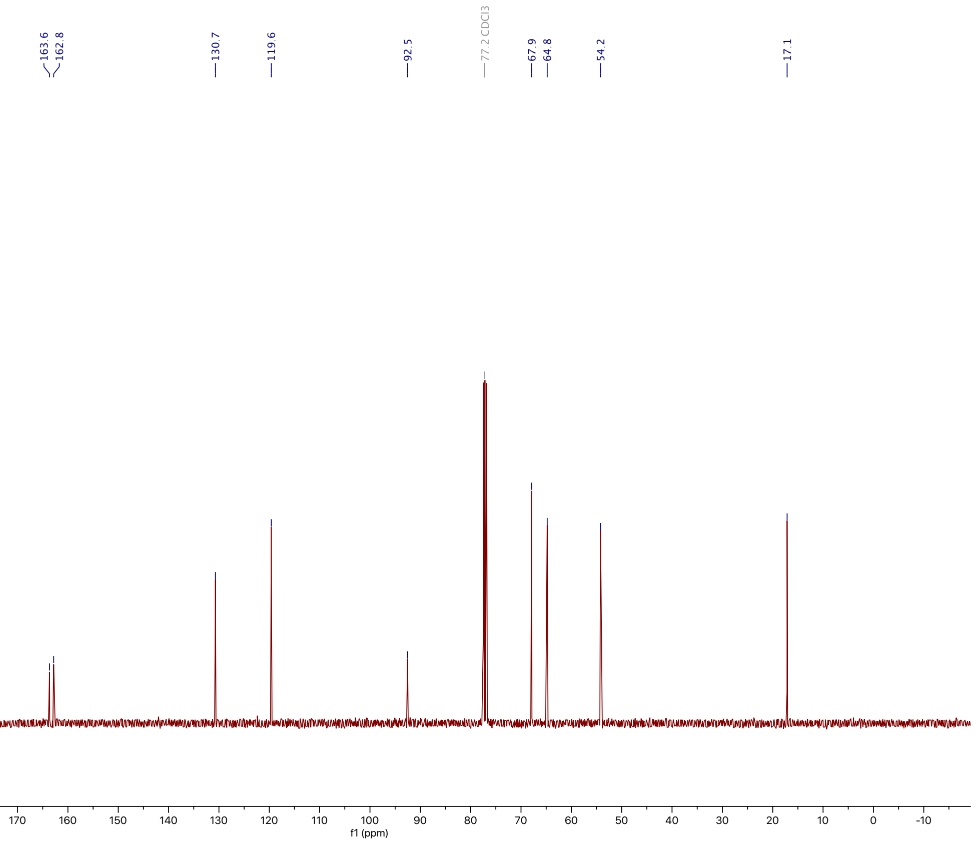

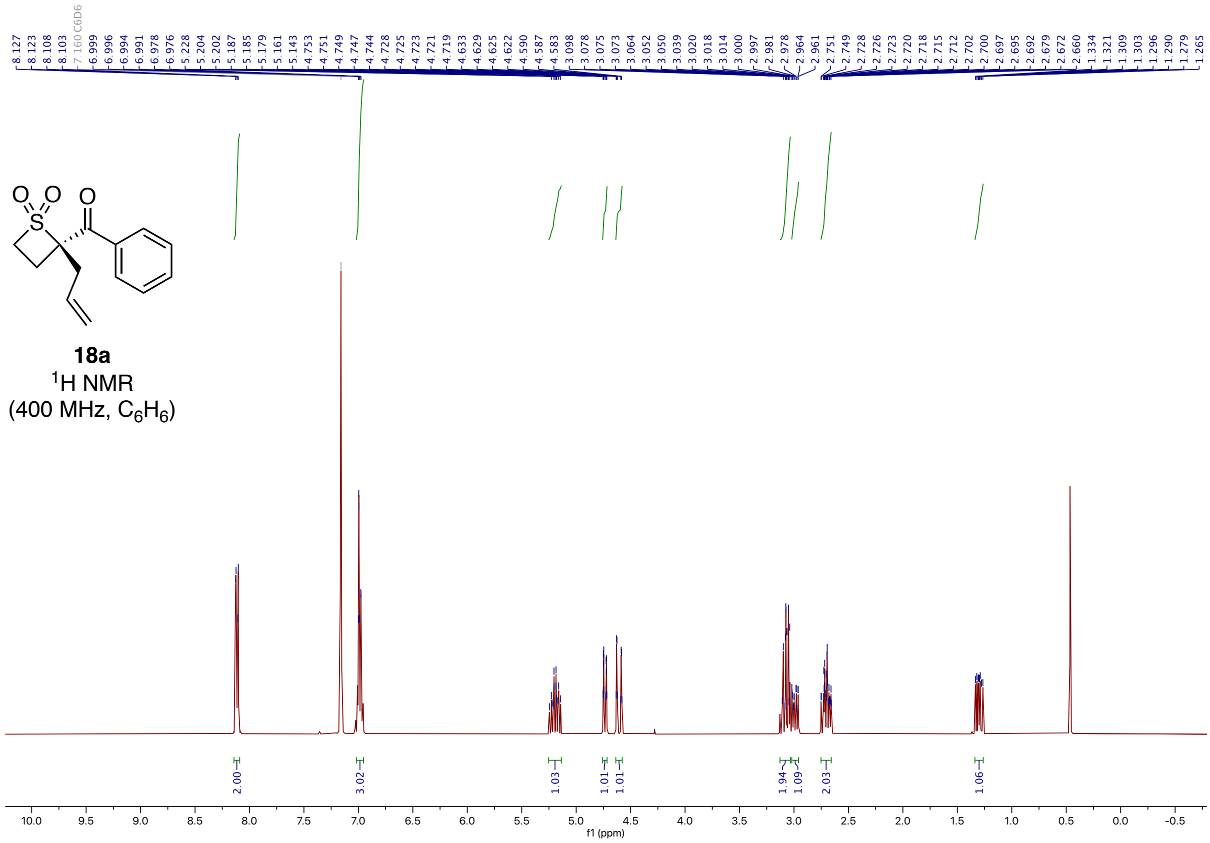

18a
^{1}H NMR
(400 MHz, C_6H_6)

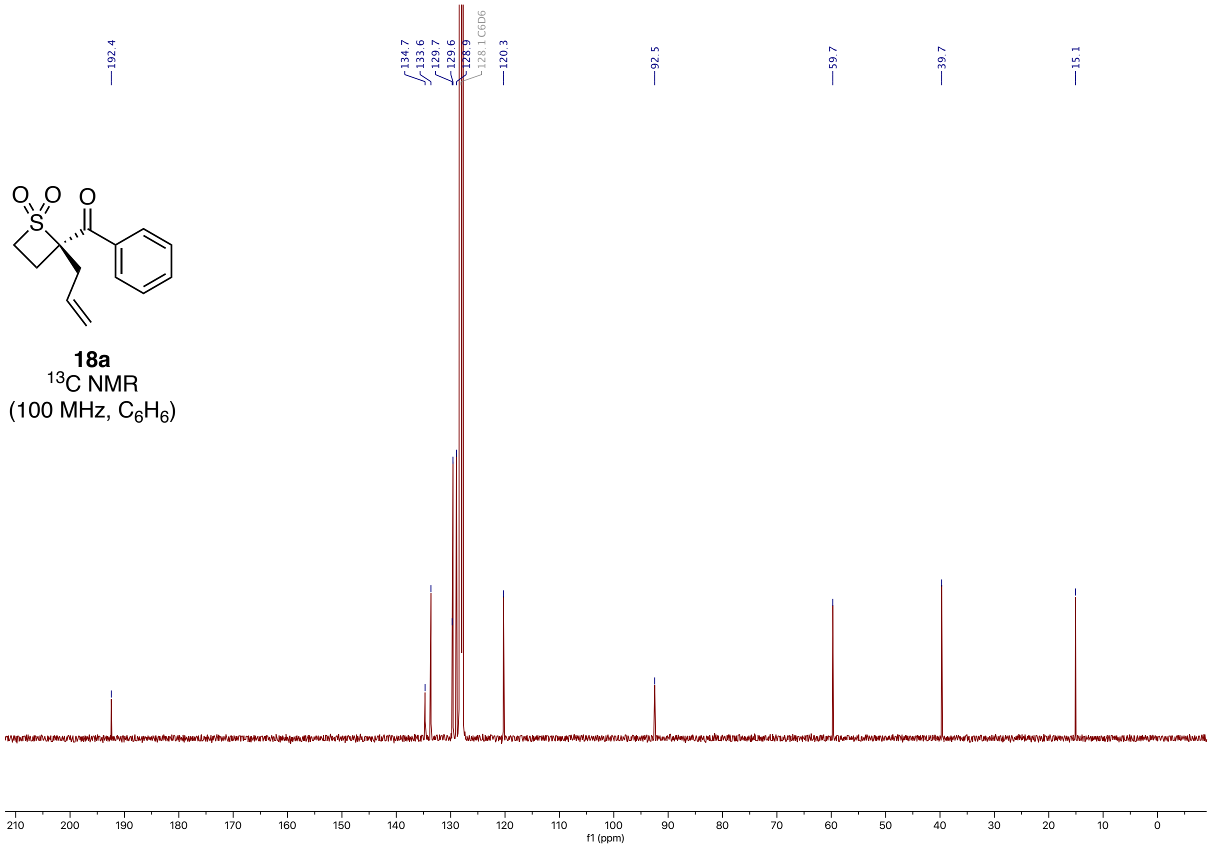

18a
^{13}C NMR
(100 MHz, C_6H_6)

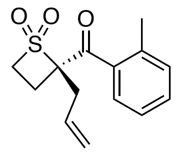

18b
^{1}H NMR
(400 MHz, $CDCl_3$)

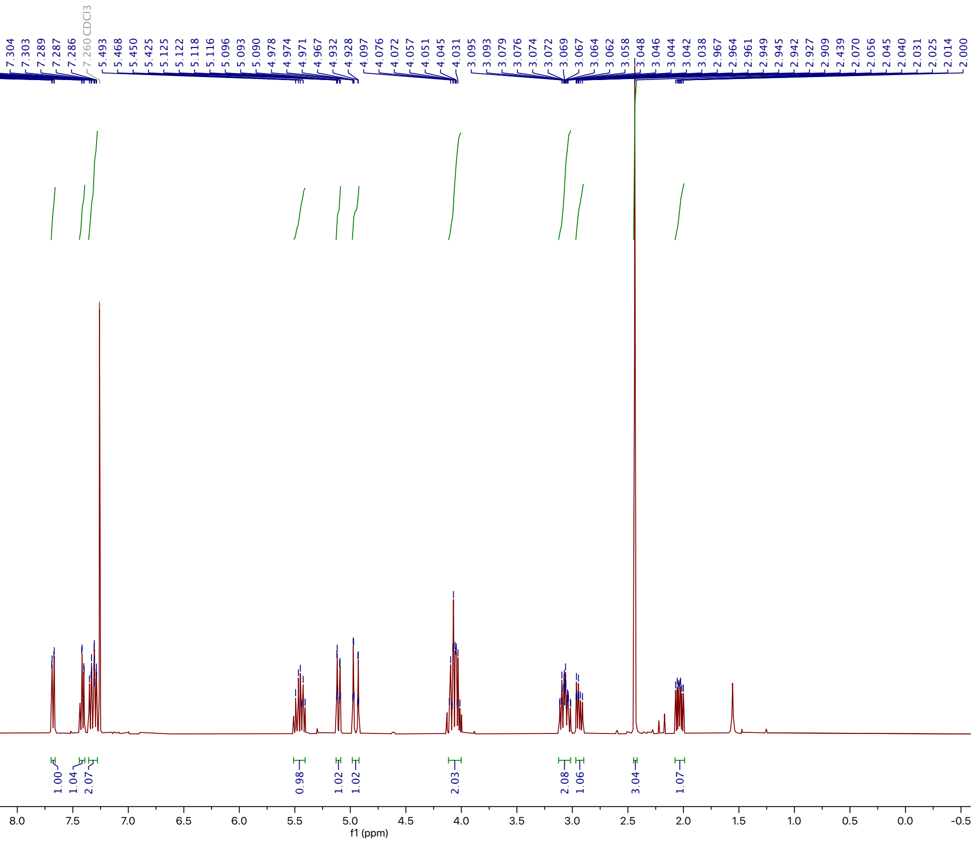

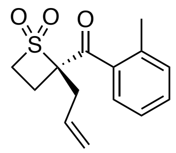

18b
^{13}C NMR
(100 MHz, $CDCl_3$)

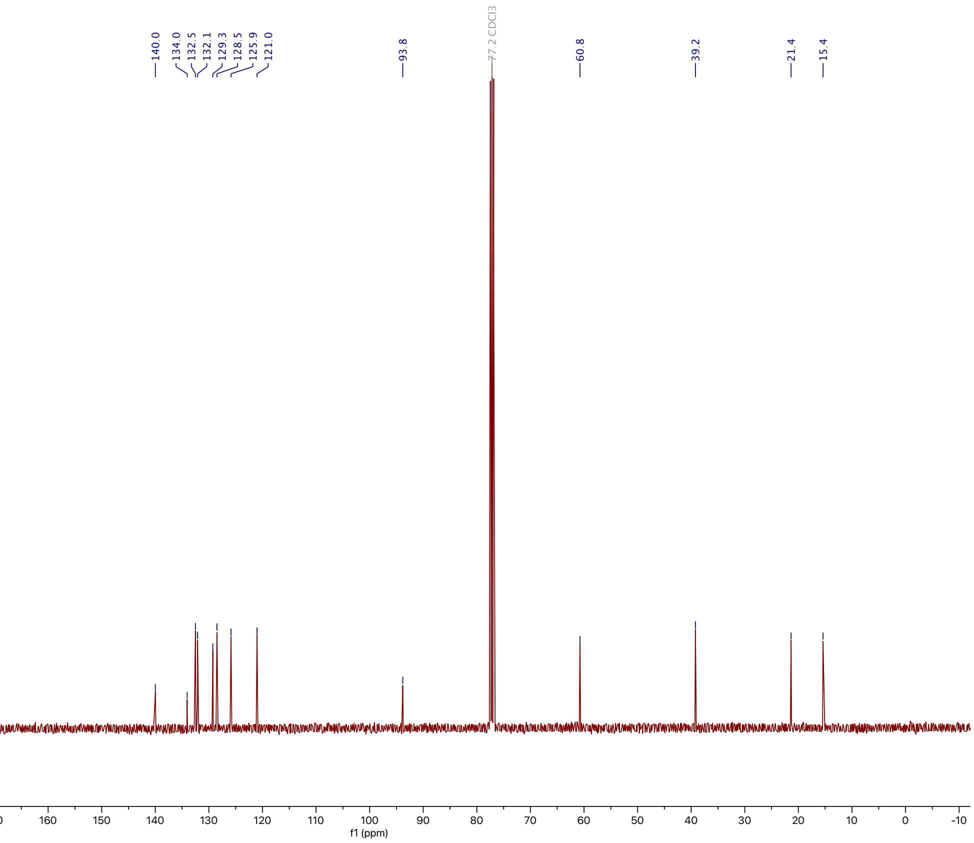

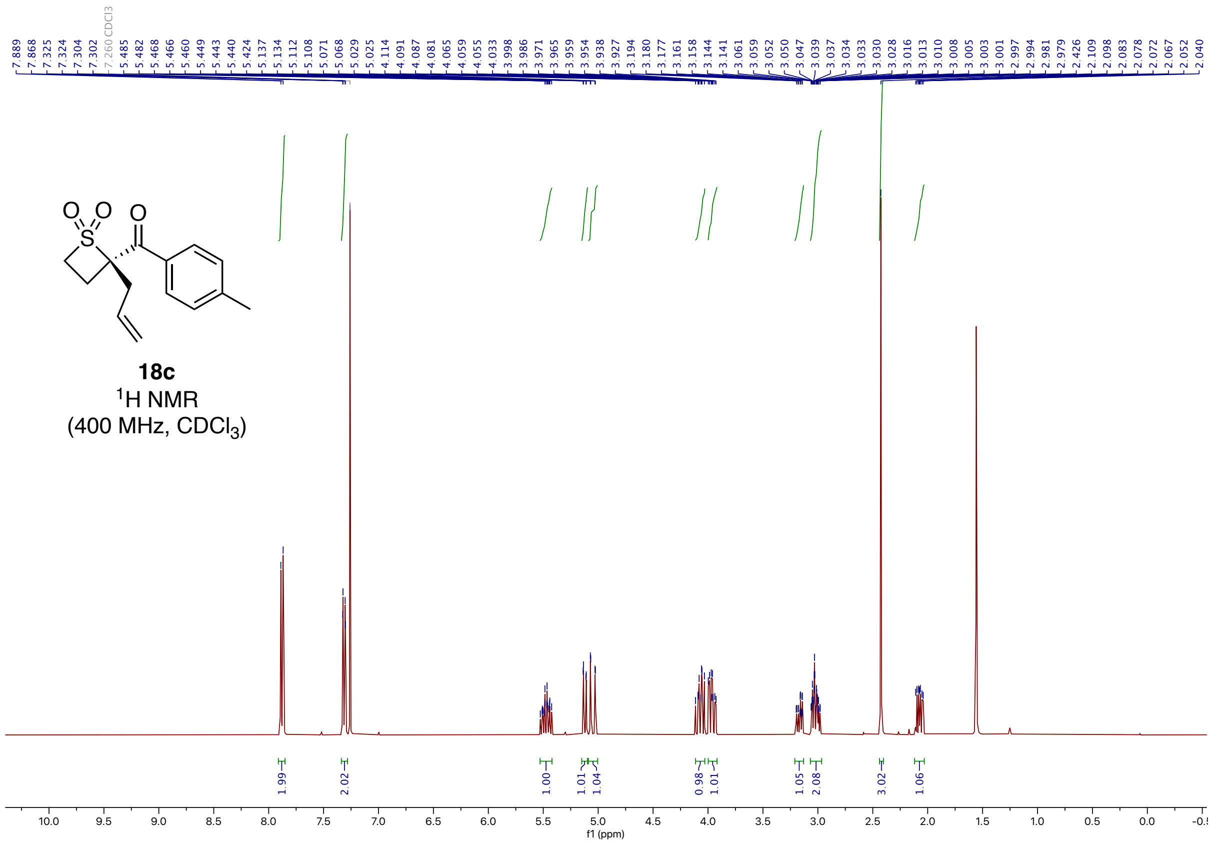
7.889
7.868
7.325
7.324
7.304
7.302
7.260 CDCl3
5.485
5.482
5.468
5.466
5.460
5.449
5.443
5.440
5.424
5.137
5.134
5.112
5.108
5.071
5.068
5.029
5.025
4.114
4.091
4.087
4.081
4.065
4.059
4.055
4.033
3.998
3.986
3.971
3.965
3.959
3.954
3.938
3.927
3.194
3.180
3.177
3.161
3.158
3.144
3.141
3.061
3.059
3.052
3.050
3.047
3.039
3.037
3.034
3.033
3.030
3.028
3.016
3.013
3.010
3.008
3.005
3.003
3.001
2.997
2.994
2.981
2.979
2.426
2.109
2.098
2.083
2.078
2.072
2.067
2.052
2.040
O O O
S
18c
1H NMR
(400 MHz, CDCl3)
1.99
2.02
1.00
1.01
1.04
0.98
1.01
1.05
2.08
3.02
1.06
f1 (ppm)

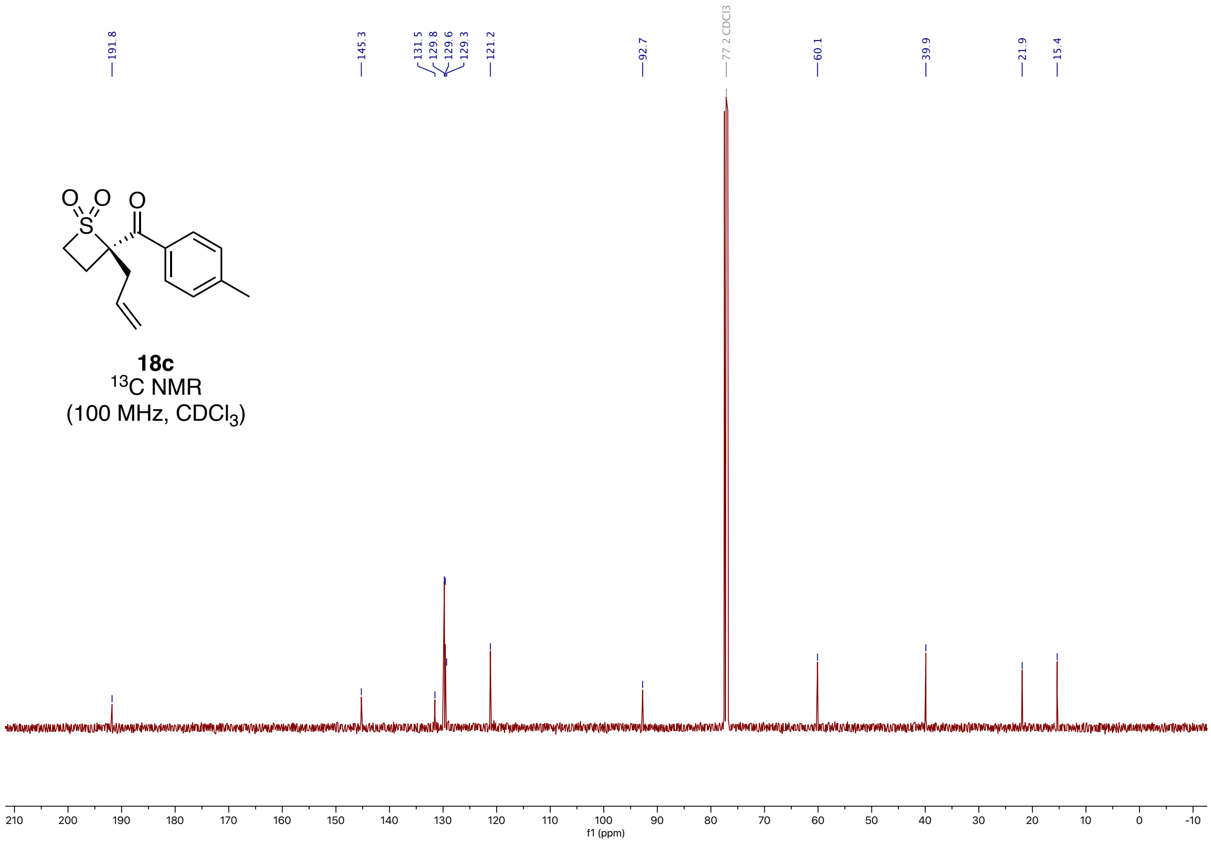
191.8
145.3
131.5
129.8
129.6
129.3
121.2
92.7
77.2 CDCl3
60.1
39.9
21.9
15.4
O O O
S
18c
13C NMR
(100 MHz, CDCl3)
f1 (ppm)

18d
^{1}H NMR
(400 MHz, $CDCl_3$)

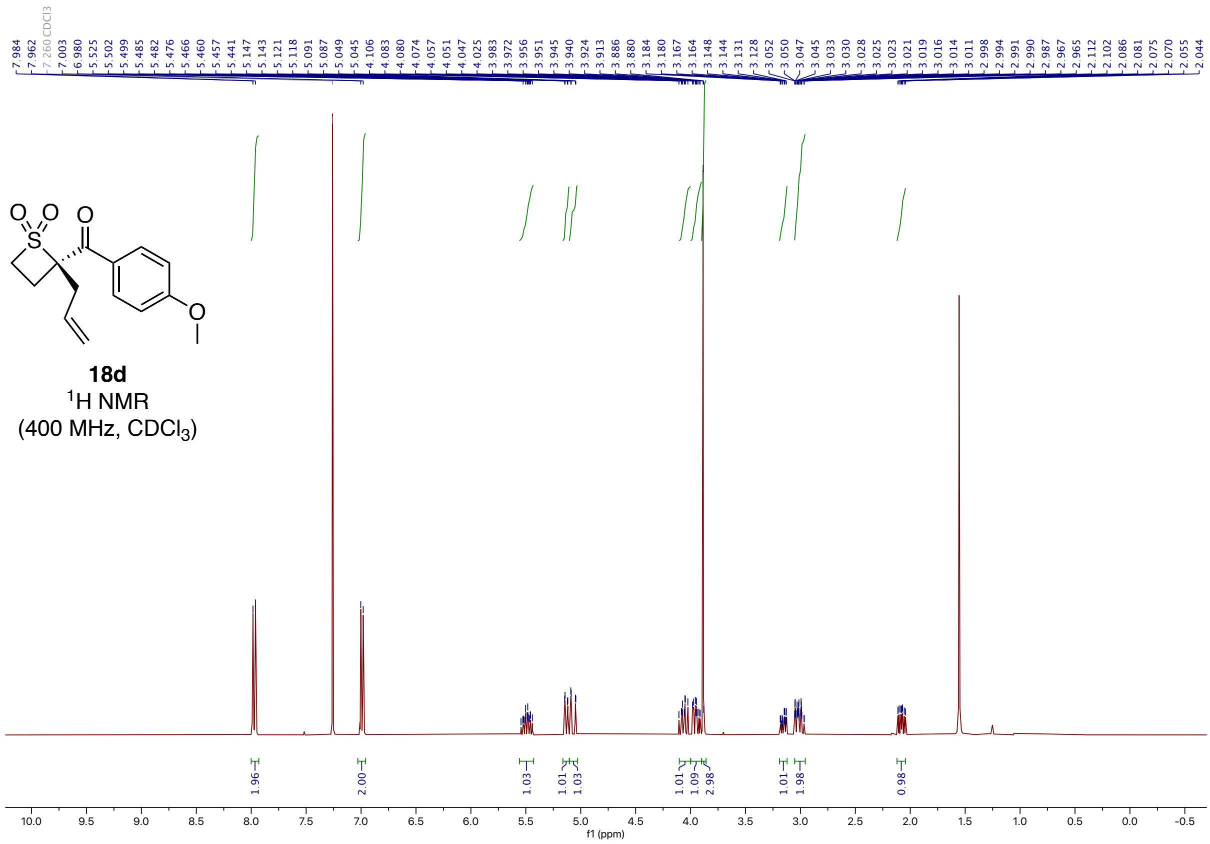

18d
^{13}C NMR
(100 MHz, $CDCl_3$)

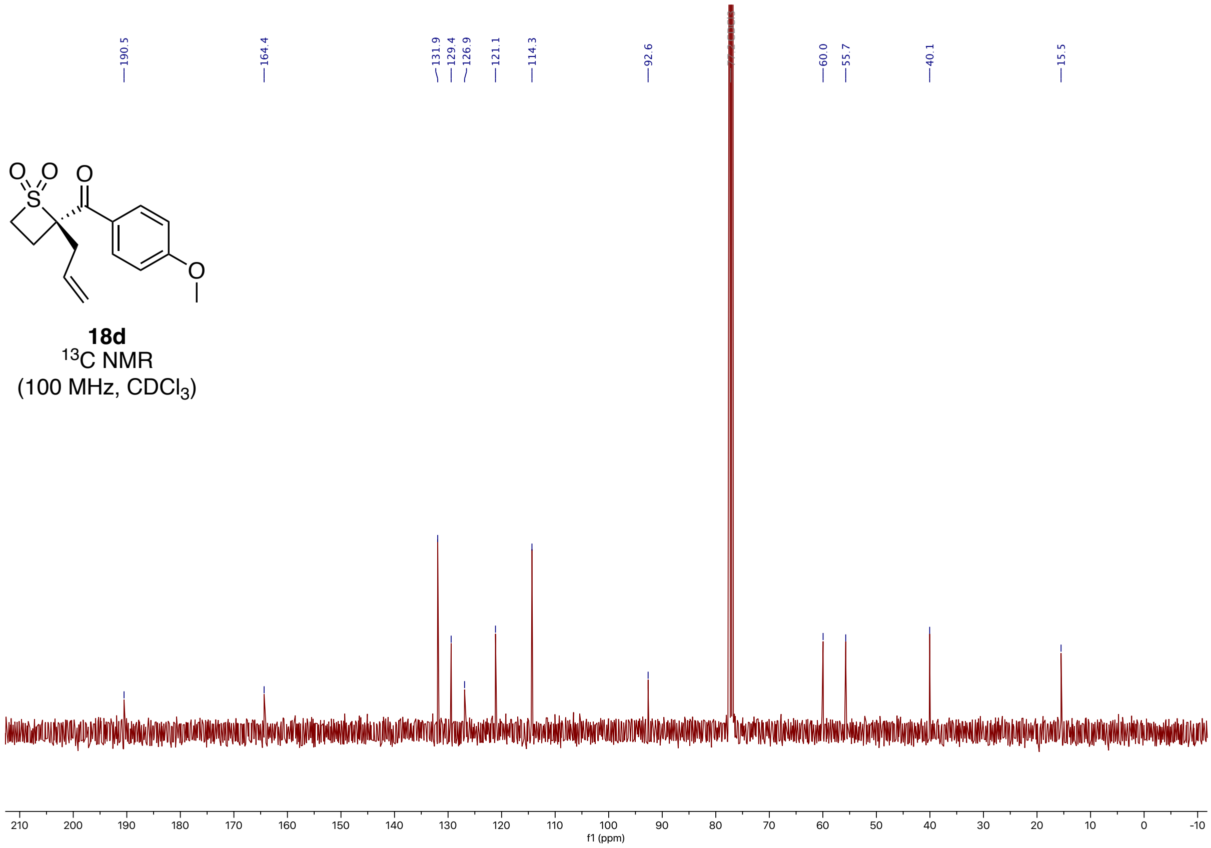

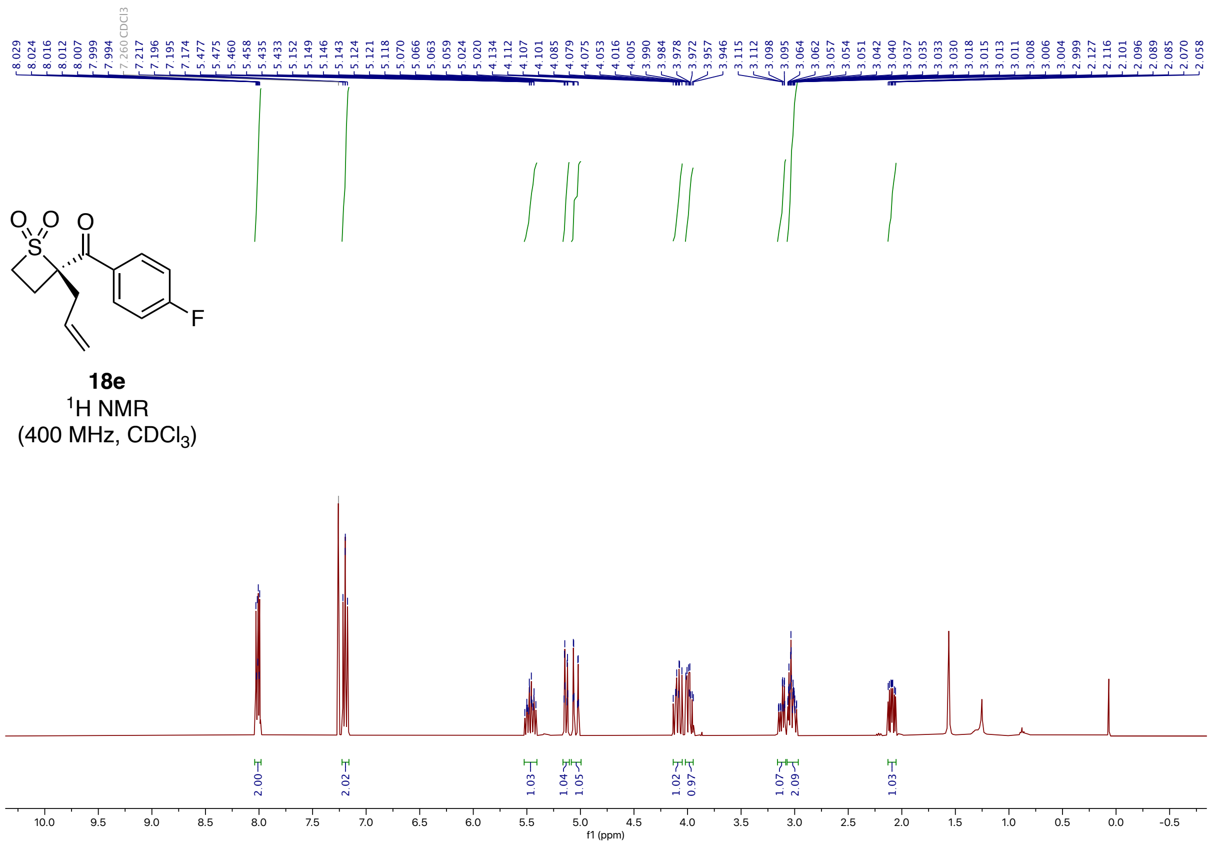

18e
^{1}H NMR
(400 MHz, $CDCl_3$)

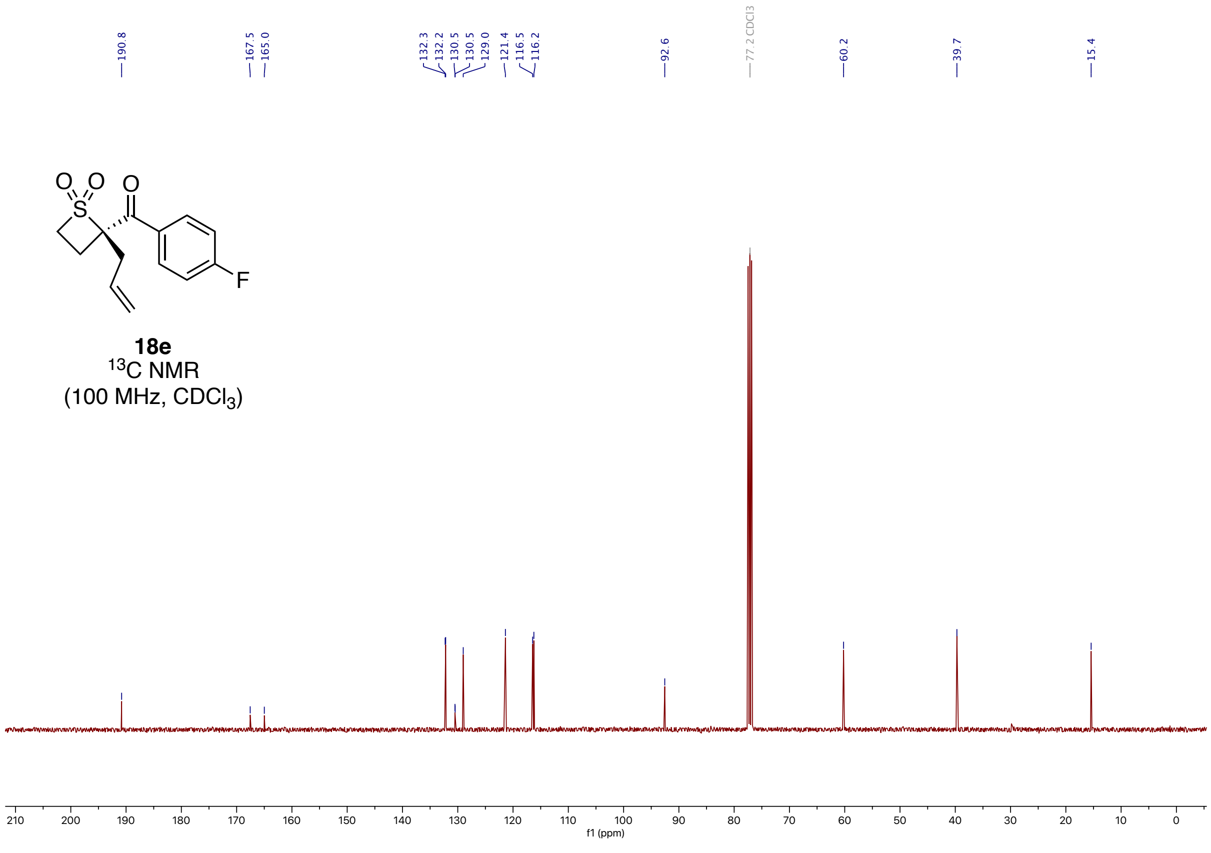

18e
^{13}C NMR
(100 MHz, $CDCl_3$)

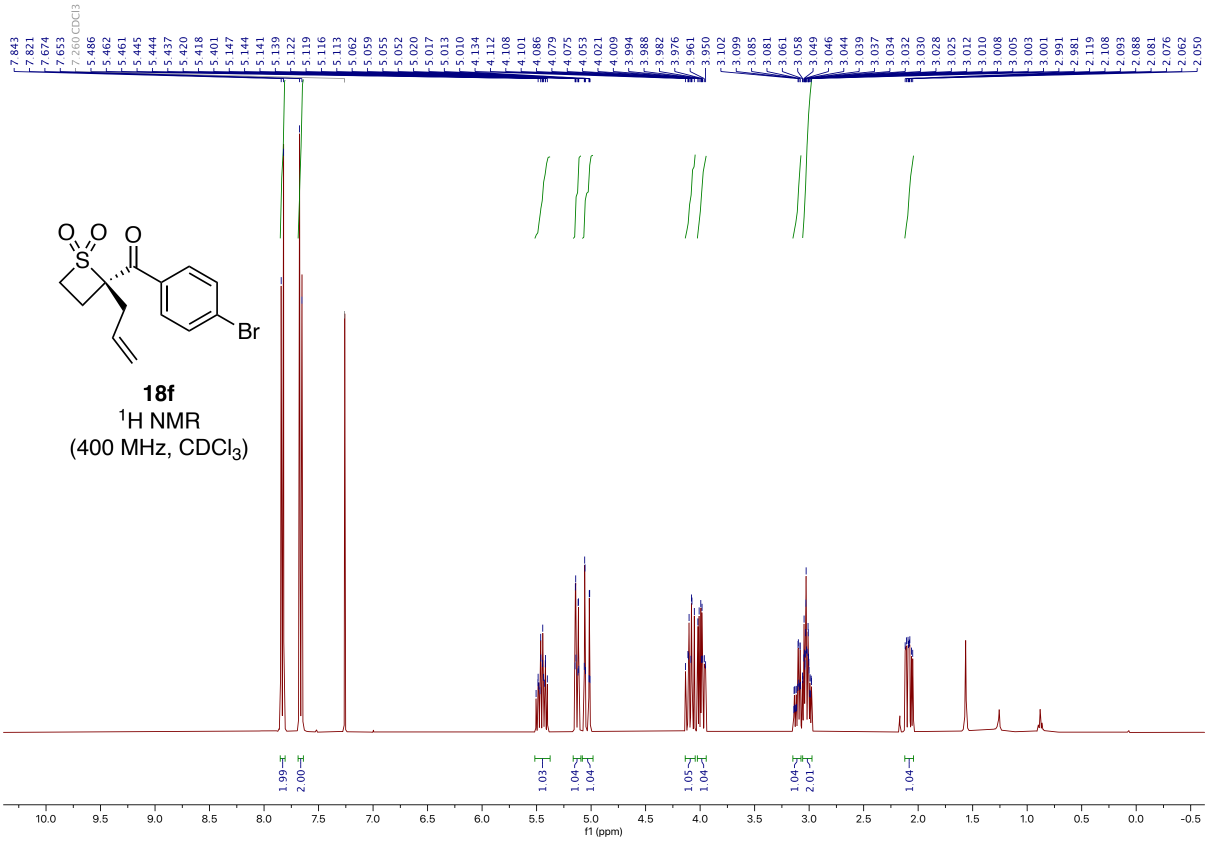
18f
^{1}H NMR
(400 MHz, $CDCl_3$)
f1 (ppm)

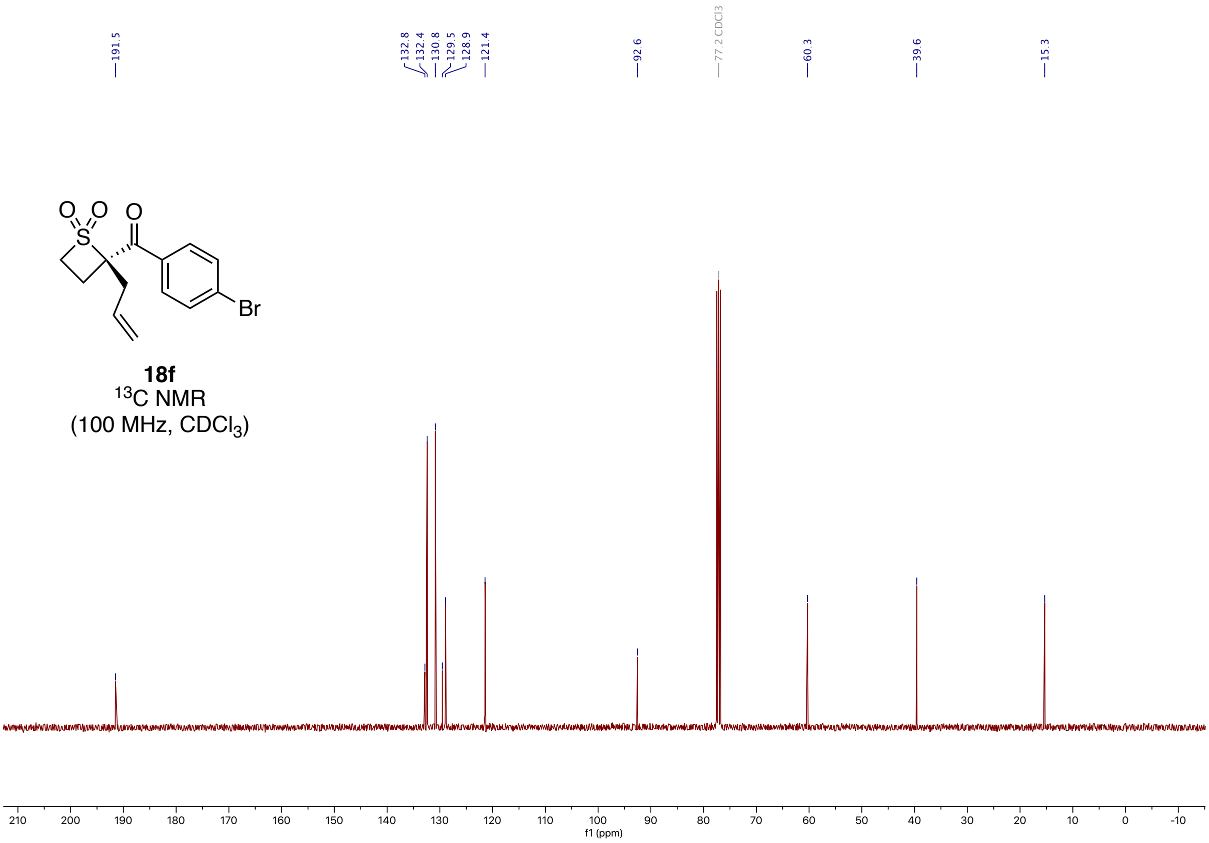
18f
^{13}C NMR
(100 MHz, $CDCl_3$)
191.5
132.8
132.4
130.8
129.5
128.9
121.4
92.6
77.2 CDCl3
60.3
39.6
15.3
f1 (ppm)

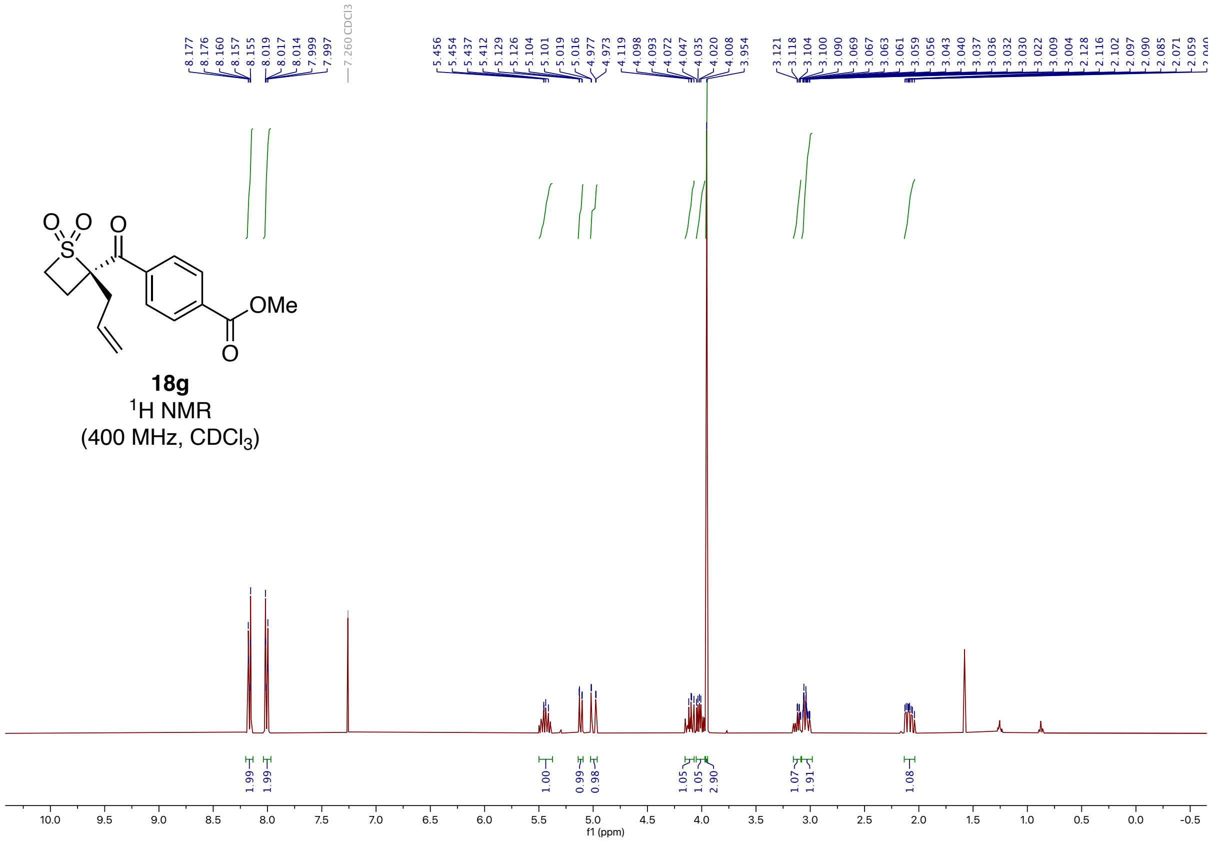
OMe
18g
1H NMR
(400 MHz, CDCl3)
f1 (ppm)

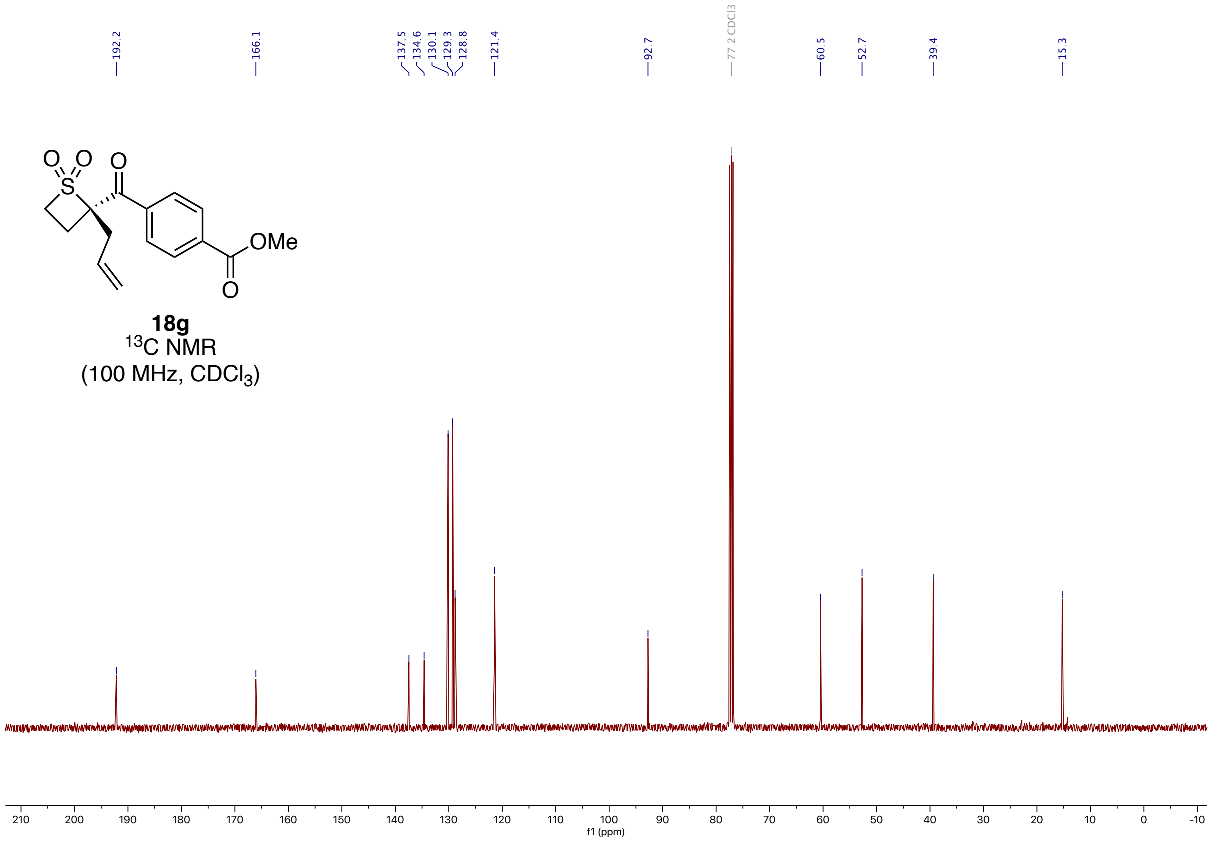
OMe
18g
13C NMR
(100 MHz, CDCl3)
192.2
166.1
137.5
134.6
130.1
129.3
128.8
121.4
92.7
77.2 CDCl3
60.5
52.7
39.4
15.3
f1 (ppm)

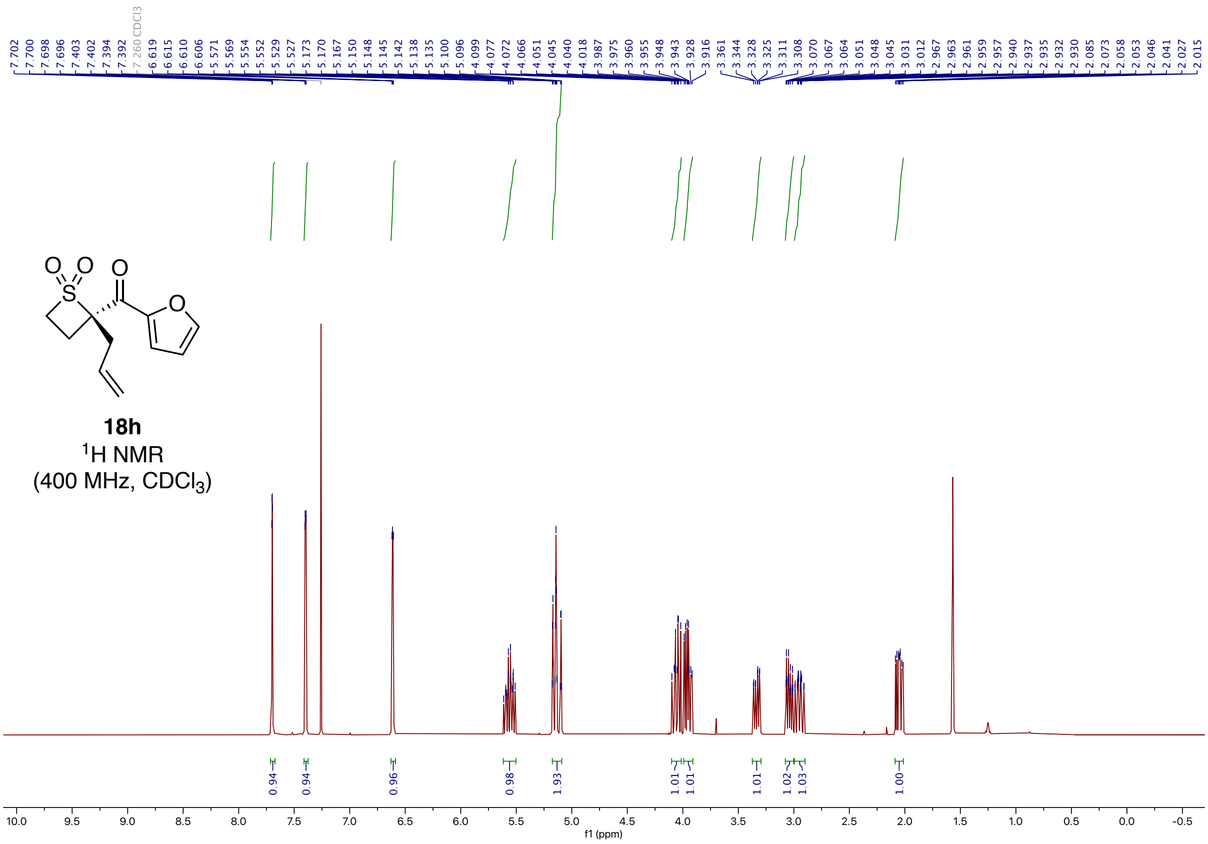

18h
^1H NMR
(400 MHz, $CDCl_3$)

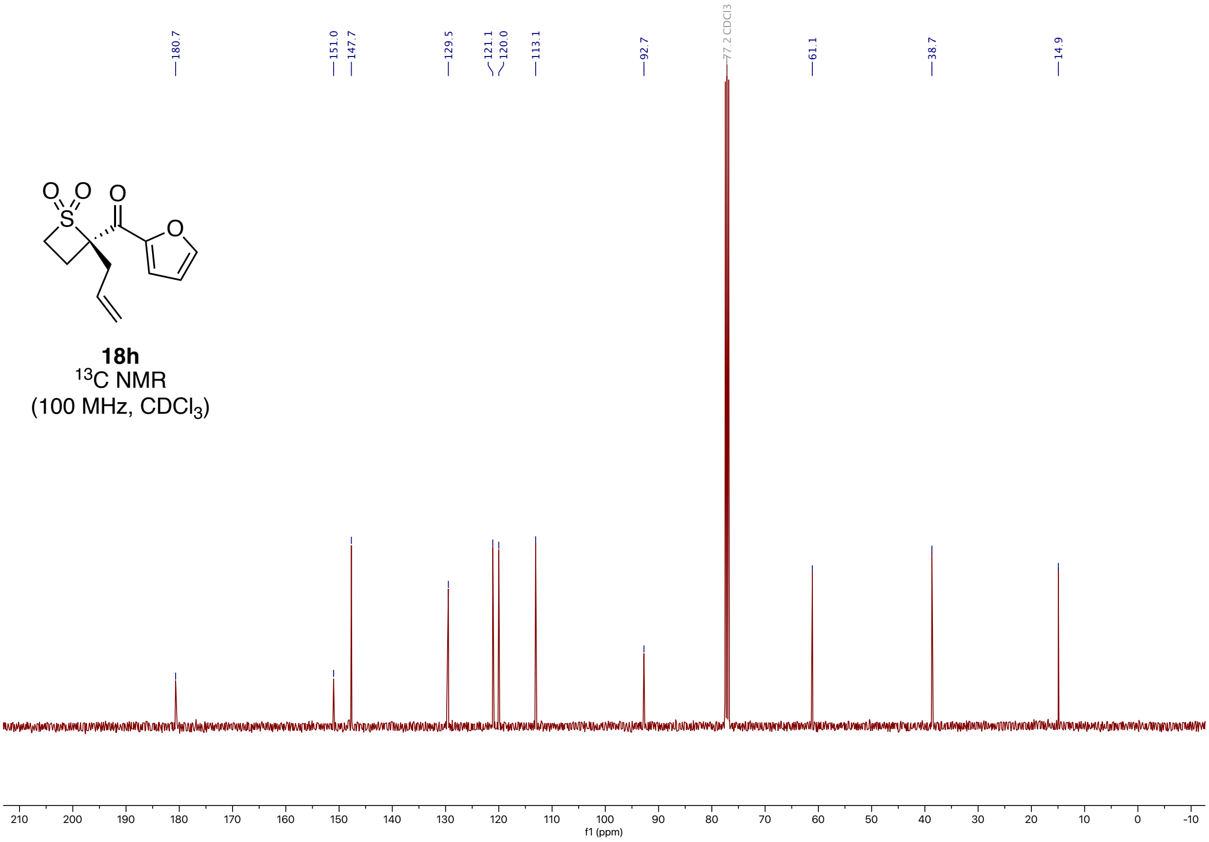

18h
^{13}C NMR
(100 MHz, $CDCl_3$)

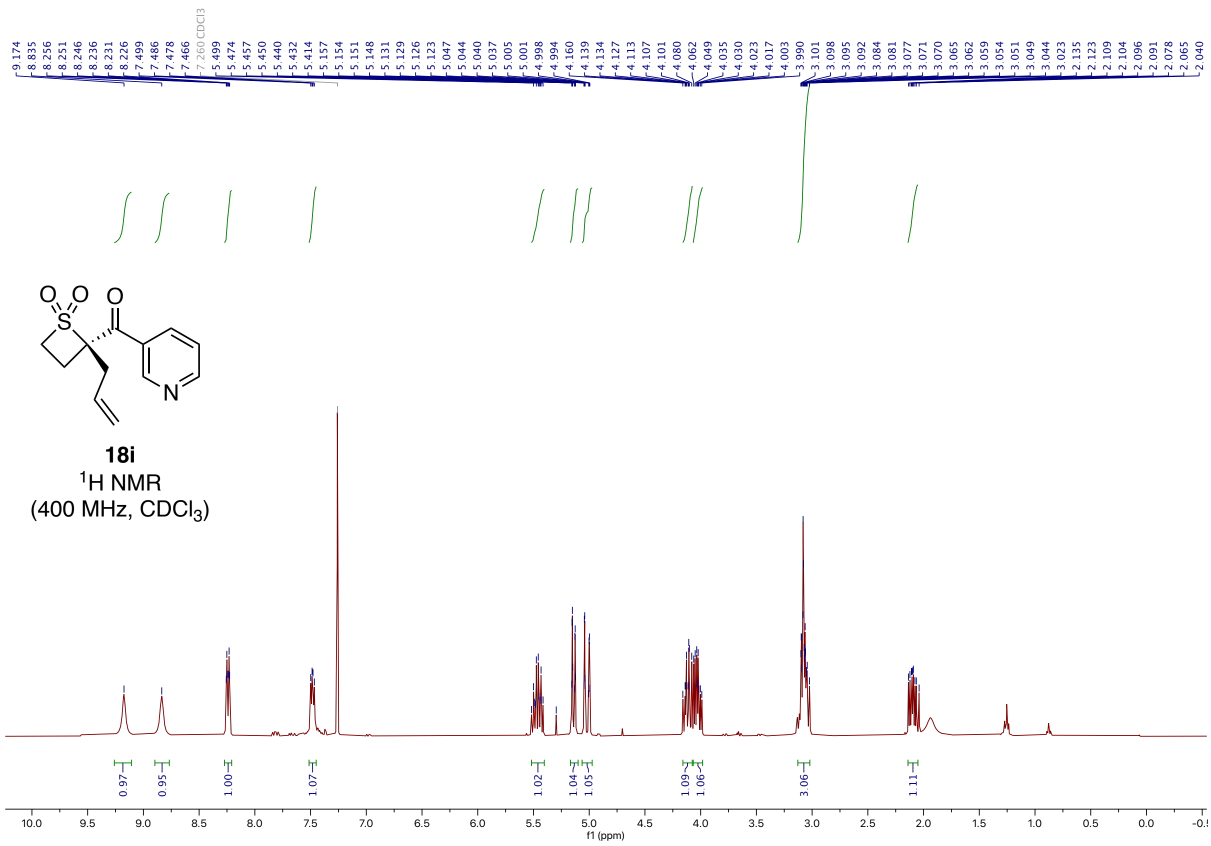

18i
^1H NMR
(400 MHz, $CDCl_3$)

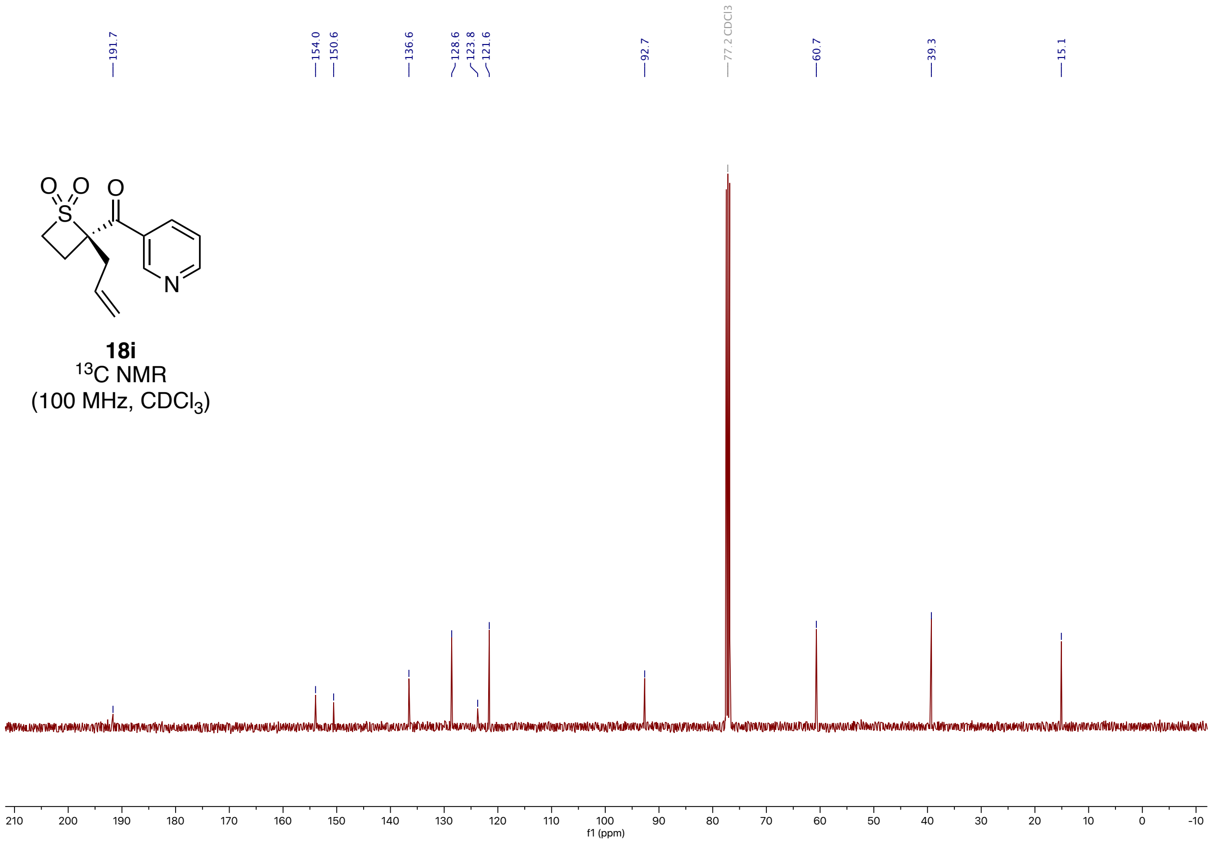

18i
^{13}C NMR
(100 MHz, $CDCl_3$)

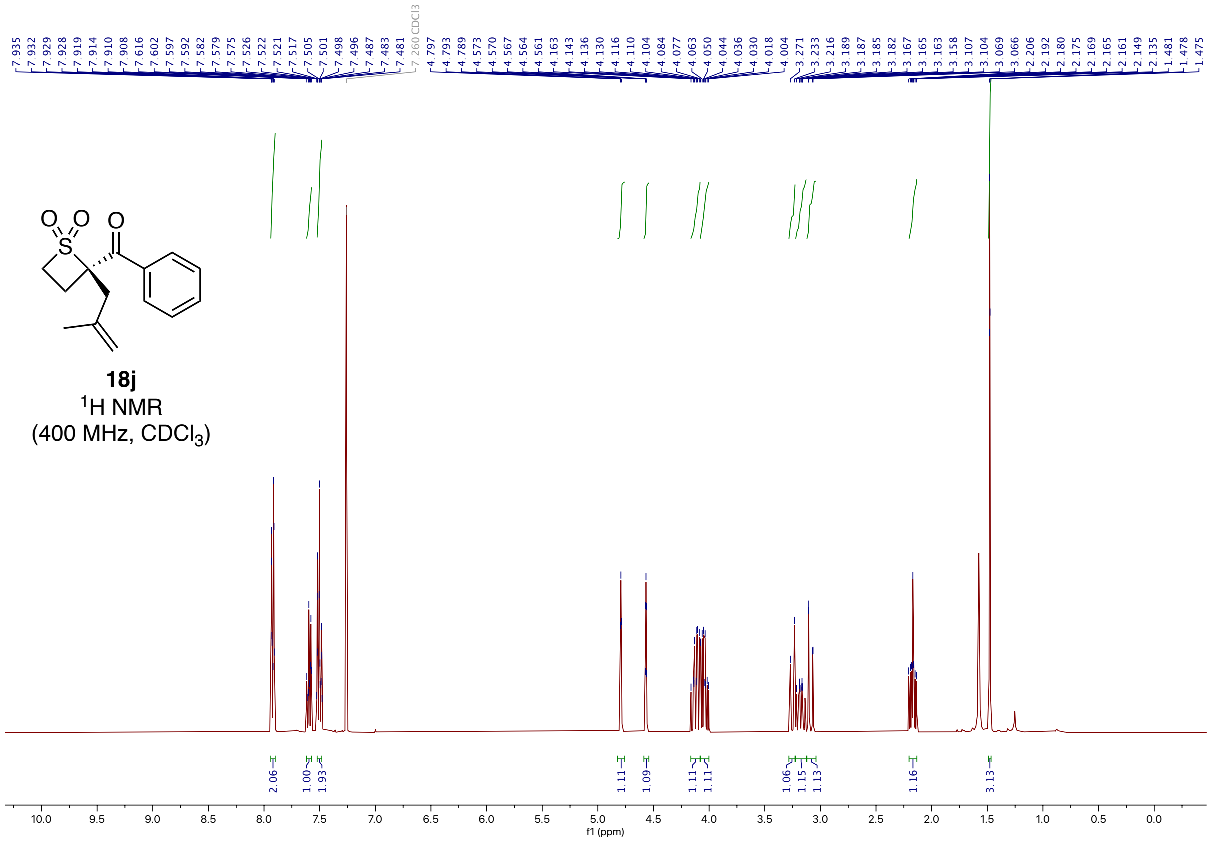
7.935 7.932 7.929 7.928 7.919 7.914 7.910 7.908 7.616 7.602 7.597 7.592 7.582 7.579 7.575 7.526 7.522 7.521 7.517 7.505 7.501 7.498 7.496 7.487 7.483 7.481
7.260 CDCl3
4.797 4.793 4.789 4.573 4.570 4.567 4.564 4.561 4.163 4.143 4.136 4.130 4.116 4.110 4.104 4.084 4.077 4.063 4.050 4.044 4.036 4.030 4.018 4.004 3.271 3.233 3.216 3.189 3.187 3.185 3.182 3.167 3.165 3.163 3.158 3.107 3.104 3.069 3.066 2.206 2.192 2.180 2.175 2.169 2.165 2.161 2.149 2.135 1.481 1.478 1.475
18j
^{1}H NMR
(400 MHz, $CDCl_3$)
2.06 1.00 1.93 1.11 1.09 1.11 1.11 1.06 1.15 1.13 1.16 3.13
10.0 9.5 9.0 8.5 8.0 7.5 7.0 6.5 6.0 5.5 5.0 4.5 4.0 3.5 3.0 2.5 2.0 1.5 1.0 0.5 0.0
f1 (ppm)

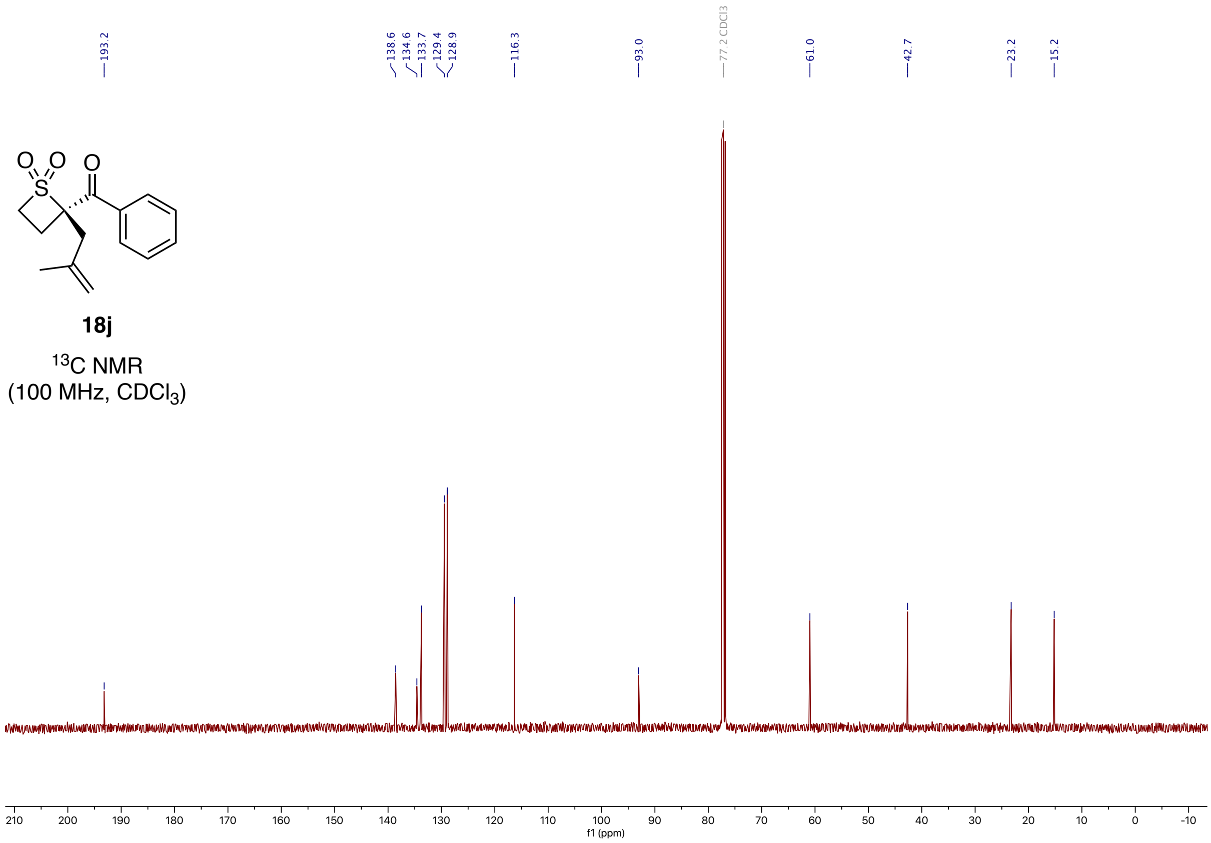
193.2
138.6 134.6 133.7 129.4 128.9
116.3
93.0
77.2 CDCl3
61.0
42.7
23.2
15.2
18j
^{13}C NMR
(100 MHz, $CDCl_3$)
210 200 190 180 170 160 150 140 130 120 110 100 90 80 70 60 50 40 30 20 10 0 -10
f1 (ppm)

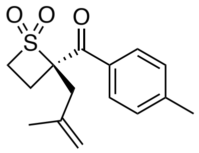

18k
^{1}H NMR
(400 MHz, $CDCl_3$)

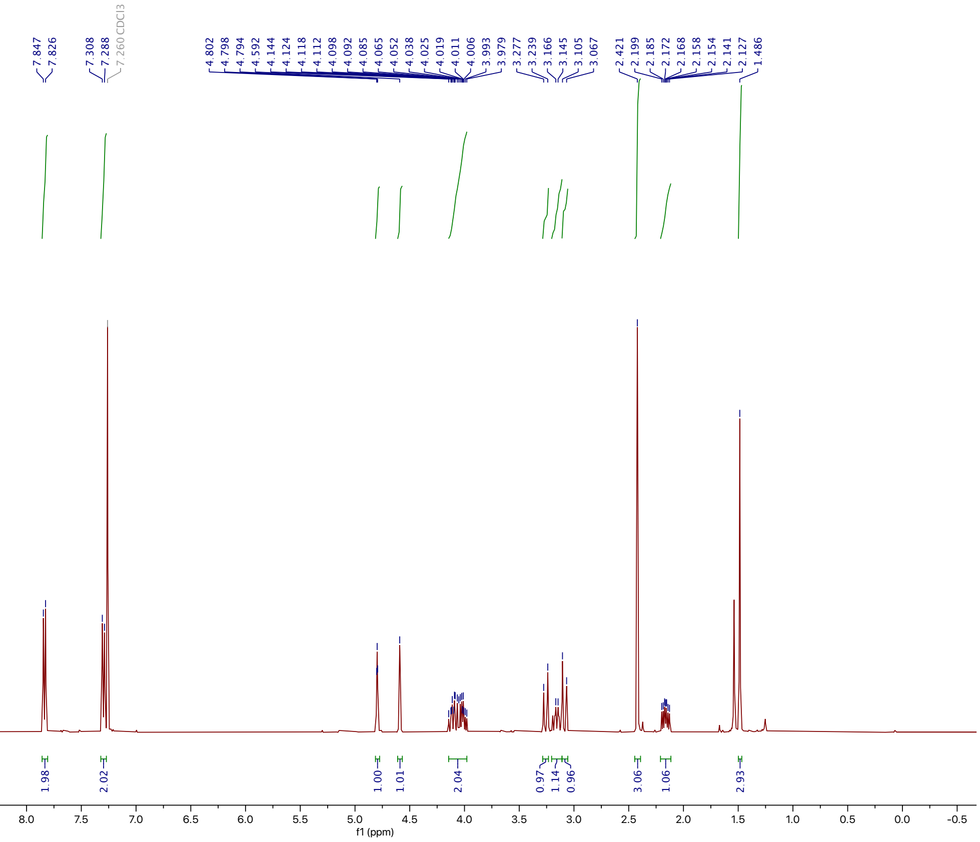

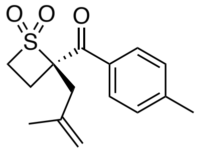

18k
^{13}C NMR
(100 MHz, $CDCl_3$)

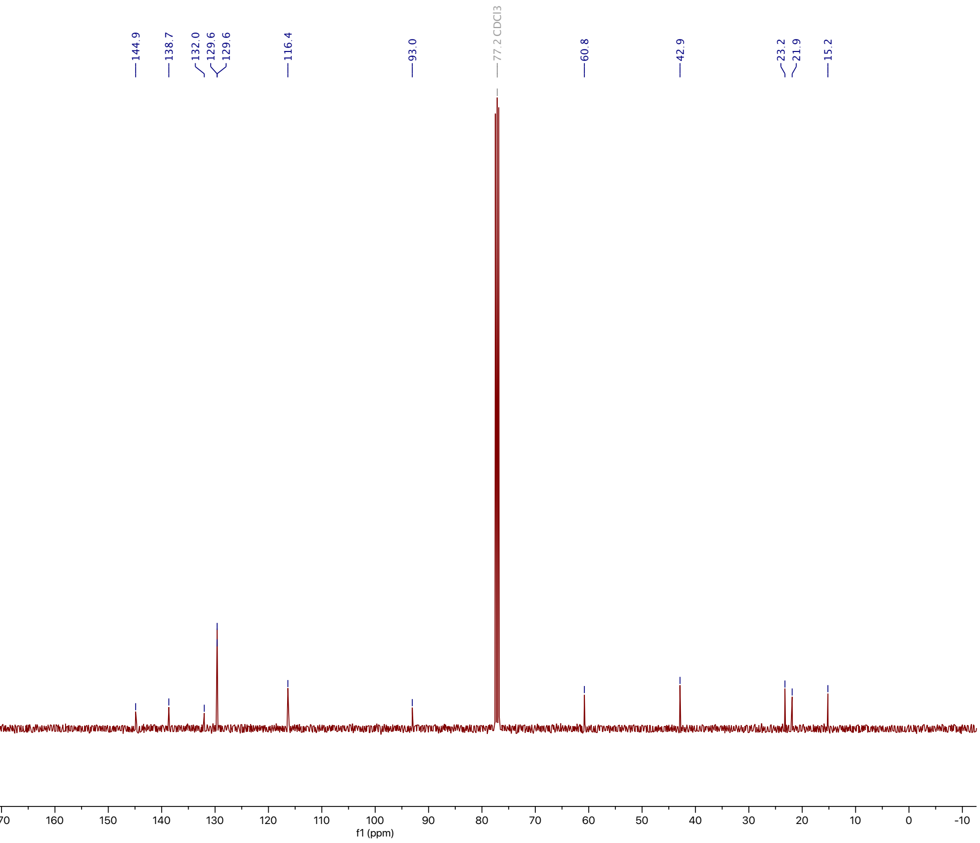

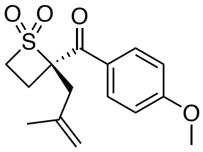

18l

^{1}H NMR

(400 MHz, $CDCl_3$)

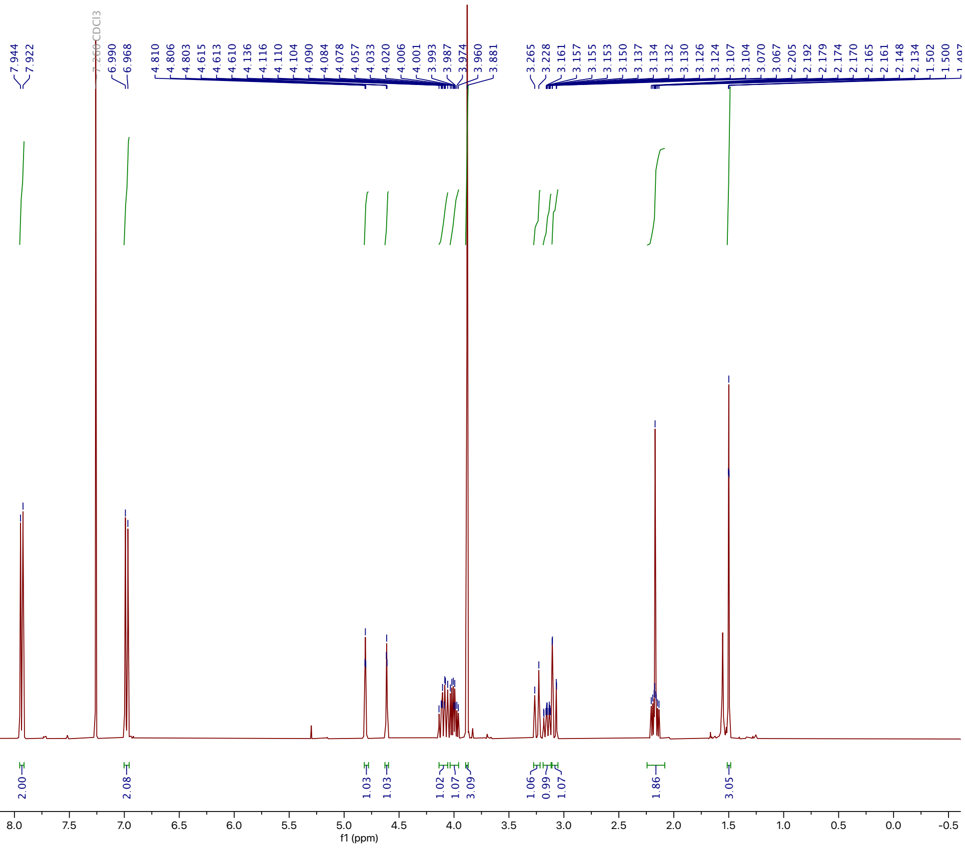

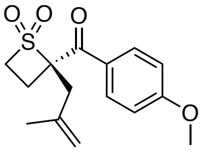

18l

^{13}C NMR

(100 MHz, $CDCl_3$)

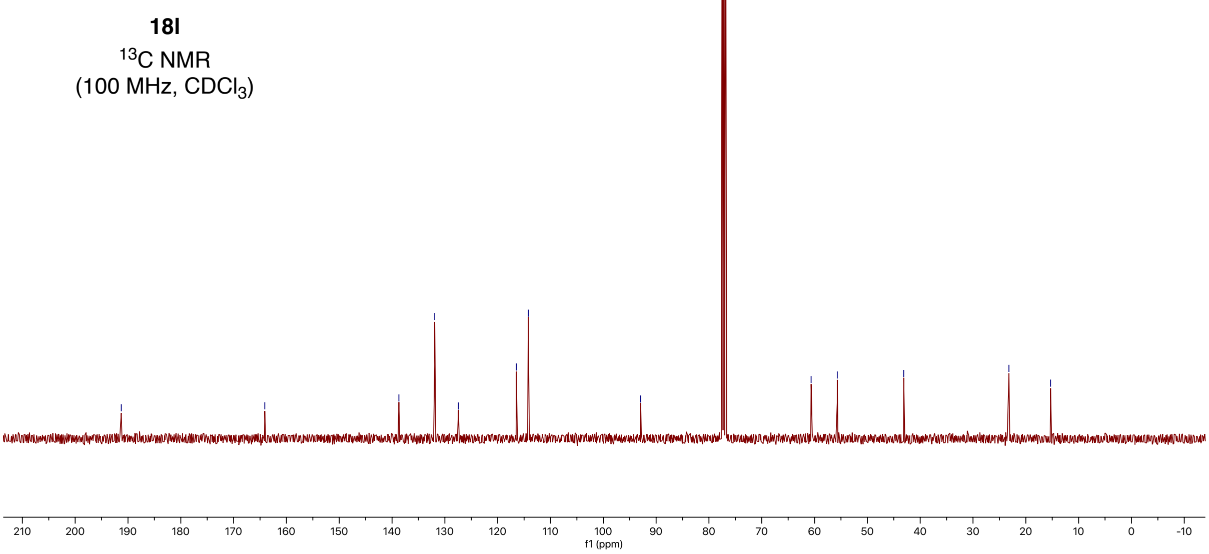

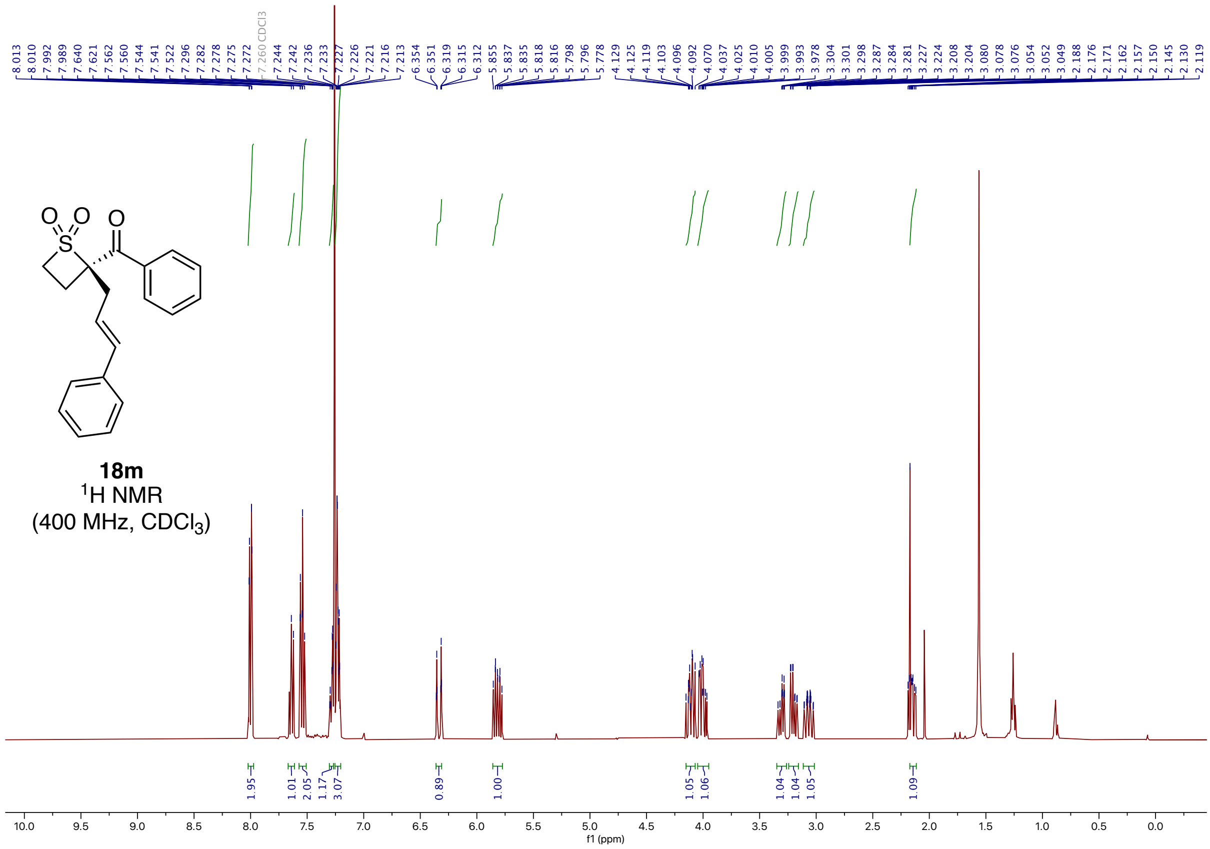

18m
^{1}H NMR
(400 MHz, $CDCl_3$)

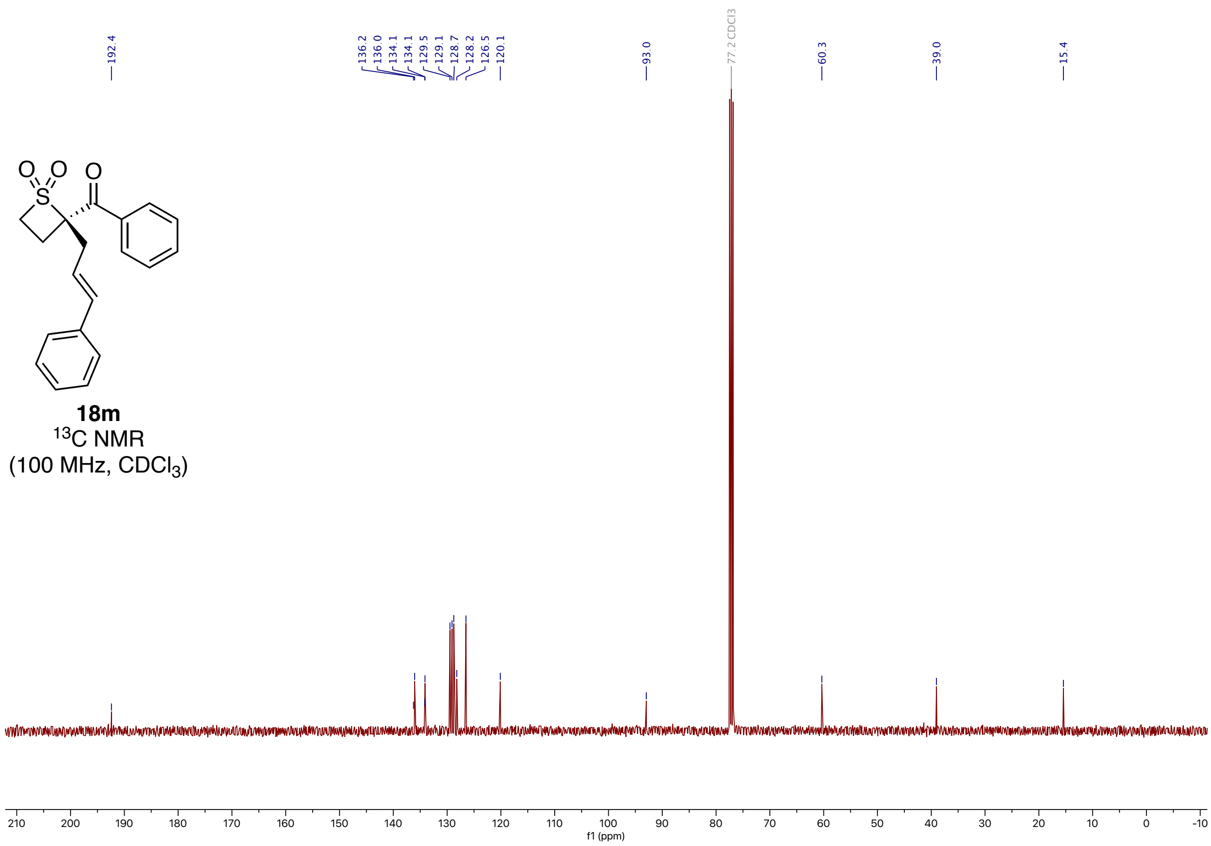

18m
^{13}C NMR
(100 MHz, $CDCl_3$)

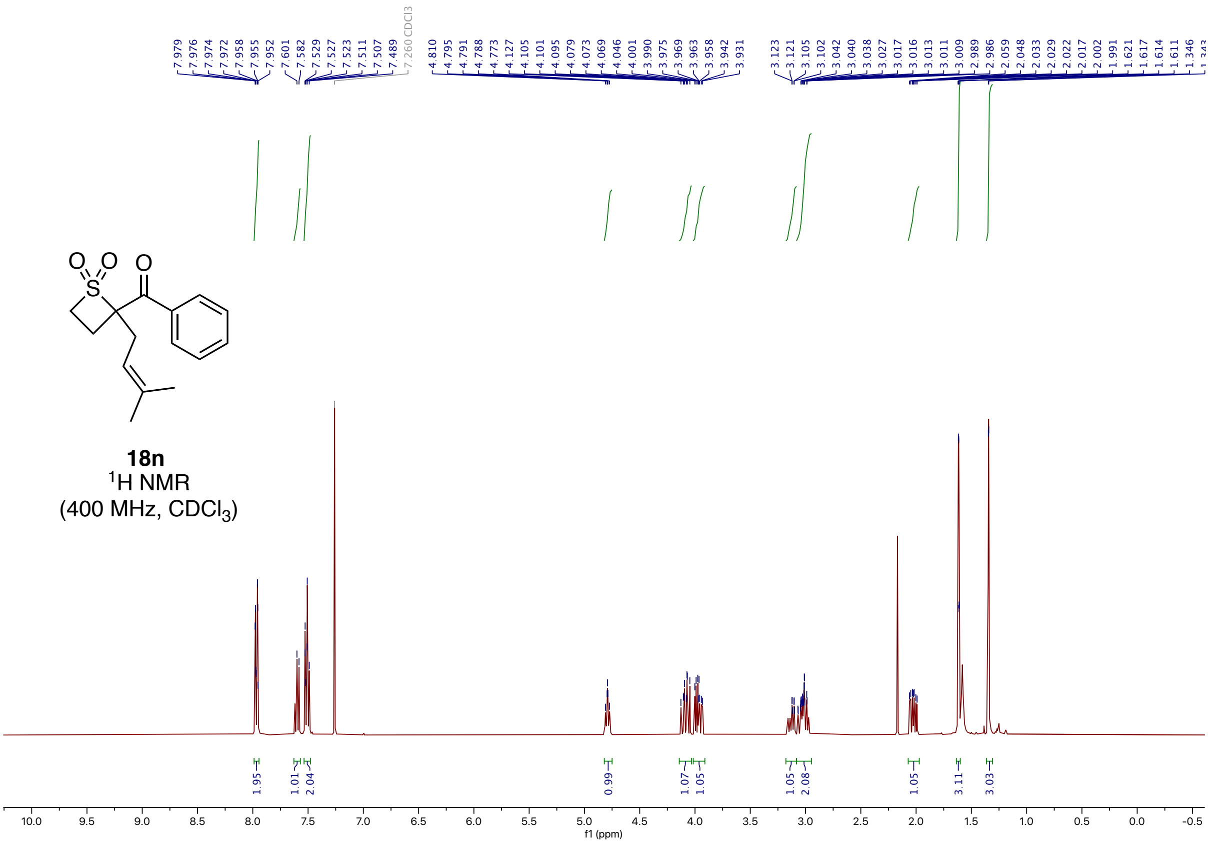

18n
^1H NMR
(400 MHz, $CDCl_3$)

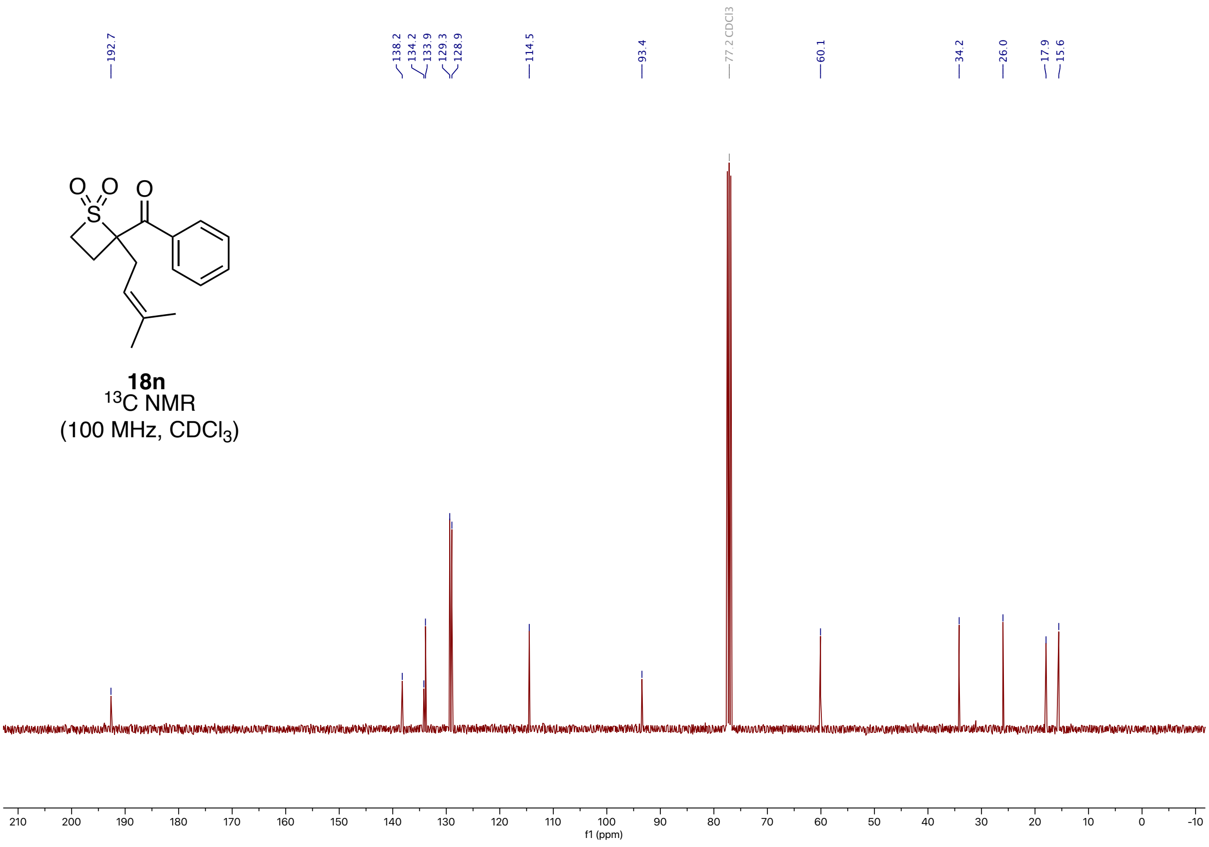

18n
^{13}C NMR
(100 MHz, $CDCl_3$)

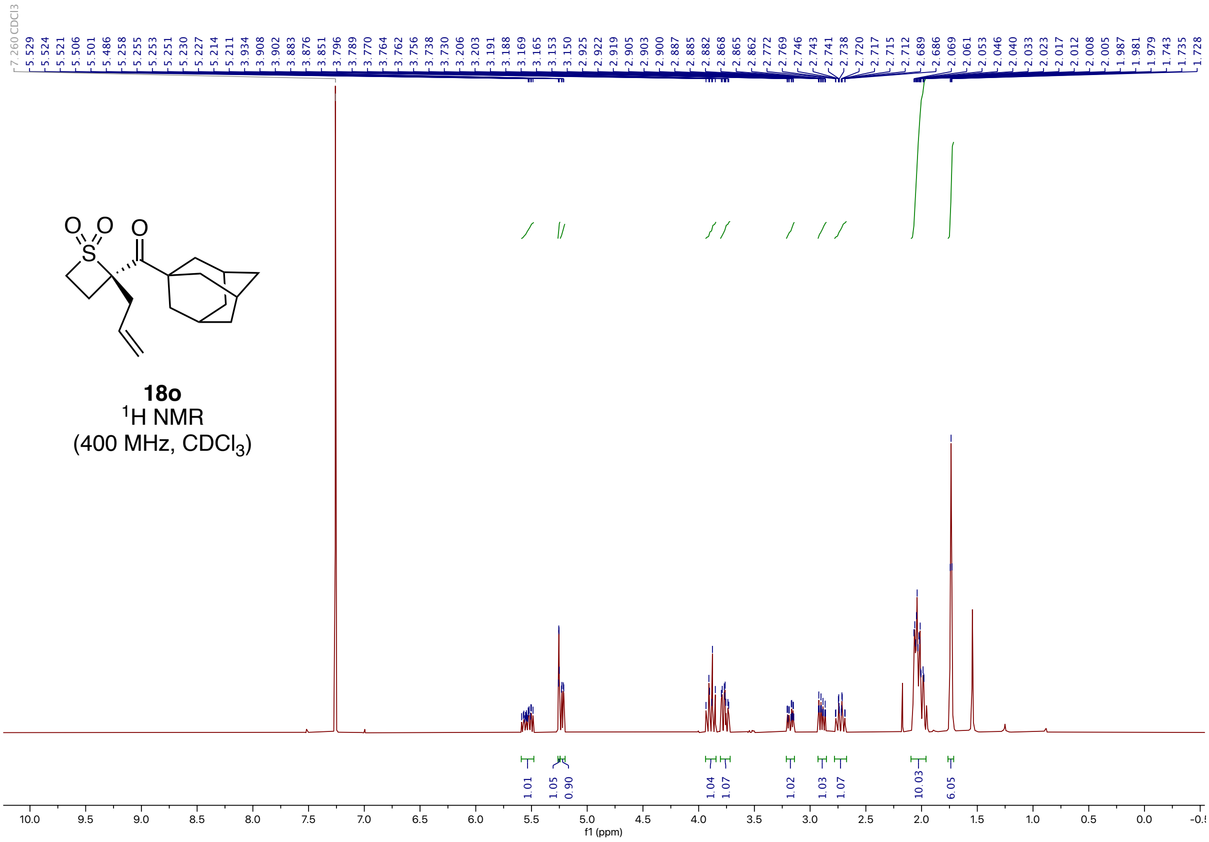
7.260 CDCl3
5.529
5.524
5.521
5.506
5.501
5.486
5.258
5.255
5.253
5.251
5.230
5.227
5.214
5.211
3.934
3.908
3.902
3.883
3.876
3.851
3.796
3.789
3.770
3.764
3.762
3.756
3.738
3.730
3.206
3.203
3.191
3.188
3.169
3.165
3.153
3.150
2.925
2.922
2.919
2.905
2.903
2.900
2.887
2.885
2.882
2.868
2.865
2.862
2.772
2.769
2.746
2.743
2.741
2.738
2.720
2.717
2.715
2.712
2.689
2.686
2.069
2.061
2.053
2.046
2.040
2.033
2.023
2.017
2.012
2.008
2.005
1.987
1.981
1.979
1.743
1.735
1.728
18o
1H NMR
(400 MHz, CDCl3)
1.01
1.05
0.90
1.04
1.07
1.02
1.03
1.07
10.03
6.05
f1 (ppm)

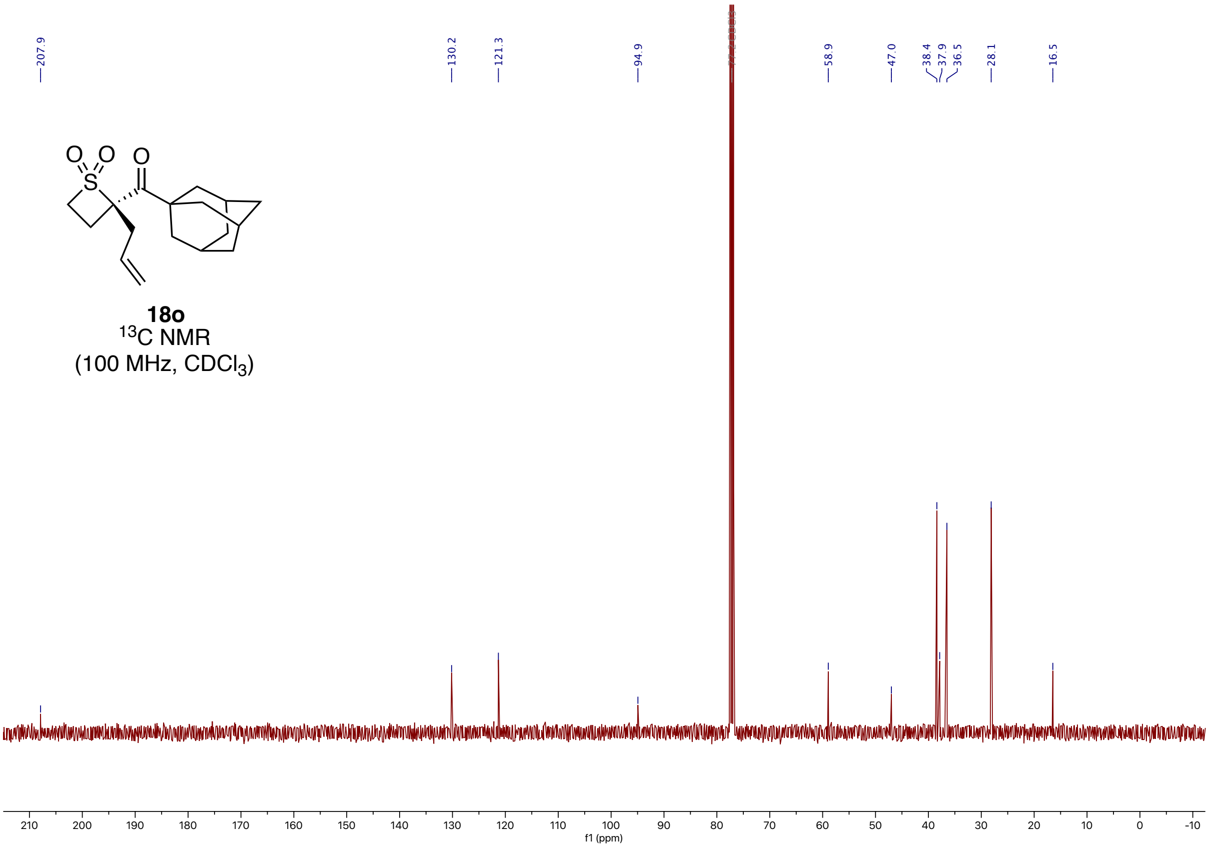
207.9
130.2
121.3
94.9
58.9
47.0
38.4
37.9
36.5
28.1
16.5
18o
13C NMR
(100 MHz, CDCl3)
f1 (ppm)

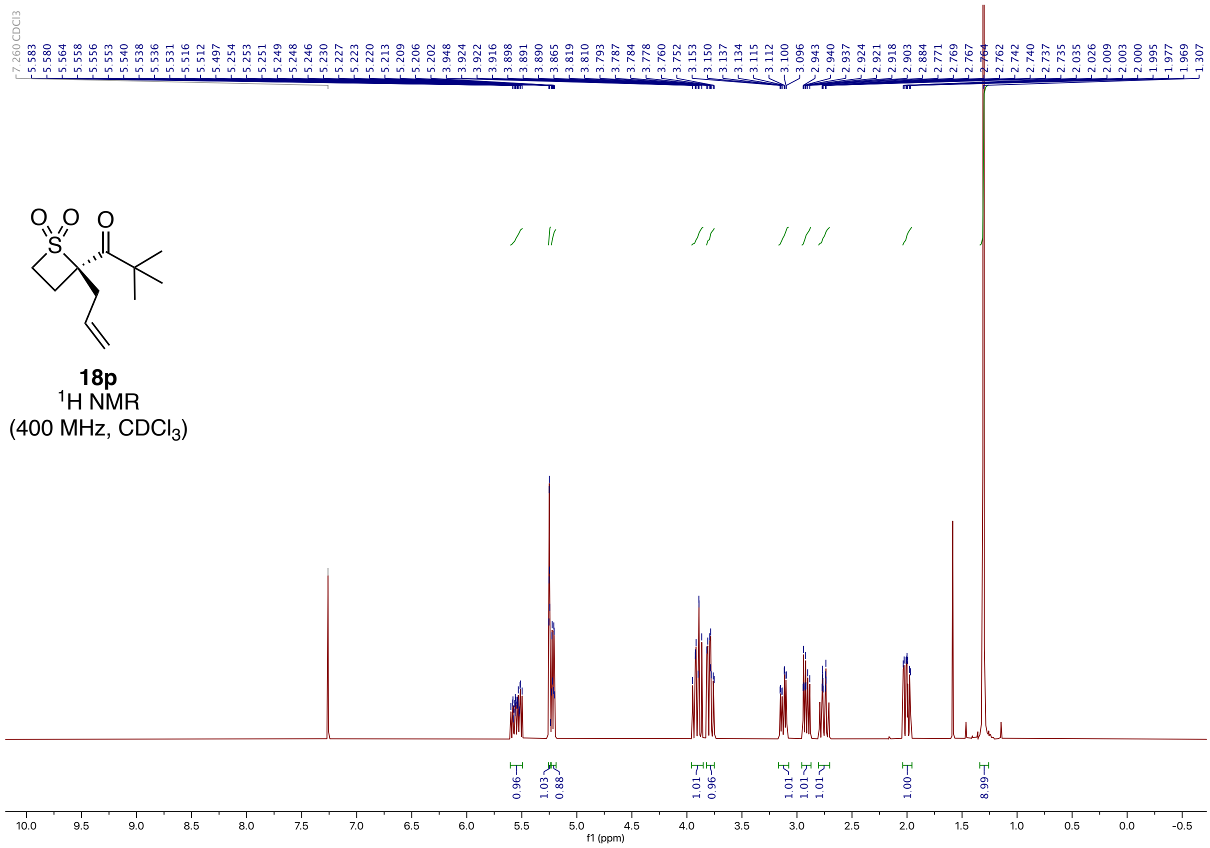
7.260 CDCl3
5.583
5.580
5.564
5.558
5.556
5.553
5.540
5.538
5.536
5.531
5.516
5.512
5.497
5.254
5.253
5.251
5.249
5.248
5.246
5.230
5.227
5.223
5.220
5.213
5.209
5.206
5.202
3.948
3.924
3.922
3.916
3.898
3.891
3.890
3.865
3.819
3.810
3.793
3.787
3.784
3.778
3.760
3.752
3.153
3.150
3.137
3.134
3.115
3.112
3.100
3.096
2.943
2.940
2.937
2.924
2.921
2.918
2.903
2.884
2.771
2.769
2.767
2.764
2.762
2.742
2.740
2.737
2.735
2.035
2.026
2.009
2.003
2.000
1.995
1.977
1.969
1.307
18p
^{1}H NMR
(400 MHz, $CDCl_3$)
0.96
1.03
0.88
1.01
0.96
1.01
1.01
1.01
1.00
8.99
f1 (ppm)

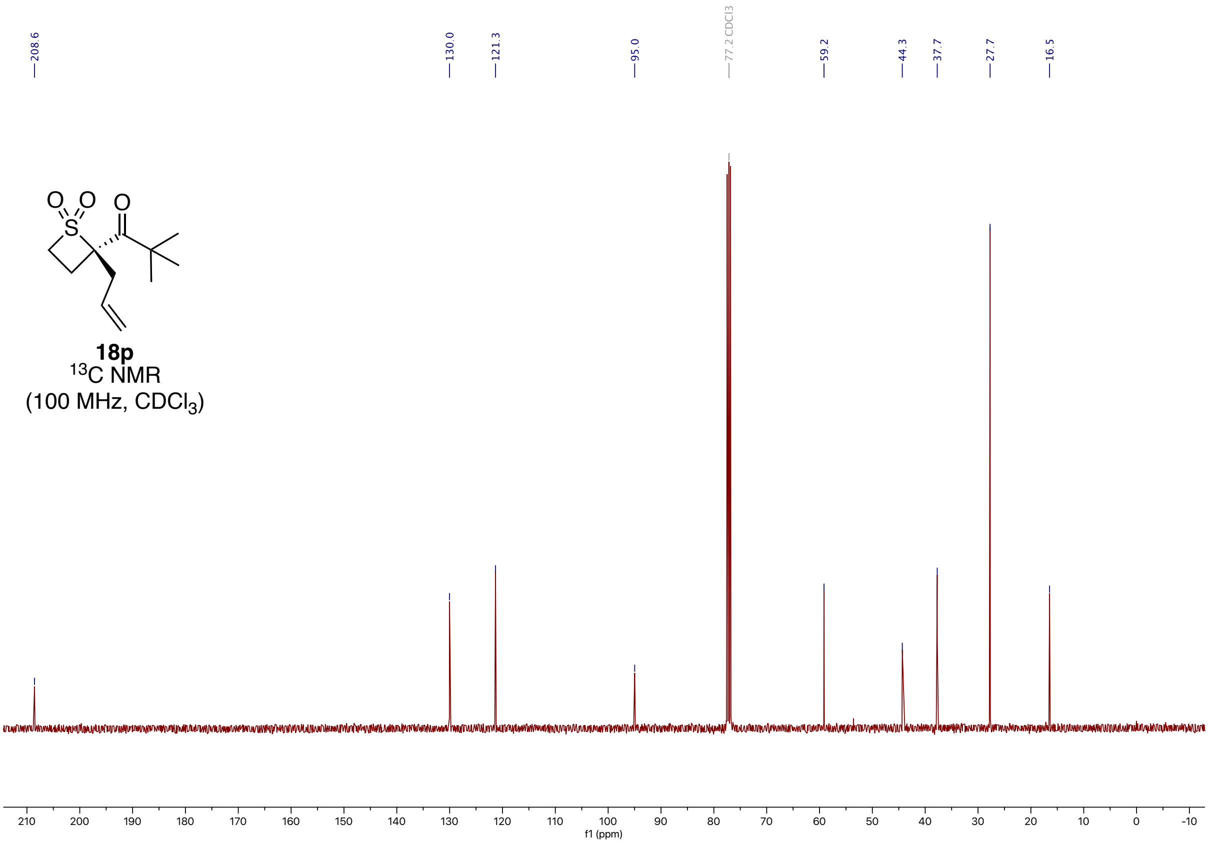
208.6
130.0
121.3
95.0
77.2 CDCl3
59.2
44.3
37.7
27.7
16.5
18p
^{13}C NMR
(100 MHz, $CDCl_3$)
f1 (ppm)

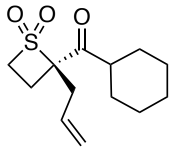

18q
^{1}H NMR
(400 MHz, $CDCl_3$)

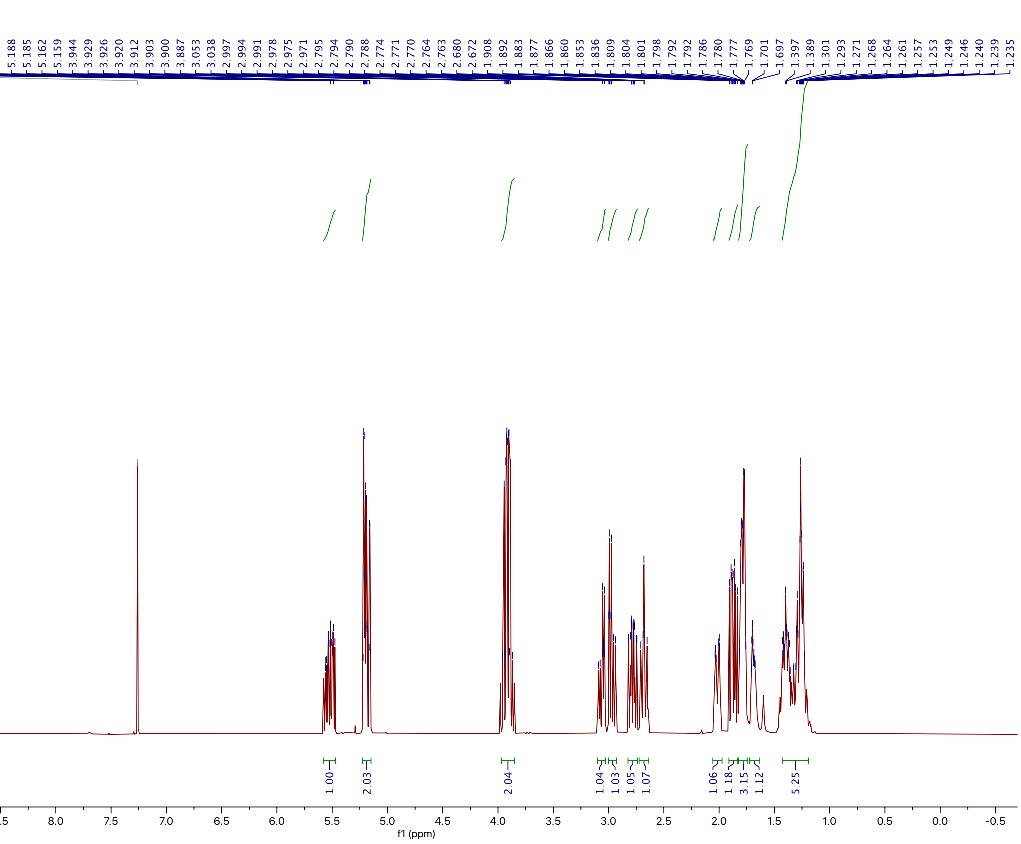

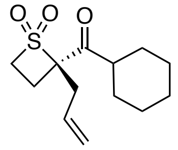

18q
^{13}C NMR
(100 MHz, $CDCl_3$)

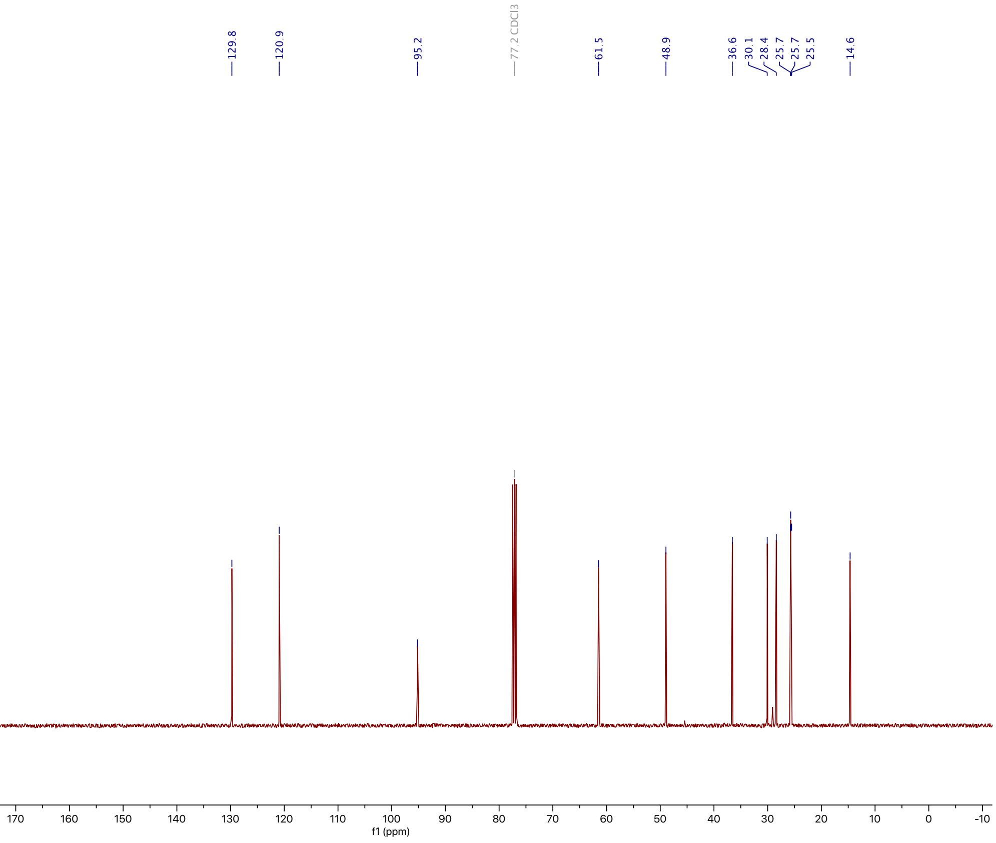

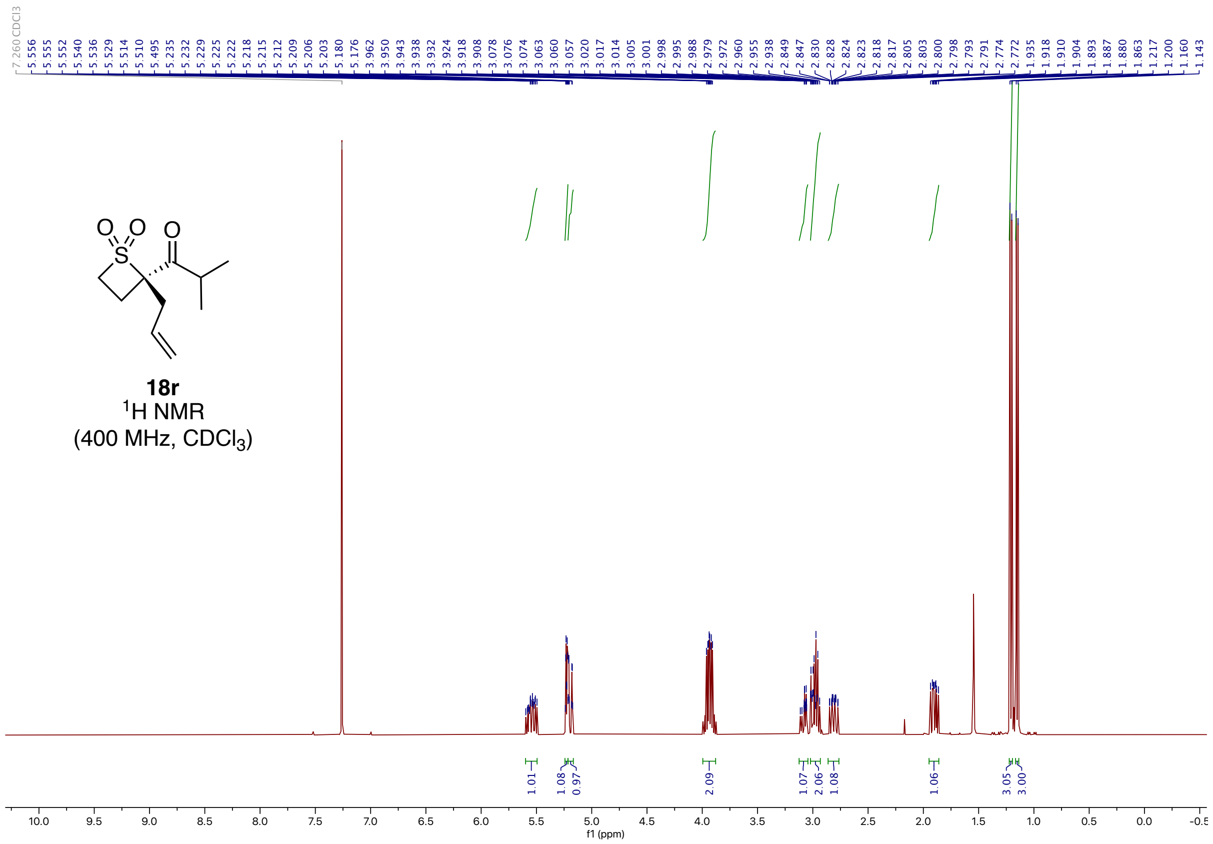

18r
^{1}H NMR
(400 MHz, $CDCl_3$)

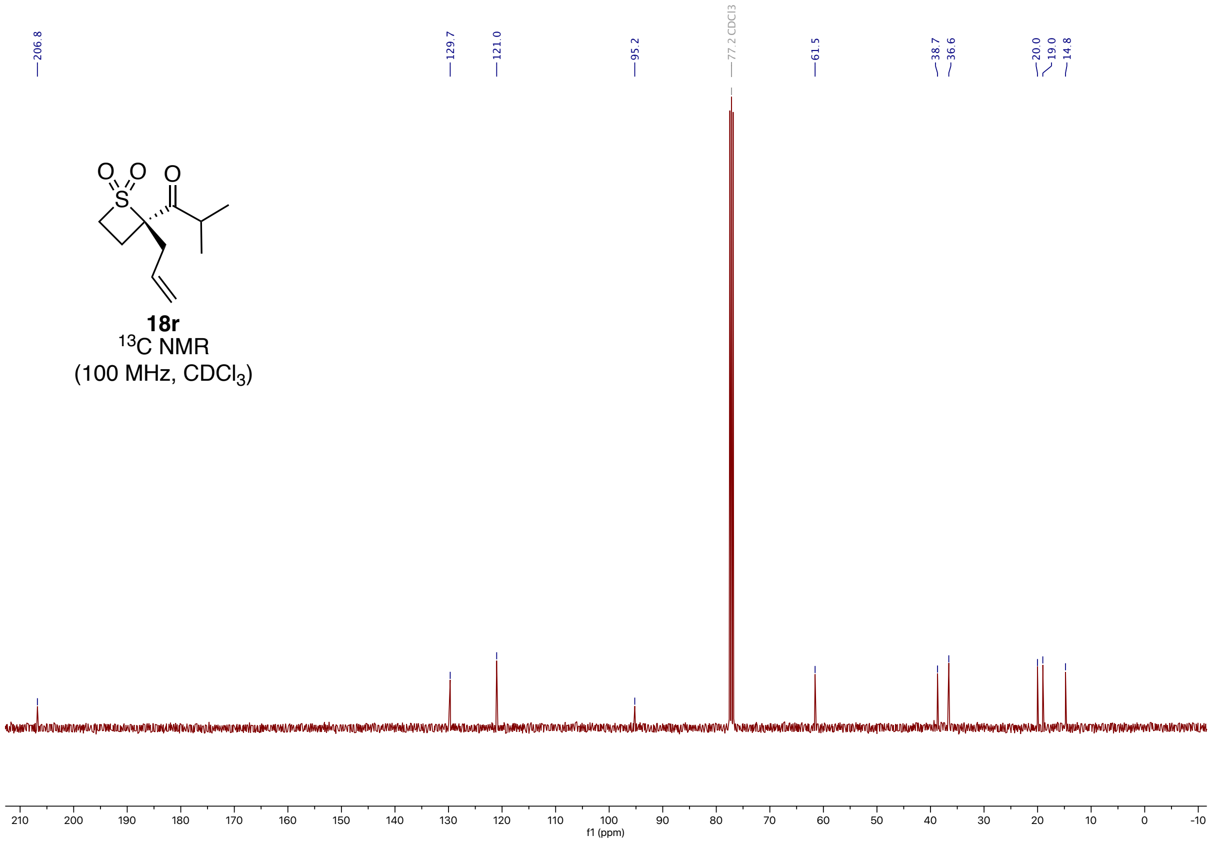

18r
^{13}C NMR
(100 MHz, $CDCl_3$)

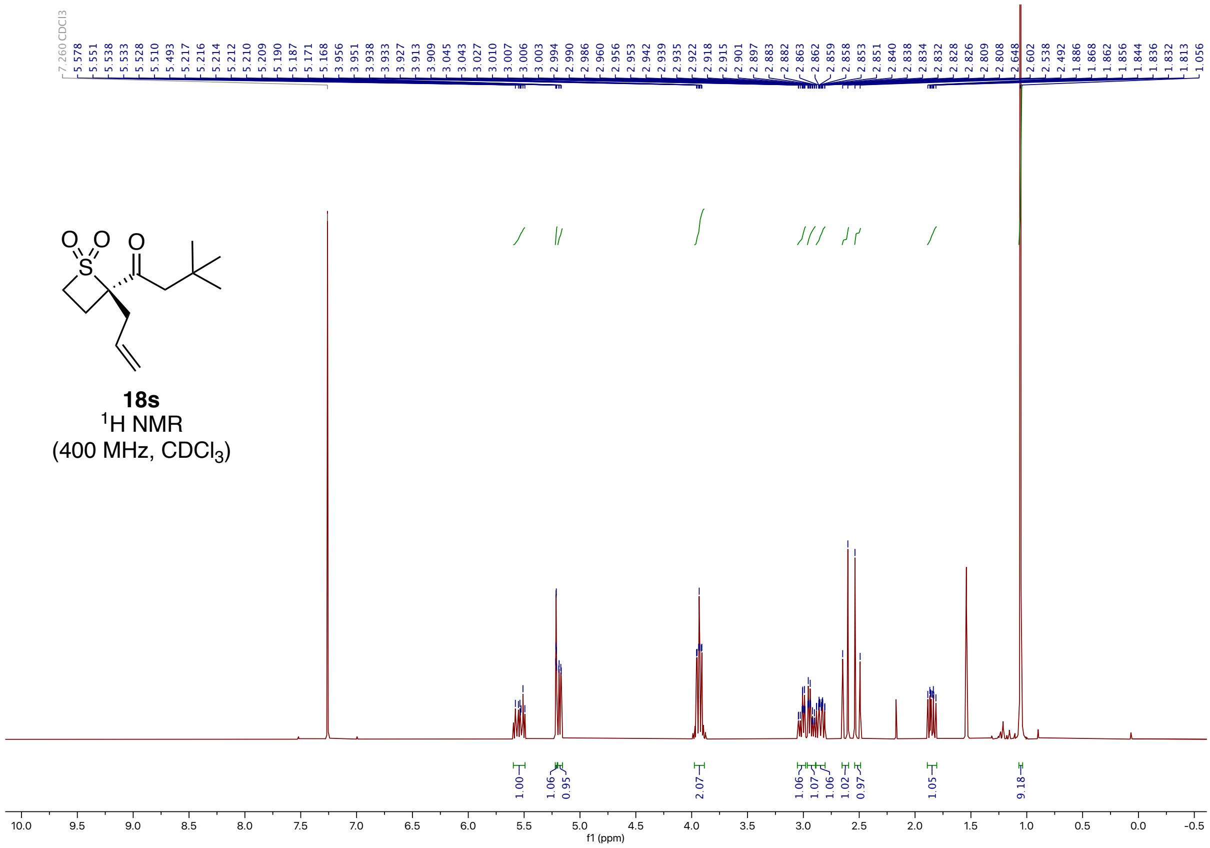
18s
^{1}H NMR
(400 MHz, $CDCl_3$)
f1 (ppm)

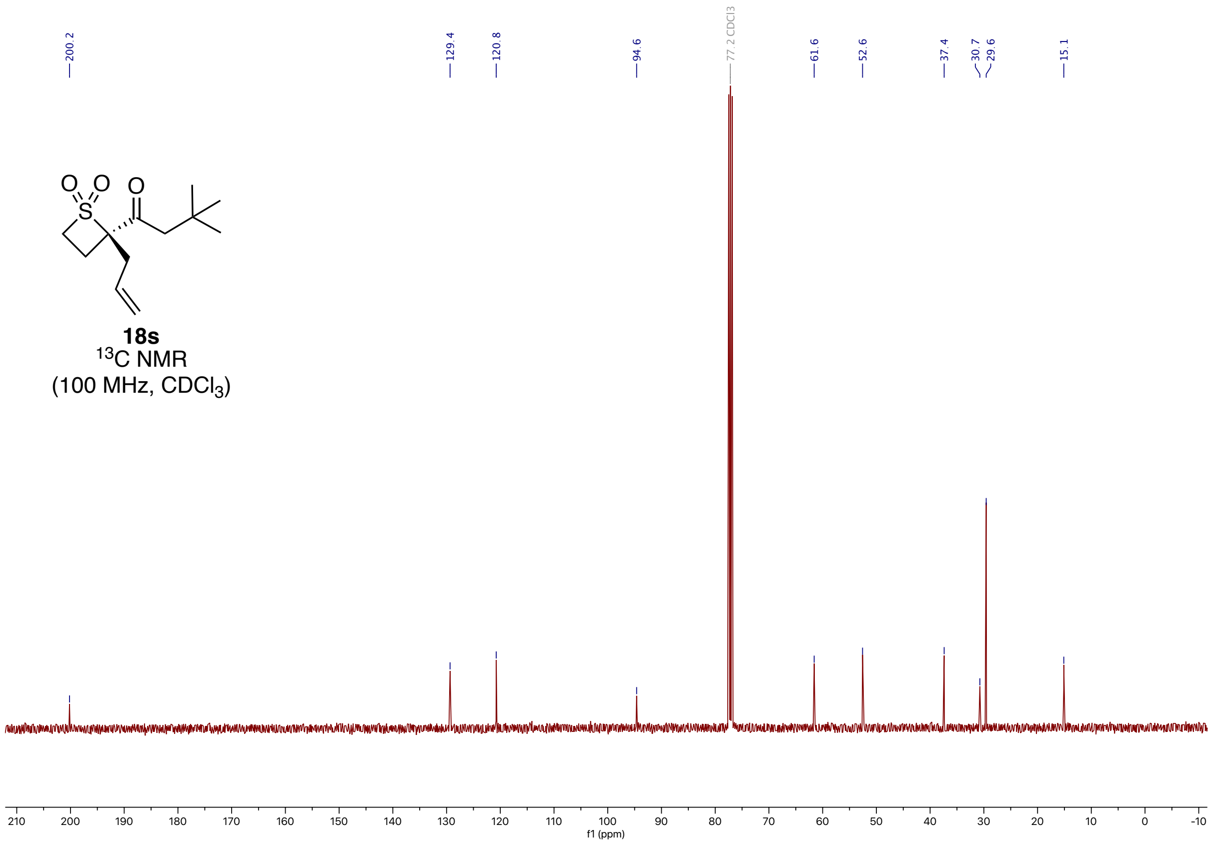
200.2
129.4
120.8
94.6
77.2 CDCl3
61.6
52.6
37.4
30.7
29.6
15.1
18s
^{13}C NMR
(100 MHz, $CDCl_3$)
f1 (ppm)

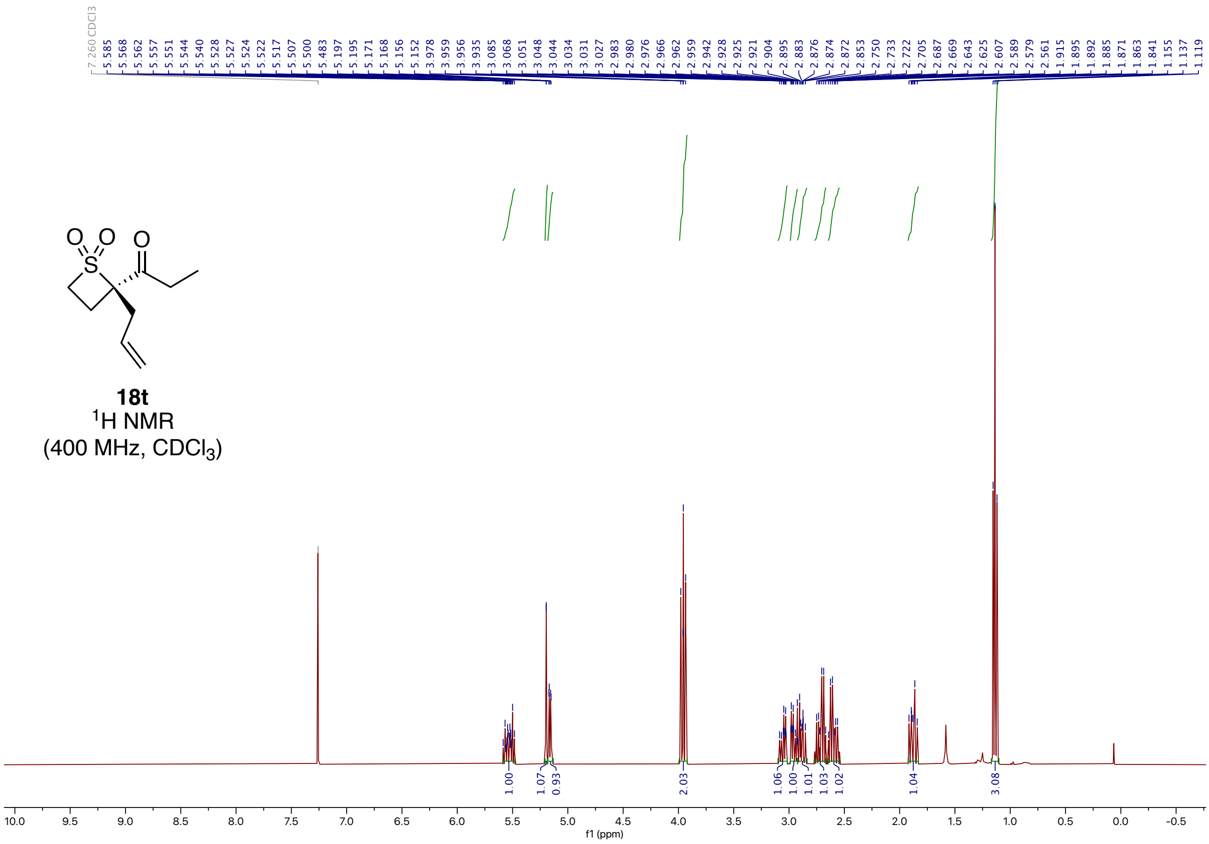

18t
^{1}H NMR
(400 MHz, $CDCl_3$)

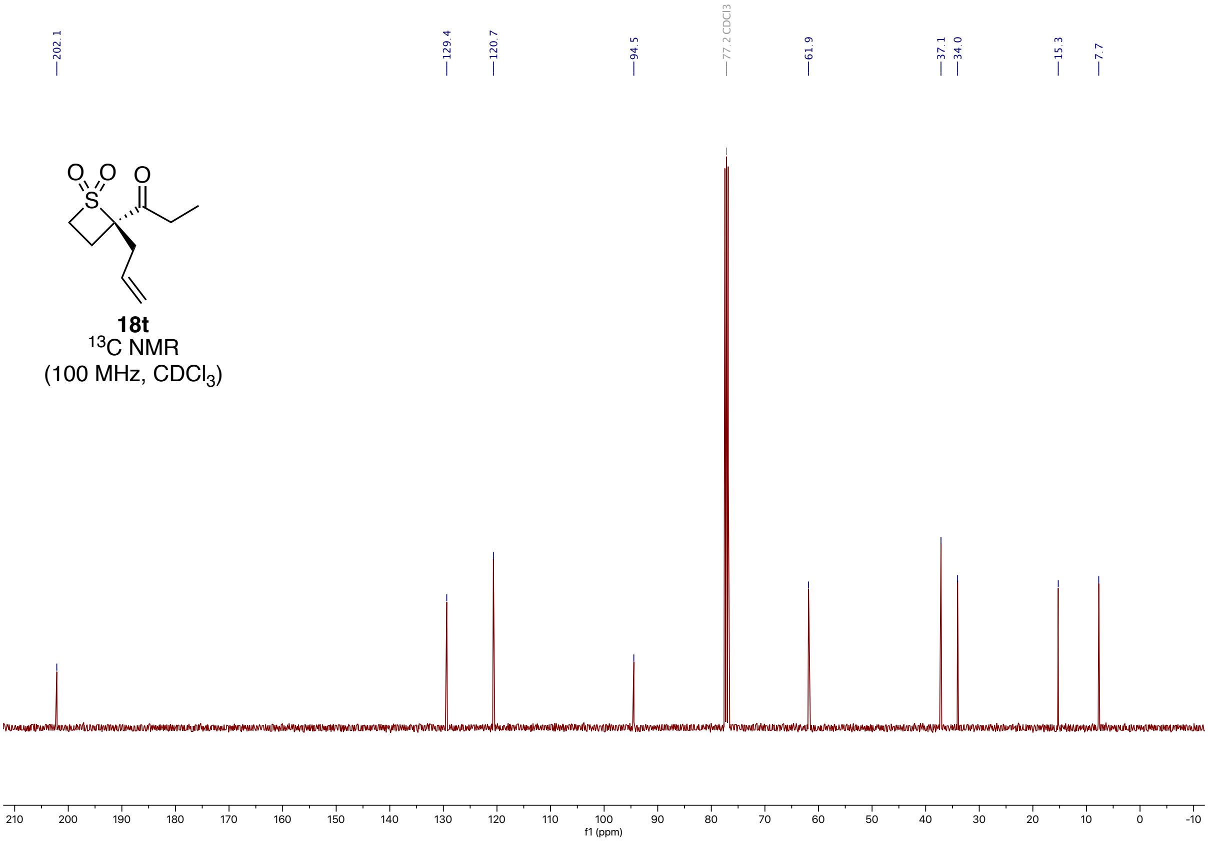

18t
^{13}C NMR
(100 MHz, $CDCl_3$)

18u
^{1}H NMR
(400 MHz, $CDCl_3$)

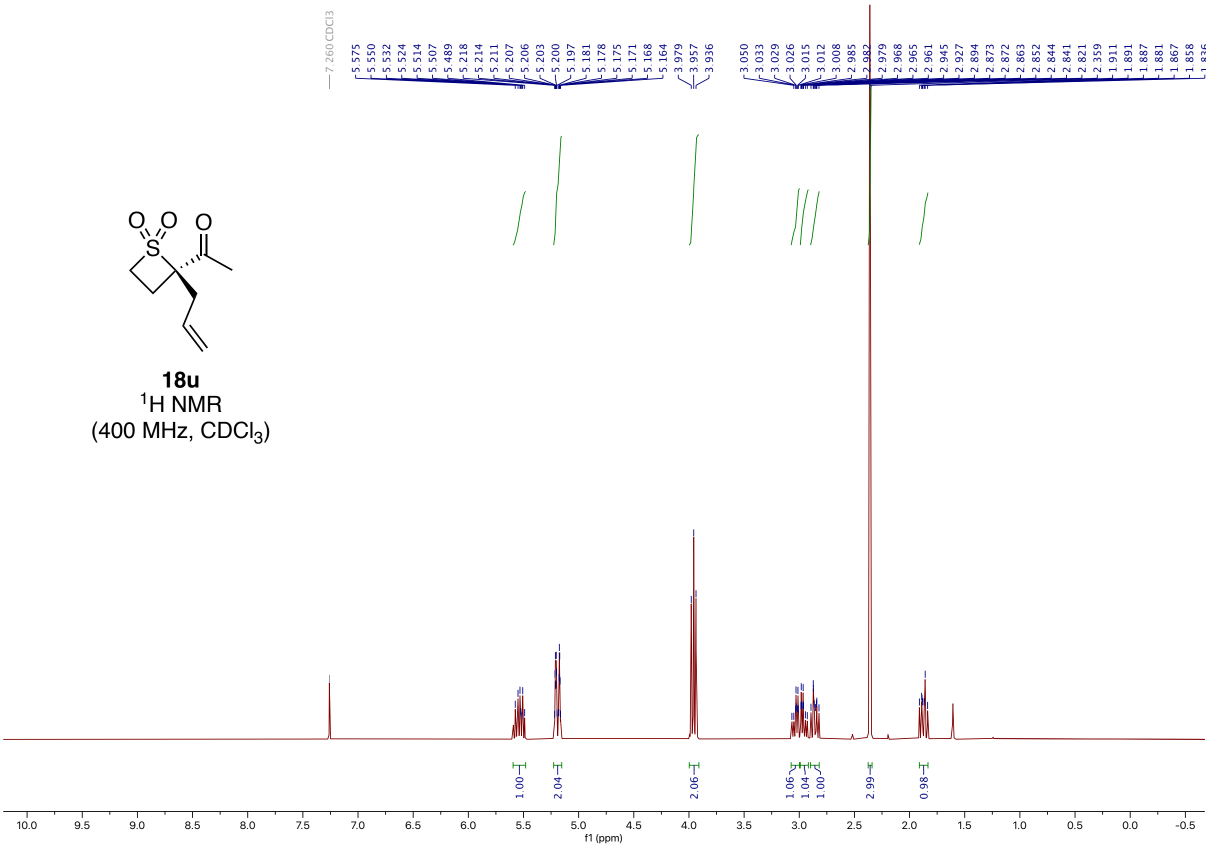

18u
^{13}C NMR
(100 MHz, $CDCl_3$)

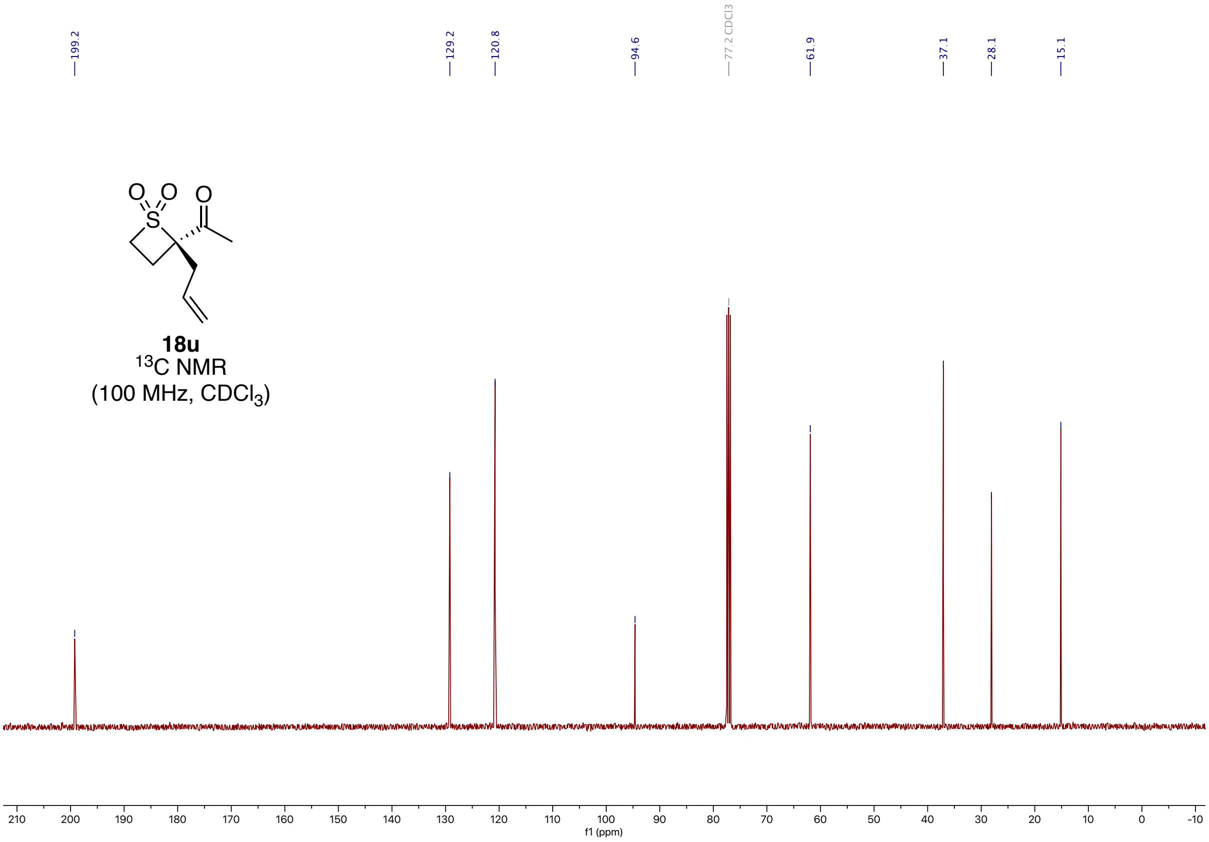

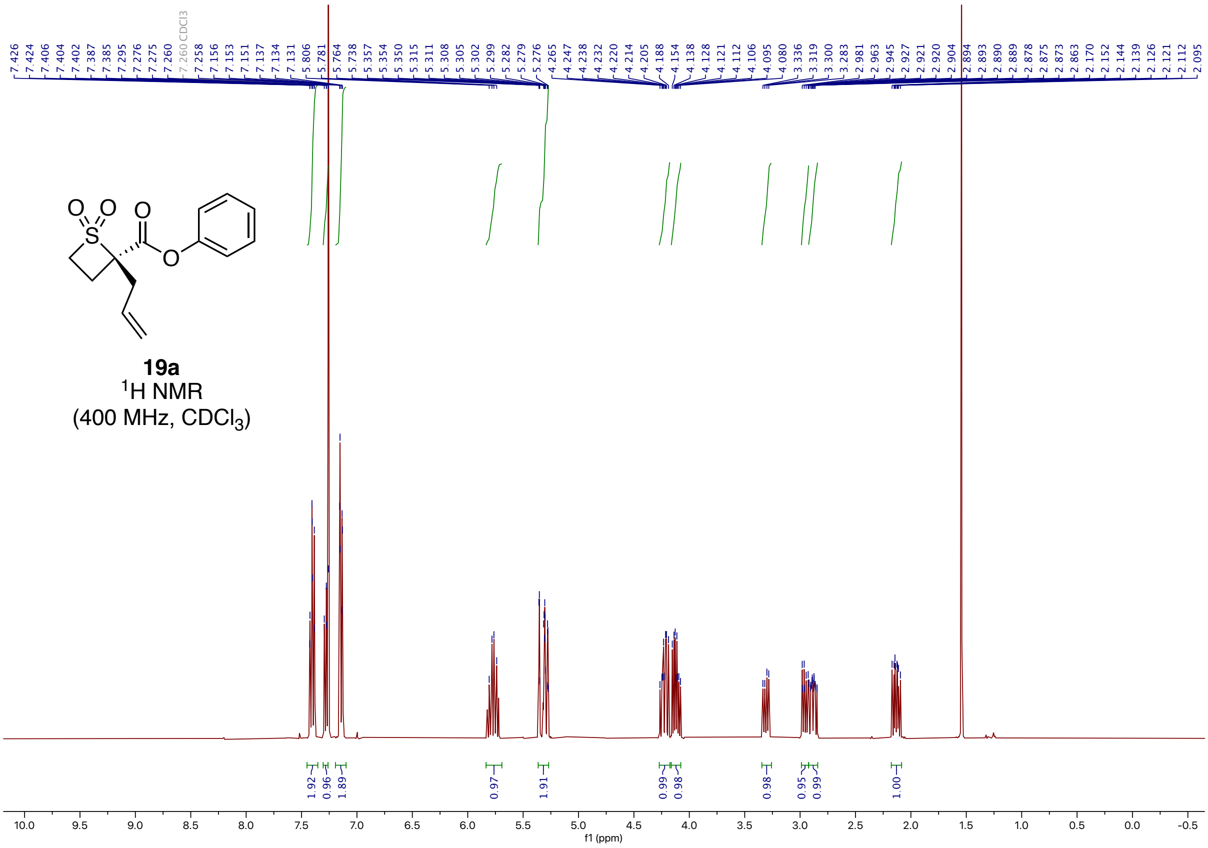
7.426 7.424 7.406 7.404 7.402 7.387 7.385 7.295 7.276 7.275 7.260 CDCl3 7.258 7.156 7.153 7.151 7.137 7.134 7.131 5.806 5.781 5.764 5.738 5.357 5.354 5.350 5.315 5.311 5.308 5.305 5.302 5.299 5.282 5.279 5.276 4.265 4.247 4.238 4.232 4.220 4.214 4.205 4.188 4.154 4.138 4.128 4.121 4.112 4.106 4.095 4.080 3.336 3.319 3.300 3.283 2.981 2.963 2.945 2.927 2.921 2.920 2.904 2.894 2.893 2.890 2.889 2.878 2.875 2.873 2.863 2.170 2.152 2.144 2.139 2.126 2.121 2.112 2.095
19a
^{1}H NMR
(400 MHz, $CDCl_3$)
1.92 0.96 1.89 0.97 1.91 0.99 0.98 0.98 0.95 0.99 1.00
10.0 9.5 9.0 8.5 8.0 7.5 7.0 6.5 6.0 5.5 5.0 4.5 4.0 3.5 3.0 2.5 2.0 1.5 1.0 0.5 0.0 -0.5
f1 (ppm)

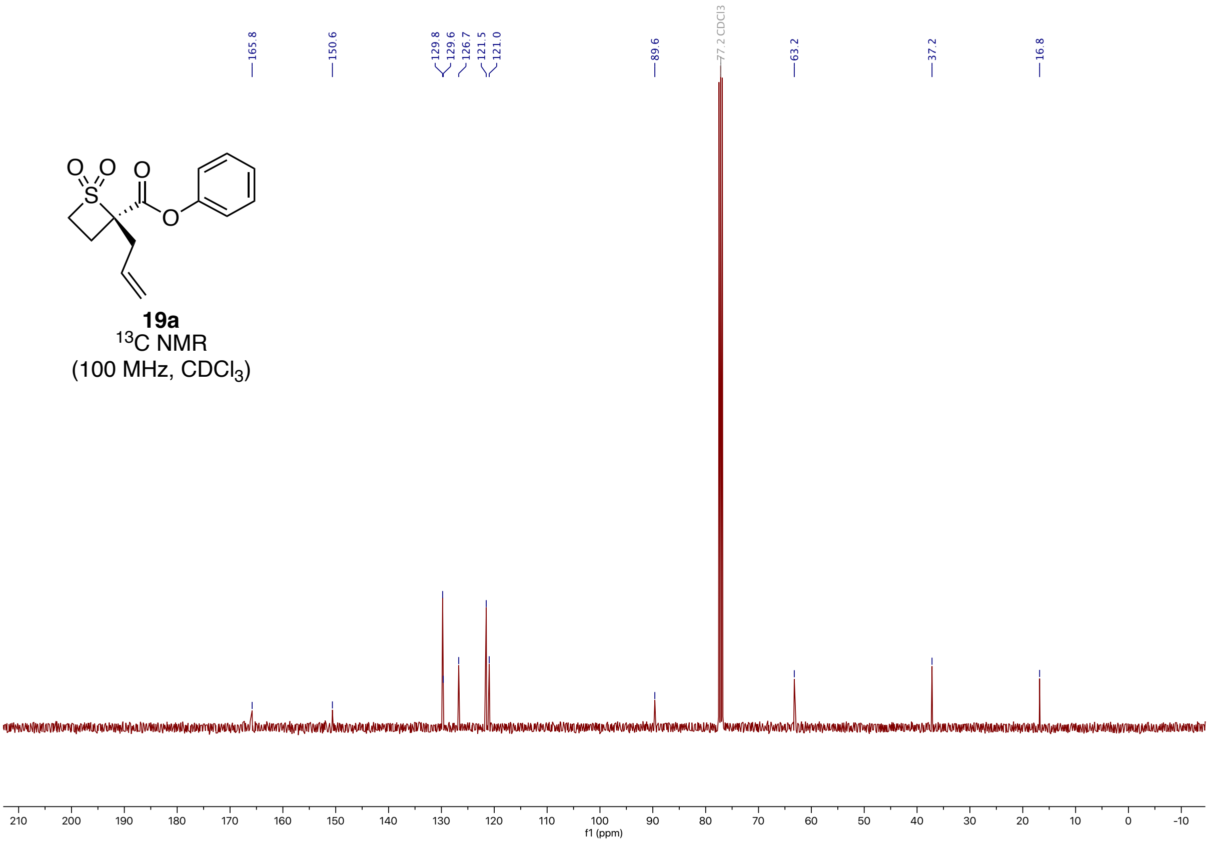
165.8 150.6 129.8 129.6 126.7 121.5 121.0 89.6 77.2 CDCl3 63.2 37.2 16.8
19a
^{13}C NMR
(100 MHz, $CDCl_3$)
210 200 190 180 170 160 150 140 130 120 110 100 90 80 70 60 50 40 30 20 10 0 -10
f1 (ppm)

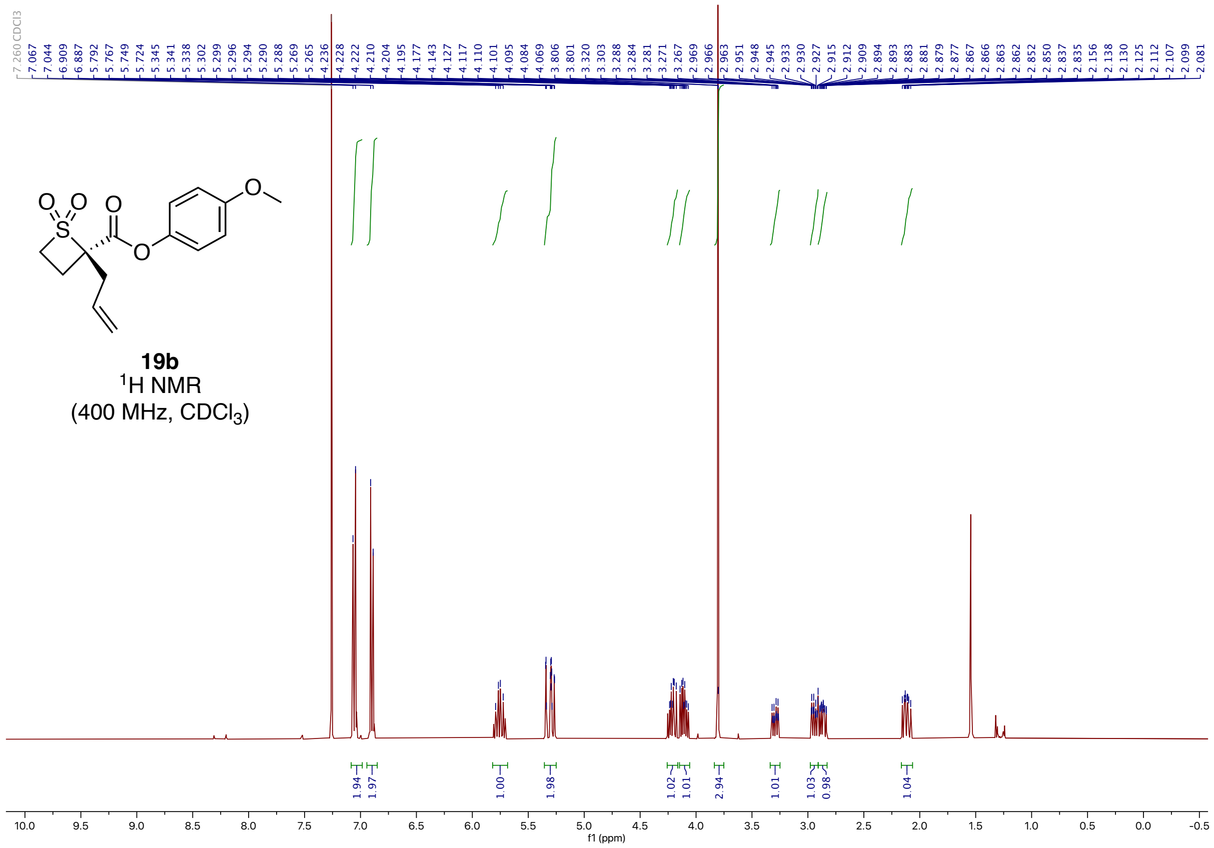
19b
^{1}H NMR
(400 MHz, $CDCl_3$)
1.94
1.97
1.00
1.98
1.02
1.01
2.94
1.01
1.03
0.98
1.04
f1 (ppm)

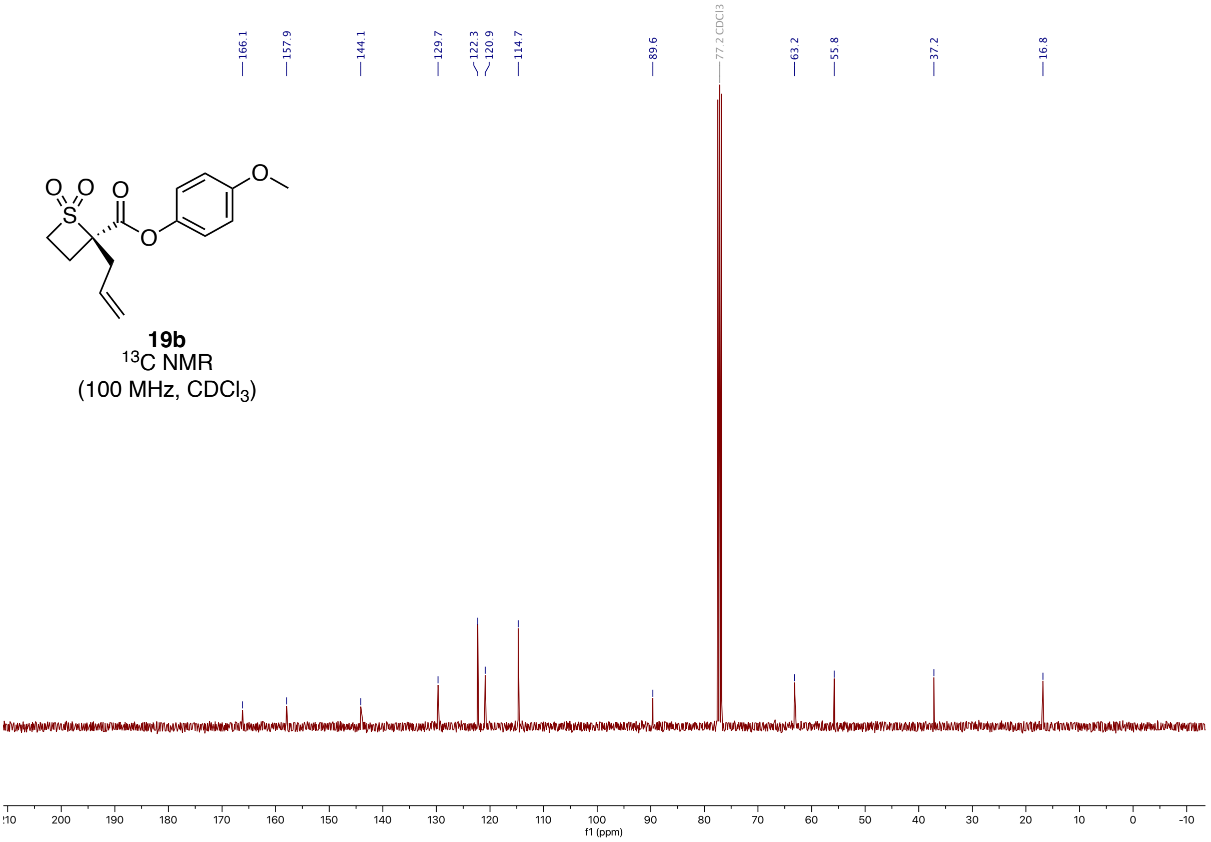
166.1
157.9
144.1
129.7
122.3
120.9
114.7
89.6
77.2 CDCl3
63.2
55.8
37.2
16.8
19b
^{13}C NMR
(100 MHz, $CDCl_3$)
f1 (ppm)

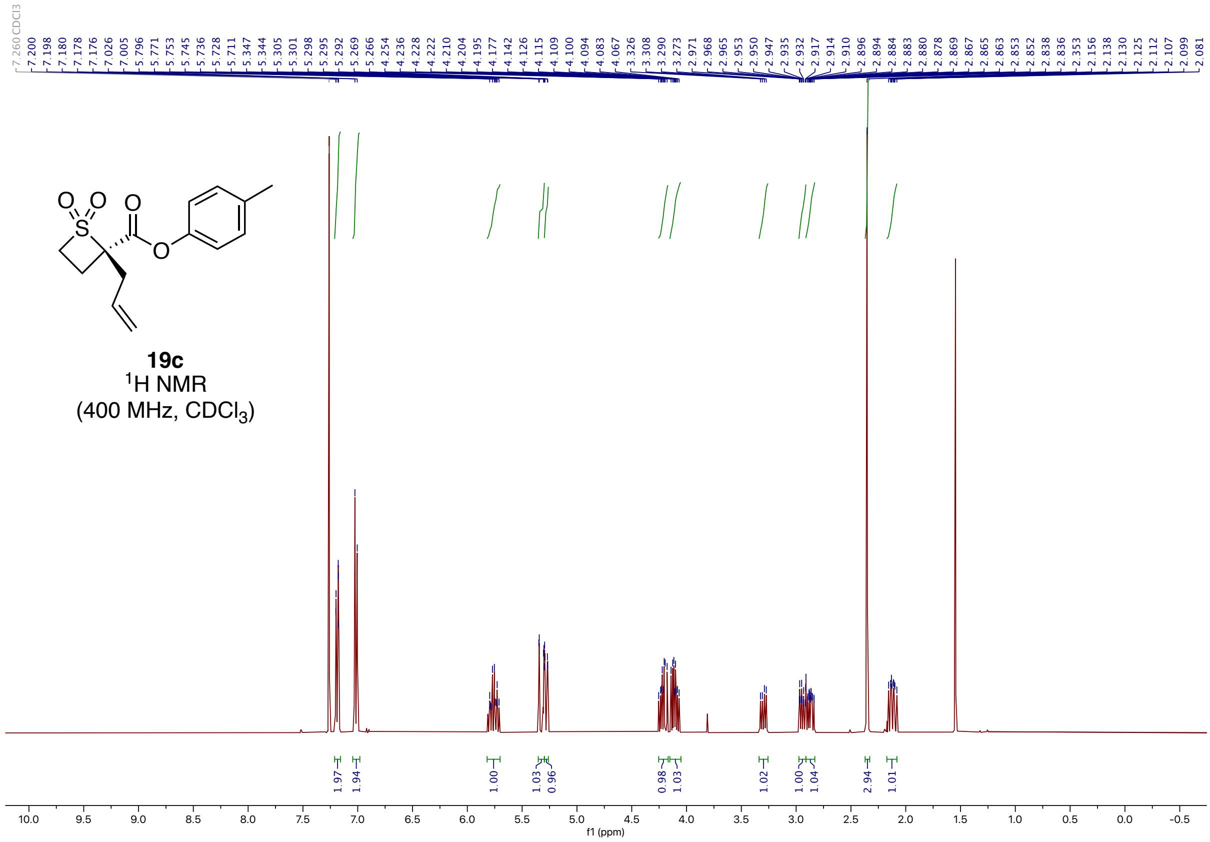
19c
^{1}H NMR
(400 MHz, CDCl$_3$)
f1 (ppm)

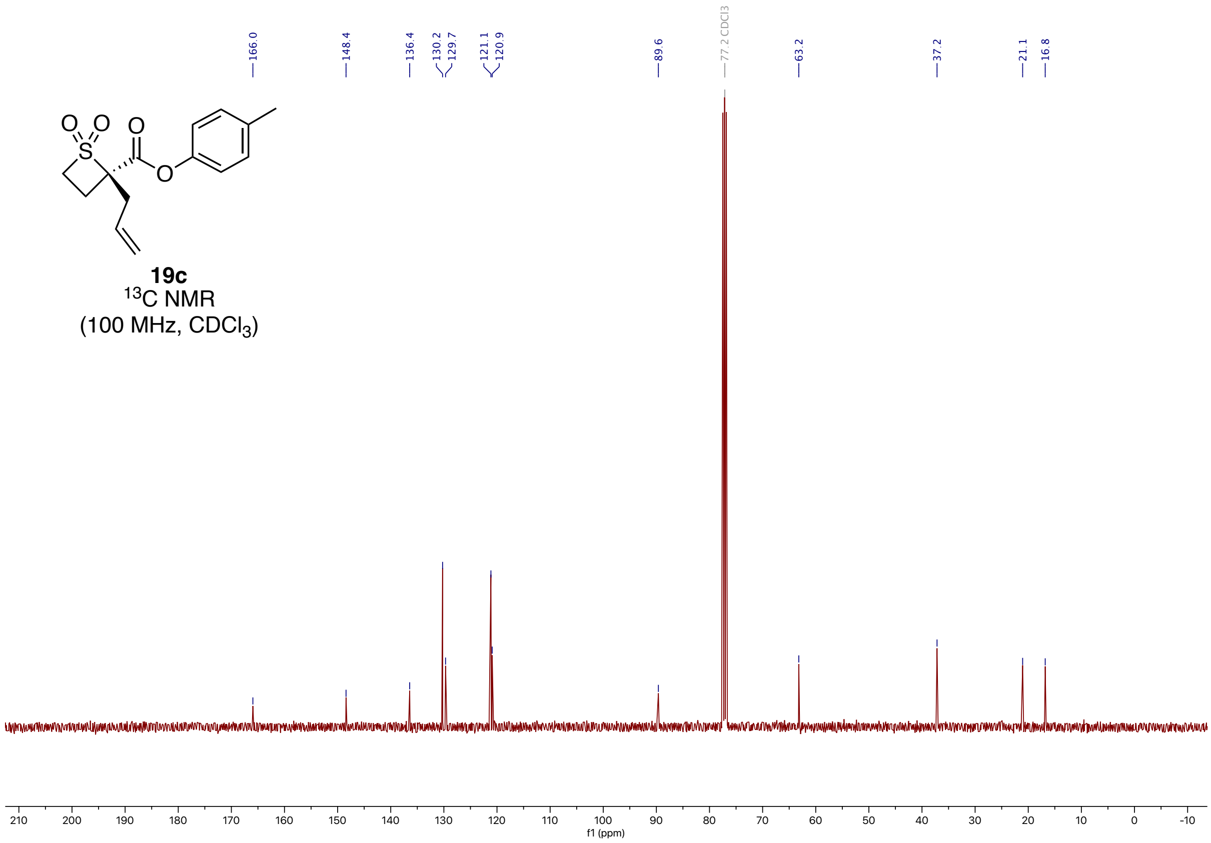
19c
^{13}C NMR
(100 MHz, CDCl$_3$)
f1 (ppm)

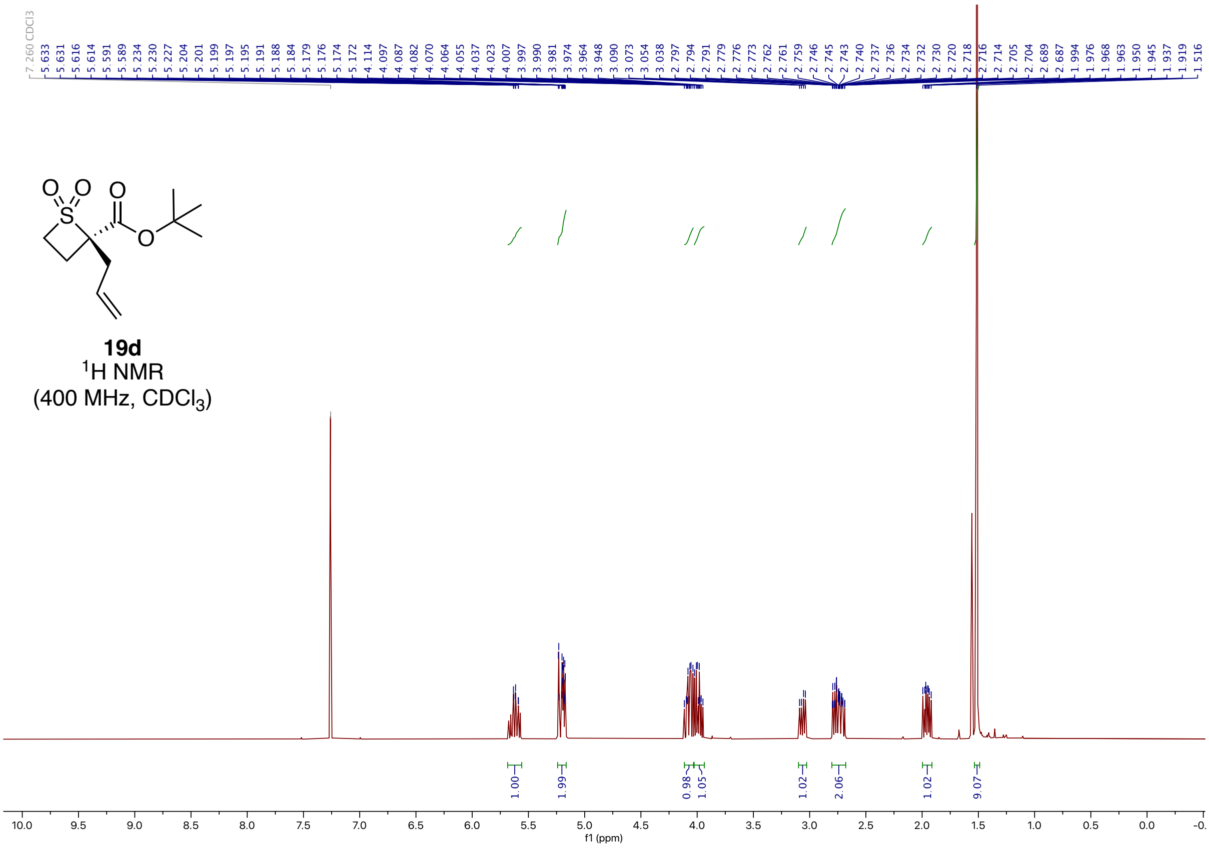

19d
^1H NMR
(400 MHz, $CDCl_3$)

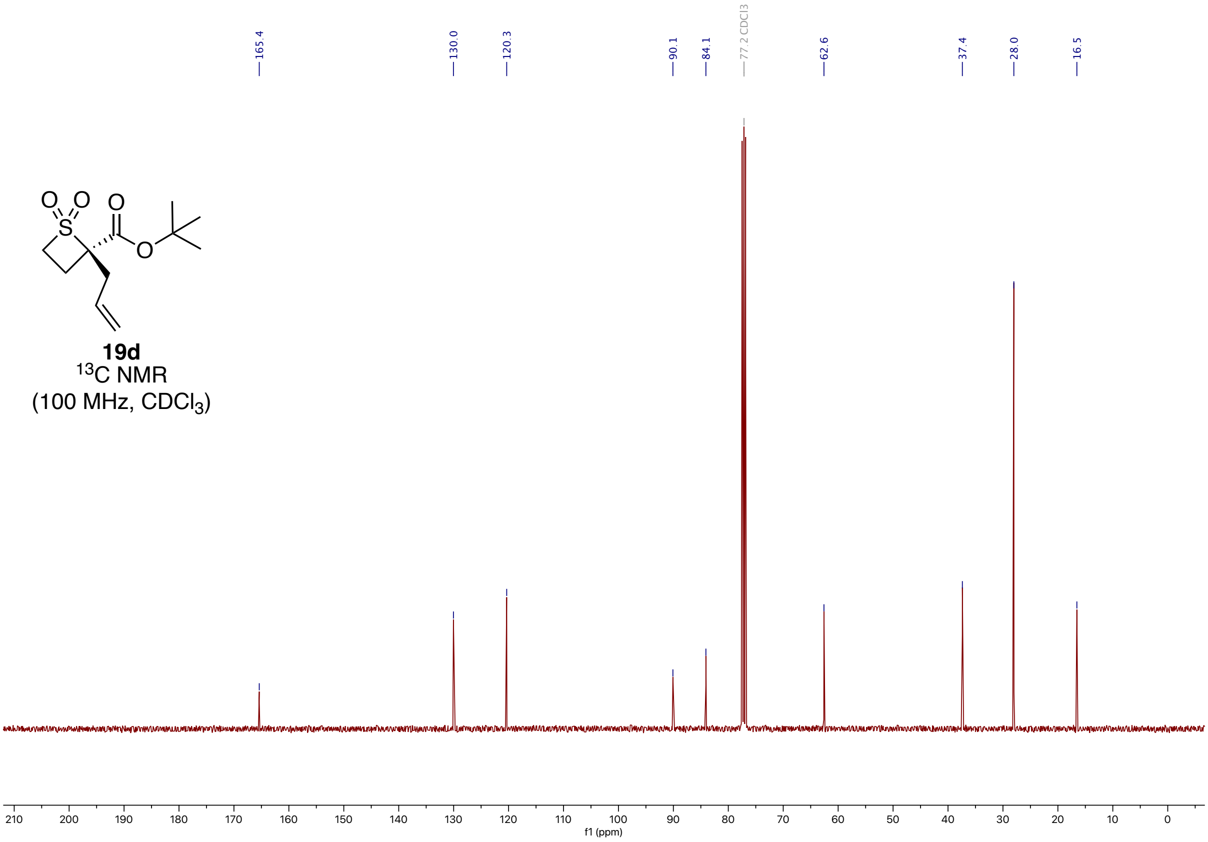

19d
^{13}C NMR
(100 MHz, $CDCl_3$)

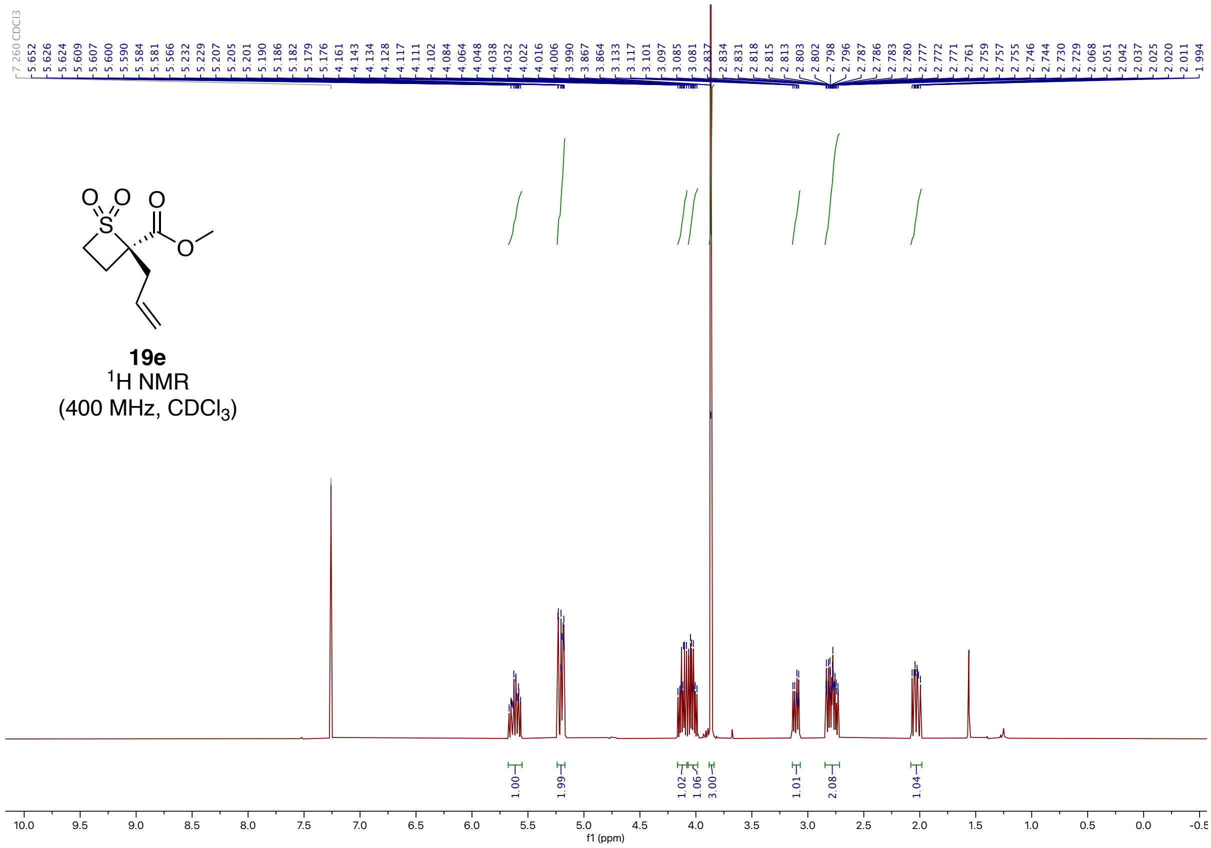
7.260 CDCl3
5.652
5.626
5.624
5.609
5.607
5.600
5.590
5.584
5.581
5.566
5.232
5.229
5.207
5.205
5.201
5.190
5.186
5.182
5.179
5.176
4.161
4.143
4.134
4.128
4.117
4.111
4.102
4.084
4.064
4.048
4.038
4.032
4.022
4.016
4.006
3.990
3.867
3.864
3.133
3.117
3.101
3.097
3.085
3.081
2.837
2.834
2.831
2.818
2.815
2.813
2.803
2.802
2.798
2.796
2.787
2.786
2.783
2.780
2.777
2.772
2.771
2.761
2.759
2.757
2.755
2.746
2.744
2.730
2.729
2.068
2.051
2.042
2.037
2.025
2.020
2.011
1.994
19e
1H NMR
(400 MHz, CDCl3)
1.00
1.99
1.02
1.06
3.00
1.01
2.08
1.04
f1 (ppm)

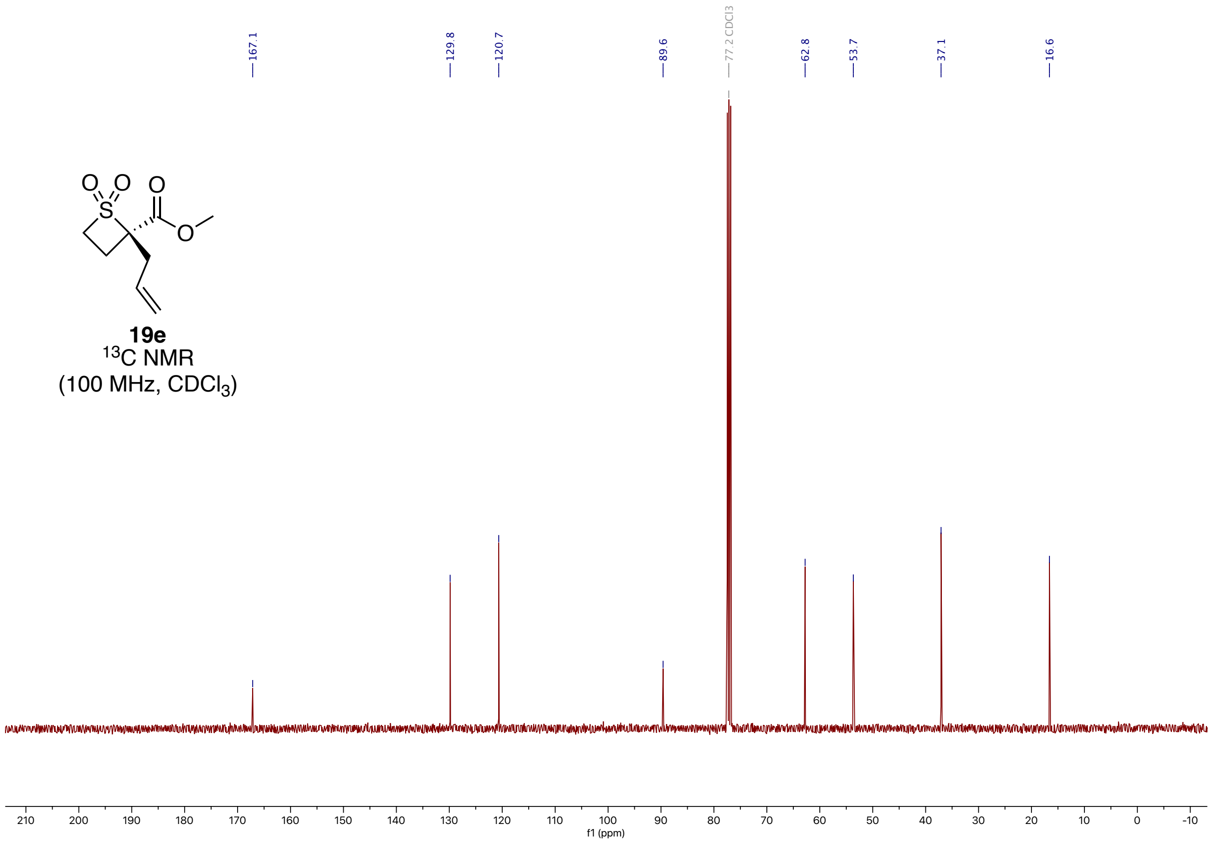
167.1
129.8
120.7
89.6
77.2 CDCl3
62.8
53.7
37.1
16.6
19e
13C NMR
(100 MHz, CDCl3)
f1 (ppm)

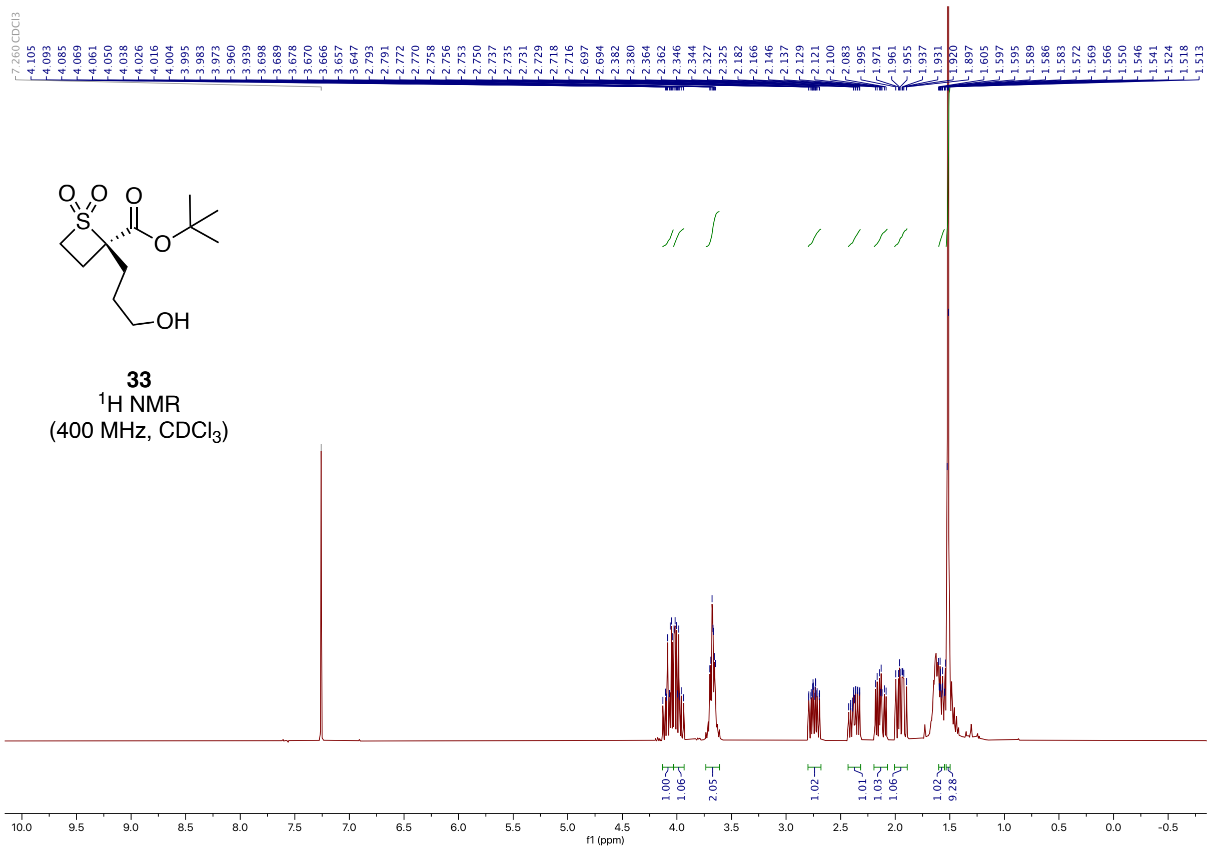

33
^1H NMR
(400 MHz, $CDCl_3$)

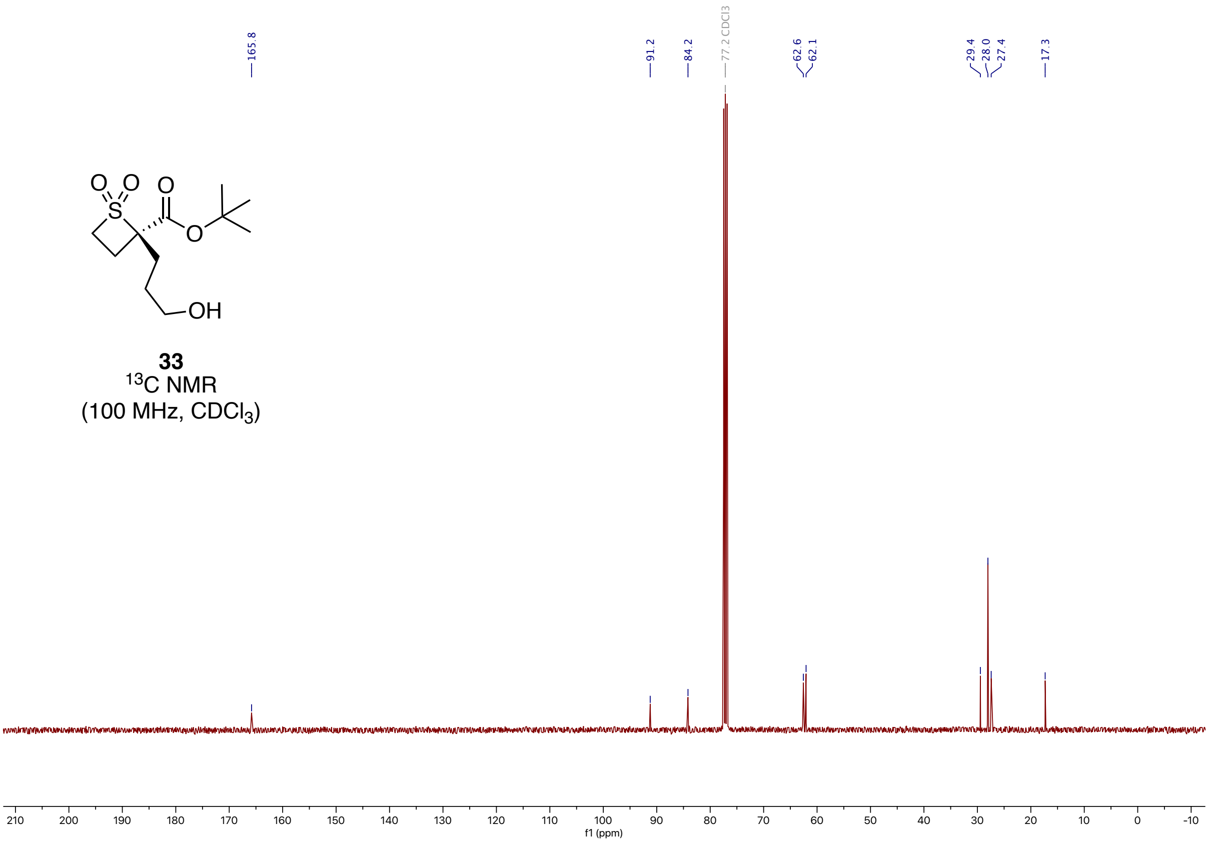

33
^{13}C NMR
(100 MHz, $CDCl_3$)

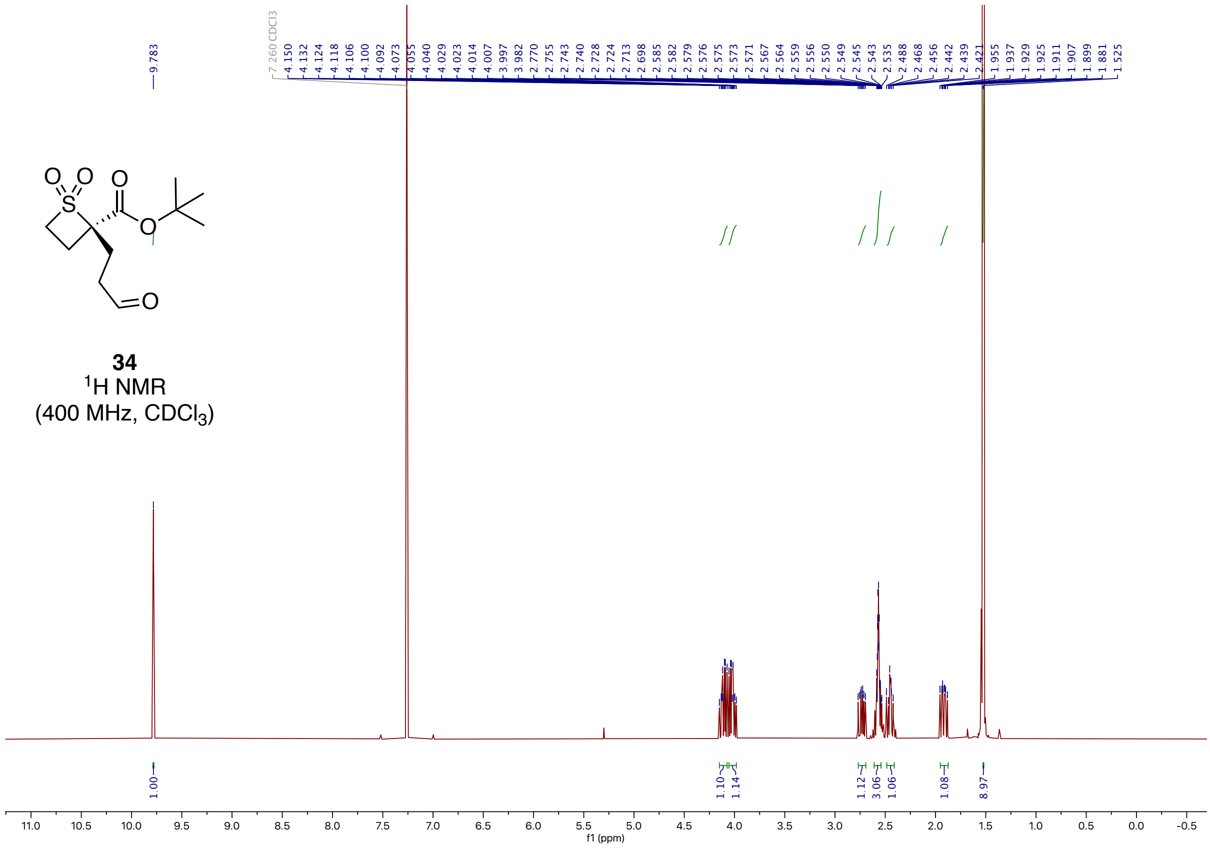

34
^{1}H NMR
(400 MHz, CDCl$_3$)
9.783
7.260 CDCl3
4.150
4.132
4.124
4.118
4.106
4.100
4.092
4.073
4.055
4.040
4.029
4.023
4.014
4.007
3.997
3.982
2.770
2.755
2.743
2.740
2.728
2.724
2.713
2.698
2.585
2.582
2.579
2.576
2.575
2.573
2.571
2.567
2.564
2.559
2.556
2.550
2.549
2.545
2.543
2.535
2.488
2.468
2.456
2.442
2.439
2.421
1.955
1.937
1.929
1.925
1.911
1.907
1.899
1.881
1.525
1.00
1.10
1.14
1.12
3.06
1.06
1.08
8.97
f1 (ppm)

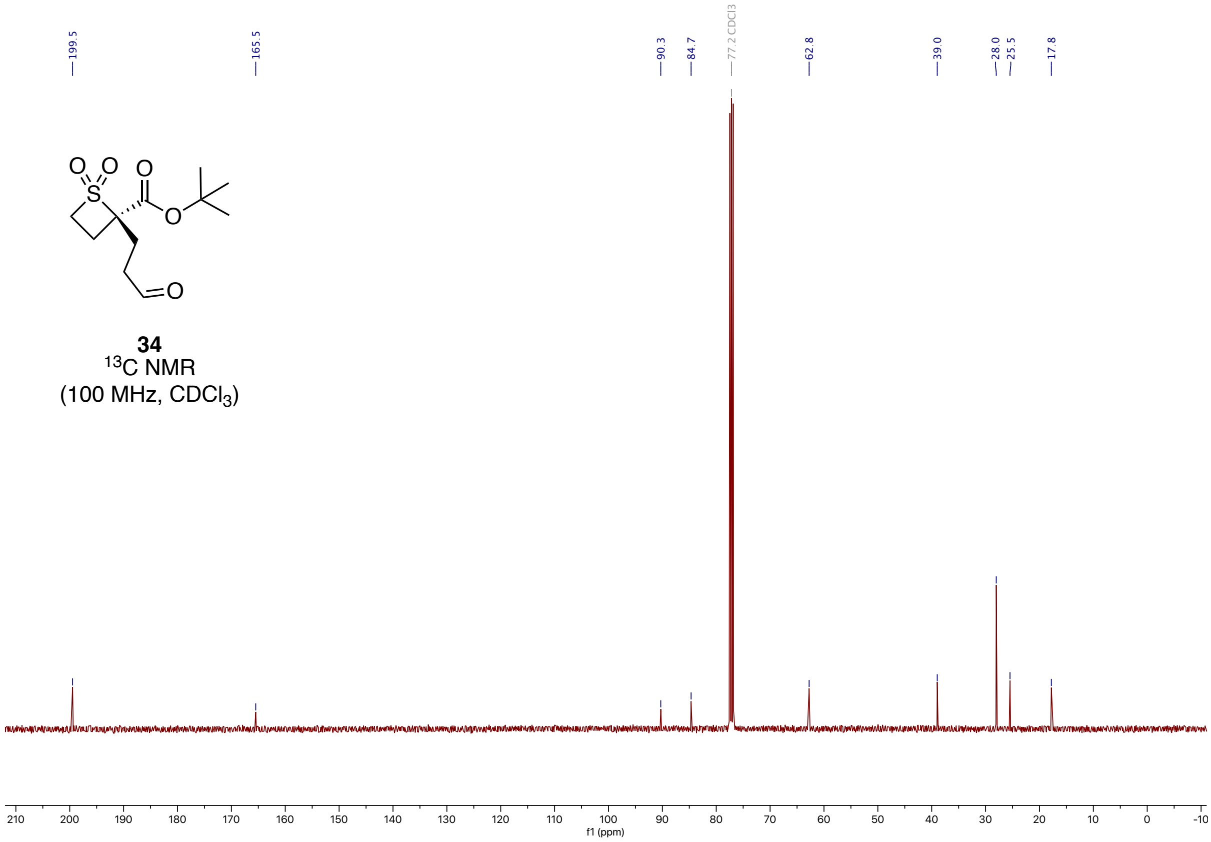

34
^{13}C NMR
(100 MHz, CDCl$_3$)
199.5
165.5
90.3
84.7
77.2 CDCl3
62.8
39.0
28.0
25.5
17.8
f1 (ppm)

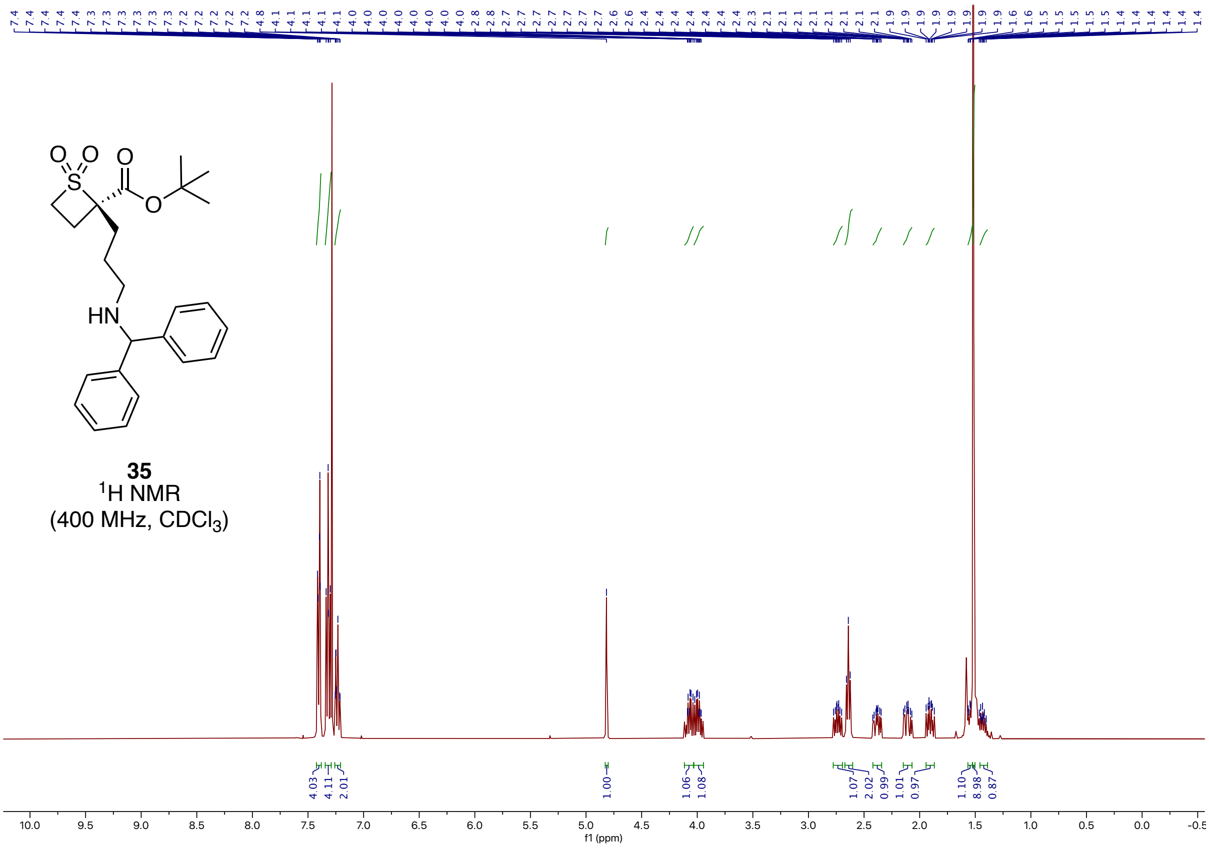

35
^{1}H NMR
(400 MHz, $CDCl_3$)

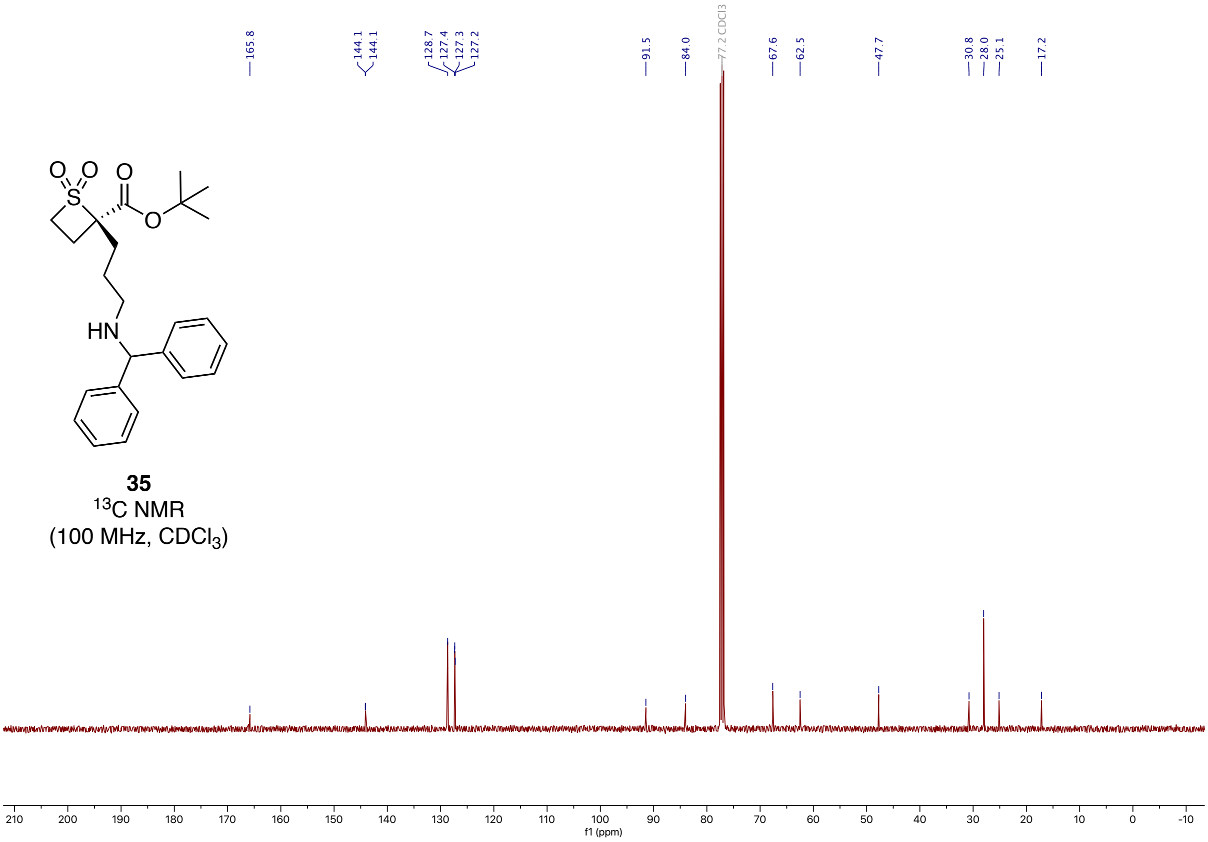

35
^{13}C NMR
(100 MHz, $CDCl_3$)

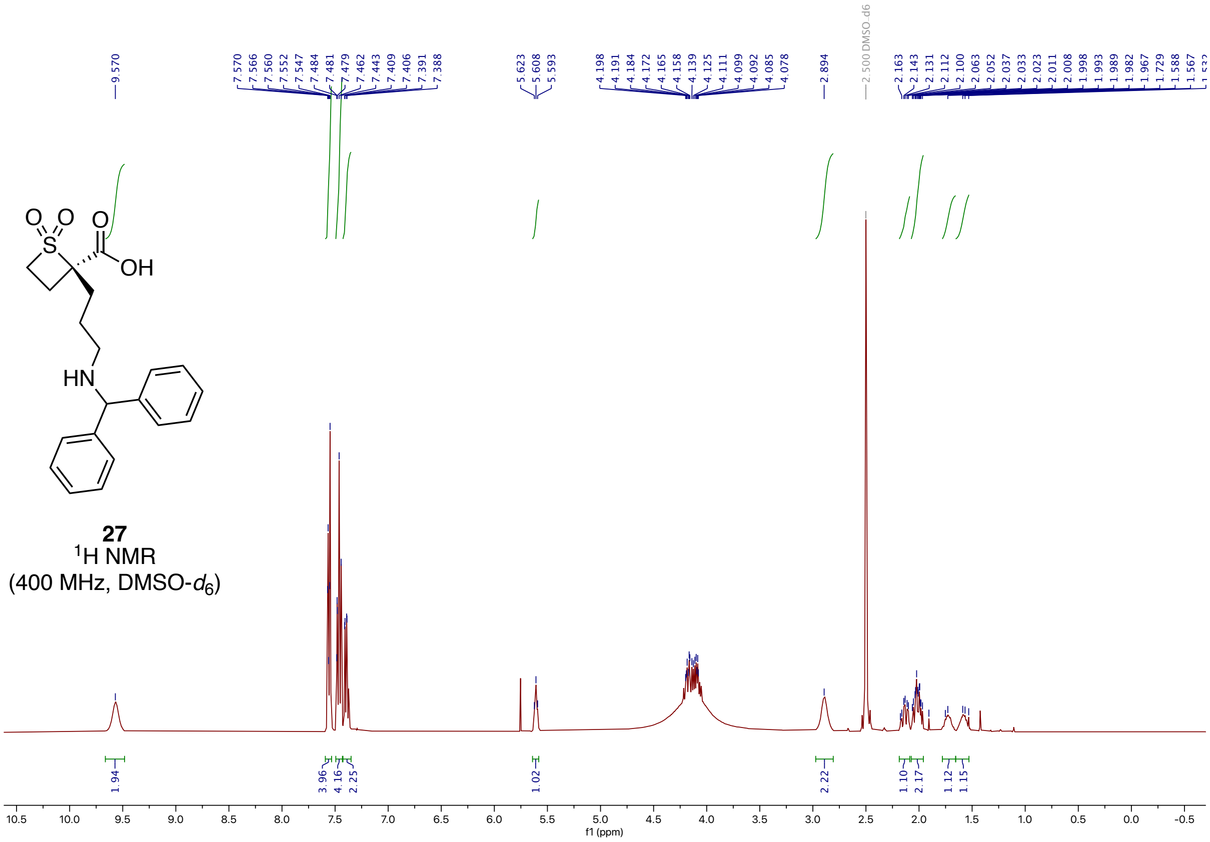
27
^{1}H NMR
(400 MHz, DMSO-d_6)
f1 (ppm)

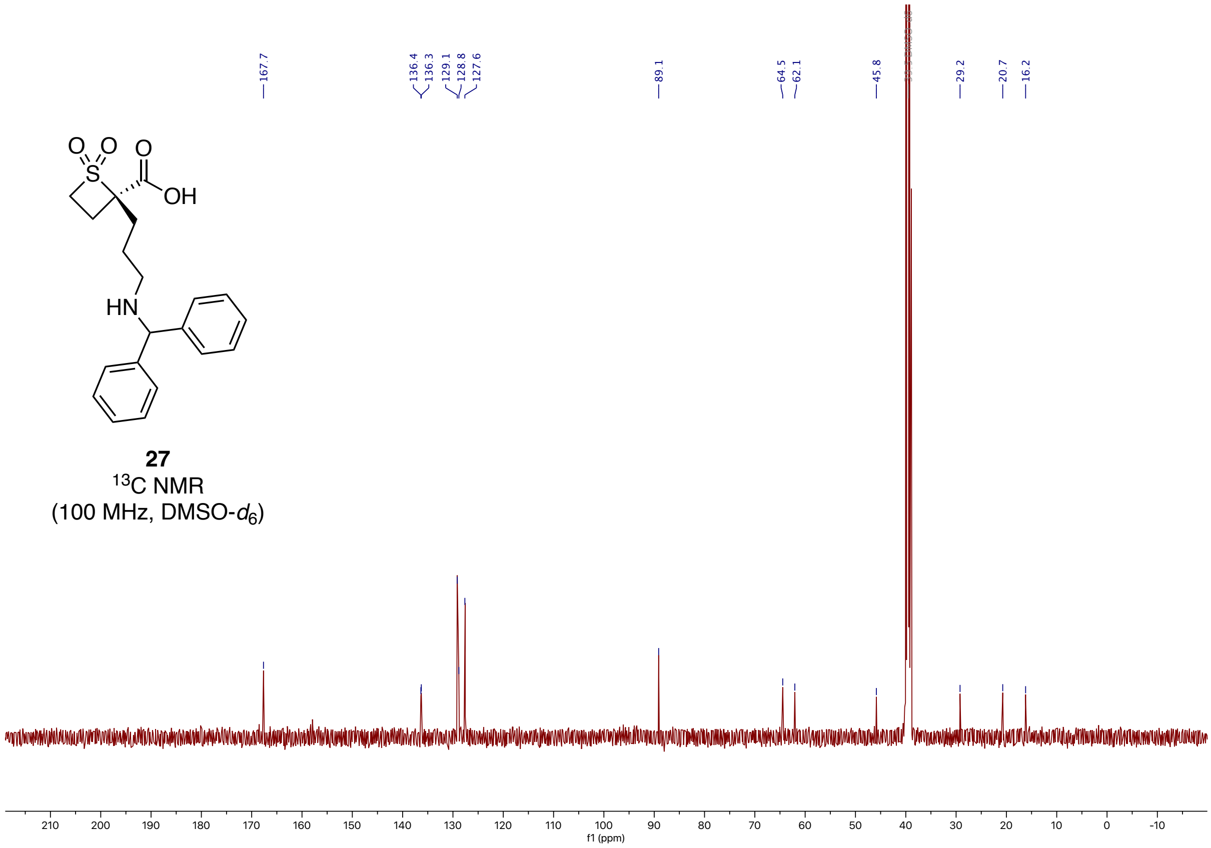
27
^{13}C NMR
(100 MHz, DMSO-d_6)
167.7
136.4
136.3
129.1
128.8
127.6
89.1
64.5
62.1
45.8
29.2
20.7
16.2
f1 (ppm)

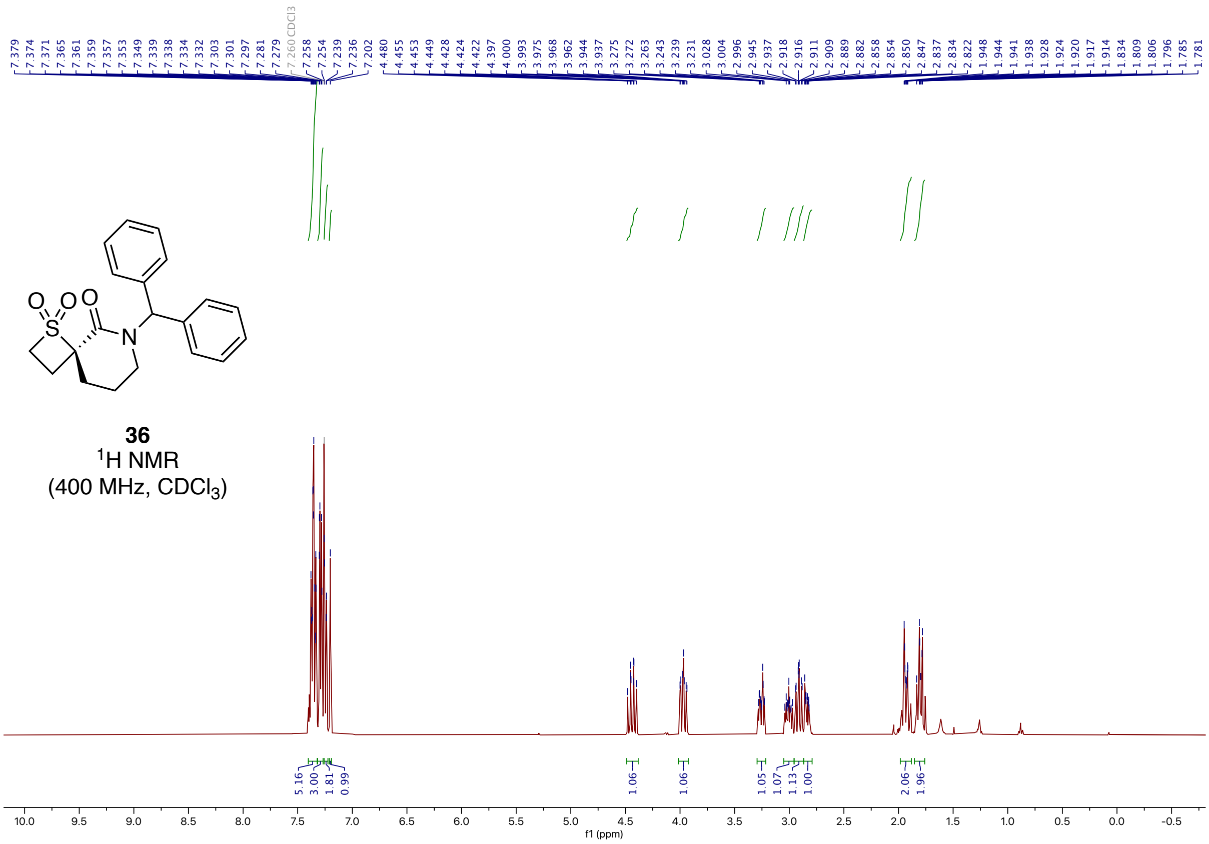

36
^{1}H NMR
(400 MHz, $CDCl_3$)

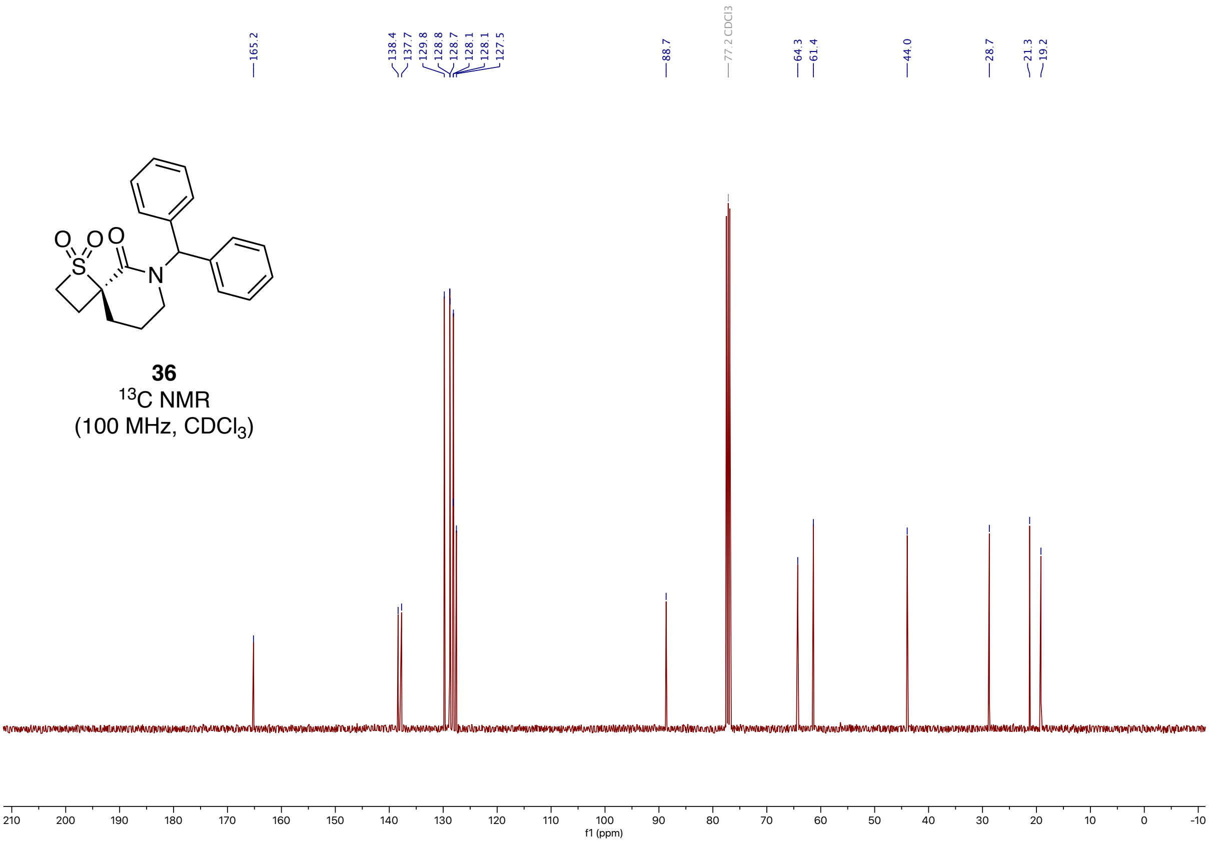

36
^{13}C NMR
(100 MHz, $CDCl_3$)

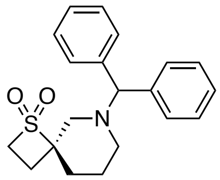

37

^{1}H NMR

(400 MHz, $CDCl_3$)

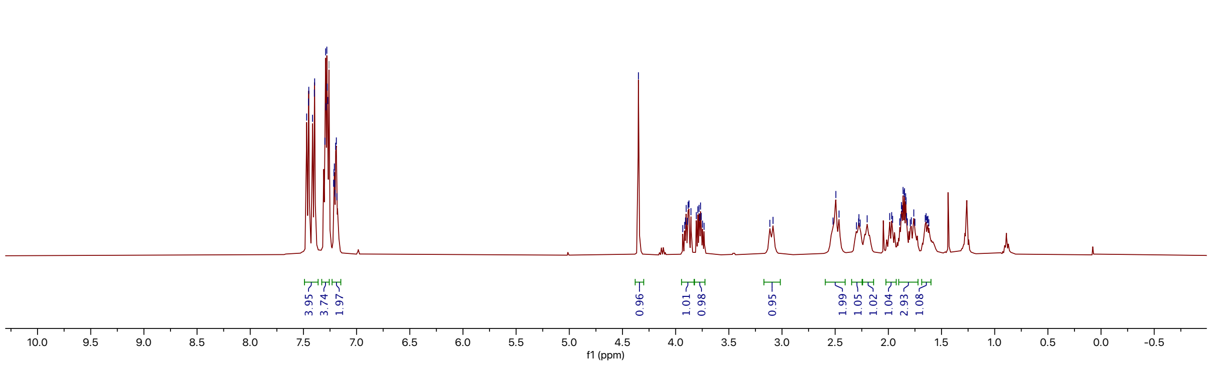

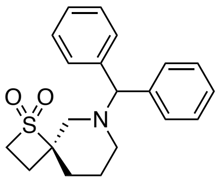

37

^{13}C NMR

(100 MHz, $CDCl_3$)

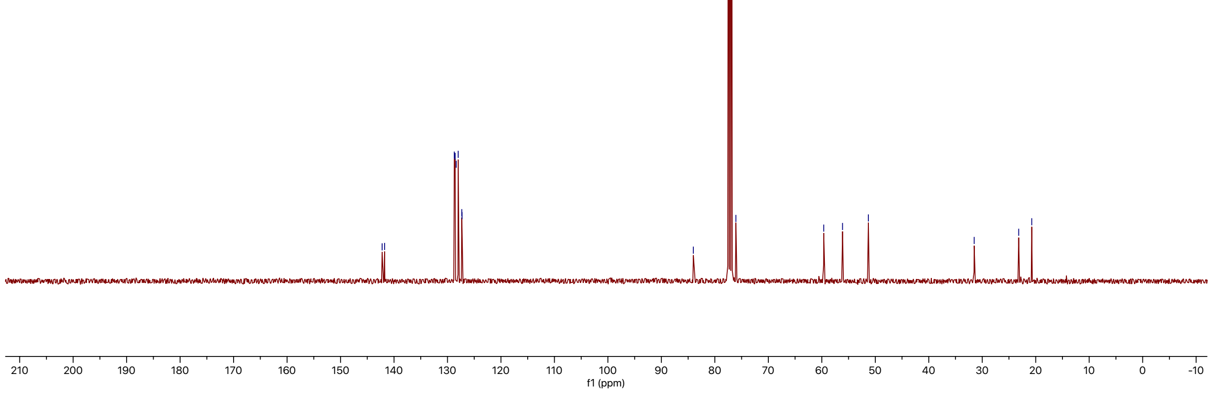

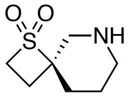

38
^{1}H NMR
(400 MHz, DMSO-d_6)

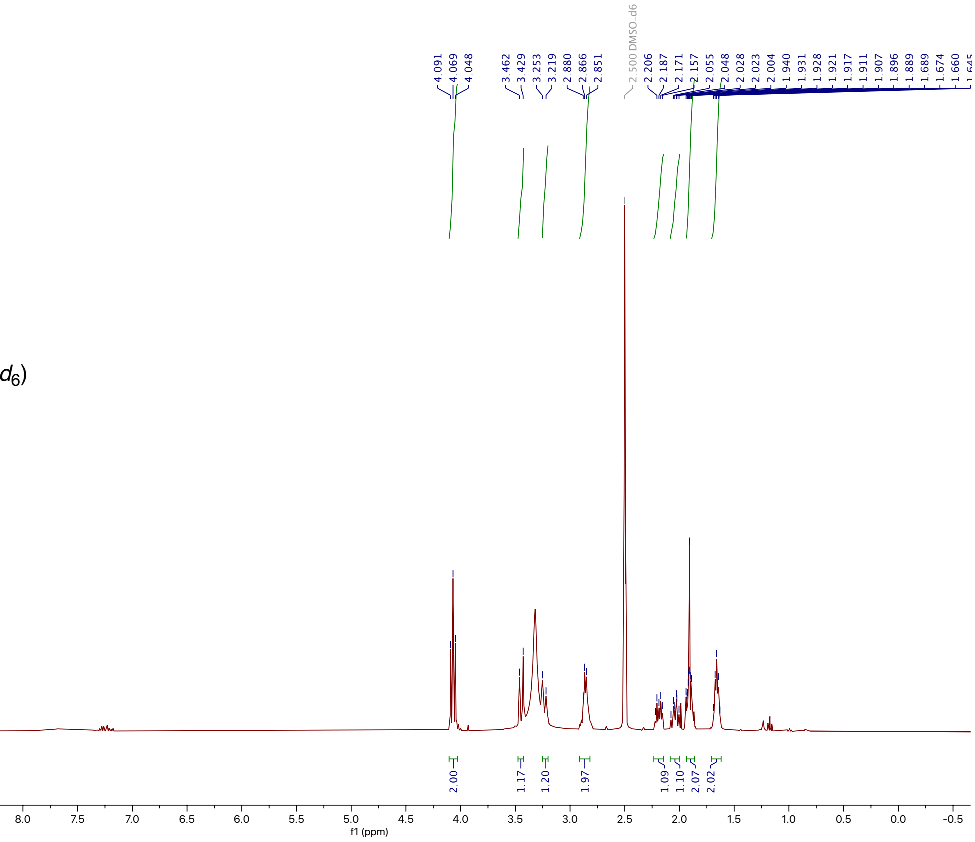

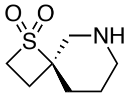

38
^{13}C NMR
(100 MHz, DMSO-d_6)

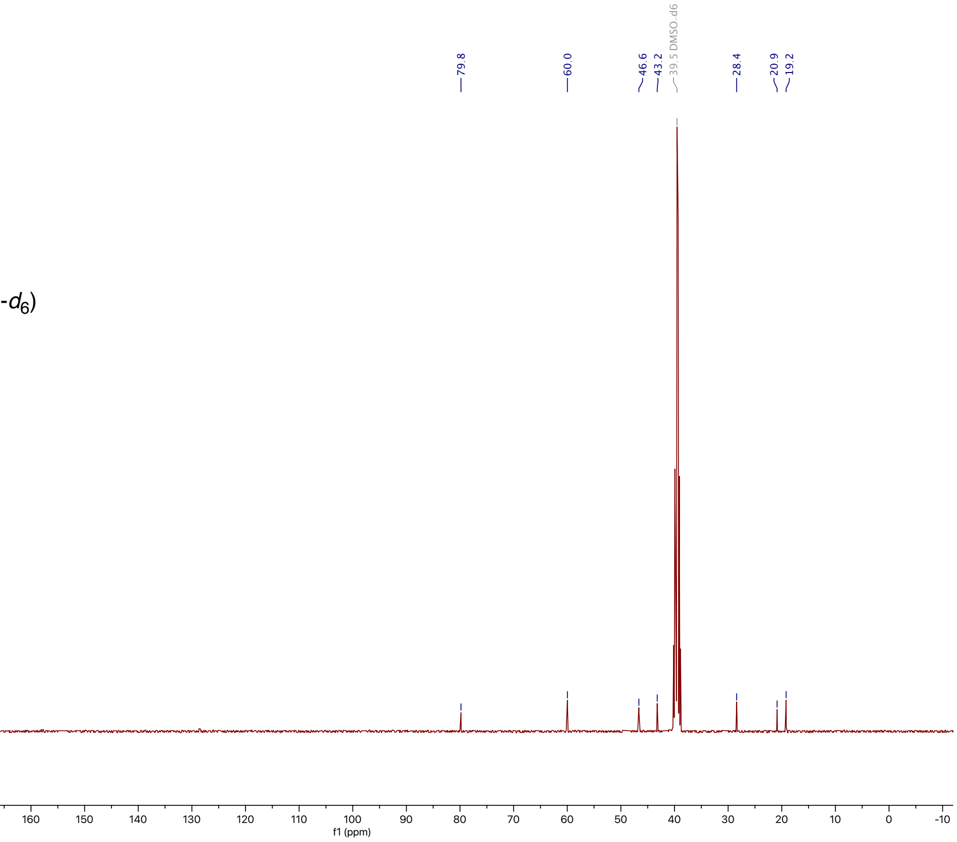

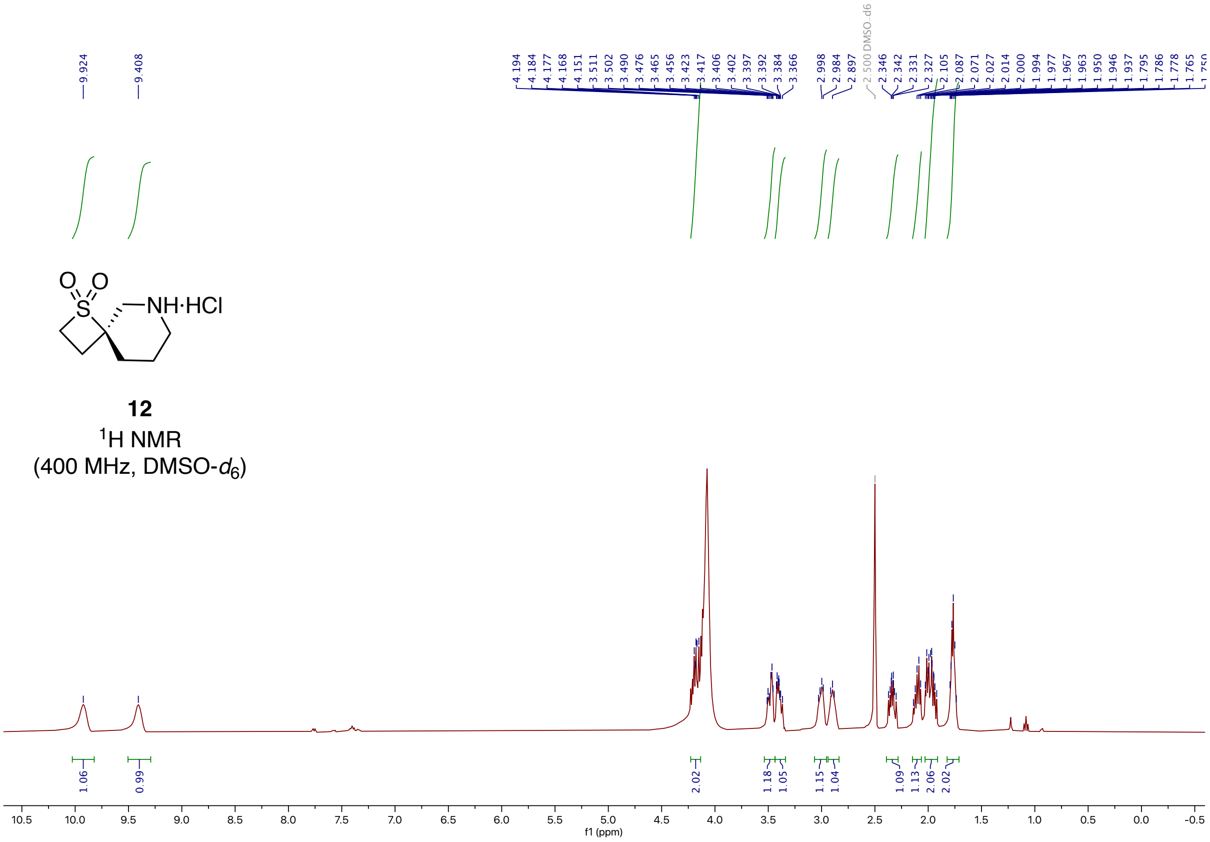
9.924
9.408
4.194 4.184 4.177 4.168 4.151 3.511 3.502 3.490 3.476 3.465 3.456 3.423 3.417 3.406 3.402 3.397 3.392 3.384 3.366 2.998 2.984 2.897 2.500 DMSO_d6 2.346 2.342 2.331 2.327 2.105 2.087 2.071 2.027 2.014 2.000 1.994 1.977 1.967 1.963 1.950 1.946 1.937 1.795 1.786 1.778 1.765
NH·HCl
12
^{1}H NMR
(400 MHz, DMSO-d_6)
1.06 0.99 2.02 1.18 1.05 1.15 1.04 1.09 1.13 2.06 2.02
f1 (ppm)

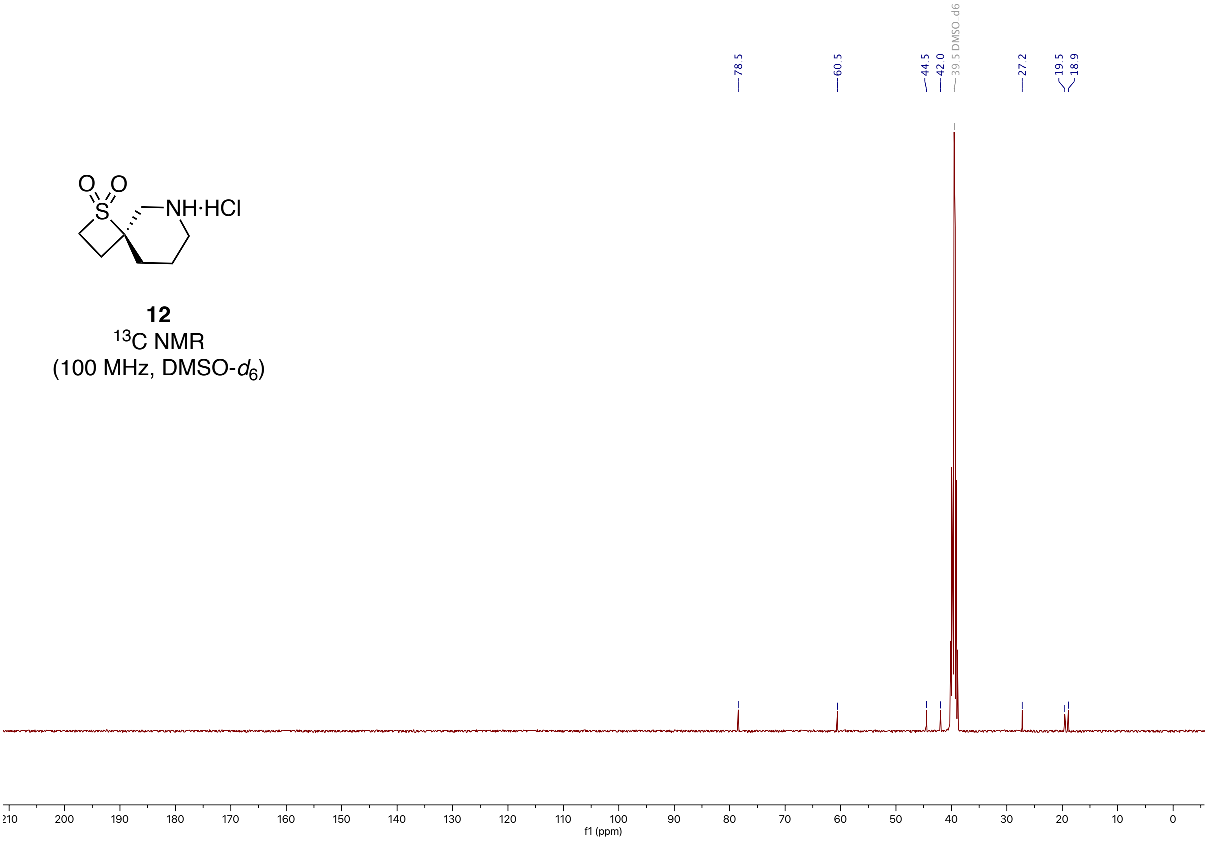
78.5
60.5
44.5
42.0
39.5 DMSO_d6
27.2
19.5
18.9
NH·HCl
12
^{13}C NMR
(100 MHz, DMSO-d_6)
f1 (ppm)

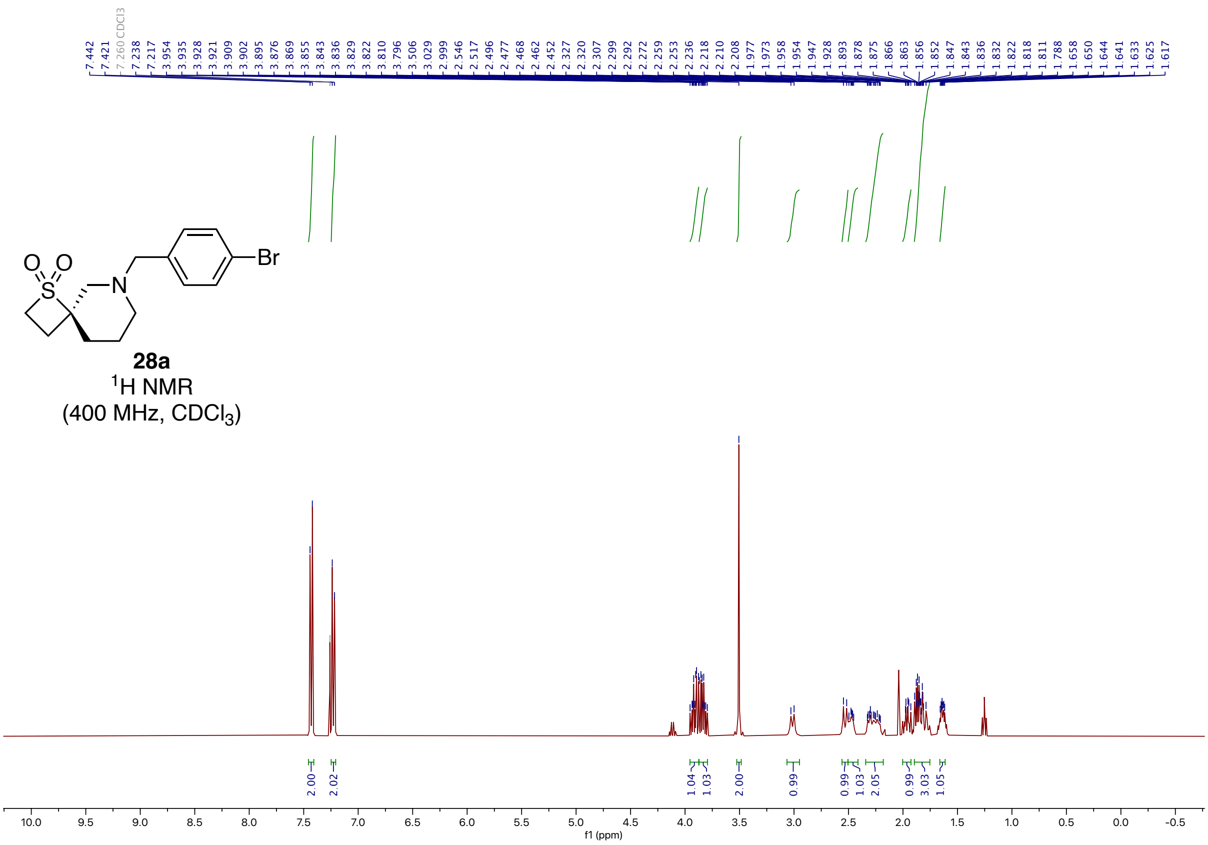

28a
^{1}H NMR
(400 MHz, $CDCl_3$)

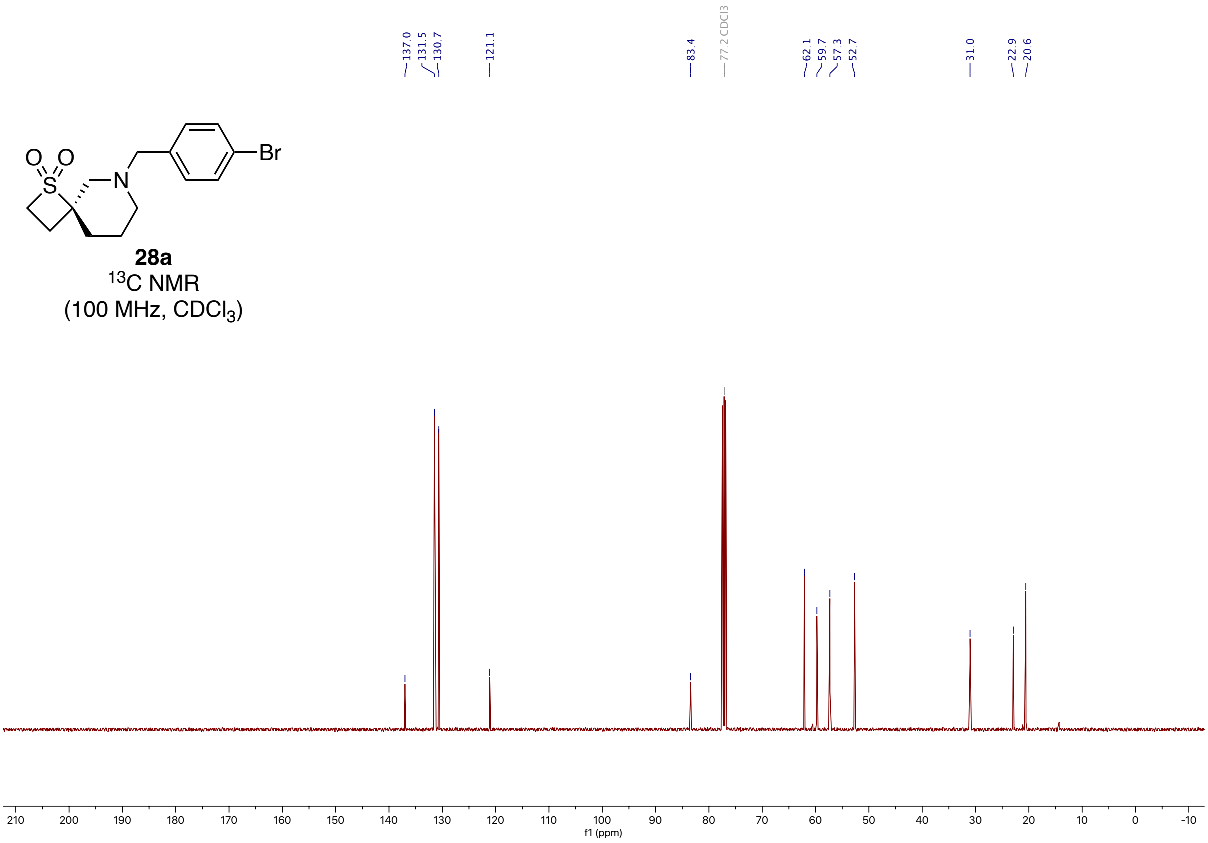

28a
^{13}C NMR
(100 MHz, $CDCl_3$)

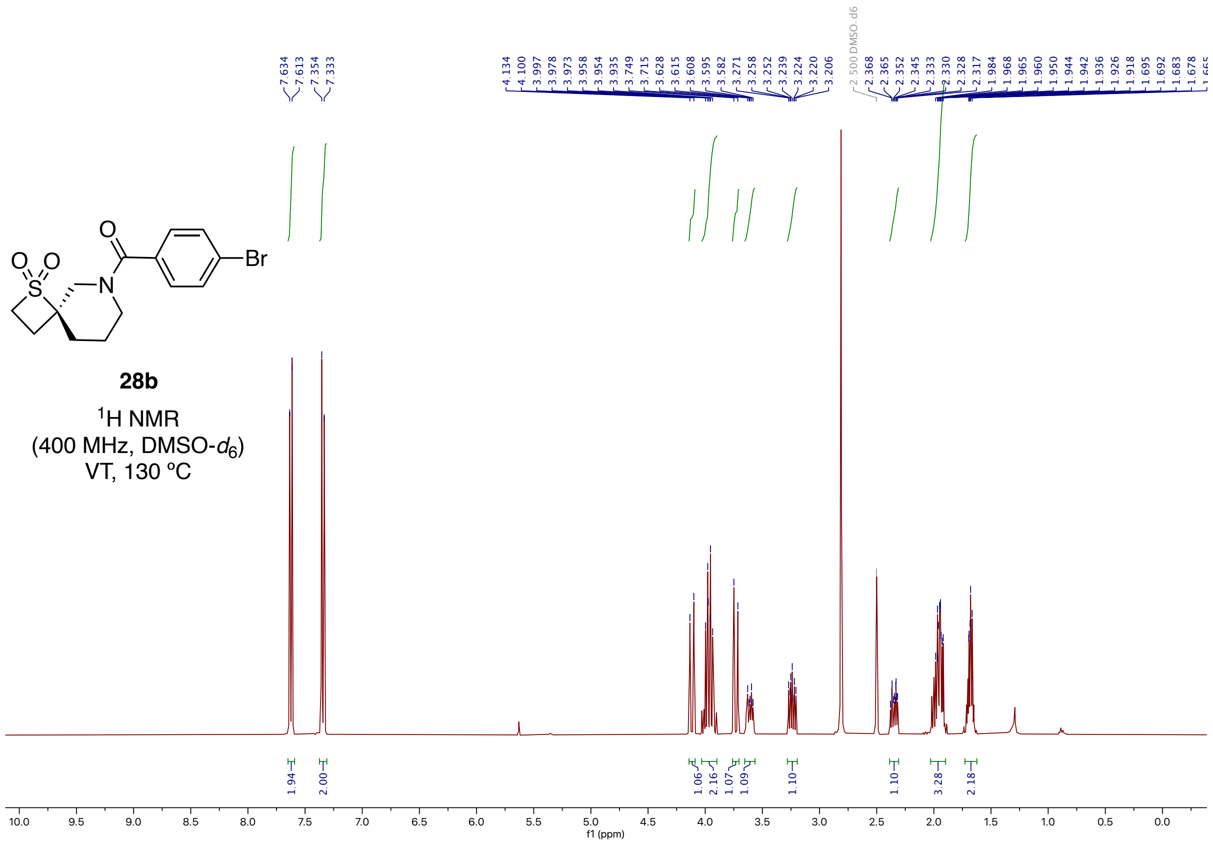
28b
^{1}H NMR
(400 MHz, DMSO-d_6)
VT, 130 °C
f1 (ppm)

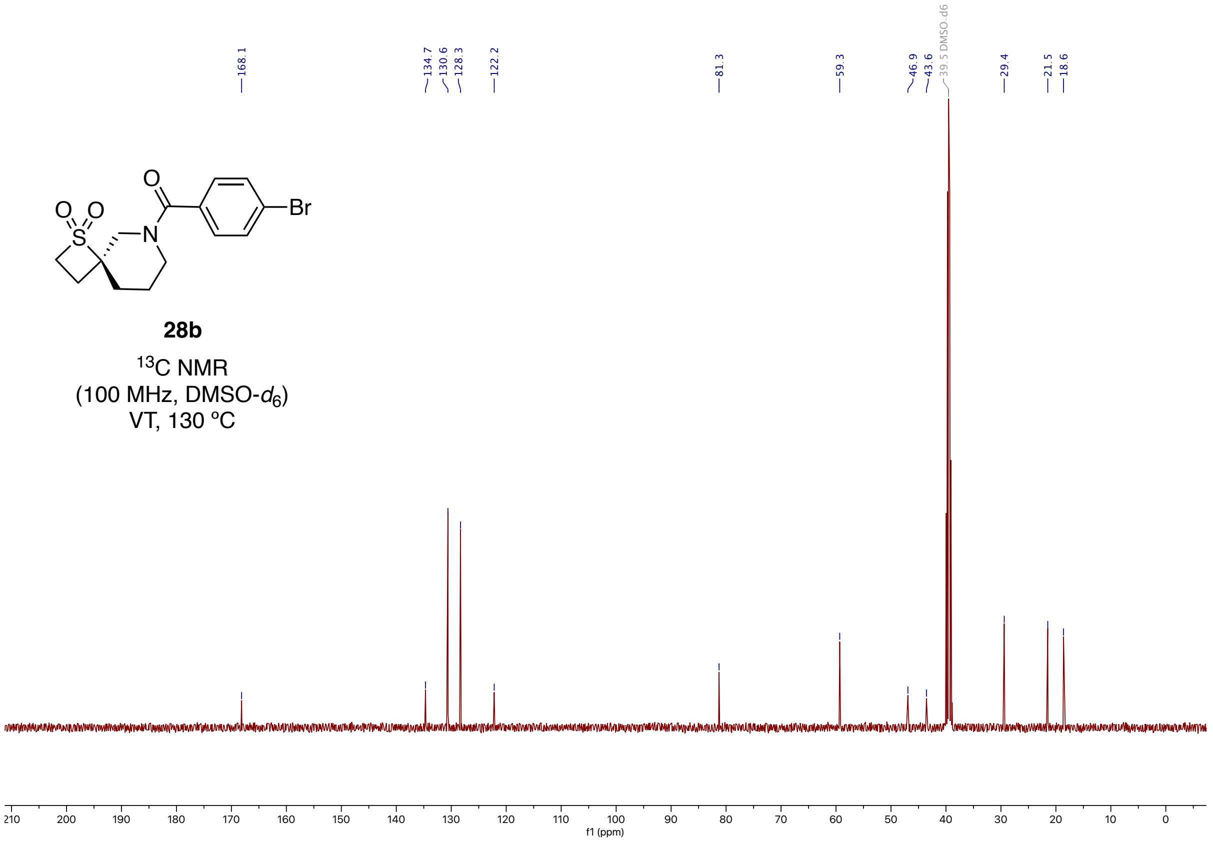
28b
^{13}C NMR
(100 MHz, DMSO-d_6)
VT, 130 °C
168.1
134.7
130.6
128.3
122.2
81.3
59.3
46.9
43.6
39.5 DMSO d6
29.4
21.5
18.6
f1 (ppm)

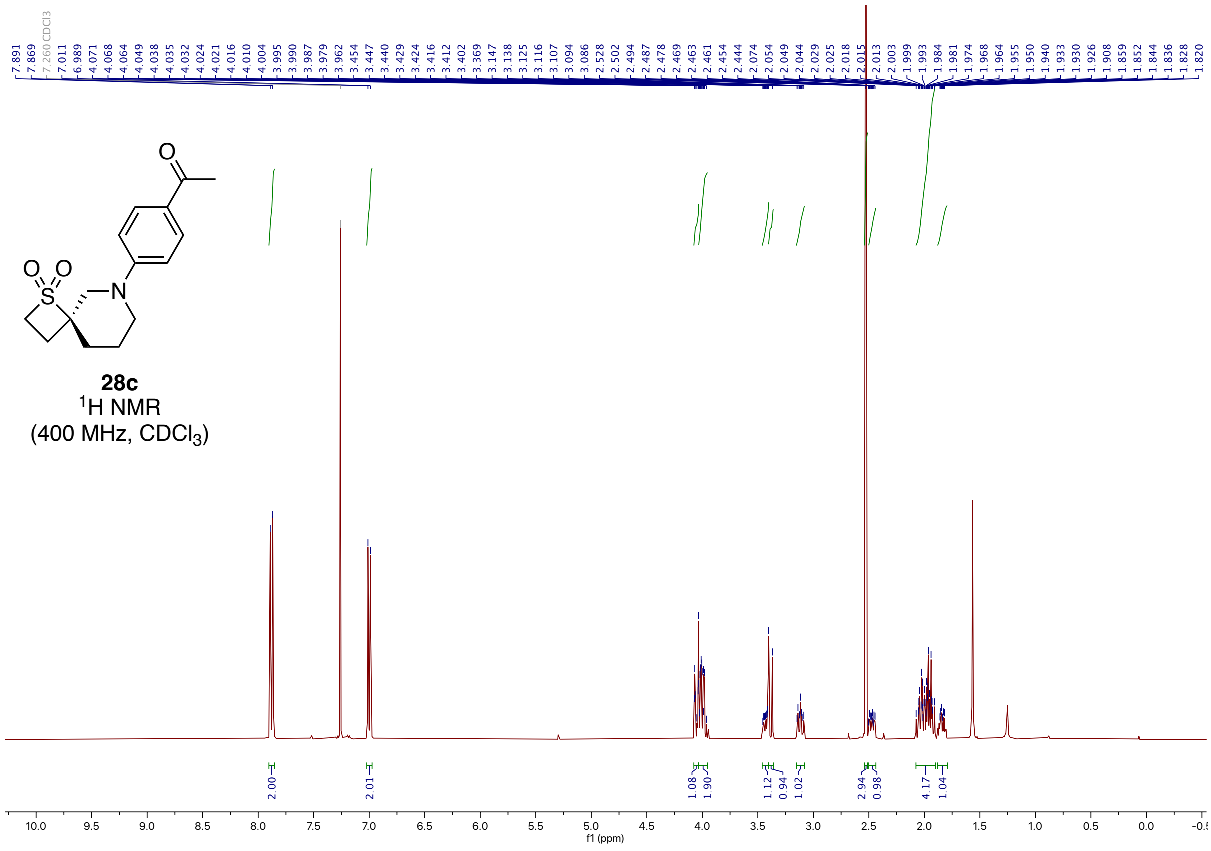

28c
^{1}H NMR
(400 MHz, $CDCl_3$)

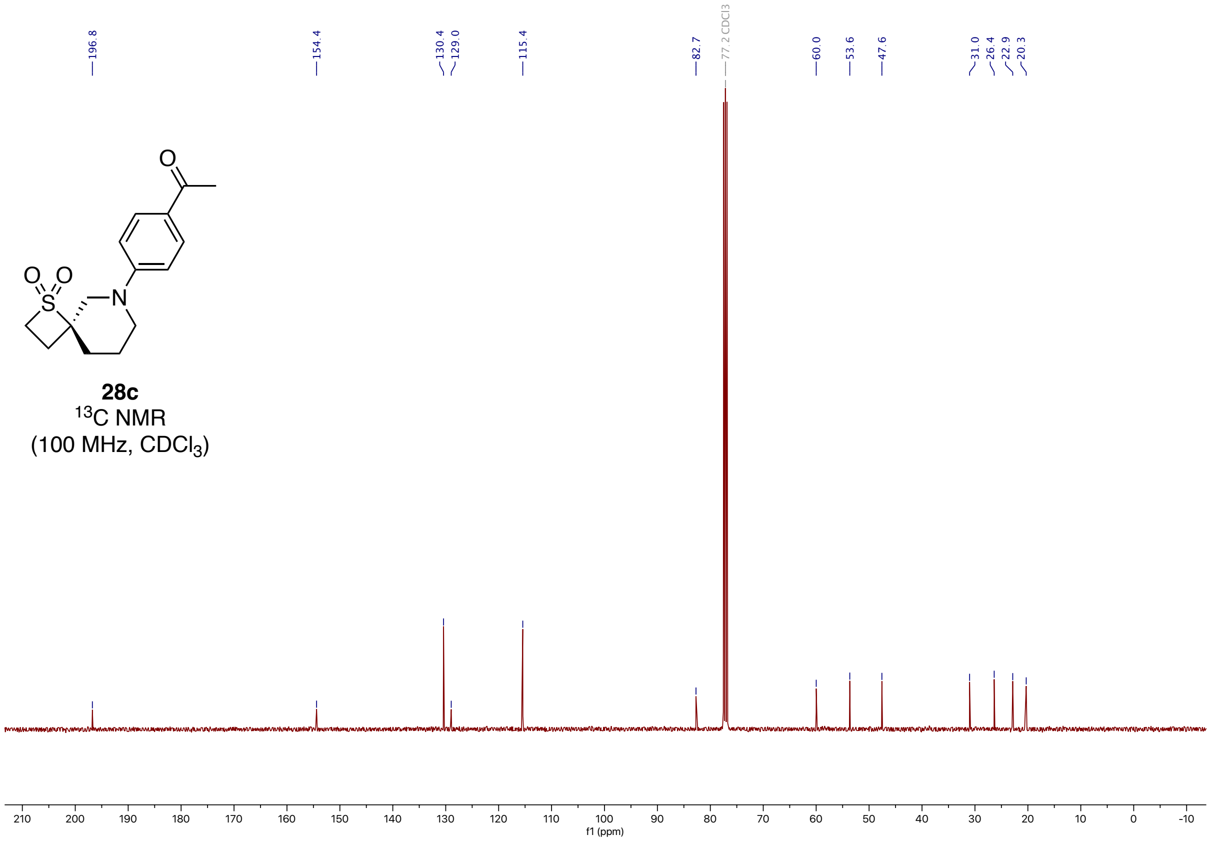

28c
^{13}C NMR
(100 MHz, $CDCl_3$)

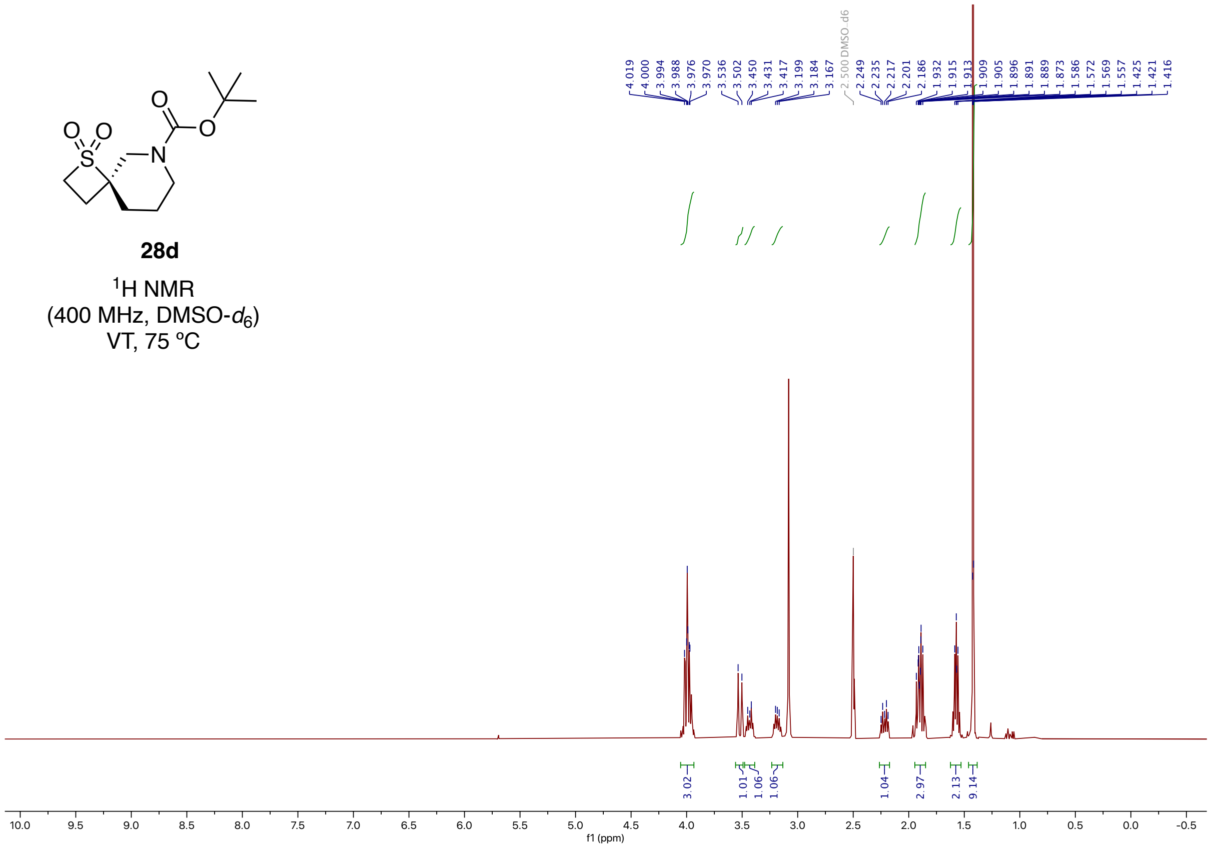
28d
^{1}H NMR
(400 MHz, DMSO-d_6)
VT, 75 °C

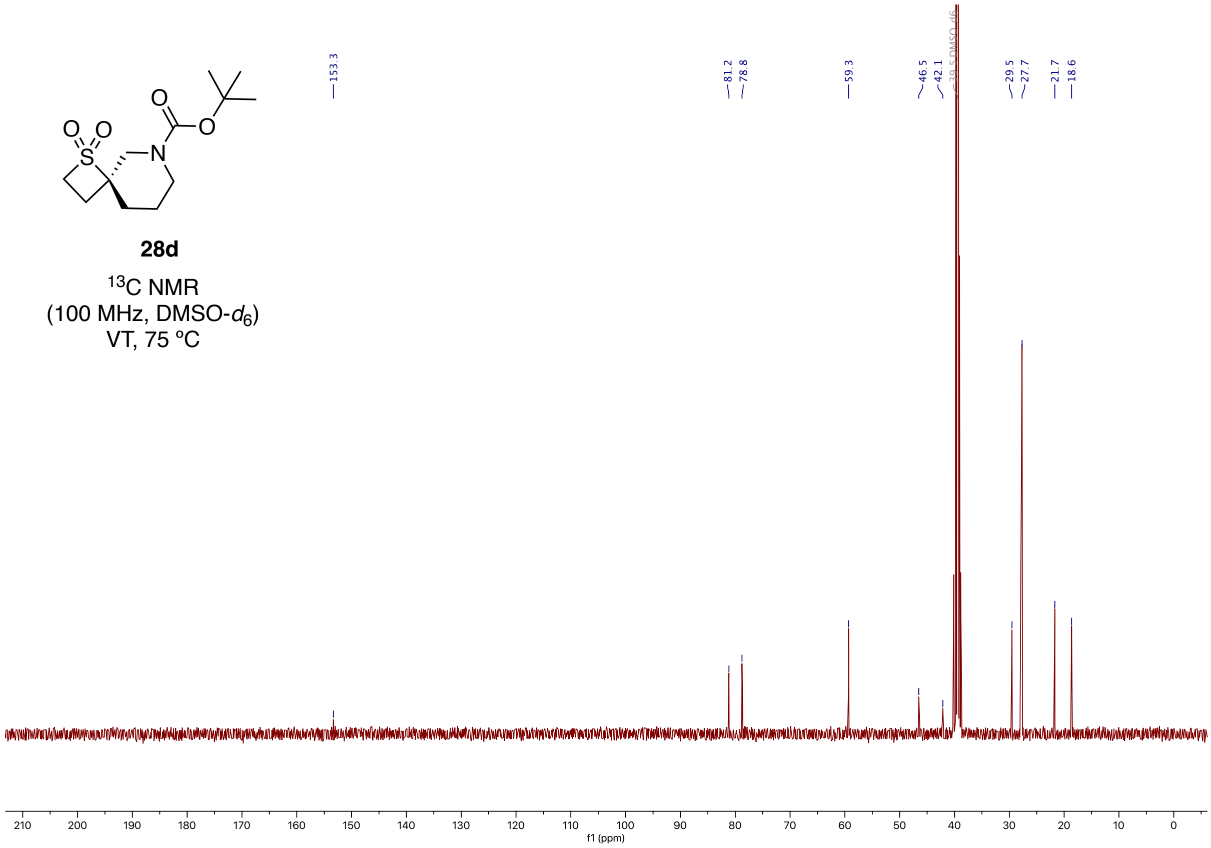
28d
^{13}C NMR
(100 MHz, DMSO-d_6)
VT, 75 °C

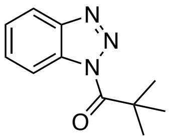

41
^{1}H NMR
(400 MHz, $CDCl_3$)

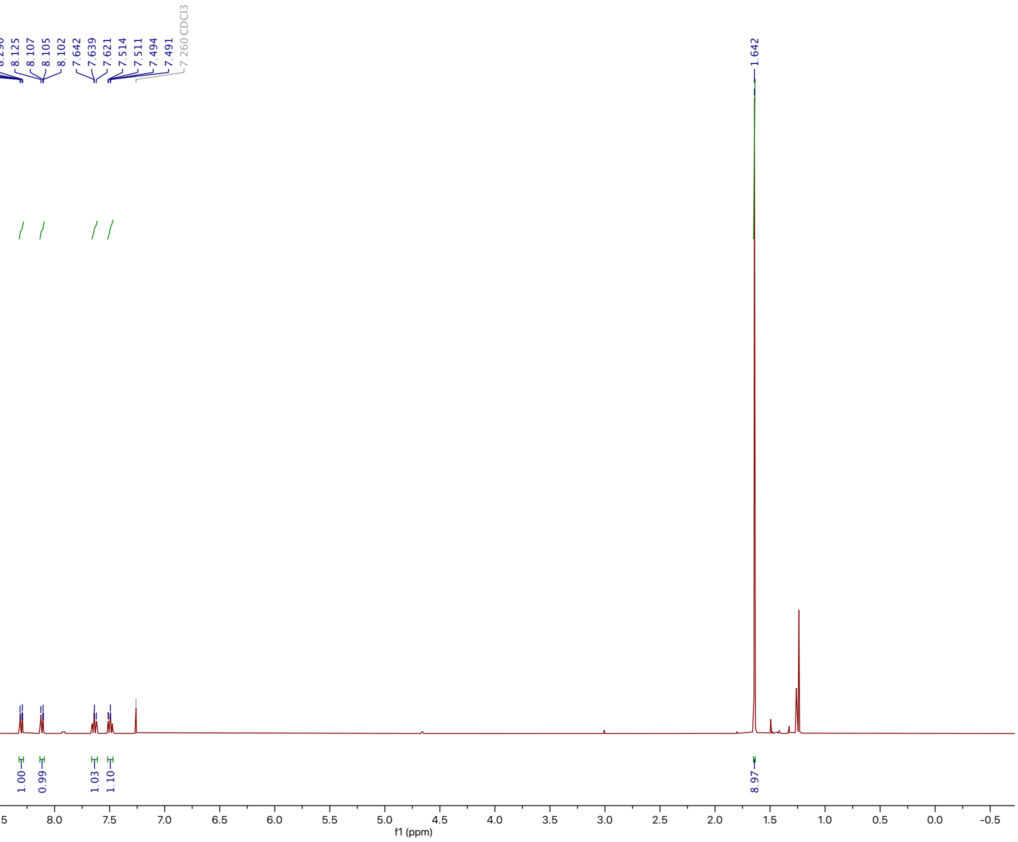

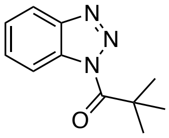

41
^{13}C NMR
(100 MHz, $CDCl_3$)

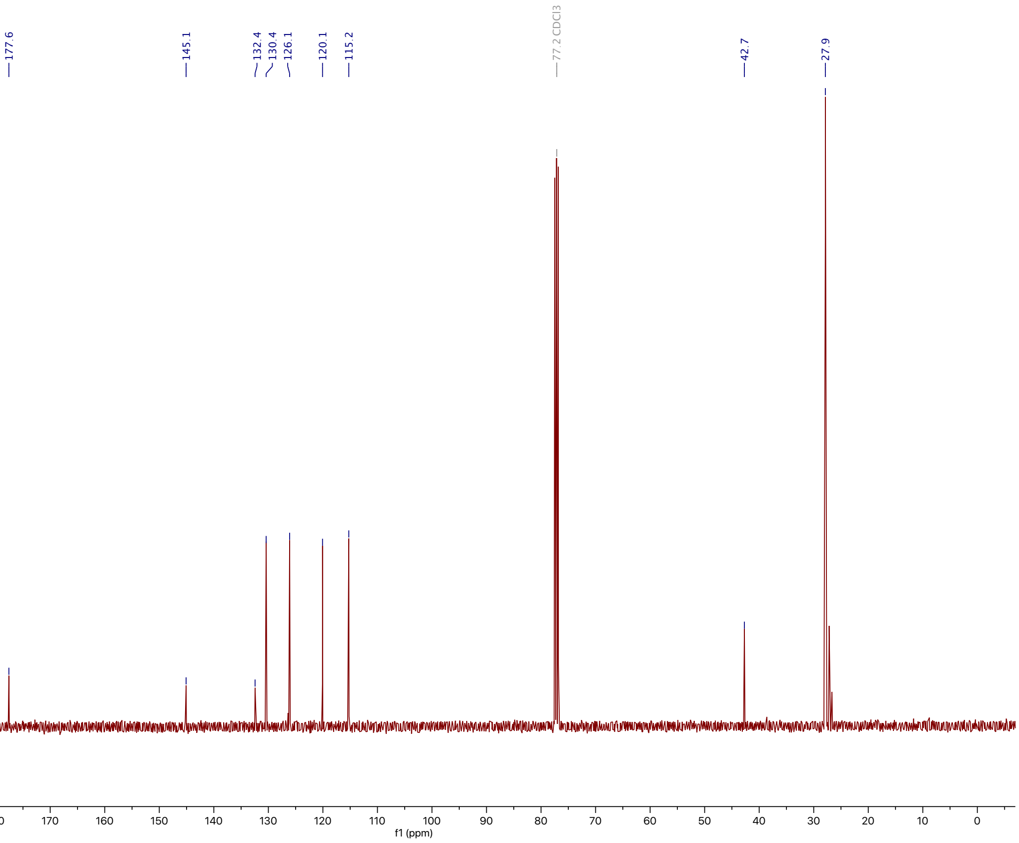

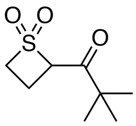

42
^{1}H NMR
(400 MHz, $CDCl_3$)

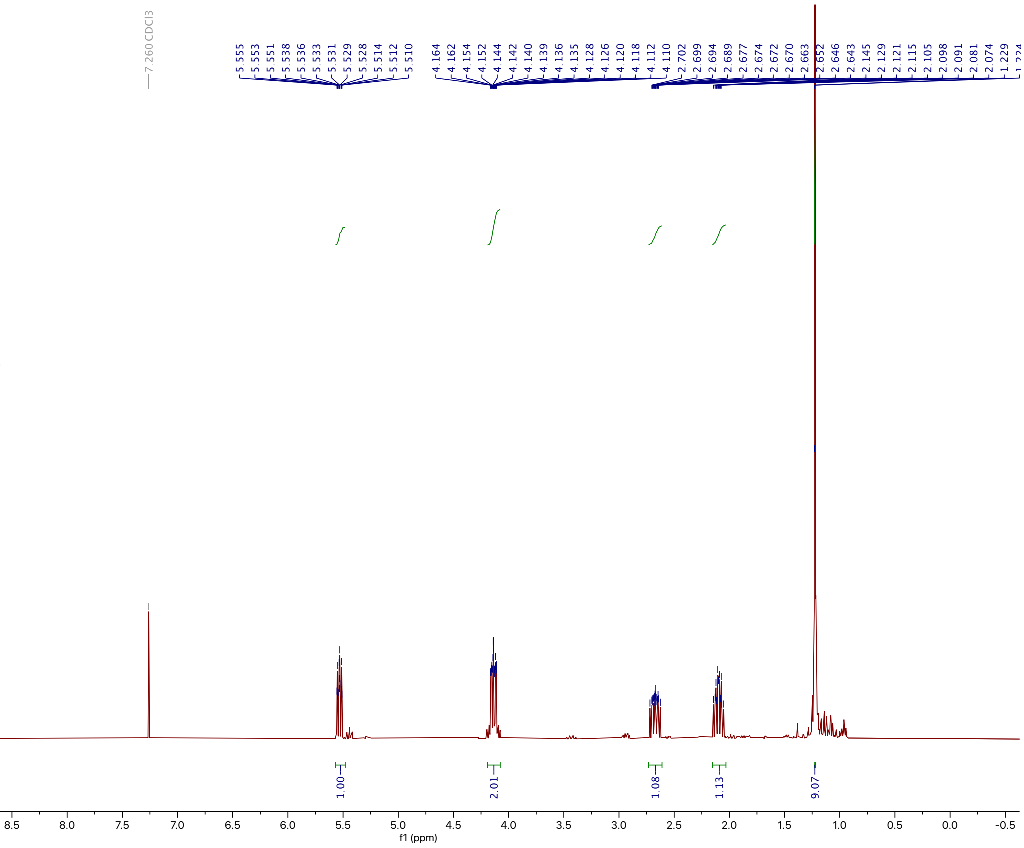

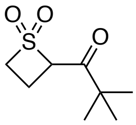

42
^{13}C NMR
(100 MHz, $CDCl_3$)

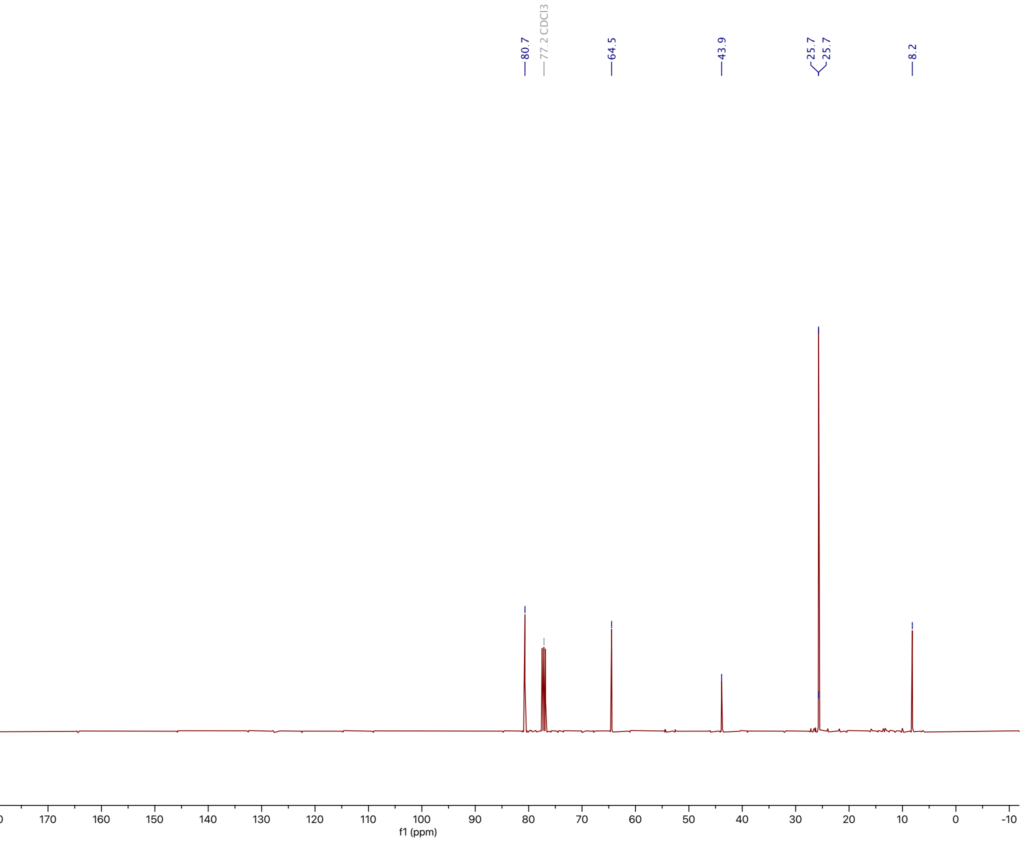

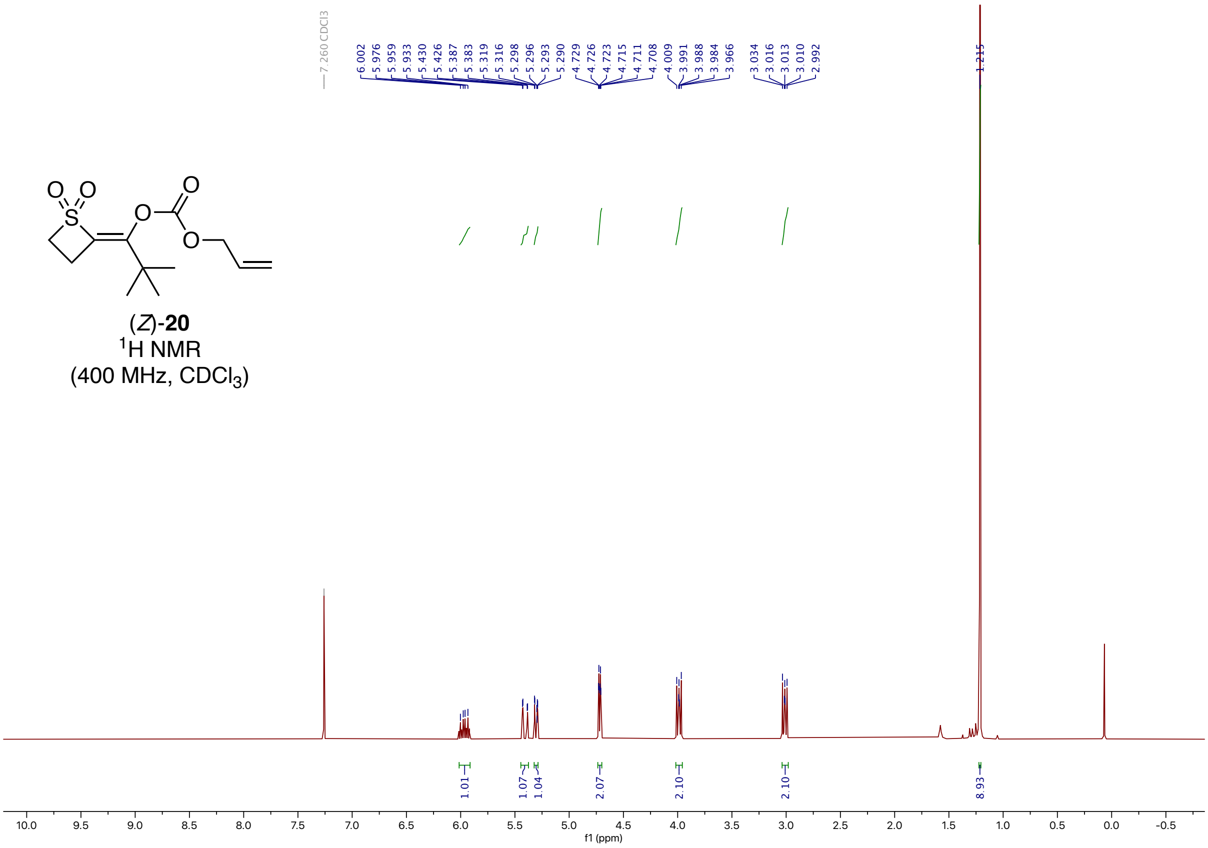
(Z)-20
1H NMR
(400 MHz, CDCl3)
7.260 CDCl3
6.002
5.976
5.959
5.933
5.430
5.426
5.387
5.383
5.319
5.316
5.298
5.296
5.293
5.290
4.729
4.726
4.723
4.715
4.711
4.708
4.009
3.991
3.988
3.984
3.966
3.034
3.016
3.013
3.010
2.992
1.215
1.01
1.07
1.04
2.07
2.10
2.10
8.93
f1 (ppm)

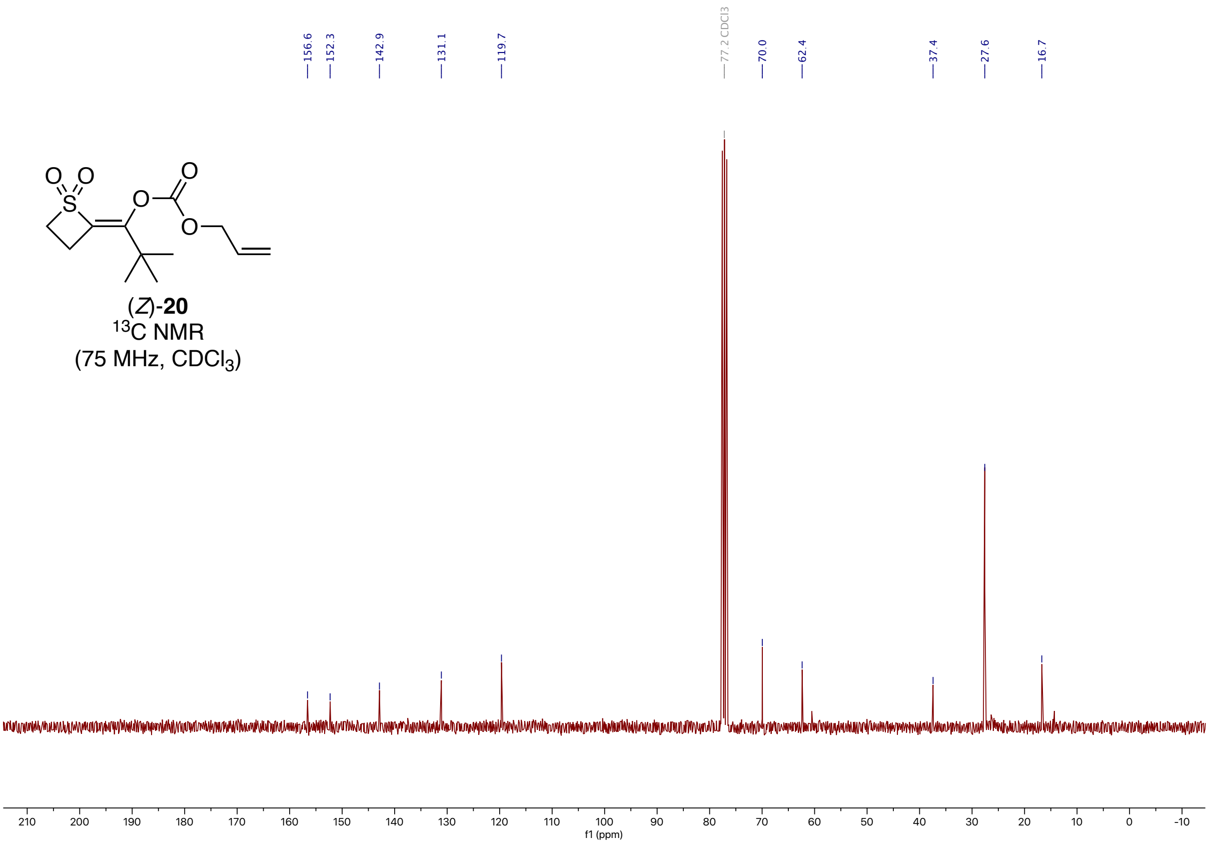
(Z)-20
13C NMR
(75 MHz, CDCl3)
156.6
152.3
142.9
131.1
119.7
77.2 CDCl3
70.0
62.4
37.4
27.6
16.7
f1 (ppm)

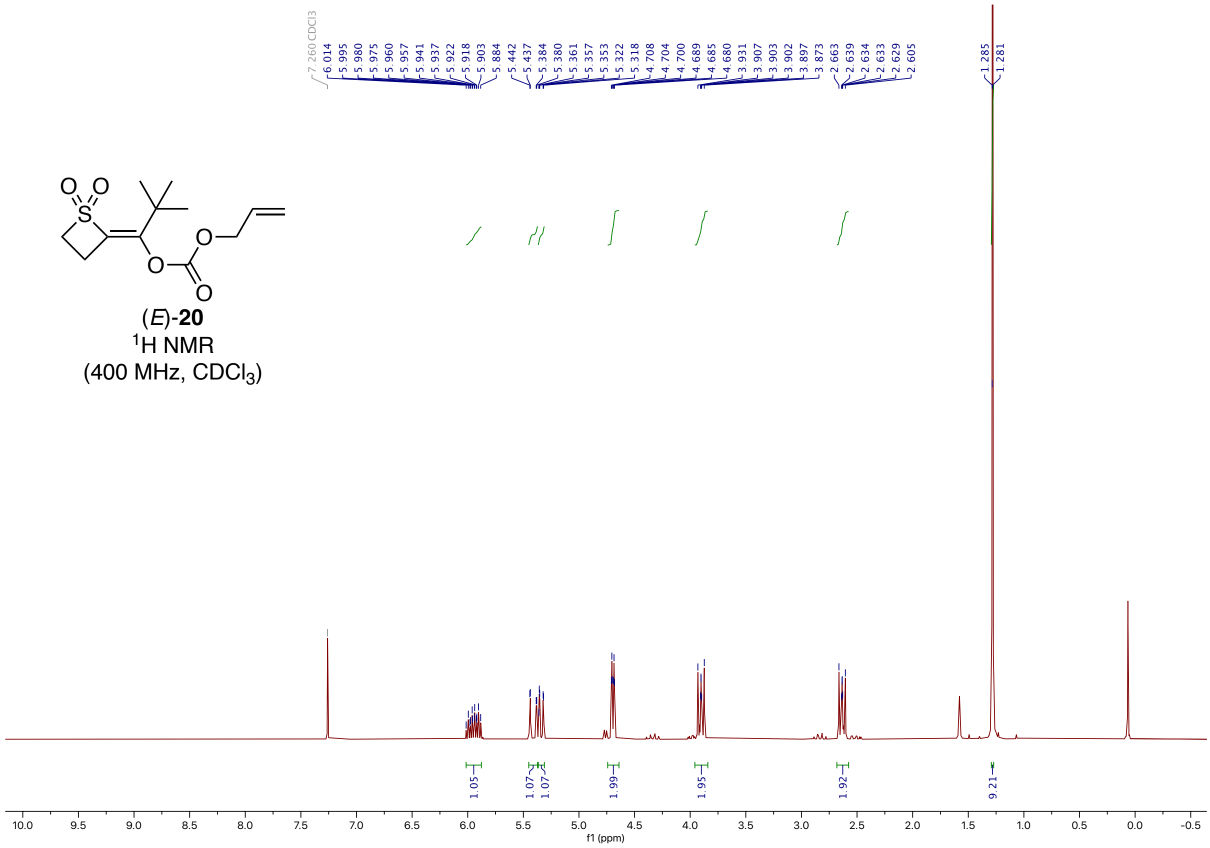

(*E*)-**20**
^{1}H NMR
(400 MHz, $CDCl_3$)

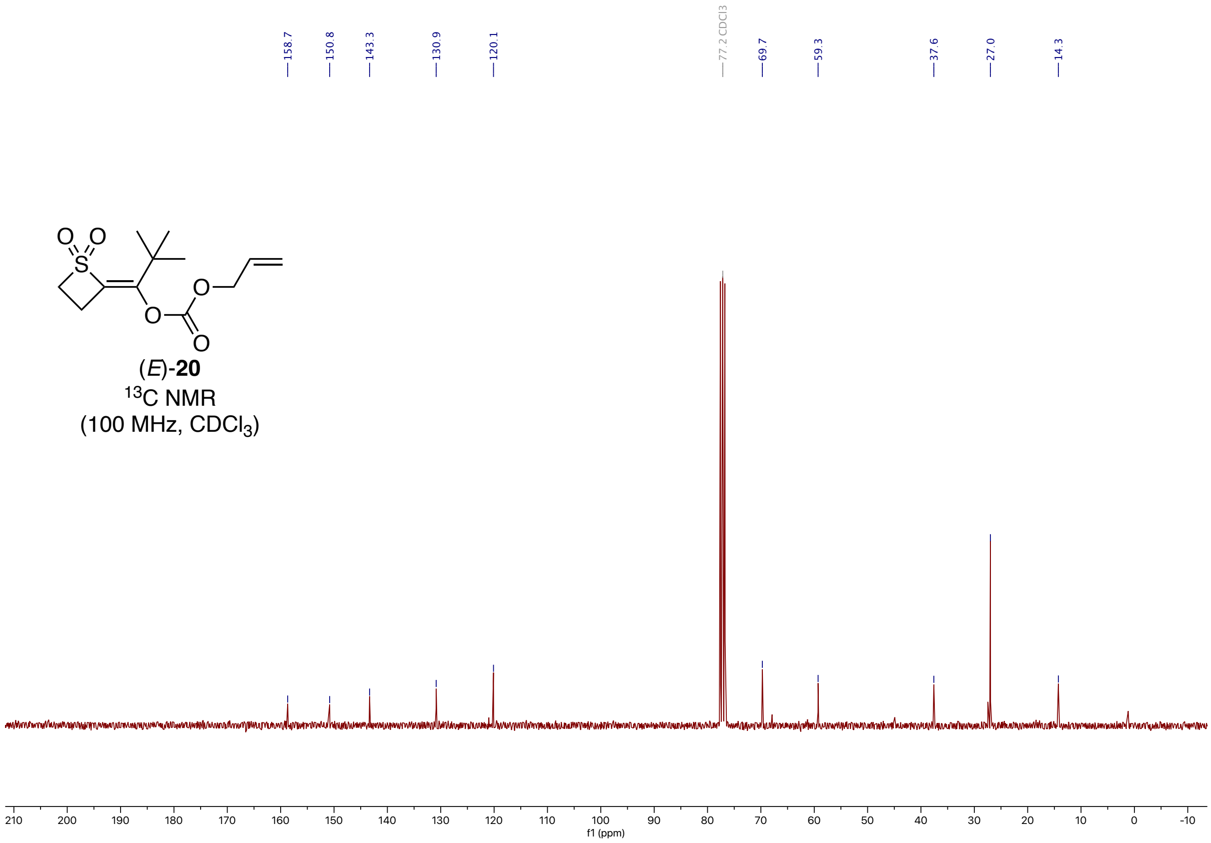

(*E*)-**20**
^{13}C NMR
(100 MHz, $CDCl_3$)

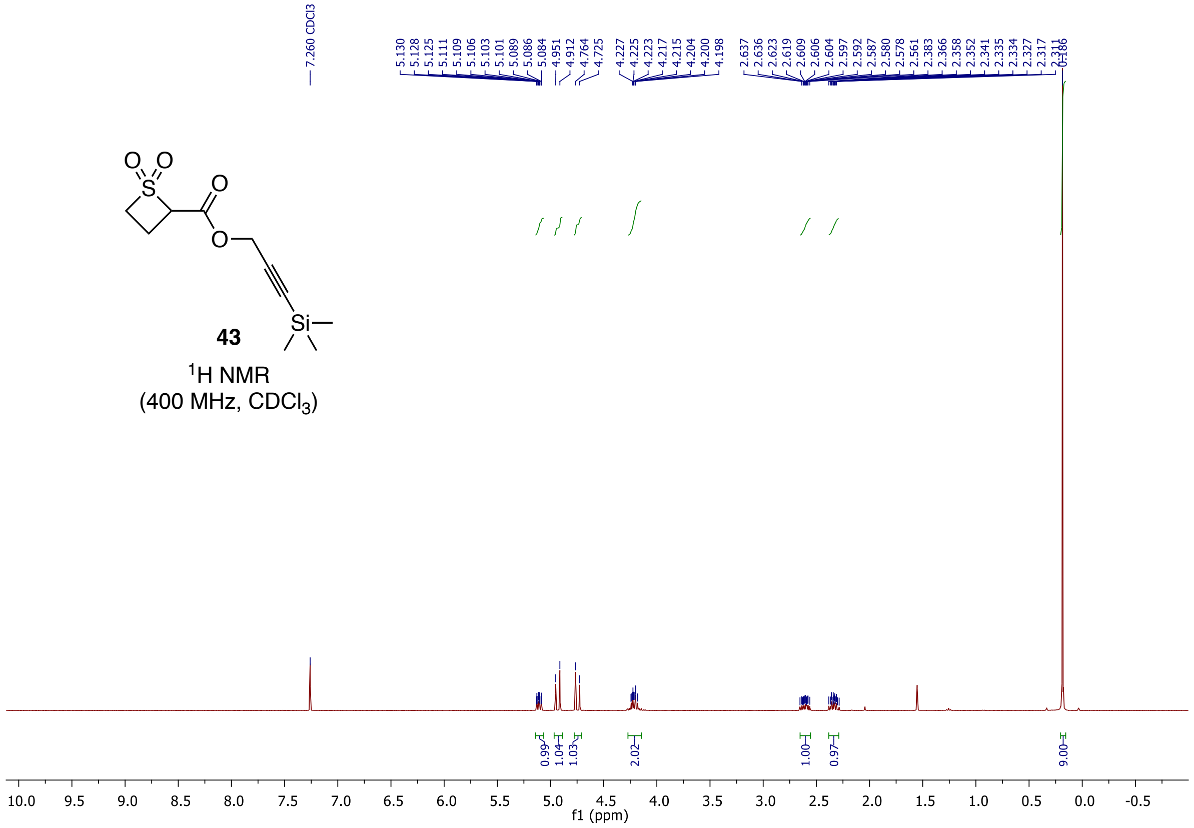

43
^{1}H NMR
(400 MHz, CDCl$_3$)

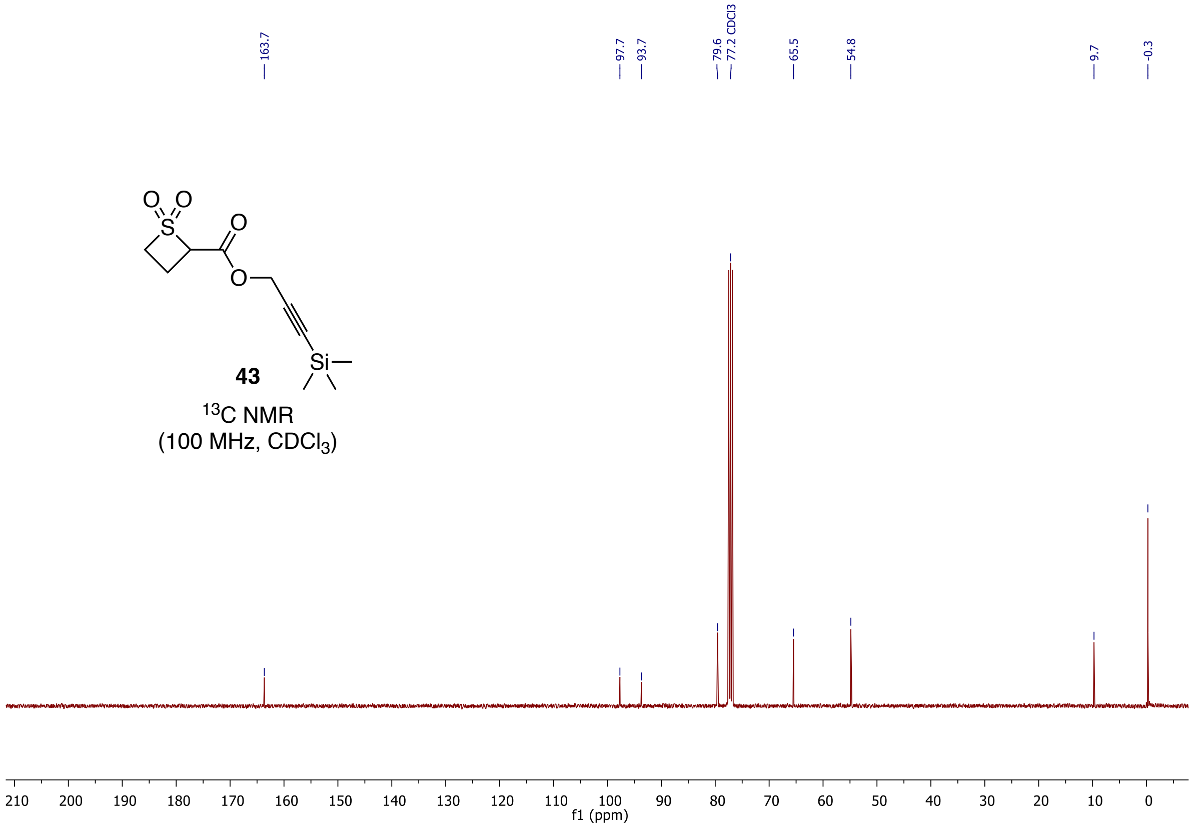

43
^{13}C NMR
(100 MHz, CDCl$_3$)

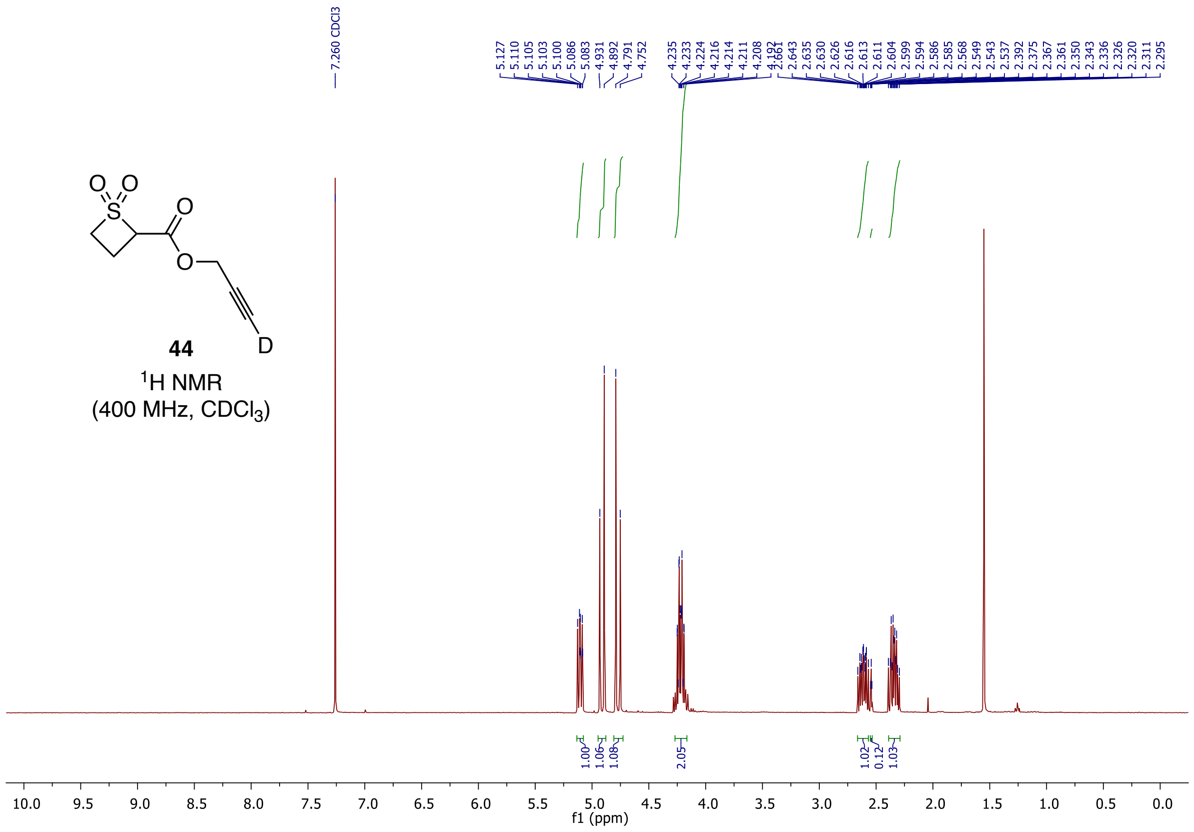
7.260 CDCl3
5.127
5.110
5.105
5.103
5.100
5.086
5.083
4.931
4.892
4.791
4.752
4.235
4.233
4.224
4.216
4.214
4.211
4.208
4.192
2.661
2.643
2.635
2.630
2.626
2.616
2.613
2.611
2.604
2.599
2.594
2.586
2.585
2.568
2.549
2.543
2.537
2.392
2.375
2.367
2.361
2.350
2.343
2.336
2.326
2.320
2.311
2.295
44
D
1H NMR
(400 MHz, CDCl3)
1.00
1.06
1.08
2.05
1.02
0.12
1.03
f1 (ppm)

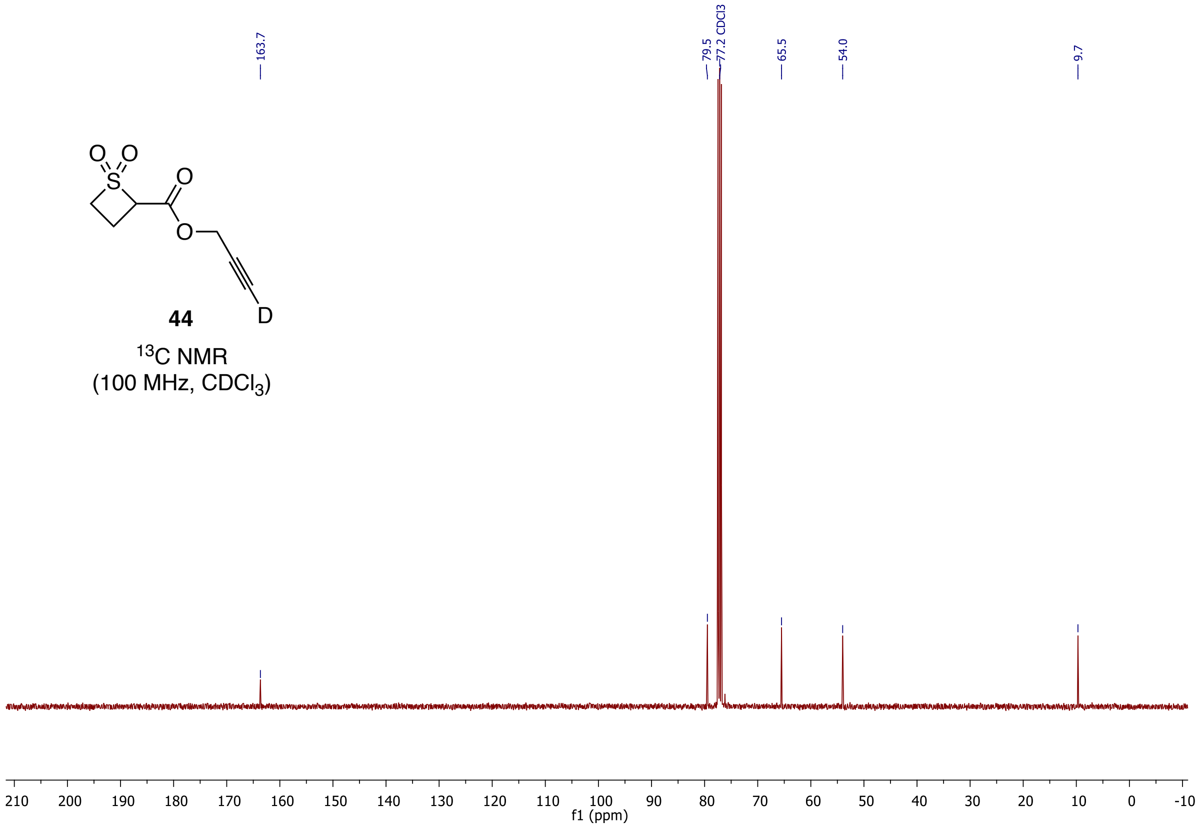
163.7
79.5
77.2 CDCl3
65.5
54.0
9.7
44
D
13C NMR
(100 MHz, CDCl3)
f1 (ppm)

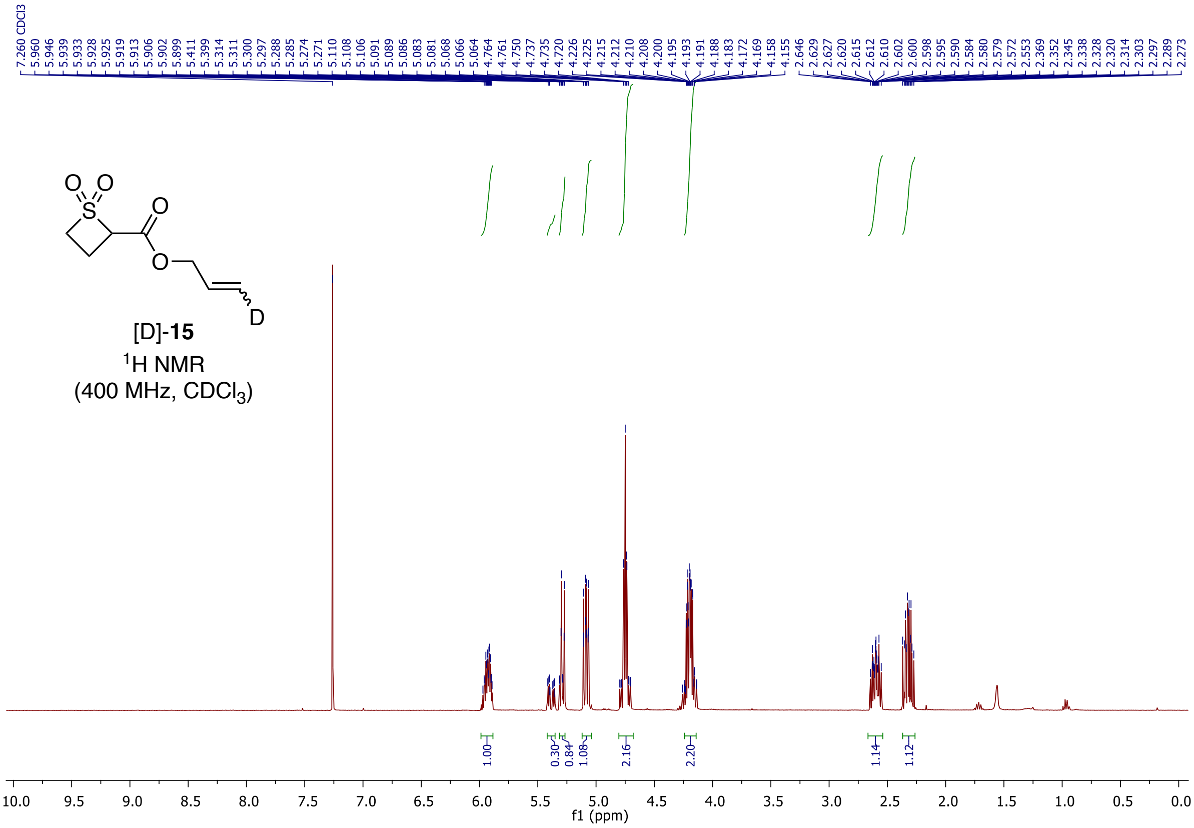
[D]-15
1H NMR
(400 MHz, CDCl3)
f1 (ppm)

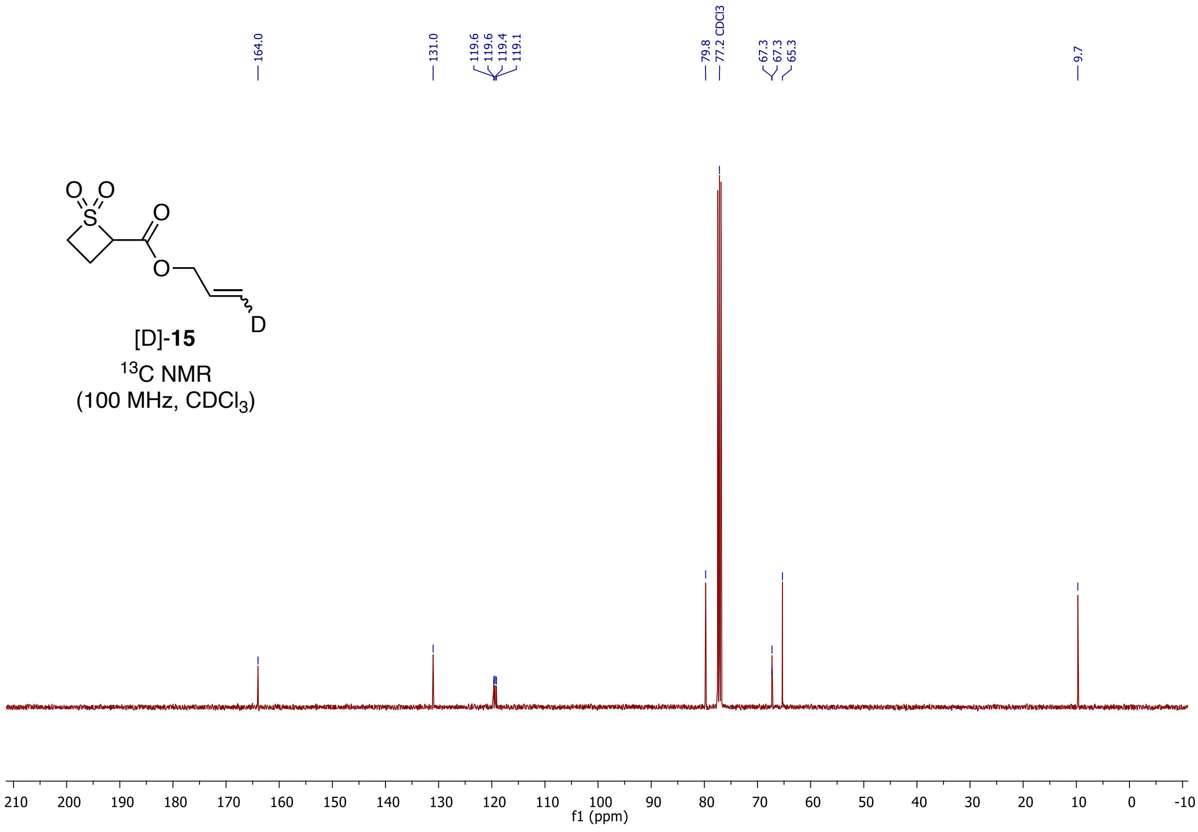
[D]-15
13C NMR
(100 MHz, CDCl3)
f1 (ppm)

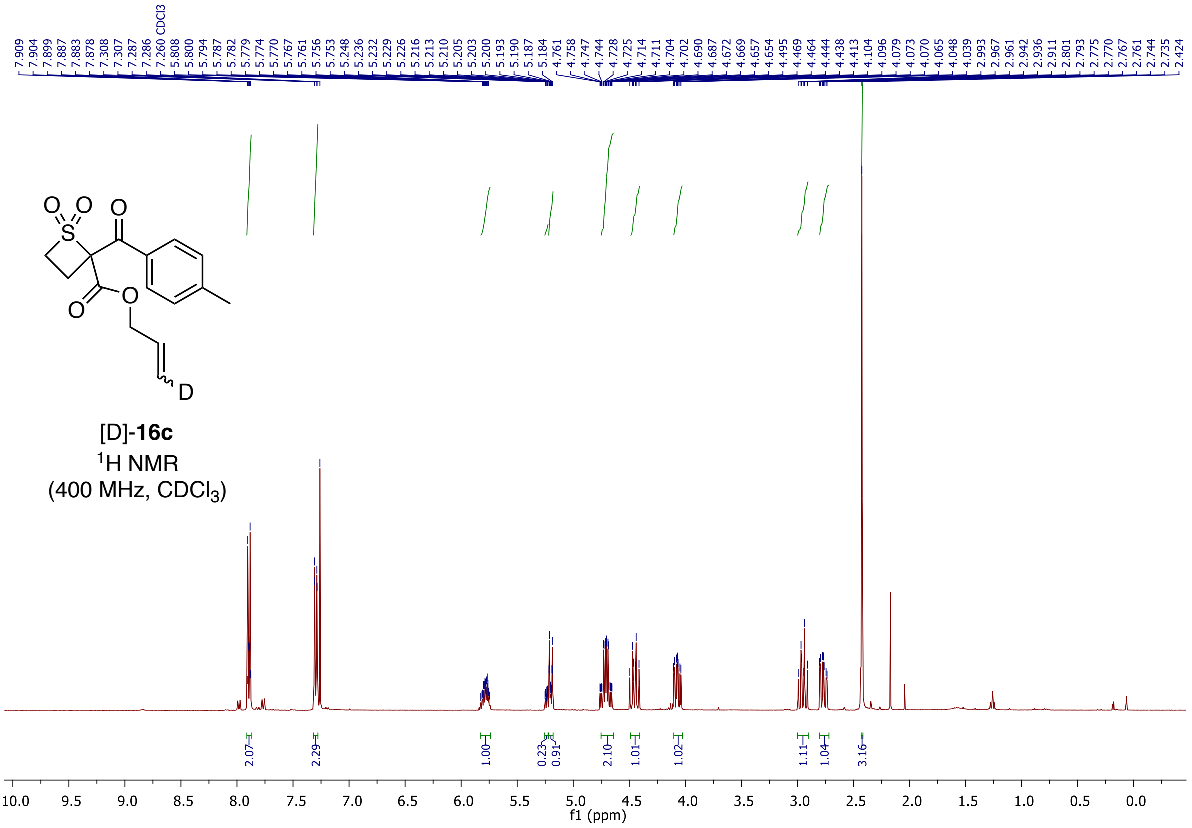
7.909
7.904
7.899
7.887
7.883
7.878
7.308
7.307
7.287
7.286
7.260 CDCl3
5.808
5.800
5.794
5.787
5.782
5.779
5.774
5.770
5.767
5.761
5.756
5.753
5.248
5.236
5.232
5.229
5.226
5.216
5.213
5.210
5.205
5.203
5.200
5.193
5.190
5.187
5.184
4.761
4.758
4.747
4.744
4.728
4.725
4.714
4.711
4.704
4.702
4.690
4.687
4.672
4.669
4.657
4.654
4.495
4.469
4.464
4.444
4.438
4.413
4.104
4.096
4.079
4.073
4.070
4.065
4.048
4.039
2.993
2.967
2.961
2.942
2.936
2.911
2.801
2.793
2.775
2.770
2.767
2.761
2.744
2.735
2.424
D
[D]-16c
1H NMR
(400 MHz, CDCl3)
2.07
2.29
1.00
0.23
0.91
2.10
1.01
1.02
1.11
1.04
3.16
10.0
9.5
9.0
8.5
8.0
7.5
7.0
6.5
6.0
5.5
5.0
4.5
4.0
3.5
3.0
2.5
2.0
1.5
1.0
0.5
0.0
f1 (ppm)

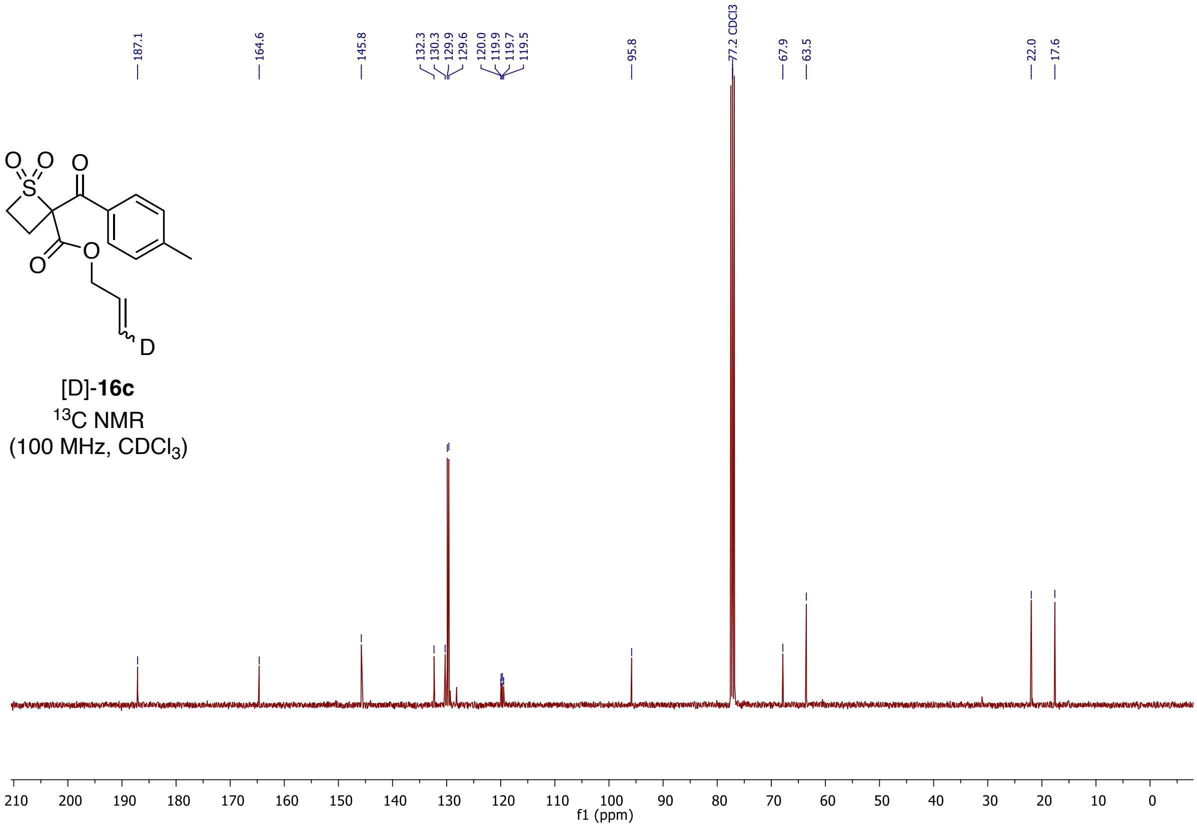
187.1
164.6
145.8
132.3
130.3
129.9
129.6
120.0
119.9
119.7
119.5
95.8
77.2 CDCl3
67.9
63.5
22.0
17.6
D
[D]-16c
13C NMR
(100 MHz, CDCl3)
210
200
190
180
170
160
150
140
130
120
110
100
90
80
70
60
50
40
30
20
10
0
f1 (ppm)

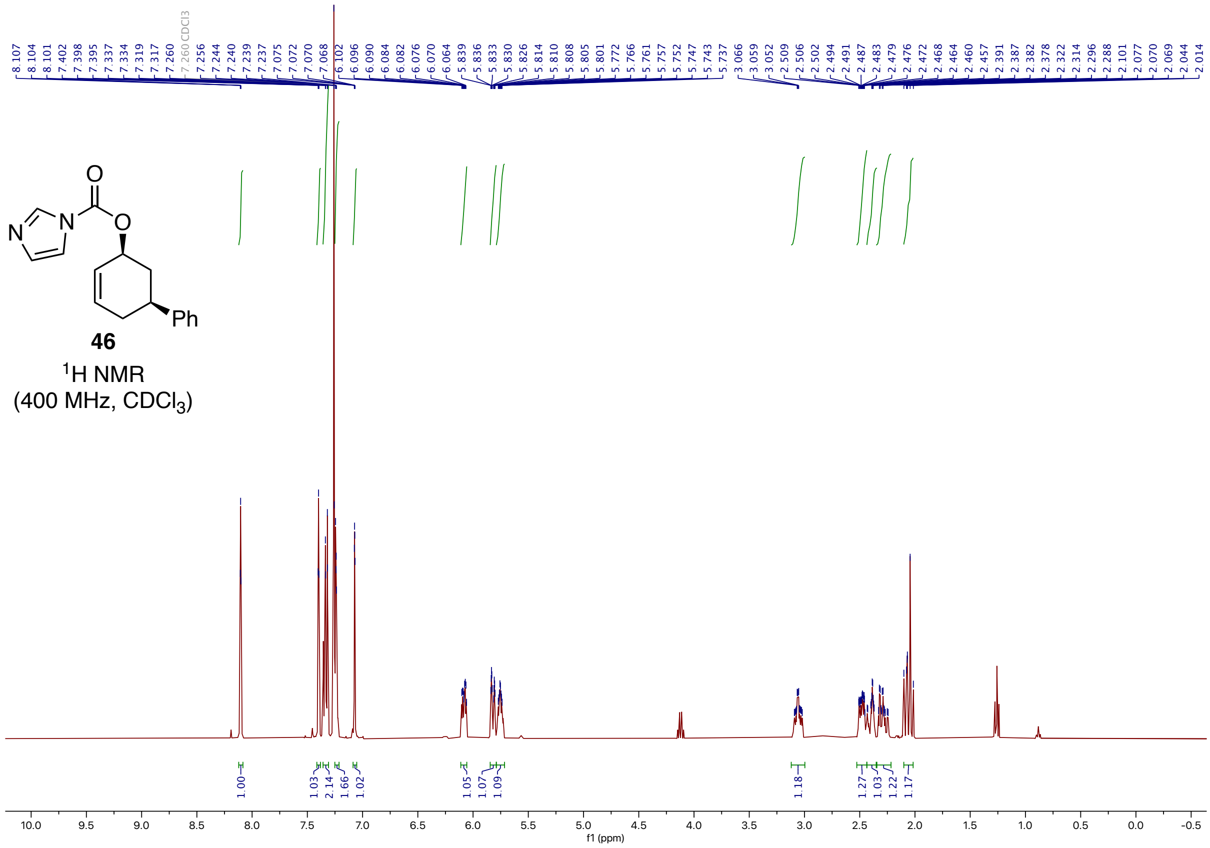
46
^{1}H NMR
(400 MHz, $CDCl_3$)
f1 (ppm)

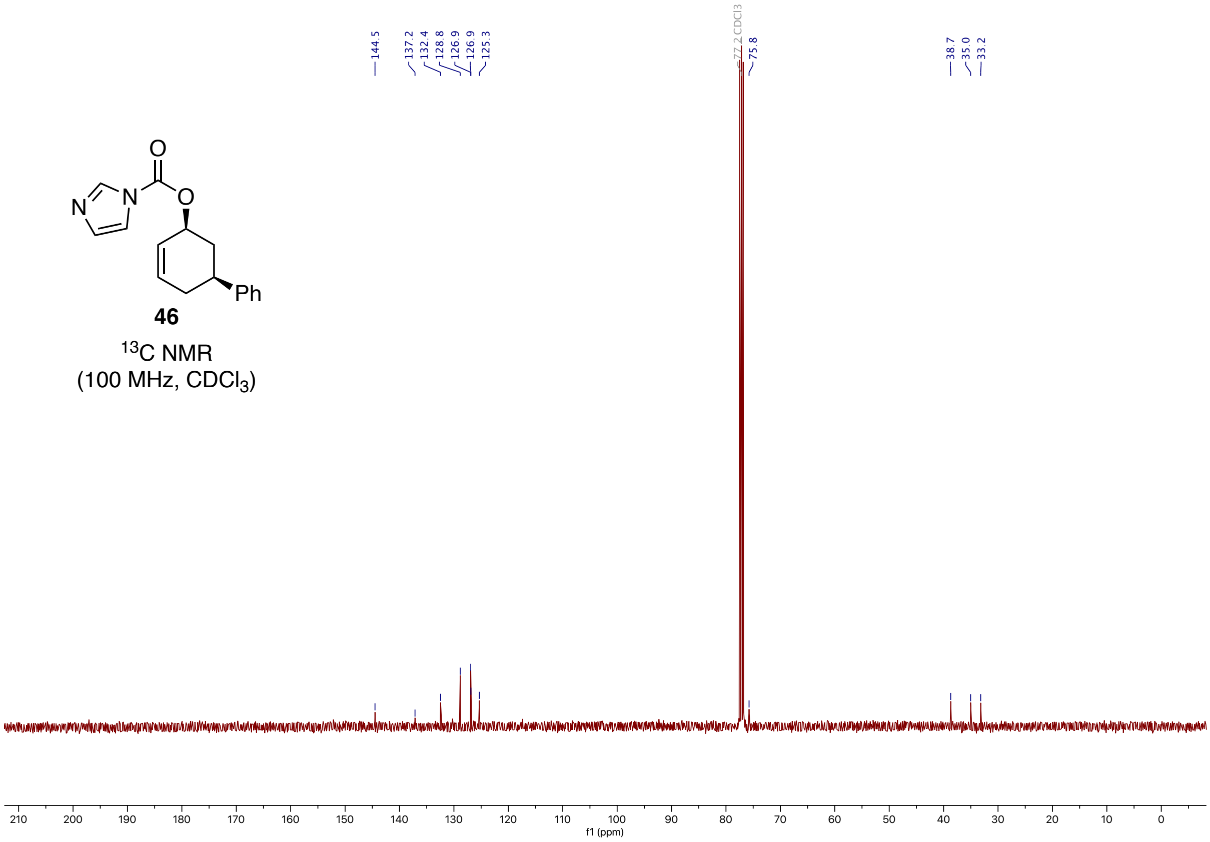
46
^{13}C NMR
(100 MHz, $CDCl_3$)
144.5
137.2
132.4
128.8
126.9
126.9
125.3
77.2 CDCl3
75.8
38.7
35.0
33.2
f1 (ppm)

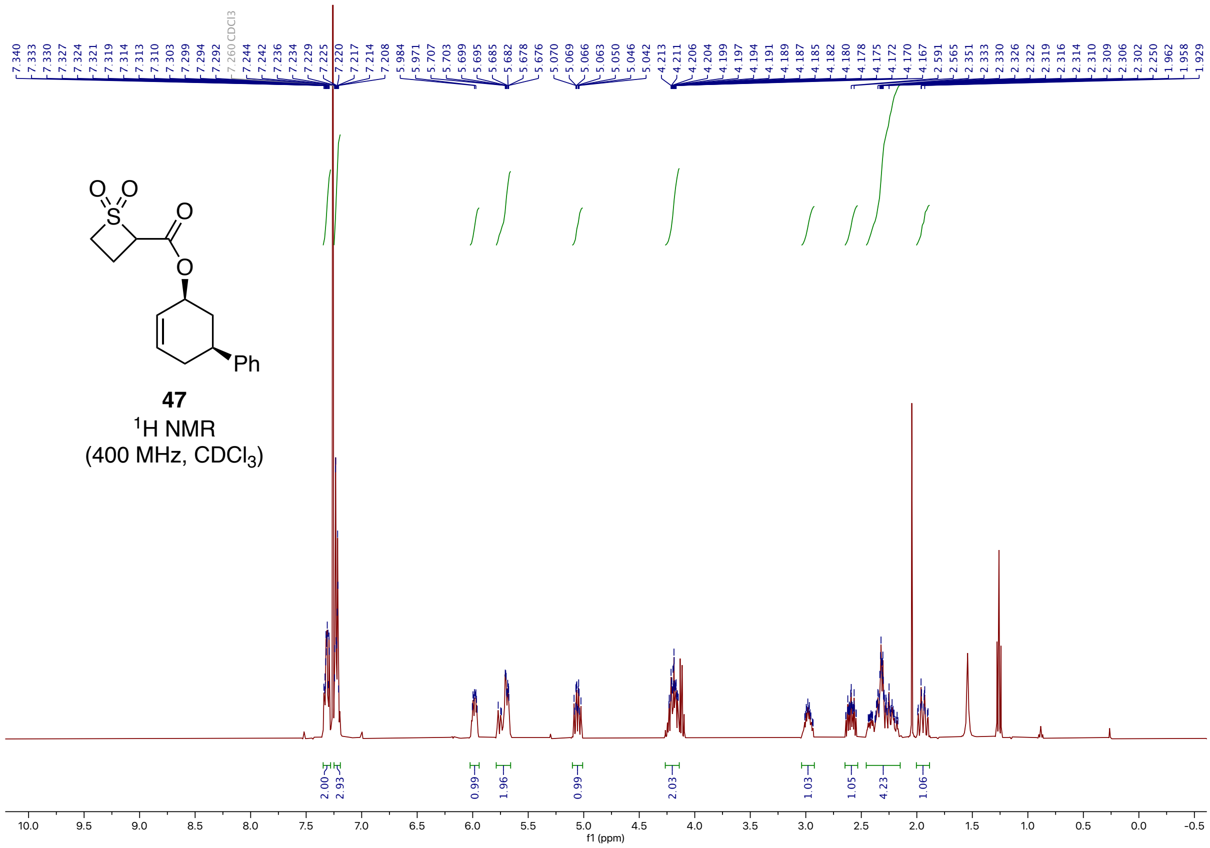
47
^{1}H NMR
(400 MHz, $CDCl_3$)
f1 (ppm)

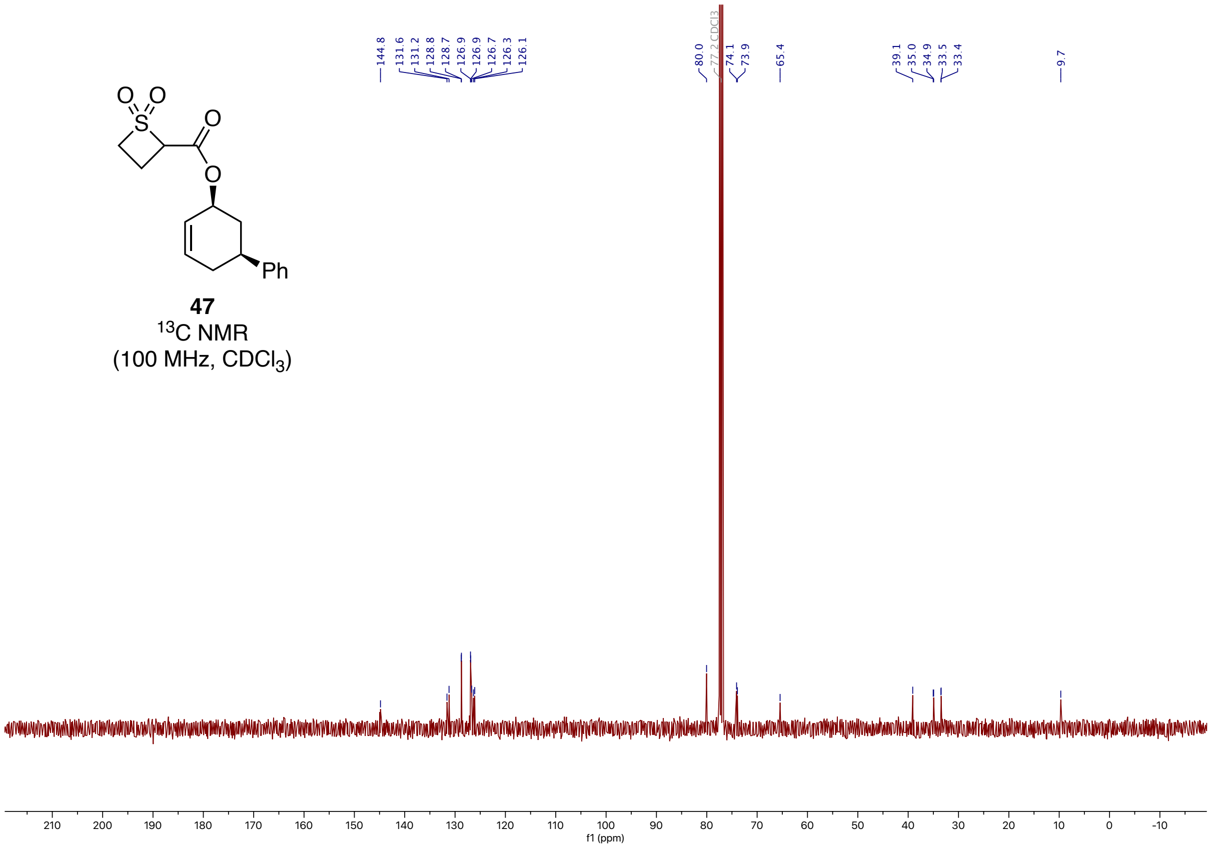
47
^{13}C NMR
(100 MHz, $CDCl_3$)
f1 (ppm)

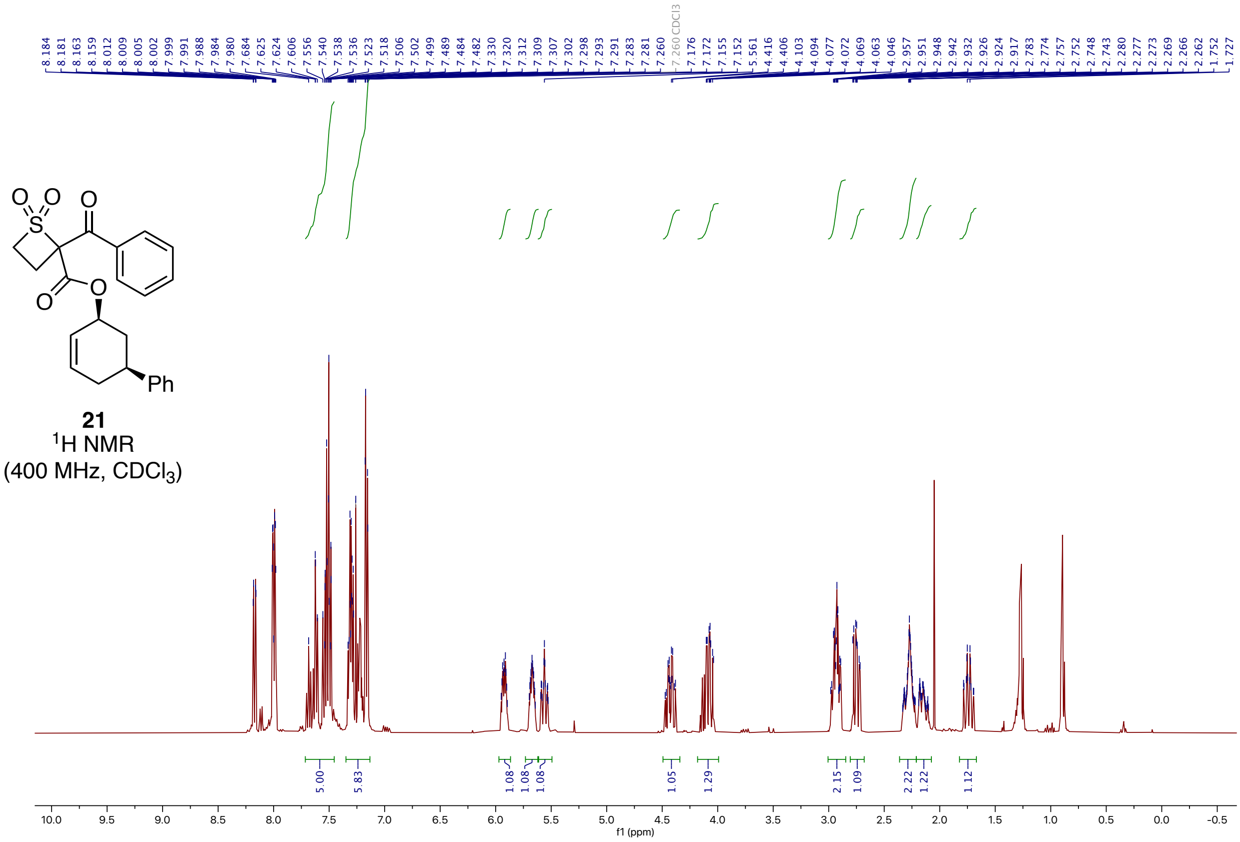
8.184 8.181 8.163 8.159 8.012 8.009 8.005 8.002 7.999 7.991 7.988 7.984 7.980 7.684 7.625 7.624 7.606 7.556 7.540 7.538 7.536 7.523 7.518 7.506 7.502 7.499 7.489 7.484 7.482 7.330 7.320 7.312 7.309 7.307 7.302 7.298 7.293 7.291 7.283 7.281 7.260 7.260 CDCl3 7.176 7.172 7.155 7.152 5.561 4.416 4.406 4.103 4.094 4.077 4.072 4.069 4.063 4.046 2.957 2.951 2.948 2.942 2.932 2.926 2.924 2.917 2.783 2.774 2.757 2.752 2.748 2.743 2.280 2.277 2.273 2.269 2.266 2.262 1.752 1.727
21
1H NMR
(400 MHz, CDCl3)
5.00 5.83 1.08 1.08 1.08 1.05 1.29 2.15 1.09 2.22 1.22 1.12
f1 (ppm)

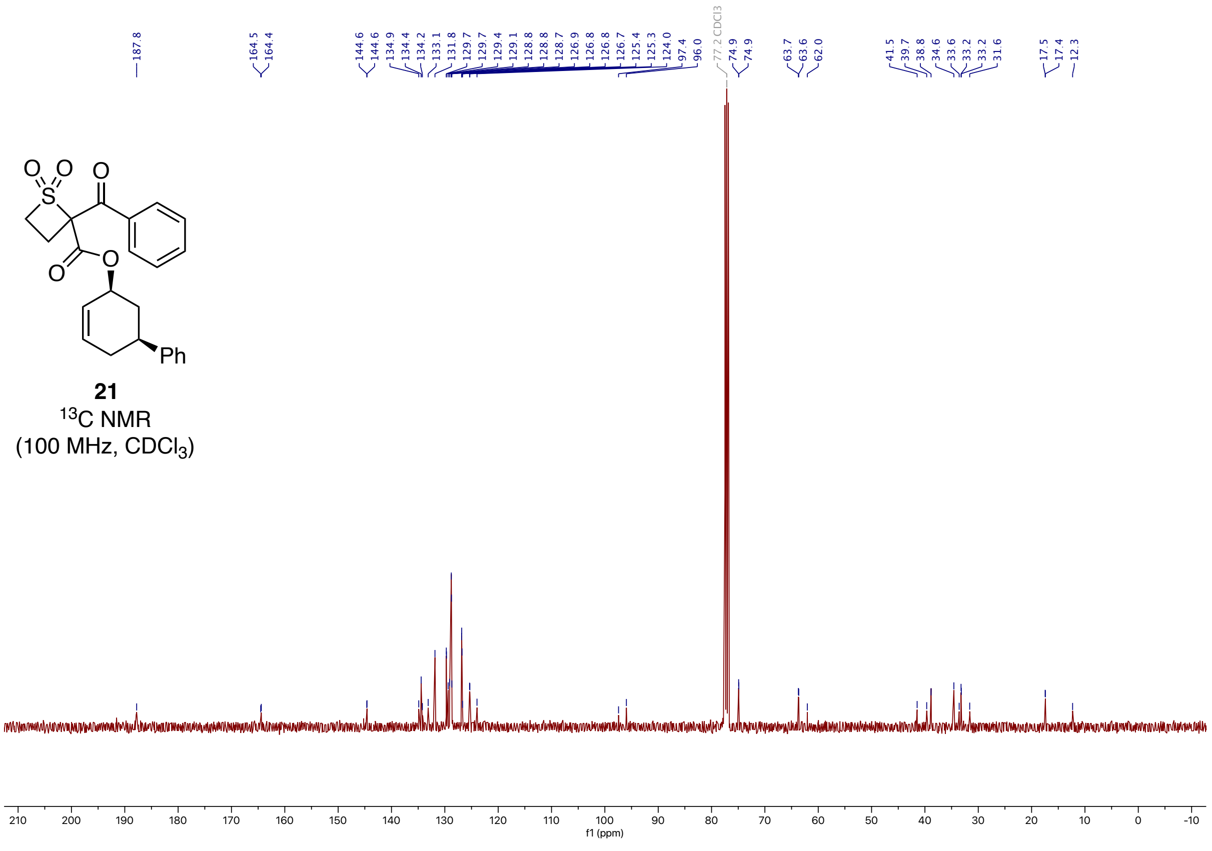
187.8 164.5 164.4 144.6 144.6 134.9 134.4 134.2 133.1 131.8 129.7 129.7 129.4 129.1 128.8 128.8 128.7 126.9 126.8 126.7 125.4 125.3 124.0 97.4 96.0 77.2 CDCl3 74.9 74.9 63.7 63.6 62.0 41.5 39.7 38.8 34.6 33.6 33.2 33.2 31.6 17.5 17.4 12.3
21
13C NMR
(100 MHz, CDCl3)
f1 (ppm)

7. HPLC Data

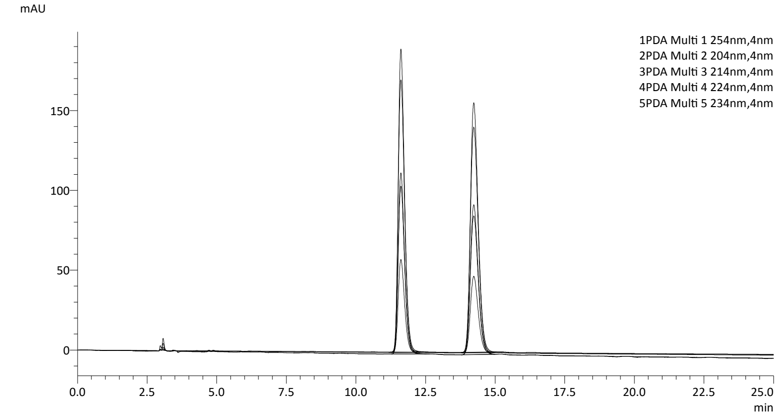

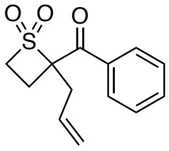

rac - **18a**

Peak Table

PDA Ch1 254nm

Name	Peak#	Ret. Time	Area	Area%	Mark
	1	11.615	3051334	49.683	
	2	14.233	3090216	50.317	
	Total		6141550	100.000	

PDA Ch2 204nm

Name	Peak#	Ret. Time	Area	Area%	Mark
	1	11.615	2780017	49.399	
	2	14.233	2847640	50.601	
	Total		5627657	100.000	

PDA Ch3 214nm

Name	Peak#	Ret. Time	Area	Area%	Mark
	1	11.615	1693068	49.562	
	2	14.233	1722996	50.438	
	Total		3416064	100.000	

PDA Ch4 224nm

Name	Peak#	Ret. Time	Area	Area%	Mark
	1	11.615	929829	49.472	
	2	14.233	949666	50.528	
	Total		1879495	100.000	

PDA Ch5 234nm

Name	Peak#	Ret. Time	Area	Area%	Mark
	1	11.615	1808687	49.722	
	2	14.233	1828888	50.278	
	Total		3637575	100.000	

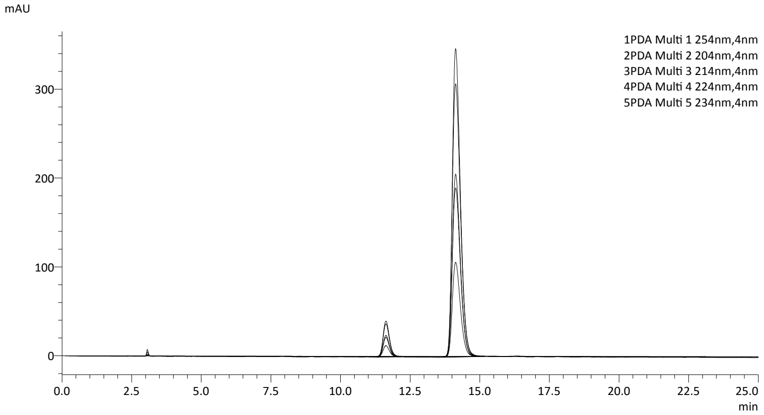

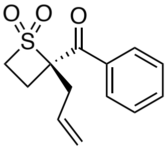

18a
(83% ee)

Peak Table

PDA Ch1 254nm

Name	Peak#	Ret. Time	Area	Area%	Mark
	1	11.631	631291	8.282	
	2	14.134	6990952	91.718	
	Total		7622244	100.000	

PDA Ch2 204nm

Name	Peak#	Ret. Time	Area	Area%	Mark
	1	11.631	587141	8.490	
	2	14.135	6328456	91.510	
	Total		6915597	100.000	

PDA Ch3 214nm

Name	Peak#	Ret. Time	Area	Area%	Mark
	1	11.631	350358	8.312	
	2	14.135	3864904	91.688	
	Total		4215262	100.000	

PDA Ch4 224nm

Name	Peak#	Ret. Time	Area	Area%	Mark
	1	11.631	192670	8.252	
	2	14.135	2142128	91.748	
	Total		2334798	100.000	

PDA Ch5 234nm

Name	Peak#	Ret. Time	Area	Area%	Mark
	1	11.631	373225	8.282	
	2	14.134	4132965	91.718	
	Total		4506190	100.000	

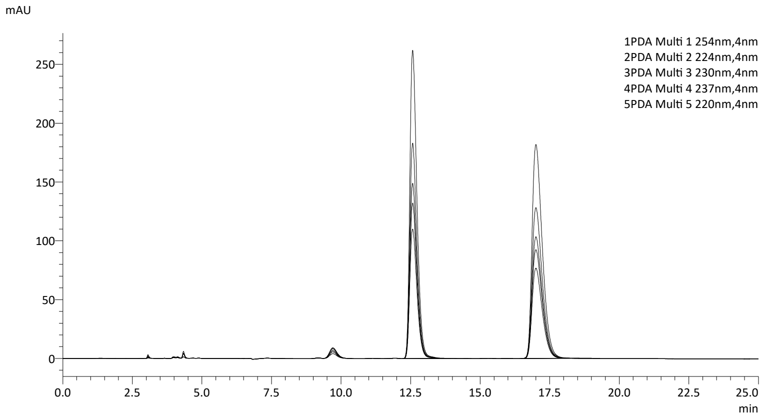

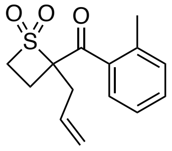

rac - **18b**

Peak Table

PDA Ch1 254nm

Name	Peak#	Ret. Time	Area	Area%	Mark
	1	12.574	4685894	50.072	
	2	17.004	4672436	49.928	
	Total		9358330	100.000	

PDA Ch2 224nm

Name	Peak#	Ret. Time	Area	Area%	Mark
	1	12.574	2381403	49.959	
	2	17.004	2385301	50.041	
	Total		4766704	100.000	

PDA Ch3 230nm

Name	Peak#	Ret. Time	Area	Area%	Mark
	1	12.574	1980454	50.093	
	2	17.004	1973071	49.907	
	Total		3953525	100.000	

PDA Ch4 237nm

Name	Peak#	Ret. Time	Area	Area%	Mark
	1	12.574	2661401	50.155	M
	2	17.004	2644912	49.845	M
	Total		5306313	100.000	

PDA Ch5 220nm

Name	Peak#	Ret. Time	Area	Area%	Mark
	1	12.574	3301178	49.888	
	2	17.004	3315996	50.112	
	Total		6617174	100.000	

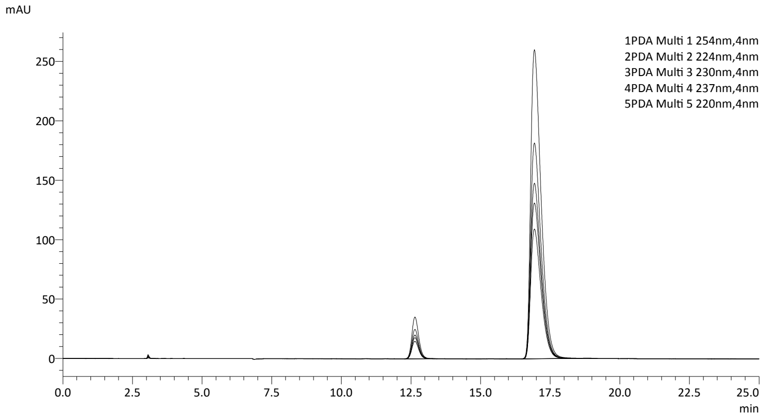

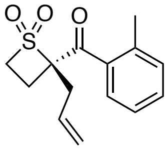

18b
(84% ee)

Peak Table

PDA Ch1 254nm

Name	Peak#	Ret. Time	Area	Area%	Mark
	1	12.643	604235	8.181	
	2	16.934	6781394	91.819	
	Total		7385629	100.000	

PDA Ch2 224nm

Name	Peak#	Ret. Time	Area	Area%	Mark
	1	12.643	305662	8.190	
	2	16.934	3426606	91.810	
	Total		3732268	100.000	

PDA Ch3 230nm

Name	Peak#	Ret. Time	Area	Area%	Mark
	1	12.643	253078	8.155	
	2	16.934	2850095	91.845	
	Total		3103172	100.000	

PDA Ch4 237nm

Name	Peak#	Ret. Time	Area	Area%	Mark
	1	12.643	343165	8.168	
	2	16.935	3858150	91.832	
	Total		4201314	100.000	

PDA Ch5 220nm

Name	Peak#	Ret. Time	Area	Area%	Mark
	1	12.643	427134	8.236	
	2	16.934	4759230	91.764	
	Total		5186364	100.000	

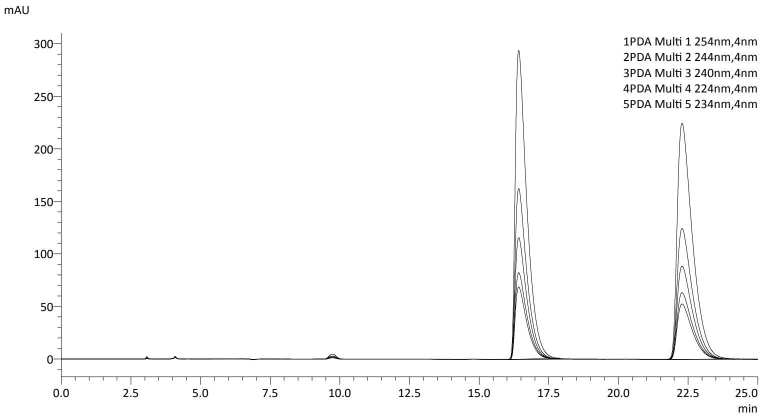

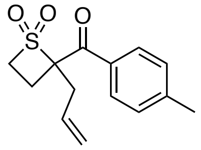

rac - **18c**

Peak Table

PDA Ch1 254nm

Name	Peak#	Ret. Time	Area	Area%	Mark
	1	16.420	8556998	49.822	M
	2	22.284	8617999	50.178	M
	Total		17174997	100.000	

PDA Ch2 244nm

Name	Peak#	Ret. Time	Area	Area%	Mark
	1	16.420	4772574	49.931	
	2	22.284	4785846	50.069	
	Total		9558420	100.000	

PDA Ch3 240nm

Name	Peak#	Ret. Time	Area	Area%	Mark
	1	16.420	3405739	49.931	
	2	22.284	3415118	50.069	
	Total		6820857	100.000	

PDA Ch4 224nm

Name	Peak#	Ret. Time	Area	Area%	Mark
	1	16.420	2450440	50.051	
	2	22.284	2445486	49.949	
	Total		4895925	100.000	

PDA Ch5 234nm

Name	Peak#	Ret. Time	Area	Area%	Mark
	1	16.420	2026919	49.997	
	2	22.284	2027158	50.003	
	Total		4054077	100.000	

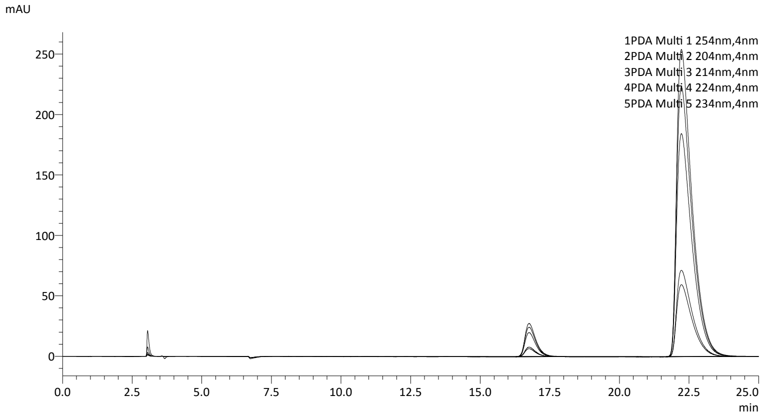

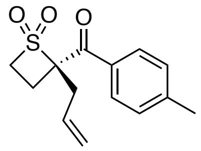

18c
(86% ee)

Peak Table

PDA Ch1 254nm

Name	Peak#	Ret. Time	Area	Area%	Mark
	1	16.756	751615	7.118	
	2	22.228	9807876	92.882	
	Total		10559491	100.000	

PDA Ch2 204nm

Name	Peak#	Ret. Time	Area	Area%	Mark
	1	16.755	666339	7.131	
	2	22.227	8678238	92.869	
	Total		9344578	100.000	

PDA Ch3 214nm

Name	Peak#	Ret. Time	Area	Area%	Mark
	1	16.756	548779	7.126	
	2	22.228	7152109	92.874	
	Total		7700888	100.000	

PDA Ch4 224nm

Name	Peak#	Ret. Time	Area	Area%	Mark
	1	16.755	212731	7.115	
	2	22.228	2777296	92.885	
	Total		2990028	100.000	

PDA Ch5 234nm

Name	Peak#	Ret. Time	Area	Area%	Mark
	1	16.755	175445	7.074	
	2	22.228	2304672	92.926	
	Total		2480118	100.000	

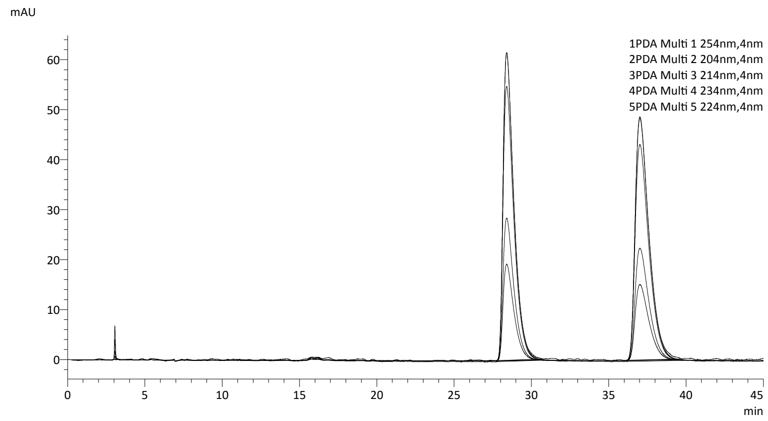

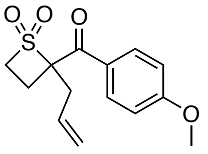

rac - **18d**

Peak Table

PDA Ch1 254nm

Name	Peak#	Ret. Time	Area	Area%	Mark
	1	28.401	1401540	50.032	
	2	37.017	1399744	49.968	
	Total		2801283	100.000	

PDA Ch2 204nm

Name	Peak#	Ret. Time	Area	Area%	Mark
	1	28.400	3030298	49.657	
	2	37.020	3072219	50.343	S
	Total		6102517	100.000	

PDA Ch3 214nm

Name	Peak#	Ret. Time	Area	Area%	Mark
	1	28.401	2707406	49.868	
	2	37.019	2721768	50.132	
	Total		5429174	100.000	

PDA Ch4 234nm

Name	Peak#	Ret. Time	Area	Area%	Mark
	1	28.401	944017	50.023	
	2	37.020	943153	49.977	
	Total		1887169	100.000	

PDA Ch5 224nm

Name	Peak#	Ret. Time	Area	Area%	Mark
	1	28.401	3030030	49.956	
	2	37.019	3035307	50.044	
	Total		6065337	100.000	

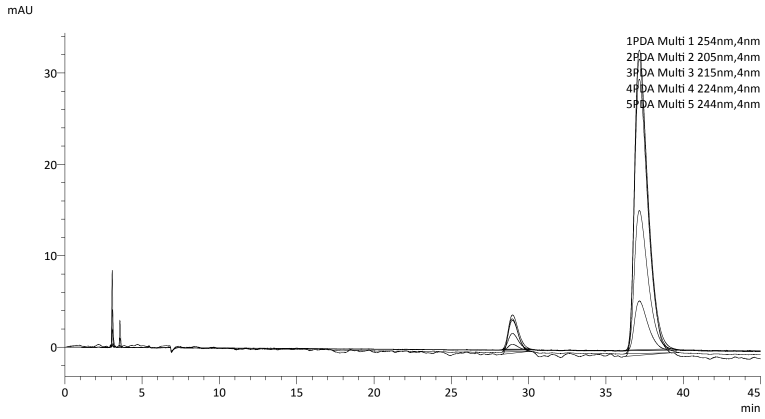

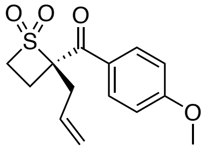

18d
(84% ee)

Peak Table

PDA Ch1 254nm

Name	Peak#	Ret. Time	Area	Area%	Mark
	1	28.963	76679	7.644	
	2	37.164	926459	92.356	
	Total		1003138	100.000	

PDA Ch2 205nm

Name	Peak#	Ret. Time	Area	Area%	Mark
	1	28.945	170102	7.961	
	2	37.163	1966606	92.039	
	Total		2136709	100.000	

PDA Ch3 215nm

Name	Peak#	Ret. Time	Area	Area%	Mark
	1	28.955	156164	7.800	
	2	37.165	1845908	92.200	
	Total		2002072	100.000	

PDA Ch4 224nm

Name	Peak#	Ret. Time	Area	Area%	Mark
	1	28.960	175276	7.985	
	2	37.165	2019880	92.015	
	Total		2195156	100.000	

PDA Ch5 244nm

Name	Peak#	Ret. Time	Area	Area%	Mark
	1	28.978	28742	7.969	M
	2	37.166	331939	92.031	M
	Total		360681	100.000	

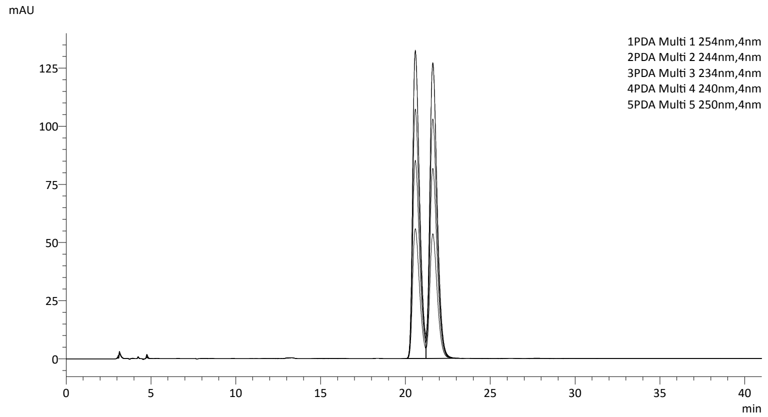

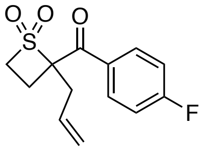

rac - **18e**

Peak Table

PDA Ch1 254nm

Name	Peak#	Ret. Time	Area	Area%	Mark
	1	20.584	3768514	49.248	
	2	21.616	3883539	50.752	V
	Total		7652053	100.000	

PDA Ch2 244nm

Name	Peak#	Ret. Time	Area	Area%	Mark
	1	20.584	3059147	49.258	
	2	21.616	3151345	50.742	V
	Total		6210492	100.000	

PDA Ch3 234nm

Name	Peak#	Ret. Time	Area	Area%	Mark
	1	20.584	1591999	49.266	
	2	21.616	1639456	50.734	V
	Total		3231455	100.000	

PDA Ch4 240nm

Name	Peak#	Ret. Time	Area	Area%	Mark
	1	20.585	2429789	49.264	
	2	21.616	2502424	50.736	V
	Total		4932213	100.000	

PDA Ch5 250nm

Name	Peak#	Ret. Time	Area	Area%	Mark
	1	20.585	3770999	49.252	
	2	21.616	3885521	50.748	V
	Total		7656520	100.000	

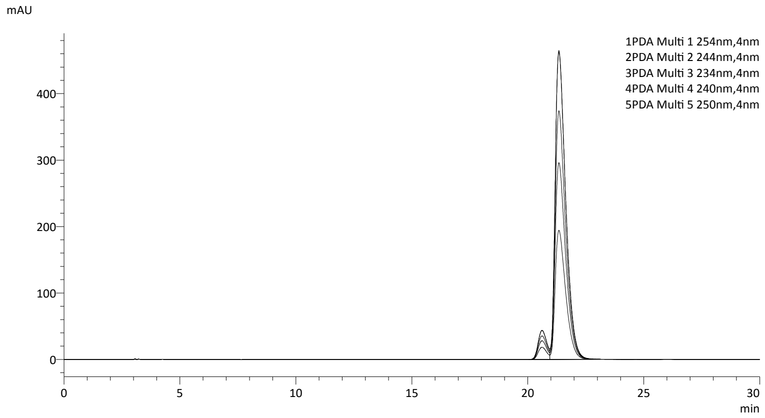

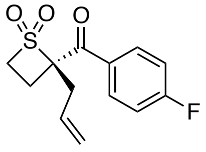

18e
(86% ee)

Peak Table

PDA Ch1 254nm

Name	Peak#	Ret. Time	Area	Area%	Mark
	1	20.615	1111978	7.059	
	2	21.343	14641297	92.941	V
	Total		15753275	100.000	

PDA Ch2 244nm

Name	Peak#	Ret. Time	Area	Area%	Mark
	1	20.615	904028	7.086	
	2	21.343	11854476	92.914	V
	Total		12758504	100.000	

PDA Ch3 234nm

Name	Peak#	Ret. Time	Area	Area%	Mark
	1	20.615	469998	7.080	
	2	21.343	6168460	92.920	V
	Total		6638457	100.000	

PDA Ch4 240nm

Name	Peak#	Ret. Time	Area	Area%	Mark
	1	20.615	718317	7.103	
	2	21.343	9395087	92.897	V
	Total		10113404	100.000	

PDA Ch5 250nm

Name	Peak#	Ret. Time	Area	Area%	Mark
	1	20.615	1111121	7.034	
	2	21.343	14684379	92.966	V
	Total		15795500	100.000	

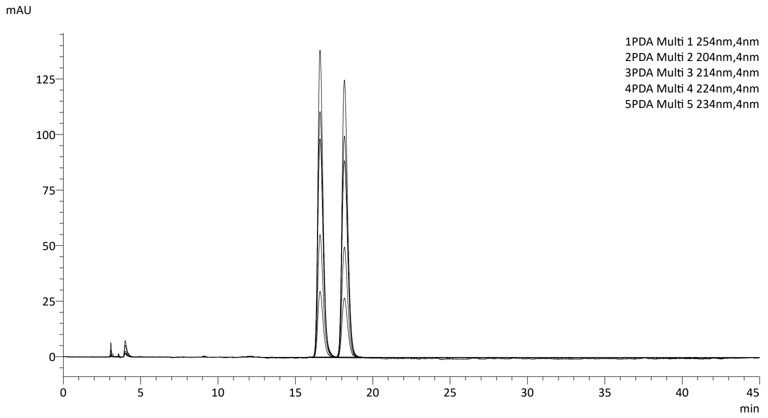

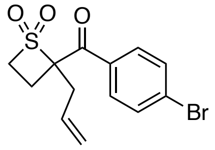

rac - **18f**

Peak Table

PDA Ch1 254nm

Name	Peak#	Ret. Time	Area	Area%	Mark
	1	16.595	3233691	50.009	
	2	18.170	3232570	49.991	V
	Total		6466261	100.000	

PDA Ch2 204nm

Name	Peak#	Ret. Time	Area	Area%	Mark
	1	16.596	2682061	50.843	
	2	18.170	2593146	49.157	V
	Total		5275207	100.000	

PDA Ch3 214nm

Name	Peak#	Ret. Time	Area	Area%	Mark
	1	16.596	2364849	50.782	
	2	18.170	2291994	49.218	V
	Total		4656843	100.000	

PDA Ch4 224nm

Name	Peak#	Ret. Time	Area	Area%	Mark
	1	16.596	1346210	51.160	
	2	18.170	1285138	48.840	V
	Total		2631349	100.000	

PDA Ch5 234nm

Name	Peak#	Ret. Time	Area	Area%	Mark
	1	16.596	716859	50.909	
	2	18.170	691252	49.091	V
	Total		1408111	100.000	

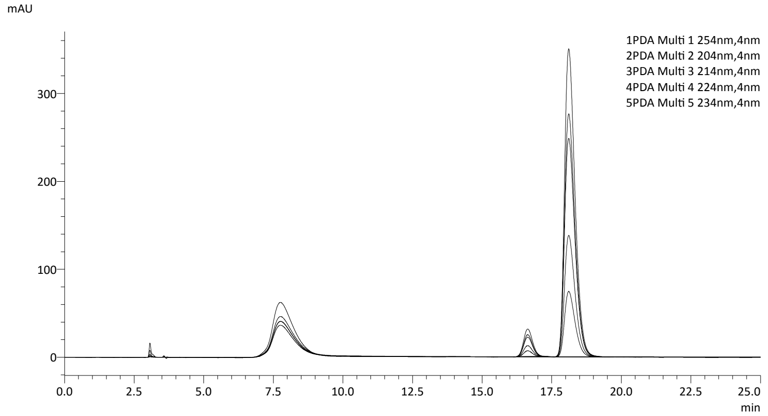

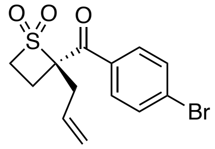

18f
(85% ee)

Peak Table

PDA Ch1 254nm

Name	Peak#	Ret. Time	Area	Area%	Mark
	1	16.639	743057	7.438	
	2	18.112	9246451	92.562	V
	Total		9989508	100.000	

PDA Ch2 204nm

Name	Peak#	Ret. Time	Area	Area%	Mark
	1	16.639	552126	7.006	M
	2	18.112	7328469	92.994	
	Total		7880595	100.000	

PDA Ch3 214nm

Name	Peak#	Ret. Time	Area	Area%	Mark
	1	16.639	522046	7.371	
	2	18.112	6560242	92.629	V
	Total		7082289	100.000	

PDA Ch4 224nm

Name	Peak#	Ret. Time	Area	Area%	Mark
	1	16.639	292519	7.405	
	2	18.112	3657775	92.595	
	Total		3950293	100.000	

PDA Ch5 234nm

Name	Peak#	Ret. Time	Area	Area%	Mark
	1	16.639	158266	7.438	
	2	18.112	1969589	92.562	
	Total		2127855	100.000	

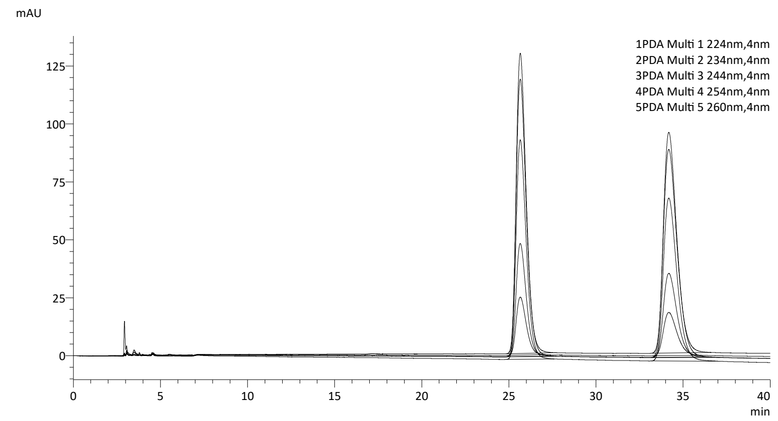

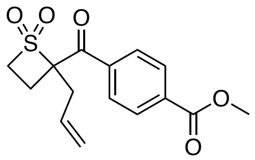

rac - **18g**

Peak Table

PDA Ch1 224nm

Name	Peak#	Ret. Time	Area	Area%	Mark
	1	25.656	1020566	49.919	
	2	34.187	1023864	50.081	
	Total		2044431	100.000	

PDA Ch2 234nm

Name	Peak#	Ret. Time	Area	Area%	Mark
	1	25.656	1999201	50.080	
	2	34.185	1992831	49.920	
	Total		3992032	100.000	

PDA Ch3 244nm

Name	Peak#	Ret. Time	Area	Area%	Mark
	1	25.655	3870277	49.850	
	2	34.184	3893627	50.150	
	Total		7763904	100.000	

PDA Ch4 254nm

Name	Peak#	Ret. Time	Area	Area%	Mark
	1	25.655	5358427	49.962	
	2	34.184	5366612	50.038	
	Total		10725038	100.000	

PDA Ch5 260nm

Name	Peak#	Ret. Time	Area	Area%	Mark
	1	25.655	4839398	50.030	
	2	34.184	4833631	49.970	
	Total		9673029	100.000	

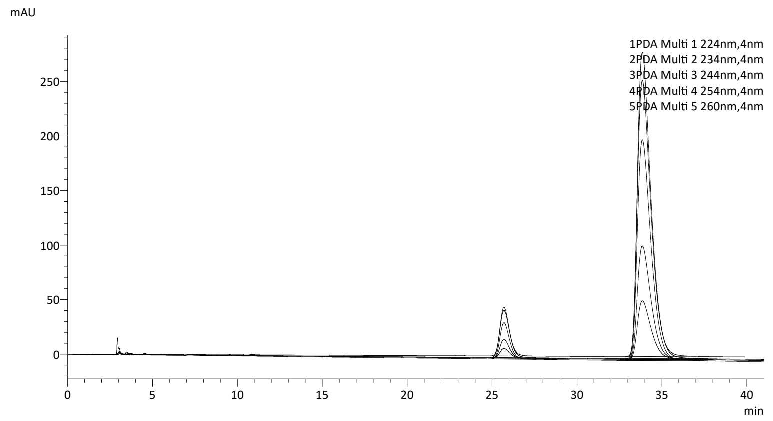

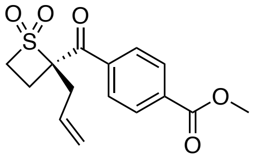

18g
(79% ee)

Peak Table

PDA Ch1 224nm

Name	Peak#	Ret. Time	Area	Area%	Mark
	1	25.701	356953	10.448	
	2	33.852	3059625	89.552	
	Total		3416578	100.000	

PDA Ch2 234nm

Name	Peak#	Ret. Time	Area	Area%	Mark
	1	25.703	694894	10.477	
	2	33.851	5937488	89.523	
	Total		6632382	100.000	

PDA Ch3 244nm

Name	Peak#	Ret. Time	Area	Area%	Mark
	1	25.702	1344978	10.470	
	2	33.850	11501433	89.530	
	Total		12846411	100.000	

PDA Ch4 254nm

Name	Peak#	Ret. Time	Area	Area%	Mark
	1	25.703	1854475	10.444	
	2	33.850	15902618	89.556	
	Total		17757093	100.000	

PDA Ch5 260nm

Name	Peak#	Ret. Time	Area	Area%	Mark
	1	25.703	1676122	10.461	
	2	33.850	14346164	89.539	
	Total		16022287	100.000	

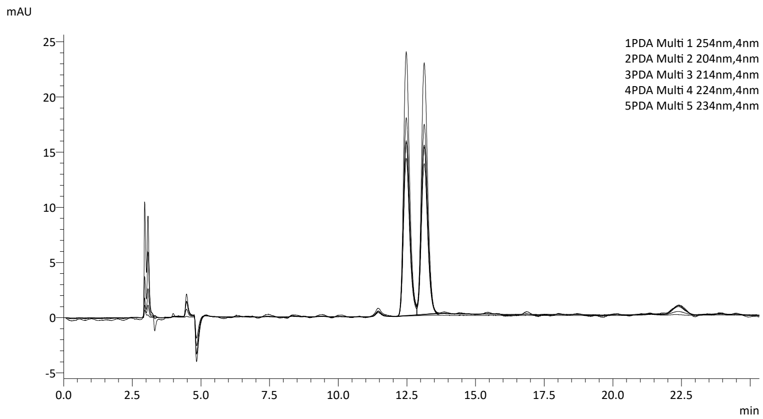

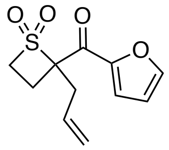

rac - **18h**

Peak Table

PDA Ch1 254nm

Name	Peak#	Ret. Time	Area	Area%	Mark
	1	12.475	372983	50.073	
	2	13.130	371902	49.927	V
	Total		744885	100.000	

PDA Ch2 204nm

Name	Peak#	Ret. Time	Area	Area%	Mark
	1	12.475	252052	49.977	
	2	13.130	252286	50.023	V
	Total		504337	100.000	

PDA Ch3 214nm

Name	Peak#	Ret. Time	Area	Area%	Mark
	1	12.476	248298	49.946	
	2	13.131	248833	50.054	V
	Total		497131	100.000	

PDA Ch4 224nm

Name	Peak#	Ret. Time	Area	Area%	Mark
	1	12.476	281088	49.948	
	2	13.130	281677	50.052	V
	Total		562765	100.000	

PDA Ch5 234nm

Name	Peak#	Ret. Time	Area	Area%	Mark
	1	12.476	222806	50.063	
	2	13.131	222246	49.937	V
	Total		445052	100.000	

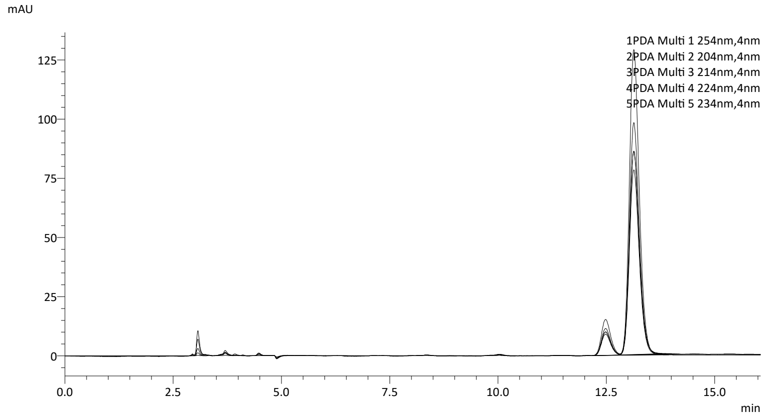

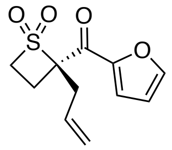

18h
(81% ee)

Peak Table

PDA Ch1 254nm

Name	Peak#	Ret. Time	Area	Area%	Mark
	1	12.483	228150	9.882	
	2	13.135	2080681	90.118	V
	Total		2308832	100.000	

PDA Ch2 204nm

Name	Peak#	Ret. Time	Area	Area%	Mark
	1	12.484	146525	9.524	
	2	13.136	1392025	90.476	V
	Total		1538550	100.000	

PDA Ch3 214nm

Name	Peak#	Ret. Time	Area	Area%	Mark
	1	12.484	149743	9.650	
	2	13.136	1401965	90.350	V
	Total		1551708	100.000	

PDA Ch4 224nm

Name	Peak#	Ret. Time	Area	Area%	Mark
	1	12.484	170101	9.718	
	2	13.136	1580309	90.282	V
	Total		1750410	100.000	

PDA Ch5 234nm

Name	Peak#	Ret. Time	Area	Area%	Mark
	1	12.483	134096	9.662	
	2	13.135	1253731	90.338	V
	Total		1387827	100.000	

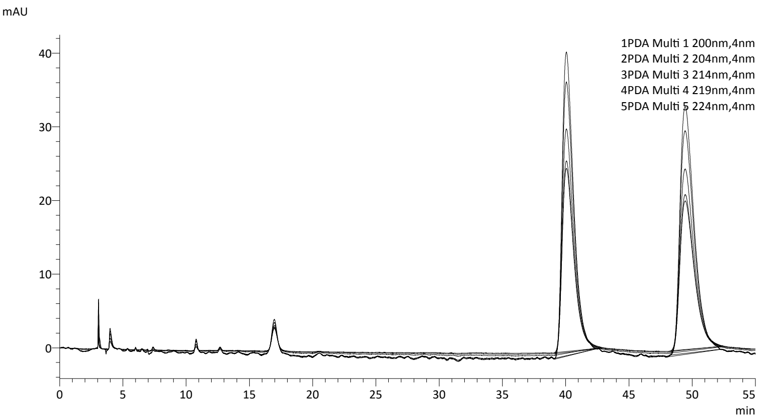

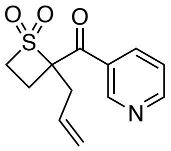

rac - **18i**

Peak Table

PDA Ch1 200nm

Name	Peak#	Ret. Time	Area	Area%	Mark
	1	40.056	2384610	49.769	S
	2	49.433	2406793	50.231	SV
	Total		4791403	100.000	

PDA Ch2 204nm

Name	Peak#	Ret. Time	Area	Area%	Mark
	1	40.054	2640651	49.589	
	2	49.440	2684395	50.411	
	Total		5325045	100.000	

PDA Ch3 214nm

Name	Peak#	Ret. Time	Area	Area%	Mark
	1	40.061	1694587	49.809	
	2	49.446	1707574	50.191	
	Total		3402162	100.000	

PDA Ch4 219nm

Name	Peak#	Ret. Time	Area	Area%	Mark
	1	40.059	1638592	50.320	
	2	49.444	1617721	49.680	
	Total		3256313	100.000	

PDA Ch5 224nm

Name	Peak#	Ret. Time	Area	Area%	Mark
	1	40.055	1958259	50.138	
	2	49.439	1947482	49.862	
	Total		3905742	100.000	

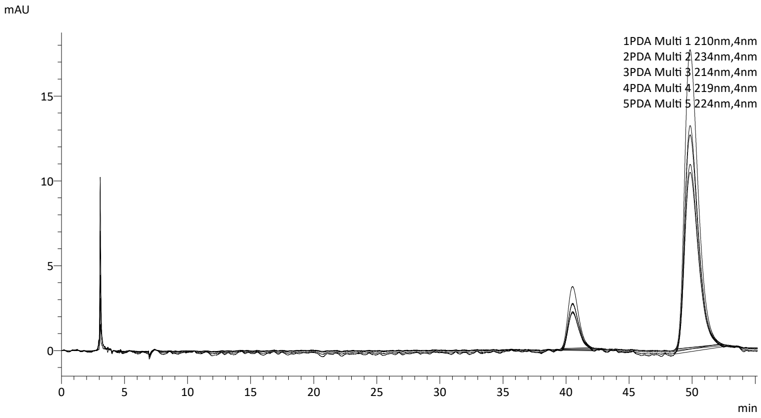

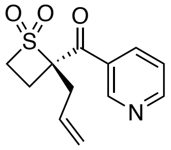

18i
(72% ee)

Peak Table

PDA Ch1 210nm

Name	Peak#	Ret. Time	Area	Area%	Mark
	1	40.530	168389	14.103	
	2	49.845	1025573	85.897	
	Total		1193962	100.000	

PDA Ch2 234nm

Name	Peak#	Ret. Time	Area	Area%	Mark
	1	40.523	213851	13.938	
	2	49.834	1320457	86.062	
	Total		1534308	100.000	

PDA Ch3 214nm

Name	Peak#	Ret. Time	Area	Area%	Mark
	1	40.534	135528	14.125	
	2	49.848	823981	85.875	
	Total		959509	100.000	

PDA Ch4 219nm

Name	Peak#	Ret. Time	Area	Area%	Mark
	1	40.532	129782	13.975	M
	2	49.845	798899	86.025	M
	Total		928681	100.000	

PDA Ch5 224nm

Name	Peak#	Ret. Time	Area	Area%	Mark
	1	40.521	153577	13.909	
	2	49.845	950587	86.091	
	Total		1104164	100.000	

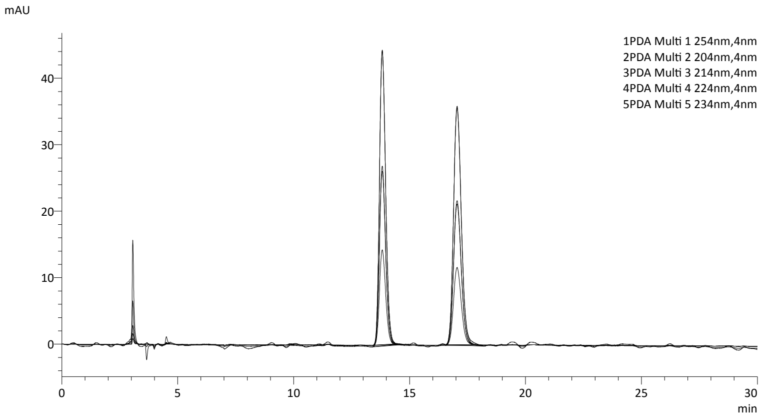

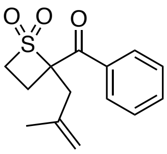

rac - **18j**

Peak Table

PDA Ch1 254nm

Name	Peak#	Ret. Time	Area	Area%	Mark
	1	13.822	824755	49.830	
	2	17.049	830388	50.170	
	Total		1655143	100.000	

PDA Ch2 204nm

Name	Peak#	Ret. Time	Area	Area%	Mark
	1	13.822	836462	49.494	S
	2	17.049	853560	50.506	
	Total		1690022	100.000	

PDA Ch3 214nm

Name	Peak#	Ret. Time	Area	Area%	Mark
	1	13.822	489063	49.337	
	2	17.050	502207	50.663	
	Total		991271	100.000	

PDA Ch4 224nm

Name	Peak#	Ret. Time	Area	Area%	Mark
	1	13.822	266204	49.435	
	2	17.050	272290	50.565	
	Total		538495	100.000	

PDA Ch5 234nm

Name	Peak#	Ret. Time	Area	Area%	Mark
	1	13.822	500797	49.764	
	2	17.049	505543	50.236	
	Total		1006341	100.000	

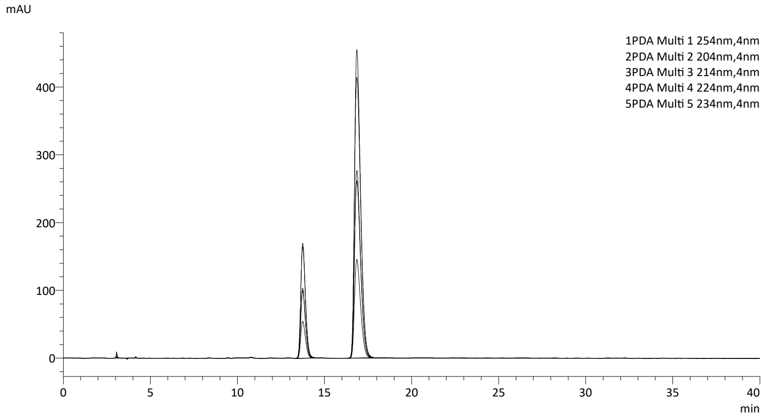

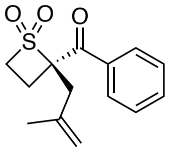

18j
(55% ee)

Peak Table

PDA Ch1 254nm

Name	Peak#	Ret. Time	Area	Area%	Mark
	1	13.750	3207839	22.368	
	2	16.860	11133625	77.632	
	Total		14341464	100.000	

PDA Ch2 204nm

Name	Peak#	Ret. Time	Area	Area%	Mark
	1	13.750	3144951	23.124	
	2	16.860	10455171	76.876	V
	Total		13600122	100.000	

PDA Ch3 214nm

Name	Peak#	Ret. Time	Area	Area%	Mark
	1	13.750	1890439	22.630	
	2	16.860	6463337	77.370	
	Total		8353776	100.000	

PDA Ch4 224nm

Name	Peak#	Ret. Time	Area	Area%	Mark
	1	13.750	1033248	22.457	
	2	16.860	3567750	77.543	
	Total		4600998	100.000	

PDA Ch5 234nm

Name	Peak#	Ret. Time	Area	Area%	Mark
	1	13.750	1948387	22.345	
	2	16.860	6771140	77.655	
	Total		8719527	100.000	

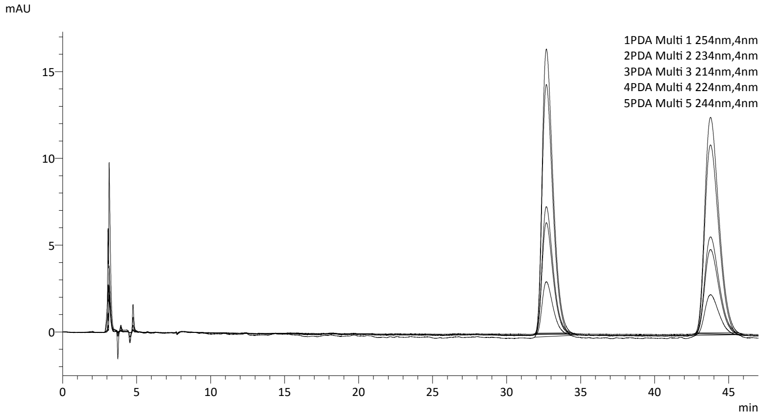

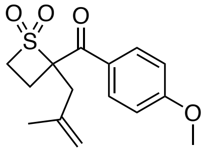

rac - **18l**

Peak Table

PDA Ch1 254nm

Name	Peak#	Ret. Time	Area	Area%	Mark
	1	32.683	374407	50.396	
	2	43.778	368526	49.604	
	Total		742933	100.000	

PDA Ch2 234nm

Name	Peak#	Ret. Time	Area	Area%	Mark
	1	32.687	328756	50.364	
	2	43.778	324007	49.636	
	Total		652762	100.000	

PDA Ch3 214nm

Name	Peak#	Ret. Time	Area	Area%	Mark
	1	32.684	749251	50.553	
	2	43.780	732856	49.447	
	Total		1482108	100.000	

PDA Ch4 224nm

Name	Peak#	Ret. Time	Area	Area%	Mark
	1	32.685	852761	50.151	
	2	43.778	847624	49.849	
	Total		1700385	100.000	

PDA Ch5 244nm

Name	Peak#	Ret. Time	Area	Area%	Mark
	1	32.690	153568	51.286	
	2	43.775	145867	48.714	
	Total		299435	100.000	

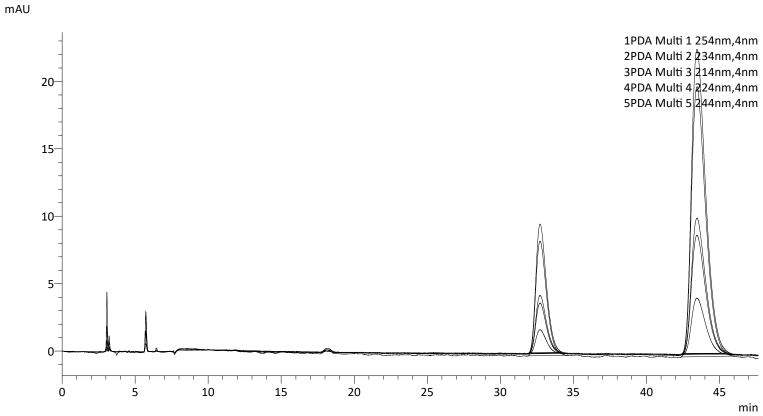

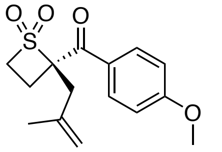

18l
(52% ee)

Peak Table

PDA Ch1 254nm

Name	Peak#	Ret. Time	Area	Area%	Mark
	1	32.719	214136	23.884	
	2	43.455	682445	76.116	
	Total		896581	100.000	

PDA Ch2 234nm

Name	Peak#	Ret. Time	Area	Area%	Mark
	1	32.720	188847	24.067	
	2	43.459	595815	75.933	
	Total		784662	100.000	

PDA Ch3 214nm

Name	Peak#	Ret. Time	Area	Area%	Mark
	1	32.718	443496	24.348	
	2	43.459	1378021	75.652	
	Total		1821517	100.000	

PDA Ch4 224nm

Name	Peak#	Ret. Time	Area	Area%	Mark
	1	32.720	497064	24.213	
	2	43.455	1555834	75.787	
	Total		2052897	100.000	

PDA Ch5 244nm

Name	Peak#	Ret. Time	Area	Area%	Mark
	1	32.718	84830	23.610	
	2	43.458	274473	76.390	
	Total		359304	100.000	

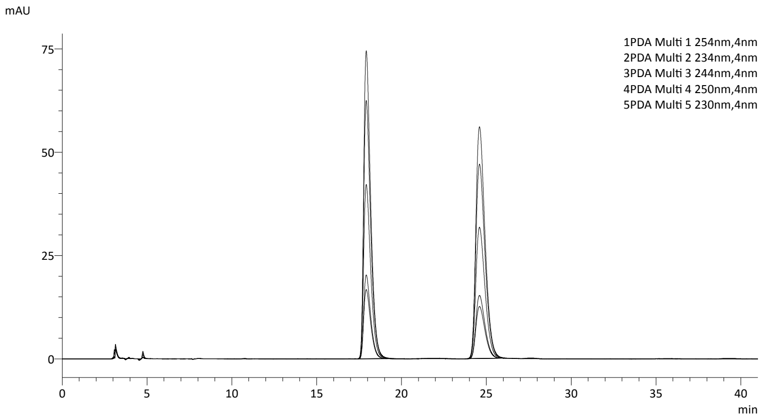

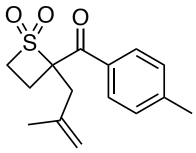

rac - **18k**

Peak Table

PDA Ch1 254nm

Name	Peak#	Ret. Time	Area	Area%	Mark
	1	17.924	2129976	50.037	
	2	24.597	2126868	49.963	
	Total		4256844	100.000	

PDA Ch2 234nm

Name	Peak#	Ret. Time	Area	Area%	Mark
	1	17.923	579868	50.229	
	2	24.596	574575	49.771	
	Total		1154443	100.000	

PDA Ch3 244nm

Name	Peak#	Ret. Time	Area	Area%	Mark
	1	17.924	1205707	50.041	
	2	24.596	1203716	49.959	
	Total		2409423	100.000	

PDA Ch4 250nm

Name	Peak#	Ret. Time	Area	Area%	Mark
	1	17.924	1786165	50.072	
	2	24.596	1781027	49.928	
	Total		3567191	100.000	

PDA Ch5 230nm

Name	Peak#	Ret. Time	Area	Area%	Mark
	1	17.924	478202	50.261	
	2	24.595	473227	49.739	
	Total		951429	100.000	

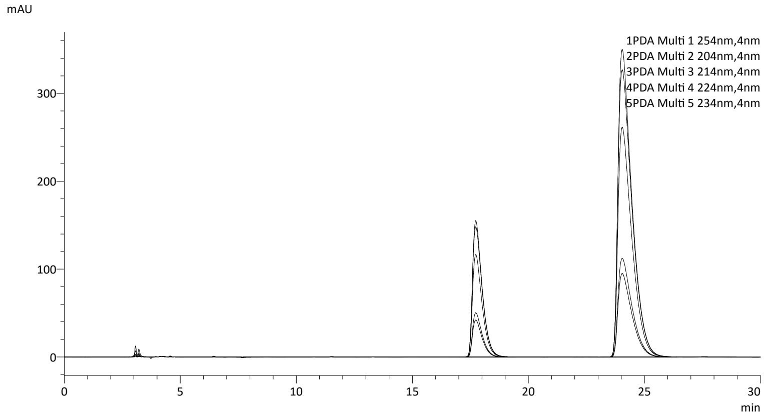

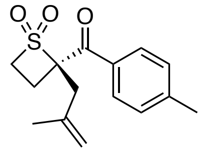

18k
(53% ee)

Peak Table

PDA Ch1 254nm

Name	Peak#	Ret. Time	Area	Area%	Mark
	1	17.733	4510810	23.601	
	2	24.043	14601592	76.399	
	Total		19112401	100.000	

PDA Ch2 204nm

Name	Peak#	Ret. Time	Area	Area%	Mark
	1	17.733	4342379	23.897	
	2	24.043	13828644	76.103	
	Total		18171024	100.000	

PDA Ch3 214nm

Name	Peak#	Ret. Time	Area	Area%	Mark
	1	17.733	3401381	23.684	
	2	24.043	10959979	76.316	
	Total		14361360	100.000	

PDA Ch4 224nm

Name	Peak#	Ret. Time	Area	Area%	Mark
	1	17.733	1468801	23.744	
	2	24.043	4717058	76.256	
	Total		6185860	100.000	

PDA Ch5 234nm

Name	Peak#	Ret. Time	Area	Area%	Mark
	1	17.733	1226194	23.603	
	2	24.043	3968847	76.397	
	Total		5195041	100.000	

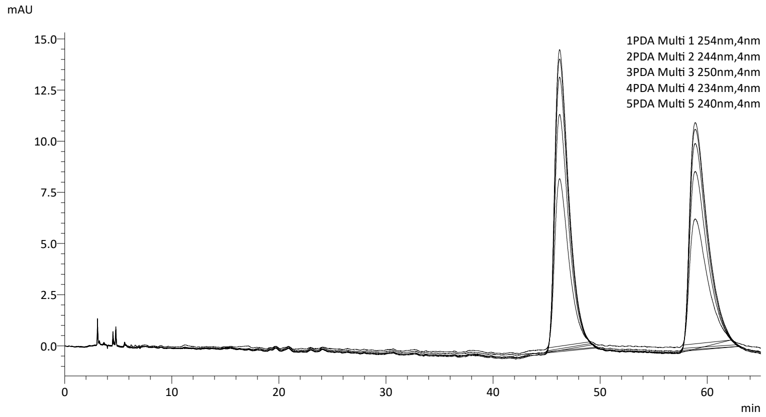

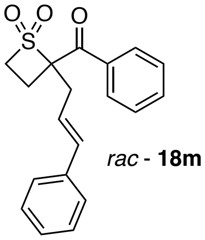

rac - **18m**

Peak Table

PDA Ch1 254nm

Name	Peak#	Ret. Time	Area	Area%	Mark
	1	46.226	1334967	50.734	
	2	58.883	1296334	49.266	
	Total		2631301	100.000	

PDA Ch2 244nm

Name	Peak#	Ret. Time	Area	Area%	Mark
	1	46.216	1233544	51.495	
	2	58.893	1161924	48.505	M
	Total		2395468	100.000	

PDA Ch3 250nm

Name	Peak#	Ret. Time	Area	Area%	Mark
	1	46.224	1381642	50.872	
	2	58.893	1334271	49.128	
	Total		2715913	100.000	

PDA Ch4 234nm

Name	Peak#	Ret. Time	Area	Area%	Mark
	1	46.227	751921	51.562	
	2	58.873	706358	48.438	
	Total		1458280	100.000	

PDA Ch5 240nm

Name	Peak#	Ret. Time	Area	Area%	Mark
	1	46.216	1055578	50.806	
	2	58.872	1022088	49.194	
	Total		2077666	100.000	

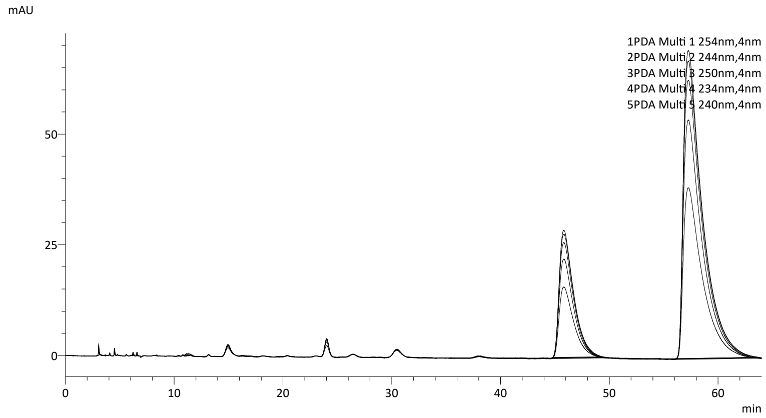

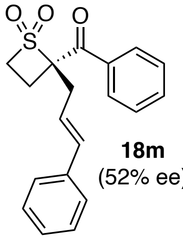

18m
(52% ee)

Peak Table

PDA Ch1 254nm

Name	Peak#	Ret. Time	Area	Area%	Mark
	1	45.816	2624614	24.181	
	2	57.268	8229256	75.819	
	Total		10853870	100.000	

PDA Ch2 244nm

Name	Peak#	Ret. Time	Area	Area%	Mark
	1	45.819	2461878	24.154	
	2	57.267	7730449	75.846	
	Total		10192327	100.000	

PDA Ch3 250nm

Name	Peak#	Ret. Time	Area	Area%	Mark
	1	45.818	2718873	24.164	
	2	57.266	8532674	75.836	
	Total		11251547	100.000	

PDA Ch4 234nm

Name	Peak#	Ret. Time	Area	Area%	Mark
	1	45.821	1480396	24.005	M
	2	57.269	4686671	75.995	
	Total		6167067	100.000	

PDA Ch5 240nm

Name	Peak#	Ret. Time	Area	Area%	Mark
	1	45.819	2036957	23.665	M
	2	57.267	6570520	76.335	
	Total		8607478	100.000	

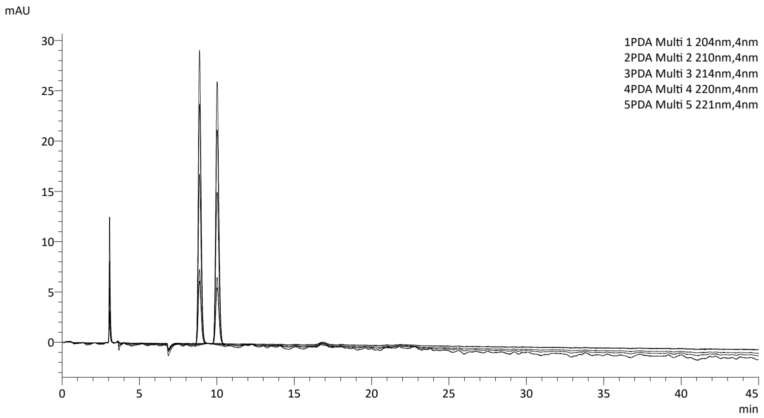

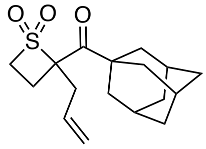

rac - **18o**

Peak Table

PDA Ch1 204nm

Name	Peak#	Ret. Time	Area	Area%	Mark
	1	8.881	373735	50.062	
	2	10.019	372802	49.938	
	Total		746537	100.000	

PDA Ch2 210nm

Name	Peak#	Ret. Time	Area	Area%	Mark
	1	8.882	304742	50.075	
	2	10.019	303830	49.925	
	Total		608572	100.000	

PDA Ch3 214nm

Name	Peak#	Ret. Time	Area	Area%	Mark
	1	8.882	215615	50.071	
	2	10.019	215004	49.929	
	Total		430620	100.000	

PDA Ch4 220nm

Name	Peak#	Ret. Time	Area	Area%	Mark
	1	8.881	93633	50.074	
	2	10.019	93356	49.926	
	Total		186989	100.000	

PDA Ch5 221nm

Name	Peak#	Ret. Time	Area	Area%	Mark
	1	8.881	79039	50.084	
	2	10.019	78774	49.916	
	Total		157813	100.000	

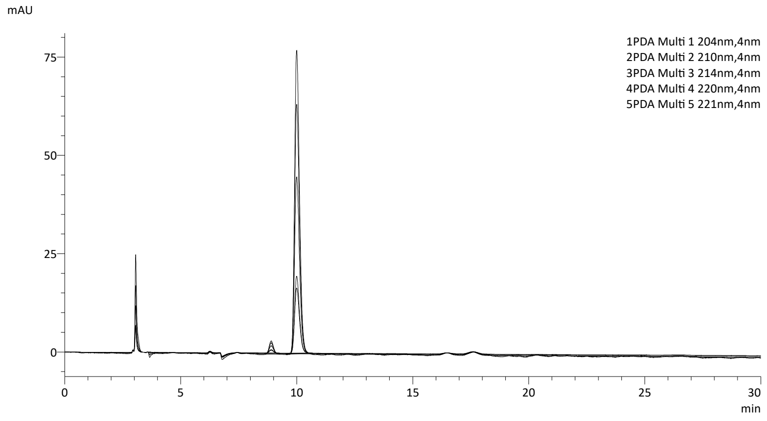

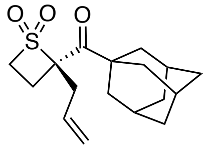

18o
(93% ee)

Peak Table

PDA Ch1 204nm

Name	Peak#	Ret. Time	Area	Area%	Mark
	1	8.900	44101	3.721	M
	2	9.999	1140977	96.279	M
	Total		1185079	100.000	

PDA Ch2 210nm

Name	Peak#	Ret. Time	Area	Area%	Mark
	1	8.900	31353	3.239	M
	2	9.999	936730	96.761	M
	Total		968083	100.000	

PDA Ch3 214nm

Name	Peak#	Ret. Time	Area	Area%	Mark
	1	8.901	25333	3.685	M
	2	9.999	662177	96.315	M
	Total		687511	100.000	

PDA Ch4 220nm

Name	Peak#	Ret. Time	Area	Area%	Mark
	1	8.903	11340	3.766	
	2	9.999	289804	96.234	
	Total		301144	100.000	

PDA Ch5 221nm

Name	Peak#	Ret. Time	Area	Area%	Mark
	1	8.903	9467	3.723	
	2	9.999	244847	96.277	
	Total		254314	100.000	

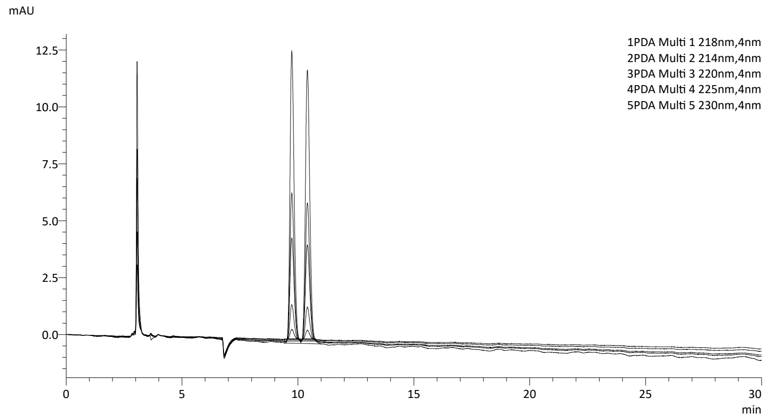

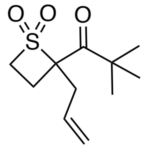

rac - **18p**

Peak Table

PDA Ch1 218nm

Name	Peak#	Ret. Time	Area	Area%	Mark
	1	9.737	75854	49.755	M
	2	10.410	76602	50.245	M
	Total		152455	100.000	

PDA Ch2 214nm

Name	Peak#	Ret. Time	Area	Area%	Mark
	1	9.737	160270	49.796	
	2	10.410	161583	50.204	V
	Total		321853	100.000	

PDA Ch3 220nm

Name	Peak#	Ret. Time	Area	Area%	Mark
	1	9.736	56537	50.168	
	2	10.410	56159	49.832	V
	Total		112696	100.000	

PDA Ch4 225nm

Name	Peak#	Ret. Time	Area	Area%	Mark
	1	9.736	19394	50.318	
	2	10.410	19149	49.682	V
	Total		38544	100.000	

PDA Ch5 230nm

Name	Peak#	Ret. Time	Area	Area%	Mark
	1	9.734	5203	50.644	
	2	10.412	5071	49.356	
	Total		10274	100.000	

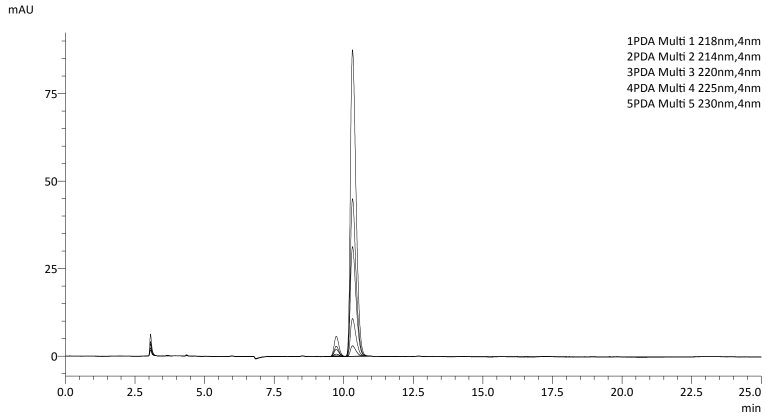

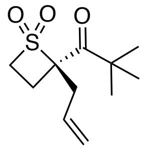

18p
(90% ee)

Peak Table

PDA Ch1 218nm

Name	Peak#	Ret. Time	Area	Area%	Mark
	1	9.734	35044	5.249	
	2	10.317	632648	94.751	
	Total		667692	100.000	

PDA Ch2 214nm

Name	Peak#	Ret. Time	Area	Area%	Mark
	1	9.734	69864	5.327	
	2	10.317	1241672	94.673	V
	Total		1311536	100.000	

PDA Ch3 220nm

Name	Peak#	Ret. Time	Area	Area%	Mark
	1	9.734	24297	5.240	
	2	10.317	439400	94.760	
	Total		463697	100.000	

PDA Ch4 225nm

Name	Peak#	Ret. Time	Area	Area%	Mark
	1	9.732	8213	5.166	
	2	10.317	150778	94.834	
	Total		158991	100.000	

PDA Ch5 230nm

Name	Peak#	Ret. Time	Area	Area%	Mark
	1	9.733	2045	4.624	
	2	10.318	42188	95.376	
	Total		44233	100.000	

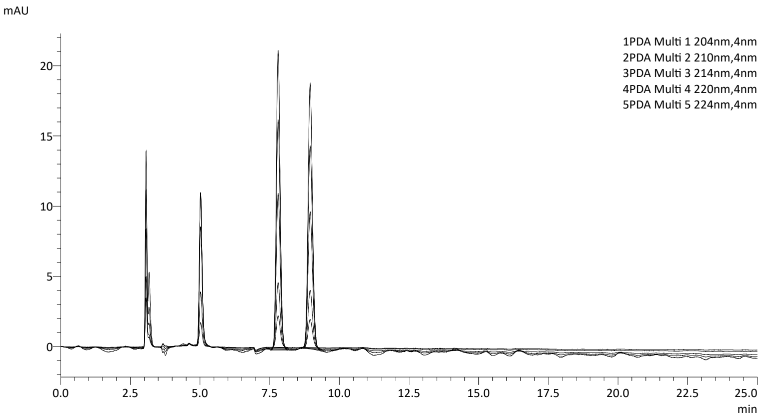

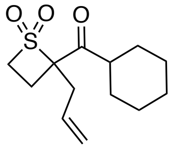

rac - **18q**

Peak Table

PDA Ch1 204nm

Name	Peak#	Ret. Time	Area	Area%	Mark
	1	7.802	212140	49.728	
	2	8.952	214461	50.272	
	Total		426601	100.000	

PDA Ch2 210nm

Name	Peak#	Ret. Time	Area	Area%	Mark
	1	7.803	162808	49.878	
	2	8.952	163605	50.122	
	Total		326413	100.000	

PDA Ch3 214nm

Name	Peak#	Ret. Time	Area	Area%	Mark
	1	7.803	110272	49.944	
	2	8.952	110519	50.056	
	Total		220790	100.000	

PDA Ch4 220nm

Name	Peak#	Ret. Time	Area	Area%	Mark
	1	7.803	46547	50.023	
	2	8.953	46503	49.977	
	Total		93050	100.000	

PDA Ch5 224nm

Name	Peak#	Ret. Time	Area	Area%	Mark
	1	7.803	22600	50.001	
	2	8.952	22599	49.999	
	Total		45200	100.000	

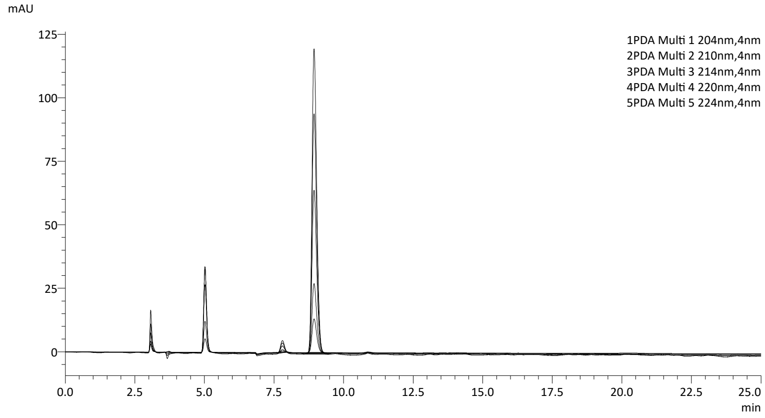

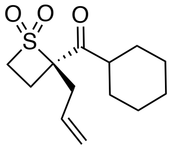

18q
(94% ee)

Peak Table

PDA Ch1 204nm

Name	Peak#	Ret. Time	Area	Area%	Mark
	1	7.803	46204	3.135	M
	2	8.940	1427603	96.865	
	Total		1473807	100.000	

PDA Ch2 210nm

Name	Peak#	Ret. Time	Area	Area%	Mark
	1	7.803	34378	2.996	M
	2	8.940	1113178	97.004	
	Total		1147555	100.000	

PDA Ch3 214nm

Name	Peak#	Ret. Time	Area	Area%	Mark
	1	7.804	23027	2.958	M
	2	8.940	755378	97.042	
	Total		778405	100.000	

PDA Ch4 220nm

Name	Peak#	Ret. Time	Area	Area%	Mark
	1	7.805	10286	3.152	M
	2	8.940	316060	96.848	M
	Total		326347	100.000	

PDA Ch5 224nm

Name	Peak#	Ret. Time	Area	Area%	Mark
	1	7.804	5731	3.550	M
	2	8.940	155714	96.450	M
	Total		161444	100.000	

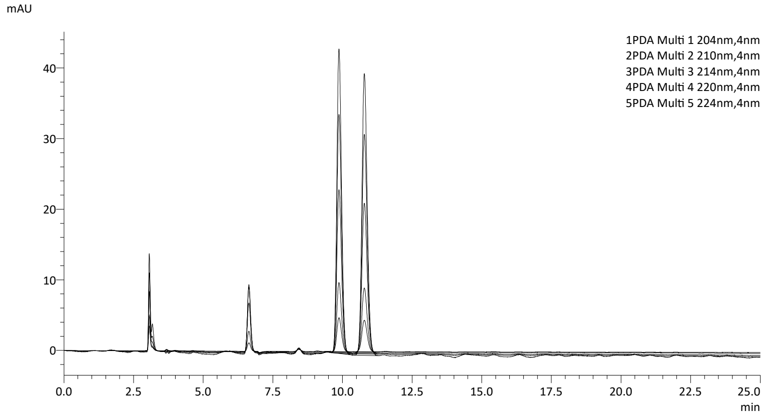

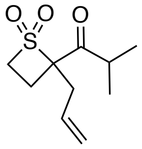

rac - **18r**

Peak Table

PDA Ch1 204nm

Name	Peak#	Ret. Time	Area	Area%	Mark
	1	9.875	510072	48.902	
	2	10.786	532982	51.098	
	Total		1043055	100.000	

PDA Ch2 210nm

Name	Peak#	Ret. Time	Area	Area%	Mark
	1	9.875	400359	49.132	
	2	10.786	414498	50.868	
	Total		814857	100.000	

PDA Ch3 214nm

Name	Peak#	Ret. Time	Area	Area%	Mark
	1	9.875	273713	49.159	
	2	10.786	283080	50.841	
	Total		556793	100.000	

PDA Ch4 220nm

Name	Peak#	Ret. Time	Area	Area%	Mark
	1	9.875	116359	48.900	
	2	10.786	121596	51.100	
	Total		237955	100.000	

PDA Ch5 224nm

Name	Peak#	Ret. Time	Area	Area%	Mark
	1	9.875	56386	48.408	
	2	10.785	60093	51.592	
	Total		116479	100.000	

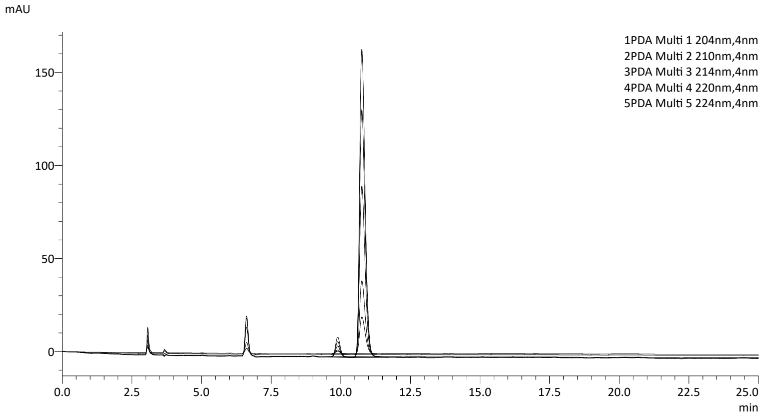

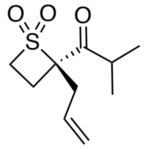

18r
(90% ee)

Peak Table

PDA Ch1 204nm

Name	Peak#	Ret. Time	Area	Area%	Mark
	1	9.888	131859	5.374	
	2	10.756	2321636	94.626	
	Total		2453495	100.000	

PDA Ch2 210nm

Name	Peak#	Ret. Time	Area	Area%	Mark
	1	9.889	99888	5.125	
	2	10.756	1849194	94.875	
	Total		1949082	100.000	

PDA Ch3 214nm

Name	Peak#	Ret. Time	Area	Area%	Mark
	1	9.888	67180	5.042	
	2	10.757	1265167	94.958	
	Total		1332347	100.000	

PDA Ch4 220nm

Name	Peak#	Ret. Time	Area	Area%	Mark
	1	9.889	28059	4.851	
	2	10.757	550323	95.149	
	Total		578382	100.000	

PDA Ch5 224nm

Name	Peak#	Ret. Time	Area	Area%	Mark
	1	9.889	13422	4.613	
	2	10.759	277528	95.387	
	Total		290950	100.000	

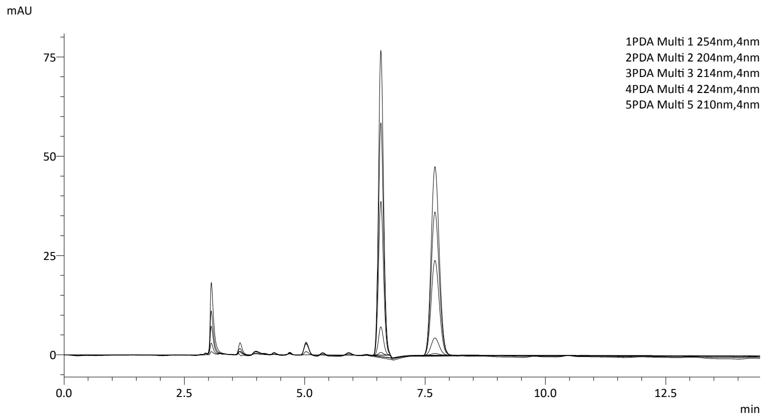

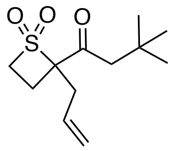

rac - **18s**

Peak Table

PDA Ch1 254nm

Name	Peak#	Ret. Time	Area	Area%	Mark
	1	6.579	4807	50.259	M
	2	7.705	4758	49.741	
	Total		9565	100.000	

PDA Ch2 204nm

Name	Peak#	Ret. Time	Area	Area%	Mark
	1	6.579	580520	50.374	
	2	7.706	571901	49.626	
	Total		1152421	100.000	

PDA Ch3 214nm

Name	Peak#	Ret. Time	Area	Area%	Mark
	1	6.579	292768	50.617	
	2	7.706	285636	49.383	
	Total		578405	100.000	

PDA Ch4 224nm

Name	Peak#	Ret. Time	Area	Area%	Mark
	1	6.579	51646	50.033	M
	2	7.706	51578	49.967	
	Total		103223	100.000	

PDA Ch5 210nm

Name	Peak#	Ret. Time	Area	Area%	Mark
	1	6.579	439915	50.392	
	2	7.706	433070	49.608	
	Total		872984	100.000	

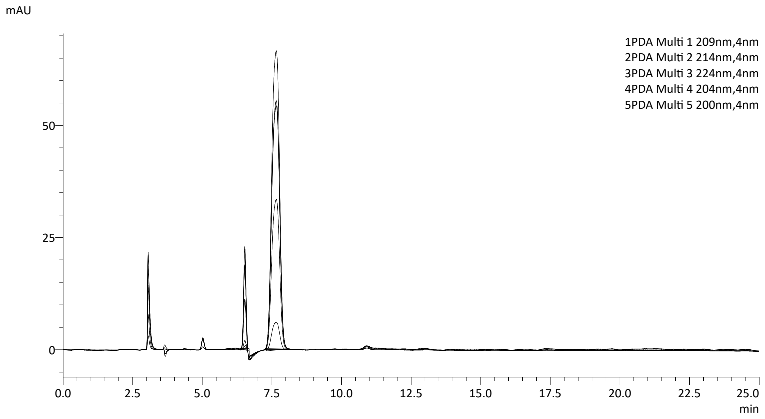

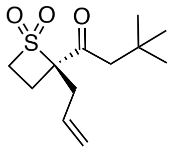

18s
(81% ee)

Peak Table

PDA Ch1 209nm

Name	Peak#	Ret. Time	Area	Area%	Mark
	1	6.527	108839	9.180	M
	2	7.657	1076761	90.820	M
	Total		1185600	100.000	

PDA Ch2 214nm

Name	Peak#	Ret. Time	Area	Area%	Mark
	1	6.527	66355	9.256	M
	2	7.657	650529	90.744	M
	Total		716884	100.000	

PDA Ch3 224nm

Name	Peak#	Ret. Time	Area	Area%	Mark
	1	6.527	11434	8.809	M
	2	7.657	118370	91.191	M
	Total		129804	100.000	

PDA Ch4 204nm

Name	Peak#	Ret. Time	Area	Area%	Mark
	1	6.527	140484	9.692	M
	2	7.657	1308941	90.308	M
	Total		1449424	100.000	

PDA Ch5 200nm

Name	Peak#	Ret. Time	Area	Area%	Mark
	1	6.527	109445	9.319	M
	2	7.656	1065020	90.681	M
	Total		1174466	100.000	

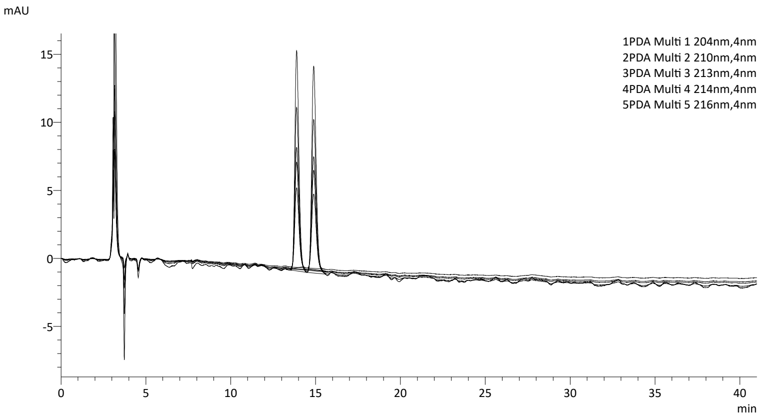

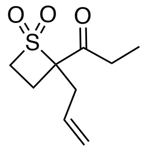

rac - **18t**

Peak Table

PDA Ch1 204nm

Name	Peak#	Ret. Time	Area	Area%	Mark
	1	13.885	273490	49.312	
	2	14.890	281117	50.688	M
	Total		554607	100.000	

PDA Ch2 210nm

Name	Peak#	Ret. Time	Area	Area%	Mark
	1	13.885	192411	49.574	M
	2	14.890	195719	50.426	M
	Total		388130	100.000	

PDA Ch3 213nm

Name	Peak#	Ret. Time	Area	Area%	Mark
	1	13.885	150817	49.481	
	2	14.890	153980	50.519	
	Total		304797	100.000	

PDA Ch4 214nm

Name	Peak#	Ret. Time	Area	Area%	Mark
	1	13.885	131894	49.604	
	2	14.890	134000	50.396	
	Total		265894	100.000	

PDA Ch5 216nm

Name	Peak#	Ret. Time	Area	Area%	Mark
	1	13.885	98283	49.794	
	2	14.890	99096	50.206	
	Total		197380	100.000	

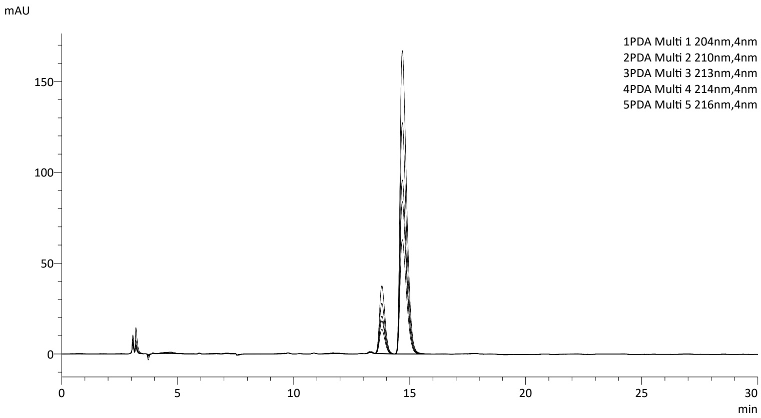

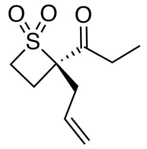

18t
(69% ee)

Peak Table

PDA Ch1 204nm

Name	Peak#	Ret. Time	Area	Area%	Mark
	1	13.801	613790	15.637	
	2	14.684	3311427	84.363	
	Total		3925217	100.000	

PDA Ch2 210nm

Name	Peak#	Ret. Time	Area	Area%	Mark
	1	13.801	455252	15.405	
	2	14.684	2499994	84.595	
	Total		2955246	100.000	

PDA Ch3 213nm

Name	Peak#	Ret. Time	Area	Area%	Mark
	1	13.801	340108	15.343	
	2	14.684	1876575	84.657	
	Total		2216682	100.000	

PDA Ch4 214nm

Name	Peak#	Ret. Time	Area	Area%	Mark
	1	13.801	297077	15.313	
	2	14.684	1642940	84.687	
	Total		1940017	100.000	

PDA Ch5 216nm

Name	Peak#	Ret. Time	Area	Area%	Mark
	1	13.801	221173	15.236	
	2	14.685	1230470	84.764	
	Total		1451643	100.000	

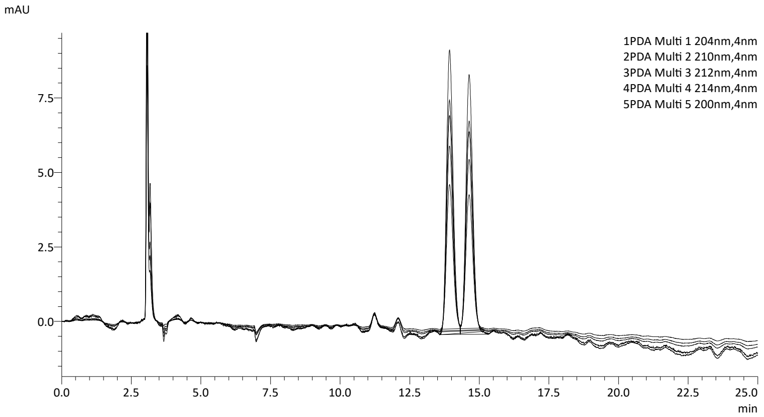

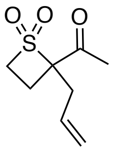

rac - **18u**

Peak Table

PDA Ch1 204nm

Name	Peak#	Ret. Time	Area	Area%	Mark
	1	13.932	159598	51.153	
	2	14.633	152402	48.847	SV
	Total		312000	100.000	

PDA Ch2 210nm

Name	Peak#	Ret. Time	Area	Area%	Mark
	1	13.932	120476	50.988	
	2	14.634	115809	49.012	V
	Total		236285	100.000	

PDA Ch3 212nm

Name	Peak#	Ret. Time	Area	Area%	Mark
	1	13.932	102896	50.890	
	2	14.634	99295	49.110	V
	Total		202191	100.000	

PDA Ch4 214nm

Name	Peak#	Ret. Time	Area	Area%	Mark
	1	13.932	80338	50.821	
	2	14.634	77742	49.179	V
	Total		158080	100.000	

PDA Ch5 200nm

Name	Peak#	Ret. Time	Area	Area%	Mark
	1	13.932	131252	51.697	
	2	14.636	122637	48.303	V
	Total		253889	100.000	

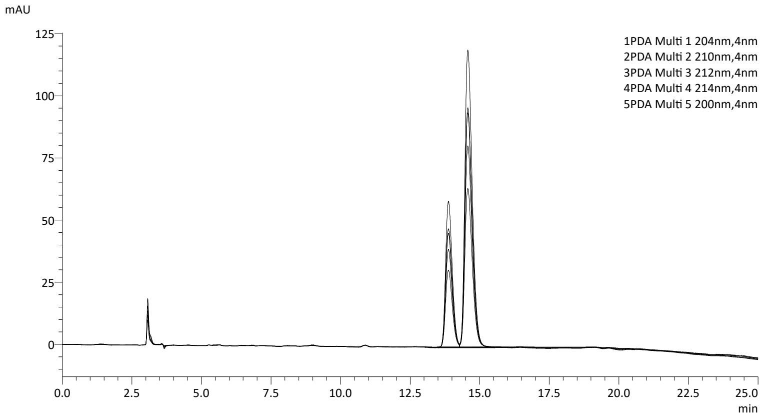

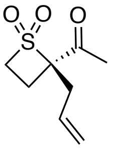

18u
(39% ee)

Peak Table

PDA Ch1 204nm

Name	Peak#	Ret. Time	Area	Area%	Mark
	1	13.877	986666	30.574	
	2	14.574	2240426	69.426	V
	Total		3227093	100.000	

PDA Ch2 210nm

Name	Peak#	Ret. Time	Area	Area%	Mark
	1	13.877	766225	30.415	
	2	14.574	1753044	69.585	V
	Total		2519269	100.000	

PDA Ch3 212nm

Name	Peak#	Ret. Time	Area	Area%	Mark
	1	13.877	656124	30.399	
	2	14.574	1502233	69.601	V
	Total		2158357	100.000	

PDA Ch4 214nm

Name	Peak#	Ret. Time	Area	Area%	Mark
	1	13.877	515404	30.373	
	2	14.574	1181515	69.627	V
	Total		1696919	100.000	

PDA Ch5 200nm

Name	Peak#	Ret. Time	Area	Area%	Mark
	1	13.877	801478	30.661	
	2	14.574	1812529	69.339	V
	Total		2614007	100.000	

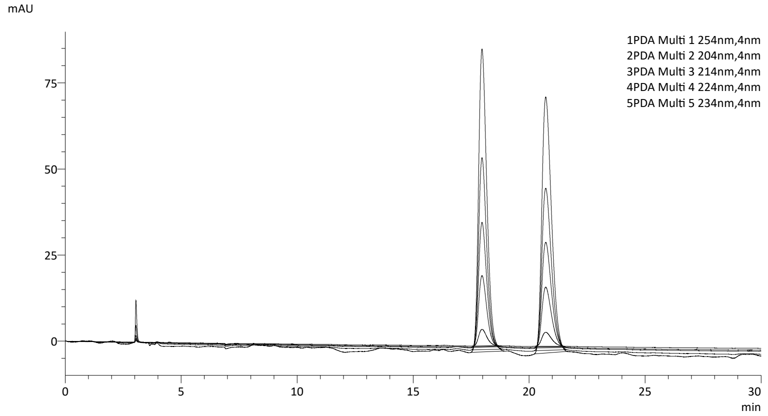

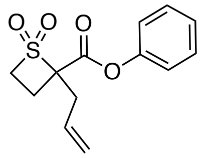

rac - **19a**

Peak Table

PDA Ch1 254nm

Name	Peak#	Ret. Time	Area	Area%	Mark
	1	17.972	118769	49.767	M
	2	20.718	119879	50.233	M
	Total		238648	100.000	

PDA Ch2 204nm

Name	Peak#	Ret. Time	Area	Area%	Mark
	1	17.970	2204977	50.350	
	2	20.718	2174337	49.650	
	Total		4379314	100.000	

PDA Ch3 214nm

Name	Peak#	Ret. Time	Area	Area%	Mark
	1	17.970	1379377	50.139	
	2	20.717	1371725	49.861	
	Total		2751102	100.000	

PDA Ch4 224nm

Name	Peak#	Ret. Time	Area	Area%	Mark
	1	17.970	891161	50.088	
	2	20.717	888030	49.912	
	Total		1779191	100.000	

PDA Ch5 234nm

Name	Peak#	Ret. Time	Area	Area%	Mark
	1	17.971	502620	50.136	
	2	20.718	499900	49.864	
	Total		1002521	100.000	

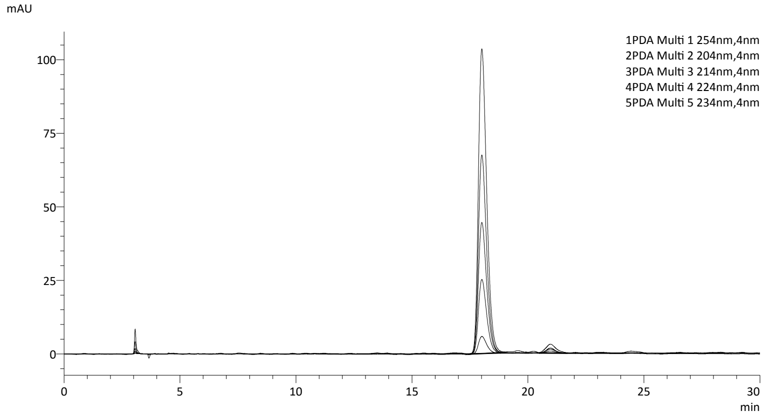

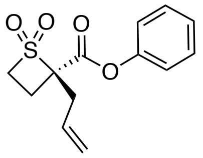

19a
(94% ee)

Peak Table

PDA Ch1 254nm

Name	Peak#	Ret. Time	Area	Area%	Mark
	1	18.016	146111	97.564	
	2	20.951	3649	2.436	M
	Total		149760	100.000	

PDA Ch2 204nm

Name	Peak#	Ret. Time	Area	Area%	Mark
	1	18.014	2587770	96.762	
	2	20.967	86591	3.238	
	Total		2674362	100.000	

PDA Ch3 214nm

Name	Peak#	Ret. Time	Area	Area%	Mark
	1	18.014	1681418	96.972	
	2	20.973	52502	3.028	
	Total		1733920	100.000	

PDA Ch4 224nm

Name	Peak#	Ret. Time	Area	Area%	Mark
	1	18.014	1104487	97.041	
	2	20.970	33677	2.959	
	Total		1138163	100.000	

PDA Ch5 234nm

Name	Peak#	Ret. Time	Area	Area%	Mark
	1	18.015	623865	97.355	
	2	20.972	16950	2.645	
	Total		640814	100.000	

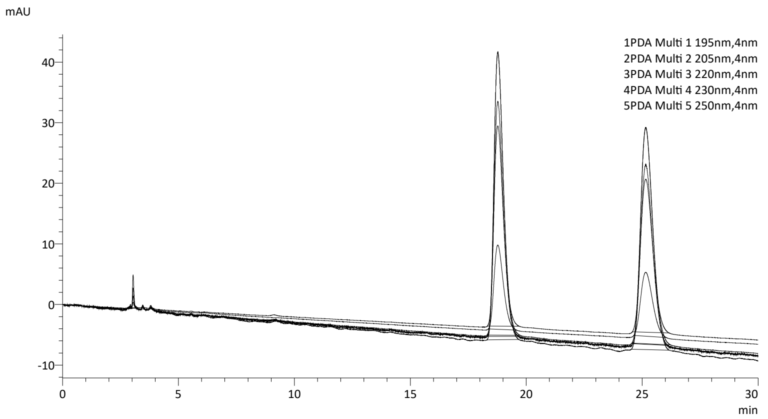

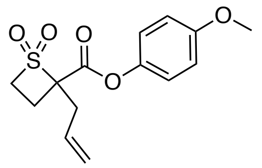

rac - **19b**

Peak Table

PDA Ch1 195nm

Name	Peak#	Ret. Time	Area	Area%	Mark
	1	18.774	1116103	50.609	
	2	25.154	1089259	49.391	
	Total		2205362	100.000	

PDA Ch2 205nm

Name	Peak#	Ret. Time	Area	Area%	Mark
	1	18.776	1369803	50.170	
	2	25.155	1360518	49.830	
	Total		2730322	100.000	

PDA Ch3 220nm

Name	Peak#	Ret. Time	Area	Area%	Mark
	1	18.775	1344197	50.254	
	2	25.156	1330613	49.746	
	Total		2674810	100.000	

PDA Ch4 230nm

Name	Peak#	Ret. Time	Area	Area%	Mark
	1	18.776	951091	50.432	
	2	25.156	934806	49.568	
	Total		1885897	100.000	

PDA Ch5 250nm

Name	Peak#	Ret. Time	Area	Area%	Mark
	1	18.775	394314	50.858	
	2	25.157	381009	49.142	
	Total		775324	100.000	

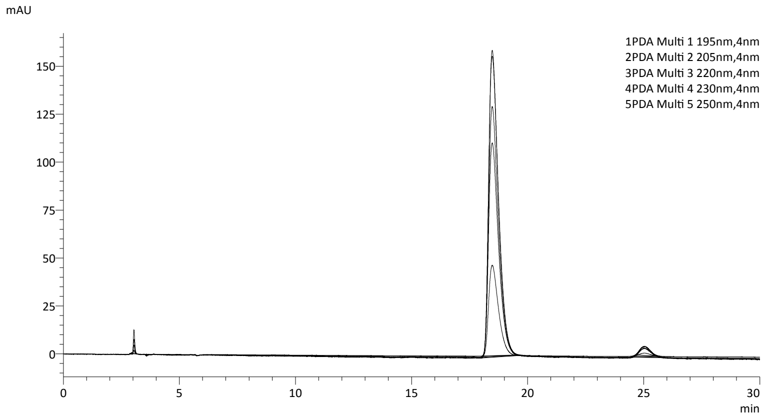

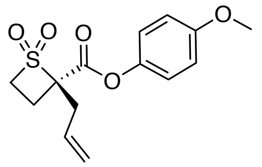

19b
(93% ee)

Peak Table

PDA Ch1 195nm

Name	Peak#	Ret. Time	Area	Area%	Mark
	1	18.478	3887464	95.842	
	2	25.008	168653	4.158	M
	Total		4056117	100.000	

PDA Ch2 205nm

Name	Peak#	Ret. Time	Area	Area%	Mark
	1	18.477	4753423	96.314	
	2	25.035	181895	3.686	
	Total		4935319	100.000	

PDA Ch3 220nm

Name	Peak#	Ret. Time	Area	Area%	Mark
	1	18.477	4648311	96.270	
	2	25.030	180080	3.730	
	Total		4828392	100.000	

PDA Ch4 230nm

Name	Peak#	Ret. Time	Area	Area%	Mark
	1	18.477	3291626	96.749	
	2	25.035	110610	3.251	
	Total		3402236	100.000	

PDA Ch5 250nm

Name	Peak#	Ret. Time	Area	Area%	Mark
	1	18.477	1389353	96.358	
	2	25.030	52520	3.642	M
	Total		1441873	100.000	

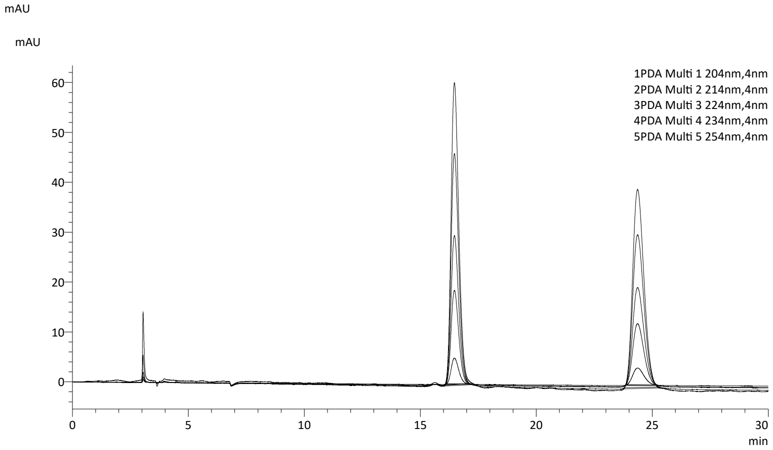

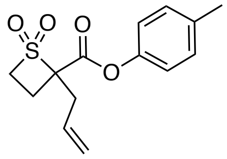

rac - **19c**

Peak Table

PDA Ch1 204nm

Name	Peak#	Ret. Time	Area	Area%	Mark
	1	16.472	1402878	50.674	
	2	24.368	1365541	49.326	
	Total		2768419	100.000	

PDA Ch2 214nm

Name	Peak#	Ret. Time	Area	Area%	Mark
	1	16.472	1076161	50.680	
	2	24.369	1047299	49.320	
	Total		2123460	100.000	

PDA Ch3 224nm

Name	Peak#	Ret. Time	Area	Area%	Mark
	1	16.472	688309	50.779	
	2	24.369	667182	49.221	
	Total		1355491	100.000	

PDA Ch4 234nm

Name	Peak#	Ret. Time	Area	Area%	Mark
	1	16.472	430723	51.200	
	2	24.368	410529	48.800	
	Total		841252	100.000	

PDA Ch5 254nm

Name	Peak#	Ret. Time	Area	Area%	Mark
	1	16.474	116405	50.049	M
	2	24.364	116176	49.951	M
	Total		232582	100.000	

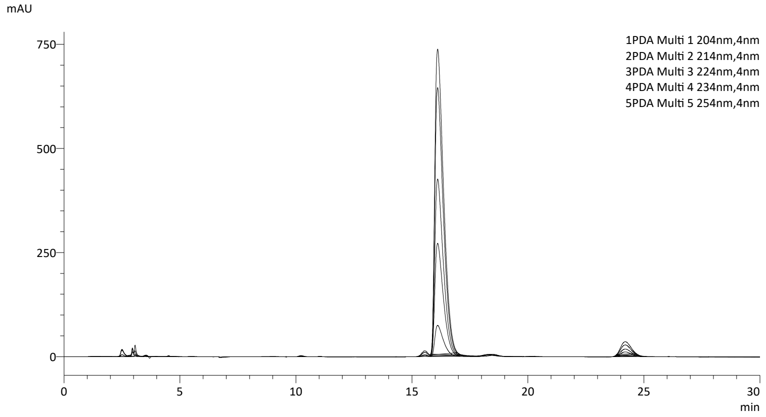

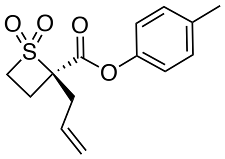

19c
(92% ee)

Peak Table

PDA Ch1 204nm

Name	Peak#	Ret. Time	Area	Area%	Mark
	1	16.110	19540471	96.012	M
	2	24.198	811724	3.988	M
	Total		20352195	100.000	

PDA Ch2 214nm

Name	Peak#	Ret. Time	Area	Area%	Mark
	1	16.110	16258932	95.970	M
	2	24.198	682709	4.030	M
	Total		16941642	100.000	

PDA Ch3 224nm

Name	Peak#	Ret. Time	Area	Area%	Mark
	1	16.110	10754885	95.792	M
	2	24.199	472486	4.208	M
	Total		11227371	100.000	

PDA Ch4 234nm

Name	Peak#	Ret. Time	Area	Area%	Mark
	1	16.110	6878844	95.587	M
	2	24.197	317575	4.413	M
	Total		7196419	100.000	

PDA Ch5 254nm

Name	Peak#	Ret. Time	Area	Area%	Mark
	1	16.110	1913582	95.861	M
	2	24.196	82620	4.139	M
	Total		1996202	100.000	

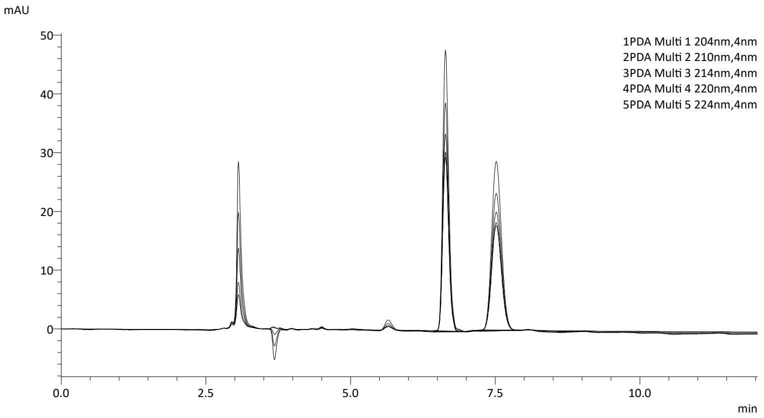

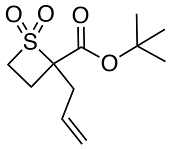

rac - **19d**

Peak Table

PDA Ch1 204nm

Name	Peak#	Ret. Time	Area	Area%	Mark
	1	6.641	353330	50.597	
	2	7.519	344990	49.403	
	Total		698320	100.000	

PDA Ch2 210nm

Name	Peak#	Ret. Time	Area	Area%	Mark
	1	6.641	285038	50.529	
	2	7.519	279073	49.471	
	Total		564111	100.000	

PDA Ch3 214nm

Name	Peak#	Ret. Time	Area	Area%	Mark
	1	6.641	244648	50.372	
	2	7.519	241031	49.628	
	Total		485679	100.000	

PDA Ch4 220nm

Name	Peak#	Ret. Time	Area	Area%	Mark
	1	6.641	221339	50.321	
	2	7.519	218516	49.679	
	Total		439855	100.000	

PDA Ch5 224nm

Name	Peak#	Ret. Time	Area	Area%	Mark
	1	6.641	214948	50.314	
	2	7.519	212266	49.686	
	Total		427214	100.000	

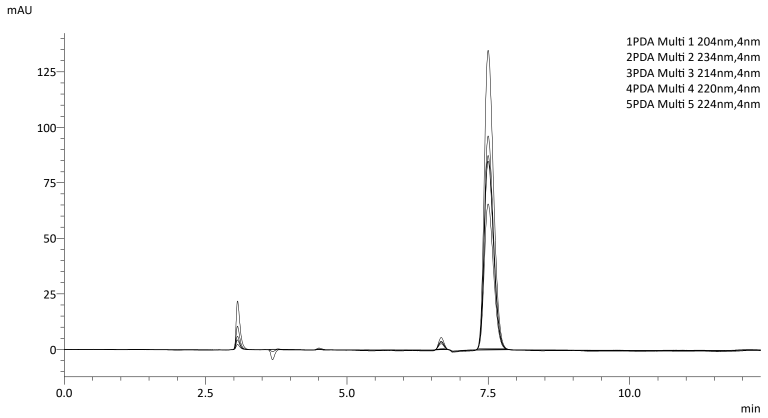

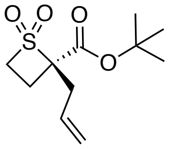

19d
(96% ee)

Peak Table

PDA Ch1 204nm

Name	Peak#	Ret. Time	Area	Area%	Mark
	1	6.666	35386	2.235	M
	2	7.498	1547909	97.765	M
	Total		1583295	100.000	

PDA Ch2 234nm

Name	Peak#	Ret. Time	Area	Area%	Mark
	1	6.666	15970	2.089	M
	2	7.498	748621	97.911	M
	Total		764591	100.000	

PDA Ch3 214nm

Name	Peak#	Ret. Time	Area	Area%	Mark
	1	6.667	21905	1.947	M
	2	7.498	1103268	98.053	M
	Total		1125172	100.000	

PDA Ch4 220nm

Name	Peak#	Ret. Time	Area	Area%	Mark
	1	6.667	23819	2.329	M
	2	7.498	998693	97.671	M
	Total		1022512	100.000	

PDA Ch5 224nm

Name	Peak#	Ret. Time	Area	Area%	Mark
	1	6.667	18222	1.872	M
	2	7.498	955179	98.128	M
	Total		973401	100.000	

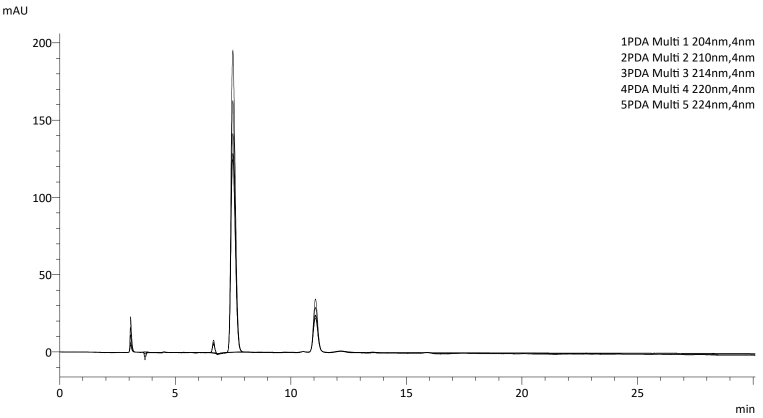

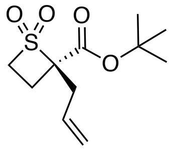

19d [5 g]
(96% ee)

Peak Table

PDA Ch1 204nm

Name	Peak#	Ret. Time	Area	Area%	Mark
	1	6.655	58560	2.212	M
	2	7.489	2589175	97.788	M
	Total		2647735	100.000	

PDA Ch2 210nm

Name	Peak#	Ret. Time	Area	Area%	Mark
	1	6.655	47693	2.182	M
	2	7.489	2137729	97.818	M
	Total		2185422	100.000	

PDA Ch3 214nm

Name	Peak#	Ret. Time	Area	Area%	Mark
	1	6.655	42009	2.213	M
	2	7.489	1855891	97.787	M
	Total		1897900	100.000	

PDA Ch4 220nm

Name	Peak#	Ret. Time	Area	Area%	Mark
	1	6.655	38218	2.217	M
	2	7.489	1685576	97.783	M
	Total		1723794	100.000	

PDA Ch5 224nm

Name	Peak#	Ret. Time	Area	Area%	Mark
	1	6.655	37170	2.228	M
	2	7.489	1630876	97.772	M
	Total		1668045	100.000	

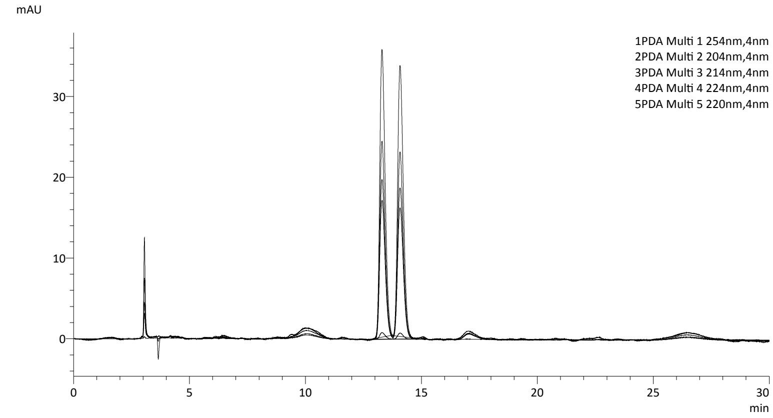

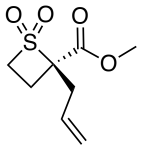

rac - **19e**

Peak Table

PDA Ch1 254nm

Name	Peak#	Ret. Time	Area	Area%	Mark
	1	13.302	13149	50.801	
	2	14.085	12734	49.199	
	Total		25883	100.000	

PDA Ch2 204nm

Name	Peak#	Ret. Time	Area	Area%	Mark
	1	13.302	579808	50.094	M
	2	14.080	577626	49.906	
	Total		1157434	100.000	

PDA Ch3 214nm

Name	Peak#	Ret. Time	Area	Area%	Mark
	1	13.302	404201	49.997	
	2	14.081	404246	50.003	V
	Total		808447	100.000	

PDA Ch4 224nm

Name	Peak#	Ret. Time	Area	Area%	Mark
	1	13.302	283289	49.951	
	2	14.080	283850	50.049	V
	Total		567139	100.000	

PDA Ch5 220nm

Name	Peak#	Ret. Time	Area	Area%	Mark
	1	13.302	326160	49.956	
	2	14.080	326728	50.044	V
	Total		652888	100.000	

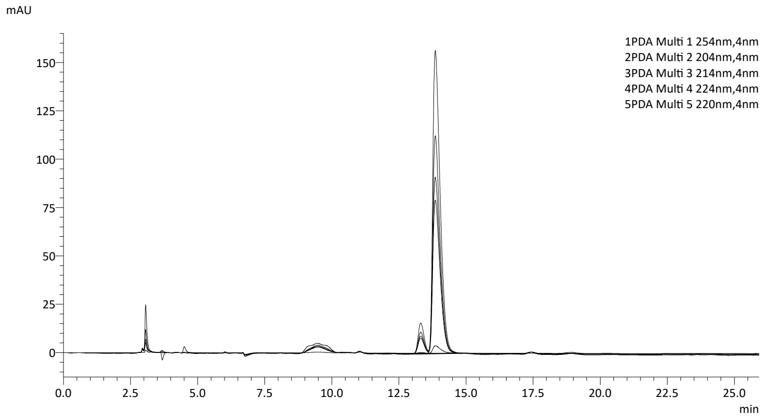

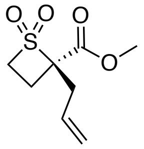

19e
(85% ee)

Peak Table

PDA Ch1 254nm

Name	Peak#	Ret. Time	Area	Area%	Mark
	1	13.320	5179	6.213	
	2	13.864	78181	93.787	V
	Total		83359	100.000	

PDA Ch2 204nm

Name	Peak#	Ret. Time	Area	Area%	Mark
	1	13.315	250298	7.662	
	2	13.859	3016499	92.338	V
	Total		3266797	100.000	

PDA Ch3 214nm

Name	Peak#	Ret. Time	Area	Area%	Mark
	1	13.316	173473	7.484	
	2	13.859	2144414	92.516	V
	Total		2317888	100.000	

PDA Ch4 224nm

Name	Peak#	Ret. Time	Area	Area%	Mark
	1	13.316	122151	7.514	
	2	13.859	1503494	92.486	V
	Total		1625645	100.000	

PDA Ch5 220nm

Name	Peak#	Ret. Time	Area	Area%	Mark
	1	13.316	140235	7.493	
	2	13.859	1731355	92.507	V
	Total		1871590	100.000	

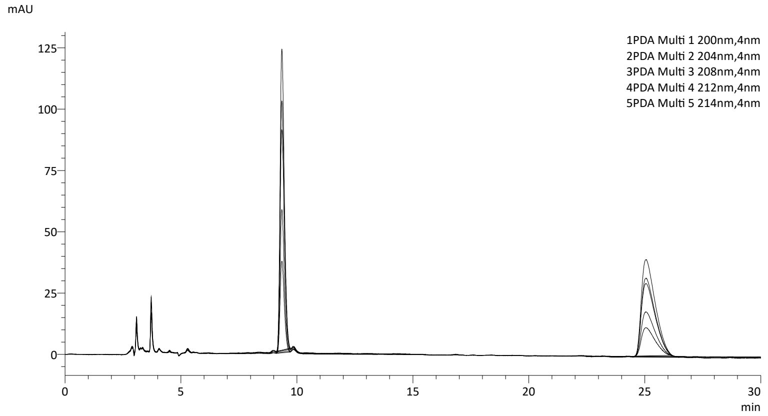

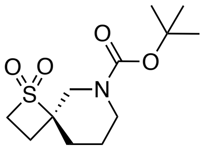

rac - **28d**

Peak Table

PDA Ch1 200nm

Name	Peak#	Ret. Time	Area	Area%	Mark
	1	9.353	1159328	48.482	M
	2	25.049	1231908	51.518	M
	Total		2391237	100.000	

PDA Ch2 204nm

Name	Peak#	Ret. Time	Area	Area%	Mark
	1	9.353	1582097	48.122	
	2	25.050	1705548	51.878	
	Total		3287645	100.000	

PDA Ch3 208nm

Name	Peak#	Ret. Time	Area	Area%	Mark
	1	9.353	1296467	48.593	
	2	25.051	1371568	51.407	
	Total		2668035	100.000	

PDA Ch4 212nm

Name	Peak#	Ret. Time	Area	Area%	Mark
	1	9.353	734960	48.663	
	2	25.050	775333	51.337	
	Total		1510293	100.000	

PDA Ch5 214nm

Name	Peak#	Ret. Time	Area	Area%	Mark
	1	9.353	470930	48.656	
	2	25.051	496945	51.344	M
	Total		967875	100.000	

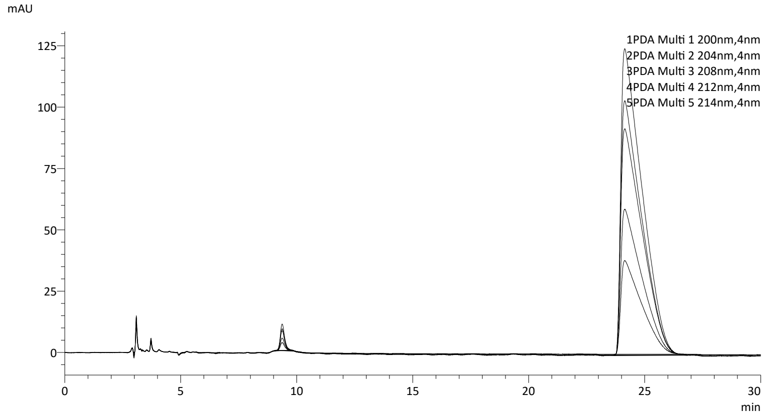

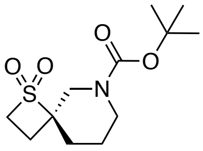

28d
(96% ee)

Peak Table

PDA Ch1 200nm

Name	Peak#	Ret. Time	Area	Area%	Mark
	1	9.373	104059	1.723	M
	2	24.142	5935117	98.277	S
	Total		6039176	100.000	

PDA Ch2 204nm

Name	Peak#	Ret. Time	Area	Area%	Mark
	1	9.373	139485	1.721	M
	2	24.141	7966322	98.279	
	Total		8105806	100.000	

PDA Ch3 208nm

Name	Peak#	Ret. Time	Area	Area%	Mark
	1	9.373	127435	1.919	M
	2	24.141	6514948	98.081	
	Total		6642383	100.000	

PDA Ch4 212nm

Name	Peak#	Ret. Time	Area	Area%	Mark
	1	9.374	77510	2.050	M
	2	24.141	3704358	97.950	
	Total		3781868	100.000	

PDA Ch5 214nm

Name	Peak#	Ret. Time	Area	Area%	Mark
	1	9.374	43668	1.801	M
	2	24.142	2380694	98.199	
	Total		2424362	100.000	

8. References

1 G. W. Gokel, H. M. Gerdes and D. M. Dishong, *J. Org. Chem.*, 1980, **45**, 3634–3639.

2 Y. Choi, T. Kim, S. Jang and J. Kang, *New J. Chem.*, 2016, **40**, 794–802.

3 A. R. Katritzky, N. Shobana, J. Pernak, A. S. Afridi and W.-Q. Fan, *Tetrahedron*, 1992, **48**, 7817–7822.

4 G. Fontana, A. Lubineau and M.-C. Scherrmann, *Org. Biomol. Chem.*, 2005, **3**, 1375–1380.

5 J. K. Laha and S. Sharma, *ACS Omega*, 2018, **3**, 4860–4870.
